# Design, Synthesis, and Biological Activity of Boron-Bearing Sugar Derivatives for Boron Neutron Capture Therapy (BNCT)

**DOI:** 10.3390/molecules31081230

**Published:** 2026-04-08

**Authors:** Mengyan Hou, Xia Li, Yan Li, Wenhao Shi, Haotian Tang, Fang Feng, Xuan Wan, Hua Xie, Guilong Zhao

**Affiliations:** 1 School of Chinese Materia Medica, Nanjing University of Chinese Medicine, Nanjing 210023, China; houmengyan1062@zidd.ac.cn (M.H.); lixia1063@zidd.ac.cn (X.L.); 2 Zhongshan Institute for Drug Discovery, Shanghai Institute of Materia Medica, Chinese Academy of Sciences, Zhongshan 528400, China; shiwenhao@zidd.ac.cn (W.S.); tanghaotian18@mails.ucas.ac.cn (H.T.); 3 Shanghai Institute of Materia Medica, Chinese Academy of Sciences, Shanghai 201203, China; yanli@simm.ac.cn (Y.L.); fengfang@simm.ac.cn (F.F.); wanxuan@simm.ac.cn (X.W.)

**Keywords:** boron neutron capture therapy, BNCT, GLUT1, carbohydrate, sugar

## Abstract

Radiotherapy is one of the conventional methods for the treatment of cancers. Boron neutron capture therapy (BNCT) has emerged as a promising and well-recognized modality for treating certain types of cancers. BNCT is a binary radiotherapy that largely depends on neutron beams and ^10^B carriers. Although an “ideal” boron carrier should fulfill multiple criteria, high tumor/normal tissue ratio (T/N > 5) and high tumor uptake of boron (>20 μg/g) are critically important. First-generation (boric acid and derivatives) and second-generation (BPA and BSH) boron carriers suffer from poor T/N and extremely high dose in clinical use (500 mg/kg and usually >30 g for each patient). Glucose transporter 1 (GLUT1) is overexpressed on the membrane surface of multiple tumors and is a potential target for third-generation boron carrier to achieve high T/N and high tumor uptake of boron. However, the boron-bearing sugar derivatives designed in the last few decades have suffered from suboptimal T/N values and significant cytotoxicity. In the present study, a total of two categories comprising 6 series (28 in total) of boron-bearing sugar derivatives were designed and synthesized and their cellular boron uptake, T/N, and cytotoxicity were evaluated. The structure–activity relationship (SAR) of these target compounds was analyzed, and one of the target compounds, **B3**, a phenyl *C*-mannoside with an *o*-carborane moiety, exhibited the best boron-carrying profile, which featured 10.6-fold higher boron uptake by the SCC-9 cell line and a largely improved T/N (3.3 for **B3** vs. 1.4 for BPA) compared with the current clinical gold standard BPA. Therefore, the chemical structure of **B3** represents a privileged candidate structure for the future design of “ideal” boron carriers for BNCT.

## 1. Introduction

Cancer is one of the leading causes of death in the world. Radiotherapy is one of the conventional methods for the treatment of cancers. Boron neutron capture therapy (BNCT) has emerged as a promising and well-recognized modality for the treatment of certain types of cancers, including melanoma, glioma, and head and neck cancers [1]. BNCT is a binary cancer radiotherapy. A carrier that contains non-radioactive ^10^B is first administered to patients, which is selectively absorbed by and accumulated in cancer cells; subsequently, the cancer cells with ^10^B are irradiated with a thermal or epithermal neutron beam to induce neutron capture and fission reaction (^10^B + ^1^n → [^11^B]* → ^4^He + ^7^Li) and the generated α particles (^4^He) and γ rays from the de-excitation of recoiling ^7^Li ion cause DNA damage and cancer cell death. The α particle has a travel distance of <10 μm, approximately the diameter of one single cancer cell, and therefore BNCT therapy can selectively kill cancer cells, sparing the adjacent normal tissues [2]. Historically, the neutron beam used in BNCT came from a fission reactor, which was profoundly inconvenient for treatment; recently, clinical BNCT in hospital was mainly performed using accelerator-based sources, which are easier to install and more cost-effective [2].

**Figure 1:**
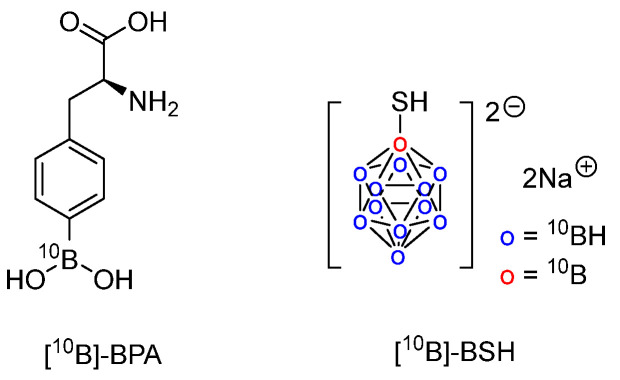
The chemical structures of BPA and BSH.

The ^10^B carrier compound has remained the mainstay of BNCT therapy. An “ideal” ^10^B carrier compound should fulfill multiple criteria, such as rational water solubility, low systemic toxicity, high tumor/normal tissue ratio (T/N > 5), high tumor/blood ratio (T/B > 3.5), high tumor uptake of ^10^B (>20 μg/g), long retention of ^10^B in tumor cells (several hours), and rapid clearance from the body after therapy [3,4]. Among these criteria, high tumor specificity (T/N and T/B ratios) and high ^10^B tumor uptake are of crucial importance for a ^10^B carrier compound to achieve highly selective uptake by tumor cells and then accumulation in tumor tissues compared with normal adjacent tissues. [^10^B] boric acid and its derivatives, as the “first-generation” ^10^B carrier compounds, were studied from the 1950s to the 1960s with moderate efficacy, but they lacked tumor selectivity and therefore were discontinued later [1]. The “second-generation” carriers were then developed and became popular in clinical use in the 1960s, including sodium borocaptate (BSH, Na_2_B_12_H_11_SH) and (L)-4-boronophenylalanine (BPA) (Figure 1). BSH features good water solubility, high capacity for transporting ^10^B atoms (12 ^10^B atoms for each BSH molecule), and low systemic toxicity but suffers from no tumor selectivity and being expensive; it is now rarely available in the clinical setting [5]. BPA is the only well-recognized and widely used BNCT carrier today, which is highlighted by its tumor selectivity (T/N = 2.5–3.3) and being relatively cheap, it but suffers from low water solubility and low capacity for transporting ^10^B atoms (only one ^10^B atom for each BPA molecule). BPA has proven to be efficacious in the treatment of melanoma and head and neck cancers (among others); however, its low T/N and low ^10^B-carrying capacity necessitate an extremely high dose in clinical use (500 mg/kg and usually >30 g for each patient depending on body weight) [6,7].

**Figure 2:**
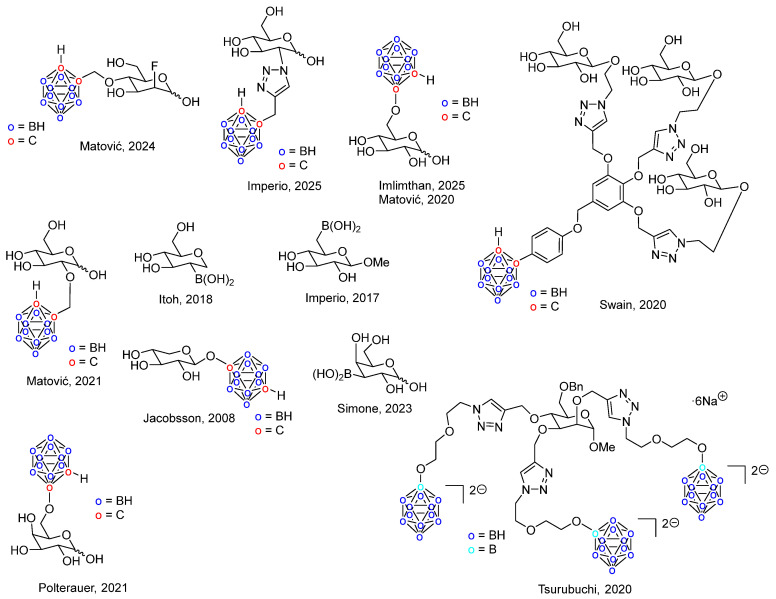
Representative boron-bearing sugar derivatives for BNCT reported in earlier work [15,16,17,18,19,20,21,22,23,24,25,26].

**Figure 3:**
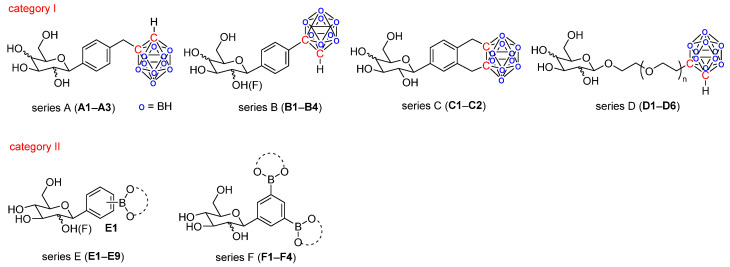
Design of the boron-bearing sugar derivatives in the present study.

To overcome the shortcomings associated with “second-generation” ^10^B carriers, the last few decades have witnessed substantial efforts to develop “third-generation” carriers to enhance their tumor targeting ability, which are based on a variety of modalities, including new chemical entities (nanotechnology-based delivery systems) and cancer-specific biochemical and physiological processes (boron-bearing sugar derivatives, polyamines, peptides, DNA intercalators and monoclonal antibodies) [8,9,10,11]. Among these, boron-bearing sugar derivatives constitute an important class of boron carriers because they are the potential substrates of glucose transporter 1 (GLUT1) due to their structural similarity to the natural substrate of GLUT1 (D-glucose). GLUT1 is a key rate-limiting transporter in the transportation of glucose in cancer cells for energy supply, and it is overexpressed on the membrane surface of multiple tumors [12,13,14]. Furthermore, incorporation of sugar moiety can dramatically increase water solubility. Some representative boron-bearing sugar derivatives as potential BNCT carriers reported in earlier work are summarized in Figure 2 [15,16,17,18,19,20,21,22,23,24,25,26]. Despite some advantages, such as high capacity for transporting boron [15,16,17,20,22,25] mainly due to the incorporation of *o*-carborane or *closo*-dodecaborane moiety and enhanced water solubility [16,23] compared with BSH and BPA, most of the reported boron-bearing sugar derivatives are associated with multiple unfavorable properties required for BNCT, such as high plasma protein binding [15], suboptimal T/N or T/B values [15,16,17,18,19,20,21,22,23,24], and pronounced cytotoxicity, particularly at high concentrations [17,18,20,23]. Inspired by these observations, we designed and synthesized a total of two categories comprising 6 series (28 in total) of boron-bearing sugars (Figure 3) to explore novel chemical structures for enhanced tumor-targeting ability and evaluated their cellular boron uptake in normal and high GLUT1-expressing cancer cells as well as their cytotoxicity. The structure–activity relationship (SAR) of the target compounds was analyzed, and one of the target compounds, **B3**, displayed the best boron-carrying profile, with 10.6-fold higher boron uptake by the SCC-9 cell line and a largely improved T/N (3.3 for **B3** vs. 1.4 for BPA) compared with BPA. The findings in the present study, especially the chemical structure of **B3**, should be helpful for the future design of “ideal” boron carriers for BNCT.

## 2. Results and Discussion

### 2.1. Chemistry

#### 2.1.1. Synthesis of Intermediates

**Scheme 1:**
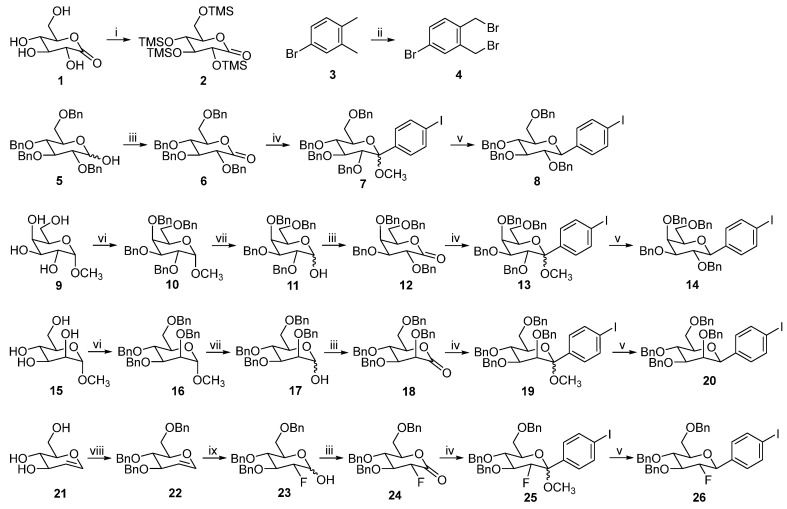
Synthetic route to intermediates **2**, **4**, **8**, **14**, **20,** and **26**. Reagents and conditions: (i) TMSCl, NMM, DMAP, dried THF, 0 °C–r.t., N_2_, 68%; (ii) NBS/AIBN, dried CCl_4_, reflux, N_2_, 37%; (iii) Ac_2_O/DMSO, 0 °C–r.t., N_2_, 95% for **6**, 30% for **12** (over 2 steps from **10**), 35% for **18** (over 2 steps from **16**), 17% for **24** (over 2 steps from **22**); (iv) (a) 1,4-diiodobenzene (1.2 eq), *n*-BuLi (1.2 eq), dried THF, −78 °C, N_2_, (b) **6**, **12**, **18,** or **24** (1 eq), dried THF, −78 °C, N_2_, c) MeSO_3_H, MeOH, −78 °C–r.t., N_2_; (v) Et_3_SiH/BF_3_·Et_2_O, dried CH_2_Cl_2_, −35 °C–r.t., N_2_, 27% for **8** (over 2 steps from **6**), 48% for **14** (over 2 steps from **12**), 26% for **20** (over 2 steps from **18**), 13% for **26** (over 2 steps from **24**); (vi) BnBr, NaH, dried THF/dried DMF, 0 °C–r.t., N_2_, 91% for **10**, 77% for **16**; (vii) 5 M HCl, AcOH, 80 °C, N_2_; (viii) BnBr, NaH, TBAI, dried THF, 0 °C–r.t., N_2_, 51%; (ix) selectfluor, DMF/H_2_O (1/1 by *v*/*v*), 50 °C.

The synthetic routes to some intermediates are shown in Scheme 1. Treatment of commercially available D-glucono-δ-lactone (**1**) with trimethylsilyl chloride (TMSCl) in the presence of 4-dimethylaminopyridine (DMAP) and *N*-methylmorpholine (NMM) in THF afforded pertrimethylsilylated lactone **2** [26]. Treatment of commercially available 4-bromo-1,2-dimethylbenzene (**3**) with *N*-bromosuccinimide (NBS) in the presence of 2,2′-azobisisobutyronitrile (AIBN) as an initiator in refluxing CCl_4_ produced bisbenzyl bromide **4** through free radical substitution [27]. Commercially available tetrabenzylated D-glucose lactol **5** was oxidized to corresponding lactone **6** with DMSO/Ac_2_O at room temperature [28]. 1,4-Diiodobenzene was treated with 1 eq of *n*-BuLi to undergo iodine–lithium exchange to furnish 4-iodophenyl lithium, and the latter was trapped with lactone **6** to give a 1-*C*-aryl lactol intermediate, which gave corresponding 1-*C*-aryl methyl glucopyranoside **7** upon treatment with MeOH in the presence of MeSO_3_H; BF_3_·Et_2_O-mediated reduction of **7** with Et_3_SiH produced *C*-aryl glucopyranoside **8** [29]. Perbenzylation of commercially available methyl α-D-galactopyranoside (**9**) with BnBr in the presence of NaH afforded **10**. Selective acidic hydrolysis of the anomeric methyl galactoside moiety with 5 M HCl in AcOH at 80 °C liberated the anomeric OH to give lactol **11** [30], which was oxidized to corresponding lactone **12** with DMSO/Ac_2_O. Following the procedure identical to the conversion of **6** to **8** discussed above, **12** was converted to **14**. Following the same procedure as the conversion of **9** to **14**, commercially available methyl α-D-mannopyranoside (**15**) was converted to *C*-aryl mannoside **20**. Commercially available glycal **21** was perbenzylated to **22** through treatment with BnBr in the presence of NaH and tetra-*n*-butylammonium iodide (TBAI) in THF. Treatment of **22** with 1-chloromethyl-4-fluoro-1,4-diazoniabicyclo [2.2.2]octane bis(tetrafluoroborate) (selectfluor^®^) in DMF/H_2_O at 50 °C afforded 2-deoxy-2-F-glucose lactol **23** [31], which was converted to F-bearing intermediate **26,** as described above.

#### 2.1.2. Synthesis of Series A

**Scheme 2:**
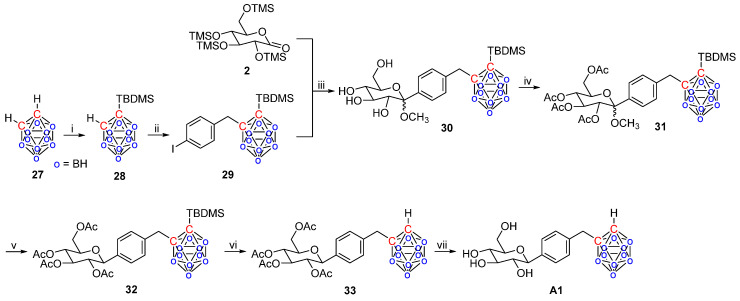
Synthetic route to target compound **A1**. Reagents and conditions: (i) (a) *n*-BuLi (1.2 eq), dried THF, 0 °C–r.t., N_2_, (b) TBDMSCl (1.3 eq), dried THF, 0 °C–r.t., N_2_, 81%; (ii) (a) *n*-BuLi (1.2 eq), dried THF, 0 °C–r.t., N_2_, (b) 4-iodobenzyl bromide (1.2 eq), dried THF, 0 °C–r.t., N_2_, 31%; (iii) (a) *n*-BuLi, dried THF, −78 °C, N_2_, (b) **2**, dried THF, −78 °C, N_2_, (c) MeSO_3_H, MeOH, −78 °C–r.t., N_2_; (iv) Ac_2_O, DMAP, pyridine, 0 °C–r.t., N_2_; (v) Et_3_SiH/BF_3_·Et_2_O, dried CH_2_Cl_2_, −35 °C–r.t., N_2_, 25% (over 3 steps from **29**); (vi) TBAF, dried THF, −78 °C–r.t., N_2_, 71%; (vii) MeONa, MeOH, 0 °C–r.t., N_2_, 89%.

The synthetic route to target compound **A1** is shown in Scheme 2. Commercially available *o*-carborane **27** was deprotonated with 1 eq of *n*-BuLi and then treated with *tert*-butyldimethylsilyl chloride (TBDMSCl) to give **28** with TBDMS at one of its two carbon atoms [32]. Deprotonation of **28** with 1 eq of *n*-BuLi followed by treatment with 4-iodobenzyl bromide gave **29** with the incoming 4-iodobenzyl group attached to the other carbon atom of *o*-carborane [33]. Treatment of **29** with *n*-BuLi followed by trapping of the generated aryl lithium with lactone **2** afforded a 1-*C*-aryl lactol intermediate, which was further converted to corresponding 1-*C*-aryl methyl glucopyranoside **30** upon treatment with MeSO_3_H/MeOH. Peracetylation of **30** with Ac_2_O in the presence of DMAP in pyridine afforded **31**, which was reduced to **32** with Et_3_SiH in the presence of BF_3_·Et_2_O, as discussed above. The TBDMS group in **32** was cleaved with tetra-*n*-butylammonium fluoride (TBAF) to give **33** [32]. Zemplén deacetylation of **33** through treatment with MeONa in MeOH to remove all of the acetyl groups yielded target compound **A1**.

**Scheme 3:**
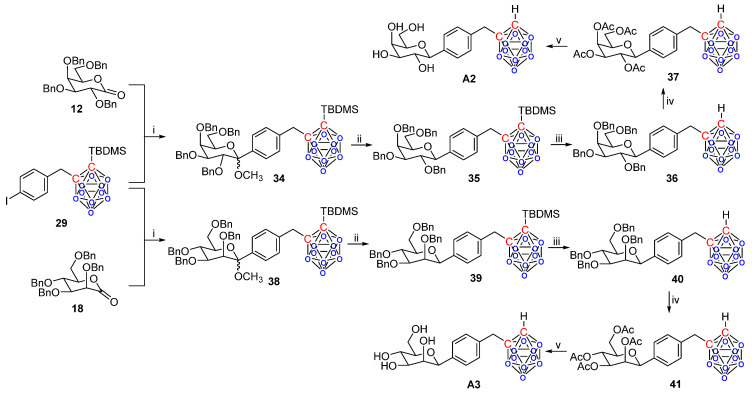
Synthetic route to target compounds **A2** and **A3**. Reagents and conditions: (i) (a) *n*-BuLi, dried THF, −78 °C, N_2_, (b) **12** (for **34**) or **18** (for **38**), dried THF, −78 °C, N_2_, (c) MeSO_3_H, MeOH, −78 °C–r.t., N_2_; (ii) Et_3_SiH/BF_3_·Et_2_O, dried CH_2_Cl_2_, −35 °C–r.t., N_2_, 41% for **35** (over 2 steps from **29**), 39% for **39** (over 2 steps from **29**); (iii) TBAF, dried THF, −78 °C–r.t., N_2_, 57% for **36**, 66% for **40**; (iv) BF_3_·Et_2_O/Ac_2_O, 0–50 °C, N_2_, 42% for **37**, 74% for **41**; (v) MeONa, MeOH, 0 °C–r.t., N_2_, 87% for **A2**, 92% for **A3**.

The synthetic routes to target compounds **A2** and **A3** are shown in Scheme 3. Following an identical procedure described above, aryl iodide **29** underwent iodine–lithium exchange upon treatment with *n*-BuLi, and the aryl lithium intermediate generated subsequently was trapped with **12** followed by treatment with MeSO_3_H/MeOH to give **34**, which was then reduced to **35** with Et_3_SiH/BF_3_·Et_2_O. TBAF-mediated cleavage of TBDMS in **35** produced **36**. Concomitant debenzylation and acetylation of **36** to give **37** was achieved by treatment with Ac_2_O in the presence of BF_3_·Et_2_O [34]. Zemplén deacetylation of **37** through treatment with MeONa in MeOH afforded target compound **A2**. Following the procedure for the synthesis of **A2**, the target compound **A3** with a mannose moiety was produced starting from **29** and a mannopyranose lactone **18**.

#### 2.1.3. Synthesis of Series B

**Scheme 4:**
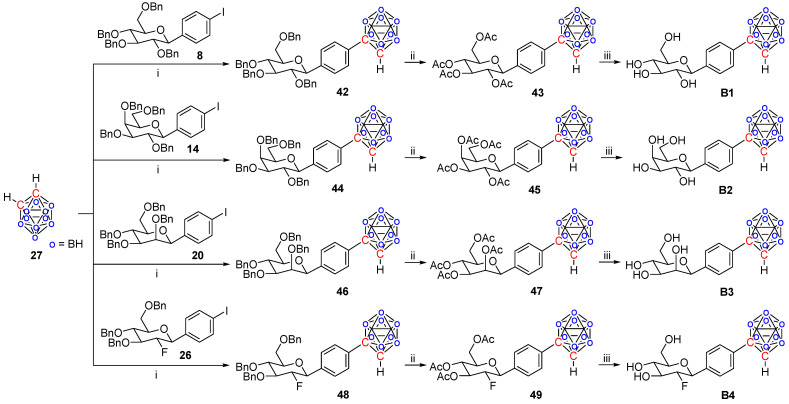
Synthetic route to target compounds **B1**–**B4**. Reagents and conditions: (i) (a) *n*-BuLi (1.5 eq), dried THF, 0 °C–r.t., N_2_, (b) **8** (for **42**), **14** (for **44**), **20** (for **46**) or **26** (for **48**), Pd[P(*t*-Bu)_3_]_2_, PCy_3_, dried *m*-xylene, 130 °C, N_2_, 21% for **42**, 49% for **44**, 24% for **46**, 23% for **48**; (ii) BF_3_·Et_2_O/Ac_2_O, 0–50 °C, N_2_, 64% for **43**, 52% for **45**, 73% for **47**, 64% for **49**; (iii) MeONa, MeOH, 0 °C–r.t., N_2_, 91% for **B1**, 90% for **B2**, 54% for **B3**, 50% for **B4**.

The synthetic routes to target compounds **B1**–**B4** are summarized in Scheme 4. Thus, *o*-carborane **27** was deprotonated with 1 eq of *n*-BuLi to generate a carbanion intermediate, which underwent cross coupling with aryl iodides **8**, **14**, **20,** and **26** mediated by Pd[P(*t*-Bu)_3_]_2_ and tricyclohexylphosphine (PCy_3_) to give **42**, **44**, **46,** and **48**, respectively [35]. Following the identical procedure discussed above for the conversion of **36** to **A2**, **42**, **44**, **46,** and **48** were converted to target compounds **B1**–**B4**, respectively.

#### 2.1.4. Synthesis of Series C

**Scheme 5:**
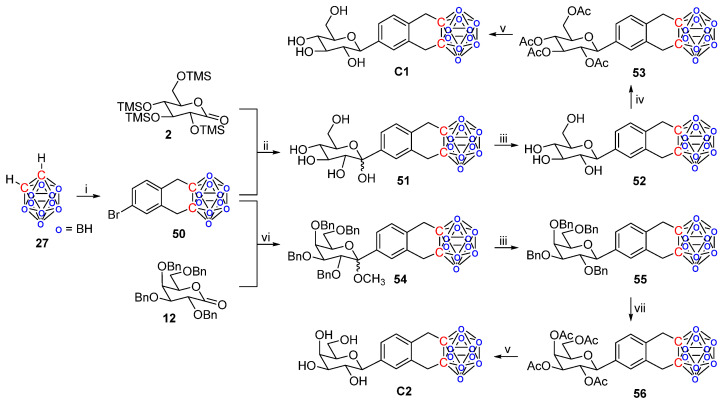
Synthetic route to target compounds **C1** and **C2**. Reagents and conditions: (i) (a) *n*-BuLi (2.5 eq), dried THF/dried toluene (1/2 by *v*/*v*), 0 °C–r.t., N_2_, (b) **4**, dried toluene, 0–100 °C, N_2_, 50%; (ii) (a) *n*-BuLi, dried THF, −78 °C, N_2_, (b) **2**, dried THF, −78 °C, N_2_, (c) strongly acidic resin (H^+^ form), MeOH, r.t., N_2_; (iii) Et_3_SiH/BF_3_·Et_2_O, dried CH_2_Cl_2_, −35 °C–r.t., N_2_; (iv) Ac_2_O, DMAP, pyridine, 0 °C–r.t., N_2_, 7% (over 3 steps from **50**); (v) MeONa, MeOH, 0 °C–r.t., N_2_, 52% for **C1**, 80% for **C2**; (vi) (a) *n*-BuLi, dried THF, −78 °C, N_2_, (b) **12**, dried THF, −78 °C, N_2_, (c) MeSO_3_H, MeOH, −78 °C–r.t., N_2_; (vii) BF_3_·Et_2_O/Ac_2_O, 0–50 °C, N_2_, 15% (over 3 steps from **50**).

The synthetic routes to target compounds **C1** and **C2** are depicted in Scheme 5. Thus, treatment of *o*-carborane (**27**) with 2 eq of *n*-BuLi led to deprotonation at its two carbon atoms, and the carbanion generated then reacted with bisbenzyl bromide **4** to form a cyclized product **50** [36]. Following a similar procedure discussed above, aryl bromide **50** underwent bromine–lithium exchange upon treatment with *n*-BuLi to generate an aryl lithium intermediate, which was trapped with pertrimethylsilyl-protected lactone **2** followed by removal of all of the TMS-protecting group with strongly acidic resin (H^+^ form) to give 1-*C*-aryl lactol **51**. The latter was reduced to **52** with Et_3_SiH/BF_3_·Et_2_O. Compound **52** was actually the target compound **C1**, but it was rather impure and hard to purify through conventional silica gel column chromatography; instead of directly purifying **52** through preparative HPLC, it was first peracetylated with Ac_2_O in the presence of DMAP in pyridine to afford **53**, which was purified through conventional column chromatography and then subjected to Zemplén deacetylation through treatment with MeONa/MeOH to give pure target compound **C1**. Following a similar procedure as the synthesis of **A2** from **29** and **12**, the target compound **C2** was prepared from **50** and **12**.

#### 2.1.5. Synthesis of Series D

**Scheme 6:**
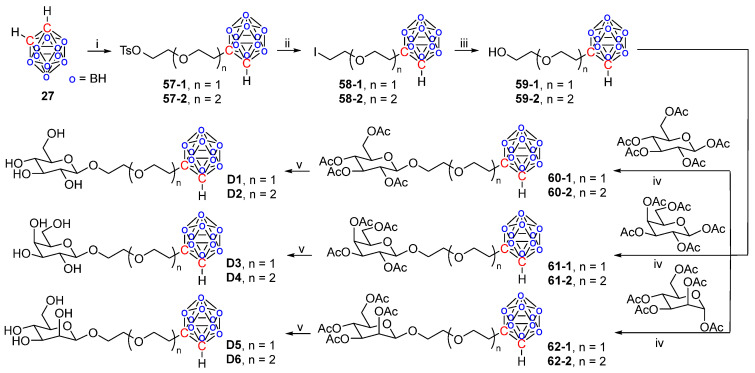
Synthetic route to target compounds **D1**–**D6**. Reagents and conditions: (i) (a) *n*-BuLi (1.2 eq), dried THF, 0 °C–r.t., N_2_, (b) TsO-(CH_2_CH_2_O)_2_-Ts or TsO-(CH_2_CH_2_O)_3_-Ts (1.2 eq), 0 °C–r.t., N_2_, 15% for **57-1**, 22% for **57-2**; (ii) NaI, TBAI, dried toluene, 100 °C, N_2_, 62% for **58-1**, 89% for **58-2**; (iii) (a) AcONa, dried DMF, 80 °C, N_2_, (b) NaOH, MeOH, r.t., N_2_, 56% for **59-1**, 50% for **59-2**; (iv) β-D-glucose pentaacetate (for **60-1** and **60-2**), β-D-galactose pentaacetate (for **61-1** and **61-2**), or α-D-mannose pentaacetate (for **62-1** and **62-2**), BF_3_·Et_2_O, dried CH_2_Cl_2_, 0 °C–r.t., N_2_, 41% for **60-1**, 31% for **60-2**, 33% for **61-1**, 28% for **61-2**, 39% for **62-1**, 55% for **62-2**; (v) MeONa, MeOH, 0 °C–r.t., N_2_, 88% for **D1**, 81% for **D2**, 53% for **D3**, 69% for **D4**, 77% for **D5**, 75% for **D6**.

The synthetic routes to the target compounds **D1**–**D6** are shown in Scheme 6. Thus, deprotonation of *o*-carborane (**27**) with 1 eq of *n*-BuLi generated a carbanion at one of its two carbon atoms, which then underwent SN2 nucleophilic substitution with diethylene glycol di(*p*-toluenesulfonate) and triethylene glycol bis(*p*-toluenesulfonate) to produce **57-1** and **57-2**, respectively. Treatment of tosylates **57-1** and **57-2** with NaI in the presence of TBAI in toluene at 100 °C gave corresponding iodides **58-1** and **58-2**, respectively; the latter were first converted to their corresponding acetates through SN2 nucleophilic substitution with AcONa in DMF at 80 °C and then hydrolyzed with NaOH in MeOH to alcohols **59-1** and **59-2**, respectively. Primary alcohol **59** (including **59-1** and **59-2**) was reacted with β-D-glucose pentaacetate, β-D-galactose pentaacetate, and α-D-mannose pentaacetate in the presence of BF_3_·Et_2_O to afford *O*-glucosides **60-1** and **60-2**, *O*-galactosides **61-1** and **61-2**, and *O*-mannosides **62-1** and **62-2**, respectively [37]. Zemplén deacetylation of these compounds with MeONa in MeOH as described above gave target compounds **D1**–**D6**.

#### 2.1.6. Synthesis of Series E

**Scheme 7:**
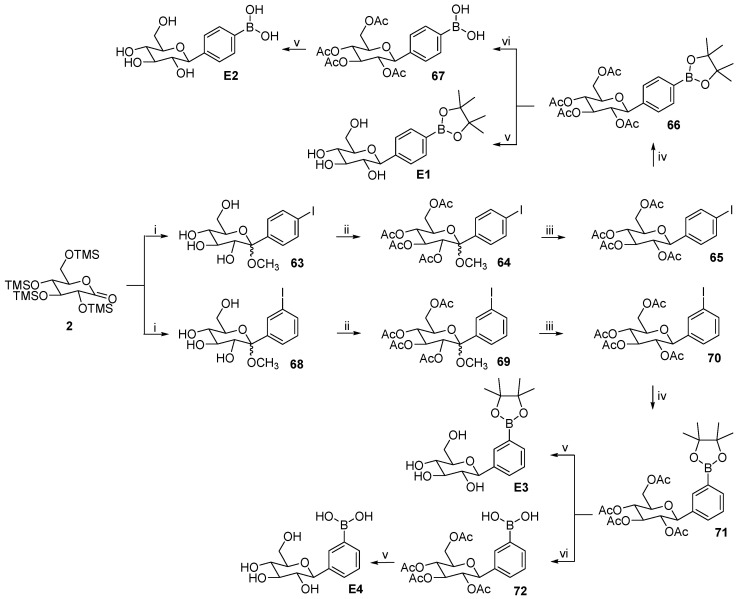
Synthetic route to target compounds **E1**–**E4**. Reagents and conditions: (i) (a) 1,4-diiodobenzene (1.2 eq) or 1,3-diiodobenzene (1.2 eq), *n*-BuLi (1.2 eq), dried THF, −78 °C, N_2_, (b) **2** (1 eq), dried THF, −78 °C, N_2_, (c) MeSO_3_H, MeOH, −78 °C–r.t., N_2_; (ii) DMAP, pyridine, Ac_2_O, 0 °C–r.t., N_2_, 44% for **64** (over 2 steps from **2**); (iii) Et_3_SiH/BF_3_·Et_2_O, dried CH_2_Cl_2_, −35 °C–r.t., N_2_, 84% for **65**, 14% for **70** (over 3 steps from **2**); (iv) B_2_Pin_2_, KOAc, Pd(dppf)Cl_2_, dried DMSO, 87 °C, N_2_, 72% for **66**, 38% for **71**; (v) MeONa, MeOH, 0 °C–r.t., N_2_, 32% for **E1**, 74% for **E2**, 49% for **E3**, 66% for **E4**; (vi) NaIO_4_, NH_4_OAc, acetone/H_2_O (1/1 by *v*/*v*), r.t., 76% for **67**, 60% for **72**.

The synthetic routes to the target compounds **E1**–**E4** are shown in Scheme 7. Following the procedure for the synthesis of **32** from **29** and **2**, **65** was prepared from 1,4-diiodobenzene and **2**. Cross coupling of aryl iodide **65** and bis(pinacolato)diboron (B_2_Pin_2_) in the presence of Pd(dppf)Cl_2_ and KOAc in DMSO at 87 °C gave **66**. Zemplén deacetylation of **66** with MeONa in MeOH gave target compound **E1**; oxidative cleavage of the pinacol ester functionality in **66** with NaIO_4_ in the presence of NH_4_OAc in acetone/H_2_O afforded phenylboronic acid **67**, and Zemplén deacetylation of the latter with MeONa in MeOH gave the target compound **E2**. Following the same procedures for the synthesis of **E1** and **E2** described above, **E3** and **E4** were prepared from 1,3-diiodobenzene and **2**.

**Scheme 8:**
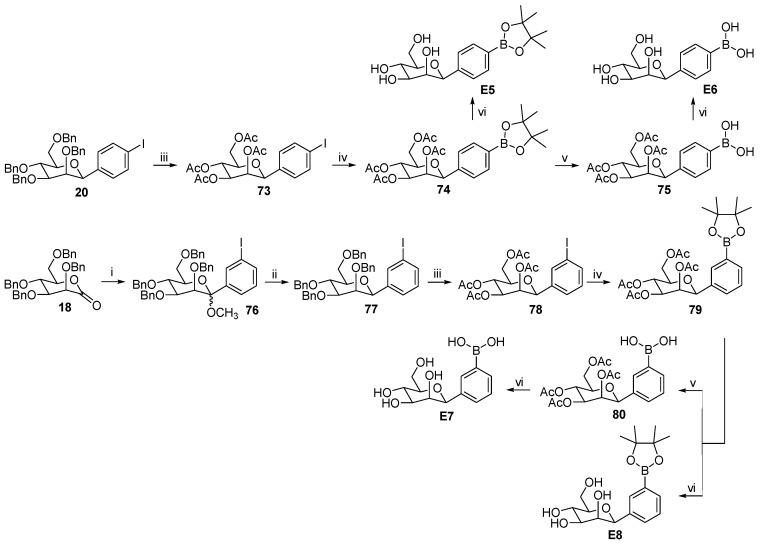
Synthetic route to target compounds **E5**–**E8**. Reagents and conditions: (i) (a) 1,3-diiodobenzene (1.5 eq), *n*-BuLi (1.5 eq), dried THF, −78 °C, N_2_, (b) **18** (1 eq), dried THF, −78 °C, N_2_, (c) MeSO_3_H, MeOH, −78 °C–r.t., N_2_; (ii) Et_3_SiH/BF_3_·Et_2_O, dried CH_2_Cl_2_, −35 °C–r.t., N_2_, 22% (over 2 steps from **18**); (iii) BF_3_·Et_2_O/Ac_2_O, 0–70 °C, N_2_, 80% for **73**, 67% for **78**; (iv) B_2_Pin_2_, KOAc, Pd(dppf)Cl_2_, dried DMSO, 87 °C, N_2_, 87% for **74**, 33% for **79**; (v) NaIO_4_, NH_4_OAc, acetone/H_2_O (1/1 by *v*/*v*), r.t., 59% for **75**, 68% for **80**; (vi) MeONa, MeOH, 0 °C–r.t., N_2_, 49% for **E5**, 39% for **E6**, 36% for **E7**, 53% for **E8**.

The synthetic routes to the target compounds **E5**–**E8** are shown in Scheme 8. Following the procedure for the synthesis of **8** from **6** and 1,4-diiodobenzene, **77** was prepared from **18** and 1,3-diiodobenzene. Perbenzylated mannosides **20** and **77** were treated with Ac_2_O/BF_3_·Et_2_O as described above to give peracetylated counterparts **73** and **78**, respectively. Following the procedure from **65** for the synthesis of **E1** and **E2**, **73** and **78** were converted to **E5**/**E6** and **E7**/**E8**, respectively.

**Scheme 9:**
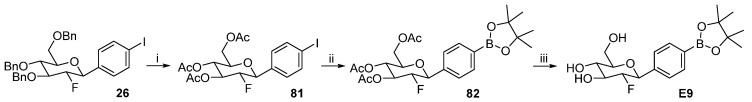
Synthetic route to target compounds **E9**. Reagents and conditions: (i) BF_3_·Et_2_O/Ac_2_O, 0 °C–r.t., N_2_, 80%; (ii) B_2_Pin_2_, KOAc, Pd(dppf)Cl_2_, dried DMSO, 87 °C, N_2_, 34%; (iii) MeONa, MeOH, 0 °C–r.t., N_2_, 15%.

The synthetic route to the target compound **E9** is shown in Scheme 9. **E9** was prepared from **26** following the procedure for the synthesis of **E5** from **20**.

#### 2.1.7. Synthesis of Series F

**Scheme 10:**
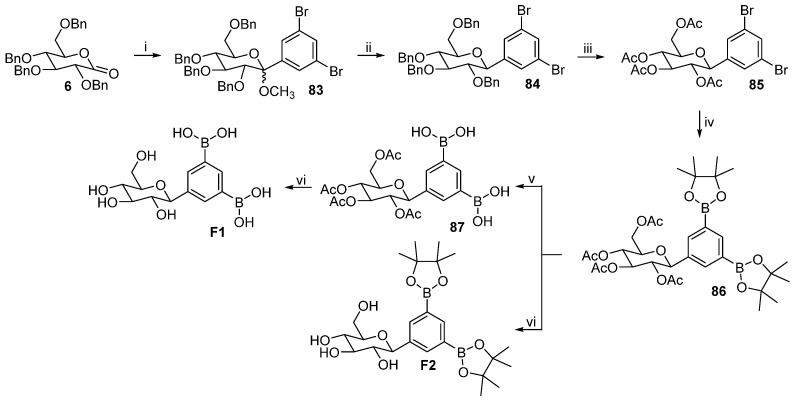
Synthetic route to target compounds **F1** and **F2**. Reagents and conditions: (i) (a) 1,3,5-tribromobenzene (2.5 eq), *n*-BuLi (2 eq), dried THF, −78 °C, N_2_, (b) **6** (1 eq), dried THF, −78 °C, N_2_, (c) MeSO_3_H, MeOH, −78 °C–r.t., N_2_; (ii) Et_3_SiH/BF_3_·Et_2_O, dried CH_2_Cl_2_, −35 °C–r.t., N_2_; (iii) BF_3_·Et_2_O/Ac_2_O, 0 °C–r.t., N_2_, 11% (over 3 steps from **6**); (iv) B_2_Pin_2_, KOAc, Pd(dppf)Cl_2_, dried DMSO, 87 °C, N_2_, 41%; (v) NaIO_4_, NH_4_OAc, acetone/H_2_O (1/1 by *v*/*v*), r.t.; (vi) MeONa, MeOH, 0 °C–r.t., N_2_, 17% for **F1** (over 2 steps from **86**), 49% for **F2**.

The synthetic routes to the target compounds **F1** and **F2** are shown in Scheme 10. Following the procedure for the synthesis of **E7** and **E8** from **18**, **F1** and **F2** were prepared from **6** and 1,3,5-tribromobenzene.

**Scheme 11:**
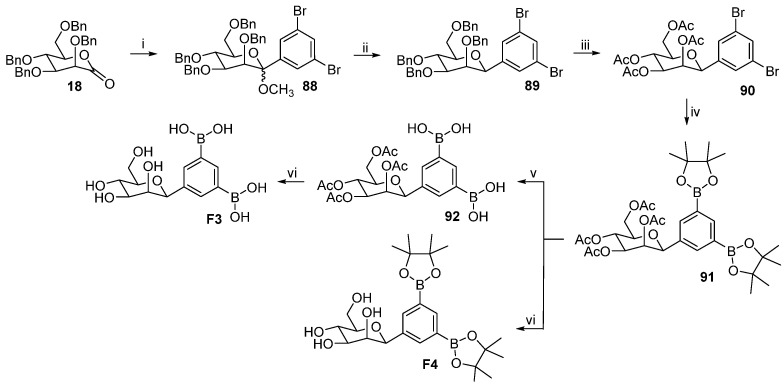
Synthetic route to target compounds **F3** and **F4**. Reagents and conditions: (i) (a) 1,3,5-tribromobenzene (2.5 eq), *n*-BuLi (2 eq), dried THF, −78 °C, N_2_, (b) **18** (1 eq), dried THF, −78 °C, N_2_, (c) MeSO_3_H, MeOH, −78 °C–r.t., N_2_; (ii) Et_3_SiH/BF_3_·Et_2_O, dried CH_2_Cl_2_, −35 °C–r.t., N_2_; (iii) BF_3_·Et_2_O/Ac_2_O, 0–70 °C, N_2_, 11% (over 3 steps from **18**); (iv) B_2_Pin_2_, KOAc, Pd(dppf)Cl_2_, dried DMSO, 87 °C, N_2_, 39%; (v) NaIO_4_, NH_4_OAc, acetone/H_2_O (1/1 by *v*/*v*), r.t.; (vi) MeONa, MeOH, 0 °C–r.t., N_2_, 11% for **F3** (over 2 steps from **91)**, 41% for **F4**.

The synthetic routes to the target compounds **F3** and **F4** are shown in Scheme 11. Following the procedure for the synthesis of **F1** and **F2** from **6** and 1,3,5-tribromobenzene, the mannose-bearing target compounds **F3** and **F4** were prepared from mannose lactone **18** and 1,3,5-tribromobenzene.

It should be noted that the compounds in series E and F were found to be slightly unstable after prolonged storage at 0 °C to room temperature.

The anomeric configurations of the sugar ring in all of the target compounds were found to be β, as evidenced by the coupling constants of H1-H2 in their peracetylated derivatives. Specifically, for all of the target compounds with a glucopyranose or galactopyranose moiety, the H1-H2 coupling constants were in the range of 8.0–10.0 Hz, while for those with a mannopyranose moiety the H1-H2 coupling constants were in the range of 0–2.0 Hz. For **B4** and **E9**, both with a 2-deoxy-2-fluoro-D-glucopyranose moiety, the anomeric configuration was hard to determine directly due to the complication of signal splitting given the presence of an F atom and signal overlapping in the ^1^H NMR spectra; however, a similar synthetic procedure reported earlier led to the formation of the same 2-deoxy-2-fluoro-D-glucopyranose with an anomeric β configuration [38], supporting the anomeric β configuration in **B4** and **E9**.

### 2.2. Cytotoxicity Assay

As discussed above, low systemic toxicity is one of the criteria for “ideal” boron carriers. Thus, a 24 h cytotoxicity assay was conducted to determine the anti-proliferative activity of the target compounds against two selected cell lines, SCC-9 human tongue squamous cell carcinoma cells and HaCaT immortalized human keratinocytes, and the results were expressed as the half-maximal inhibitory concentration (IC_50_) and are summarized in Table 1. As will be discussed in the next section, SCC-9 and HaCaT cell lines were selected to represent GLUT1 high expressing tumor and normal cell lines, respectively, for the determination of selective cellular boron uptake (T/N). As shown in Table 1, the IC_50_ values of the target compounds in series A–D against SCC-9 and HaCaT cell lines were in the range of 0.20–0.71 mM, indicating that their cytotoxicity was relatively small and acceptable compared with the boron-bearing compounds reported earlier [17,18,20,23]; the IC_50_ values of the target compounds in series E–F were >1 mM, indicating that those compounds in series E–F exhibited smaller cytotoxicity than those in series A–D. BPA was used as a reference compound and was found to exhibit no significant cytotoxicity toward either cell line within the tested concentration range, comparable to the target compounds in series E–F (IC_50_ values > 1 mM).

### 2.3. Cellular Boron Uptake Assay

As discussed above, among the criteria for an “ideal” boron carrier, high tumor specificity (T/N and T/B ratios) and high boron tumor uptake are of crucial importance for a boron carrier compound to achieve highly selective uptake by tumor cells and then accumulation in tumor tissues compared with normal adjacent tissues. In order to determine these two factors for the target compounds in the present study, we selected the SCC-9 cell line, a kind of human tongue squamous cell carcinoma cell, and the HaCaT cell line, a kind of human keratinocyte, to represent tumor cells and normal cells, respectively. It should be noted that the GLUT1 high expressing SCC-9 cell line was selected to represent tumor cells because the target compounds in the present study were designed to target GLUT1 [39]. Thus, the SCC-9 and HaCaT cells were incubated with a solution of target compounds at specific concentrations for 3 h, and the boron taken up by the cells was quantified through inductively coupled plasma–mass spectrometry (ICP-MS) and expressed as μg/10^6^ cell; the T/N was calculated as [boron taken up by SCC-9 cell line (μg/10^6^ cell)]/[boron taken up by HaCaT cell (μg/10^6^ cell)].

The designed six series of target compounds in the present study fall into two categories: category I includes series A–D, with each target compound having one *o*-carborane moiety, while category II includes series E–F, with each target compound having one or two boronic acid or its pinacol ester moieties (Figure 3). The boron uptake results for target compounds series A–D (category I) and series E–F (category II) are summarized in Table 2 and Table 3, respectively, and their calculated T/N ratios are summarized in Table 4 and Table 5, respectively. For the target compounds in series A–D (category I), their boron uptakes were determined at two concentrations (0.1 mM and 0.05 mM), both lower than their cytotoxicity IC_50_ values. Three compounds in series A, **A1**–**A3**, were initially designed by connecting three individual phenyl *C*-glycoside moieties to *o*-carborane by a CH_2_ linker. All three compounds could be efficiently taken up by both cell lines, with boron uptake at 0.88–1.42 μg/10^6^ cell by the SCC-9 cell line and 0.53–0.60 μg/10^6^ cell by the HaCaT cell line at 0.1 mM (T/N = 1.6–2.4), which were significantly higher than those for BPA, with boron uptake at 0.21 μg/10^6^ cell by the SCC-9 cell line and 0.15 μg/10^6^ cell by the HaCaT cell line at 5 mM (T/N = 1.4). Considering **A3**, a phenyl *C*-mannoside, exhibited the highest boron uptake and the largest T/N value (2.4) in series A, we moved on with the phenyl *C*-mannoside moiety to further design series B by removing the CH_2_ linker in series A. Overall, the compounds in series B exhibited higher boron uptake and larger T/N value than those in series A, with **B3**, also a phenyl *C*-mannoside, being the best (T/N = 3.3 at 0.1 mM). Series C was then designed by adding one more CH_2_ linker to series A to form a fused tricycle system, but it was associated with decreased boron uptake and a comparable T/N value. Although the exact reason remains unknown, it was observed that cellular boron uptake across series A–C depended on the rigidity of aglycons, and reasonable rigidity seemed beneficial to cellular boron uptake (rigidity increased in this order: series A < series B < series C). Series D was designed by connecting three individual *O*-glycosides to *o*-carborane with two long hydrophilic poly ethylene glycol (PEG) linkers with an aim to improve aqueous solubility; unfortunately, although the T/N values were comparable to those in series A–C, series D was associated with dramatically decreased boron uptake. Finally, we designed series E by incorporating boronic acid or its pinacol ester moiety with the benzene ring of two phenyl *C*-glycosides and series F by incorporating two boronic acid or its pinacol ester moieties on the base of series E. The boron uptakes for both series were determined at 1 mM and 2.5 mM for these two series; disappointingly, almost no boron uptake was observed in both series by SCC-9 and HaCaT (no more than 0.1 μg/10^6^ cell) and, in this case, the calculated T/N values were of little value, indicating that the structures of target compounds in series E–F are not favorable for BNCT. As discussed above, category I was significantly better than category II in terms of cellular boron uptake. The exact reason for this significant difference is unknown but can be partially explained by the following two factors. (1) The *o*-carborane moiety in category I has a 10-fold boron-carrying capacity compared to the boronic acid and its pinacol ester moieties in category II. (2) The compounds in category I could be transported more efficiently than those in category II, probably due to the difference between their structures and physicochemical properties, such as lipophilicity and water solubility. In summary, compounds in series A–C (category I) were associated with higher cellular boron uptake by SCC-9 and HaCaT cell lines and larger T/N values. Notably, compound **B3** was the best one and demonstrated 10.6-fold higher boron uptake by the SCC-9 cell line and a superior T/N (3.3 for **B3** vs. 1.4 for BPA) compared with BPA. The chemical structure of **B3** represents a privileged candidate structure for the future design of “ideal” boron carriers for BNCT. Further SAR exploration around the structure of **B3** is promising to discover an “ideal” boron carrier for BNCT and will be conducted in the future because the T/N of **B3** still falls short of that for an “ideal” boron carrier (T/N = 3.3 for **B3** vs. T/N > 5 for “ideal” boron carriers).

## 3. Materials and Methods

### 3.1. Chemistry

Unless stated otherwise, all of the chemical reagents were obtained commercially and used without further purification. All of the dried solvents were prepared through standard methods. Reaction progress was monitored through thin-layer chromatography (TLC) on commercially available precoated TLC silica gel plates, and separation and purification on flash column chromatography were performed through silica gel column (200–300 or 300–400 mesh) eluting with EtOAc/petroleum ether (PE, b.p. 60–90 °C) or MeOH/CH_2_Cl_2_ (by *v*/*v*). Melting points were measured in open capillaries with an SGW X-4A microscopic melting point apparatus (Shanghai INESA Physico-Optical Instrument Co., Ltd., Shanghai, China) and are uncorrected. Optical rotations were determined on an Anton Paar MCP 4100 polarimeter (Anton Paar OptoTec, Seelze, Germany) at 20 °C in MeOH, DMSO, or H_2_O as solvent. NMR spectra were recorded on a Bruker Ascend 500 or 600 NMR spectrometer (Bruker Switzerland AG, Fällanden, Switzerland) using CDCl_3_, DMSO-*d*_6_, CD_3_OD, or D_2_O as the solvent and TMS (for ^1^H NMR) or known chemical shifts of carbon signals of deuterated solvents (for ^13^C NMR) as the internal standard. High-resolution mass spectra (HR-MS) were determined with a Thermo Scientific Exactive Plus mass spectrometer (Thermo Fisher Scientific, Bremen, Germany) equipped with electrospray ionization (ESI) and an Orbitrap mass analyzer. The purities were determined with a Waters Arc HPLC instrument equipped with a UV detector (Waters Corporation, Singapore); Column, chromcore C_18_ (Nanochrom), 4.6 × 150 mm, 3 μm; eluent, H_2_O/MeOH = 20/80; flow rate, 0.7 min/min; column temperature, room temperature; wavelength, 201 nm for **C1** and **C2**, 220 nm for the others.

The copies of ^1^H NMR, ^13^C NMR, ^19^F NMR, ^11^B NMR and HR-MS spectra of the synthesized intermediates and target compounds can be seen in Supplementary Materials.

General synthetic procedure 1: To a stirred solution of sugar lactol (1.0 eq) in DMSO (about 10 mL/g of sugar derivative) cooled at 0 °C under N_2_ was added dropwise Ac_2_O (5 mL/g of sugar derivative) and, after addition, the reaction mixture was stirred at room temperature overnight when TLC analysis indicated completion of the reaction. The reaction mixture was poured into stirred ice water, and the resulting mixture was extracted with EtOAc in three portions (3×). The combined extracts were washed successively with saturated aqueous NaHCO_3_ and brine, dried (MgSO_4_), and evaporated on a rotary evaporator to afford a residue, which was purified through column chromatography to obtain the product sugar lactone.

General synthetic procedure 2: To a stirred solution of aryl halide (1.0–1.2 eq) in dried THF (about 10 mL/g of aryl halide) cooled at −78 °C under N_2_ was added dropwise a solution of *n*-BuLi in THF (1.0–1.2 eq) and, after addition, the resulting mixture was stirred at this temperature for 1 h followed by dropwise addition of a solution of sugar lactone in dried THF. After addition, the resulting mixture was stirred at this temperature for 1 h followed by dropwise addition of a solution of MeSO_3_H (5.0 eq) in MeOH. After that, the reaction mixture was stirred at room temperature overnight when TLC analysis indicated completion of the reaction and poured into ice water, and the mixture thus obtained was extracted with EtOAc in three portions (3×). The combined extracts were washed successively with saturated aqueous NaHCO_3_ and brine, dried (MgSO_4_), and evaporated on a rotary evaporator to afford a crude methyl glycopyranoside, which was used in the next step without further purification or characterization.

General synthetic procedure 3: To a stirred solution of the crude methyl glycopyranoside prepared above in general synthetic procedure 2 (1.0 eq, calculated by assuming the sample was pure) in dried CH_2_Cl_2_ (about 10 mL/g of crude methyl glycopyranoside) cooled at −35 °C under N_2_ were added successively Et_3_SiH (2.0–3.0 eq) and BF_3_·Et_2_O (1.2–5.2 eq) each in a dropwise manner, and, after additions, the reaction mixture was stirred at room temperature overnight when TLC analysis indicated completion of the reaction. The reaction mixture was poured into ice water, and the mixture thus obtained was extracted with CH_2_Cl_2_ in three portions (3×). The combined extracts were washed successively with saturated aqueous NaHCO_3_ and brine, dried (MgSO_4_), and evaporated on a rotary evaporator to afford a residue, which was purified through column chromatography to obtain the desired product.

General synthetic procedure 4: To a stirred solution of *o*-carborane (1.0 eq) in dried THF (about 8 mL/g of *o*-carborane) cooled at 0 °C under N_2_ was added dropwise a solution of *n*-BuLi in THF (1.5 eq) and, after addition, the resulting mixture was stirred at room temperature for 1 h followed by the addition of bis(tri-*tert*-butylphosphine)palladium(0) (Pd[P(*t*-Bu)_3_]_2_) (0.1–1.0 eq) and tricyclohexylphosphine (PCy_3_) (0.1–1.0 eq). After that, stirring was continued for 0.5 h, and a solution of aryl iodide in dried *m*-xylene (20 mL/g of aryl iodide) was added. The reaction mixture was then stirred at 130 °C overnight when TLC analysis indicated completion of the reaction. Upon cooling to room temperature, the reaction mixture was quenched with saturated aqueous NH_4_Cl, and the mixture thus obtained was extracted with EtOAc in three portions (3×). The combined extracts were washed with brine, dried (MgSO_4_), and evaporated on a rotary evaporator to afford a residue, which was purified through column chromatography to obtain the desired product.

General synthetic procedure 5: To a stirred dried MeOH (about 20 mL/g of substrate) cooled at 0 °C under N_2_ was added portion-wise freshly cut sodium (3.0–12.0 eq), and the mixture was stirred until the sodium was consumed completely. The substrate to be deacetylated was added, and stirring was continued at room temperature until completion, as indicated by TLC analysis (typically within 0.5 h). Strongly acidic cation exchange resin (Amberlite 732) was added to neutralize the OH^−^ and absorb the Na^+^ until pH = 7. The mixture was filtered off, and the filtrate was evaporated on a rotary evaporator to afford a residue, which was purified through column chromatography to obtain the desired product.

General synthetic procedure 6: To a stirred solution of alcohol (1.0 eq) and β-D-glucose pentaacetate (2.0 eq) in dried CH_2_Cl_2_ cooled at 0 °C under N_2_ was added dropwise BF_3_·Et_2_O (4.0 eq) and, after addition, the reaction mixture was stirred at room temperature when TLC analysis indicated completion of the reaction. The reaction mixture was poured into ice water, and the resulting mixture was extracted with CH_2_Cl_2_ in three portions (3×). The combined extracts were washed successively with saturated aqueous NaHCO_3_ and brine, dried (MgSO_4_), and evaporated on a rotary evaporator to afford a residue, which was purified through column chromatography to obtain the desired product.

General synthetic procedure 7: A mixture of aryl halide (1.0 eq), B_2_Pin_2_ (1.0–3.0 eq), KOAc (3.0 eq), and Pd(dppf)Cl_2_ (0.2–0.3 eq) in dried DMSO under N_2_ was stirred at 87 °C until completion of the reaction, as indicated by TLC analysis (typically within 5–6 h). Upon cooling to room temperature, the reaction mixture was poured into ice water, and the resulting mixture was filtered off through celite. The filtrate was extracted with EtOAc in three portions (3×), and the combined extracts were washed with brine, dried (MgSO_4_), and evaporated on a rotary evaporator to afford a residue, which was purified through column chromatography to obtain the desired product.

General synthetic procedure 8: To a stirred solution of phenylboronic acid pinacol ester (1.0 eq) in a mixed solvent of acetone/H_2_O (8/2 by *v*/*v*; in total 10 mL/g of phenylboronic acid pinacol ester) at room temperature were added NaIO_4_ (2.5 eq) and NH_4_OAc (1.5 eq) and, after addition, the reaction mixture was stirred at room temperature overnight when TLC indicated completion of the reaction. The reaction mixture was then brought to pH = 3 with 1 M HCl and extracted with CH_2_Cl_2_ in three portions (3×), and the combined extracts were washed with brine, dried (MgSO_4_), and evaporated on a rotary evaporator to afford a residue, which was purified through column chromatography to obtain the desired product.

Synthesis of (3*R*,4*S*,5*R*,6*R*)-3,4,5-tris((trimethylsilyl)oxy)-6-(((trimethylsilyl)oxy)methyl)tetrahydro-2H-pyran-2-one (**2**): To a stirred mixture of D-glucono-δ-lactone (**1**, 30.00 g, 0.168 mol) in dried THF (300 mL) cooled at 0 °C under N_2_ were added NMM (136.27 g, 1.35 mol) and DMAP (2.06 g, 16.8 mmol), followed by dropwise addition of TMSCl (109.77 g, 1.01 mol), and, after addition, the reaction mixture was stirred at room temperature overnight. The reaction mixture was poured into ice water (300 mL), and the resulting mixture was extracted with *n*-hexane (b.p., 60–90 °C; 200 mL × 3). The combined extracts were washed with brine, dried (MgSO_4_), and evaporated on a rotary evaporator to afford a residue, which was purified through column chromatography (PE) to give **2**. Colorless oil, 53.15 g (68%). ^1^H NMR (500 MHz, CDCl_3_) *δ* 4.19–4.16 (m, 1H), 4.00 (d, *J* = 8.0 Hz, 1H), 3.90 (t, *J* = 7.5 Hz, 1H), 3.83–3.74 (m, 3H), 0.20–0.13 (m, 36H). The ^1^H NMR data were consistent with those reported [40].

Synthesis of 4-bromo-1,2-bis(bromomethyl)benzene (**4**): To a stirred mixture of 4-bromo-1,2-dimethylbenzene (**3**, 10.00 g, 54.0 mmol) and NBS (19.23 g, 0.108 mol) in dried CCl_4_ (100 mL) heated at 60 °C under N_2_ was added AIBN (0.89 g, 5.40 mmol) portion-wise, and, after addition, the reaction mixture was refluxed (*caution: violent exotherm may occur*) until completion of the reaction, as indicated by TLC analysis (typically within 1 h). Upon cooling to room temperature, the reaction mixture was filtered off through celite, and the filtrate was evaporated on a rotary evaporator to afford a residue, which was purified through column chromatography (PE) to give **4**. Colorless oil, 5.90 g (37%). ^1^H NMR (500 MHz, CDCl_3_) *δ* 7.52 (d, *J* = 2.0 Hz, 1H), 7.43 (dd, *J* = 8.0, 2.0 Hz, 1H), 7.25–7.23 (m, 1H), 4.59 (s, 2H), 4.58 (s, 2H). The ^1^H NMR data were consistent with those reported [41].

Synthesis of (3*R*,4*S*,5*R*,6*R*)-3,4,5-tris(benzyloxy)-6-((benzyloxy)methyl)tetrahydro-2H-pyran-2-one (**6**): Following the general synthetic procedure 1, **6** was prepared from **5** (25.00 g, 46.2 mmol) using Ac_2_O (125 mL) and DMSO (250 mL). The crude **5** was purified through column chromatography (EtOAc/PE = 1/5 by *v*/*v*) to give **6**. Yellow oil, 23.59 g (95%). ^1^H NMR (500 MHz, CDCl_3_) *δ* 7.39–7.37 (m, 2H), 7.35–7.28 (m, 14H), 7.26–7.24 (m, 2H), 7.18–7.16 (m, 2H), 4.99 (d, *J* = 11.5 Hz, 1H), 4.74–4.69 (m, 2H), 4.65–4.49 (m, 5H), 4.46–4.44 (m, 1H), 4.12 (d, *J* = 6.5 Hz, 1H), 3.97–3.89 (m, 2H), 3.74–3.65 (m, 2H). The ^1^H NMR data were consistent with those reported [42].

Synthesis of (3*R*,4*S*,5*R*,6*R*)-3,4,5-tris(benzyloxy)-6-((benzyloxy)methyl)-2-(4-iodophenyl)-2-methoxytetrahydro-2H-pyran (**7**): Following the general synthetic procedure 2, **7** was prepared from **6** (24.00 g, 44.6 mmol) and 1,4-diiodobenzene (17.64 g, 53.5 mmol) using *n*-BuLi (33 mL, 1.6 M in THF, 52.8 mmol), MeSO_3_H (21.40 g, 0.223 mol), MeOH (107 mL), and dried THF (240 mL). The crude compound **7** was isolated as a brown oil, 41.50 g. This sample was used directly in the next step without further purification or characterization.

Synthesis of (2*R*,3*R*,4*R*,5*S*,6*S*)-3,4,5-tris(benzyloxy)-2-((benzyloxy)methyl)-6-(4-iodophenyl)tetrahydro-2H-pyran (**8**): Following the general synthetic procedure 3, **8** was prepared from crude **7** prepared above (41.50 g, deemed to be 54.8 mmol) using Et_3_SiH (12.75 g, 0.110 mol) and BF_3_·Et_2_O (9.34 g, 65.8 mmol) in dried CH_2_Cl_2_ (415 mL). The crude **8** was purified through column chromatography (EtOAc/PE = 1/5 by *v*/*v*) to give **8**. White solid, 8.90 g (27% overall for **6** to **8**). m.p. 102.4–104.9 °C. ^1^H NMR (500 MHz, CDCl_3_) *δ* 7.68–7.66 (m, 2H), 7.34–7.27 (m, 13H), 7.22–7.16 (m, 7H), 6.93–6.91 (m, 2H), 4.95–4.85 (m, 3H), 4.64–4.61 (m, 2H), 4.56–4.54 (m, 1H), 4.44 (d, *J* = 10.5 Hz, 1H), 4.18 (d, *J* = 9.5 Hz, 1H), 3.88 (d, *J* = 10.0 Hz, 1H), 3.81–3.73 (m, 4H), 3.59–3.56 (m, 1H), 3.43 (t, *J* = 9.0 Hz, 1H). The ^1^H NMR data were consistent with those reported [43].

Synthesis of (2*R*,3*S*,4*S*,5*R*,6*S*)-3,4,5-tris(benzyloxy)-2-((benzyloxy)methyl)-6-methoxytetrahydro-2H-pyran (**10**): To a stirred mixture of methyl α-D-galactopyranoside (**9**, 50.00 g, 0.257 mol) and BnBr (264.00 g, 1.54 mol) in a mixture of dried DMF (400 mL) and dried THF (100 mL) cooled at 0 °C under N_2_ was added portion-wise NaH (61.80 g, 60%, 1.54 mol), and, after addition, the reaction mixture was stirred at room temperature until completion of the reaction, as indicated by TLC analysis (typically within 2 h), and then *slowly* poured into stirred ice water (1000 mL). The resulting mixture was extracted with EtOAc (250 mL × 3), and the combined extracts were washed with brine, dried (MgSO_4_), and evaporated on a rotary evaporator to afford a residue, which was purified through column chromatography (EtOAc/PE = 1/5 by *v*/*v*) to give **10**. Yellow oil, 130.64 g (91%). ^1^H NMR (500 MHz, CDCl_3_) *δ* 7.40–7.27 (m, 20H), 4.95 (d, *J* = 11.5 Hz, 1H), 4.86–4.82 (m, 2H), 4.75–4.68 (m, 3H), 4.57 (d, *J* = 11.5 Hz, 1H), 4.50–4.47 (m, 1H), 4.41–4.39 (m, 1H), 4.05–4.02 (m, 1H), 3.95–3.89 (m, 3H), 3.53 (d, *J* = 6.5 Hz, 2H), 3.37 (s, 3H). The ^1^H NMR data were consistent with those reported [44].

Synthesis of (3*R*,4*S*,5*S*,6*R*)-3,4,5-tris(benzyloxy)-6-((benzyloxy)methyl)tetrahydro-2H-pyran-2-ol (**11**): To a stirred solution of **10** (130.64 g, 0.236 mol) in AcOH (784 mL) heated at 80 °C was added dropwise 5 M HCl (130 mL), and, after addition, the reaction mixture was stirred at this temperature until completion of the reaction as indicated by TLC (typically within 1 h). Upon completion, the reaction mixture was quickly poured into ice water (700 mL), and the resulting mixture was extracted with EtOAc (600 mL × 3). The combined extracts were washed successively with saturated aqueous NaHCO_3_ (until pH > 7) and brine, dried (MgSO_4_), and evaporated on a rotary evaporator to afford a residue, which was purified through column chromatography (EtOAc/PE = 1/6 by *v*/*v*) to give **11**. Yellow oil, 74.66 g. This sample was used directly in the next step without characterization.

Synthesis of (3*R*,4*S*,5*S*,6*R*)-3,4,5-tris(benzyloxy)-6-((benzyloxy)methyl)tetrahydro-2H-pyran-2-one (**12**): Following general synthetic procedure 1, **12** was prepared from **11** (74.66 g, 0.138 mmol) using Ac_2_O (373 mL) and DMSO (750 mL). Column chromatography purification (EtOAc/PE = 1/10 by *v*/*v*) gave **12**. Colorless oil, 38.00 g (30% overall for **10** to **12**). ^1^H NMR (500 MHz, CDCl_3_) *δ* 7.42–7.41 (m, 2H), 7.34–7.27 (m, 16H), 7.24–7.22 (m, 2H), 5.18 (d, *J* = 11.0 Hz, 1H), 4.93 (d, *J* = 11.0 Hz, 1H), 4.79–4.75 (m, 2H), 4.70–4.68 (m, 1H), 4.61 (d, *J* = 11.5 Hz, 1H), 4.52–4.44 (m, 3H), 4.35–4.32 (m, 1H), 4.16 (t, *J* = 2.0 Hz, 1H), 3.88 (dd, *J* = 9.5, 2.0 Hz, 1H), 3.72–3.64 (m, 2H). The ^1^H NMR data were consistent with those reported [45].

Synthesis of (3*R*,4*S*,5*S*,6*R*)-3,4,5-tris(benzyloxy)-6-((benzyloxy)methyl)-2-(4-iodophenyl)-2-methoxytetrahydro-2H-pyran (**13**): Following the general synthetic procedure 2, **13** was prepared from **12** (25.00 g, 46.4 mmol) and 1,4-diiodobenzene (18.37 g, 55.7 mmol) using *n*-BuLi (34.8 mL, 1.6 M in THF, 55.68 mmol), MeSO_3_H (22.30 g, 0.232 mol), MeOH (112 mL), and dried THF (250 mL). The crude **13** was isolated as a brown oil, 48.41 g, which was used in the next step without further purification or characterization.

Synthesis of (2*R*,3*S*,4*R*,5*S*,6*S*)-3,4,5-tris(benzyloxy)-2-((benzyloxy)methyl)-6-(4-iodophenyl)tetrahydro-2H-pyran (**14**): Following the general synthetic procedure 3, **14** was prepared from crude **13** prepared above (48.41 g, deemed to be 64.0 mmol) using Et_3_SiH (14.88 g, 0.128 mol) and BF_3_·Et_2_O (10.90 g, 76.8 mmol) in dried CH_2_Cl_2_ (480 mL). Column chromatography purification (EtOAc/PE = 1/10 by *v*/*v*) gave **14**. Brown oil, 16.20 g (48% overall for **12** to **14**). ^1^H NMR (500 MHz, CDCl_3_) *δ* 7.64–7.62 (m, 2H), 7.37–7.27 (m, 14H), 7.24–7.16 (m, 6H), 6.94–6.92 (m, 2H), 5.00 (d, *J* = 11.5 Hz, 1H), 4.78–4.72 (m, 2H), 4.63 (d, *J* = 12.0 Hz, 1H), 4.51 (d, *J* = 10.5 Hz, 1H), 4.47–4.40 (m, 2H), 4.13 (d, *J* = 9.0 Hz, 1H), 4.04 (d, *J* = 3.0 Hz, 1H), 3.94 (d, *J* = 10.5 Hz, 1H), 3.84 (t, *J* = 9.5 Hz, 1H), 3.69–3.67 (m, 2H), 3.64–3.57 (m, 2H). ^13^C NMR (151 MHz, CDCl_3_) *δ* 139.46, 139.13, 138.57, 137.99, 137.92, 137.31, 129.96, 128.57, 128.55, 128.44, 128.39, 128.36, 128.10, 127.93, 127.91, 127.77, 127.74, 127.63, 93.78, 84.44, 81.60, 80.43, 75.33, 74.70, 74.36, 73.67, 72.67, 68.94. HR-MS (ESI): *m*/*z* [M+H]^+^ calcd for C_40_H_40_IO_5_^+^: 727.1915, found: 727.1923.

Synthesis of (2*R*,3*R*,4*S*,5*S*,6*S*)-3,4,5-tris(benzyloxy)-2-((benzyloxy)methyl)-6-methoxytetrahydro-2H-pyran (**16**): Following the procedure for the synthesis of **10** from **9**, **16** was prepared from **15** (50.00 g, 0.257 mol) using NaH (61.80 g, 60%, 1.54 mol) and BnBr (264.00 g, 1.54 mol) in a mixture of dried DMF (400 mL) and dried THF (100 mL). Column chromatography purification (EtOAc/PE = 1/8 by *v*/*v*) gave **16**. Yellow oil, 109.46 g (77%). ^1^H NMR (500 MHz, CDCl_3_) *δ* 7.38–7.26 (m, 16H), 7.24–7.23 (m, 2H), 7.17–7.15 (m, 2H), 4.88 (d, *J* = 11.0 Hz, 1H), 4.77–4.76 (m, 1H), 4.74–4.70 (m, 2H), 4.66 (d, *J* = 12.0 Hz, 1H), 4.61 (s, 2H), 4.57–4.50 (m, 2H), 3.97 (t, *J* = 9.3 Hz, 1H), 3.89–3.87 (m, 1H), 3.79–3.72 (m, 4H), 3.32 (s, 3H). The ^1^H NMR data were consistent with those reported [46].

Synthesis of (3*S*,4*S*,5*R*,6*R*)-3,4,5-tris(benzyloxy)-6-((benzyloxy)methyl)tetrahydro-2H-pyran-2-ol (**17**): Following the procedure for the synthesis of **11** from **10**, **17** was prepared from **16** (109.46 g, 0.197 mol) using 5 M HCl (110 mL) in AcOH (660 mL). Column chromatography purification (EtOAc/PE = 1/6 by *v*/*v*) gave **17**. Yellow oil, 42.23 g. This sample was used directly in the next step without characterization.

Synthesis of (3*S*,4*S*,5*R*,6*R*)-3,4,5-tris(benzyloxy)-6-((benzyloxy)methyl)tetrahydro-2H-pyran-2-one (**18**): Following the general synthetic procedure 1, **18** was prepared from **17** (42.23 g, 78.1 mmol) using Ac_2_O (211 mL) and DMSO (422 mL). Column chromatography purification (EtOAc/PE = 1/8 by *v*/*v*) gave **18**. Colorless oil, 37.00 g (35% overall for **16** to **18**). ^1^H NMR (500 MHz, CDCl_3_) *δ* 7.40–7.29 (m, 18H), 7.10–7.08 (m, 2H), 5.06 (d, *J* = 12.0 Hz, 1H), 4.83 (d, *J* = 12.0 Hz, 1H), 4.65–4.58 (m, 2H), 4.56–4.51 (m, 2H), 4.35–4.32 (m, 2H), 4.26–4.22 (m, 2H), 4.05–4.04 (m, 1H), 3.79–3.77 (m, 1H), 3.63 (d, *J* = 4.5 Hz, 2H). The ^1^H NMR data were consistent with those reported [47].

Synthesis of (3*S*,4*S*,5*R*,6*R*)-3,4,5-tris(benzyloxy)-6-((benzyloxy)methyl)-2-(4-iodophenyl)-2-methoxytetrahydro-2H-pyran (**19**): Following the general synthetic procedure 2, **19** was prepared from **18** (22.00 g, 40.8 mmol) and 1,4-diiodobenzene (16.17 g, 49.0 mmol) using *n*-BuLi (30.6 mL, 1.6 M in THF, 48.96 mmol), MeSO_3_H (19.63 g, 0.204 mol), MeOH (98 mL), and dried THF (220 mL). The crude product was isolated as a brown oil, 33.68 g, and used directly in the next step without further purification or characterization.

Synthesis of (2*R*,3*R*,4*R*,5*R*,6*S*)-3,4,5-tris(benzyloxy)-2-((benzyloxy)methyl)-6-(4-iodophenyl)tetrahydro-2H-pyran (**20**): Following the general synthetic procedure 3, **20** was prepared from crude **19** prepared above (33.68 g, deemed to be 44.5 mmol) using Et_3_SiH (10.35 g, 89.0 mmol) and BF_3_·Et_2_O (7.58 g, 53.4 mmol) in dried CH_2_Cl_2_ (337 mL). Column chromatography purification (EtOAc/PE = 1/15 by *v*/*v*) gave **20**. Yellow oil, 7.74 g (26% overall for **18** to **20**). ^1^H NMR (500 MHz, CDCl_3_) *δ* 7.61–7.58 (m, 2H), 7.38–7.27 (m, 13H), 7.23–7.14 (m, 5H), 7.08–7.07 (m, 2H), 6.90–6.89 (m, 2H), 4.91 (d, *J* = 10.5 Hz, 1H), 4.76–4.69 (m, 3H), 4.62–4.54 (m, 3H), 4.40 (s, 1H), 4.22 (d, *J* = 11.5 Hz, 1H), 4.01 (t, *J* = 9.5 Hz, 1H), 3.89–3.88 (m, 1H), 3.82–3.77 (m, 3H), 3.61–3.58 (m, 1H). ^13^C NMR (151 MHz, CDCl_3_) *δ* 138.84, 138.56, 138.46, 138.42, 138.08, 137.10, 128.66, 128.61, 128.51, 128.47, 128.23, 128.20, 128.17, 128.02, 127.85, 127.83, 127.67, 127.50, 92.84, 85.02, 80.03, 79.41, 75.43, 75.07, 74.57, 73.65, 72.52, 69.71. HR-MS (ESI): *m*/*z* [M+Na]^+^ calcd for C_40_H_39_INaO_5_^+^: 749.1734, found: 749.1732.

Synthesis of (2*R*,3*S*,4*R*)-3,4-bis(benzyloxy)-2-((benzyloxy)methyl)-3,4-dihydro-2H-pyran (**22**): To a stirred mixture of **21** (33.00 g, 0.226 mol) in dried THF (462 mL) cooled at 0 °C under N_2_ were added NaH (32.5 g, 60%, 0.813 mol) portion-wise and TBAI (5.00 g, 13.5 mmol) in one portion, and, after additions, stirring was continued for 0.5 h, followed by dropwise addition of BnBr (139.06 g, 0.813 mol). After that, the reaction mixture was stirred at room temperature overnight, when TLC analysis indicated completion of the reaction, and then poured *slowly* into stirred ice water (1000 mL). The resulting mixture was extracted with EtOAc (300 mL × 3), and the combined extracts were washed with brine, dried (MgSO_4_), and evaporated on a rotary evaporator to afford a residue, which was purified through column chromatography (EtOAc/PE = 1/12 by *v*/*v*) to give **22**. White solid, 48.00 g (51%). m.p. 52.8–55.2 °C. ^1^H NMR (500 MHz, CDCl_3_) *δ* 7.33–7.27 (m, 13H), 7.25–7.23 (m, 2H), 6.42 (d, *J* = 6.0 Hz, 1H), 4.87 (dd, *J* = 6.3, 2.8 Hz, 1H), 4.83 (d, *J* = 11.5 Hz, 1H), 4.65–4.62 (m, 2H), 4.61–4.54 (m, 3H), 4.21 (d, *J* = 5.5 Hz, 1H), 4.08–4.05 (m, 1H), 3.87–3.84 (m, 1H), 3.82–3.75 (m, 2H). The ^1^H NMR data were consistent with those reported [48].

Synthesis of (3*R*,4*S*,5*R*,6*R*)-4,5-bis(benzyloxy)-6-((benzyloxy)methyl)-3-fluorotetrahydro-2H-pyran-2-ol (**23**): A mixture of **22** (7.70 g, 18.5 mmol) and selectfluor^®^ (26.20 g, 73.9 mmol) in a mixture of DMF (39 mL) and H_2_O (39 mL) was stirred at 50 °C overnight when TLC analysis indicated completion of the reaction. Upon cooling to room temperature, the reaction mixture was poured into ice water (90 mL), and the resulting mixture was extracted with EtOAc (80 mL × 3). The combined extracts were washed with brine, dried (MgSO_4_), and evaporated on a rotary evaporator to afford crude **23** as a yellow oil, 10.90 g, which was used directly in the next step without further purification or characterization.

Synthesis of (3*R*,4*S*,5*R*,6*R*)-4,5-bis(benzyloxy)-6-((benzyloxy)methyl)-3-fluorotetrahydro-2H-pyran-2-one (**24**): Following the general synthetic procedure 1, **24** was prepared from crude **23** prepared above (10.90 g, deemed to be 24.1 mmol) using Ac_2_O (55 mL) and DMSO (109 mL). Column chromatography purification (EtOAc/PE = 1/6 by *v*/*v*) gave **24**. White solid, 1.39 g (17% overall for **22** to **24**). m.p. 58.8–60.2 °C. ^1^H NMR (600 MHz, DMSO-*d*_6_) *δ* 7.35–7.29 (m, 13H), 7.23–7.22 (m, 2H), 5.38 (dd, *J* = 46.8, 8.4 Hz, 1H), 4.76–4.71 (m, 3H), 4.57–4.53 (m, 3H), 4.51–4.49 (m, 1H), 4.26–4.21 (m, 1H), 3.98 (t, *J* = 7.8 Hz, 1H), 3.70 (d, *J* = 3.6 Hz, 2H). The m.p. and ^1^H NMR were consistent with those reported [38].

Synthesis of (3*R*,4*S*,5*R*,6*R*)-4,5-bis(benzyloxy)-6-((benzyloxy)methyl)-3-fluoro-2-(4-iodophenyl)-2-methoxytetrahydro-2H-pyran (**25**): Following the general synthetic procedure 2, **25** was prepared from **24** (0.50 g, 1.11 mmol) and 1,4-diiodobenzene (0.44 g, 1.33 mmol) using *n*-BuLi (0.83 mL, 1.6 M in THF, 1.328 mmol), MeSO_3_H (0.53 g, 5.55 mmol), MeOH (2.7 mL), and dried THF (5 mL). The crude **25** was isolated as a yellow oil, 0.24 g, which was used directly in the next step without further purification or characterization.

Synthesis of (2*R*,3*R*,4*S*,5*S*,6*S*)-3,4-bis(benzyloxy)-2-((benzyloxy)methyl)-5-fluoro-6-(4-iodophenyl)tetrahydro-2H-pyran (**26**): Following the general synthetic procedure 3, **26** was prepared from crude **25** prepared above (0.24 g, deemed to be 0.359 mmol) using Et_3_SiH (83 mg, 0.718 mmol) and BF_3_·Et_2_O (61 mg, 0.431 mmol) in dried CH_2_Cl_2_ (2.4 mL). Column chromatography purification (EtOAc/PE = 1/20 by *v*/*v*) gave **26**. Yellow oil, 94 mg (13% overall for **24** to **26**). ^1^H NMR (500 MHz, CDCl_3_) *δ* 7.76 (d, *J* = 8.0 Hz, 2H), 7.41–7.27 (m, 15H), 7.21 (d, *J* = 8.0 Hz, 2H), 4.96 (t, *J* = 10.3 Hz, 2H), 4.85–4.82 (m, 1H), 4.67–4.65 (m, 2H), 4.60–4.58 (m, 1H), 4.50–4.39 (m, 1H), 4.35–4.32 (m, 1H), 3.98–3.92 (m, 1H), 3.82–3.79 (m, 3H), 3.68–3.65 (m, 1H). ^13^C NMR (151 MHz, CDCl_3_) *δ* 138.25, 138.19, 138.11, 137.57, 137.48, 129.03, 128.52, 128.51, 128.49, 128.10, 127.94, 127.89, 127.81, 127.76, 95.57, 94.32 (d, *J* = 5.1 Hz), 84.6 (d, *J* = 16.3 Hz), 79.39, 78.74 (d, *J* = 23.2 Hz), 75.34, 75.00 (d, *J* = 3.0 Hz), 73.56, 68.93. HR-MS (ESI): *m*/*z* [M+H]^+^ calcd for C_33_H_33_FIO_4_^+^: 639.1402, found: 639.1407.

Synthesis of 1-(*tert*-butyldimethylsilyl)-1,2-dicarba-*closo*-dodecaborane (**28**): To a stirred solution of *o*-carborane (**27**, 0.20 g, 1.39 mmol) in dried THF (1.6 mL) cooled at 0 °C under N_2_ was added *n*-BuLi (1 mL, 1.6 M in THF, 1.6 mmol), and, after addition, the reaction mixture was stirred at room temperature for 1 h and cooled back to 0 °C again, followed by the addition of a solution of TBDMSCl (0.27 g, 1.80 mmol) in dried THF (0.4 mL). After that, the reaction mixture was stirred at room temperature overnight, when TLC analysis indicated completion of the reaction, and then poured into ice water (20 mL). The mixture thus obtained was extracted with EtOAc (30 mL × 3), and the combined extracts were washed with brine, dried (MgSO_4_), and evaporated on a rotary evaporator to afford a residue, which was purified through column chromatography (PE) to give **28**. White solid, 0.29 g (81%). m.p. 60.8–62.2 °C. ^1^H NMR (500 MHz, CDCl_3_) *δ* 3.44 (s, 1H), 2.85–1.50 (m, 10H), 1.02 (s, 9H), 0.23 (s, 6H). The m.p. and ^1^H NMR were consistent with those reported [49].

Synthesis of 1-*tert*-butyl-2-[(4-iodophenyl)methyl]-1,2-dicarba-*closo*-dodecaborane (**29**): To a stirred solution of **28** (0.50 g, 1.93 mmol) in dried THF (5 mL) cooled at 0 °C under N_2_ was added dropwise *n*-BuLi (1.5 mL, 1.6 M in THF, 2.4 mmol), and, after addition, the reaction mixture was stirred at room temperature for 1 h and cooled back to 0 °C again, followed by dropwise addition of 4-iodobenzyl bromide (0.69 g, 2.32 mmol) in dried THF. After that, the reaction mixture was stirred at room temperature overnight, when TLC analysis indicated completion of the reaction, and then poured into ice water (30 mL). The resulting mixture was extracted with EtOAc (30 mL × 3), and the combined extracts were washed with brine, dried (MgSO_4_), and evaporated on a rotary evaporator to afford a residue, which was purified through column chromatography (PE) to give **29**. White solid, 0.28 g (31%). m.p. 124.0–125.8 °C. ^1^H NMR (500 MHz, CDCl_3_) *δ* 7.68–7.65 (m, 2H), 6.91–6.89 (m, 2H), 3.40 (s, 2H), 2.68–1.81 (m, 10H), 1.13 (s, 9H), 0.42 (s, 6H). ^13^C NMR (151 MHz, CDCl_3_) *δ* 137.94, 135.47, 131.84, 93.95, 80.54, 75.25, 75.24, 75.22, 43.12, 27.84, 20.66, −2.02. HR-MS (ESI): *m*/*z* [M+H]^+^ calcd for C_15_H_32_B_10_ISi^+^: 477.2243, found: 477.2267.

Synthesis of (3*R*,4*S*,5*S*,6*R*)-6-(hydroxymethyl)-2-methoxy-2-(4-((2-*tert*-butyldimethylsilyl-1,2-dicarba-*closo*-dodecaboran-1-yl)methyl)phenyl)tetrahydro-2H-pyran-3,4,5-triol (**30**): To a stirred solution of **29** (4.27 g, 9.00 mmol) in dried THF (43 mL) cooled at −78 °C under N_2_ was added dropwise *n*-BuLi (6.7 mL, 1.6 M in THF, 10.72 mmol), and, after addition, the reaction mixture was stirred at this temperature for 1 h, followed by dropwise addition of a solution of **2** (8.40 g, 18.0 mmol) in dried THF (10 mL). After that, the reaction mixture was stirred at this temperature for 1 h, followed by dropwise addition of a solution of MeSO_3_H (4.32 g, 45.0 mmol) in MeOH (22 mL). After that, the reaction mixture was stirred at room temperature overnight, when TLC analysis indicated completion of the reaction, and then poured into ice water (150 mL). The resulting mixture was extracted with EtOAc (50 mL × 3), and the combined extracts were washed with brine, dried (MgSO_4_), and evaporated on a rotary evaporator to afford crude **30** as a brown foam, 5.80 g, which was used directly in the next step without further purification.

Synthesis of (3*R*,4*S*,5*R*,6*R*)-6-(acetoxymethyl)-2-methoxy-2-(4-((2-*tert*-butyldimethylsilyl-1,2-dicarba-*closo*-dodecaboran-1-yl)methyl)phenyl)tetrahydro-2H-pyran-3,4,5-triyl triacetate (**31**): To a stirred mixture of crude **30** prepared above (5.80 g, deemed to be 10.7 mmol) and DMAP (1.31 g, 10.7 mmol) in pyridine (58 mL) cooled at 0 °C was added dropwise Ac_2_O (29 mL), and, after addition, the reaction mixture was stirred at room temperature overnight, when TLC analysis indicated completion of the reaction, and then poured into ice water (70 mL). The resulting mixture was extracted with EtOAc (80 mL × 3), and the combined extracts were washed successively with 1 M HCl (until pH = 5–6) and brine, dried (MgSO_4_), and evaporated on a rotary evaporator to afford crude **31** as a brown oil, 7.06 g, which was used directly in the next step without further purification or characterization.

Synthesis of (2*R*,3*R*,4*R*,5*S*,6*S*)-2-(acetoxymethyl)-6-(4-((2-*tert*-butyldimethylsilyl-1,2-dicarba-*closo*-dodecaboran-1-yl)methyl)phenyl)tetrahydro-2H-pyran-3,4,5-triyl triacetate (**32**): Following the general synthetic procedure 3, **32** was prepared from crude **31** prepared above (7.06 g, deemed to be 9.96 mmol) using Et_3_SiH (3.47 g, 29.9 mmol) and BF_3_·Et_2_O (7.35 g, 51.8 mmol) in dried CH_2_Cl_2_ (70 mL). Column chromatography purification (EtOAc/PE = 1/3 by *v*/*v*) gave **32**. White foam, 1.50 g (25% overall for **29** to **32**). ^1^H NMR (500 MHz, CDCl_3_) *δ* 7.31 (d, *J* = 8.5 Hz, 2H), 7.14 (d, *J* = 8.0 Hz, 2H), 5.35–5.30 (m, 1H), 5.23 (t, *J* = 9.8 Hz, 1H), 5.10 (t, *J* = 9.8 Hz, 1H), 4.40 (d, *J* = 10.0 Hz, 1H), 4.32–4.28 (m, 1H), 4.19–4.11 (m, 1H), 3.86–3.83 (m, 1H), 3.45 (s, 2H), 2.89–1.51 (m, 10H), 2.10 (s, 3H), 2.06 (s, 3H), 2.00 (s, 3H), 1.79 (s, 3H), 1.14 (s, 9H), 0.43 (s, 6H). ^13^C NMR (126 MHz, CDCl_3_) *δ* 170.86, 170.47, 169.64, 168.92, 136.57, 136.32, 130.03, 127.56, 80.99, 80.00, 77.36, 76.34, 75.22, 74.21, 72.74, 68.70, 62.43, 43.31, 27.82, 20.91, 20.76, 20.62, 20.43, −2.04, −2.07. HR-MS (ESI): *m*/*z* [M+H]^+^ calcd for C_29_H_51_B_10_O_9_Si^+^: 681.4227, found: 681.4238.

Synthesis of (2*R*,3*R*,4*R*,5*S*,6*S*)-2-(acetoxymethyl)-6-(4-((1,2-dicarba-*closo*-dodecaboran-1-yl)methyl)phenyl)tetrahydro-2H-pyran-3,4,5-triyl triacetate (**33**): To a stirred solution of **32** (0.75 g, 1.10 mmol) in dried THF (7.5 mL) cooled at −78 °C under N_2_ was added dropwise a solution of TBAF in THF (1.7 mL, 1.0 M in THF, 1.7 mmol), and, after addition, the reaction mixture was stirred at room temperature for 1 h, when TLC analysis indicated completion of the reaction, and then poured into ice water (30 mL). The resulting mixture was extracted with EtOAc (30 mL × 3), and the combined extracts were washed with brine, dried (MgSO_4_), and evaporated on a rotary evaporator to afford a residue, which was purified through column chromatography (EtOAc/PE = 1/2 by *v*/*v*) to obtain **33**. White solid, 0.44 g (71%). m.p. 192.2–195.9 °C. ^1^H NMR (500 MHz, CDCl_3_) *δ* 7.34 (d, *J* = 8.0 Hz, 2H), 7.12 (d, *J* = 8.0 Hz, 2H), 5.34 (t, *J* = 9.5 Hz, 1H), 5.22 (t, *J* = 9.7 Hz, 1H), 5.02 (t, *J* = 9.8 Hz, 1H), 4.39 (d, *J* = 9.5 Hz, 1H), 4.32–4.29 (m, 1H), 4.21–4.18 (m, 1H), 3.87–3.84 (m, 1H), 3.51 (s, 2H), 3.13 (s, 1H), 2.76–1.63 (m, 10H), 2.10 (s, 3H), 2.06 (s, 3H), 2.00 (s, 3H), 1.79 (s, 3H). ^13^C NMR (151 MHz, CDCl_3_) *δ* 170.87, 170.45, 169.67, 168.92, 137.04, 135.03, 130.00, 127.84, 79.92, 76.39, 74.31, 73.93, 73.03, 68.68, 62.38, 59.46, 43.26, 20.94, 20.78, 20.48. HR-MS (ESI): *m*/*z* [M+H]^+^ calcd for C_23_H_37_B_10_O_9_^+^: 567.3363, found: 567.3361.

Synthesis of (2*R*,3*S*,4*R*,5*R*,6*S*)-2-(hydroxymethyl)-6-(4-((1,2-dicarba-*closo*-dodecaboran-1-yl)methyl)phenyl)tetrahydro-2H-pyran-3,4,5-triol (**A1**): Following the general synthetic procedure 5, **A1** was prepared from **33** (0.40 g, 0.708 mmol) using Na (98 mg, 4.25 mmol) in MeOH (8 mL). Column chromatography purification (MeOH/CH_2_Cl_2_ = 1/10 by *v*/*v*) gave **A1**. White foam, 0.25 g (89%). [α]_D_^20^ = +10.2° (*c* = 10.4, MeOH). ^1^H NMR (500 MHz, CD_3_OD) *δ* 7.43 (d, *J* = 8.0 Hz, 2H), 7.19 (d, *J* = 7.5 Hz, 2H), 4.24 (s, 1H), 4.15 (d, *J* = 9.5 Hz, 1H), 3.90–3.88 (m, 1H), 3.72–3.69 (m, 1H), 3.58 (s, 2H), 3.50–3.47 (m, 1H), 3.42–3.41 (m, 2H), 3.38–3.35 (m, 1H), 2.57–1.63 (m, 10H). ^13^C NMR (151 MHz, CD_3_OD) *δ* 140.94, 136.23, 130.85, 129.35, 83.18, 82.27, 79.81, 76.92, 76.41, 71.91, 63.13, 62.71, 43.98. ^11^B NMR (161 MHz, CD_3_OD) *δ* −2.35, −3.27, −5.39, −6.31,−8.90, −9.83, −10.57, −11.59, −12.56, −13.40. HR-MS (ESI): *m*/*z* [M-H]^−^ calcd for C_15_H_27_B_10_O_5_^−^: 397.2795, found: 397.2776. HPLC purity, 97.84%.

Synthesis of (3*R*,4*S*,5*S*,6*R*)-3,4,5-tris(benzyloxy)-6-((benzyloxy)methyl)-2-methoxy-2-(4-((2-*tert*-butyldimethylsilyl-1,2-dicarba-*closo*-dodecaboran-1-yl)methyl)phenyl)tetrahydro-2H-pyran (**34**): Following the general synthetic procedure 2, **34** was prepared from **29** (3.78 g, 7.97 mmol) and **12** (6.44 g, 11.9 mmol) using *n*-BuLi (5.5 mL, 1.6 M in THF, 8.8 mmol), MeSO_3_H (3.83 g, 39.8 mmol), MeOH (19.2 mL), and dried THF (38 mL) as a crude sample. Brown oil, 10.22 g. This sample was used directly in the next step without further purification or characterization.

Synthesis of (2*R*,3*S*,4*R*,5*S*,6*S*)-3,4,5-tris(benzyloxy)-2-((benzyloxy)methyl)-6-(4-((2-*tert*-butyldimethylsilyl-1,2-dicarba-*closo*-dodecaboran-1-yl)methyl)phenyl)tetrahydro-2H-pyran (**35**): Following the general synthetic procedure 3, **35** was prepared from crude **34** prepared above (10.22 g, deemed to be 11.3 mmol) using Et_3_SiH (2.64 g, 22.7 mmol) and BF_3_·Et_2_O (1.93 g, 13.6 mmol) in dried CH_2_Cl_2_ (102 mL). Column chromatography purification (EtOAc/PE = 1/10 by *v*/*v*) gave **35**. White foam, 2.83 g (41%, **29** to **35**). ^1^H NMR (500 MHz, CDCl_3_) *δ* 7.45–7.43 (m, 2H), 7.39–7.27 (m, 15H), 7.21–7.19 (m, 3H), 7.15–7.13 (m, 2H), 6.99–6.97 (m, 2H), 5.03 (d, *J* = 12.0 Hz, 1H), 4.79 (s, 2H), 4.65 (d, *J* = 11.5 Hz, 1H), 4.51–4.48 (m, 1H), 4.46–4.42 (m, 2H), 4.21 (d, *J* = 9.5 Hz, 1H), 4.07–4.06 (m, 1H), 3.90 (t, *J* = 9.3 Hz, 1H), 3.81 (d, *J* = 10.0 Hz, 1H), 3.72–3.70 (m, 2H), 3.68–3.61 (m, 2H), 3.47 (s, 2H), 2.98–1.71 (m, 10H), 1.14 (s, 9H), 0.44 (s, 6H). ^13^C NMR (151 MHz, CDCl_3_) *δ* 139.78, 139.22, 138.70, 138.04, 135.73, 129.73, 128.56, 128.42, 128.37, 128.36, 128.33, 128.10, 127.91, 127.73, 127.69, 127.61, 127.56, 84.29, 81.84, 81.34, 80.95, 75.40, 75.23, 74.73, 74.54, 73.67, 72.74, 68.96, 43.43, 27.87, 20.66, −1.95, −2.04. HR-MS (ESI): *m*/*z* [M+H]^+^ calcd for C_49_H_67_B_10_O_5_Si^+^: 873.5683, found: 873.5743.

Synthesis of (2*R*,3*S*,4*R*,5*S*,6*S*)-3,4,5-tris(benzyloxy)-2-((benzyloxy)methyl)-6-(4-((1,2-dicarba-*closo*-dodecaboran-1-yl)methyl)phenyl)tetrahydro-2H-pyran (**36**): Following the procedure for the synthesis of **33** from **32**, **36** was prepared from **35** (0.59 g, 0.677 mmol) using TBAF (1 mL, 1.0 M in THF) in dried THF (5.9 mL). Column chromatography purification (EtOAc/PE = 1/8 by *v*/*v*) gave **36**. Pale yellow oil, 0.29 g (57%). ^1^H NMR (500 MHz, CDCl_3_) *δ* 7.47 (d, *J* = 8.0 Hz, 2H), 7.38–7.33 (m, 8H), 7.32–7.27 (m, 7H), 7.21–7.18 (m, 3H), 7.11 (d, *J* = 8.0 Hz, 2H), 6.94–6.92 (m, 2H), 5.02 (d, *J* = 11.5 Hz, 1H), 4.80–4.74 (m, 2H), 4.65 (d, *J* = 11.5 Hz, 1H), 4.53 (d, *J* = 10.5 Hz, 1H), 4.49–4.42 (m, 2H), 4.22 (d, *J* = 9.0 Hz, 1H), 4.08–4.07 m, 1H), 3.89 (t, *J* = 9.5 Hz, 1H), 3.80 (d, *J* = 10.0 Hz, 1H), 3.73–3.70 (m, 2H), 3.66–3.60 (m, 2H), 3.52 (s, 2H), 3.17 (s, 1H), 2.60–1.70 (m, 10H). ^13^C NMR (151 MHz, CDCl_3_) *δ* 140.20, 139.13, 138.58, 137.97, 137.95, 134.14, 129.70, 128.78, 128.57, 128.40, 128.36, 128.15, 128.11, 127.97, 127.93, 127.78, 127.63, 84.28, 81.70, 80.75, 77.42, 75.33, 74.76, 74.53, 74.42, 73.68, 72.69, 68.87, 59.37, 43.40. HR-MS (ESI): *m*/*z* [M+H]^+^ calcd for C_43_H_53_B_10_O_5_^+^: 759.4818, found: 759.4868.

Synthesis of (2*R*,3*S*,4*R*,5*S*,6*S*)-2-(acetoxymethyl)-6-(4-((1,2-dicarba-*closo*-dodecaboran-1-yl)methyl)phenyl)tetrahydro-2H-pyran-3,4,5-triyl triacetate (**37**): To a stirred solution of **36** (1.16 g, 1.53 mmol) in Ac_2_O (11.6 mL) cooled at 0 °C under N_2_ was added dropwise BF_3_·Et_2_O (11.6 mL), and, after addition, the reaction mixture was stirred at 50 °C overnight, when TLC analysis indicated completion of the reaction. Upon cooling to room temperature, the reaction mixture was poured into ice water (50 mL), and the resulting mixture was extracted with EtOAc (70 mL × 3). The combined extracts were washed with brine, dried (MgSO_4_), and evaporated on a rotary evaporator to afford a residue, which was purified through column chromatography (EtOAc/PE = 1/4 by *v*/*v*) to give **37**. Yellow foam, 0.36 g (42%). ^1^H NMR (500 MHz, CDCl_3_) *δ* 7.38 (d, *J* = 8.0 Hz, 2H), 7.13 (d, *J* = 8.0 Hz, 2H), 5.54–5.53 (m, 1H), 5.24–5.17 (m, 2H), 4.35 (d, *J* = 8.5 Hz, 1H), 4.23–4.16 (m, 2H), 4.10–4.06 (m, 1H), 3.55–3.49 (m, 2H), 3.13 (s, 1H), 2.86–1.64 (m, 10H), 2.21 (s, 3H), 2.04 (s, 3H), 1.98 (s, 3H), 1.79 (s, 3H). ^13^C NMR (151 MHz, CDCl_3_) *δ* 170.59, 170.37, 170.27, 169.06, 137.38, 134.92, 129.87, 127.97, 80.46, 74.83, 74.36, 71.81, 70.44, 67.84, 61.82, 59.48, 43.19, 20.84, 20.73, 20.57. HR-MS (ESI): *m*/*z* [M+H]^+^ calcd for C_23_H_37_B_10_O_9_^+^: 567.3363, found: 567.3369.

Synthesis of (2*R*,3*R*,4*R*,5*R*,6*S*)-2-(hydroxymethyl)-6-(4-((1,2-dicarba-*closo*-dodecaboran-1-yl)methyl)phenyl)tetrahydro-2H-pyran-3,4,5-triol (**A2**): Following the general synthetic procedure 5, **A2** was prepared from **37** (0.36 g, 0.638 mmol) using Na (59 mg, 2.55 mmol) in dried MeOH (7.2 mL). Column chromatography purification (MeOH/CH_2_Cl_2_ = 1/10 by *v*/*v*) gave **A2**. White solid, 0.22 g (87%). m.p. 108.6–110.9 °C. [α]_D_^20^ = +20.0° (*c* = 10, MeOH). ^1^H NMR (500 MHz, DMSO-*d*_6_) *δ* 7.34–7.32 (m, 2H), 7.16–7.14 (m, 2H), 5.11 (s, 1H), 4.71 (d, *J* = 5.5 Hz, 1H), 4.61–4.60 (m, 1H), 4.57 (t, *J* = 5.3 Hz, 1H), 4.41–4.40 (m, 1H), 3.96 (d, *J* = 9.5 Hz, 1H), 3.77–3.75 (m, 1H), 3.57 (s, 2H), 3.56–3.47 (m, 4H), 3.42–3.38 (m, 1H), 2.85–1.47 (m, 10H). ^13^C NMR (151 MHz, DMSO-*d*_6_) *δ* 140.33, 134.53, 129.28, 127.99, 81.70, 79.38, 76.59, 75.03, 71.53, 68.90, 62.83, 60.89, 41.80. ^11^B NMR (161 MHz, DMSO-*d*_6_) *δ* −2.75, −3.68, −7.18, −9.32, −9.40, −10.22, −10.27, −12.10. HR-MS (ESI): *m*/*z* [M+Na]^+^ calcd for C_15_H_28_B_10_NaO_5_^+^: 421.2760, found: 421.2742. HPLC purity, 97.90%.

Synthesis of (3*S*,4*S*,5*R*,6*R*)-3,4,5-tris(benzyloxy)-6-((benzyloxy)methyl)-2-methoxy-2-(4-((2-*tert*-butyldimethylsilyl-1,2-dicarba-*closo*-dodecaboran-1-yl)methyl)phenyl)tetrahydro-2H-pyran (**38**): Following the general synthetic procedure 2, **38** was prepared from **29** (3.00 g, 6.32 mmol) and **18** (5.11 g, 9.48 mmol) using *n*-BuLi (4.3 mL, 1.6 M in THF, 6.88 mmol), MeSO_3_H (3.04 g, 31.6 mmol), MeOH (15.2 mL), and dried THF (30 mL) as a crude sample. Yellow oil, 7.75 g. This sample was used directly in the next step without further purification or characterization.

Synthesis of (2*R*,3*R*,4*R*,5*R*,6*S*)-3,4,5-tris(benzyloxy)-2-((benzyloxy)methyl)-6-(4-((2-*tert*-butyldimethylsilyl-1,2-dicarba-*closo*-dodecaboran-1-yl)methyl)phenyl)tetrahydro-2H-pyran (**39**): Following the general synthetic procedure 3, **39** was prepared from crude **38** prepared above (7.75 g, deemed to be 8.60 mmol) using Et_3_SiH (2.00 g, 17.2 mmol) and BF_3_·Et_2_O (1.46 g, 10.3 mmol) in dried CH_2_Cl_2_ (78 mL). Column chromatography purification (EtOAc/PE = 1/13 by *v*/*v*) gave **39**. Yellow oil, 2.13 g (39% overall for **29** to **39**). ^1^H NMR (500 MHz, CDCl_3_) *δ* 7.38–7.27 (m, 16H), 7.24–7.22 (m, 2H), 7.19–7.17 (m, 2H), 7.11 (d, *J* = 8.0 Hz, 2H), 7.06–7.04 (m, 2H), 4.93 (d, *J* = 10.5 Hz, 1H), 4.75–4.69 (m, 3H), 4.62 (t, *J* = 11.0 Hz, 2H), 4.49–4.45 (m, 2H), 4.08–4.02 (m, 2H), 3.96–3.95 (m, 1H), 3.87–3.79 (m, 3H), 3.63–3.60 (m, 1H), 3.47 (s, 2H), 2.98–1.75 (m, 10H), 1.15 (s, 9H), 0.44 (s, 6H). ^13^C NMR (151 MHz, CDCl_3_) *δ* 139.13, 138.66, 138.55, 138.52, 138.45, 135.04, 129.59, 128.57, 128.50, 128.47, 128.27, 128.15, 128.09, 128.00, 127.80, 127.78, 127.64, 127.40, 126.97, 84.77, 81.40, 80.05, 79.56, 78.15, 75.41, 75.25, 75.02, 74.88, 73.74, 72.26, 69.80, 43.41, 27.88, 20.67, −1.98, −1.99. HR-MS (ESI): *m*/*z* [M-H]^−^ calcd for C_49_H_65_B_10_O_5_Si^−^: 871.5537, found: 871.5599.

Synthesis of (2*R*,3*R*,4*R*,5*R*,6*S*)-3,4,5-tris(benzyloxy)-2-((benzyloxy)methyl)-6-(4-((1,2-dicarba-*closo*-dodecaboran-1-yl)methyl)phenyl)tetrahydro-2H-pyran (**40**): Following the procedure for the synthesis of **36** from **35**, **40** was prepared from **39** (2.00 g, 2.30 mmol) using TBAF (3.4 mL, 1.0 M in THF, 3.4 mmol) in dried THF (20 mL). Column chromatography purification (EtOAc/PE = 1/10 by *v*/*v*) gave **40**. Yellow oil, 1.14 g (66%). ^1^H NMR (500 MHz, CDCl_3_) *δ* 7.39–7.28 (m, 12H), 7.27–7.13 (m, 8H), 7.07 (d, *J* = 8.5 Hz, 2H), 6.97–6.96 (m, 2H), 4.92 (d, *J* = 10.5 Hz, 1H), 4.76–4.71 (m, 3H), 4.63–4.59 (m, 2H), 4.54–4.49 (m, 2H), 4.09 (d, *J* = 11.5 Hz, 1H), 4.03 (t, *J* = 9.5 Hz, 1H), 3.95–3.94 (m, 1H), 3.86–3.79 (m, 3H), 3.64–3.60 (m, 1H), 3.55–3.49 (m, 2H), 3.17 (s, 1H), 2.86–1.64 (m, 10H). ^13^C NMR (151 MHz, CDCl_3_) *δ* 139.60, 138.56, 138.45, 138.43, 138.28, 133.41, 129.52, 128.60, 128.51, 128.48, 128.24, 128.16, 128.05, 127.84, 127.82, 127.69, 127.65, 127.49, 127.39, 84.84, 80.01, 79.44, 77.77, 75.44, 75.02, 74.68, 74.59, 73.70, 72.44, 69.75, 59.37, 43.38. HR-MS (ESI): *m*/*z* [M-H]^−^ calcd for C_43_H_51_B_10_O_5_^−^: 757.4662, found: 757.4716.

Synthesis of (2*R*,3*R*,4*R*,5*R*,6*S*)-2-(acetoxymethyl)-6-(4-((1,2-dicarba-*closo*-dodecaboran-1-yl)methyl)phenyl)tetrahydro-2H-pyran-3,4,5-triyl triacetate (**41**): Following the procedure for the synthesis of **37** from **36**, **41** was prepared from **40** (1.14 g, 1.51 mmol) using Ac_2_O (11.4 mL) and BF_3_·Et_2_O (11.4 mL). Column chromatography purification (EtOAc/PE = 1/4 by *v*/*v*) gave **41**. White foam, 0.63 g (74%). ^1^H NMR (500 MHz, CDCl_3_) *δ* 7.33 (d, *J* = 8.0 Hz, 2H), 7.10 (d, *J* = 7.5 Hz, 2H), 5.51 (d, *J* = 3.5 Hz, 1H), 5.34 (t, *J* = 10.0 Hz, 1H), 5.27–5.24 (m, 1H), 4.79 (s, 1H), 4.36–4.33 (m, 1H), 4.27–4.24 (m, 1H), 3.84–3.81 (m, 1H), 3.50 (s, 2H), 3.15 (s, 1H), 2.87–1.63 (m, 10H), 2.12 (s, 3H), 2.09 (s, 3H), 1.99 (s, 3H), 1.89 (s, 3H). ^13^C NMR (151 MHz, CDCl_3_) *δ* 170.91, 170.37, 169.94, 169.91, 136.86, 134.28, 129.80, 126.85, 78.01, 76.67, 74.40, 72.39, 70.79, 66.13, 63.06, 59.44, 43.29, 20.99, 20.90, 20.78, 20.51. HR-MS (ESI): *m*/*z* [M+H]^+^ calcd for C_23_H_37_B_10_O_9_^+^: 567.3363, found: 567.3378.

Synthesis of (2R,3S,4R,5S,6S)-2-(hydroxymethyl)-6-(4-((1,2-dicarba-*closo*-dodecaboran-1-yl)methyl)phenyl)tetrahydro-2H-pyran-3,4,5-triol (**A3**): Following the general synthetic procedure 5, **A3** was prepared from **41** (0.54 g, 0.956 mmol) using Na (66 mg, 2.87 mmol) in MeOH (10.8 mL). Column chromatography purification (MeOH/CH_2_Cl_2_ = 1/10 by *v*/*v*) gave **A3**. White solid, 0.35 g (92%). m.p. 197.8–198.9 °C. [α]_D_^20^ = +30.4° (*c* = 7.5, MeOH). ^1^H NMR (500 MHz, DMSO-*d*_6_) *δ* 7.37 (d, *J* = 8.0 Hz, 2H), 7.14 (d, *J* = 8.0 Hz, 2H), 5.09 (s, 1H), 4.78 (d, *J* = 5.0 Hz, 1H), 4.69 (d, *J* = 5.5 Hz, 1H), 4.47 (s, 1H), 4.43 (t, *J* = 5.8 Hz, 1H), 4.16 (d, *J* = 5.5 Hz, 1H), 3.78–3.74 (m, 2H), 3.56 (s, 2H), 3.53–3.46 (m, 2H), 3.43–3.38 (m, 1H), 3.21–3.17 (m, 1H), 2.90–1.52 (m, 10H). ^13^C NMR (151 MHz, DMSO-*d*_6_) *δ* 139.82, 133.85, 129.05, 126.91, 81.47, 78.74, 76.68, 75.09, 72.02, 67.11, 62.81, 61.74, 41.83. ^11^B NMR (160 MHz, CD_3_OD) *δ* −2.36, −3.28, −5.55, −6.38, −8.97, −9.90, −10.64, −11.67, −12.69, −13.54. HR-MS (ESI): *m*/*z* [M+Na]^+^ calcd for C_15_H_28_B_10_NaO_5_^+^: 421.2760, found: 421.2761. HPLC purity, 95.05%.

Synthesis of (2*R*,3*R*,4*R*,5*S*,6*S*)-3,4,5-tris(benzyloxy)-2-((benzyloxy)methyl)-6-(4-(1,2-dicarba-*closo*-dodecaboran-1-yl)phenyl)tetrahydro-2H-pyran (**42**): Following the general synthetic procedure 4, **42** was prepared from *o*-carborane (0.83 g, 5.75 mmol) and **8** (3.76 g, 5.18 mmol) using **8** (3.76 g, 5.18 mmol), *n*-BuLi (5.4 mL, 1.6 M in THF, 8.64 mmol), Pd[P(*t*-Bu)_3_]_2_ (0.29 g, 0.575 mmol), PCy_3_ (0.16 g, 0.575 mmol), dried THF (6.6 mL), and dried *m*-xylene (16.6 mL). Column chromatography purification (EtOAc/PE = 1/7 by *v*/*v*) gave **42**. Yellow foam, 0.89 g (21%). ^1^H NMR (500 MHz, CDCl_3_) *δ* 7.45–7.43 (m, 2H), 7.38–7.36 (m, 2H), 7.33–7.32 (m, 8H), 7.31–7.28 (m, 6H), 7.20–7.18 (m, 4H), 6.83–6.81 (m, 2H), 4.95–4.93 (m, 2H), 4.86 (d, *J* = 10.5 Hz, 1H), 4.64–4.60 (m, 2H), 4.55–4.48 (m, 2H), 4.22 (d, *J* = 9.5 Hz, 1H), 3.96 (s, 1H), 3.83–3.74 (m, 5H), 3.59–3.57 (m, 1H), 3.44 (t, *J* = 9.0 Hz, 1H), 2.86–1.48 (m, 10H). ^13^C NMR (151 MHz, CDCl_3_) *δ* 141.42, 138.62, 138.33, 138.19, 137.47, 133.44, 128.63, 128.61, 128.54, 128.42, 128.24, 128.22, 128.13, 127.99, 127.86, 127.80, 127.49, 86.97, 83.88, 80.84, 79.54, 78.40, 76.39, 75.86, 75.32, 75.22, 73.64, 69.18, 60.19. HR-MS (ESI): *m*/*z* [M-H]^−^ calcd for C_42_H_49_B_10_O_5_^−^: 743.4516, found: 743.4540.

Synthesis of (2*R*,3*R*,4*R*,5*S*,6*S*)-2-(acetoxymethyl)-6-(4-(1,2-dicarba-*closo*-dodecaboran-1-yl)phenyl)tetrahydro-2H-pyran-3,4,5-triyl triacetate (**43**): Following the procedure for the synthesis of **37** from **36**, **43** was prepared from **42** (0.42 g, 0.565 mmol), Ac_2_O (4.2 mL), and BF_3_·Et_2_O (4.2 mL). Column chromatography purification (EtOAc/PE = 1/3 by *v*/*v*) gave **43**. White solid, 0.20 g (64%). m.p. 186.7–187.9 °C. ^1^H NMR (500 MHz, CDCl_3_) *δ* 7.48–7.45 (m, 2H), 7.33–7.31 (m, 2H), 5.32 (t, *J* = 9.5 Hz, 1H), 5.22 (t, *J* = 9.5 Hz, 1H), 5.09 (t, *J* = 9.5 Hz, 1H), 4.42 (d, *J* = 9.5 Hz, 1H), 4.29–4.25 (m, 1H), 4.17–4.14 (m, 1H), 3.94 (s, 1H), 3.85–3.81 (m, 1H), 2.78–1.62 (m, 10H), 2.08 (s, 3H), 2.06 (s, 3H), 2.01 (s, 3H), 1.83 (s, 3H). ^13^C NMR (151 MHz, CDCl_3_) *δ* 170.81, 170.48, 169.62, 169.00, 138.46, 134.14, 127.91, 127.74, 79.33, 76.44, 76.03, 74.30, 72.48, 68.49, 62.33, 60.29, 20.91, 20.78, 20.76, 20.58. HR-MS (ESI): *m*/*z* [M+H]^+^ calcd for C_22_H_35_B_10_O_9_^+^: 553.3206, found: 553.3228.

Synthesis of (2*R*,3*R*,4*R*,5*S*,6*S*)-3,4,5-tris(benzyloxy)-2-((benzyloxy)methyl)-6-(4-(1,2-dicarba-*closo*-dodecaboran-1-yl)phenyl)tetrahydro-2H-pyran (**B1**): Following the general synthetic procedure 5, **B1** was prepared from **43** (0.19 g, 0.345 mmol) using Na (32 mg, 1.38 mmol) in dried MeOH (3.8 mL). Column chromatography purification (MeOH/CH_2_Cl_2_ = 1/10 by *v*/*v*) gave **B1**. White solid, 0.12 g (91%). m.p. 128.8–130.2 °C. [α]_D_^20^ = +8.2° (*c* = 7.4, MeOH). ^1^H NMR (500 MHz, CD_3_OD) *δ* 7.54–7.52 (m, 2H), 7.45–7.43 (m, 2H), 5.11 (s, 1H), 4.15 (d, J = 9.5 Hz, 1H), 3.89–3.86 (m, 1H), 3.72–3.69 (m, 1H), 3.49–3.39 (m, 3H), 3.30–3.26 (m, 1H), 2.89–1.79 (m, 10H). ^13^C NMR (151 MHz, CD_3_OD) *δ* 143.01, 134.62, 129.25, 128.02, 82.61, 82.17, 79.71, 78.04, 76.44, 71.79, 63.05, 61.71. ^11^B NMR (160 MHz, CD_3_OD) *δ* −2.39, −3.31, −4.38, −8.61, −9.54, −10.39, −11.46, −13.19. HR-MS (ESI): *m*/*z* [M-H]^−^ calcd for C_14_H_25_B_10_O_5_^−^: 383.2638, found: 383.2636. HPLC purity, 96.55%.

Synthesis of (2*R*,3*S*,4*R*,5*S*,6*S*)-3,4,5-tris(benzyloxy)-2-((benzyloxy)methyl)-6-(4-(1,2-dicarba-*closo*-dodecaboran-1-yl)phenyl)tetrahydro-2H-pyran (**44**): Following the general synthetic procedure 4, **44** was prepared from *o*-carborane (0.50 g, 3.47 mmol) and **14** (3.02 g, 4.16 mmol) using *n*-BuLi (3.3 mL, 1.6 M in THF, 5.28 mmol), Pd[P(*t*-Bu)_3_]_2_ (1.77 g, 3.47 mmol), PCy_3_ (0.97 g, 3.47 mmol), dried THF (4 mL), and dried *m*-xylene (10 mL). Column chromatography purification (EtOAc/PE = 1/8 by *v*/*v*) gave **44**. Yellow oil, 1.27 g (49%). ^1^H NMR (500 MHz, CDCl_3_) *δ* 7.42–7.30 (m, 17H), 7.25–7.18 (m, 5H), 6.85–6.84 (m, 2H), 5.00 (d, *J* = 11.5 Hz, 1H), 4.80–4.73 (m, 2H), 4.64 (d, *J* = 11.5 Hz, 1H), 4.57 (d, *J* = 10.5 Hz, 1H), 4.48–4.41 (m, 2H), 4.18 (d, *J* = 9.0 Hz, 1H), 4.07–4.06 (m, 1H), 3.94–3.91 (m, 2H), 3.87 (t, *J* = 9.3 Hz, 1H), 3.72–3.68 (m, 2H), 3.64–3.56 (m, 2H), 2.77–1.61 (m, 10H). ^13^C NMR (151 MHz, CDCl_3_) *δ* 141.78, 139.08, 138.46, 137.93, 137.84, 133.24, 128.61, 128.57, 128.44, 128.41, 128.34, 128.32, 128.10, 127.97, 127.88, 127.84, 127.66, 127.35, 84.50, 81.25, 80.20, 77.45, 76.49, 75.40, 74.74, 74.29, 73.69, 72.62, 68.85, 60.20. HR-MS (ESI): *m*/*z* [M+H]^+^ calcd for C_42_H_51_B_10_O_5_^+^: 745.4662, found: 745.4706.

Synthesis of (2*R*,3*S*,4*R*,5*S*,6*S*)-2-(acetoxymethyl)-6-(4-(1,2-dicarba-*closo*-dodecaboran-1-yl)phenyl)tetrahydro-2H-pyran-3,4,5-triyl triacetate (**45**): Following the procedure for the synthesis of **37** from **36**, **45** was prepared from **44** (0.96 g, 1.29 mmol) using Ac_2_O (9.6 mL) and BF_3_·Et_2_O (9.6 mL). Column chromatography purification (EtOAc/PE = 1/3 by *v*/*v*) gave **45**. Yellow foam, 0.37 g (52%). ^1^H NMR (500 MHz, CDCl_3_) *δ* 7.48–7.46 (m, 2H), 7.37–7.35 (m, 2H), 5.52–5.51 (m, 1H), 5.30 (t, *J* = 9.8 Hz, 1H), 5.18–5.16 (m, 1H), 4.39 (d, *J* = 10.0 Hz, 1H), 4.19–4.12 (m, 2H), 4.07–4.05 (m, 1H), 3.94 (s, 1H), 2.76–1.71 (m, 10H), 2.20 (s, 3H), 2.03 (s, 3H), 1.99 (s, 3H), 1.84 (s, 3H). ^13^C NMR (151 MHz, CDCl_3_) *δ* 170.58, 170.34, 170.30, 169.03, 138.77, 134.04, 127.88, 127.81, 79.84, 76.08, 74.92, 72.24, 69.72, 67.85, 61.82, 60.30, 20.85, 20.84, 20.76, 20.65. HR-MS (ESI): *m*/*z* [M+H]^+^ calcd for C_22_H_35_B_10_O_9_^+^: 553.3206, found: 553.3212.

Synthesis of (2*R*,3*R*,4*R*,5*R*,6*S*)-2-(hydroxymethyl)-6-(4-(1,2-dicarba-*closo*-dodecaboran-1-yl)phenyl)tetrahydro-2H-pyran-3,4,5-triol (**B2**): Following the general synthetic procedure 5, **B2** was prepared from **45** (0.45 g, 0.817 mmol) using Na (56 mg, 2.45 mmol) in dried MeOH (9 mL). Column chromatography purification (MeOH/CH_2_Cl_2_ = 1/10 by *v*/*v*) gave **B2**. White solid, 0.28 g (90%). m.p. 124.5–126.1 °C. [α]_D_^20^ = +22.2° (*c* = 12.2, MeOH). ^1^H NMR (500 MHz, DMSO-*d*_6_) *δ* 7.53 (d, *J* = 8.0 Hz, 2H), 7.37 (d, *J* = 8.5 Hz, 2H), 5.78 (s, 1H), 4.72 (t, *J* = 5.3 Hz, 2H), 4.53 (t, *J* = 5.3 Hz, 1H), 4.41 (d, *J* = 5.0 Hz, 1H), 3.98 (d, *J* = 9.5 Hz, 1H), 3.76–3.74 (m, 1H), 3.54–3.46 (m, 4H), 3.41–3.38 (m, 1H), 2.92–1.64 (m, 10H). ^13^C NMR (151 MHz, DMSO-*d*_6_) *δ* 142.71, 131.96, 128.28, 126.64, 81.19, 79.45, 77.09, 74.95, 71.38, 68.89, 61.00, 60.86. ^11^B NMR (160 MHz, CD_3_OD) *δ* −2.35, −3.27, −5.14, −8.57, −9.51, −10.54, −11.44, −13.26. HR-MS (ESI): *m*/*z* [M+H]^+^ calcd for C_14_H_27_B_10_O_5_^+^: 385.2784, found: 385.2785. HPLC purity, 95.05%.

Synthesis of (2*R*,3*R*,4*R*,5*R*,6*S*)-3,4,5-tris(benzyloxy)-2-((benzyloxy)methyl)-6-(4-(1,2-dicarba-*closo*-dodecaboran-1-yl)phenyl)tetrahydro-2H-pyran (**46**): Following the general synthetic procedure 4, **46** was prepared from *o*-carborane (0.20 g, 1.39 mmol) and **20** (1.01 g, 1.39 mmol) using *n*-BuLi (1.3 mL, 1.6 M in THF, 2.08 mmol), Pd[P(*t*-Bu)_3_]_2_ (0.35 g, 0.693 mmol), PCy_3_ (0.19 g, 0.693 mmol), dried THF (1.6 mL), and dried *m*-xylene (4 mL). Column chromatography purification (EtOAc/PE = 1/10 by *v*/*v*) gave **46**. Yellow oil, 0.25 g (24%). ^1^H NMR (500 MHz, CDCl_3_) *δ* 7.38–7.28 (m, 17H), 7.23–7.11 (m, 5H), 6.81 (d, *J* = 7.0 Hz, 2H), 4.92 (d, *J* = 10.5 Hz, 1H), 4.77–4.76 (m, 2H), 4.70–4.68 (m, 1H), 4.64–4.57 (m, 3H), 4.44 (s, 1H), 4.20 (d, *J* = 12.0 Hz, 1H), 4.03 (t, *J* = 9.5 Hz, 1H), 3.95 (s, 1H), 3.91–3.90 (m, 1H), 3.82–3.78 (m, 3H), 3.61–3.58 (m, 1H), 2.96–1.64 (m, 10H). ^13^C NMR (151 MHz, CDCl_3_) *δ* 141.22, 138.51, 138.39, 138.35, 138.02, 132.58, 128.65, 128.53, 128.49, 128.22, 128.14, 128.10, 128.01, 127.92, 127.87, 127.71, 127.68, 127.62, 127.15, 127.12, 85.08, 80.07, 79.20, 76.55, 75.47, 75.05, 74.58, 73.70, 72.69, 69.68, 60.22, 29.18. HR-MS (ESI): *m*/*z* [M-H]^−^ calcd for C_42_H_49_B_10_O_5_^−^: 743.4516, found: 743.4565.

Synthesis of (2*R*,3*R*,4*R*,5*R*,6*S*)-2-(acetoxymethyl)-6-(4-(1,2-dicarba-*closo*-dodecaboran-1-yl)tetrahydro-2H-pyran-3,4,5-triyl triacetate (**47**): Following the procedure for the synthesis of **37** from **36**, **47** was prepared from **46** (0.68 g, 0.915 mmol) using Ac_2_O (6.8 mL) and BF_3_·Et_2_O (6.8 mL). Column chromatography purification (EtOAc/PE = 1/4 by *v*/*v*) gave **47**. Yellow foam, 0.37 g (73%). ^1^H NMR (500 MHz, CDCl_3_) *δ* 7.45 (d, *J* = 8.5 Hz, 2H), 7.30 (d, *J* = 8.0 Hz, 2H), 5.56 (d, *J* = 3.5 Hz, 1H), 5.34–5.30 (m, 1H), 5.24–5.21 (m, 1H), 4.75 (s, 1H), 4.34–4.31 (m, 1H), 4.25–4.22 (m, 1H), 3.94 (s, 1H), 3.83–3.79 (m, 1H), 2.90–1.65 (m, 10H), 2.10 (s, 3H), 2.08 (s, 3H), 1.99 (s, 3H), 1.92 (s, 3H). ^13^C NMR (151 MHz, CDCl_3_) *δ* 170.84, 170.37, 170.04, 169.87, 138.34, 133.39, 127.61, 126.71, 77.58, 76.69, 76.12, 72.47, 70.22, 65.99, 62.98, 60.25, 20.93, 20.87, 20.75, 20.58. HR-MS (ESI): *m*/*z* [M+H]^+^ calcd for C_22_H_35_B_10_O_9_^+^: 553.3206, found: 553.3213.

Synthesis of (2*R*,3*S*,4*R*,5*S*,6*S*)-2-(hydroxymethyl)-6-(4-(1,2-dicarba-*closo*-dodecaboran-1-yl)tetrahydro-2H-pyran-3,4,5-triol (**B3**): Following the general synthetic procedure 5, **B3** was prepared from **47** (0.37 g, 0.672 mmol) using Na (62 mg, 2.69 mmol) in dried MeOH (7.4 mL). Column chromatography purification (MeOH/CH_2_Cl_2_ = 1/10 by *v*/*v*) gave **B3**. White solid, 0.14 g (54%). m.p. 222.1–223.5 °C. [α]_D_^20^ = +12.0° (*c* = 15.2, MeOH). ^1^H NMR (500 MHz, DMSO-*d*_6_) *δ* 7.50 (d, *J* = 8.5 Hz, 2H), 7.40 (d, *J* = 8.0 Hz, 2H), 5.78 (s, 1H), 4.79 (d, *J* = 5.5 Hz, 1H), 4.73 (d, *J* = 5.5 Hz, 1H), 4.48 (s, 1H), 4.41 (t, *J* = 5.8 Hz, 1H), 4.27 (d, *J* = 5.5 Hz, 1H), 3.75–3.72 (m, 2H), 3.52–3.46 (m, 2H), 3.42–3.38 (m, 1H), 3.19–3.15 (m, 1H), 2.90–1.71 (m, 10H). ^13^C NMR (151 MHz, DMSO-*d*_6_) *δ* 142.30, 131.30, 127.26, 126.32, 81.48, 78.37, 77.18, 74.96, 71.83, 67.04, 61.68, 60.98. ^11^B NMR (160 MHz, CD_3_OD) *δ* −2.39, −3.31, −5.32, −8.63, −9.57, −10.39, −11.45, −12.44, −13.38. HR-MS (ESI): *m*/*z* [M-H]^−^ calcd for C_14_H_25_B_10_O_5_^−^: 383.2638, found: 383.2631. HPLC purity, 98.50%.

Synthesis of (2*R*,3*R*,4*S*,5*S*,6*S*)-3,4-bis(benzyloxy)-2-((benzyloxy)methyl)-5-fluoro-6-(4-(1,2-dicarba-*closo*-dodecaboran-1-yl)tetrahydro-2H-pyran (**48**): Following the general synthetic procedure 4, **48** was prepared from *o*-carborane (0.20 g, 1.39 mmol) and **26** (0.80 g, 1.25 mmol) using *n*-BuLi (1.3 mL, 1.6 M in THF, 2.08 mmol), Pd[P(*t*-Bu)_3_]_2_ (0.35 g, 0.693 mmol), PCy_3_ (0.19 g, 0.693 mmol), dried THF (1.6 mL), and dried *m*-xylene (4 mL). Column chromatography purification (EtOAc/PE = 1/12 by *v*/*v*) gave **48**. Yellow oil, 0.21 g (23%). ^1^H NMR (500 MHz, CDCl_3_) *δ* 7.50–7.49 (m, 2H), 7.41–7.30 (m, 15H), 7.22–7.20 (m, 2H), 4.93–4.89 (m, 2H), 4.81–4.78 (m, 1H), 4.61 (d, *J* = 12.0 Hz, 2H), 4.56–4.53 (m, 1H), 4.46–4.37 (m, 1H), 4.35–4.34 (m, 1H), 3.96 (s, 1H), 3.93–3.89 (m, 1H), 3.78–3.75 (m, 3H), 3.65–3.61 (m, 1H), 2.89–1.77 (m, 10H). ^13^C NMR (151 MHz, CDCl_3_) *δ* 139.85, 138.20, 138.15, 138.06, 133.63, 128.58, 128.56, 128.54, 128.14, 128.02, 127.98, 127.85, 127.76, 127.57, 95.34, 94.30, 84.59 (d, *J* = 16.3 Hz), 79.46, 78.44 (d, *J* = 23.1 Hz), 76.27, 75.42, 75.09 (d, *J* = 2.9 Hz), 73.64, 68.95, 60.23. HR-MS (ESI): *m*/*z* [M-H]^−^ calcd for C_35_H_42_B_10_FO_4_^−^: 655.4003, found: 655.4020.

Synthesis of (2*R*,3*R*,4*S*,5*S*,6*S*)-2-(acetoxymethyl)-5-fluoro-6-(4-(1,2-dicarba-*closo*-dodecaboran-1-yl)tetrahydro-2H-pyran-3,4-diyl diacetate (**49**): Following the procedure for the synthesis of **37** from **36**, **49** was prepared from **48** (0.50 g, 0.764 mmol) using Ac_2_O (5 mL) and BF_3_·Et_2_O (5 mL). Column chromatography purification (EtOAc/PE = 1/4 by *v*/*v*) gave **49**. White solid, 0.25 g (64%). m.p. 202.8–204.4 °C. ^1^H NMR (500 MHz, CDCl_3_) *δ* 7.51 (d, *J* = 8.0 Hz, 2H), 7.37 (d, *J* = 8.5 Hz, 2H), 5.48–5.42 (m, 1H), 5.14 (t, *J* = 9.8 Hz, 1H), 4.49–4.46 (m, 1H), 4.42–4.30 (m, 1H), 4.29–4.27 (m, 1H), 4.18–4.16 (m, 1H), 3.96 (s, 1H), 3.87–3.84 (m, 1H), 2.98–1.69 (m, 10H), 2.08 (s, 6H), 2.07 (s, 3H). ^13^C NMR (151 MHz, CDCl_3_) *δ* 170.74, 170.23, 169.76, 138.50, 134.10, 127.91, 127.38, 91.50, 90.24, 78.36 (d, *J* = 22.5 Hz), 76.30, 74.05 (d, *J* = 19.8 Hz), 68.32 (d, *J* = 7.6 Hz), 62.24, 60.16, 20.87, 20.81, 20.72. HR-MS (ESI): *m*/*z* [M-H]^−^ calcd for C_20_H_30_B_10_FO_7_^−^: 511.2912, found: 511.2914.

Synthesis of (2*R*,3*S*,4*S*,5*R*,6*S*)-5-fluoro-2-(hydroxymethyl)-6-(4-(1,2-dicarba-*closo*-dodecaboran-1-yl)tetrahydro-2H-pyran-3,4-diol (**B4**): Following the general synthetic procedure 5, **B4** was prepared from **47** (0.20 g, 0.392 mmol) using Na (54 mg, 2.35 mmol) in dried MeOH (4 mL). White solid, 75 mg (50%). m.p. 220.3–221.2 °C. [α]_D_^20^ = +18.6° (*c* = 6.2, MeOH). ^1^H NMR (500 MHz, DMSO-*d*_6_) *δ* 7.58 (d, *J* = 8.0 Hz, 2H), 7.44 (d, *J* = 8.0 Hz, 2H), 5.80 (s, 1H), 5.52 (d, *J* = 5.0 Hz, 1H), 5.27 (d, *J* = 5.5 Hz, 1H), 4.54 (s, 1H), 4.42–4.39 (m, 1H), 4.20–4.06 (m, 1H), 3.69 (d, *J* = 11.5 Hz, 1H), 3.61–3.54 (m, 1H), 3.47–3.44 (m, 1H), 3.34–3.31 (m, 1H), 3.27–3.23 (m, 1H), 2.99–1.66 (m, 10H). ^13^C NMR (151 MHz, DMSO-*d*_6_) *δ* 140.33, 132.86, 128.04, 127.18, 94.33, 93.12, 81.30, 77.25 (d, *J* = 23.4 Hz), 76.79, 75.60 (d, *J* = 16.5 Hz), 70.07 (d, *J* = 8.3 Hz, 2H), 61.04. ^11^B NMR (160 MHz, DMSO-*d*_6_) *δ* −2.80, −3.61, −8.72, −9.66, −11.33. ^19^F NMR (471 MHz, DMSO-*d*_6_) *δ* −193.99. HR-MS (ESI): *m*/*z* [M-H]^−^ calcd for C_14_H_24_B_10_FO_4_^−^: 385.2595, found: 385.2587. HPLC purity, 95.49%.

Synthesis of 1,2-[4-bromo-1,2-phenylenebis(methylene)]-1,2-dicarba-*closo*-dodecaborane (**50**): To a stirred solution of *o*-carborane (**27**, 4.00 g, 27.7 mmol) in a mixture of dried toluene (32 mL) and THF (16 mL) cooled at 0 °C under N_2_ was added dropwise a solution of *n*-BuLi in THF (43 mL, 1.6 M in THF, 68.8 mmol), and, after addition, the reaction mixture was stirred at room temperature for 1 h and then cooled back to 0 °C again, followed by dropwise addition of a solution of **4** (14.26 g, 41.6 mmol) in dried toluene (15 mL). After that, the reaction mixture was stirred at 100 °C overnight, when TLC analysis indicated completion of the reaction. Upon cooling to room temperature, the reaction mixture was poured into ice water (100 mL), and the resulting mixture was extracted with EtOAc (70 mL × 3). The combined extracts were washed with brine, dried (MgSO_4_), and evaporated on a rotary evaporator to afford a residue, which was purified through column chromatography (PE) to give **50**. White solid, 4.50 g (50%). m.p. 140.6–142.3 °C. ^1^H NMR (500 MHz, CDCl_3_) *δ* 7.37–7.35 (m, 1H), 7.23 (s, 1H), 6.94 (d, *J* = 8.5 Hz, 1H), 3.67 (s, 2H), 3.65 (s, 2H), 2.85–1.79 (m, 10H). ^13^C NMR (151 MHz, CDCl_3_) *δ* 131.59, 131.42, 130.90, 130.36, 128.33, 121.35, 70.90, 70.59, 37.34, 37.28. HR-MS (ESI): *m*/*z* [M+Na]^+^ calcd for C_10_H_17_B_10_BrNa^+^: 349.1336, found: 349.1360.

Synthesis of (3*R*,4*S*,5*S*,6*R*)-6-(hydroxymethyl)-2-(1,2-(1,2-dicarba-*closo*-dodecaboranylene)bis(methylene)benzen-4-yl)tetrahydro-2H-pyran-2,3,4,5-tetraol (**51**): To a stirred solution of **50** (1.30 g, 4.00 mmol) in dried THF (13 mL) cooled at −78 °C under N_2_ was added dropwise a solution of *n*-BuLi in THF (3 mL, 1.6 M in THF, 4.8 mmol), and, after addition, stirring was continued at this temperature for 1 h, followed by dropwise addition of a solution of **2** (2.80 g, 6.00 mmol) in dried THF (3 mL). After that, the reaction mixture was stirred at this temperature for 1 h and quenched through dropwise addition of saturated aqueous NH_4_Cl (10 mL). The resulting mixture was extracted with EtOAc (20 mL × 3), and the combined extracts were washed with brine, dried (MgSO_4_), and evaporated on a rotary evaporator to afford a residue. The residue was dissolved in MeOH (10 mL) and strongly acidic cation exchange resin (Amberlite 732) was added to adjust the pH = 5–6. The reaction mixture was stirred for an additional 2 h, adjusted to pH = 7–8 through the addition of Et_3_N and evaporated on a rotary evaporator to give crude **51**. Yellow oil, 1.00 g. This sample was used directly in the next step without further purification or characterization.

Synthesis of (2*R*,3*S*,4*R*,5*R*,6*S*)-2-(hydroxymethyl)-6-(1,2-(1,2-dicarba-*closo*-dodecaboranylene)bis(methylene)benzen-4-yl)tetrahydro-2H-pyran-3,4,5-triol (**52**): To a stirred solution of crude **51** prepared above (1.00 g, deemed to be 2.45 mmol) in dried CH_2_Cl_2_ (10 mL) cooled at −35 °C under N_2_ was added Et_3_SiH (0.57 g, 4.90 mmol) in one portion, followed by dropwise addition of BF_3_·Et_2_O (0.52 g, 3.67 mmol). After that, the reaction mixture was stirred at room temperature overnight, when TLC analysis indicated completion of the reaction. Et_3_N was added dropwise to the reaction mixture to adjust the pH = 7–8, and the resulting mixture was stirred for another 10 min and then evaporated on a rotary evaporator to dryness to give crude **52**. Yellow oil, 1.06 g. This sample was used directly in the next step without further purification or characterization.

Synthesis of (2*R*,3*R*,4*R*,5*S*,6*S*)-2-(acetoxymethyl)-6-(1,2-(1,2-dicarba-*closo*-dodecaboranylene)bis(methylene)benzen-4-yl)tetrahydro-2H-pyran-3,4,5-triyl triacetate (**53**): Following the procedure for the synthesis of **31** from **30**, **53** was prepared from crude **52** prepared above (1.06 g, deemed to be 2.59 mmol) using DMAP (0.48 g, 3.89 mmol) and Ac_2_O (5.3 mL) in pyridine (10.6 mL). Column chromatography purification (EtOAc/PE = 1/4 by *v*/*v*) gave **53**. White solid, 0.15 g (7% overall for **50** to **53**). m.p. 226.2–227.8 °C. ^1^H NMR (500 MHz, CDCl_3_) *δ* 7.21–7.19 (m, 1H), 7.06–7.03 (m, 2H), 5.32 (t, *J* = 9.5 Hz, 1H), 5.22 (t, *J* = 9.8 Hz, 1H), 5.08 (t, *J* = 9.8 Hz, 1H), 4.36 (d, *J* = 10.0 Hz, 1H), 4.31–4.28 (m, 1H), 4.17–4.14 (m, 1H), 3.85–3.81 (m, 1H), 3.70–3.65 (m, 4H), 2.89–1.69 (m, 10H), 2.09 (s, 3H), 2.06 (s, 3H), 2.00 (s, 3H), 1.82 (s, 3H). ^13^C NMR (126 MHz, CDCl_3_) *δ* 170.84, 170.49, 169.64, 168.93, 136.09, 130.07, 129.65, 128.94, 127.51, 126.55, 79.71, 76.41, 74.24, 72.65, 71.13, 71.09, 68.59, 62.41, 37.73, 37.55, 20.94, 20.79, 20.77, 20.57. HR-MS (ESI): *m*/*z* [M+Na]^+^ calcd for C_24_H_36_B_10_NaO_9_^+^: 601.3182, found: 601.3187.

Synthesis of (2*R*,3*S*,4*R*,5*R*,6*S*)-2-(hydroxymethyl)-6-(1,2-(1,2-dicarba-*closo*-dodecaboranylene)bis(methylene)benzen-4-yl)tetrahydro-2H-pyran-3,4,5-triol (**C1**): Following the general synthetic procedure 5, **C1** was prepared from **53** (60 mg, 0.112 mmol) using Na (31 mg, 1.35 mmol) in MeOH (0.8 mL). Column chromatography purification (MeOH/CH_2_Cl_2_ = 1/10 by *v*/*v*) gave **C1**. White solid, 24 mg (52%). m.p. 119.6–121.4 °C. [α]_D_^20^ = +0.5° (*c* = 10.0, MeOH). ^1^H NMR (600 MHz, DMSO-*d*_6_) *δ* 7.22–7.11 (m, 3H), 4.96–4.79 (m, 3H), 4.44 (s, 1H), 3.98 (d, *J* = 7.5 Hz, 1H), 3.82 (s, 4H), 3.69 (d, *J* = 10.0 Hz, 1H), 3.28–3.12 (m, 5H), 2.77–1.56 (m, 10H). ^13^C NMR (151 MHz, DMSO-*d*_6_) *δ* 139.65, 128.47, 128.22, 127.98, 127.79, 127.09, 81.25, 80.95, 78.43, 74.61, 72.73, 70.36, 61.40, 36.48, 36.24. ^11^B NMR (160 MHz, CD_3_OD) *δ* −5.10, −6.01, −9.64, −10.35. HR-MS (ESI): *m*/*z* [M+H]^+^ calcd for C_16_H_27_B_10_O_5_^+^: 409.2784, found: 409.2763. HPLC purity, 94.68%.

Synthesis of (3*R*,4*S*,5*S*,6*R*)-3,4,5-tris(benzyloxy)-6-((benzyloxy)methyl)-2-methoxy-2-(1,2-(1,2-dicarba-*closo*-dodecaboranylene)bis(methylene)benzen-4-yl)tetrahydro-2H-pyran (**54**): Following the general synthetic procedure 2, **54** was prepared from **50** (0.37 g, 1.14 mmol) and **12** (0.74 g, 1.37 mmol) using *n*-BuLi (0.85 mL, 1.6 M in THF, 1.36 mmol), MeSO_3_H (0.55 g, 5.69 mmol), dried MeOH (2.8 mL), and dried THF (3.7 mL) as a crude sample. Yellow oil, 0.96 g. This sample was used directly in the next step without further purification or characterization.

Synthesis of (2*R*,3*S*,4*R*,5*S*,6*S*)-3,4,5-tris(benzyloxy)-2-((benzyloxy)methyl)-6-(1,2-(1,2-dicarba-*closo*-dodecaboranylene)bis(methylene)benzen-4-yl)tetrahydro-2H-pyran (**55**): Following the general synthetic procedure 3, **55** was prepared from crude **54** prepared above (0.96 g, deemed to be 1.20 mmol) using Et_3_SiH (0.28 g, 2.40 mmol) and BF_3_·Et_2_O (0.26 g, 1.80 mmol) in dried CH_2_Cl_2_ (9.6 mL) as a crude sample. Yellow oil, 0.20 g. This sample was used directly in the next step without further purification or characterization.

Synthesis of (2*R*,3*S*,4*R*,5*S*,6*S*)-2-(acetoxymethyl)-6-(1,2-(1,2-dicarba-*closo*-dodecaboranylene)bis(methylene)benzen-4-yl)tetrahydro-2H-pyran-3,4,5-triyl triacetate (**56**): Following the procedure for the synthesis of **37** from **36**, **56** was prepared from crude **55** prepared above (0.20 g, deemed to be 0.764 mmol) using Ac_2_O (2 mL) and BF_3_·Et_2_O (2 mL). Column chromatography purification (EtOAc/PE = 1/4 by *v*/*v*) gave **56**. White foam, 0.10 g (15% overall **50** to **56**). ^1^H NMR (500 MHz, CDCl_3_) *δ* 7.23 (d, *J* = 7.5 Hz, 1H), 7.10 (s, 1H), 7.04 (d, *J* = 8.0 Hz, 1H), 5.52 (d, *J* = 3.5 Hz, 1H), 5.28 (t, *J* = 10.0 Hz, 1H), 5.18–5.15 (m, 1H), 4.32 (d, *J* = 9.5 Hz, 1H), 4.18–4.14 (m, 2H), 4.07–4.04 (m, 1H), 3.72–3.67 (m, 4H), 2.80–1.67 (m, 10H), 2.21 (s, 3H), 2.04 (s, 3H), 1.99 (s, 3H), 1.82 (s, 3H). ^13^C NMR (151 MHz, CDCl_3_) *δ* 170.60, 170.38, 170.33, 169.02, 136.38, 129.98, 129.60, 128.83, 127.63, 126.76, 80.26, 74.88, 72.18, 71.16, 71.13, 69.94, 67.86, 61.82, 37.74, 37.55, 20.89, 20.86, 20.77, 20.67. HR-MS (ESI): *m*/*z* [M+H]^+^ calcd for C_24_H_37_B_10_O_9_^+^: 579.3363, found: 579.3380.

Synthesis of (2*R*,3*R*,4*R*,5*R*,6*S*)-2-(hydroxymethyl)-6-(1,2-(1,2-dicarba-*closo*-dodecaboranylene)bis(methylene)benzen-4-yl)tetrahydro-2H-pyran-3,4,5-triol (**C2**): Following the general synthetic procedure 5, **C2** was prepared from **56** (74 mg, 0.128 mmol) using Na (21 mg, 0.898 mmol) in dried MeOH (1.5 mL). Column chromatography purification (MeOH/CH_2_Cl_2_ = 1/10 by *v*/*v*) gave **C2**. White solid, 42 mg (52%). m.p. 118.3–119.2 °C. [α]_D_^20^ = +4.1° (*c* = 2.2, MeOH). ^1^H NMR (500 MHz, DMSO-*d*_6_) *δ* 7.23–7.22 (m, 1H), 7.15 (s, 1H), 7.13–7.11 (m, 1H), 4.69 (d, *J* = 5.5 Hz, 1H), 4.59 (d, *J* = 5.5 Hz, 1H), 4.54–4.52 (m, 1H), 4.38 (d, *J* = 5.0 Hz, 1H), 3.91 (d, *J* = 9.0 Hz, 1H), 3.82 (d, *J* = 5.0 Hz, 4H), 3.76–3.74 (m, 1H), 3.53–3.45 (m, 4H), 3.39–3.37 (m, 1H), 2.88–1.57 (m, 10H). ^13^C NMR (151 MHz, DMSO-*d*_6_) *δ* 139.94, 128.35, 128.08, 127.90, 127.85, 127.12, 81.63, 79.43, 75.02, 72.68, 71.35, 68.85, 60.86, 54.91, 36.42, 36.19. ^11^B NMR (160 MHz, CD_3_OD) *δ* −5.16, −6.09, −9.54, −9.76, −10.46. HR-MS (ESI): *m*/*z* [M-H]^−^ calcd for C_16_H_27_B_10_O_5_^−^: 409.2795, found: 409.2800. HPLC purity, 95.34%.

Synthesis of 2-(2-(1,2-dicarba-*closo*-dodecaboran-1-yl)ethoxy)ethyl 4-methylbenzenesulfonate (**57-1**): To a stirred solution of *o*-carborane (**27**, 2.50 g, 17.3 mmol) in dried THF (25 mL) cooled at 0 °C was added a solution of *n*-BuLi in THF (13 mL, 1.6 M in THF, 20.8 mmol), and, after addition, the reaction mixture was stirred at room temperature for 1 h and then cooled back to 0 °C, followed by dropwise addition of a solution of TsO-(CH_2_CH_2_O)_2_-Ts (8.62 g, 20.8 mmol) in dried THF (25 mL). After that, the reaction mixture was stirred at room temperature overnight, when TLC analysis indicated completion of the reaction, and then poured into ice water (50 mL). The resulting mixture was extracted with EtOAc (50 mL × 3), and the combined extracts were washed with brine, dried (MgSO_4_), and evaporated on a rotary evaporator to afford a residue, which was purified through column chromatography (EtOAc/PE = 1/3 by *v*/*v*) to give **57-1**. Yellow oil, 1.03 g (15%). ^1^H NMR (500 MHz, CDCl_3_) *δ* 7.79 (d, *J* = 8.0 Hz, 2H), 7.37 (d, *J* = 8.0 Hz, 2H), 4.15–4.13 (m, 2H), 3.85 (s, 1H), 3.61–3.59 (m, 2H), 3.51 (t, *J* = 5.8 Hz, 2H), 2.75–1.68 (m, 10H), 2.47 (s, 3H), 2.46–2.45 (m, 2H). The ^1^H NMR data were consistent with those reported [50].

Synthesis of 2-(2-(2-(1,2-dicarba-*closo*-dodecaboran-1-yl)ethoxy)ethoxy)ethyl 4-methylbenzenesulfonate (**57-2**): Following the procedure for the synthesis of **57-1** from **27**, **57-2** was prepared from *o*-carborane (**27**, 3.0 g, 20.8 mmol) and TsO-(CH_2_CH_2_O)_3_-Ts (11.45 g, 25.0 mmol) using *n*-BuLi (15.6 mL, 1.6 M in THF, 24.96 mmol) in dried THF (60 mL). Column chromatography purification (EtOAc/PE = 1/5 by *v*/*v*) gave **57-2**. Yellow oil, 1.94 g (22%). ^1^H NMR (500 MHz, CDCl_3_) *δ* 7.80 (d, *J* = 8.0 Hz, 2H), 7.36 (d, *J* = 8.0 Hz, 2H), 4.14 (t, *J* = 4.8 Hz, 2H), 4.08 (s, 1H), 3.68 (t, *J* = 5.0 Hz, 2H), 3.57–3.53 (m, 4H), 3.51–3.49 (m, 2H), 2.85–1.58 (m, 10H), 2.50 (t, *J* = 5.8 Hz, 2H), 2.46 (s, 3H). The ^1^H NMR data were consistent with those reported [50].

Synthesis of 1-(2-(2-iodoethoxy)ethyl)-1,2-dicarba-*closo*-dodecaborane (**58-1**): A mixture of **57-1** (1.03 g, 2.66 mmol), NaI (1.60 g, 10.7 mmol), and TBAI (0.30 g, 0.799 mmol) in dried toluene (10.3 mL) was stirred at 100 °C under N_2_ overnight, when TLC analysis indicated completion of the reaction. Upon cooling to room temperature, the reaction mixture was poured into ice water (30 mL), and the resulting mixture was extracted with EtOAc (30 mL × 3). The combined extracts were washed with brine, dried (MgSO_4_), and evaporated on a rotary evaporator to afford a residue, which was purified through column chromatography (EtOAc/PE = 1/5 by *v*/*v*) to give **58-1**. Yellow oil, 0.61 g (62%). ^1^H NMR (500 MHz, CDCl_3_) *δ* 4.11 (s, 1H), 3.67 (t, *J* = 6.0 Hz, 2H), 3.58 (t, *J* = 5.5 Hz, 2H), 3.24 (t, *J* = 6.0 Hz, 2H), 2.89–1.71 (m, 10H), 2.55 (t, *J* = 5.8 Hz, 2H). ^13^C NMR (151 MHz, CDCl_3_) *δ* 73.18, 71.44, 68.34, 60.63, 37.72, 2.73. HR-MS (ESI): *m*/*z* [M-H]^−^ calcd for C_6_H_18_B_10_IO^−^: 343.1338, found: 343.1328.

Synthesis of 1-(2-(2-(2-iodoethoxy)ethoxy)ethyl)-1,2-dicarba-*closo*-dodecaborane (**58-2**): Following the procedure for the synthesis of **58-1** from **57-1**, **58-2** was prepared from **57-2** (0.50 g, 1.16 mmol) using NaI (0.70 g, 4.65 mmol) and TBAI (0.13 g, 0.348 mmol) in dried toluene (5 mL). Column chromatography purification (EtOAc/PE = 1/9 by *v*/*v*) gave **58-2**. Yellow oil, 0.38 g (89%). ^1^H NMR (500 MHz, CDCl_3_) *δ* 4.16 (s, 1H), 3.73 (t, *J* = 6.8 Hz, 2H), 3.62–3.55 (m, 6H), 3.25 (t, *J* = 6.8 Hz, 2H), 2.81–1.71 (m, 10H), 2.53 (t, *J* = 5.5 Hz, 2H). ^13^C NMR (151 MHz, CDCl_3_) *δ* 73.45, 71.99, 70.14, 69.96, 68.75, 60.37, 37.52, 2.70. HR-MS (ESI): *m*/*z* [M+H]^+^ calcd for C_8_H_24_B_10_IO_2_^+^: 389.1746, found: 389.1738.

Synthesis of 2-(2-(1,2-dicarba-*closo*-dodecaboran-1-yl)ethoxy)ethan-1-ol (**59-1**): A mixture of **58-1** (0.61 g, 1.78 mmol) and AcONa (0.58 g, 7.13 mmol) in dried DMF (6.1 mL) was stirred at 80 °C under N_2_ overnight, when TLC analysis indicated completion of the reaction. Upon cooling to room temperature, the reaction mixture was poured into ice water (10 mL), and the resulting mixture was extracted with EtOAc (20 mL × 3). The combined extracts were washed with brine, dried (MgSO_4_), and evaporated on a rotary evaporator to afford a residue, which was dissolved in MeOH (6.1 mL), followed by the addition of NaOH (0.14 g, 3.56 mmol). The resulting mixture was stirred at room temperature under N_2_ for 1 h, when TLC analysis indicated completion of the reaction, and poured into ice water (10 mL). The mixture thus obtained was extracted with EtOAc (20 mL × 3), and the combined extracts were washed with brine, dried (MgSO_4_), and evaporated on a rotary evaporator to give a residue, which was purified through column chromatography (EtOAc/PE = 1/3 by *v*/*v*) to give **59-1**. Yellow oil, 0.23 g (56%). ^1^H NMR (500 MHz, DMSO-*d*_6_) *δ* 5.12 (s, 1H), 4.62 (t, *J* = 5.5 Hz, 1H), 3.50–3.46 (m, 4H), 3.38 (t, *J* = 5.0 Hz, 2H), 2.86–1.64 (m, 10H), 2.53–2.52 (m, 2H). ^13^C NMR (151 MHz, DMSO-*d*_6_) *δ* 74.29, 71.92, 67.97, 62.47, 60.01, 36.35. HR-MS (ESI): *m*/*z* [M-H]^−^ calcd for C_6_H_19_B_10_O_2_^−^: 233.2321, found: 233.2318.

Synthesis of 2-(2-(2-(1,2-dicarba-*closo*-dodecaboran-1-yl)ethoxy)ethoxy)ethan-1-ol (**59-2**): Following the procedure for the synthesis of **59-1** from **58-1**, **59-2** was prepared from **58-2** (0.36 g, 0.932 mmol) using AcONa (0.31 g, 3.73 mmol), NaOH (75 mg, 1.86 mmol) dried DMF (3.6 mL) and MeOH (3.6 mL). Column chromatography purification (EtOAc/PE = 1/3 by *v*/*v*) gave **59-2**. Yellow oil, 0.13 g (50%). ^1^H NMR (500 MHz, DMSO-*d*_6_) *δ* 5.07 (s, 1H), 4.57 (t, *J* = 5.5 Hz, 1H), 3.50–3.46 (m, 8H), 3.41 (t, *J* = 5.0 Hz, 2H), 2.83–1.59 (m, 10H), 2.53–2.52k (m, 2H). ^13^C NMR (151 MHz, DMSO-*d*_6_) *δ* 74.27, 72.28, 69.51, 69.39, 67.95, 62.44, 60.20, 36.31. HR-MS (ESI): *m*/*z* [M+H]^+^ calcd for C_8_H_25_B_10_O_3_^+^: 279.2729, found: 279.2729.

Synthesis of (2*R*,3*R*,4*S*,5*R*,6*R*)-2-(acetoxymethyl)-6-(2-(2-(1,2-dicarba-*closo*-dodecaboran-1-yl)ethoxy)ethoxy)tetrahydro-2H-pyran-3,4,5-triyl triacetate (**60-1**): Following the general synthetic procedure 6, **60-1** was prepared from **59-1** (0.23 g, 0.990 mmol) using β-D-glucose pentaacetate (0.77 g, 1.98 mmol) and BF_3_·Et_2_O (0.56 g, 3.96 mmol) in dried CH_2_Cl_2_ (2.3 mL). Column chromatography purification (EtOAc/PE = 1/3 by *v*/*v*) gave **60-1**. White solid, 0.23 g (41%). m.p. 130.6–131.3 °C. ^1^H NMR (500 MHz, CDCl_3_) *δ* 5.21 (t, *J* = 9.5 Hz, 1H), 5.08 (t, *J* = 9.8 Hz, 1H), 4.99–4.95 (m, 1H), 4.55 (d, *J* = 8.0 Hz, 1H), 4.29–4.25 (m, 1H), 4.17–4.14 (m, 1H), 4.05 (s, 1H), 3.93–3.89 (m, 1H), 3.72–3.65 (m, 2H), 3.57–3.54 (m, 4H), 2.86–1.60 (m, 10H), 2.50 (t, *J* = 5.8 Hz, 2H), 2.10 (s, 3H), 2.05 (s, 3H), 2.03 (s, 3H), 2.02 (s, 3H). ^13^C NMR (151 MHz, CDCl_3_) *δ* 170.80, 170.42, 169.53, 169.39, 100.85, 73.30, 72.85, 72.12, 71.32, 69.95, 68.87, 68.75, 68.48, 62.03, 60.40, 37.62, 20.90, 20.85, 20.76, 20.74. HR-MS (ESI): *m*/*z* [M+Na]^+^ calcd for C_20_H_38_B_10_NaO_11_^+^: 587.3237, found: 587.3257.

Synthesis of (2*R*,3*R*,4*S*,5*R*,6*R*)-2-(acetoxymethyl)-6-(2-(2-(2-(1,2-dicarba-*closo*-dodecaboran-1-yl)ethoxy)ethoxy)ethoxy)tetrahydro-2H-pyran-3,4,5-triyl triacetate (**60-2**). Following the general synthetic procedure 6, **60-2** was prepared from **59-2** (0.42 g, 1.52 mmol) using β-D-glucose pentaacetate (1.19 g, 3.04 mmol) and BF_3_·Et_2_O (0.86 g, 6.08 mmol) in dried CH_2_Cl_2_ (4.2 mL). White solid, 0.29 g (31%). m.p. 93.7–94.8 °C. ^1^H NMR (500 MHz, CDCl_3_) *δ* 5.21 (t, *J* = 9.5 Hz, 1H), 5.09 (t, *J* = 9.8 Hz, 1H), 5.01–4.98 (m, 1H), 4.58 (d, *J* = 8.0 Hz, 1H), 4.28–4.25 (m, 1H), 4.18 (s, 1H), 4.16–4.13 (m, 1H), 3.97–3.93 (m, 1H), 3.72–3.68 (m, 2H), 3.63–3.61 (m, 2H), 3.59–3.55 (m, 4H), 3.54–3.51 (m, 2H), 2.86–1.65 (m, 10H), 2.53 (t, *J* = 5.8 Hz, 2H), 2.09 (s, 3H), 2.04 (s, 3H), 2.03 (s, 3H), 2.01 (s, 3H). ^13^C NMR (151 MHz, CDCl_3_) *δ* 170.82, 170.43, 169.58, 169.47, 101.00, 73.46, 72.94, 72.01, 71.40, 70.48, 70.36, 70.21, 69.24, 68.65, 68.53, 62.08, 60.43, 37.47, 20.89, 20.83, 20.77, 20.75. HR-MS (ESI): *m*/*z* [M-H]^−^ calcd for C_22_H_41_B_10_O_12_^−^: 607.3534, found: 607.3549.

Synthesis of (2*R*,3*S*,4*S*,5*R*,6*R*)-2-(acetoxymethyl)-6-(2-(2-(1,2-dicarba-*closo*-dodecaboran-1-yl)ethoxy)ethoxy)tetrahydro-2H-pyran-3,4,5-triyl triacetate (**61-1**): Following the general synthetic procedure 6, **61-1** was prepared from **59-1** (0.20 g, 0.861 mmol) using β-D-galactose pentaacetate (0.67 g, 1.72 mmol) and BF_3_·Et_2_O (0.49 g, 3.44 mmol) in dried CH_2_Cl_2_ (2 mL). Column chromatography purification (EtOAc/PE = 1/3 by *v*/*v*) gave **61-1**. Yellow oil, 0.16 g (33%). ^1^H NMR (500 MHz, CDCl_3_) *δ* 5.40–5.39 (m, 1H), 5.21–5.17 (m, 1H), 5.04–5.01 (m, 1H), 4.50 (d, *J* = 8.0 Hz, 1H), 4.22–4.18 (m, 1H), 4.14–4.11 (m, 2H), 3.94–3.89 (m, 2H), 3.69–3.65 (m, 1H), 3.60–3.52 (m, 4H), 2.85–1.63 (m, 10H), 2.51 (t, *J* = 5.8 Hz, 2H), 2.17 (s, 3H), 2.06 (s, 6H), 1.99 (s, 3H). ^13^C NMR (151 MHz, CDCl_3_) *δ* 170.55, 170.45, 170.31, 169.49, 101.40, 73.38, 70.96, 69.96, 68.84, 68.83, 68.82, 67.08, 61.36, 60.45, 37.60, 29.84, 20.94, 20.84, 20.81, 20.73. HR-MS (ESI): *m*/*z* [M-H]^−^ calcd for C_20_H_37_B_10_O_11_^−^: 563.3272, found: 563.3288.

Synthesis of (2*R*,3*S*,4*S*,5*R*,6*R*)-2-(acetoxymethyl)-6-(2-(2-(2-(1,2-dicarba-*closo*-dodecaboran-1-yl)ethoxy)ethoxy)ethoxy)tetrahydro-2H-pyran-3,4,5-triyl triacetate (**61-2**): Following the general synthetic procedure 6, **61-2** was prepared from **59-2** (0.40 g, 1.45 mmol) using β-D-galactose pentaacetate (1.13 g, 2.89 mmol) and BF_3_·Et_2_O (0.82 g, 5.79 mmol) in dried CH_2_Cl_2_ (4 mL). Column chromatography purification (EtOAc) gave **61-2**. Colorless oil, 0.25 g (28%). ^1^H NMR (500 MHz, CDCl_3_) *δ* 5.40–5.39 (m, 1H), 5.22–5.19 (m, 1H), 5.03–5.00 (m, 1H), 4.53 (d, *J* = 8.0 Hz, 1H), 4.20–4.18 (m, 1H), 4.16–4.10 (m, 2H), 3.98–3.94 (m, 1H), 3.93–3.90 (m, 1H), 3.73–3.68 (m, 1H), 3.65–3.62 (m, 2H), 3.59–3.55 (m, 4H), 3.54–3.52 (m, 2H), 2.81–1.62 (m, 10H), 2.53 (t, *J* = 5.5 Hz, 2H), 2.16 (s, 3H), 2.06 (s, 3H), 2.05 (s, 3H), 1.99 (s, 3H). ^13^C NMR (201 MHz, CDCl_3_) *δ* 170.52, 170.36, 170.27, 169.54, 101.46, 70.99, 70.82, 70.43, 70.30, 70.17, 69.17, 68.87, 68.59, 67.13, 61.37, 60.40, 45.77, 37.42, 20.89, 20.78, 20.77, 20.69. HR-MS (ESI): *m*/*z* [M-H]^−^ calcd for C_22_H_41_B_10_O_12_^−^: 607.3534, found: 607.3525.

Synthesis of (2*R*,3*R*,4*S*,5*S*,6*R*)-2-(acetoxymethyl)-6-(2-(2-(1,2-dicarba-*closo*-dodecaboran-1-yl)ethoxy)ethoxy)tetrahydro-2H-pyran-3,4,5-triyl triacetate (**62-1**): Following the general synthetic procedure 6, **62-1** was prepared from **59-1** (0.40 g, 1.72 mmol) using α-D-mannose pentaacetate (1.34 g, 3.44 mmol) and BF_3_·Et_2_O (0.98 g, 6.89 mmol) in dried CH_2_Cl_2_ (4 mL). Column chromatography purification (EtOAc/PE = 1/4 by *v*/*v*) gave **62-1**. Colorless oil, 0.38 g (39%). ^1^H NMR (500 MHz, CDCl_3_) *δ* 5.34–5.32 (m, 1H), 5.30–5.27 (m, 1H), 5.24–5.23 (m, 1H), 4.85 (d, *J* = 2.0 Hz, 1H), 4.30–4.26 (m, 1H), 4.13–4.10 (m, 1H), 4.01–3.98 (m, 1H), 3.94 (s, 1H), 3.82–3.78 (m, 1H), 3.63–3.57 (m, 5H), 2.90–1.65 (m, 10H), 2.53 (t, *J* = 6.0 Hz, 2H), 2.16 (s, 3H), 2.11 (s, 3H), 2.06 (s, 3H), 2.00 (s, 3H). ^13^C NMR (151 MHz, CDCl_3_) *δ* 170.75, 170.22, 170.03, 169.88, 97.79, 73.18, 69.95, 69.61, 69.06, 69.01, 68.72, 67.33, 66.27, 62.65, 60.50, 37.65, 21.01, 20.88, 20.83, 20.80. HR-MS (ESI): *m*/*z* [M-H]^−^ calcd for C_20_H_37_B_10_O_11_^−^: 563.3272, found: 563.3290.

Synthesis of (2*R*,3*R*,4*S*,5*S*,6*R*)-2-(acetoxymethyl)-6-(2-(2-(2-(1,2-dicarba-*closo*-dodecaboran-1-yl)ethoxy)ethoxy)ethoxy)tetrahydro-2H-pyran-3,4,5-triyl triacetate (**62-2**): Following general synthetic procedure 6, **62-2** was prepared from **59-2** (0.47 g, 1.70 mmol) using α-D-mannose pentaacetate (1.33 g, 3.40 mmol) and BF_3_·Et_2_O (0.97 g, 6.80 mmol) in dried CH_2_Cl_2_ (4.7 mL). Column chromatography purification (EtOAc/PE = 1/3 by *v*/*v*) gave **62-2**. Colorless oil, 0.57 g (55%). ^1^H NMR (500 MHz, CDCl_3_) *δ* 5.36–5.33 (m, 1H), 5.30 (t, *J* = 5.0 Hz, 1H), 5.27–5.26 (m, 1H), 4.87 (d, *J* = 1.5 Hz, 1H), 4.31–4.27 (m, 1H), 4.15–4.13 (m, 1H), 4.12–4.09 (m, 1H), 4.05–4.01 (m, 1H), 3.82–3.78 (m, 1H), 3.67–3.64 (m, 3H), 3.62–3.60 (m, 2H), 3.59–3.54 (m, 4H), 2.92–1.70 (m, 10H), 2.53 (t, *J* = 6.0 Hz, 2H), 2.16 (s, 3H), 2.11 (s, 3H), 2.05 (s, 3H), 2.00 (s, 3H). ^13^C NMR (151 MHz, CDCl_3_) *δ* 170.84, 170.23, 170.13, 169.85, 97.91, 73.44, 70.60, 70.21, 70.14, 69.66, 69.19, 68.73, 68.64, 67.60, 66.25, 62.55, 60.47, 37.45, 21.04, 20.90, 20.85, 20.83. HR-MS (ESI): *m*/*z* [M-H]^−^ calcd for C_22_H_41_B_10_O_12_^−^: 607.3534, found: 607.3555.

Synthesis of (2*R*,3*R*,4*S*,5*S*,6*R*)-2-(2-(2-(1,2-dicarba-*closo*-dodecaboran-1-yl)ethoxy)ethoxy)-6-(hydroxymethyl)tetrahydro-2H-pyran-3,4,5-triol (**D1**): Following the general synthetic procedure 5, **D1** was prepared from **60-1** (0.21 g, 0.373 mmol) using Na (43 mg, 1.87 mmol) in dried MeOH (4.2 mL). Column chromatography purification (MeOH/CH_2_Cl_2_ = 1/10 by *v*/*v*) gave **D1**. White solid, 0.13 g (88%). m.p. 137.5–138.8 °C. [α]_D_^20^ = −8.5° (*c* = 10.0, MeOH). ^1^H NMR (500 MHz, DMSO-*d*_6_) *δ* 5.07 (s, 1H), 4.95–4.89 (m, 3H), 4.47 (t, *J* = 5.8 Hz, 1H), 4.14 (d, *J* = 8.0 Hz, 1H), 3.85–3.81 (m, 1H), 3.70–3.66 (m, 1H), 3.61–3.57 (m, 1H), 3.54–3.48 (m, 4H), 3.44–3.39 (m, 1H), 3.15–3.07 (m, 2H), 3.03–2.99 (m, 1H), 2.96–2.91 (m, 1H), 2.73–1.58 (m, 10H), 2.54–2.50 (m, 2H). ^13^C NMR (151 MHz, DMSO-*d*_6_) *δ* 103.03, 76.93, 76.74, 74.24, 73.43, 70.14, 69.38, 67.91, 67.76, 62.45, 61.18, 36.33. ^11^B NMR (160 MHz, CD_3_OD) *δ* −2.25, −3.17, −5.36, −6.29, −9.41, −10.35, −11.41, −12.47, −13.46. HR-MS (ESI): *m*/*z* [M+Na]^+^ calcd for C_12_H_30_B_10_NaO_7_^+^: 419.2814, found: 419.2812.

Synthesis of (2*R*,3*R*,4*S*,5*S*,6*R*)-2-(2-(2-(2-(1,2-dicarba-*closo*-dodecaboran-1-yl)ethoxy)ethoxy)ethoxy)-6-(hydroxymethyl)tetrahydro-2H-pyran-3,4,5-triol (**D2**): Following the general synthetic procedure 5, **D2** was prepared from **60-2** (0.29 g, 0.478 mmol) using Na (55 mg, 2.39 mmol) in dried MeOH (5.8 mL). Column chromatography purification (MeOH/CH_2_Cl_2_ = 1/15 by *v*/*v*) gave **D2**. Colorless oil, 0.17 g (81%). [α]_D_^20^ = −8.7° (*c* = 7.8, MeOH). ^1^H NMR (500 MHz, DMSO-*d*_6_) *δ* 5.06 (s, 1H), 4.95 (d, *J* = 5.0 Hz, 1H), 4.92 (d, *J* = 4.5 Hz, 1H), 4.89 (d, *J* = 5.0 Hz, 1H), 4.47 (t, *J* = 5.8 Hz, 1H), 4.14 (d, *J* = 8.0 Hz, 1H), 3.87–3.83 (m, 1H), 3.68–3.64 (m, 1H), 3.60–3.54 (m, 3H), 3.53–3.47 (m, 6H), 3.44–3.41 (m, 1H), 3.13–3.02 (m, 3H), 2.96–2.92 (m, 1H), 2.81–1.62 (m, 10H), 2.53–2.52 (m, 2H). ^13^C NMR (151 MHz, DMSO-*d*_6_) *δ* 103.01, 76.90, 76.78, 74.25, 73.41, 70.07, 69.71, 69.58, 69.42, 68.02, 67.85, 62.54, 61.10, 36.32. ^11^B NMR (161 MHz, DMSO-*d*_6_) *δ* −2.79, −3.71, −6.52, −9.55, −10.54, −11.89, −12.75, −15.04. HR-MS (ESI): *m*/*z* [M-H]^−^ calcd for C_14_H_33_B_10_O_8_^−^: 439.3111, found: 439.3097.

Synthesis of (2*R*,3*R*,4*S*,5*R*,6*R*)-2-(2-(2-(1,2-dicarba-*closo*-dodecaboran-1-yl)ethoxy)ethoxy)-6-(hydroxymethyl)tetrahydro-2H-pyran-3,4,5-triol (**D3**): Following the general synthetic procedure 5, **D3** was prepared from **61-1** (0.27 g, 0.480 mmol) using Na (55 mg, 2.40 mmol) in dried MeOH (5.4 mL). Column chromatography purification (MeOH/CH_2_Cl_2_ = 1/15 by *v*/*v*) gave **D3**. Colorless oil, 0.10 g (53%). [α]_D_^20^ = −2.9° (*c* = 5.2, MeOH). ^1^H NMR (500 MHz, DMSO-*d*_6_) *δ* 5.07 (s, 1H), 4.75 (d, *J* = 4.0 Hz, 1H), 4.69 (d, *J* = 5.0 Hz, 1H), 4.54 (t, *J* = 5.5 Hz, 1H), 4.33 (d, *J* = 4.0 Hz, 1H), 4.09 (d, *J* = 6.5 Hz, 1H), 3.81–3.78 (m, 1H), 3.63–3.62 (m, 1H), 3.57–3.46 (m, 7H), 3.32–3.23 (m, 3H), 2.89–1.59 (m, 10H), 2.53–2.52 (m, 2H). ^13^C NMR (151 MHz, DMSO-*d*_6_) *δ* 103.60, 75.21, 74.18, 73.50, 70.49, 69.44, 68.09, 68.04, 67.63, 62.59, 60.41, 36.31. ^11^B NMR (161 MHz, DMSO-*d*_6_) *δ* −2.78, −3.70, −5.65, −6.51, −9.50, −10.45, −11.43, −11.76, −13.20, −13.78. HR-MS (ESI): *m*/*z* [M+H]^+^ calcd for C_12_H_31_B_10_O_7_^+^: 397.2995, found: 397.2984.

Synthesis of (2*R*,3*R*,4*S*,5*R*,6*R*)-2-(2-(2-(2-(1,2-dicarba-*closo*-dodecaboran-1-yl)ethoxy)ethoxy)ethoxy)-6-(hydroxymethyl)tetrahydro-2H-pyran-3,4,5-triol (**D4**): Following the general synthetic procedure 5, **D4** was prepared from **61-2** (0.22 g, 0.363 mmol) using Na (50 mg, 2.18 mmol) in dried MeOH (4.4 mL). Column chromatography purification (MeOH/CH_2_Cl_2_ = 1/15 by *v*/*v*) gave **D4**. Colorless oi, 0.11 g (69%). [α]_D_^20^ = −2.7° (*c* = 6.8, MeOH). ^1^H NMR (500 MHz, DMSO-*d*_6_) *δ* 5.06 (s, 1H), 4.80 (d, *J* = 4.0 Hz, 1H), 4.68 (d, *J* = 5.0 Hz, 1H), 4.55 (t, *J* = 5.8 Hz, 1H), 4.34 (d, *J* = 4.5 Hz, 1H), 4.09 (d, *J* = 6.5 Hz, 1H), 3.84–3.80 (m, 1H), 3.63–3.61 (m, 1H), 3.57–3.51 (m, 7H), 3.49–3.46 (m, 4H), 3.31–3.23 (m, 3H), 2.81–1.58 (m, 10H), 2.53–2.52 (m, 2H). ^13^C NMR (126 MHz, DMSO-*d*_6_) *δ* 103.62, 75.20, 74.27, 73.50, 70.52, 69.74, 69.58, 69.41, 68.13, 68.01, 67.75, 62.53, 60.41, 36.32. ^11^B NMR (161 MHz, DMSO-*d*_6_) *δ* −2.76, −3.69, −5.74, −6.63, −9.50, −10.49, −11.61, −12.38, −13.80. HR-MS (ESI): *m*/*z* [M-H]^−^ calcd for C_14_H_33_B_10_O_8_^−^: 439.3111, found: 439.3099.

Synthesis of (2*R*,3*S*,4*S*,5*S*,6*R*)-2-(2-(2-(1,2-dicarba-*closo*-dodecaboran-1-yl)ethoxy)ethoxy)-6-(hydroxymethyl)tetrahydro-2H-pyran-3,4,5-triol (**D5**): Following the general synthetic procedure 5, **D5** was prepared from **62-1** (0.35 g, 0.622 mmol) using Na (57 mg, 2.49 mmol) in dried MeOH (7 mL). Column chromatography purification (MeOH/CH_2_Cl_2_ = 1/10 by *v*/*v*) gave **D5**. Colorless oil, 0.19 g (77%). [α]_D_^20^ = +33.2° (*c* = 6.6, MeOH). ^1^H NMR (500 MHz, DMSO-*d*_6_) *δ* 5.03 (s, 1H), 4.70 (t, *J* = 4.8 Hz, 2H), 4.62 (s, 1H), 4.53 (d, *J* = 6.0 Hz, 1H), 4.42 (t, *J* = 6.0 Hz, 1H), 3.68–3.62 (m, 2H), 3.60–3.58 (m, 1H), 3.52–3.47 (m, 4H), 3.46–3.41 (m, 3H), 3.39–3.35 (m, 1H), 3.31–3.27 (m, 1H), 2.87–1.60 (m, 10H), 2.53–2.51 (m, 2H). ^13^C NMR (151 MHz, DMSO-*d*_6_) *δ* 99.92, 74.23, 73.93, 70.99, 70.25, 69.14, 67.98, 67.00, 65.50, 62.40, 61.26, 36.33. ^11^B NMR (161 MHz, DMSO-*d*_6_) *δ* −2.56, −3.49, −5.65, −6.50, −9.42, −10.39, −11.43, −12.54, −13.60. HR-MS (ESI): *m*/*z* [M-H]^−^ calcd for C_12_H_29_B_10_O_7_^−^: 395.2849, found: 395.2838.

Synthesis of (2*R*,3*S*,4*S*,5*S*,6*R*)-2-(2-(2-(2-(1,2-dicarba-*closo*-dodecaboran-1-yl)ethoxy)ethoxy)ethoxy)-6-(hydroxymethyl)tetrahydro-2H-pyran-3,4,5-triol (**D6**): Following the general synthetic procedure 5, **D6** was prepared from **62-2** (0.55 g, 0.907 mmol) using Na (63 mg, 2.72 mmol) in dried MeOH (11 mL). Column chromatography purification (MeOH/CH_2_Cl_2_ = 1/12 by *v*/*v*) gave **D6**. Colorless oil, 0.30 g (75%). [α]_D_^20^ = +31.1° (*c* = 8.6, MeOH). ^1^H NMR (500 MHz, DMSO-*d*_6_) *δ* 5.05 (s, 1H), 4.70 (dd, *J* = 11.0, 4.5 Hz, 2H), 4.62 (s, 1H), 4.53 (d, *J* = 6.0 Hz, 1H), 4.42 (t, *J* = 6.0 Hz, 1H), 3.68–3.62 (m, 2H), 3.59–3.58 (m, 1H), 3.55–3.43 (m, 11H), 3.39–3.36 (m, 1H), 3.31–3.28 (m, 1H), 2.85–1.57 (m, 10H), 2.53–2.52 (m, 2H). ^13^C NMR (151 MHz, DMSO-*d*_6_) *δ* 99.99, 74.27, 73.92, 70.95, 70.29, 69.56, 69.50, 69.39, 68.02, 66.94, 65.72, 62.49, 61.26, 36.30. ^11^B NMR (161 MHz, DMSO-*d*_6_) *δ* −2.75, −3.66, −5.62, −6.55, −9.44, −9.57, −10.48, −11.56, −12.45, −13.64. HR-MS (ESI): *m*/*z* [M-H]^−^ calcd for C_14_H_33_B_10_O_8_^−^: 439.3111, found: 439.3113.

Synthesis of (3*R*,4*S*,5*S*,6*R*)-6-(hydroxymethyl)-2-(4-iodophenyl)-2-methoxytetrahydro-2H-pyran-3,4,5-triol (**63**): Following the general synthetic procedure 2, **63** was prepared from **2** (10.00 g, 21.4 mmol) and 1,4-diiodide (8.48 g, 25.6 mmol) using *n*-BuLi (16.1 mL, 1.6 M in THF, 25.76 mmol), MeSO_3_H (4.12 g, 42.8 mmol), MeOH (21 mL) and dried THF (120 mL) as a crude sample. Brown oil, 8.76 g. This sample was used directly in the next step without further purification or characterization.

Synthesis of (3*R*,4*S*,5*R*,6*R*)-6-(acetoxymethyl)-2-(4-iodophenyl)-2-methoxytetrahydro-2H-pyran-3,4,5-triyl triacetate (**64**): Following the procedure for the synthesis of **31** from **30**, **64** was prepared from crude **63** prepared above (8.76 g, deemed to be 22.2 mmol) using DMAP (3.24 g, 26.6 mmol), Ac_2_O (36 mL), and pyridine (70 mL). Column chromatography purification (EtOAc/PE = 1/3 by *v*/*v*) gave **64**. Colorless oil, 5.31 g (44% overall for **2** to **64**). ^1^H NMR (500 MHz, CDCl_3_) *δ* 7.70 (d, *J* = 8.5 Hz, 2H), 7.18 (d, *J* = 8.5 Hz, 2H), 5.60 (t, *J* = 9.8 Hz, 1H), 5.22 (t, *J* = 10.0 Hz, 1H), 4.95 (d, *J* = 10.0 Hz, 1H), 4.36 (dd, *J* = 12.0, 5.0 Hz, 1H), 4.23 (dd, *J* = 12.3, 2.3 Hz, 1H), 4.07–4.03 (m, 1H), 3.12 (s, 3H), 2.12 (s, 3H), 2.06 (s, 3H), 1.96 (s, 6H) ^13^C NMR (151 MHz, CDCl_3_) *δ* 170.84, 170.31, 169.69, 169.11, 137.73, 135.79, 128.76, 100.29, 95.65, 73.96, 71.43, 69.08, 68.90, 62.27, 49.66, 20.91, 20.80, 20.77, 20.62. HR-MS (ESI): *m*/*z* [M+Na]^+^ calcd for C_21_H_25_INaO_10_^+^: 587.0385, found: 587.0387.

Synthesis of (2*R*,3*R*,4*R*,5*S*,6*S*)-2-(acetoxymethyl)-6-(4-iodophenyl)tetrahydro-2H-pyran-3,4,5-triyl triacetate (**65**): Following the general synthetic procedure 3, **65** was prepared from **64** (1.00 g, 1.77 mmol) using Et_3_SiH (0.25 g, 2.13 mmol) and BF_3_·Et_2_O (0.50 g, 3.54 mmol) in dried CH_2_Cl_2_ (10 mL). Column chromatography purification (EtOAc/PE = 1/5 by *v*/*v*) gave **65**. White solid, 0.80 g (84%). m.p. 142.1–143.7 °C. ^1^H NMR (500 MHz, CDCl_3_) *δ* 7.69–7.66 (m, 2H), 7.10–7.07 (m, 2H), 5.32 (t, *J* = 9.5 Hz, 1H), 5.22 (t, *J* = 9.8 Hz, 1H), 5.09 (t, *J* = 9.5 Hz, 1H), 4.35 (d, *J* = 9.5 Hz, 1H), 4.28 (dd, *J* = 12.5, 5.0 Hz, 1H), 4.17–4.12 (m, 1H), 3.84–3.81 (m, 1H), 2.09 (s, 3H), 2.06 (s, 3H), 2.00 (s, 3H), 1.83 (s, 3H). ^13^C NMR (151 MHz, CDCl_3_) *δ* 170.88, 170.53, 169.65, 169.00, 137.76, 136.11, 129.14, 94.99, 79.82, 74.29, 72.58, 68.65, 62.45, 20.92, 20.80, 20.78, 20.57. HR-MS (ESI): *m*/*z* [M+Na]^+^ calcd for C_20_H_23_INaO_9_^+^: 557.0279, found: 557.0280.

Synthesis of (2*R*,3*R*,4*R*,5*S*,6*S*)-2-(acetoxymethyl)-6-(4-(4,4,5,5-tetramethyl-1,3,2-dioxaborolan-2-yl)phenyl)tetrahydro-2H-pyran-3,4,5-triyl triacetate (**66**): Following the general synthetic procedure 7, **66** was prepared from **65** (0.80 g, 1.50 mmol) using B_2_Pin_2_ (0.57 g, 2.25 mmol), KOAc (0.44 g, 4.50 mmol) and Pd(dppf)Cl_2_ (0.20 g, 0.300 mmol) in dried DMSO (8 mL). Column chromatography purification (EtOAc/PE = 1/2 by *v*/*v*) gave **66**. White foam, 0.58 g (72%). m.p. 127.8–133.4 °C. ^1^H NMR (500 MHz, CDCl_3_) *δ* 7.77 (d, *J* = 8.0 Hz, 2H), 7.34 (d, *J* = 8.0 Hz, 2H), 5.33 (t, *J* = 9.3 Hz, 1H), 5.23 (t, *J* = 9.8 Hz, 1H), 5.13 (t, *J* = 9.8 Hz, 1H), 4.41 (d, *J* = 10.0 Hz, 1H), 4.29 (dd, *J* = 12.5, 5.0 Hz, 1H), 4.16 (dd, *J* = 12.3, 2.3 Hz, 1H), 3.85–3.82 (m, 1H), 2.08 (s, 3H), 2.06 (s, 3H), 2.00 (s, 3H), 1.80 (s, 3H), 1.34 (s, 12H). ^13^C NMR (151 MHz, CDCl_3_) *δ* 170.87, 170.52, 169.63, 168.98, 139.27, 134.96, 126.50, 83.99, 80.28, 76.25, 74.42, 72.72, 68.77, 62.53, 25.03, 24.96, 20.89, 20.78, 20.76, 20.53. HR-MS (ESI): *m*/*z* [M+NH_4_]^+^ calcd for C_26_H_39_BNO_11_^+^: 552.2611, found: 552.2610.

Synthesis of (2*R*,3*S*,4*R*,5*R*,6*S*)-2-(hydroxymethyl)-6-(4-(4,4,5,5-tetramethyl-1,3,2-dioxaborolan-2-yl)phenyl)tetrahydro-2H-pyran-3,4,5-triol (**E1**): Following the general synthetic procedure 5, **E1** was prepared from **66** (0.43 g, 1.17 mmol) using Na (50 mg, 2.17 mmol) in dried MeOH (8.6 mL). Column chromatography purification (MeOH/CH_2_Cl_2_ = 1/10 by *v*/*v*) gave **E1**. White solid, 0.94 g (32%). m.p. 176.8–180.1 °C. [α]_D_^20^ = +12.9° (*c* = 10.0, DMSO). ^1^H NMR (500 MHz, DMSO-*d*_6_) *δ* 7.62 (d, *J* = 7.5 Hz, 2H), 7.35 (d, *J* = 8.0 Hz, 2H), 4.95–4.93 (m, 2H), 4.78 (d, *J* = 6.0 Hz, 1H), 4.45 (t, *J* = 5.5 Hz, 1H), 4.02 (d, *J* = 9.5 Hz, 1H), 3.73–3.69 (m, 1H), 3.48–3.43 (m, 1H), 3.28–3.26 (m, 1H), 3.24–3.22 (m, 1H), 3.19–3.16 (m, 1H), 3.13–3.08 (m, 1H), 1.29 (s, 12H). ^13^C NMR (151 MHz, DMSO-*d*_6_) δ 143.82, 133.85, 129.01, 127.24, 83.57, 81.32, 81.20, 78.42, 74.92, 70.39, 61.44, 24.72, 24.67. HR-MS (ESI): *m*/*z* [M+NH_4_]^+^ calcd for C_18_H_31_BNO_7_^+^: 384.2188, found: 384.2184.

Synthesis of (4-((2*S*,3*S*,4*R*,5*R*,6*R*)-3,4,5-triacetoxy-6-(acetoxymethyl)tetrahydro-2H-pyran-2-yl)phenyl)boronic acid (**67**): Following the general synthetic procedure 8, **67** was prepared from **66** (1.20 g, 2.25 mmol) using NaIO_4_ (1.21 g, 5.61 mmol) and NH_4_OAc (0.26 g, 3.37 mmol) in a mixture of acetone (9.6 mL) and H_2_O(4.5 mL). Column chromatography purification (MeOH/CH_2_Cl_2_ = 1/10 by *v*/*v*) gave **67**. White solid, 0.77 g (76%). m.p. 288.8–292.3 °C. ^1^H NMR (500 MHz, DMSO-*d*_6_) *δ* 8.05 (s, 2H), 7.74 (d, *J* = 8.0 Hz, 2H), 7.29 (d, *J* = 7.5 Hz, 2H), 5.37 (t, *J* = 9.5 Hz, 1H), 5.07 (t, *J* = 9.8 Hz, 1H), 5.00 (t, *J* = 9.8 Hz, 1H), 4.69 (d, *J* = 9.5 Hz, 1H), 4.16–4.12 (m, 1H), 4.10–4.06 (m, 2H), 2.02 (s, 3H), 2.01 (s, 3H), 1.93 (s, 3H), 1.75 (s, 3H). ^13^C NMR (151 MHz, DMSO-*d*_6_) *δ* 170.18, 169.71, 169.48, 168.56, 138.61, 134.50, 134.01, 126.27, 78.24, 74.72, 73.53, 72.36, 68.56, 62.43, 20.57, 20.46, 20.34, 20.14. HR-MS (ESI): *m*/*z* [M+NH_4_]^+^ calcd for C_20_H_29_BNO_11_^+^: 470.1828, found: 470.1833.

Synthesis of (4-((2*S*,3*R*,4*R*,5*S*,6*R*)-3,4,5-trihydroxy-6-(hydroxymethyl)tetrahydro-2H-pyran-2-yl)phenyl)boronic acid (**E2**): Following the general synthetic procedure 5, **E2** was prepared from **67** (0.64 g, 1.42 mmol) using Na (55 mg, 2.39 mmol) in dried MeOH (12.8 mL). Column chromatography purification (MeOH/CH_2_Cl_2_ = 1/10 by *v*/*v*) gave **E2**. White solid, 0.30 g (74%). m.p. 330 °C (decomposition). [α]_D_^20^ = +7.9° (*c* = 3.4, H_2_O). ^1^H NMR (500 MHz, D_2_O) *δ* 7.78 (d, *J* = 7.5 Hz, 2H), 7.48 (d, *J* = 7.5 Hz, 2H), 4.33–4.31 (m, 1H), 3.90 (d, *J* = 12.5 Hz, 1H), 3.81–3.77 (m, 1H), 3.65–3.59 (m, 4H). ^13^C NMR (151 MHz, D_2_O) *δ* 139.52, 133.62, 129.66, 127.40, 81.87, 80.02, 77.28, 74.01, 69.76, 60.85. HR-MS (ESI): *m*/*z* [M+Na]^+^ calcd for C_12_H_17_BNaO_7_^+^: 307.0960, found: 307.0963.

Synthesis of (3*R*,4*S*,5*S*,6*R*)-6-(hydroxymethyl)-2-(3-iodophenyl)-2-methoxytetrahydro-2H-pyran-3,4,5-triol (**68**): Following the general synthetic procedure 2, **68** was prepared from **2** (10.00 g, 21.4 mmol) and 1,3-diiodobenzene (8.48 g, 25.6 mmol) using *n*-BuLi (16.1 mL, 1.6 M in THF, 25.76 mmol), MeSO_3_H (4.12 g, 42.8mmol), MeOH (21 mL), and dried THF (120 mL) as a crude sample. Yellow oil, 9.86 g. This sample was used directly in the next step without further purification or characterization.

Synthesis of (3*R*,4*S*,5*R*,6*R*)-6-(acetoxymethyl)-2-(3-iodophenyl)-2-methoxytetrahydro-2H-pyran-3,4,5-triyl triacetate (**69**): Following the procedure for the synthesis of **31** from **30**, **69** was prepared from crude **68** prepared above (9.86 g, deemed to be 24.8 mmol) using DMAP (3.66 g, 29.8 mmol), Ac_2_O (40 mL), and pyridine (80 mL). Column chromatography purification (EtOAc/PE = 1/3 by *v*/*v*) gave **69**. Yellow foam, 6.04 g. This sample was used directly in the next step without characterization.

Synthesis of (2*R*,3*R*,4*R*,5*S*,6*S*)-2-(acetoxymethyl)-6-(3-iodophenyl)tetrahydro-2H-pyran-3,4,5-triyl triacetate (**70**): Following the general synthetic procedure 3, **70** was prepared from **69** (6.04 g, 10.7 mmol) using Et_3_SiH (1.41 g, 12.1 mmol) and BF_3_·Et_2_O (1.03 g, 7.25 mmol) in dried CH_2_Cl_2_ (60 mL). Column chromatography purification (EtOAc/PE = 1/5 by *v*/*v*) gave **70**. White solid, 1.62 g (14% overall for **2** to **70**). m.p. 136.4–144.2 °C. ^1^H NMR (500 MHz, CDCl_3_) *δ* 7.68–7.65 (m, 2H), 7.34–7.32 (m, 1H), 7.09 (t, *J* = 7.8 Hz, 1H), 5.32 (t, *J* = 9.5 Hz, 1H), 5.21 (t, *J* = 9.8 Hz, 1H), 5.05 (t, *J* = 9.8 Hz, 1H), 4.34 (d, *J* = 9.5 Hz, 1H), 4.29 (dd, *J* = 12.5, 5.0 Hz, 1H), 4.17 (dd, *J* = 12.3, 2.3 Hz, 1H), 3.84–3.81 (m, 1H), 2.10 (s, 3H), 2.06 (s, 3H), 2.00 (s, 3H), 1.86 (s, 3H). ^13^C NMR (126 MHz, CDCl_3_) *δ* 170.88, 170.50, 169.62, 168.98, 138.70, 138.04, 136.33, 130.38, 126.19, 94.13, 79.37, 76.37, 74.18, 72.73, 68.62, 62.42, 20.93, 20.79, 20.77, 20.58. HR-MS (ESI): *m*/*z* [M+H]^+^ calcd for C_20_H_24_IO_9_^+^: 535.0460, found: 535.0457.

Synthesis of (2R,3R,4R,5S,6S)-2-(acetoxymethyl)-6-(3-(4,4,5,5-tetramethyl-1,3,2-dioxaborolan-2-yl)phenyl)tetrahydro-2H-pyran-3,4,5-triyl triacetate (**71**): Following the general synthetic procedure 7, **71** was prepared from **70** (3.55 g, 6.66 mmol) using B_2_Pin_2_ (3.38 g, 13.3 mmol), KOAc (1.96 g, 20.0 mmol), and Pd(dppf)Cl_2_ (1.46 g, 1.20 mmol) in dried DMSO (36 mL). Column chromatography purification (EtOAc/PE = 1/1 by *v*/*v*) gave **71**. Colorless oil, 1.35 g (38%). ^1^H NMR (500 MHz, CDCl_3_) *δ* 7.76–7.74 (m, 2H), 7.49–7.746 (m, 1H), 7.36 (t, *J* = 7.5 Hz, 1H), 5.33 (t, *J* = 9.5 Hz, 1H), 5.23 (t, *J* = 9.8 Hz, 1H), 5.14 (t, *J* = 9.5 Hz, 1H), 4.42 (d, *J* = 9.5 Hz, 1H), 4.28 (dd, *J* = 12.5, 5.0 Hz, 1H), 4.18 (dd, *J* = 12.5, 2.5 Hz, 1H), 3.85–3.81 (m, 1H), 2.09 (s, 3H), 2.06 (s, 3H), 2.00 (s, 3H), 1.81 (s, 3H), 1.37–1.34 (m, 12H). ^13^C NMR (201 MHz, CDCl_3_) *δ* 170.92, 170.54, 169.65, 169.07, 135.64, 135.42, 133.97, 129.69, 128.59, 128.13, 84.02, 80.32, 76.31, 74.47, 72.76, 68.82, 62.57, 25.04, 24.98, 20.93, 20.81, 20.78, 20.51. HR-MS (ESI): *m*/*z* [M+NH_4_]^+^ calcd for C_26_H_39_BNO_11_^+^: 552.2611, found: 552.2607.

Synthesis of (2*R*,3*S*,4*R*,5*R*,6*S*)-2-(hydroxymethyl)-6-(3-(4,4,5,5-tetramethyl-1,3,2-dioxaborolan-2-yl)phenyl)tetrahydro-2H-pyran-3,4,5-triol (**E3**): Following the general synthetic procedure 5, **E3** was prepared from **71** (0.22 g, 0.599 mmol) using Na (30 mg, 1.30 mmol) in MeOH (5 mL). Column chromatography purification (MeOH/CH_2_Cl_2_ = 1/10 by *v*/*v*) gave **E3**. White solid, 73 mg (49%). m.p. 151.7–160.5 °C. [α]_D_^20^ = +4.1° (*c* = 11.6, DMSO). ^1^H NMR (500 MHz, DMSO-*d*_6_) *δ* 7.66 (s, 1H), 7.58–7.56 (m, 1H), 7.45–7.43 (m, 1H), 7.32 (t, *J* = 7.3 Hz, 1H), 4.94–4.91 (m, 2H), 4.76 (d, *J* = 6.0 Hz, 1H), 4.47 (t, *J* = 5.5 Hz, 1H), 4.02 (d, *J* = 9.5 Hz, 1H), 3.72–3.68 (m, 1H), 3.46–3.44 (m, 1H), 3.28–3.16 (m, 4H), 1.30 (s, 12H). ^13^C NMR (151 MHz, DMSO-*d*_6_) *δ* 139.80, 133.70, 133.49, 131.33, 127.85, 127.22, 83.64, 81.54, 81.33, 78.49, 70.46, 67.32, 61.38, 24.75, 24.66. HR-MS (ESI): *m*/*z* [M+H]^+^ calcd for C_18_H_28_BO_7_^+^: 367.1923, found: 367.1918.

Synthesis of (3-((2*S*,3*S*,4*R*,5*R*,6*R*)-3,4,5-triacetoxy-6-(acetoxymethyl)tetrahydro-2H-pyran-2-yl)phenyl)boronic acid (**72**): Following the general synthetic procedure 8, **72** was prepared from **71** (0.63 g, 1.18 mmol) using NaIO_4_ (0.64 g, 2.95 mmol) and NH_4_OAc (0.14 g, 1.77 mmol) in a mixture of acetone (5.1 mL) and H_2_O (1.3 mL). Column chromatography purification (MeOH/CH_2_Cl_2_ = 1/10 by *v*/*v*) gave **72**. White solid, 0.32 g (76%). m.p. 214.4–217.3 °C. ^1^H NMR (500 MHz, DMSO-*d*_6_) *δ* 8.07 (s, 2H), 7.76–7.73 (m, 2H), 7.37 (d, *J* = 8.0 Hz, 1H), 7.31 (t, *J* = 7.5 Hz, 1H), 5.39 (t, *J* = 9.3 Hz, 1H),5.06 (t, *J* = 9.3 Hz, 1H), 4.99 (t, *J* = 9.8 Hz, 1H), 4.68 (d, *J* = 10.0 Hz, 1H), 4.15–4.07 (m, 3H), 2.02 (s, 3H), 2.01 (s, 3H), 1.93 (s, 3H), 1.73 (s, 3H). ^13^C NMR (151 MHz, DMSO-*d*_6_) *δ* 170.18, 169.70, 169.48, 168.54, 135.76, 134.33, 133.98, 133.32, 128.84, 127.30, 78.46, 74.66, 73.50, 72.41, 68.57, 62.43, 20.59, 20.46, 20.35, 20.12. HR-MS (ESI): *m*/*z* [M+H]^+^ calcd for C_20_H_26_BO_11_^+^: 453.1563, found: 453.1555.

Synthesis of (3-((2*S*,3*R*,4*R*,5*S*,6*R*)-3,4,5-trihydroxy-6-(hydroxymethyl)tetrahydro-2H-pyran-2-yl)phenyl)boronic acid (**E4**): Following the general synthetic procedure 5, **E4** was prepared from **72** (0.30 g, 0.663 mmol) using Na (50 mg, 2.17 mmol) in MeOH (8 mL). Column chromatography purification (Me/CH_2_Cl_2_ = 1/10 by *v*/*v*) gave **E4**. White solid, 0.13 g (66%). m.p. 340 °C (decomposition). [α]_D_^20^ = +1.4° (*c* = 7.8, DMSO). ^1^H NMR (500 MHz, DMSO-*d*_6_) *δ* 8.01 (s, 2H), 7.78 (s, 1H), 7.68 (d, *J* = 7.5 Hz, 1H), 7.35 (d, *J* = 7.5 Hz, 1H), 7.26 (t, *J* = 7.5 Hz, 1H), 4.96–4.94 (m, 2H), 4.73 (d, *J* = 5.5 Hz, 1H), 4.43 (t, *J* = 5.8 Hz, 1H), 3.99 (d, *J* = 9.5 Hz, 1H), 3.72–3.68 (m, 1H), 3.46–3.43 (m, 1H), 3.27–3.26 (m, 1H), 3.23–3.22 (m, 1H), 3.19–3.17 (m, 1H). HR-MS (ESI): *m*/*z* [M+K]^+^ calcd for C_12_H_17_BKO_7_^+^: 323.0699, found: 323.0709.

Synthesis of (2*R*,3*R*,4*R*,5*R*,6*S*)-2-(acetoxymethyl)-6-(4-iodophenyl)tetrahydro-2H-pyran-3,4,5-triyl triacetate (**73**): Following the procedure for the synthesis of **37** from **36**, **73** was prepared from **20** (1.70 g, 2.34 mmol) using Ac_2_O (17 mL) and BF_3_·Et_2_O (17 mL). Column chromatography purification (EtOAc/PE = 1/5 by *v*/*v*) gave **73**. Yellow oil, 1.00 g (80%). ^1^H NMR (500 MHz, CDCl_3_) *δ* 7.65 (d, *J* = 8.5 Hz, 2H), 7.07 (d, *J* = 8.0 Hz, 2H), 5.54–5.53 (m, 1H), 5.32 (t, *J* = 9.8 Hz, 1H), 5.23 (dd, *J* = 10.3, 3.3 Hz, 1H), 4.71 (s, 1H), 4.33 (dd, *J* = 12.3, 5.8 Hz, 1H), 4.22 (dd, *J* = 12.3, 2.3 Hz, 1H), 3.83–3.79 (m, 1H), 2.10 (s, 3H), 2.08 (s, 3H), 1.99 (s, 3H), 1.93 (s, 3H). ^13^C NMR (126 MHz, CDCl_3_) *δ* 170.87, 170.36, 170.03, 169.89, 137.44, 136.01, 128.12, 93.90, 77.87, 76.59, 72.47, 70.38, 66.11, 63.06, 20.94, 20.88, 20.76, 20.58. HR-MS (ESI): *m*/*z* [M+H]^+^ calcd for C_20_H_24_IO_9_^+^: 535.0460, found: 535.0457.

Synthesis of (2*R*,3*R*,4*R*,5*R*,6*S*)-2-(acetoxymethyl)-6-(4-(4,4,5,5-tetramethyl-1,3,2-dioxaborolan-2-yl)phenyl)tetrahydro-2H-pyran-3,4,5-triyl triacetate (**74**): Following the general synthetic procedure 7, **74** was prepared from **73** (0.75 g, 1.41 mmol) using B_2_Pin_2_ (0.54 g, 2.11 mmol), KOAc (0.42 g, 4.23 mmol), and Pd(dppf)Cl_2_ (0.21 g, 0.280 mmol) in dried DMSO (7.5 mL). Column chromatography purification (EtOAc/PE = 1/1 by *v*/*v*) gave **74**. Colorless oil, 0.65 g (87%). ^1^H NMR (500 MHz, CDCl_3_) *δ* 7.75 (d, *J* = 8.0 Hz, 2H), 7.32 (d, *J* = 7.5 Hz, 2H), 5.57–5.56 (m, 1H), 5.33 (t, *J* = 10.0 Hz, 1H), 5.25 (dd, *J* = 10.0, 3.0 Hz, 1H), 4.77 (s, 1H), 4.35 (dd, *J* = 12.0, 6.0 Hz, 1H), 4.23 (dd, *J* = 12.3, 2.3 Hz, 1H), 3.84–3.801 (m, 1H), 2.10 (s, 3H), 2.08 (s, 3H), 1.99 (s, 3H), 1.90 (s, 3H), 1.34 (s, 12H). ^13^C NMR (151 MHz, CDCl_3_) *δ* 170.92, 170.39, 170.06, 169.93, 139.25, 134.74, 128.33, 125.47, 83.97, 78.46, 76.55, 72.62, 70.63, 66.34, 63.19, 25.06, 24.93, 20.94, 20.89, 20.77, 20.58. HR-MS (ESI): *m*/*z* [M+NH_4_]^+^ calcd for C_26_H_39_BNO_11_^+^: 552.2611, found: 552.2611.

Synthesis of (2*R*,3*S*,4*R*,5*S*,6*S*)-2-(hydroxymethyl)-6-(4-(4,4,5,5-tetramethyl-1,3,2-dioxaborolan-2-yl)phenyl)tetrahydro-2H-pyran-3,4,5-triol (**E5**): Following the general synthetic procedure 5, **E5** was prepared from **74** (0.50 g, 0.936 mmol) using Na (50 mg, 2.17 mmol) in dried MeOH (10 mL). Column chromatography purification (MeOH/CH_2_Cl_2_ = 1/10 by *v*/*v*) gave **E5**. White solid, 0.17 g (49%). m.p. 149.8–155.7 °C. [α]_D_^20^ = +3.2° (*c* = 12.8, DMSO). ^1^H NMR (500 MHz, DMSO-*d*_6_) *δ* 7.60 (d, *J* = 7.5 Hz, 2H), 7.39 (d, *J* = 8.0 Hz, 2H), 4.78 (d, *J* = 5.5 Hz, 1H), 4.68 (d, *J* = 5.5 Hz, 1H), 4.49 (s, 1H), 4.44 (t, *J* = 5.8 Hz, 1H), 4.15 (d, *J* = 5.5 Hz, 1H), 3.78–3.75 (m, 2H), 3.53–3.47 (m, 2H), 3.43–3.39 (m, 1H), 3.21–3.18 (m, 1H), 1.29 (s, 12H). ^13^C NMR (151 MHz, DMSO-*d*_6_) *δ* 143.72, 133.61, 127.34, 126.11, 83.49, 81.43, 78.98, 75.09, 72.19, 67.10, 61.70, 24.70, 24.67. HR-MS (ESI): *m*/*z* [M+NH_4_]^+^ calcd for C_18_H_31_BNO_7_^+^: 384.2188, found: 384.2187.

Synthesis of (4-((2*S*,3*R*,4*R*,5*R*,6*R*)-3,4,5-triacetoxy-6-(acetoxymethyl)tetrahydro-2H-pyran-2-yl)phenyl)boronic acid (**75**): Following the general synthetic procedure 8, **75** was prepared from **74** (1.32 g, 2.10 mmol) using NaIO_4_ (1.34 g, 6.18 mmol) and NH_4_OAc (0.29 g, 3.71 mmol) in a mixture of acetone (10.6 mL) and H_2_O (2.6 mL). Column chromatography purification (MeOH/CH_2_Cl_2_ = 1/10 by *v*/*v*) gave **75**. Colorless oil, 0.66 g (59%). ^1^H NMR (500 MHz, DMSO-*d*_6_) *δ* 8.02 (s, 2H), 7.74–7.72 (m, 2H), 7.27–7.25 (m, 2H), 5.42–5.41 (m, 1H), 5.39–5.36 (m, 1H), 5.15–5.11 (m, 2H), 4.20 (dd, *J* = 12.3, 5.8 Hz, 1H), 4.14 (dd, *J* = 12.0, 2.5 Hz, 1H), 4.04–4.00 (m, 1H), 2.05 (s, 3H), 2.04 (s, 3H), 1.92 (s, 3H), 1.86 (s, 3H). ^13^C NMR (151 MHz, DMSO-*d*_6_) *δ* 170.15, 169.70, 169.57, 169.45, 138.57, 133.77, 133.58, 125.07, 76.78, 74.97, 71.59, 70.65, 65.94, 62.59, 20.60, 20.54, 20.37, 20.18. HR-MS (ESI): *m*/*z* [M+NH_4_]^+^ calcd for C_20_H_29_BNO_11_^+^: 470.1828, found: 470.1830.

Synthesis of (4-((2*S*,3*S*,4*R*,5*S*,6*R*)-3,4,5-trihydroxy-6-(hydroxymethyl)tetrahydro-2H-pyran-2-yl)phenyl)boronic acid (**E6**): Following the general synthetic procedure 5, **E6** was prepared from **75** (0.65 g, 1.44 mmol) using Na (50 mg, 2.17 mmol) in dried MeOH (13 mL). White solid, 0.16 g (39%). m.p. 348.9–356.7 °C. [α]_D_^20^ = +60.0° (*c* = 0.1, H_2_O-DMSO (1/1 by *v*/*v*)). ^1^H NMR (500 MHz, D_2_O) *δ* 7.79 (d, *J* = 8.0 Hz, 2H), 7.49 (d, *J* = 8.0 Hz, 2H), 4.94–4.90 (m, 1H), 4.14–4.13 (m, 1H), 3.99 (dd, *J* = 12.5, 2.5 Hz, 1H), 3.89–3.84 (m, 2H), 3.71 (t, *J* = 9.8 Hz, 1H), 3.56–3.53 (m, 1H). ^13^C NMR (151 MHz, D_2_O) *δ* 140.91, 133.56, 127.79, 125.62, 80.10, 79.12, 74.28, 72.52, 66.91, 61.24. HR-MS (ESI): *m*/*z* [M+Na]^+^ calcd for C_12_H_17_BNaO_7_^+^: 307.0960, found: 307.0960.

Synthesis of (3*S*,4*S*,5*R*,6*R*)-3,4,5-tris(benzyloxy)-6-((benzyloxy)methyl)-2-(3-iodophenyl)-2-methoxytetrahydro-2H-pyran (**76**): Following the general synthetic procedure 2, **76** was prepared from **18** (10.00 g, 18.6 mmol) and 1,3-diiodide (9.20 g, 27.9 mmol) using *n*-BuLi (17.4 mL, 1.6 M in THF, 27.84 mmol), MeSO_3_H (7.50 g, 78.0 mmol), MeOH (38 mL), and dried THF (200 mL) as a crude sample. Brown oil, 16.23 g. This sample was used directly in the next step without further purification or characterization.

Synthesis of (2*R*,3*R*,4*R*,5*R*,6*S*)-3,4,5-tris(benzyloxy)-2-((benzyloxy)methyl)-6-(3-iodophenyl)tetrahydro-2H-pyran (**77**): Following the general synthetic procedure 3, **77** was prepared from crude **76** prepared above (16.23 g, deemed to be 21.5 mmol) using Et_3_SiH (5.00 g, 43.0 mmol) and BF_3_·Et_2_O (3.66 g, 25.8 mmol) in dried CH_2_Cl_2_ (163 mL). Column chromatography purification (EtOAc/PE = 1/6 by *v*/*v*) gave **77**. Pale yellow oil, 2.97 g (22% overall for **18** to **77**). ^1^H NMR (500 MHz, CDCl_3_) *δ* 7.75 (s, 1H), 7.61 (d, *J* = 8.0 Hz, 1H), 7.38–7.28 (m, 14H), 7.24–7.17 (m, 5H), 7.02 (t, *J* = 7.8 Hz, 1H), 6.95–6.93 (m, 2H), 4.91 (d, *J* = 10.5 Hz, 1H), 4.74–4.69 (m, 3H), 4.58 (dd, *J* = 25.5, 11.5 Hz, 3H), 4.40 (s, 1H), 4.20 (d, *J* = 11.5 Hz, 1H), 3.99 (t, *J* = 9.8 Hz, 1H), 3.90–3.89 (m, 1H), 3.81 (d, *J* = 3.5 Hz, 2H), 3.77 (dd, *J* = 9.5, 3.0 Hz, 1H), 3.61–3.58 (m, 1H). ^13^C NMR (126 MHz, CDCl_3_) *δ* 141.42, 138.56, 138.43, 138.39, 138.01, 136.47, 135.82, 129.77, 128.58, 128.47, 128.21, 128.18, 127.93, 127.81, 127.78, 127.61, 127.59, 127.54, 125.84, 94.17, 84.93, 80.00, 79.09, 75.38, 75.02, 74.58, 73.50, 72.41, 69.61. HR-MS (ESI): *m*/*z* [M+NH_4_]^+^ calcd for C_40_H_43_INO_5_^+^: 744.2180, found: 744.2186.

Synthesis of (2*R*,3*R*,4*R*,5*R*,6*S*)-2-(acetoxymethyl)-6-(3-iodophenyl)tetrahydro-2H-pyran-3,4,5-triyl triacetate (**78**): Following the procedure for the synthesis of **37** from **36**, **78** was prepared from **77** (2.50 g, 3.44 mmol) using Ac_2_O (25 mL) and BF_3_·Et_2_O (25 mL). Column chromatography purification (EtOAc/PE = 1/5 by *v*/*v*) gave **78**. Yellow oil, 1.23 g (67%). ^1^H NMR (500 MHz, CDCl_3_) *δ* 7.73–7.72 (m, 1H), 7.62 (d, *J* = 8.0 Hz, 1H), 7.26–7.24 (m, 1H), 7.05 (t, *J* = 7.8 Hz, 1H), 5.51–5.50 (m, 1H), 5.35–5.31 (m, 1H), 5.22 (dd, *J* = 10.3, 3.3 Hz, 1H), 4.71 (s, 1H), 4.33 (dd, *J* = 12.3, 5.8 Hz, 1H), 4.24 (dd, *J* = 12.5, 2.5 Hz, 1H), 3.82–3.79 (m, 1H), 2.11 (s, 3H), 2.08 (s, 3H), 1.99 (s, 3H), 1.96 (s, 3H). ^13^C NMR (126 MHz, CDCl_3_) *δ* 170.93, 170.39, 170.04, 169.90, 138.46, 137.37, 135.58, 130.07, 125.48, 94.17, 77.71, 76.66, 72.44, 70.50, 66.10, 63.09, 20.98, 20.89, 20.77, 20.58. HR-MS (ESI): *m*/*z* [M+NH_4_]^+^ calcd for C_20_H_27_INO_9_^+^: 552.0725, found: 552.0724.

Synthesis of (2*R*,3*R*,4*R*,5*R*,6*S*)-2-(acetoxymethyl)-6-(3-(4,4,5,5-tetramethyl-1,3,2-dioxaborolan-2-yl)phenyl)tetrahydro-2H-pyran-3,4,5-triyl triacetate (**79**): Following the general synthetic procedure 7, **79** was prepared from **78** (1.94 g, 3.63 mmol) using B_2_Pin_2_ (1.38 g, 5.45 mmol), KOAc (1.07 g, 10.9 mmol), and Pd(dppf)Cl_2_ (0.53 g, 0.726 mmol) in DMSO (19.4 mL). Column chromatography purification (EtOAc/PE = 1/1 by *v*/*v*) gave **79**. Colorless oil, 0.64 g (33%). ^1^H NMR (500 MHz, CDCl_3_) *δ* 7.73–7.70 (m, 2H), 7.46–7.43 (m, 1H), 7.33 (t, *J* = 7.5 Hz, 1H), 5.52–5.51 (m, 1H), 5.35 (t, *J* = 9.8 Hz, 1H), 5.23 (dd, *J* = 10.0, 3.5 Hz, 1H), 4.76 (s, 1H), 4.33 (dd, *J* = 12.0, 6.0 Hz, 1H), 4.25 (dd, *J* = 12.0, 2.5 Hz, 1H), 3.83–3.79 (m, 1H), 2.10 (s, 3H), 2.08 (s, 3H), 1.98 (s, 3H), 1.94 (s, 3H), 1.34 (s, 1H), 1.33 (s, 1H). ^13^C NMR (151 MHz, CDCl_3_) *δ* 170.96, 170.38, 170.09, 169.97, 135.59, 134.73, 132.84, 129.39, 128.35, 127.75, 84.02, 78.74, 76.65, 72.69, 70.87, 66.37, 63.27, 25.09, 24.93, 20.97, 20.91, 20.80, 20.61. HR-MS (ESI): *m*/*z* [M+H]^+^ calcd for C_26_H_36_BO_11_^+^: 535.2345, found: 535.2346.

Synthesis of (3-((2*S*,3*R*,4*R*,5*R*,6*R*)-3,4,5-triacetoxy-6-(acetoxymethyl)tetrahydro-2H-pyran-2-yl)phenyl)boronic acid (**80**): Following the general synthetic procedure 8, **80** was prepared from **74** (1.36 g, 2.16 mmol) using NaIO_4_ (1.38 g, 6.37 mmol) and NH_4_OAc (0.30 g, 3.82 mmol) in a mixture of acetone (10.9 mL) and H_2_O (2.7 mL). Column chromatography purification (MeOH/CH_2_Cl_2_ = 1/10 by *v*/*v*) gave **80**. Colorless oil, 1.26 g (68%). ^1^H NMR (500 MHz, DMSO-*d*_6_) *δ* 8.04 (s, 2H), 7.73–7.69 (m, 2H), 7.71–7.69 (m, 1H), 7.35–7.33 (m, 1H), 7.29 (t, *J* = 7.5 Hz, 1H), 5.40–5.36 (m, 2H), 5.14 (t, *J* = 10.0 Hz, 1H), 5.10 (s, 1H),4.20–4.17 (m, 1H), 4.15–4.12 (m, 1H), 4.05–4.02 (m, 1H), 2.06 (s, 3H), 2.03 (s, 3H), 1.92 (s, 3H), 1.88 (s, 3H). ^13^C NMR (151 MHz, DMSO-*d*_6_) *δ* 170.17, 169.73, 169.61, 169.46, 135.75, 133.80, 133.59, 132.16, 128.04, 127.02, 77.08, 75.04, 71.65, 70.82, 65.98, 62.71, 20.61, 20.54, 20.39, 20.18. HR-MS (ESI): *m*/*z* [M+H]^+^ calcd for C_20_H_26_BO_11_^+^: 453.1563, found: 453.1566.

Synthesis of (3-((2*S*,3*S*,4*R*,5*S*,6*R*)-3,4,5-trihydroxy-6-(hydroxymethyl)tetrahydro-2H-pyran-2-yl)phenyl)boronic acid (**E7**): Following the general synthetic procedure 5, **E7** was prepared from **80** (0.21 g, 0.465 mmol) using Na (30 mg, 1.30 mmol) in dried MeOH (4.2 mL). Column chromatography purification (MeOH/CH_2_Cl_2_ = 1/10 by *v*/*v*) gave **E7**. White solid. 44 mg (36%). m.p. 105.4–110.3 °C. [α]_D_^20^ = +24.6° (*c* = 2.8, H_2_O). ^1^H NMR (500 MHz, D_2_O) *δ* 7.80 (s, 1H), 7.73 (d, *J* = 7.5 Hz, 1H), 7.58 (d, *J* = 8.0 Hz, 1H), 7.49 (t, *J* = 7.5 Hz, 1H), 4.81 (s, 1H), 4.13 (d, *J* = 3.5 Hz, 1H), 4.01–3.98 (m, 1H), 3.90–3.84 (m, 2H), 3.71 (t, *J* = 9.8 Hz, 1H), 3.57–3.53 (m, 1H). ^13^C NMR (151 MHz, D_2_O) *δ* 137.59, 132.86, 131.98, 131.17, 128.69, 127.94, 80.06, 79.07, 74.30, 72.53, 66.92, 61.26. HR-MS (ESI): *m*/*z* [M+NH_4_]^+^ calcd for C_12_H_21_BNO_7_^+^: 302.1406, found: 302.1408.

Synthesis of (2*R*,3*S*,4*R*,5*S*,6*S*)-2-(hydroxymethyl)-6-(3-(4,4,5,5-tetramethyl-1,3,2-dioxaborolan-2-yl)phenyl)tetrahydro-2H-pyran-3,4,5-triol (**E8**): Following the general synthetic procedure 5, **E8** was prepared from **79** (0.50 g, 1.11 mmol) using Na (50 mg, 2.17 mmol) in dried MeOH (10 mL). Column chromatography purification (MeOH/CH_2_Cl_2_ = 1/10 by *v*/*v*) gave **E8**. White solid. 0.18 g (53%). m.p. 277.6–286.3 °C. [α]_D_^20^ = +11.8° (*c* = 11.8, DMSO). ^1^H NMR (500 MHz, DMSO-*d*_6_) *δ* 7.72 (s, 1H), 7.53 (d, *J* = 7.0 Hz, 1H), 7.48 (d, *J* = 8.0 Hz, 1H), 7.30 (t, *J* = 7.5 Hz, 1H), 4.78 (d, *J* = 5.5 Hz, 1H), 4.68 (d, *J* = 5.5 Hz, 1H), 4.48 (s, 1H), 4.45 (t, *J* = 5.8 Hz, 1H), 4.19 (d, *J* = 5.5 Hz, 1H), 3.79–3.76 (m, 1H), 3.73–3.71 (m, 1H), 3.54–3.48 (m, 2H), 3.43–3.39 (m, 1H), 3.21–3.18 (m, 1H), 1.30 (s, 12H). ^13^C NMR (151 MHz, DMSO-*d*_6_) *δ* 139.67, 133.08, 132.82, 132.42, 129.97, 126.95, 83.58, 81.65, 79.20, 75.11, 72.28, 67.17, 61.69, 24.74, 24.71. HR-MS (ESI): *m*/*z* [M+NH_4_]^+^ calcd for C_18_H_31_BNO_7_^+^: 384.2188, found: 384.2190.

Synthesis of (2*R*,3*R*,4*S*,5*S*,6*S*)-2-(acetoxymethyl)-5-fluoro-6-(4-iodophenyl)tetrahydro-2H-pyran-3,4-diyl diacetate (**81**): Following the procedure for the synthesis of **37** from **36**, **81** was prepared from **26** (0.40 g, 0.627 mmol) using Ac_2_O (4 mL) and BF_3_·Et_2_O (4 mL). Column chromatography purification (EtOAc/PE = 1/6 by *v*/*v*) gave **81**. Colorless oil, 0.25 g (80%). ^1^H NMR (500 MHz, CDCl_3_) *δ* 7.74–7.71 (m, 2H), 7.14–7.13 (m, 2H), 5.45–5.40 (m, 1H), 5.14 (t, *J* = 9.8 Hz, 1H), 4.42–4.41 (m, 1H), 4.40–4.30 (m, 1H), 4.30–4.27 (m, 1H), 4.16 (dd, *J* = 12.3, 2.3 Hz, 1H), 3.86–3.83 (m, 1H), 2.08 (s, 6H), 2.06 (s, 3H). ^13^C NMR (126 MHz, CDCl_3_) *δ* 170.79, 170.28, 169.78, 137.83, 136.20, 128.85, 90.93 (d, *J* = 189.6 Hz), 78.82 (d, *J* = 22.6 Hz), 74.16 (d, *J* = 19.8 Hz), 68.45 (d, *J* = 7. 6 Hz), 20.90, 20.85, 20.75. HR-MS (ESI): *m*/*z* [M+NH_4_]^+^ calcd for C_18_H_24_FINO_7_^+^: 512.0576, found: 512.0583.

Synthesis of (2*R*,3*R*,4*S*,5*S*,6*S*)-2-(acetoxymethyl)-5-fluoro-6-(4-(4,4,5,5-tetramethyl-1,3,2-dioxaborolan-2-yl)phenyl)tetrahydro-2H-pyran-3,4-diyl diacetate (**82**): Following the general synthetic procedure 7, **81** was prepared from **81** (0.24 g, 0.486 mmol) using B_2_Pin_2_ (0.19 g, 0.729 mmol), KOAc (0.14 g, 1.458 mmol) and Pd(dppf)Cl_2_ (0.07 g, 0.097 mmol) in dried DMSO (2.4 mL). Column chromatography purification (EtOAc/PE = 1/2 by *v*/*v*) gave **82**. Colorless oil, 0.82 g (34%). ^1^H NMR (500 MHz, CDCl_3_) *δ* 7.84–7.82 (m, 2H), 7.40–7.38 (m, 2H), 5.48–5.43 (m, 1H), 5.17–5.13 (m, 1H), 4.48–4.47 (m, 1H), 4.46–4.36 (m, 1H), 4.30 (dd, *J* = 12.3, 5.3 Hz, 1H), 4.16 (dd, *J* = 12.5, 2.5 Hz, 1H), 3.88–3.84 (m, 1H), 2.08 (s, 6H), 2.07 (s, 3H), 1.34 (s, 12H). ^13^C NMR (201 MHz, CDCl_3_) *δ* 170.85, 170.33, 169.82, 139.37, 135.12, 128.73, 126.38, 90.95 (d, *J* = 188.9 Hz), 84.04, 79.51, 79.40, 76.20, 74.36 (d, *J* = 19.7 Hz), 68.61 (d, *J* = 7.4 Hz), 62.47, 25.03, 24.96, 20.91, 20.88, 20.78. HR-MS (ESI): *m*/*z* [M+NH_4_]^+^ calcd for C_24_H_36_BFNO_9_^+^: 512.2462, found: 512.2469.

Synthesis of (2*R*,3*S*,4*S*,5*R*,6*S*)-5-fluoro-2-(hydroxymethyl)-6-(4-(4,4,5,5-tetramethyl-1,3,2-dioxaborolan-2-yl)phenyl)tetrahydro-2H-pyran-3,4-diol (**E9**): Following the general synthetic procedure 5, **E9** was prepared from **82** (0.30 g, 0.607 mmol) using Na (50 mg, 2.17 mmol) in dried MeOH (3 mL). Column chromatography purification (MeOH/CH_2_Cl_2_ = 1/10 by *v*/*v*) gave **E9**. White solid, 34 mg (15%). m.p. 145.8–153.7 °C. [α]_D_^20^ = +9.5° (*c* = 5.8, DMSO). ^1^H NMR (500 MHz, DMSO-*d*_6_) *δ* 7.67 (d, *J* = 8.0 Hz, 2H), 7.41 (d, *J* = 8.0 Hz, 2H), 5.49–5.48 (m, 1H), 5.26–5.25 (m, 1H), 4.56 (t, *J* = 6.0 Hz, 1H), 4.38 (dd, *J* = 9.5, 3.0 Hz, 1H), 4.14–4.00 (m, 1H), 3.74–3.70 (m, 1H), 3.62–3.58 (m, 1H), 3.57–3.49 (m, 1H), 3.48–3.44 (m, 1H), 3.28–3.25 (m, 1H), 1.29 (s, 12H). ^19^F NMR (471 MHz, DMSO-*d*_6_) *δ* −193.80. HR-MS (ESI): *m*/*z* [M+H]^+^ calcd for C_18_H_27_BFO_6_^+^: 369.1879, found: 369.1880.

Synthesis of (3*R*,4*S*,5*R*,6*R*)-3,4,5-tris(benzyloxy)-6-((benzyloxy)methyl)-2-(3,5-dibromophenyl)-2-methoxytetrahydro-2H-pyran (**83**): Following the general synthetic procedure 2, **83** was prepared from **6** (10.00 g, 18.6 mmol) and 1,3,5-tribromobenzene (14.6 g, 46.4 mmol) using *n*-BuLi (23.2 mL, 1.6 M in THF, 37.12 mmol), MeSO_3_H (8.94 g, 0.093 mol), MeOH (45 mL), and dried THF (200 mL) as a crude sample. Brown oil, 18.33 g. This sample was used directly in the next step without further purification or characterization.

Synthesis of (2*R*,3*R*,4*R*,5*S*,6*S*)-3,4,5-tris(benzyloxy)-2-((benzyloxy)methyl)-6-(3,5-dibromophenyl)tetrahydro-2H-pyran (**84**): Following the general synthetic procedure 3, **84** was prepared from crude **83** prepared above (18.33 g, deemed to be 23.2 mmol) using Et_3_SiH (5.40 g, 46.4 mmol) and BF_3_·Et_2_O (3.95 g, 27.8 mmol) in dried CH_2_Cl_2_ (183 mL). Column chromatography purification (EtOAc/PE = 1/5 by *v*/*v*) gave **84**. Colorless oil, 3.10 g. This sample was used directly in the next step without characterization.

Synthesis of (2*R*,3*R*,4*R*,5*S*,6*S*)-2-(acetoxymethyl)-6-(3,5-dibromophenyl)tetrahydro-2H-pyran-3,4,5-triyl triacetate (**85**): Following the procedure for the synthesis of **37** from **36**, **85** was prepared from **84** (3.10 g, 4.07 mmol) using Ac_2_O (31 mL) and BF_3_·Et_2_O (31 mL). Column chromatography purification (EtOAc/PE = 1/3 by *v*/*v*) gave **85**. Yellow oil, 1.41 g (11% overall for **6** to **85**). ^1^H NMR (500 MHz, CDCl_3_) *δ* 7.63–7.62 (m, 1H), 7.43 (d, *J* = 1.5 Hz, 2H), 5.31–5.29 (m, 1H), 5.20 (t, *J* = 9.8 Hz, 1H), 4.99 (t, *J* = 9.8 Hz, 1H), 4.34 (d, *J* = 9.5 Hz, 1H), 4.29 (dd, *J* = 12.5, 5.0 Hz, 1H), 4.17 (dd, *J* = 12.5, 2.5 Hz, 1H), 3.84–3.81 (m, 1H), 2.11 (s, 3H), 2.06 (s, 3H), 2.00 (s, 3H), 1.91 (s, 3H). ^13^C NMR (151 MHz, CDCl_3_) *δ* 170.86, 170.46, 169.58, 168.96, 140.30, 134.58, 128.88, 123.06, 78.70, 76.45, 74.00, 72.69, 68.47, 62.34, 20.93, 20.77, 20.75, 20.57. HR-MS (ESI): *m*/*z* [M+Na]^+^ calcd for C_20_H_22_Br_2_NaO_9_^+^: 586.9523, found: 586.9535.

Synthesis of (2*R*,3*R*,4*R*,5*S*,6*S*)-2-(acetoxymethyl)-6-(3,5-bis(4,4,5,5-tetramethyl-1,3,2-dioxaborolan-2-yl)phenyl)tetrahydro-2H-pyran-3,4,5-triyl triacetate (**86**): Following the general synthetic procedure 7, **86** was prepared from **85** (0.78 g, 1.38 mmol) using B_2_Pin_2_ (1.05 g, 4.14 mmol), KOAc (0.82 g, 8.28 mmol), and Pd(dppf)Cl_2_ (0.40 g, 0.552 mmol) in dried DMSO (7.8 mL). Column chromatography purification (EtOAc/PE = 1/2 by *v*/*v*) gave **86**. Colorless oil, 0.37 g (41%). ^1^H NMR (500 MHz, CDCl_3_) *δ* 8.22 (s, 1H), 7.88–7.86 (m, 2H), 5.31 (d, *J* = 9.5 Hz, 1H), 5.23 (t, *J* = 9.5 Hz, 1H), 5.14 (t, *J* = 9.8 Hz, 1H), 4.44 (d, *J* = 10.0 Hz, 1H), 4.27 (dd, *J* = 12.5, 5.0 Hz, 1H), 4.20 (dd, *J* = 12.3, 2.3 Hz, 1H), 3.84–3.80 (m, 1H), 2.10 (s, 3H), 2.06 (s, 3H), 1.99 (s, 3H), 1.80 (s, 3H), 1.34 (s, 12H), 1.33 (s, 12H) ^13^C NMR (151 MHz, CDCl_3_) *δ* 171.02, 170.61, 169.67, 169.08, 141.93, 136.39, 134.96, 84.01, 80.37, 76.38, 74.58, 72.91, 68.90, 62.68, 25.04, 25.02, 20.97, 20.83, 20.80, 20.51. HR-MS (ESI): *m*/*z* [M+H]^+^ calcd for C_32_H_47_B_2_O_13_^+^: 661.3197, found: 661.3214.

Synthesis of (5-((2*S*,3*S*,4*R*,5*R*,6*R*)-3,4,5-triacetoxy-6-(acetoxymethyl)tetrahydro-2H-pyran-2-yl)-1,3-phenylene)diboronic acid (**87**): Following the general synthetic procedure 8, **87** was prepared from **86** (0.40 g, 0.806 mmol) using NaIO_4_ (0.86 g, 4.03 mmol) and NH_4_OAc (0.19 g, 2.42 mmol) in a mixture of acetone (3.2 mL) and H_2_O (0.80 mL). Column chromatography purification (MeOH/CH_2_Cl_2_ = 1/10 by *v*/*v*) gave **87**. Colorless oil, 0.16 g. This sample was used directly in the next step without characterization.

Synthesis of (5-((2*S*,3*R*,4*R*,5*S*,6*R*)-3,4,5-trihydroxy-6-(hydroxymethyl)tetrahydro-2H-pyran-2-yl)-1,3-phenylene)diboronic acid (**F1**): Following the general synthetic procedure 5, **F1** was prepared from **87** (0.16 g, 0.323 mmol) using Na (30 mg, 1.74 mmol) in dried MeOH (3.2 mL). Column chromatography purification (MeOH/CH_2_Cl_2_ = 1/10 by *v*/*v*) gave **F1**. White solid, 34 mg (17% overall for **86** to **F1**). m.p. 362.2–378.9 °C. [α]_D_^20^ = +5.3° (*c* = 4.0, H_2_O). ^1^H NMR (500 MHz, D_2_O) δ 8.12 (s, 1H), 7.90 (s, 2H), 4.36–4.35 (m, 1H), 3.91–3.88 (m, 1H), 3.80–3.75 (m, 2H), 3.65–3.60 (m, 3H). ^13^C NMR (151 MHz, D_2_O) *δ* 139.21, 136.63, 135.53, 81.84, 79.92, 77.25, 74.06, 69.71, 60.82. HR-MS (ESI): *m*/*z* [M+Na]^+^ calcd for C_12_H_18_B_2_NaO_9_^+^: 351.1029, found: 351.1028.

Synthesis of (2*S*,3*R*,4*R*,5*S*,6*R*)-2-(3,5-bis(4,4,5,5-tetramethyl-1,3,2-dioxaborolan-2-yl)phenyl)-6-(hydroxymethyl)tetrahydro-2H-pyran-3,4,5-triol (**F2**): Following the general synthetic procedure 5, **F2** was prepared from **86** (0.40 g, 0.606 mmol) using Na (50 mg, 2.17 mmol) in dried MeOH (8 mL). Column chromatography purification (MeOH/CH_2_Cl_2_ = 1/10 by *v*/*v*) gave **F2**. White solid, 0.15 g (49%). m.p. 175.7–181.3 °C. [α]_D_^20^ = +2.4° (*c* = 14.0, MeOH). ^1^H NMR (500 MHz, DMSO-*d*_6_) *δ* 7.96–7.95 (m, 1H), 7.73–7.72 (m, 2H), 4.94–4.93 (m, 1H), 4.91–4.90 (m, 1H), 4.77–4.75 (m, 1H), 4.49 (t, *J* = 5.5 Hz, 1H), 4.04 (d, *J* = 9.5 Hz, 1H), 3.71–3.68 (m, 1H), 3.48–3.46 (m, 1H), 3.27–3.20 (m, 3H), 3.14–3.11 (m, 1H), 1.30 (s, 24H). ^13^C NMR (151 MHz, DMSO-*d*_6_) *δ* 140.20, 139.23, 136.82, 127.14, 83.72, 81.53, 81.39, 78.47, 70.47, 67.30, 61.30, 24.75, 24.63. HR-MS (ESI): *m*/*z* [M-H]^−^ calcd for C_24_H_39_B_2_O_9_^−^: 493.2786, found: 493.2773.

Synthesis of (3*S*,4*S*,5*R*,6*R*)-3,4,5-tris(benzyloxy)-6-((benzyloxy)methyl)-2-(3,5-dibromophenyl)-2-methoxytetrahydro-2H-pyran (**88**): Following the general synthetic procedure 2, **88** was prepared from **18** (10.00 g, 18.6 mmol) and 1,3,5-tribromobenzene (8.78 g, 27.9 mmol) using *n*-BuLi (14 mL, 1.6 M in THF, 22.4 mmol), MeSO_3_H (8.94 g, 93 mmol), MeOH (45 mL), and dried THF (200 mL) as a crude sample. Brown oil, 15.65 g. This sample was used directly in the next step without further purification or characterization.

Synthesis of (2*R*,3*R*,4*R*,5*R*,6*S*)-3,4,5-tris(benzyloxy)-2-((benzyloxy)methyl)-6-(3,5-dibromophenyl)tetrahydro-2H-pyran (**89**): Following the general synthetic procedure 3, **89** was prepared from crude **88** prepared above (15.65 g, deemed to be 19.9 mmol) using Et_3_SiH (4.63 g, 39.8 mmol) and BF_3_·Et_2_O (3.39 g, 23.9 mmol) in dried CH_2_Cl_2_ (157 mL). Column chromatography purification (EtOAc/PE = 1/5 by *v*/*v*) gave **89**. Colorless oil, 2.25 g. This sample was used directly in the next step without characterization.

Synthesis of (2*R*,3*R*,4*R*,5R,6*S*)-2-(acetoxymethyl)-6-(3,5-dibromophenyl)tetrahydro-2H-pyran-3,4,5-triyl triacetate (**90**): Following the procedure for the synthesis of **37** from **36**, **90** was prepared from **89** (2.25 g, 2.97 mmol) using Ac_2_O (23 mL) and BF_3_·Et_2_O (23 mL). Column chromatography purification (EtOAc/PE = 1/3 by *v*/*v*) gave **90**. Brown oil, 1.08 g (11% overall for **18** to **90**). ^1^H NMR (500 MHz, CDCl_3_) *δ* 7.59–7.57 (m, 1H), 7.45–7.42 (s, 2H), 5.48–5.47 (m, 1H), 5.31–5.29 (m, 1H), 5.19 (dd, *J* = 10.0, 3.5 Hz, 1H), 4.69 (s, 1H), 4.31 (dd, *J* = 12.3, 5.8 Hz, 1H), 4.23–4.20 (m, *m*, 1H), 3.81–3.78 (m, 1H), 2.11 (s, 3H), 2.07 (s, 3H), 1.98 (s, 6H). ^13^C NMR (126 MHz, CDCl_3_) *δ* 170.85, 170.30, 169.91, 169.82, 140.05, 133.98, 128.39, 122.89, 77.24, 76.72, 72.28, 70.28, 65.88, 62.98, 20.94, 20.85, 20.73, 20.53. HR-MS (ESI): *m*/*z* [M+H]^+^ calcd for C_20_H_23_Br_2_O_9_^+^: 564.9703, found: 564.9712.

Synthesis of (2*R*,3*R*,4*R*,5*R*,6*S*)-2-(acetoxymethyl)-6-(3,5-bis(4,4,5,5-tetramethyl-1,3,2-dioxaborolan-2-yl)phenyl)tetrahydro-2H-pyran-3,4,5-triyl triacetate (**91**): Following the general synthetic procedure 7, **91** was prepared from **90** (0.85 g, 1.50 mmol) using B_2_Pin_2_ (1.14 g, 4.50 mmol), KOAc (0.88 g, 9.00 mmol), and Pd(dppf)Cl_2_ (0.44 g, 0.60 mmol) in dried DMSO (8.5 mL). Column chromatography purification (EtOAc/PE = 1/2 by *v*/*v*) gave **91**. Colorless oil, 0.39 g (39%). ^1^H NMR (500 MHz, CDCl_3_) *δ* 8.19 (s, 1H), 7.86–7.85 (m, 2H), 5.45–5.44 (m, 1H), 5.35 (t, *J* = 10.0, 1H), 5.22 (dd, *J* = 10.0, 3.5 Hz, 1H), 4.74 (s, 1H), 4.29–4.28 (m, 1H), 3.82–3.79 (m, 1H), 3.74–3.70 (m, 1H), 2.10 (s, 3H), 2.08 (s, 3H), 1.98 (s, 6H), 1.34 (s, 12H), 1.32 (s, 12H). ^13^C NMR (201 MHz, CDCl_3_) *δ* 171.00, 170.36, 170.10, 170.00, 141.41, 136.11, 134.83, 83.97, 79.11, 76.78, 72.69, 70.94, 66.48, 63.43, 25.12, 24.91, 20.99, 20.92, 20.81, 20.58. HR-MS (ESI): *m*/*z* [M+H]^+^ calcd for C_32_H_47_B_2_O_13_^+^: 661.3197, found: 661.3211.

Synthesis of (5-((2*S*,3*R*,4*R*,5*R*,6*R*)-3,4,5-triacetoxy-6-(acetoxymethyl)tetrahydro-2H-pyran-2-yl)-1,3-phenylene)diboronic acid (**92**): Following the general synthetic procedure 8, **92** was prepared from **91** (0.48 g, 0.727 mmol) using NaIO_4_ (0.78 g, 3.64 mmol) and NH_4_OAc (0.17 g, 2.18 mmol) in a mixture of acetone (3.8 mL) and H_2_O (1 mL). Column chromatography purification (MeOH/CH_2_Cl_2_ = 1/10 by *v*/*v*) gave **92**. Colorless oil, 0.18 g. This sample was used directly in the next step without characterization.

Synthesis of (5-((2*S*,3*S*,4*R*,5*S*,6*R*)-3,4,5-trihydroxy-6-(hydroxymethyl)tetrahydro-2H-pyran-2-yl)-1,3-phenylene)diboronic acid (**F3**): Following the general synthetic procedure 5, **F3** was prepared from **92** (0.18 g, 0.363 mmol) using Na (30 mg, 1.74 mmol) in MeOH (3.2 mL). Column chromatography purification (MeOH/CH_2_Cl_2_ = 1/10 by *v*/*v*) gave **F3**. White solid, 26 mg (11% overall for **91** to **F3**). m.p. 348.9–350.7 °C. [α]_D_^20^ = +12.8° (*c* = 3.6, H_2_O). ^1^H NMR (500 MHz, D_2_O) *δ* 8.06 (s, 1H), 7.90 (s, 2H), 4.96 (s, 1H), 4.15–4.14 (m, 1H), 4.01–3.99 (m, 1H), 3.90–3.84 (m, 2H), 3.71 (t, *J* = 9.8 Hz, 1H), 3.57–3.53 (m, 1H). ^13^C NMR (151 MHz, D_2_O) *δ* 138.01, 137.01, 133.66, 80.03, 78.97, 74.31, 72.44, 66.93, 61.31. HR-MS (ESI): *m*/*z* [M+NH_4_]^+^ calcd for C_12_H_22_B_2_NO_9_^+^: 346.1475, found: 346.1474.

Synthesis of (2*S*,3*S*,4*R*,5*S*,6*R*)-2-(3,5-bis(4,4,5,5-tetramethyl-1,3,2-dioxaborolan-2-yl)phenyl)-6-(hydroxymethyl)tetrahydro-2H-pyran-3,4,5-triol (**F4**): Following the general synthetic procedure 5, **F4** was prepared from **91** (0.14 g, 0.212 mmol) using Na (30 mg, 1.74 mmol) in dried MeOH (2.8 mL). Column chromatography purification (MeOH/CH_2_Cl_2_ = 1/10 by *v*/*v*) gave **F4**. White solid, 43 mg (41%). m.p. 157.8–166.1 °C. [α]_D_^20^ = +1.9° (*c* = 10.6, MeOH). ^1^H NMR (500 MHz, DMSO-*d*_6_) *δ* 7.92 (s, 1H), 7.79–7.78 (m, 2H), 4.77 (d, *J* = 5.5 Hz, 1H), 4.66 (d, *J* = 5.5 Hz, 1H), 4.52 (s, 1H), 4.48 (t, *J* = 5.5 Hz, 1H), 4.21 (d, *J* = 5.5 Hz, 1H), 3.80–3.76 (m, 1H), 3.69–3.68 (m, 1H), 3.52–3.49 (m, 2H), 3.42–3.38 (m, 1H), 3.21–3.17 (m, 1H), 1.30 (s, 24H). ^13^C NMR (151 MHz, DMSO-*d*_6_) *δ* 139.63, 139.19, 135.93, 126.76, 83.67, 81.76, 79.19, 75.00, 72.36, 67.16, 61.64, 24.73, 24.69. HR-MS (ESI): *m*/*z* [M+NH_4_]^+^ calcd for C_24_H_42_B_2_NO_9_^+^: 510.3140, found: 510.3043.

### 3.2. Cytotoxicity Assay

Cell culture: SCC-9 human tongue squamous cell carcinoma cells were cultured in Dulbecco’s modified eagle medium (DMEM)/Ham’s F-12 nutrient mixture (1/1) medium supplemented with 10% fetal bovine serum (FBS), 0.5 mM sodium pyruvate, and 400 ng/mL of hydrocortisone. HaCaT immortalized human keratinocytes were maintained in DMEM medium containing 10% FBS. All cells were maintained at 37 °C with 5% CO_2_.

Cell viability assay: Cells were seeded in 96-well plates at 5 × 10^3^ cells per well (SCC-9) and 6.5 × 10^3^ cells per well (HaCaT) and treated with the indicated concentrations of compounds. After 24 h, cell viability was assayed using the CCK-8 reagent according to the manufacturer’s instructions. Absorbance was measured at 450 nm using a microplate reader. The IC_50_ values were calculated through nonlinear regression analysis using a four-parameter logistic model.

### 3.3. Cellular Boron Uptake Assay

Determination of intracellular boron content by ICP-MS: SCC-9 and HaCaT cells were seeded in 6-well plates and incubated overnight. Cells were subsequently treated with the indicated concentrations of the test compounds for 3 h. After treatment, cells were gathered and washed with ice-cold PBS and then digested with HNO_3_ until complete clarification was achieved. The digested samples were then diluted with ultrapure water to the appropriate volume for analysis. Boron concentrations were quantified using ICP-MS (Agilent 7850, Agilent Technologies, Singapore). Quantification was performed based on an external standard calibration curve.

## 4. Conclusions

Based on the strategy of glucose transporter 1 (GLUT1), we designed and synthesized a total of two categories comprising 6 series (28 boron-bearing sugars in total). Their boron-carrying abilities were determined by boron uptake by the SCC-9 and HaCaT cell lines, and their T/N values were calculated. Among the target compounds, almost no boron uptake was observed for series E–F (category II); higher boron uptake and larger T/N values were observed for series A–C (category I) compared with BPA. Notably, compound **B3** exhibited the best profile, with 10.6-fold higher boron uptake by the SCC-9 cell line and a largely improved T/N (3.3 for **B3** vs. 1.4 for BPA) compared with BPA. The chemical structure of **B3** represents a privileged candidate structure for the future design of “ideal” boron carriers for BNCT. Considering that the T/N of **B3** still falls short of that for an “ideal” boron carrier (T/N = 3.3 for **B3** vs. T/N > 5 for “ideal” boron carriers), further SAR exploration based on **B3** will be conducted in the future.

## Figures and Tables

**Table 1:** Cytotoxicity of target compounds and BPA against SCC-9 and HaCaT cell lines.

Compd	IC_50_ (mM)		Compd	IC_50_ (mM)	
	SCC-9	HaCaT		SCC-9	HaCaT
**A1**	0.71	0.60	**E1**	>1	>1
**A2**	0.64	0.61	**E2**	>1	>1
**A3**	0.56	0.46	**E3**	>1	>1
**B1**	0.52	0.52	**E4**	>1	>1
**B2**	0.50	0.52	**E5**	>1	>1
**B3**	>0.2	>0.2	**E6**	>1	>1
**B4**	0.25	0.38	**E7**	>1	>1
**C1**	0.50	0.53	**E8**	>1	>1
**C2**	0.51	0.53	**E9**	>1	>1
**D1**	0.30	0.26	**F1**	>1	>1
**D2**	0.22	0.21	**F2**	>1	>1
**D3**	0.35	0.31	**F3**	>1	>1
**D4**	0.63	0.49	**F4**	>1	>1
**D5**	0.31	0.28	BPA	>1	>1
**D6**	0.40	0.34			

**Table 2:** Summary of boron uptake by SCC-9 and HaCaT cell lines for target compounds in series A–D.

Compd	B Uptake (μg/10^6^ cell)				Compd	B Uptake (μg/10^6^ cell)			
	SCC-9		HaCaT			SCC-9		HaCaT	
	0.1 mM	0.05 mM	0.1 mM	0.05 mM		0.1 mM	0.05 mM	0.1 mM	0.05 mM
**A1**	1.28	0.84	0.53	0.33	**C2**	0.91	0.67	0.55	0.29
**A2**	0.88	0.72	0.54	0.30	**D1**	0.13	0.1	0.09	0.05
**A3**	1.42	1.34	0.60	0.63	**D2**	0.17	0.07	0.07	0.04
**B1**	1.66	0.85	1.02	0.38	**D3**	0.25	0.23	0.15	0.16
**B2**	1.25	0.82	0.65	0.38	**D4**	0.21	0.21	0.22	0.10
**B3**	2.22	1.17	0.68	0.51	**D5**	0.23	0.23	0.10	0.06
**B4**	1.62	1.03	0.88	0.48	**D6**	0.21	0.13	0.13	0.08
**C1**	0.87	0.81	0.42	0.33					

**Table 3:** Summary of boron uptake by SCC-9 and HaCaT cell lines for target compounds in series E–F and BPA.

Compd	B Uptake (μg/10^6^ cell)					
	SCC-9			HaCaT		
	5 mM	2.5 mM	1 mM	5 mM	2.5 mM	1 mM
**E1**		0.08	0.01		0.01	0
**E2**		0	0		0	0
**E3**		0.01	0		0.01	0
**E4**		0	0		0.01	0
**E5**		0.06	0.02		0.02	0.01
**E6**		0.02	0.01		0.01	0
**E7**		0.06	0.03		0.07	0.03
**E8**		0.10	0.04		0.02	0.01
**E9**		0.09	0.02		0.05	0.01
**F1**		0	0		0	0
**F2**		0.02	0		0.01	0
**F3**		0.03	0		0	0
**F4**		0.05	0.01		0	0
BPA	0.21	0.07		0.15	0.05	

**Table 4:** Summary of T/N values for target compounds in series A–D.

Compd	T/N ^a^		Compd	T/N ^a^	
	0.1 mM	0.05 mM		0.1 mM	0.05 mM
**A1**	2.4	2.5	**C2**	1.7	2.3
**A2**	1.6	2.4	**D1**	1.4	2
**A3**	2.4	2.1	**D2**	2.4	1.8
**B1**	1.6	2.2	**D3**	1.7	1.4
**B2**	1.9	2.2	**D4**	1.0	2.1
**B3**	3.3	2.3	**D5**	2.3	3.8
**B4**	1.8	2.1	**D6**	1.6	1.6
**C1**	2.1	2.5			

^a^ T/N = boron uptake by SCC-9 cell (μg/10^6^ cell)/boron uptake by HaCaT cell (μg/10^6^ cell).

**Table 5:** Summary of T/N values for target compounds in series E–F and BPA.

Compd	T/N ^a^		Compd	T/N ^a^		
	2.5 mM	1 mM		5 mM	2.5 mM	1 mM
**E1**	8		**E8**		5	4
**E2**			**E9**		1.8	2
**E3**	1		**F1**			
**E4**			**F2**		2	
**E5**	3	2	**F3**			
**E6**	2		**F4**			
**E7**	0.86	1	BPA	1.4	1.50	

^a^ T/N = boron uptake by SCC-9 cell (μg/10^6^ cell)/boron uptake by HaCaT cell (μg/10^6^ cell).

## Data Availability

The data presented in this study are available upon request from the corresponding authors.

## Supplementary Materials

The following supporting information can be downloaded at https://www.mdpi.com/article/10.3390/molecules31081230/s1, the synthetic procedure for synthesized compounds, and copies of all of the ^1^H NMR, ^13^C NMR, ^19^F NMR, ^11^B NMR, and HR-MS spectra thereof.

## Abbreviations

The following abbreviations are used in this manuscript:

AIBN	2,2′-azobisisobutyronitrile
BNCT	boron neutron capture therapy
BPA	(L)-4-boronophenylalanine
BSH	sodium borocaptate
DMAP	4-dimethylaminopyridine
ESI	electrospray ionization
GLUT1	glucose transporter 1 (GLUT1)
HR-MS	high-resolution mass spectra
ICP-MS	inductively coupled plasma–mass spectrometry
NBS	*N*-bromosuccinimide
NMM	*N*-methylmorpholine
PCy_3_	tricyclohexylphosphine
PEG	poly ethylene glycol
SAR	structure-activity relationship
selectfluor	1-chloromethyl-4-fluoro-1,4-diazoniabicyclo [2.2.2]octane bis(tetrafluoroborate)
T/B	tumor/blood ratio
TBAF	tetra-*n*-butylammonium fluoride
TBAI	tetra-*n*-butylammonium iodide
TBDMSCl	*tert*-butyldimethylsilyl chloride
TLC	thin-layer chromatography
TMSCl	trimethylsilyl chloride
T/N	tumor/normal tissue ratio
