# Supplementary material for: Design, Synthesis, and Biological Activity of Boron-Bearing Sugar Derivatives for Boron Neutron Capture Therapy (BNCT)
Source: Molecules. 2026 Apr 8;31(8):1230. doi: 10.3390/molecules31081230 (PMC13118604; doi:10.3390/molecules31081230)
Supplement: Supplementary file 1 [file molecules-31-01230-s001.zip › molecules-4228752-supplementary.pdf]

Design, Synthesis, and Biological Activity of Boron-Bearing Sugar Derivatives for Boron Neutron Capture Therapy (BNCT)

Mengyan Hou [1,2,†], Xia Li [1,2,†], Yan Li [3,†], Wenhao Shi [2,†], Haotian Tang [2], Fang Feng [3], Xuan Wan [3], Hua Xie [1,2,3,*] and Guilong Zhao [1,2,3,*]

[1] School of Chinese Materia Medica, Nanjing University of Chinese Medicine, Nanjing 210023, China; houmengyan1062@zidd.ac.cn (M.H.); lixia1063@zidd.ac.cn (X.L.)

[2] Zhongshan Institute for Drug Discovery, Shanghai Institute of Materia Medica, Chinese Academy of Sciences, Zhongshan 528400, China; shiwenhao@zidd.ac.cn (W.S.); tanghaotian18@mails.ucas.ac.cn (H.T.)

[3] Shanghai Institute of Materia Medica, Chinese Academy of Sciences, Shanghai 201203, China; yanli@simm.ac.cn (Y.L.); fengfang@simm.ac.cn (F.F.); wanxuan@simm.ac.cn (X.W.)

* Correspondence: hxie@simm.ac.cn (H.X.); zhao_guilong@126.com (G.Z.)

† These authors contributed equally.

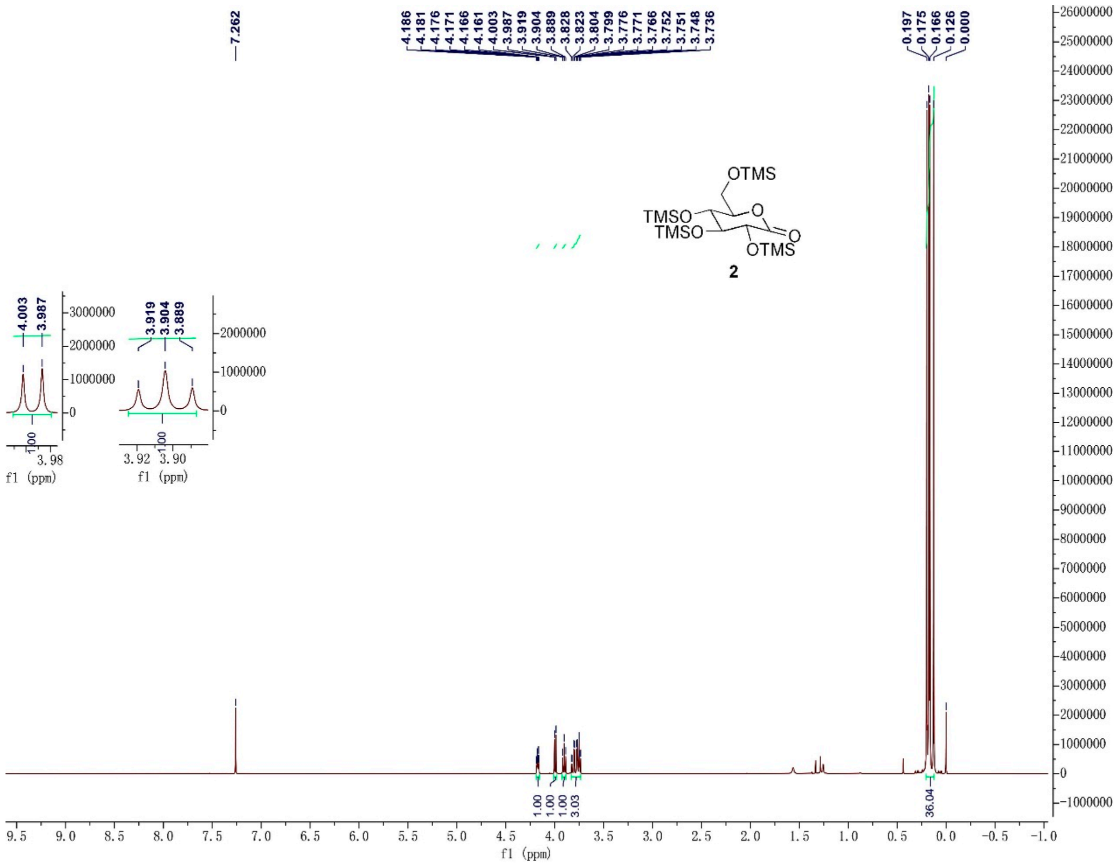

Figure S1 ^{1}H NMR spectrum of **2**

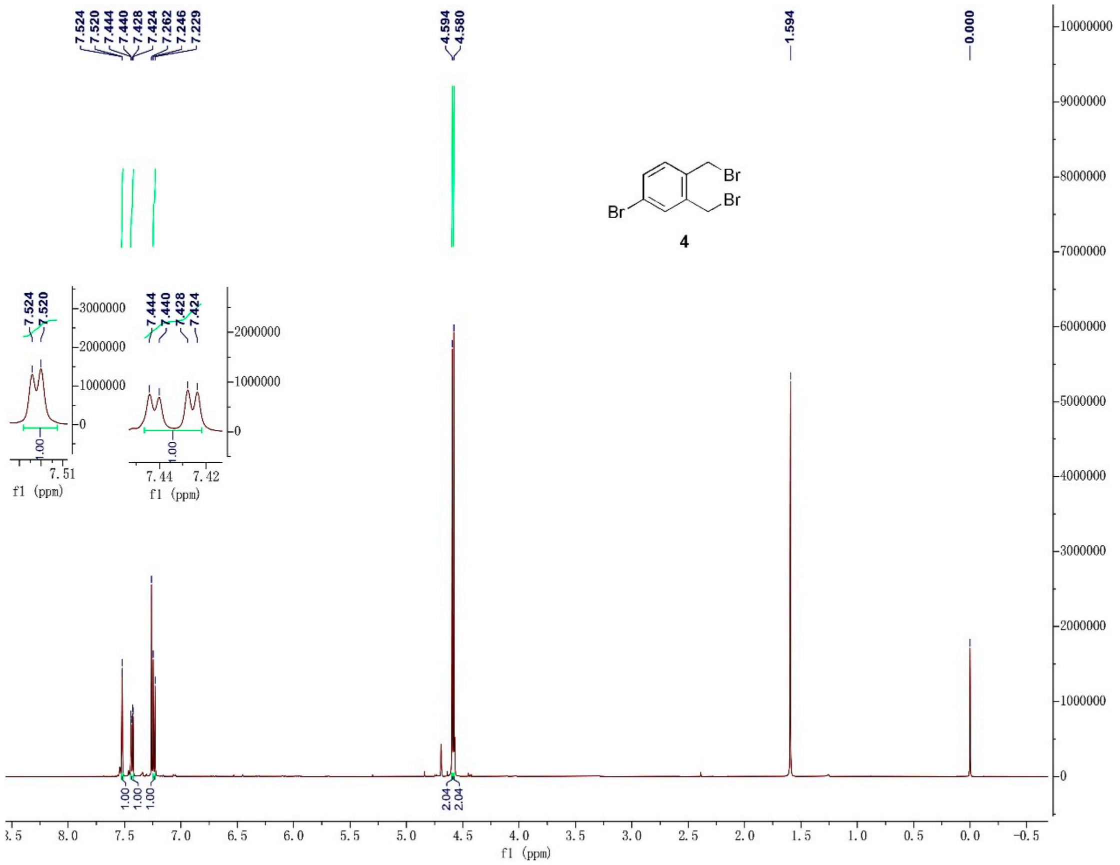

Figure S2 ^{1}H NMR spectrum of **4**

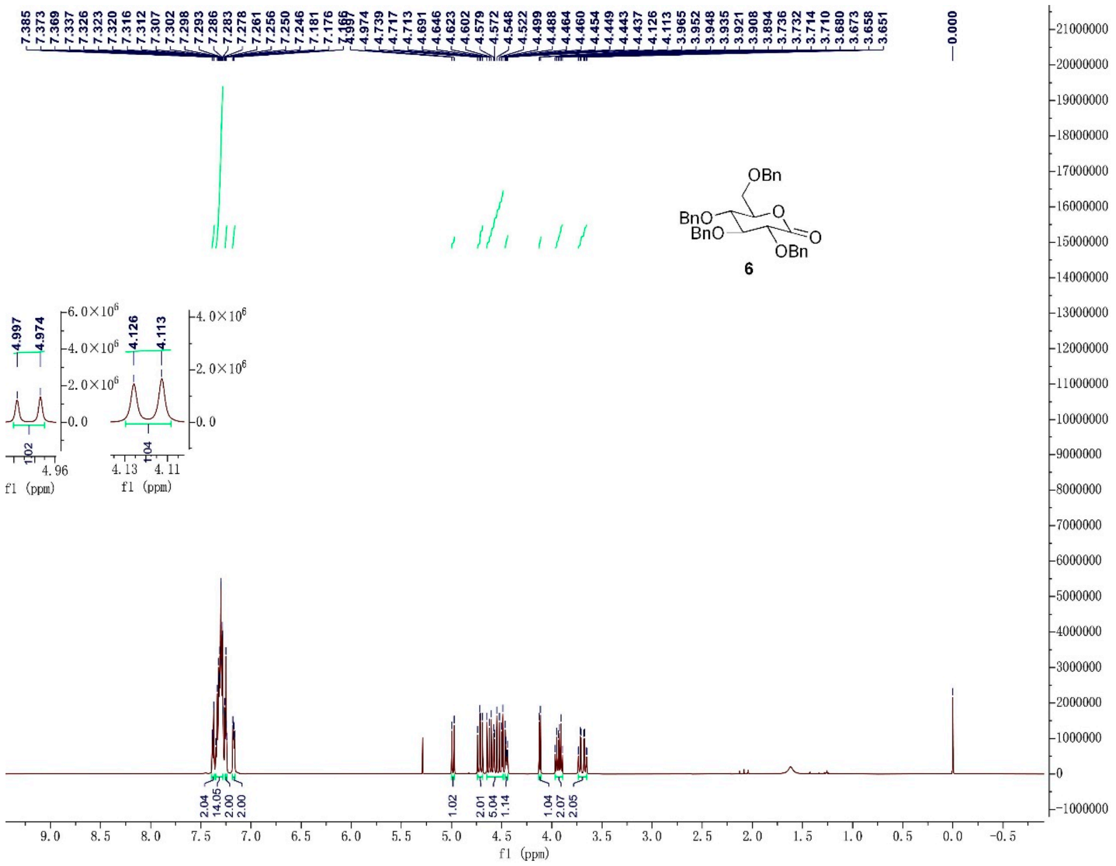

Figure S3 ^{1}H NMR spectrum of **6**

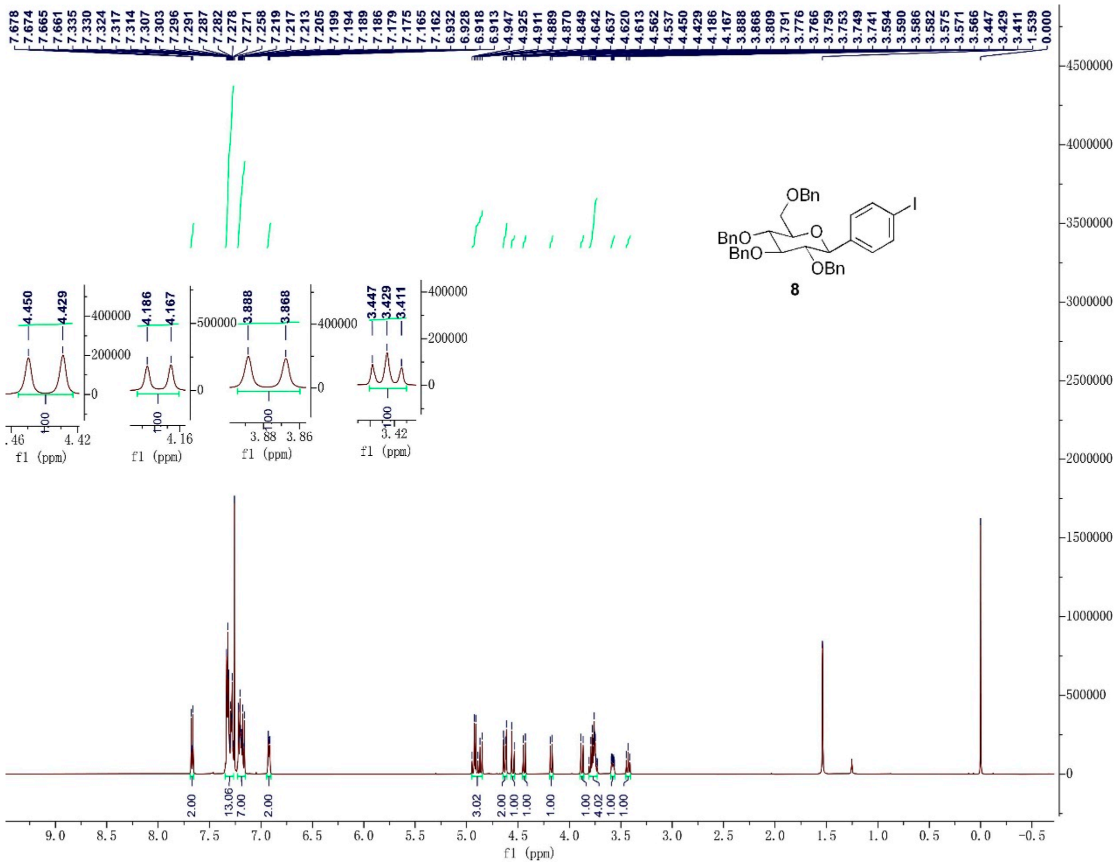

Figure S4 ^{1}H NMR spectrum of **8**

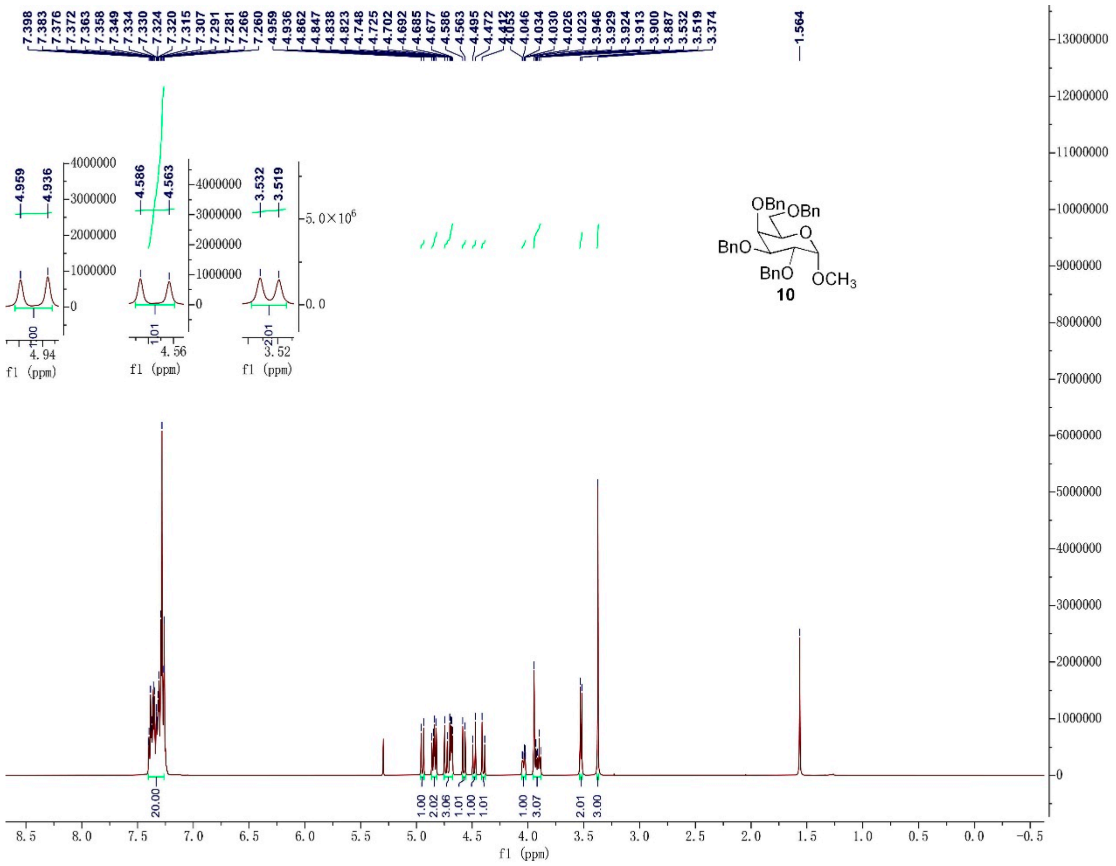

Figure S5 ^{1}H NMR spectrum of **10**

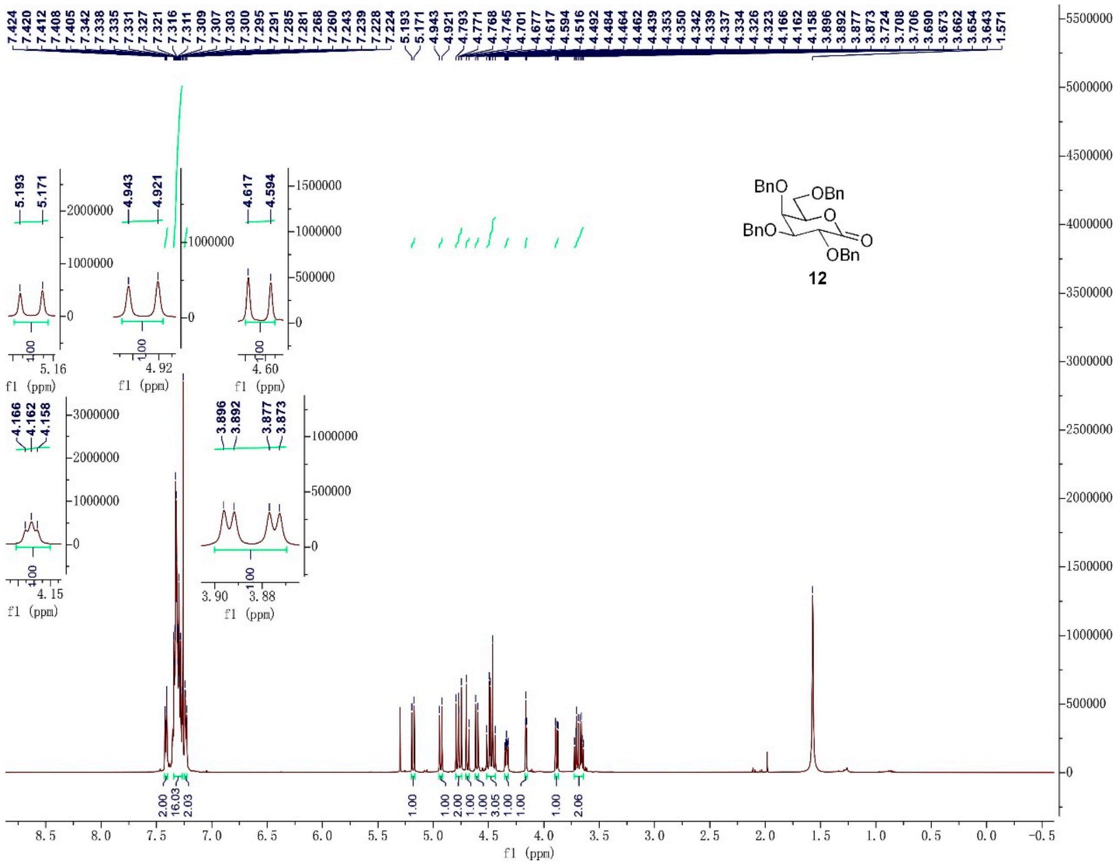

Figure S6 ^{1}H NMR spectrum of **12**

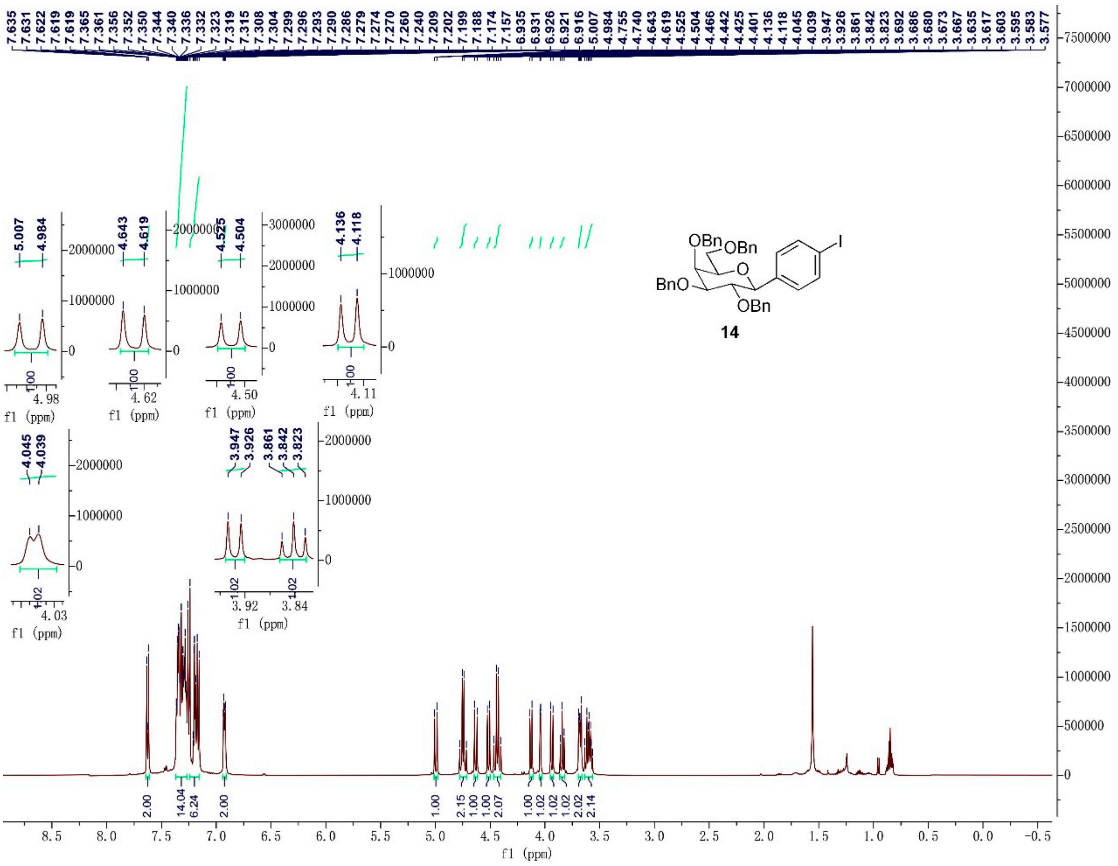

Figure S7 ^{1}H NMR spectrum of **14**

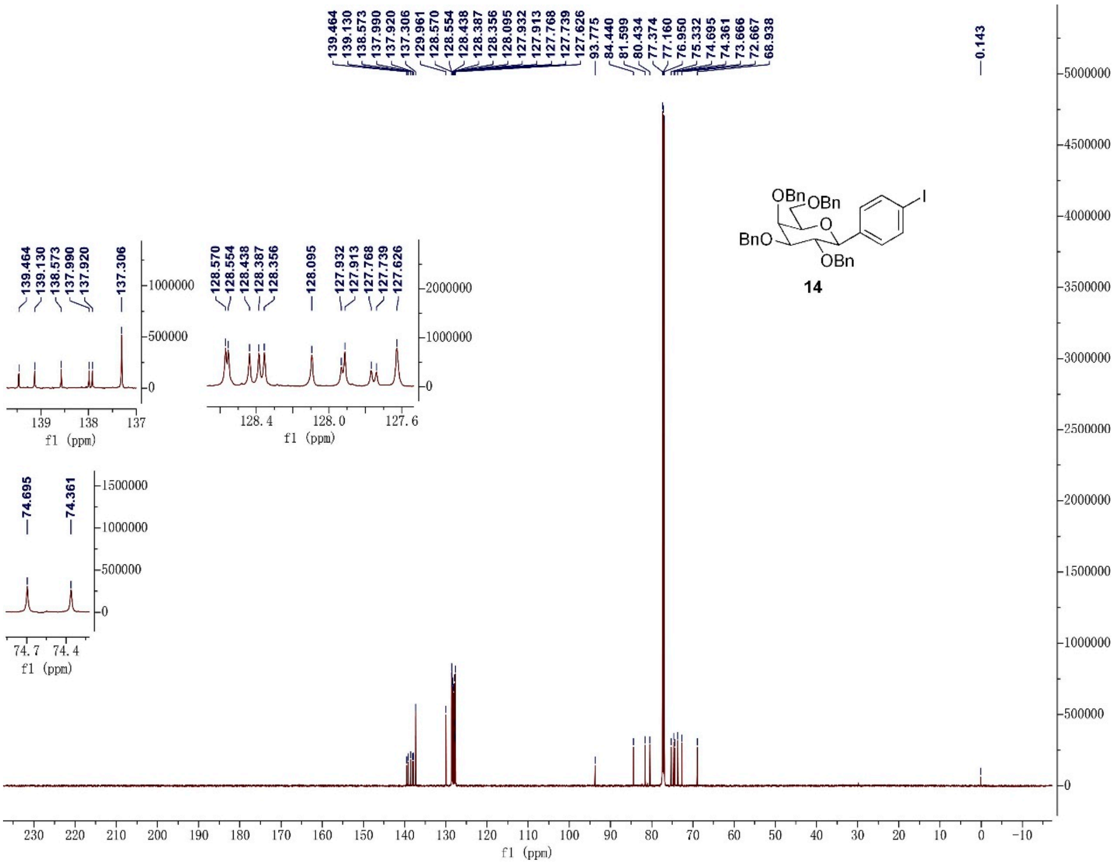

Figure S8 ^{13}C NMR spectrum of **14**

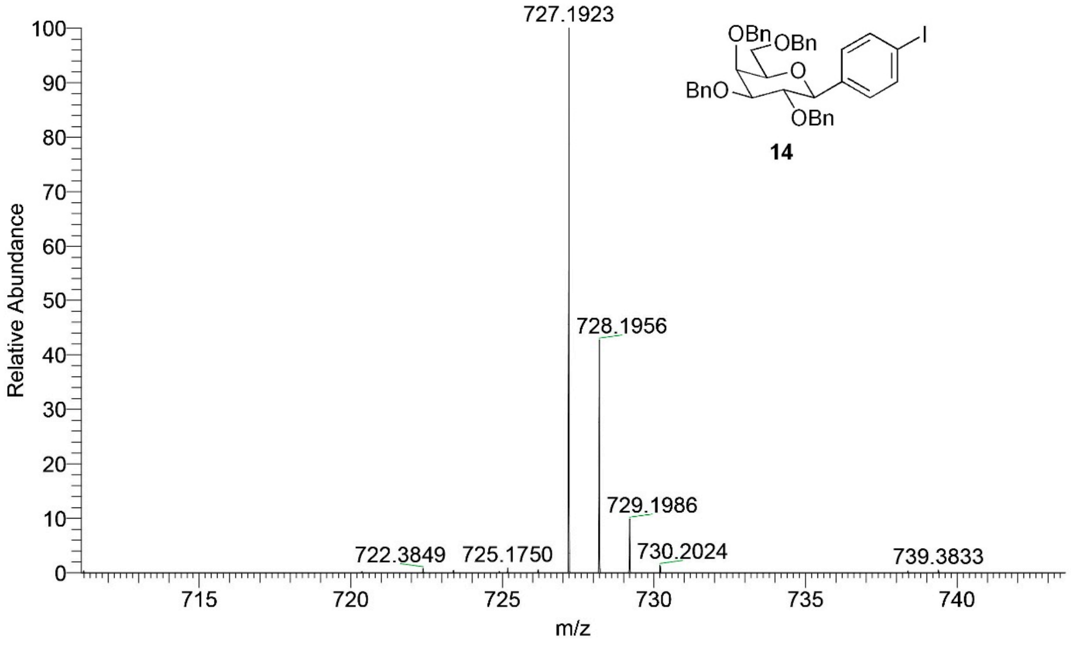

Figure S9 HR-MS (ESI/ion trap) spectrum of **14**

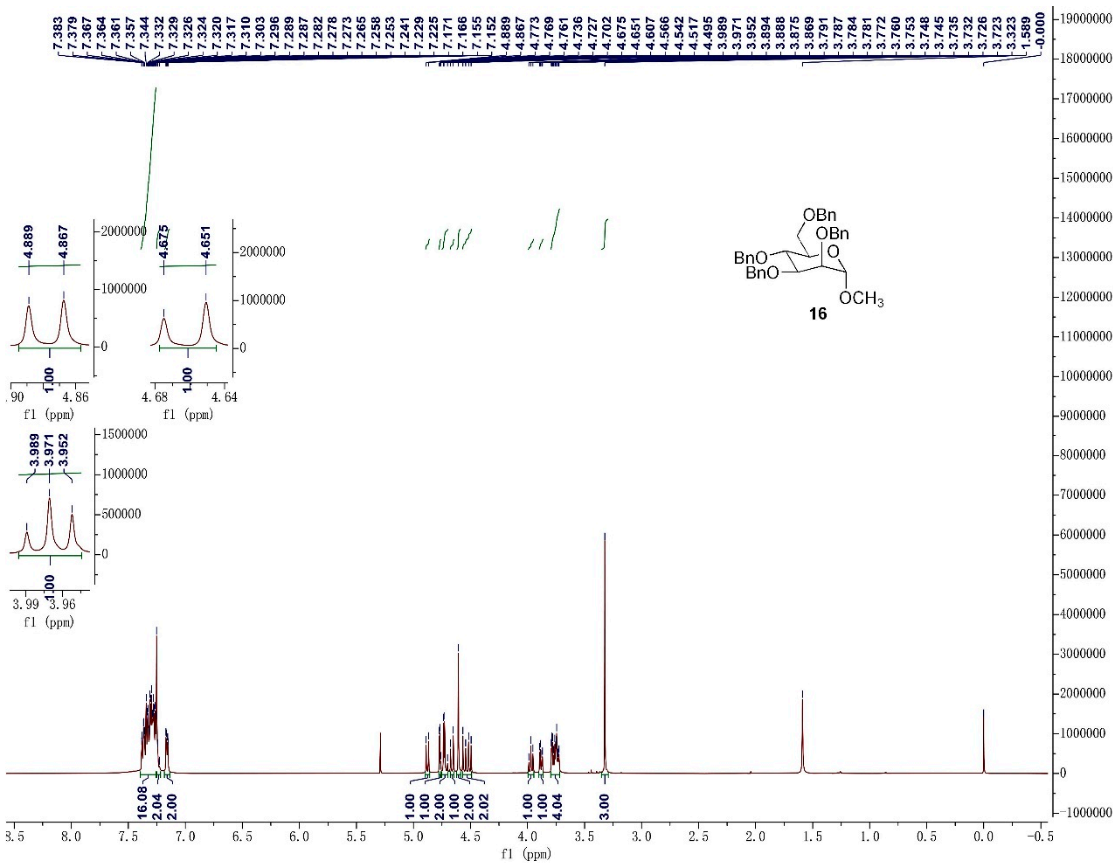

Figure S10 ^{1}H NMR spectrum of **16**

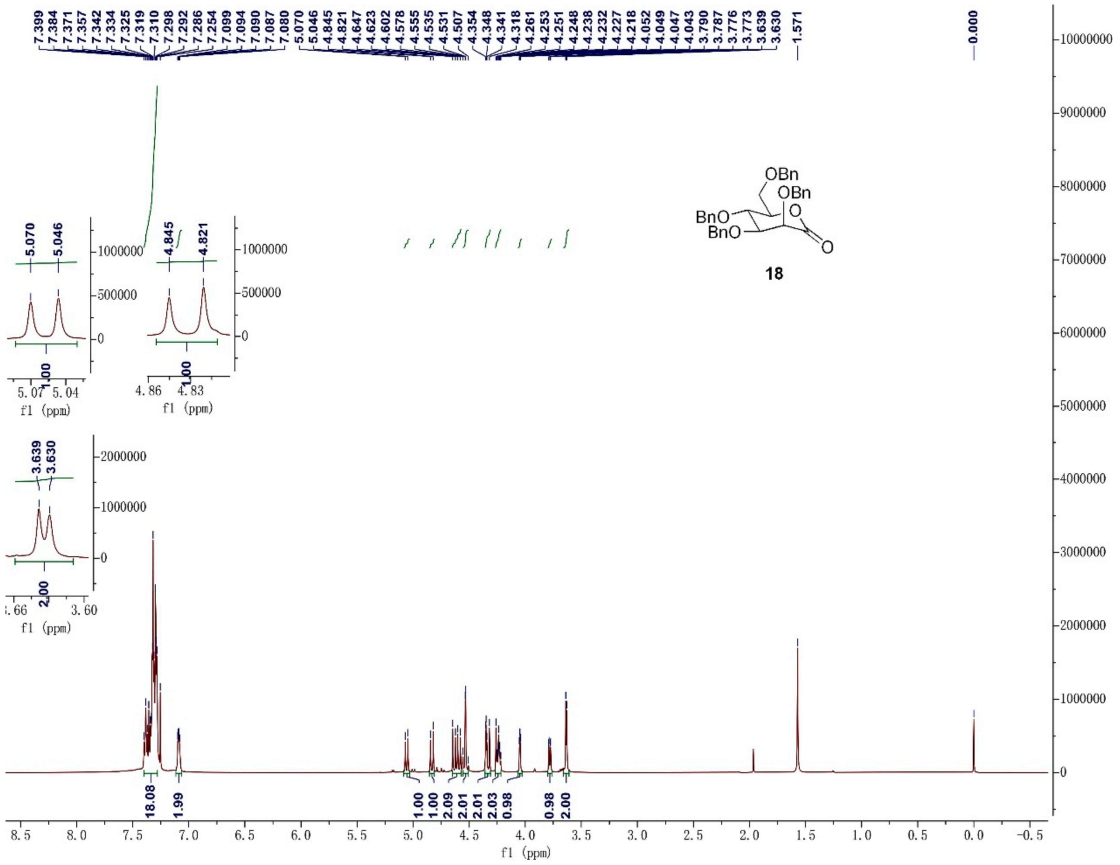

Figure S11 ^{1}H NMR spectrum of **18**

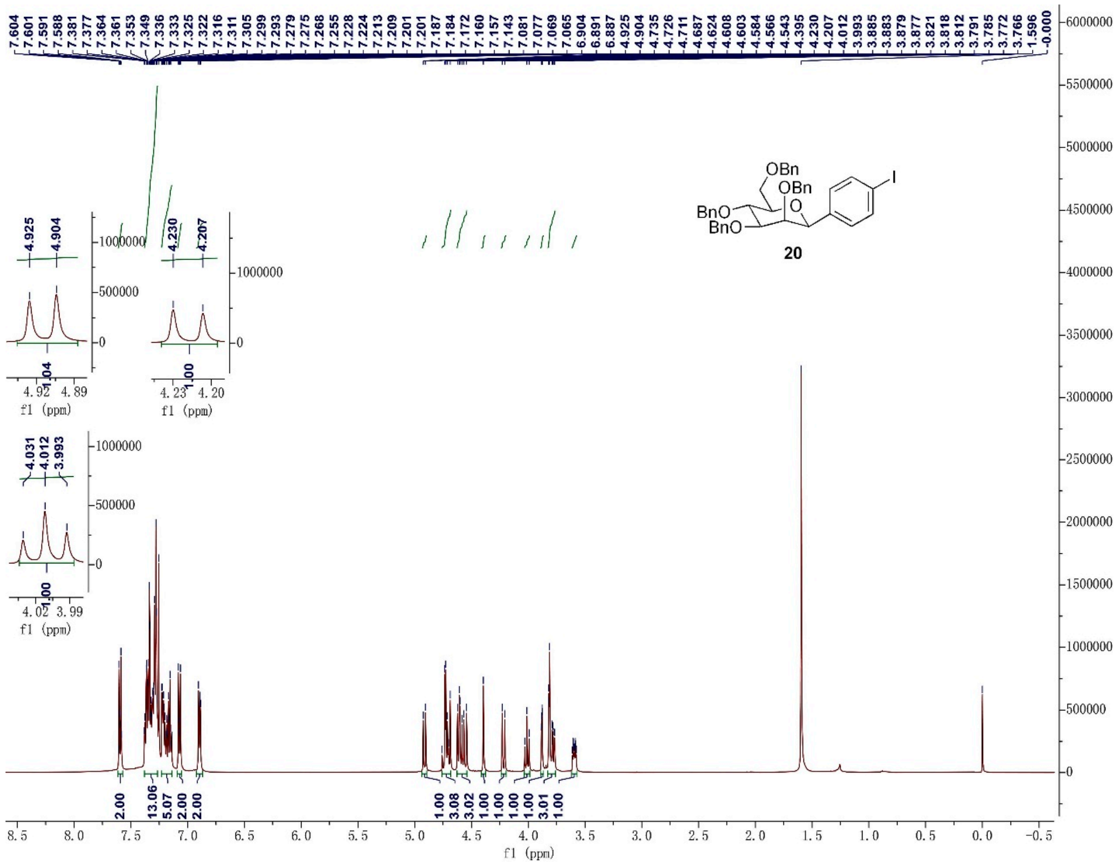

Figure S12 ^{1}H NMR spectrum of **20**

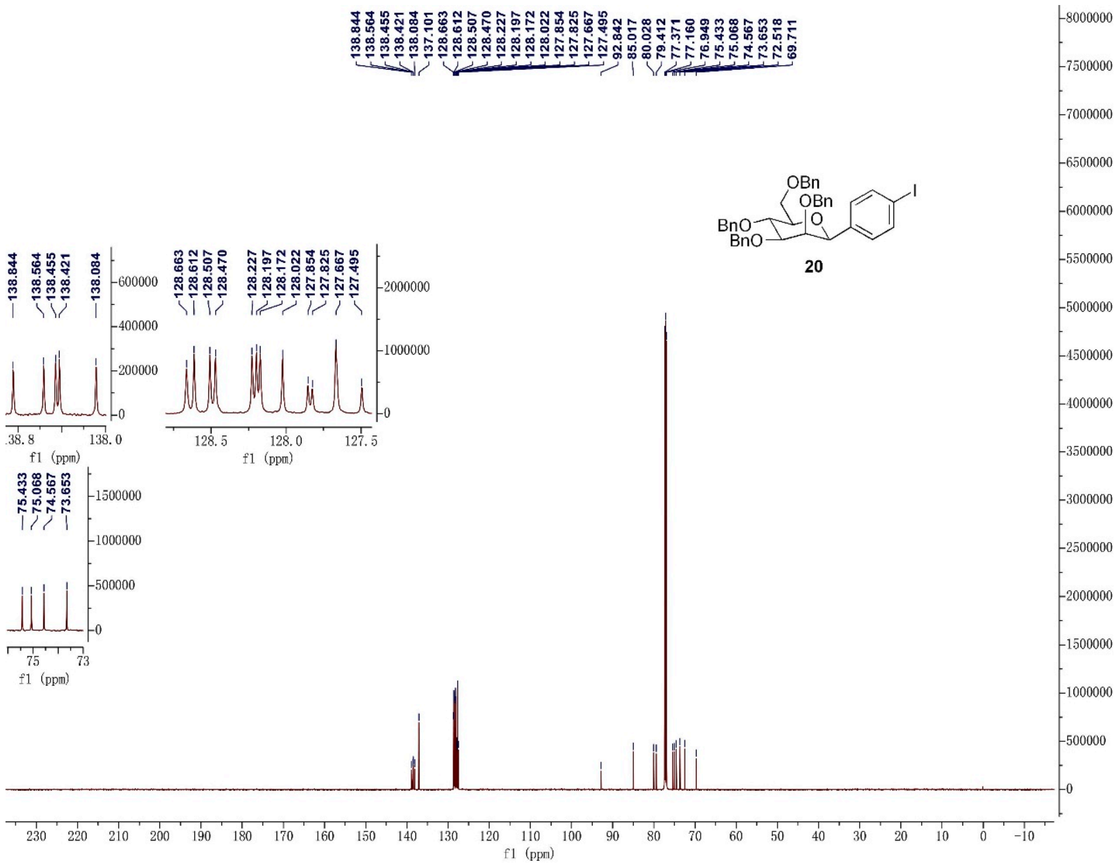

Figure S13 ^{13}C NMR spectrum of **20**

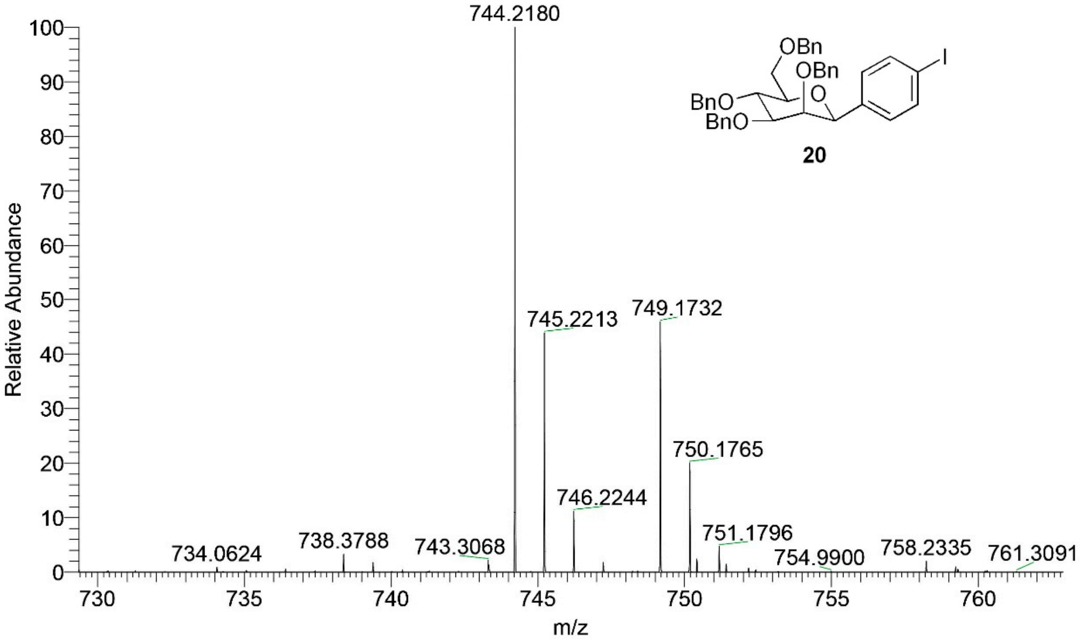

Figure S14 HR-MS (ESI/ion trap) spectrum of **20**

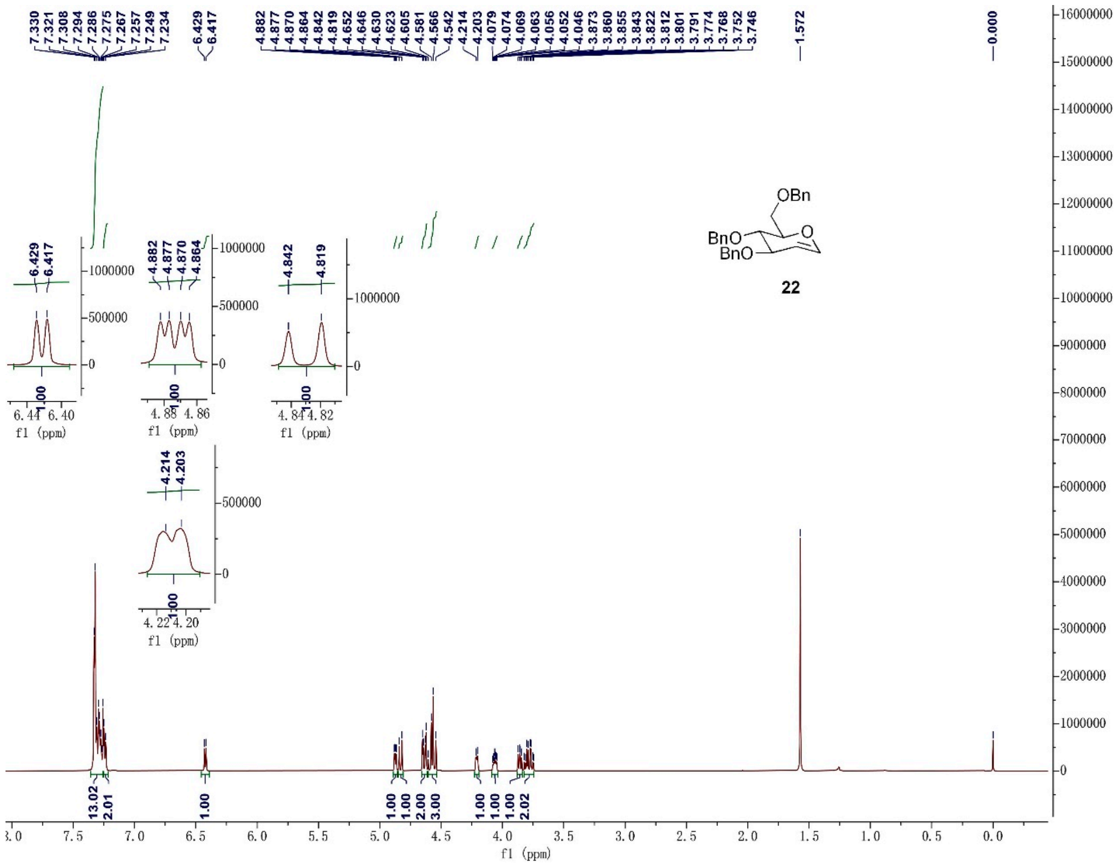

Figure S15 ^{1}H NMR spectrum of **22**

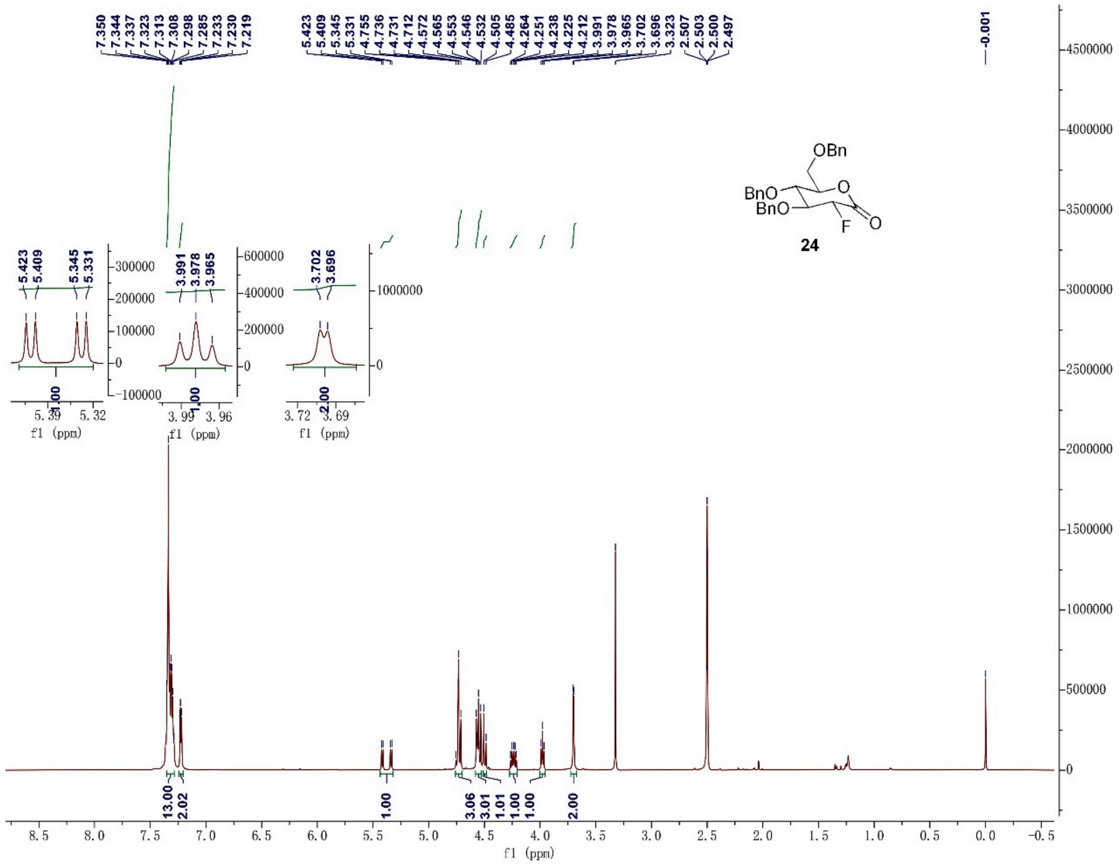

Figure S16 ^{1}H NMR spectrum of **24**

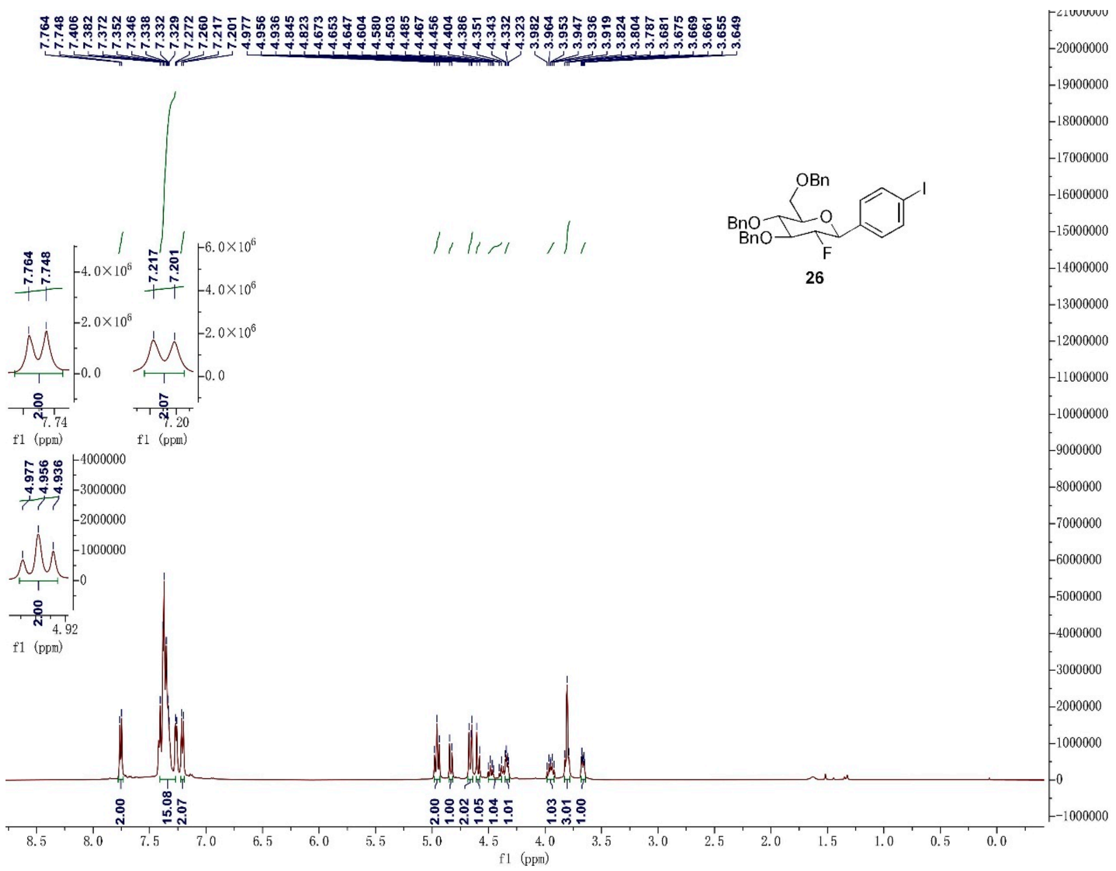

Figure S17 ^{1}H NMR spectrum of **26**

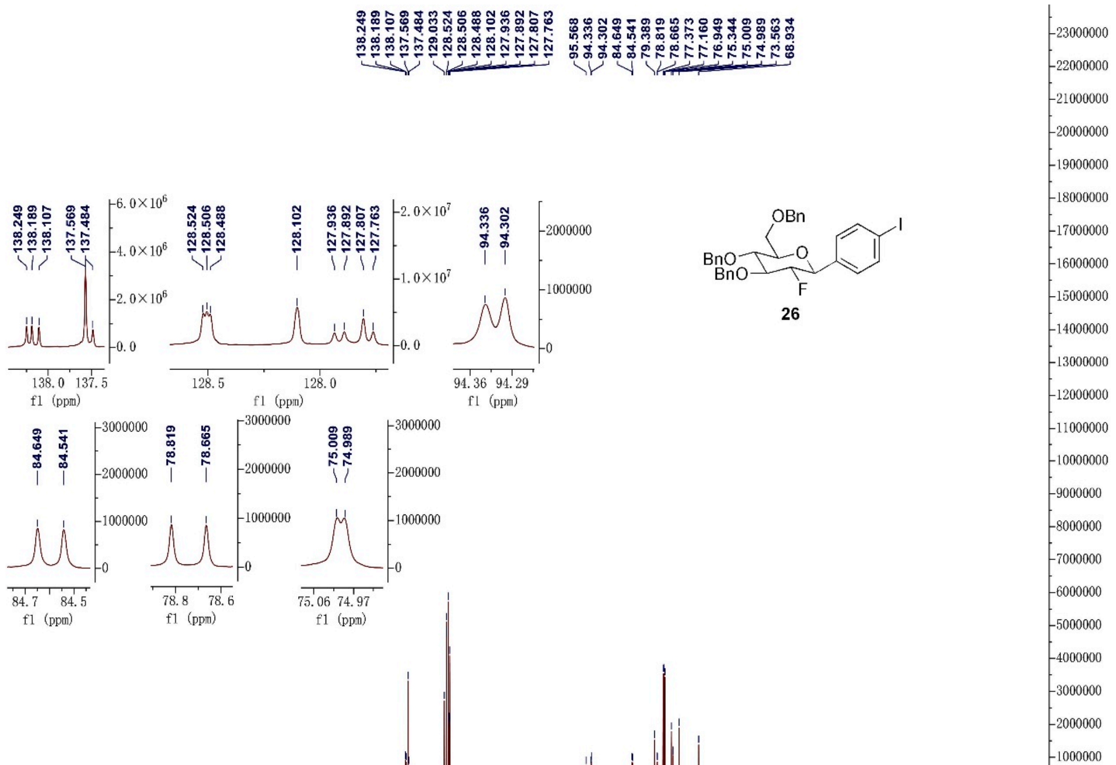

Figure S18 ^{13}C NMR spectrum of **26**

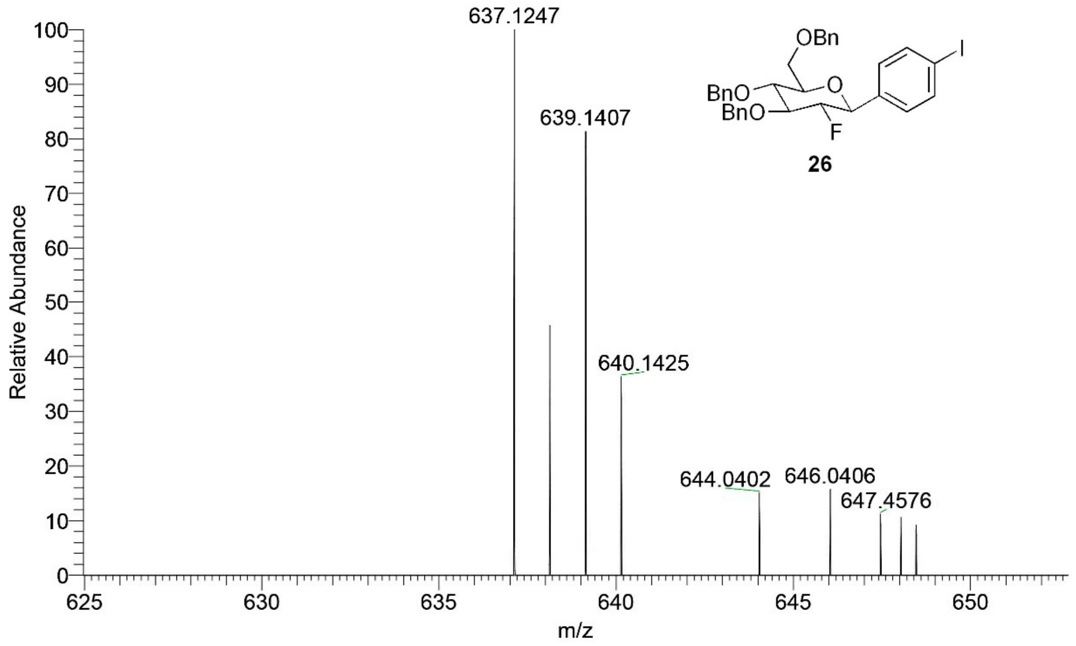

Figure S19 HR-MS (ESI/ion trap) spectrum of **26**

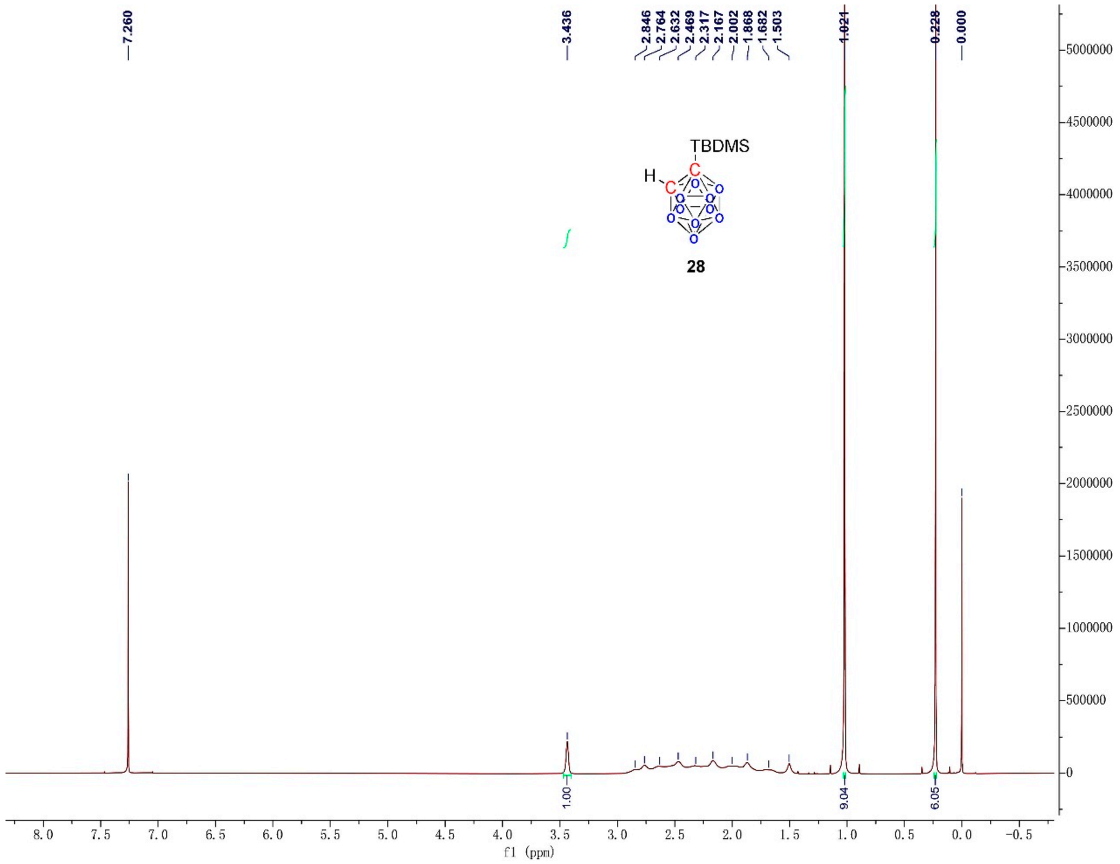

Figure S20 ^{1}H NMR spectrum of **28**

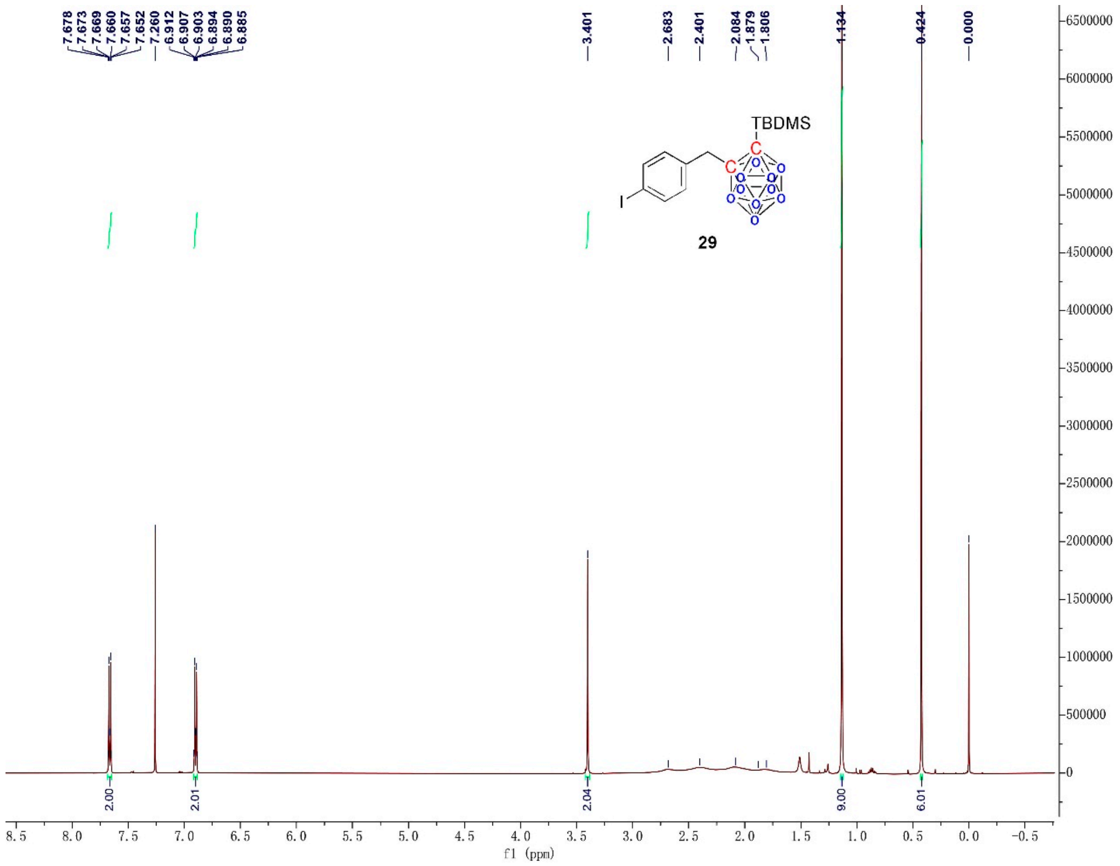

Figure S21 ^{1}H NMR spectrum of **29**

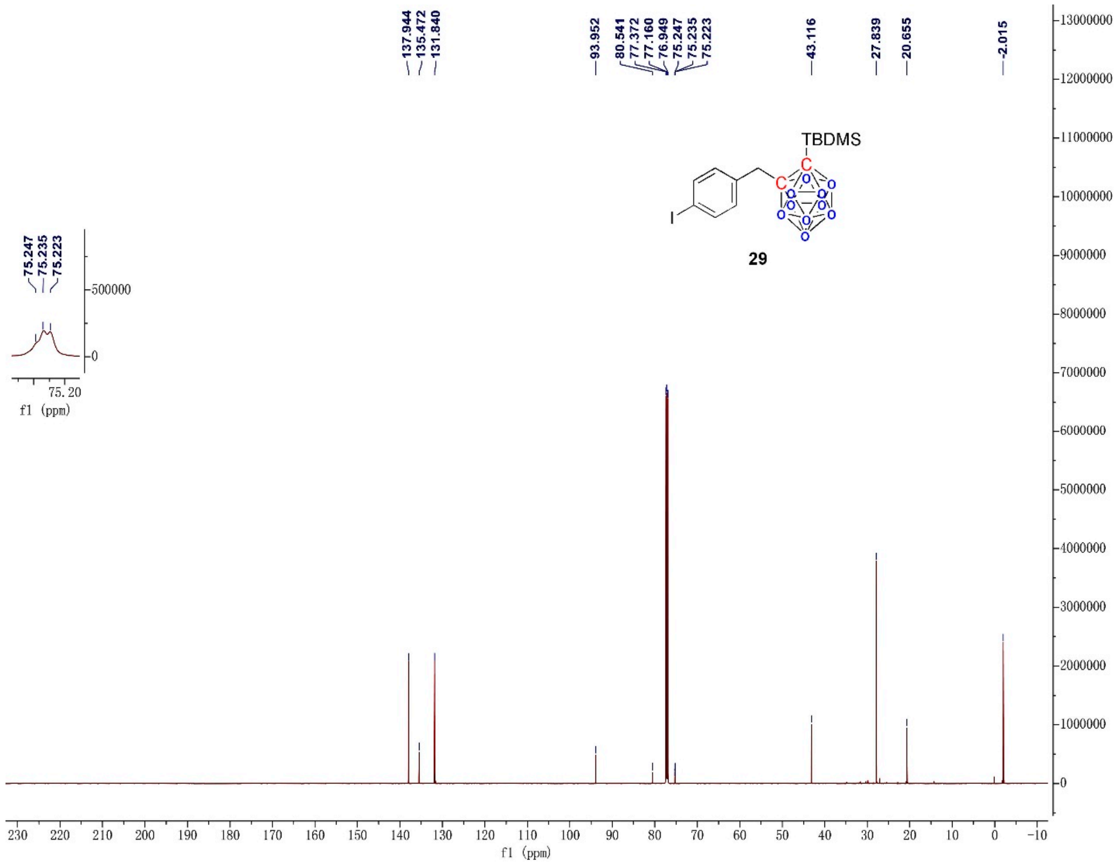

Figure S22 ^{13}C NMR spectrum of **29**

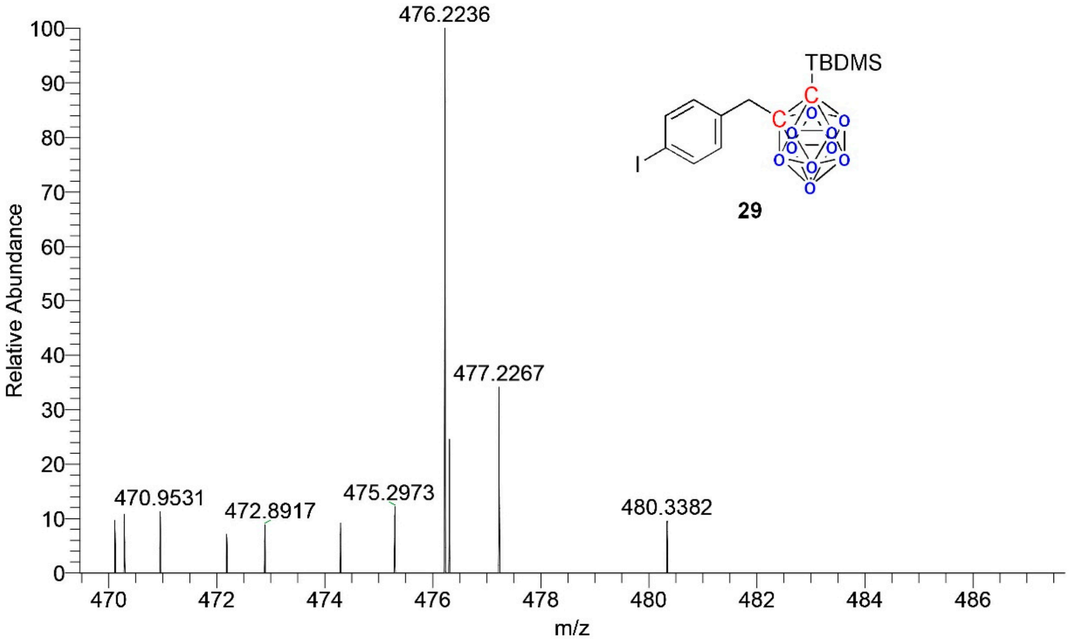

Figure S23 HR-MS (ESI/ion trap) spectrum of **29**

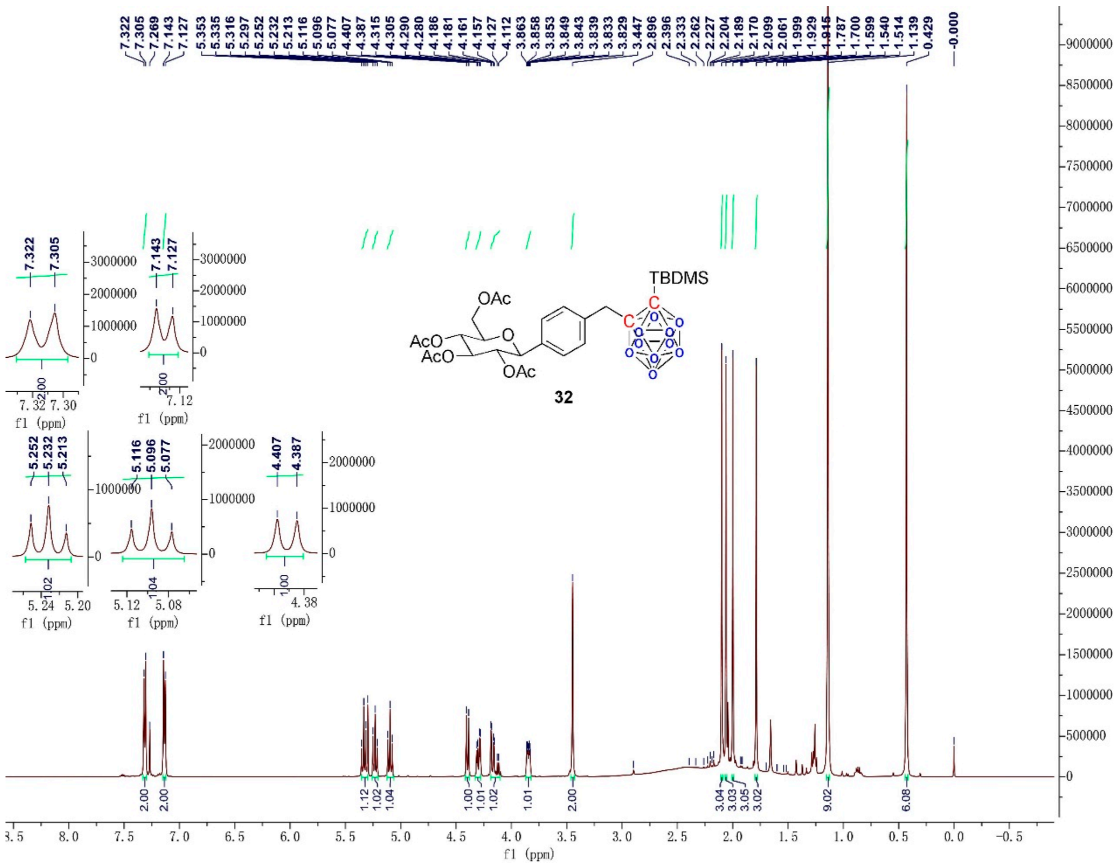

Figure S24 ^{1}H NMR spectrum of **32**

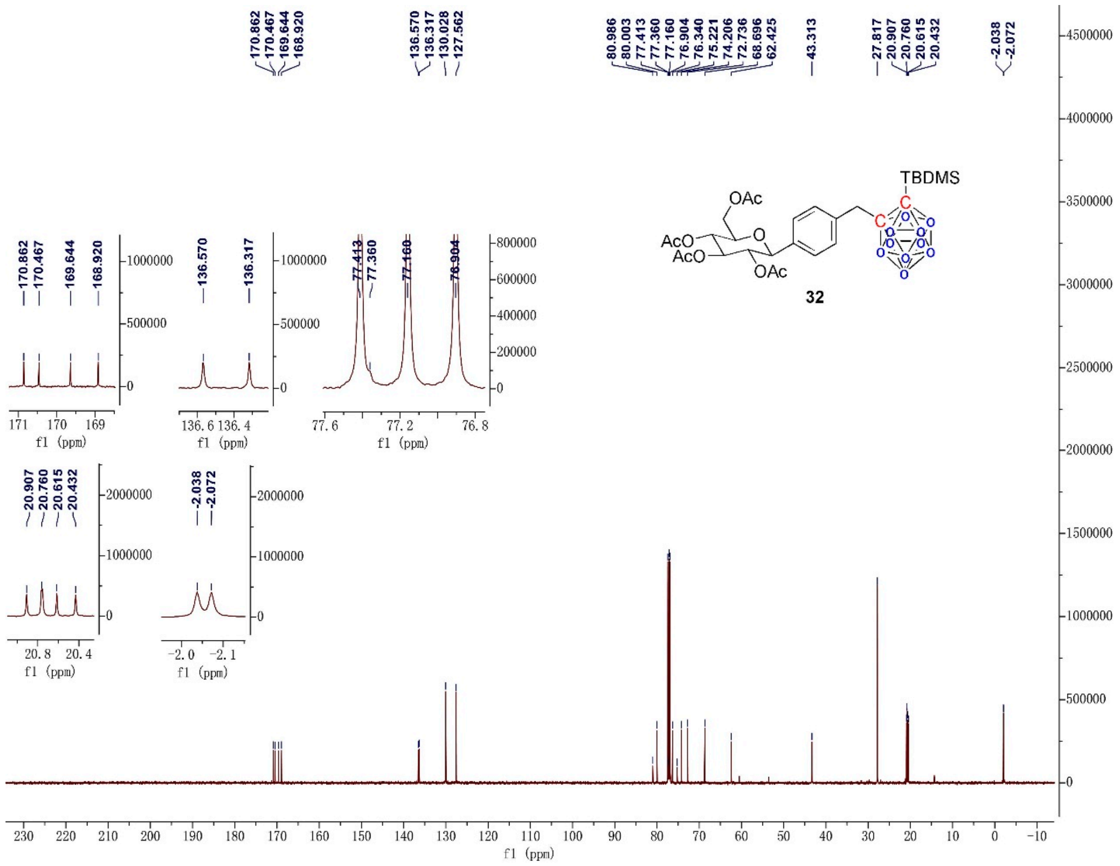

Figure S25 ^{13}C NMR spectrum of **32**

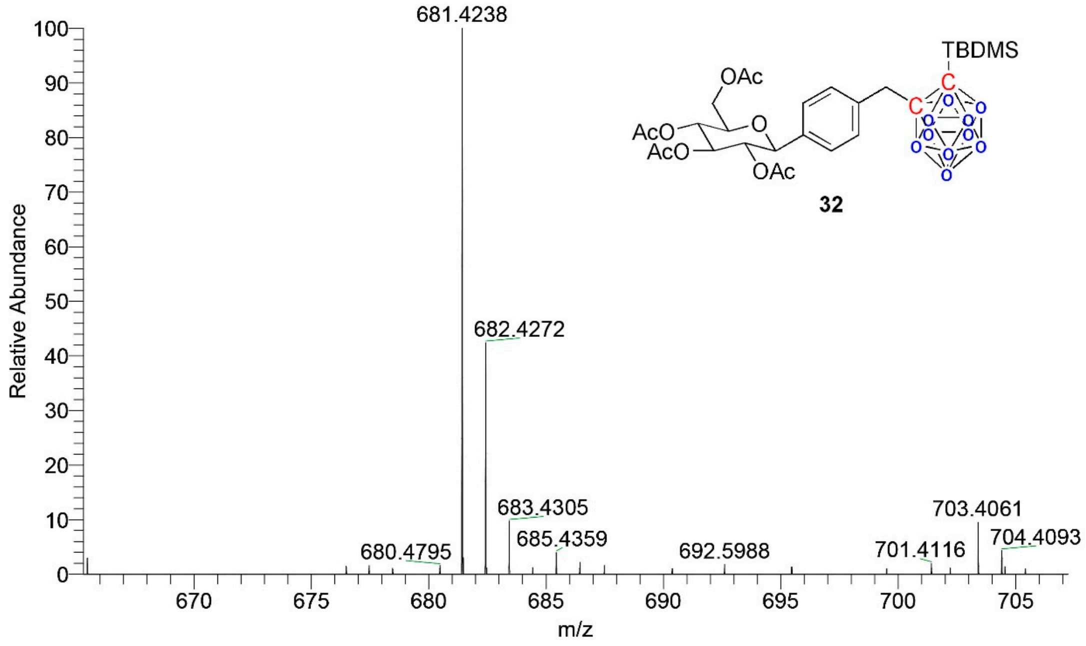

Figure S26 HR-MS (ESI/ion trap) spectrum of **32**

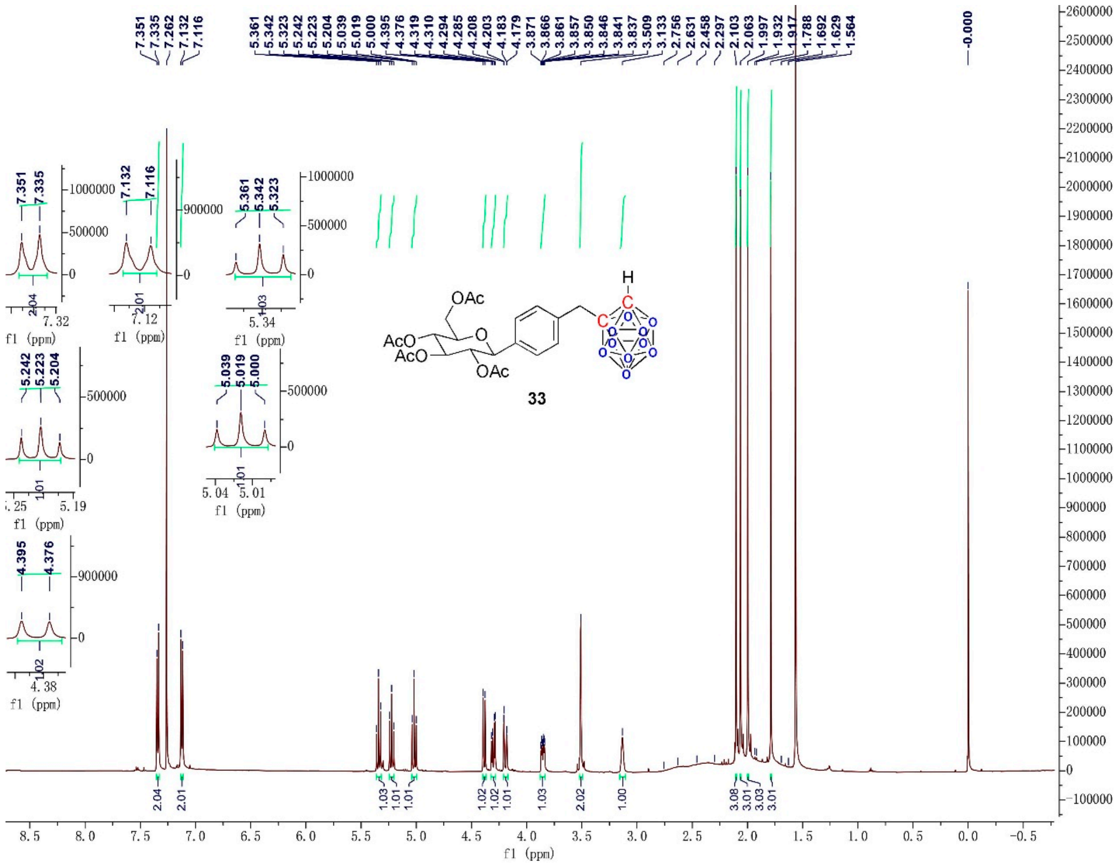

Figure S27 ^{1}H NMR spectrum of **33**

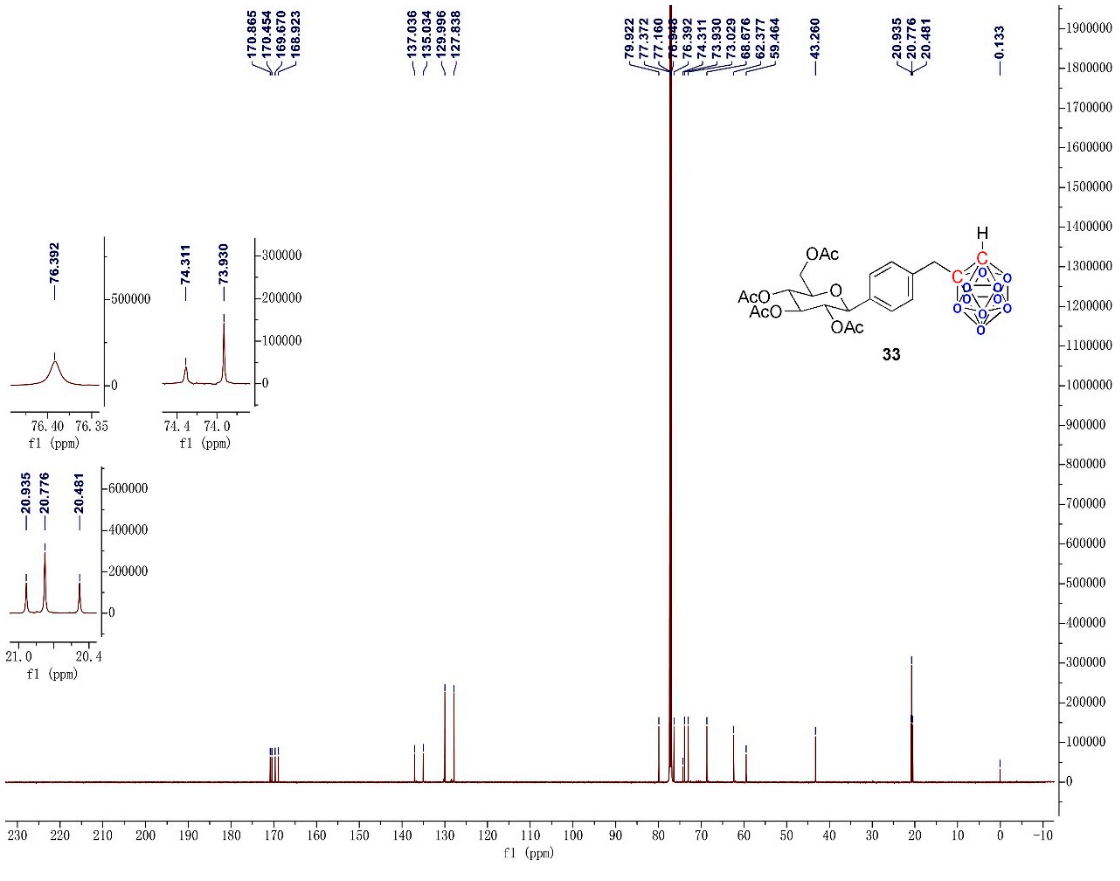

Figure S28 ^{13}C NMR spectrum of **33**

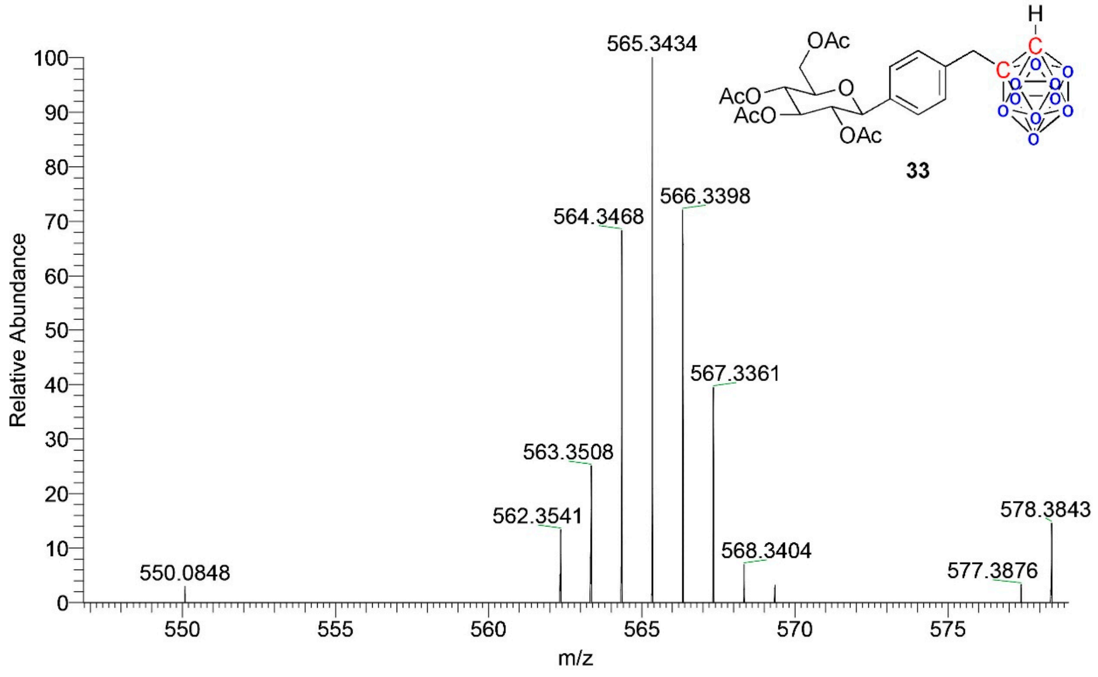

Figure S29 HR-MS (ESI/ion trap) spectrum of **33**

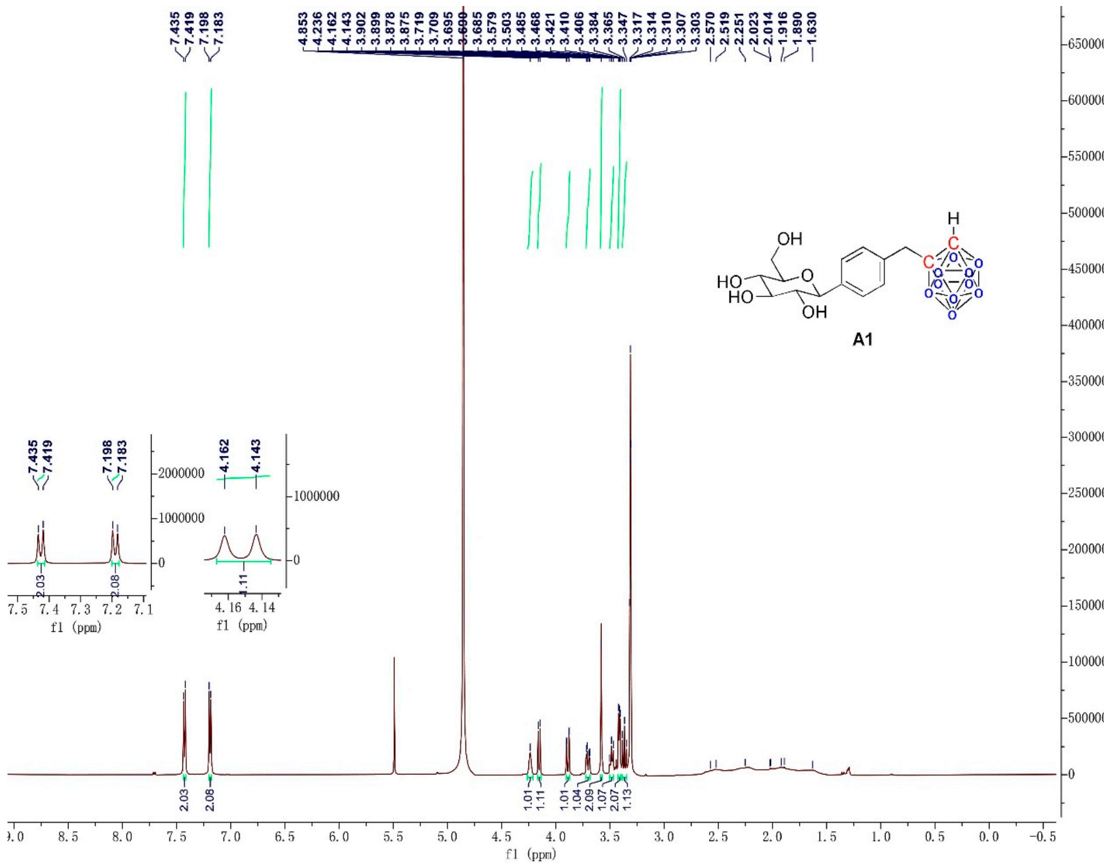

Figure S30 ^{1}H NMR spectrum of **A1**

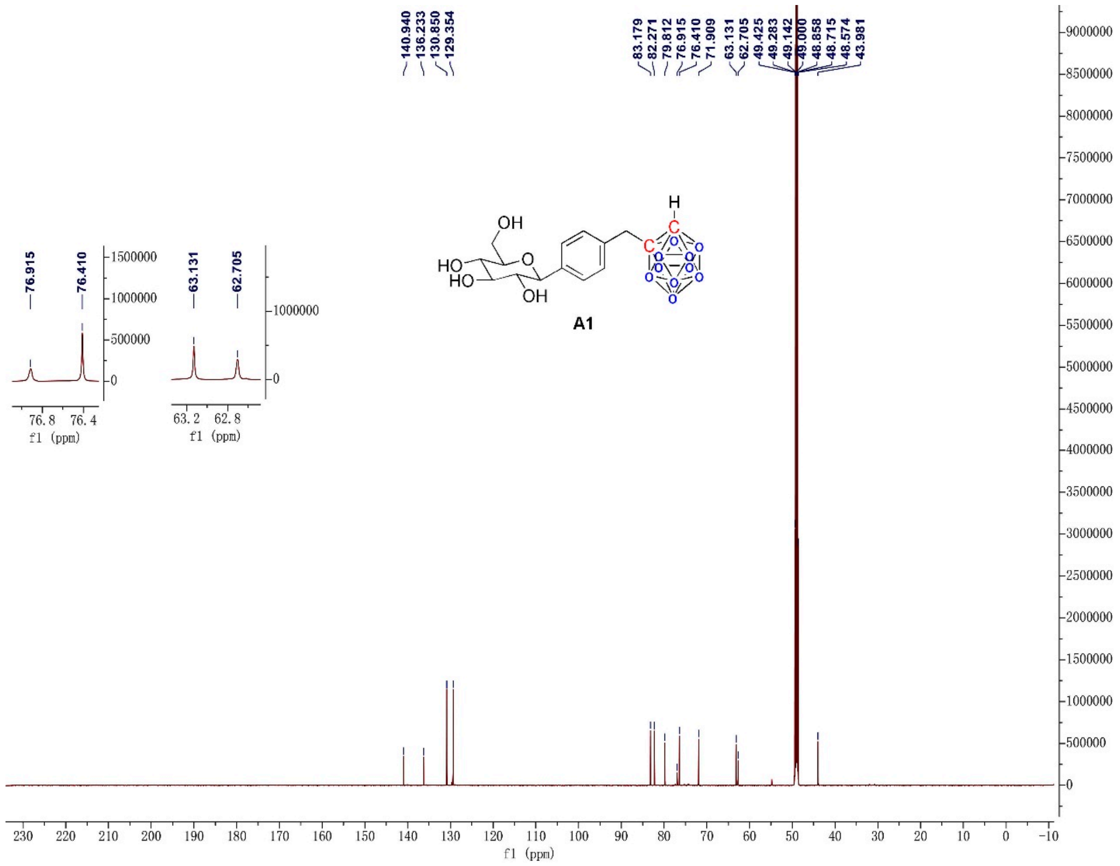

Figure S31 ^{13}C NMR spectrum of **A1**

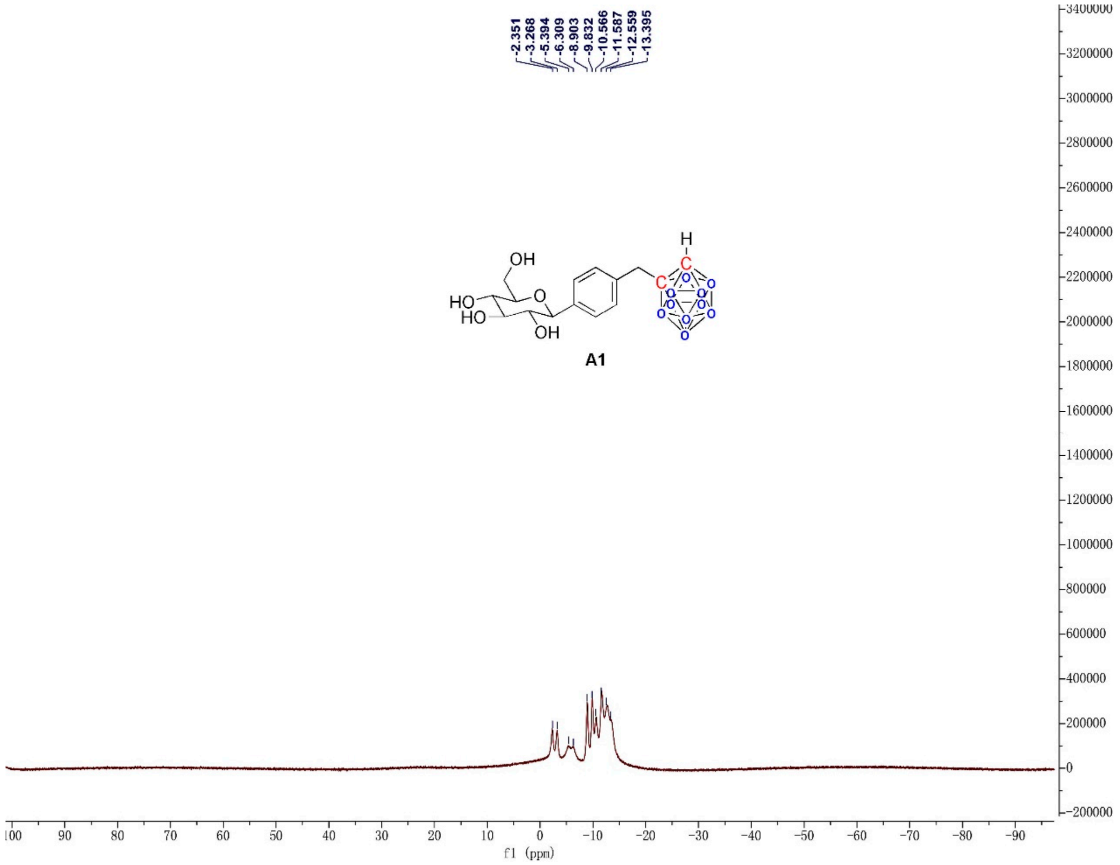

Figure S32 ^{11}B NMR spectrum of **A1**

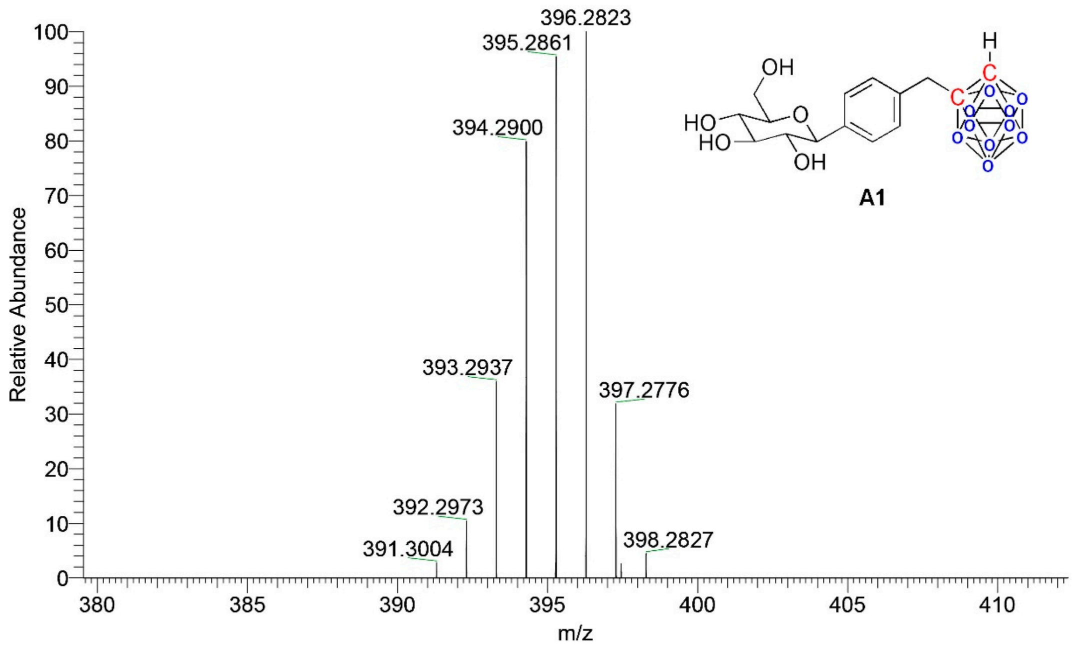

Figure S33 HR-MS (ESI/ion trap) spectrum of **A1**

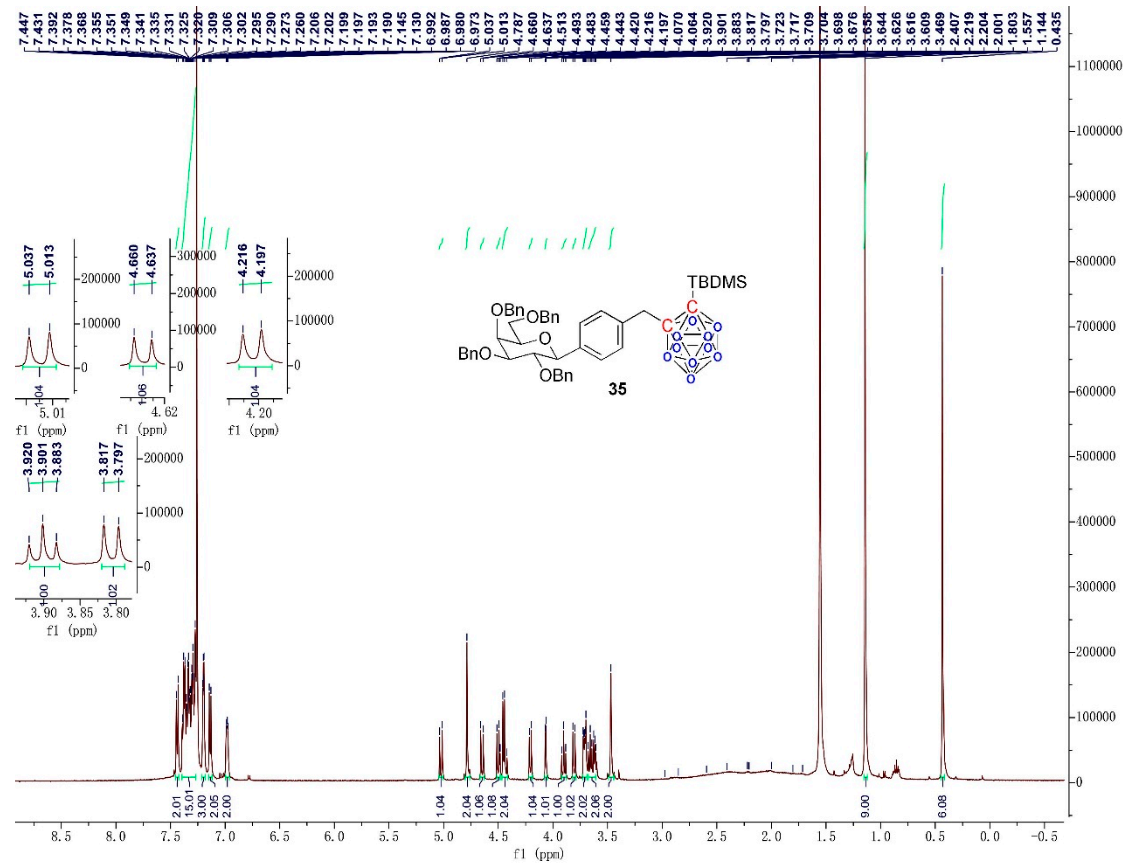

Figure S34 ^{1}H NMR spectrum of **35**

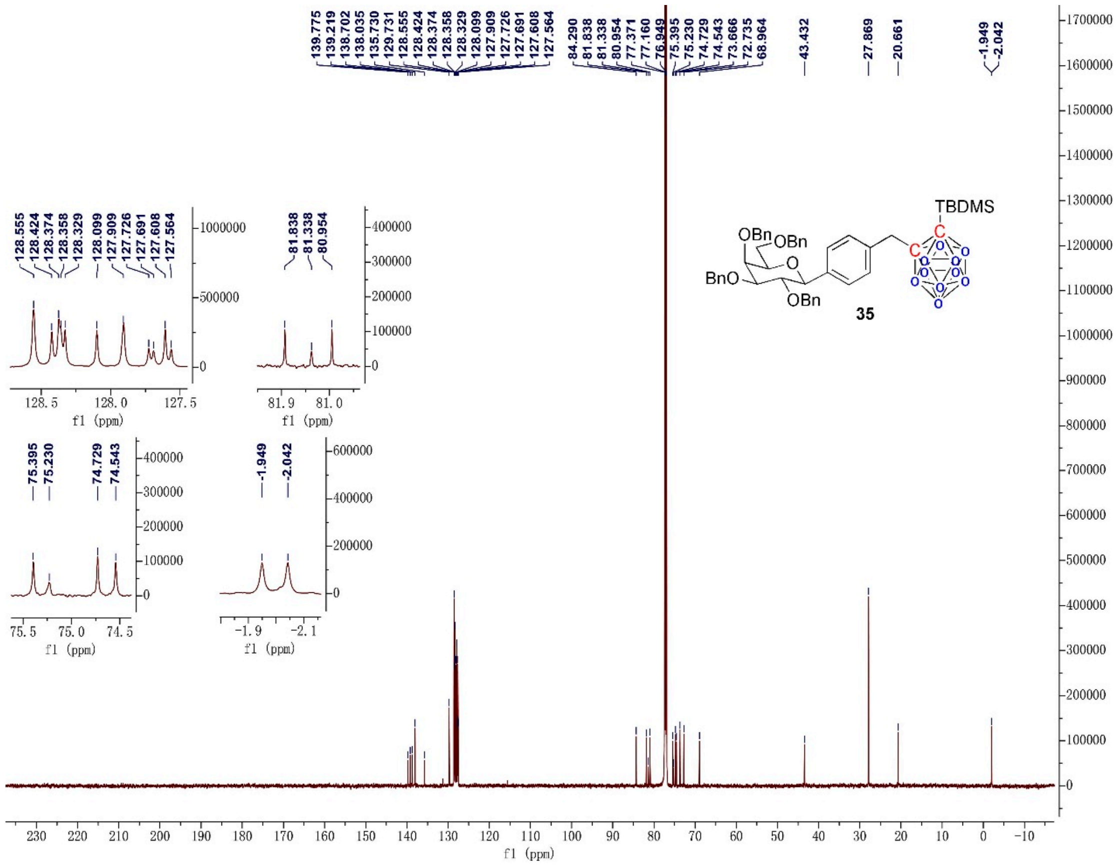

Figure S35 ^{13}C NMR spectrum of **35**

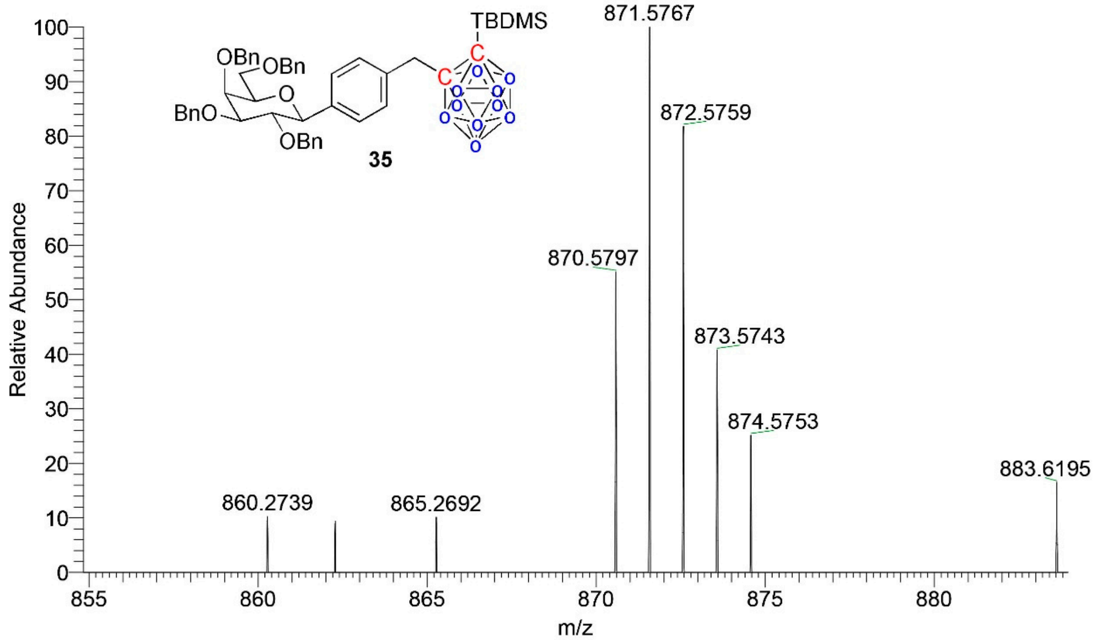

Figure S36 HR-MS (ESI/ion trap) spectrum of **35**

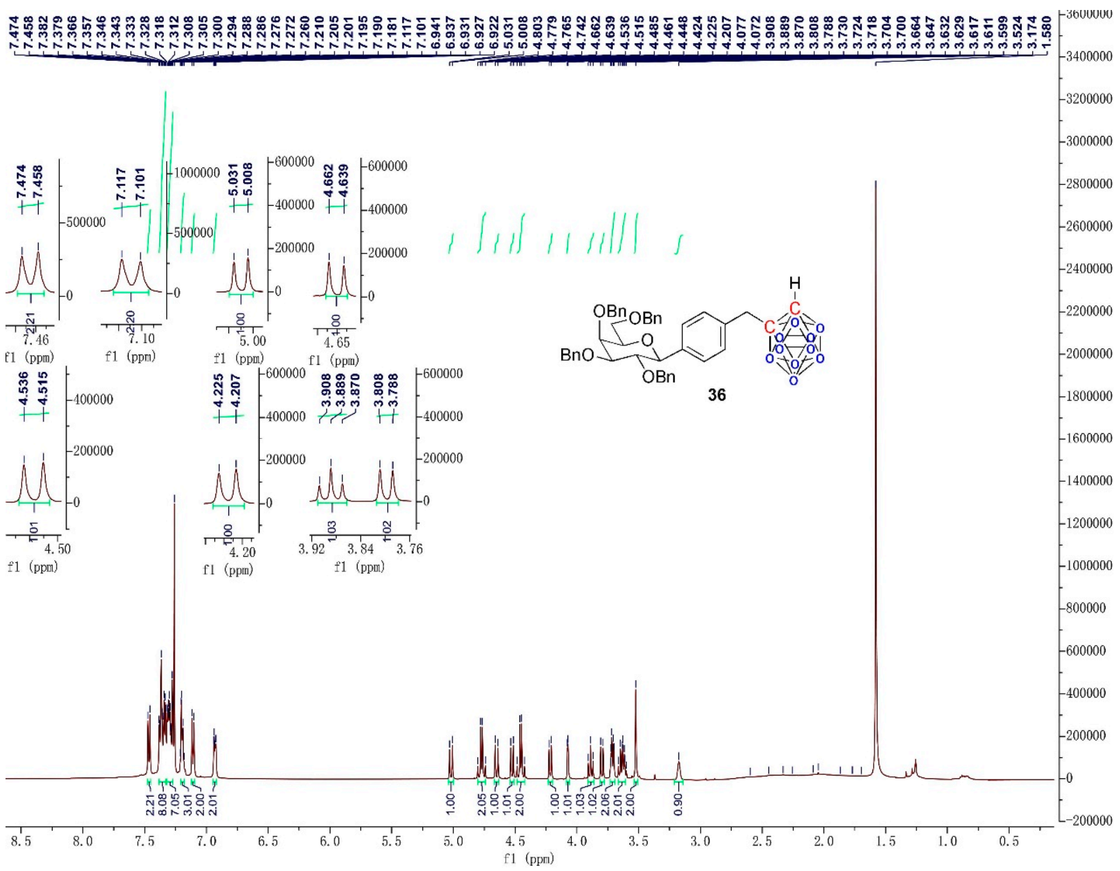

Figure S37 ^{1}H NMR spectrum of **36**

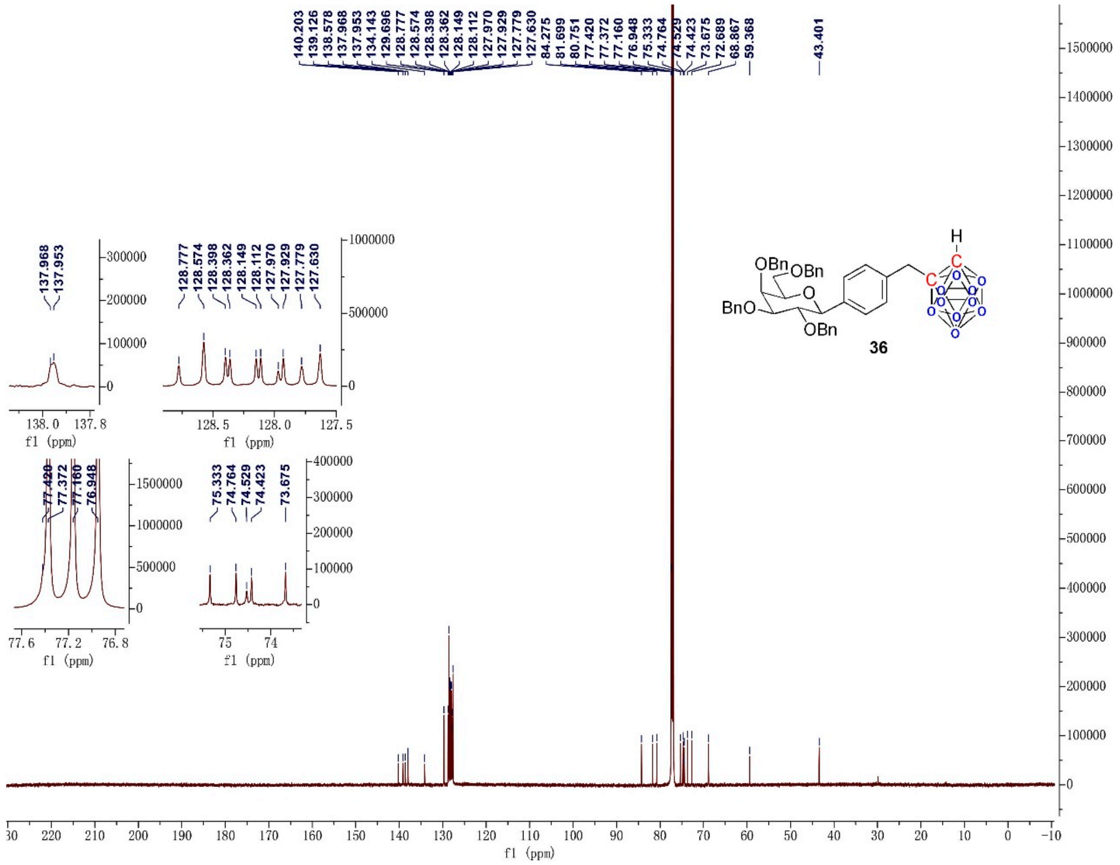

Figure S38 ^{13}C NMR spectrum of **36**

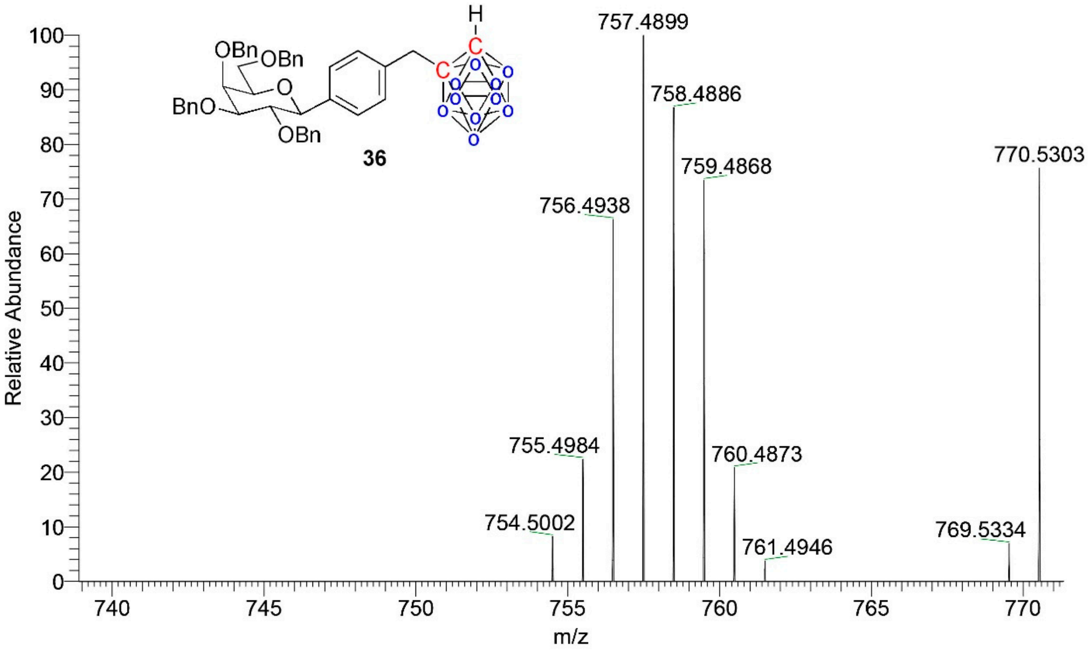

Figure S39 HR-MS (ESI/ion trap) spectrum of **36**

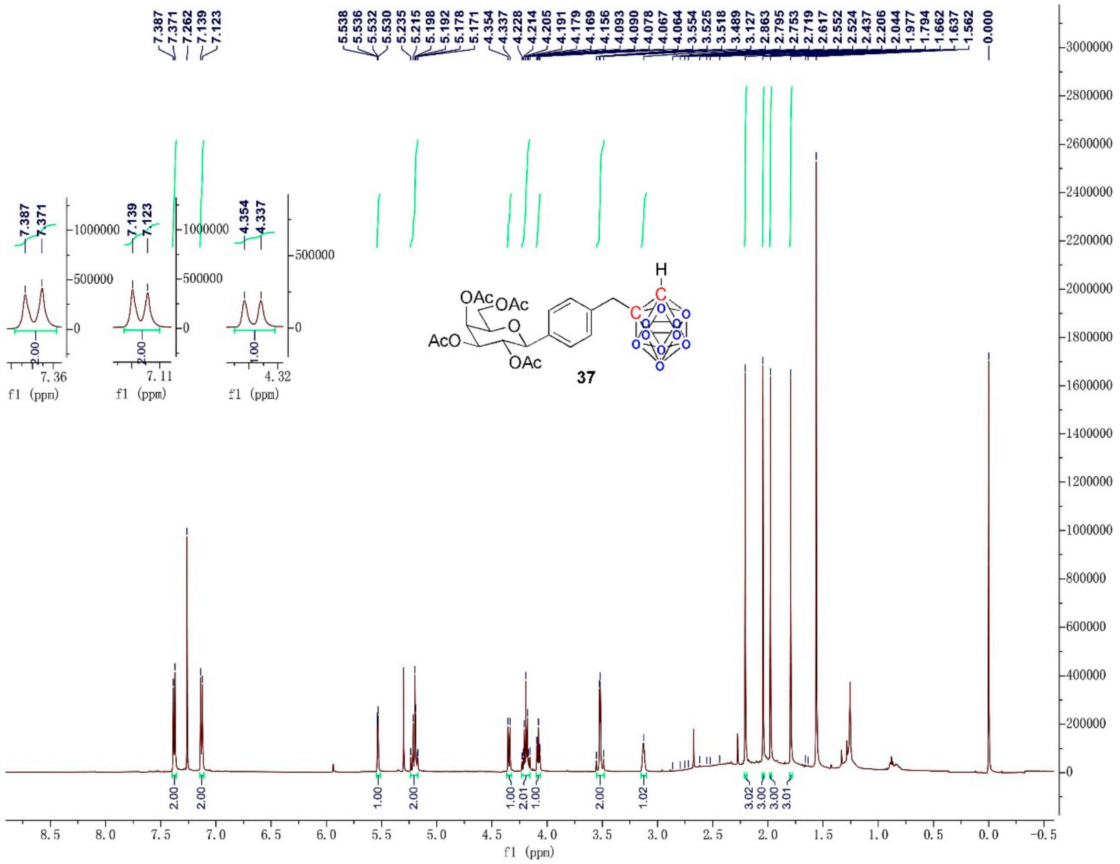

Figure S40 ^{1}H NMR spectrum of **37**

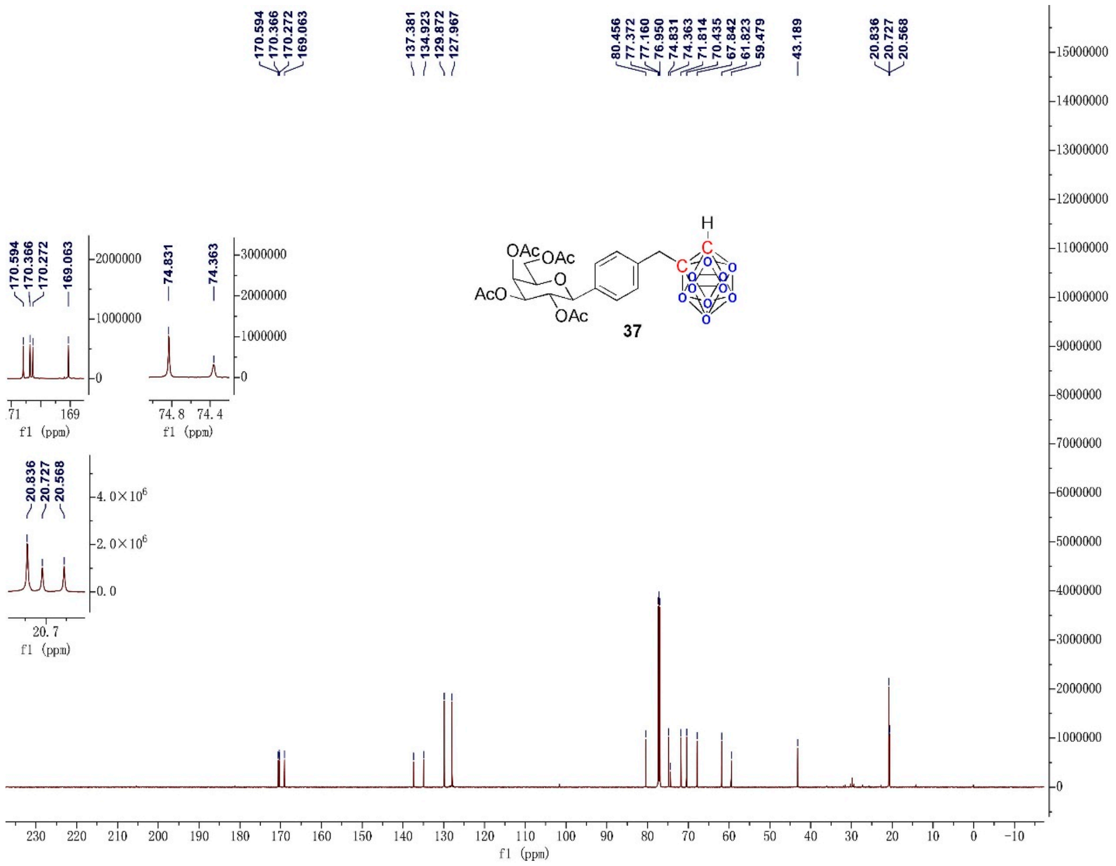

Figure S41 ^{13}C NMR spectrum of **37**

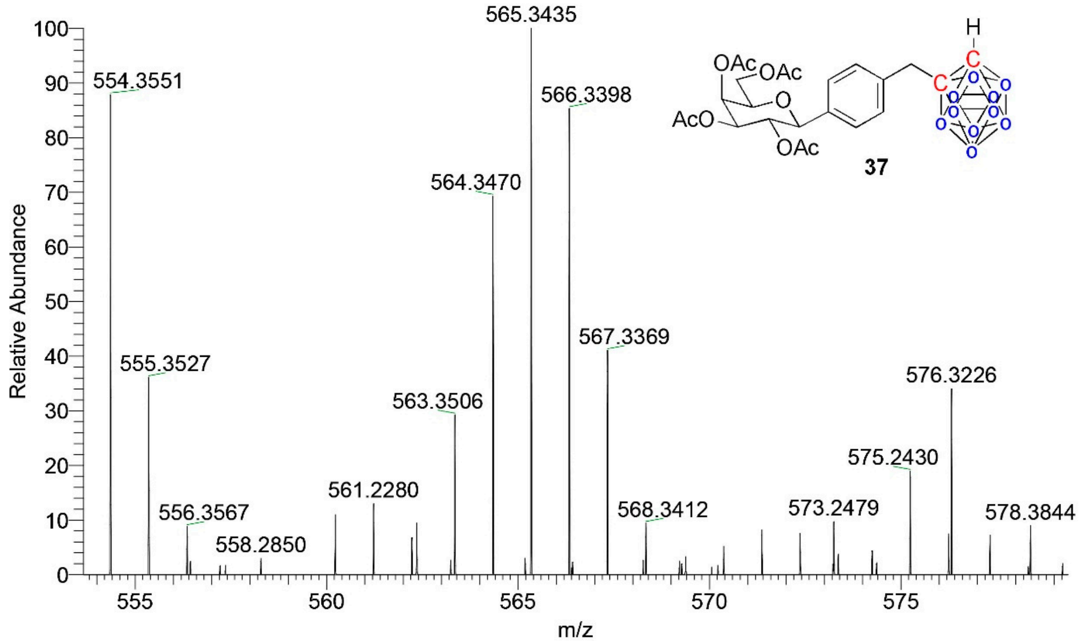

Figure S42 HR-MS (ESI/ion trap) spectrum of **37**

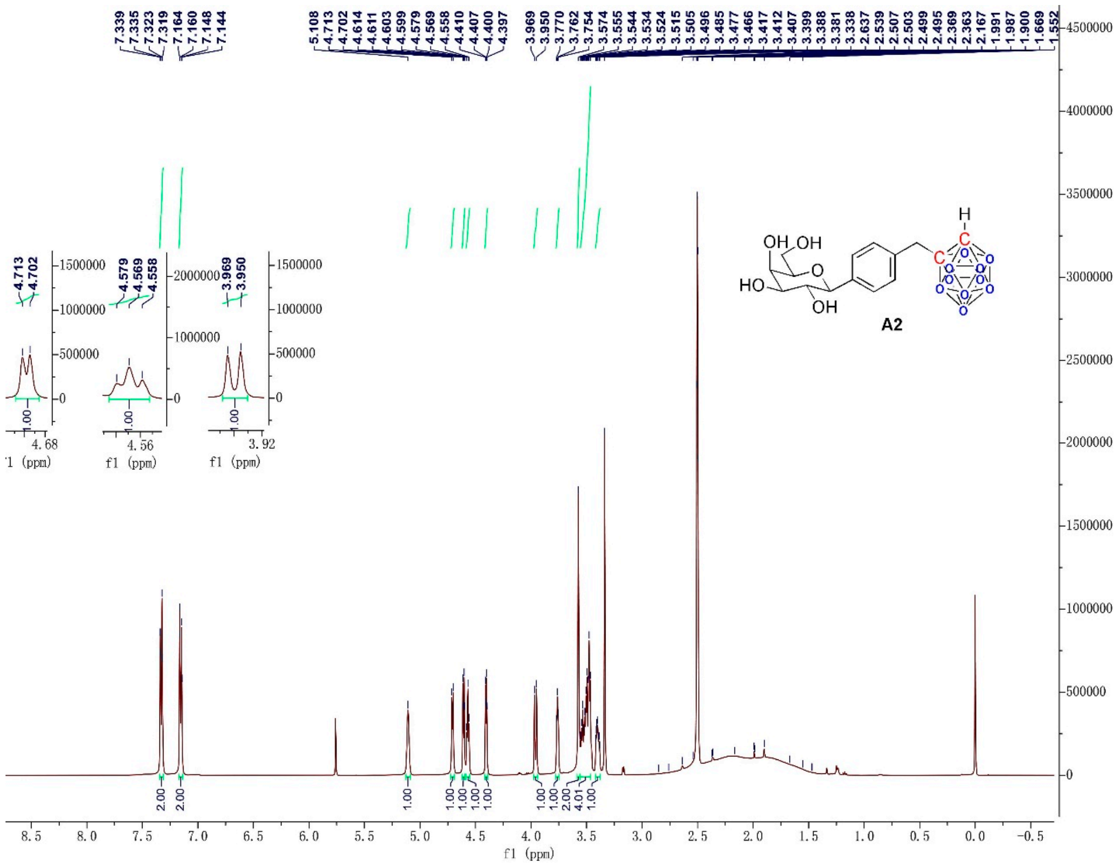

Figure S43 ^{1}H NMR spectrum of **A2**

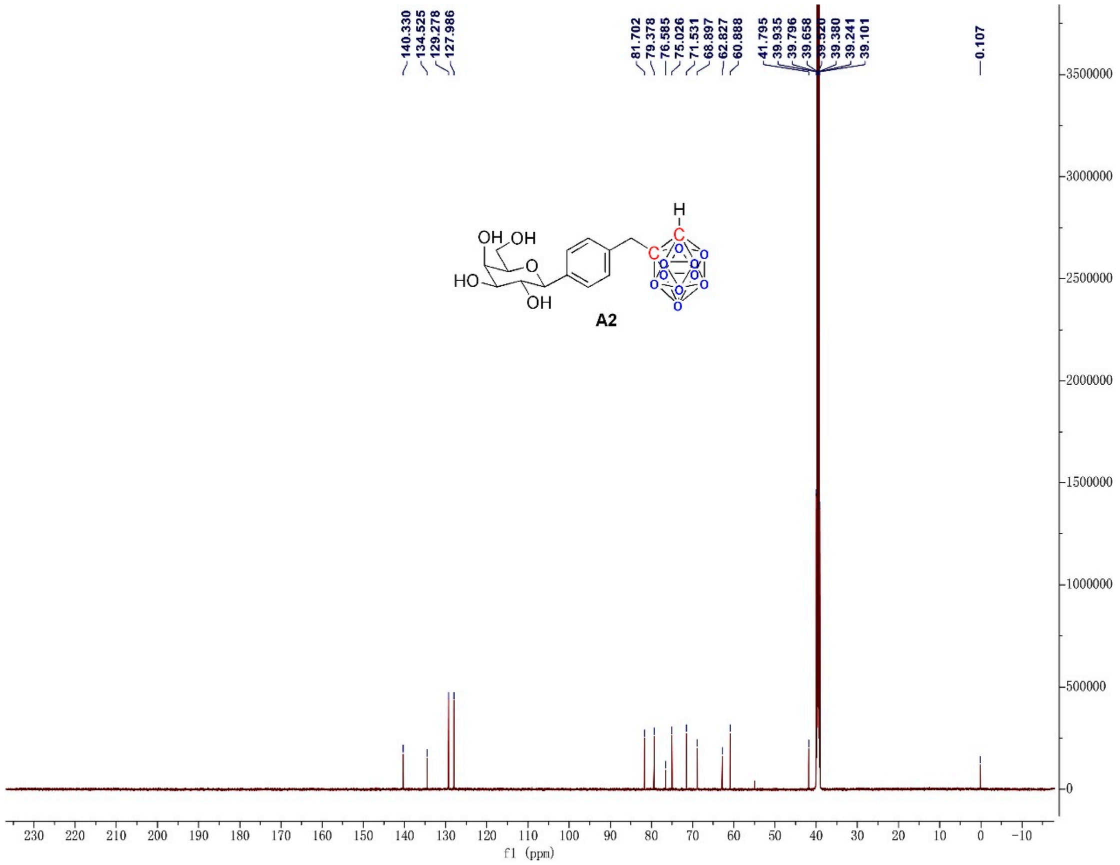

Figure S44 ^{13}C NMR spectrum of **A2**

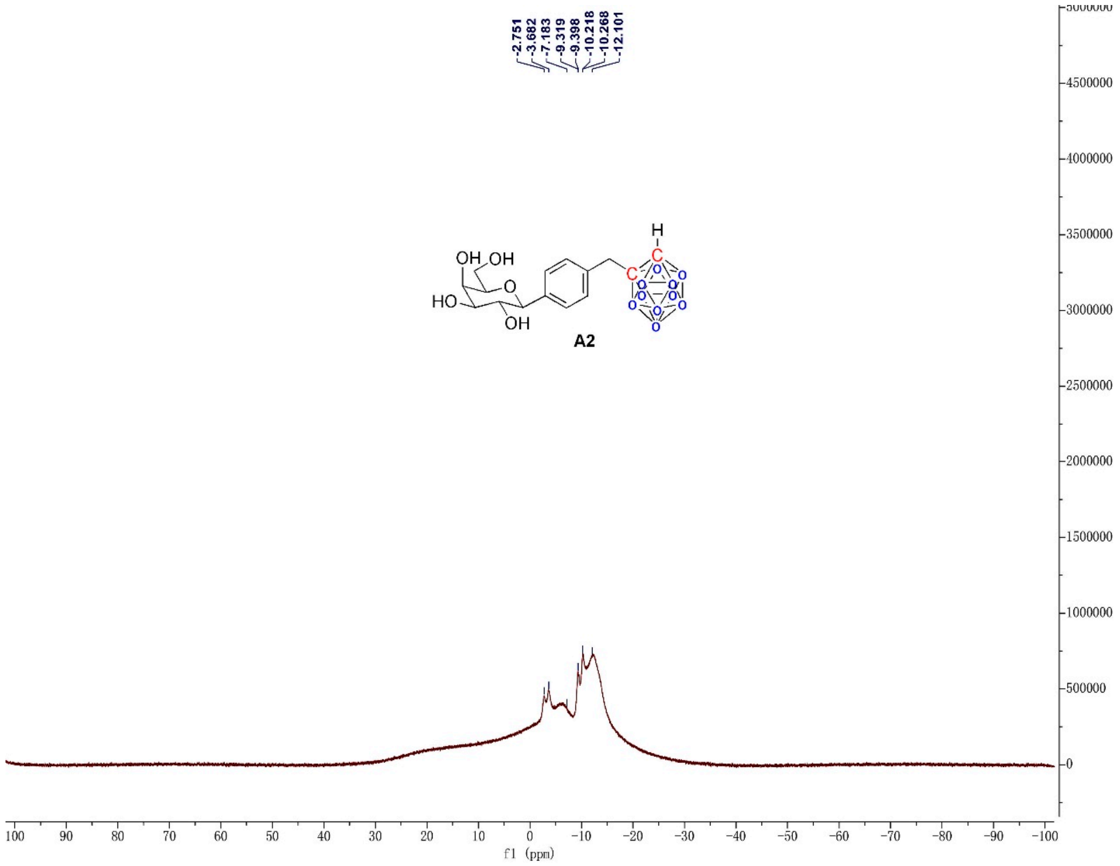

Figure S45 ^{11}B NMR spectrum of **A2**

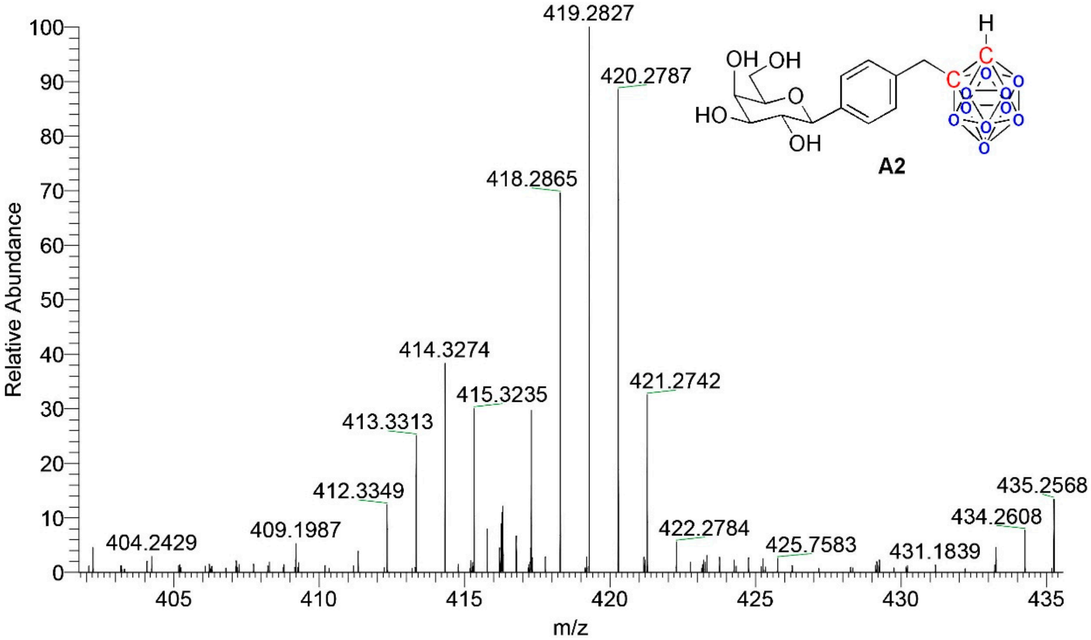

Figure S46 HR-MS (ESI/ion trap) spectrum of **A2**

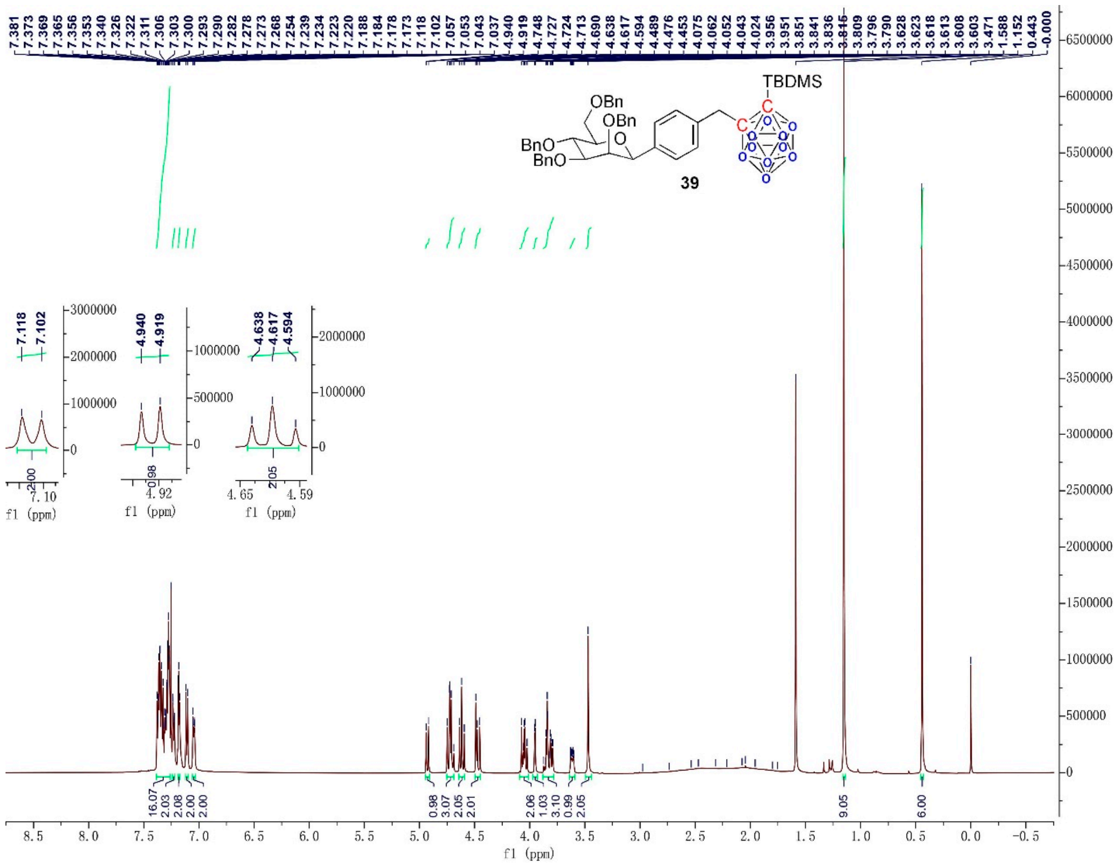

Figure S47 ^{1}H NMR spectrum of **39**

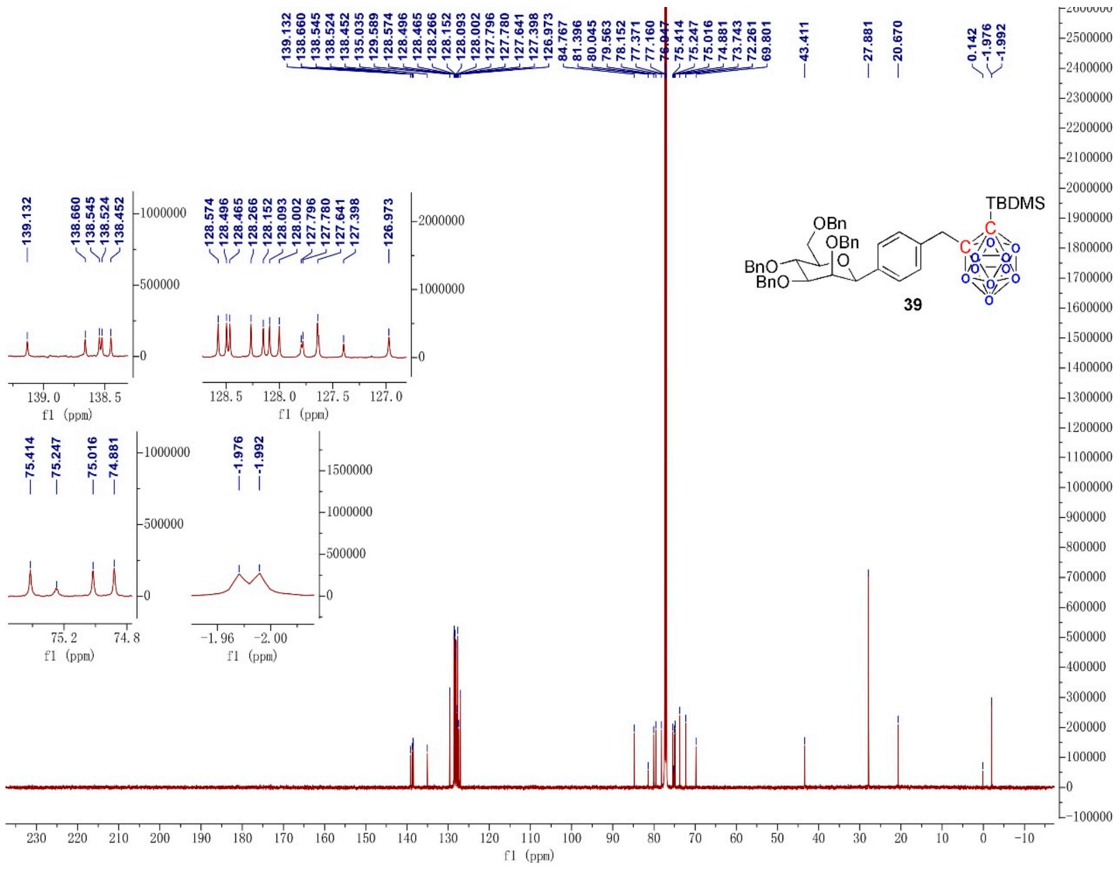

Figure S48 ^{13}C NMR spectrum of **39**

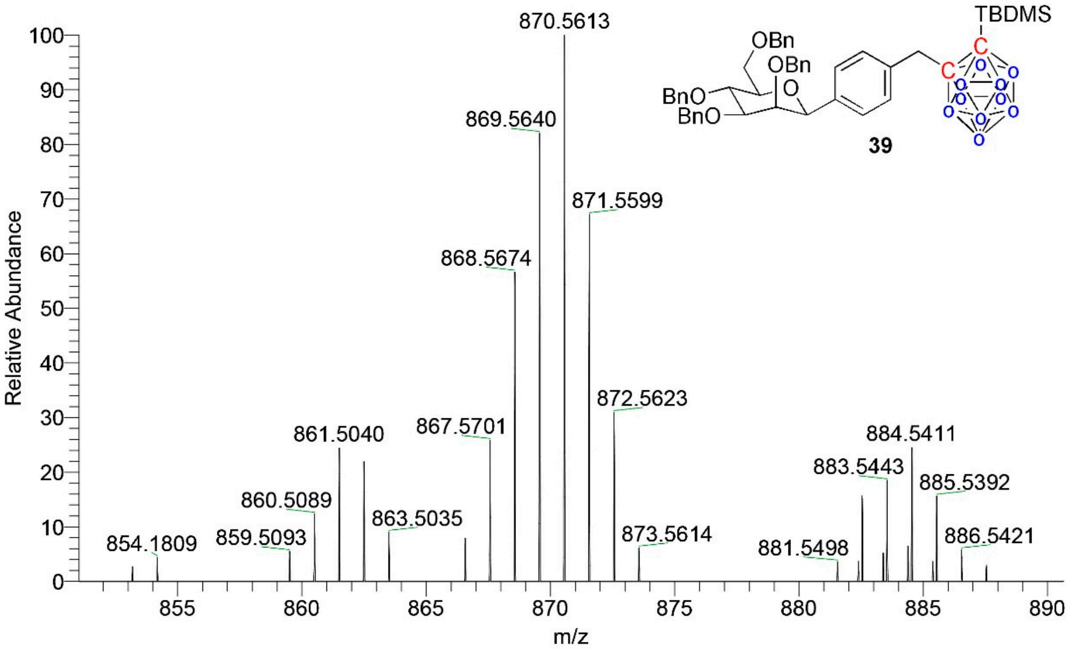

Figure S49 HR-MS (ESI/ion trap) spectrum of **39**

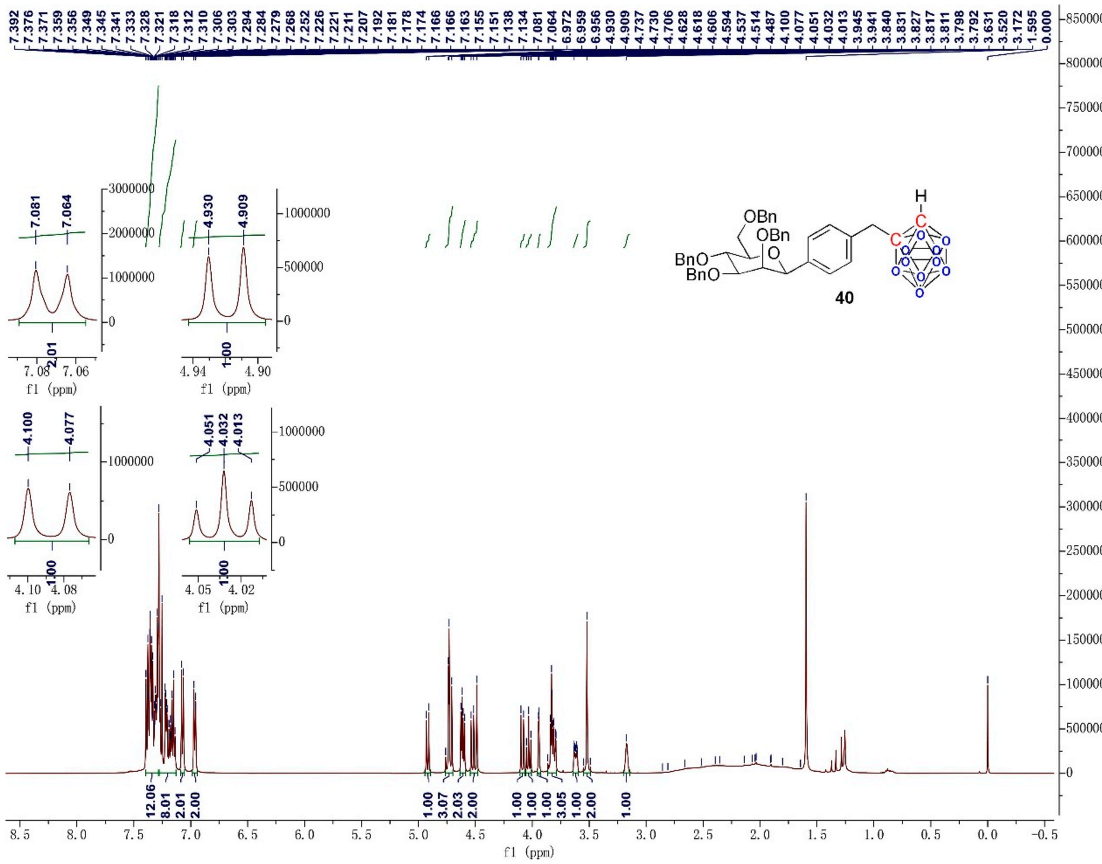

Figure S50 ^{1}H NMR spectrum of **40**

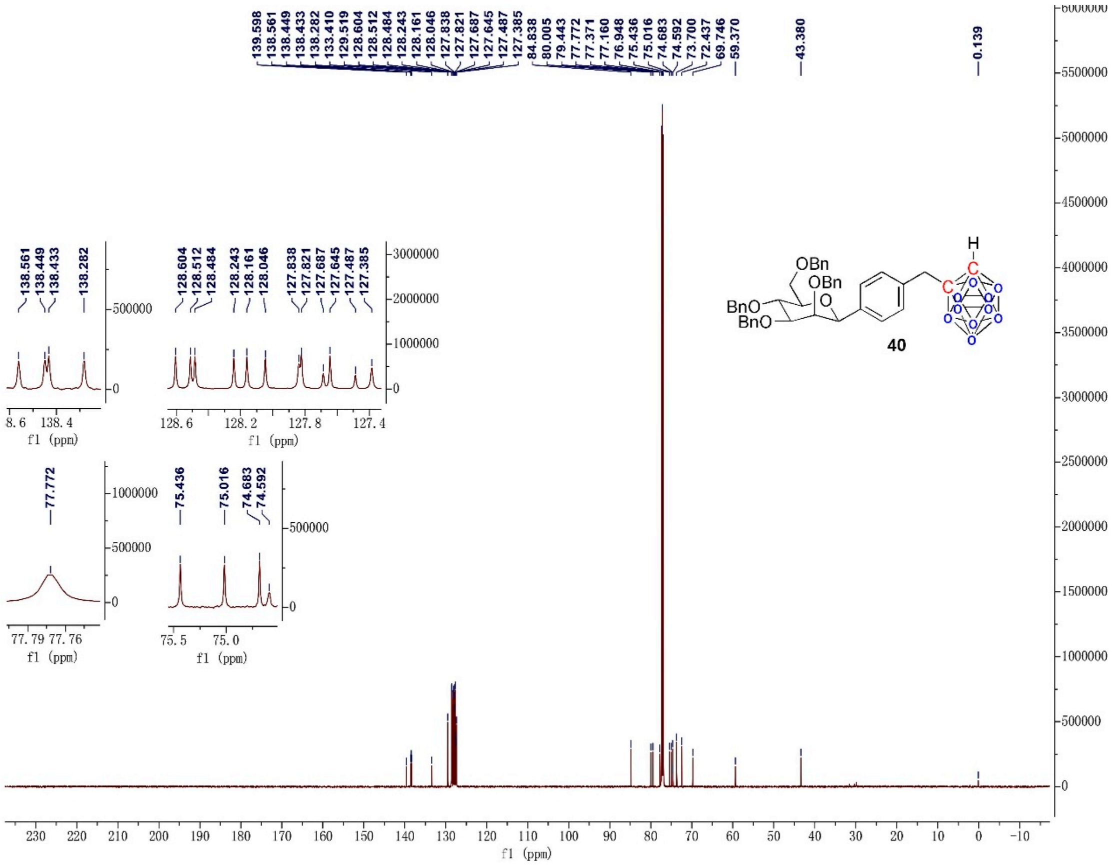

Figure S51 ^{13}C NMR spectrum of **40**

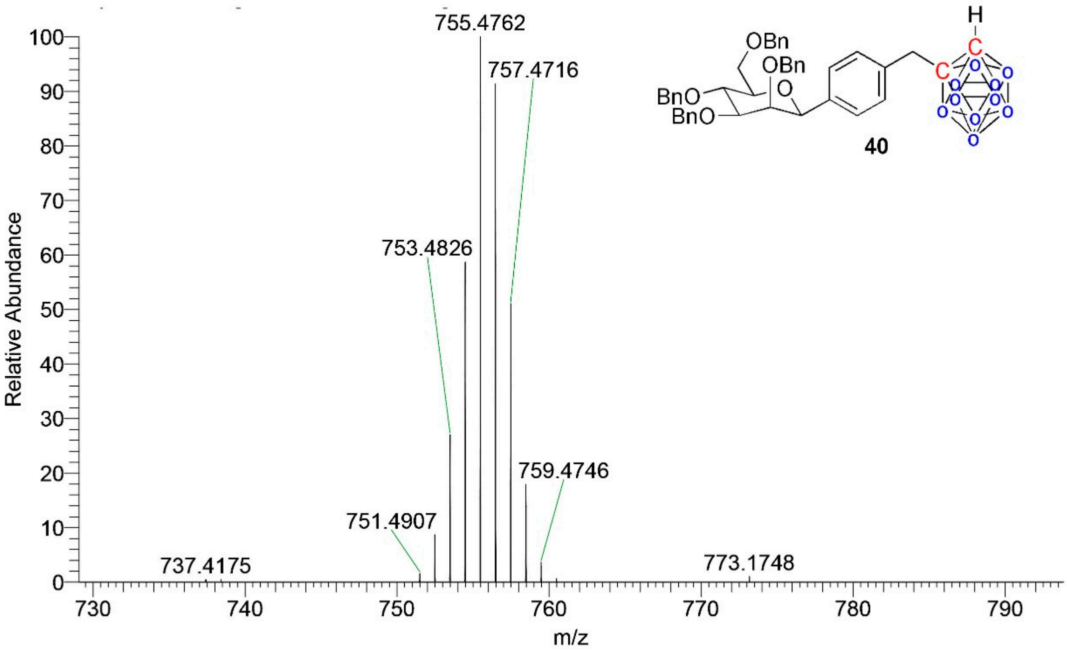

Figure S52 HR-MS (ESI/ion trap) spectrum of **40**

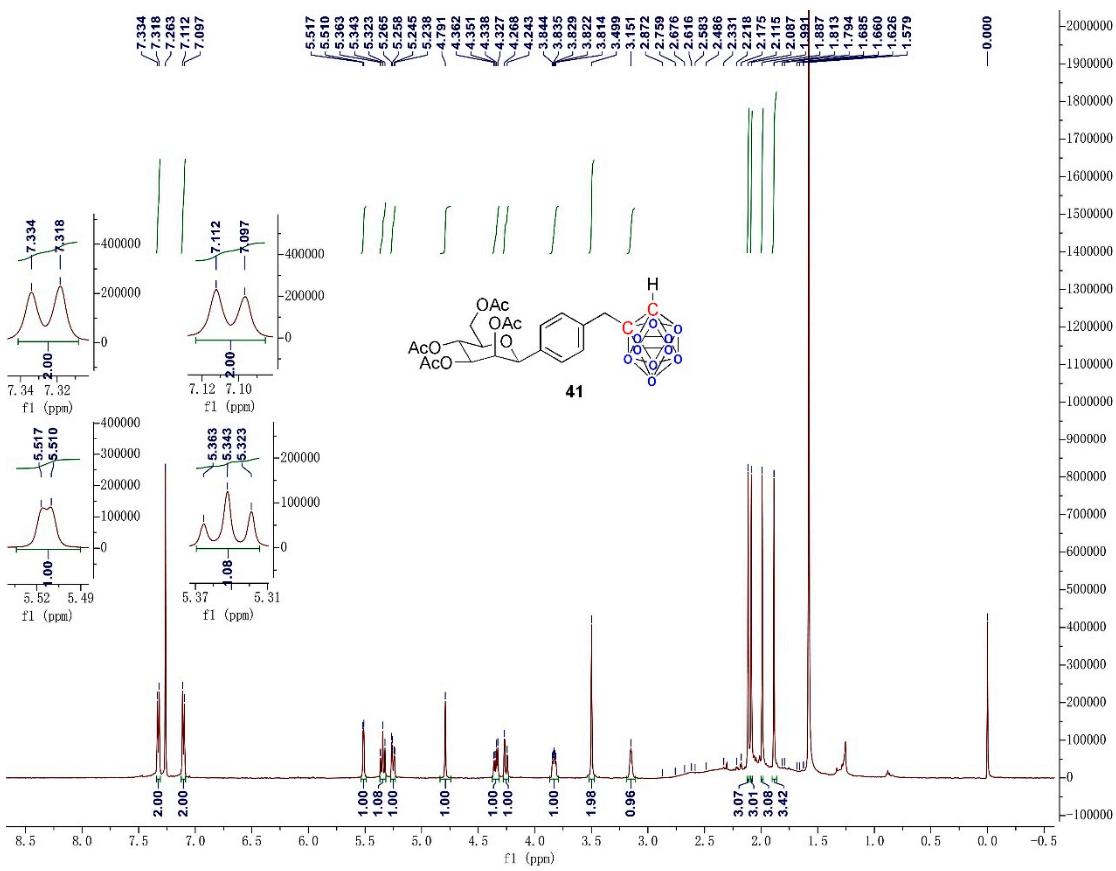

Figure S53 ^{1}H NMR spectrum of **41**

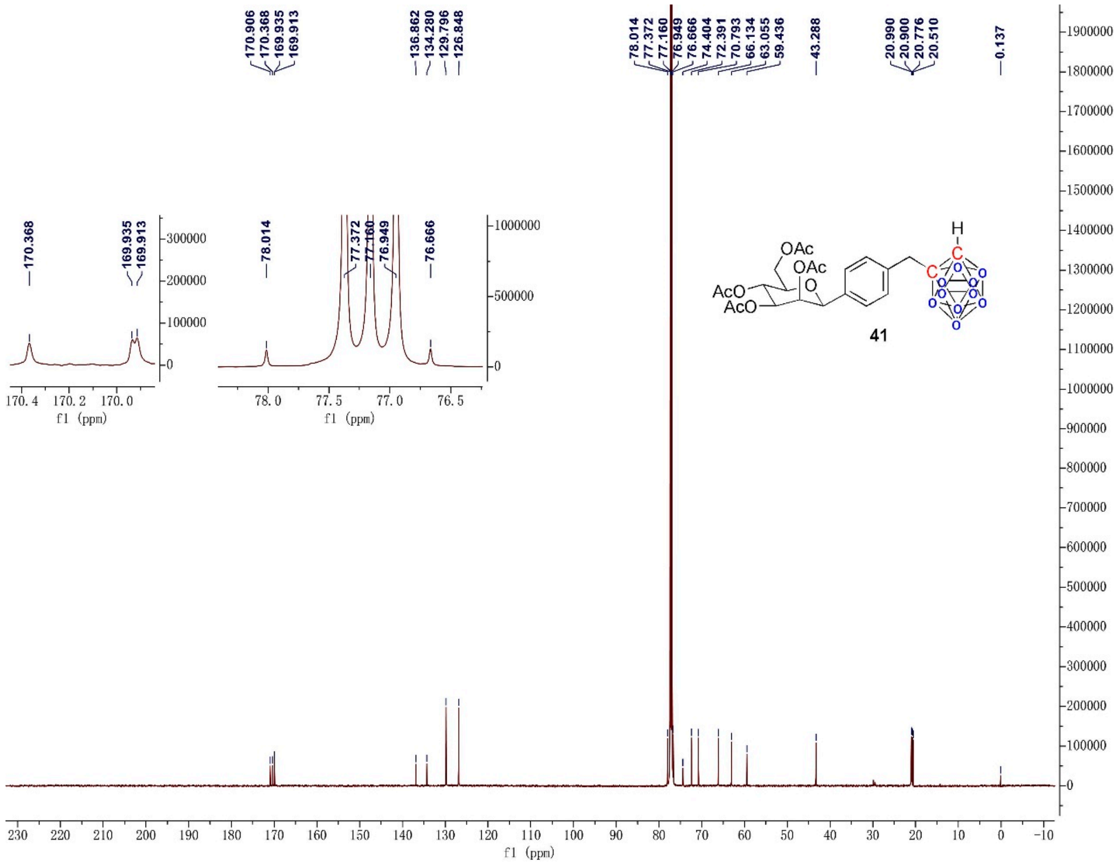

Figure S54 ^{13}C NMR spectrum of **41**

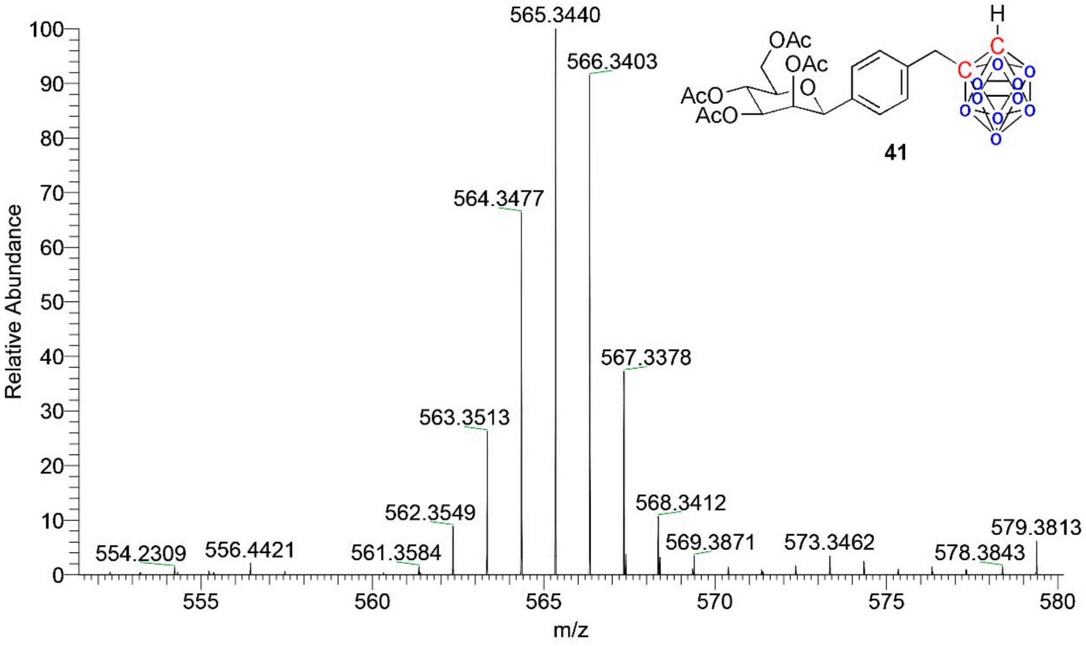

Figure S55 HR-MS (ESI/ion trap) spectrum of **41**

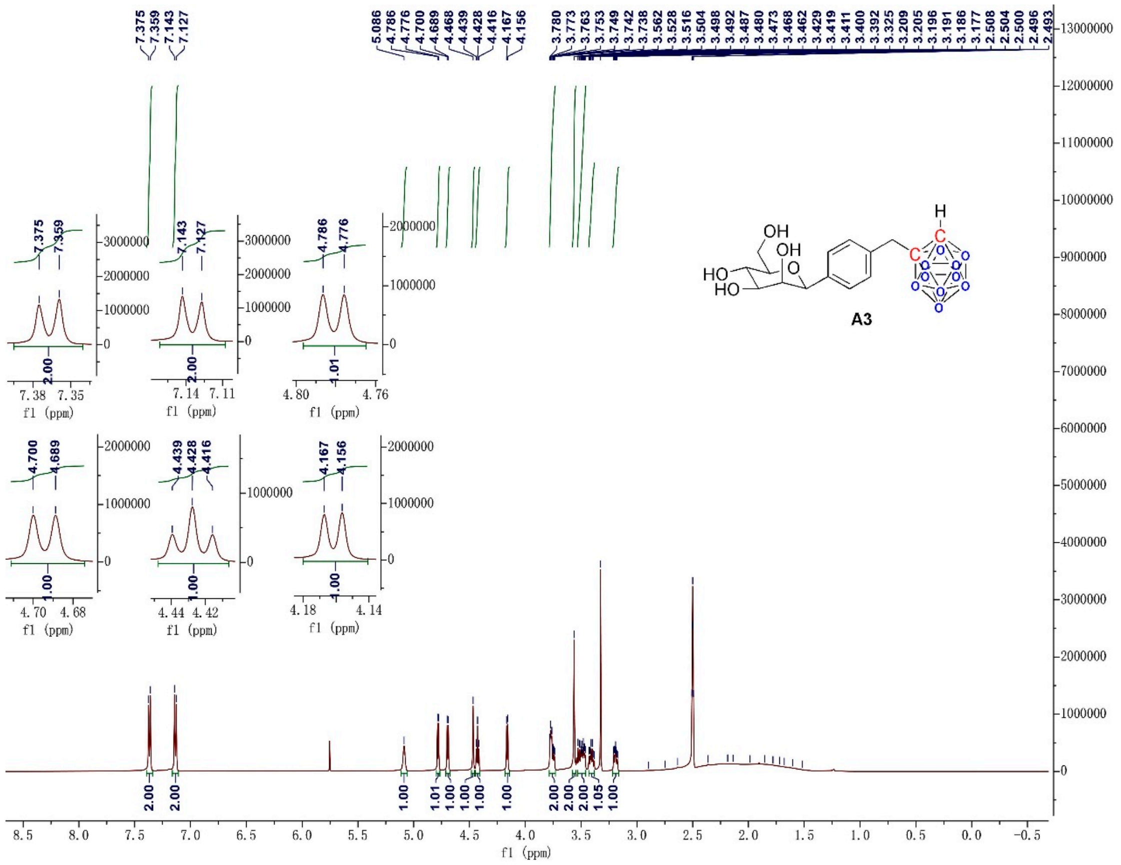

Figure S56 ^{1}H NMR spectrum of **A3**

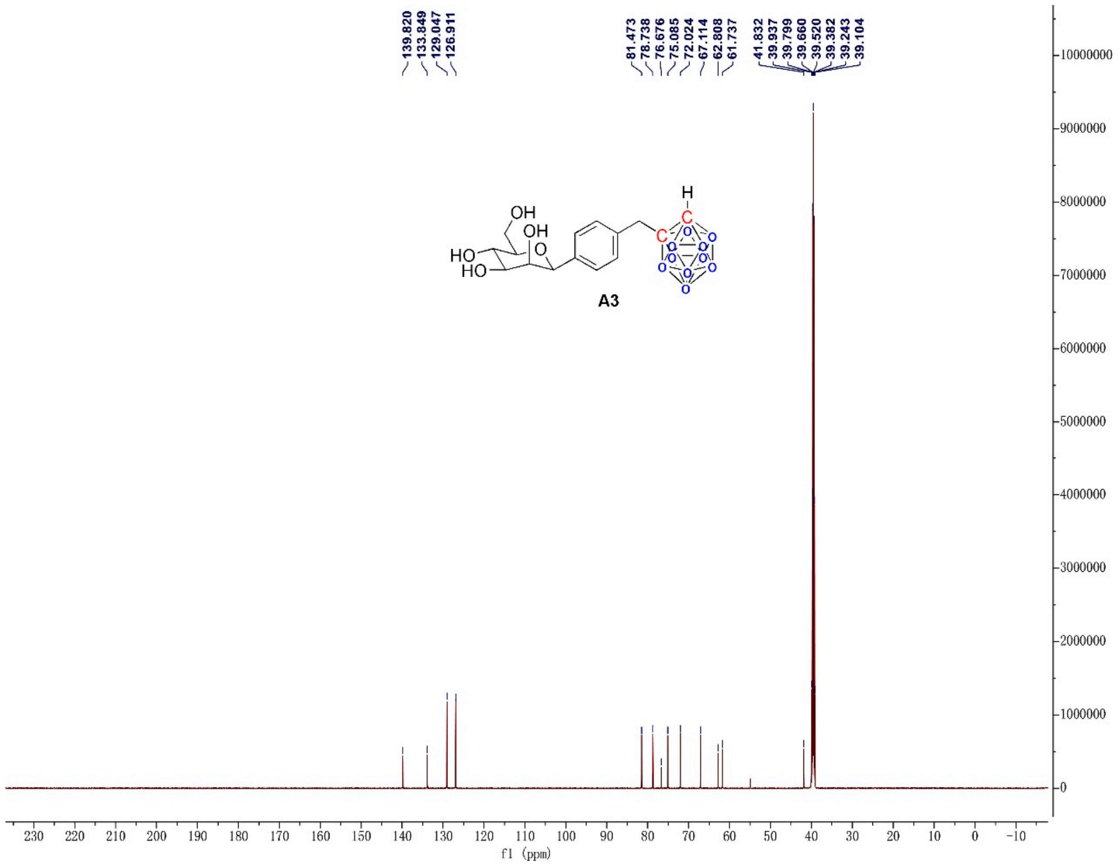

Figure S57 ^{13}C NMR spectrum of **A3**

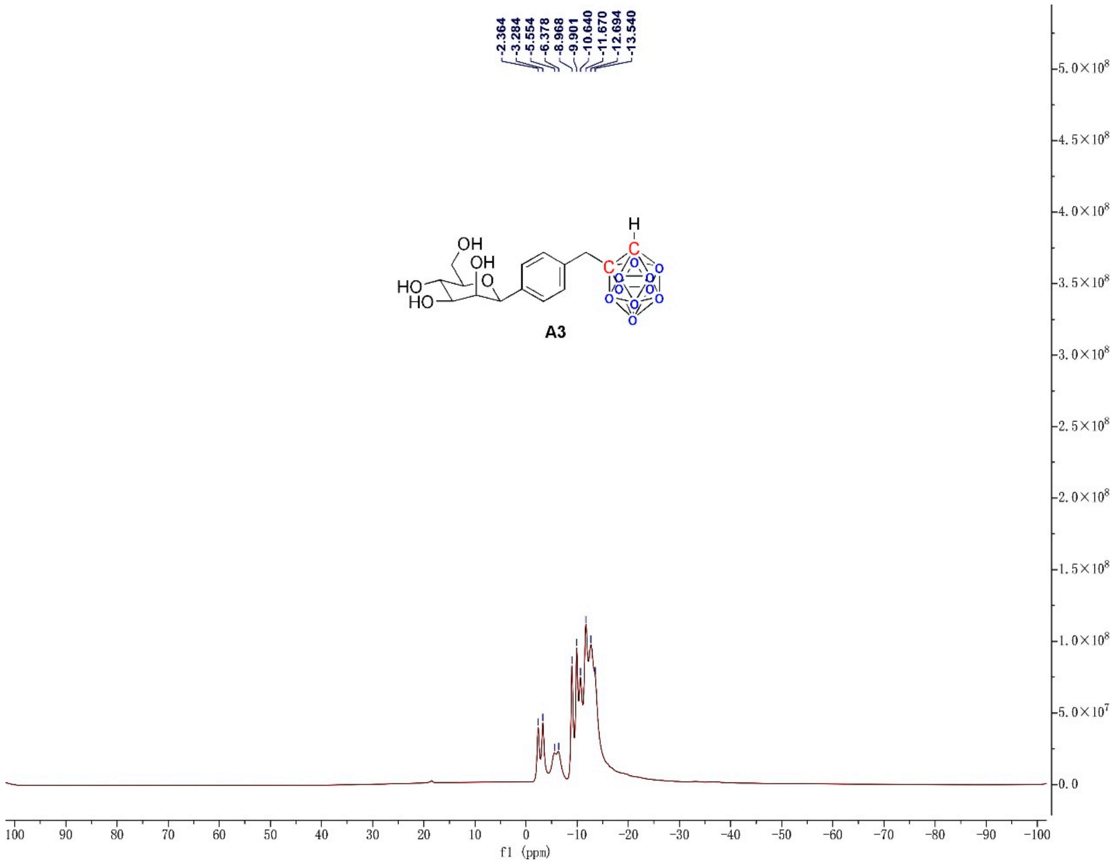

Figure S58 ^{11}B NMR spectrum of **A3**

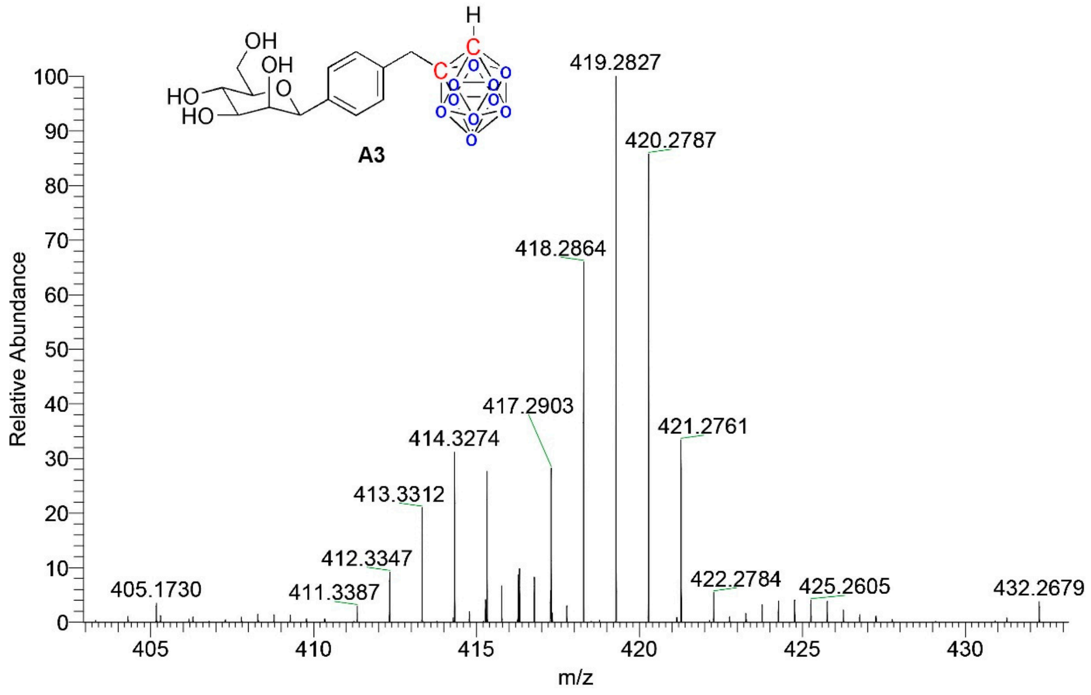

Figure S59 HR-MS (ESI/ion trap) spectrum of **A3**

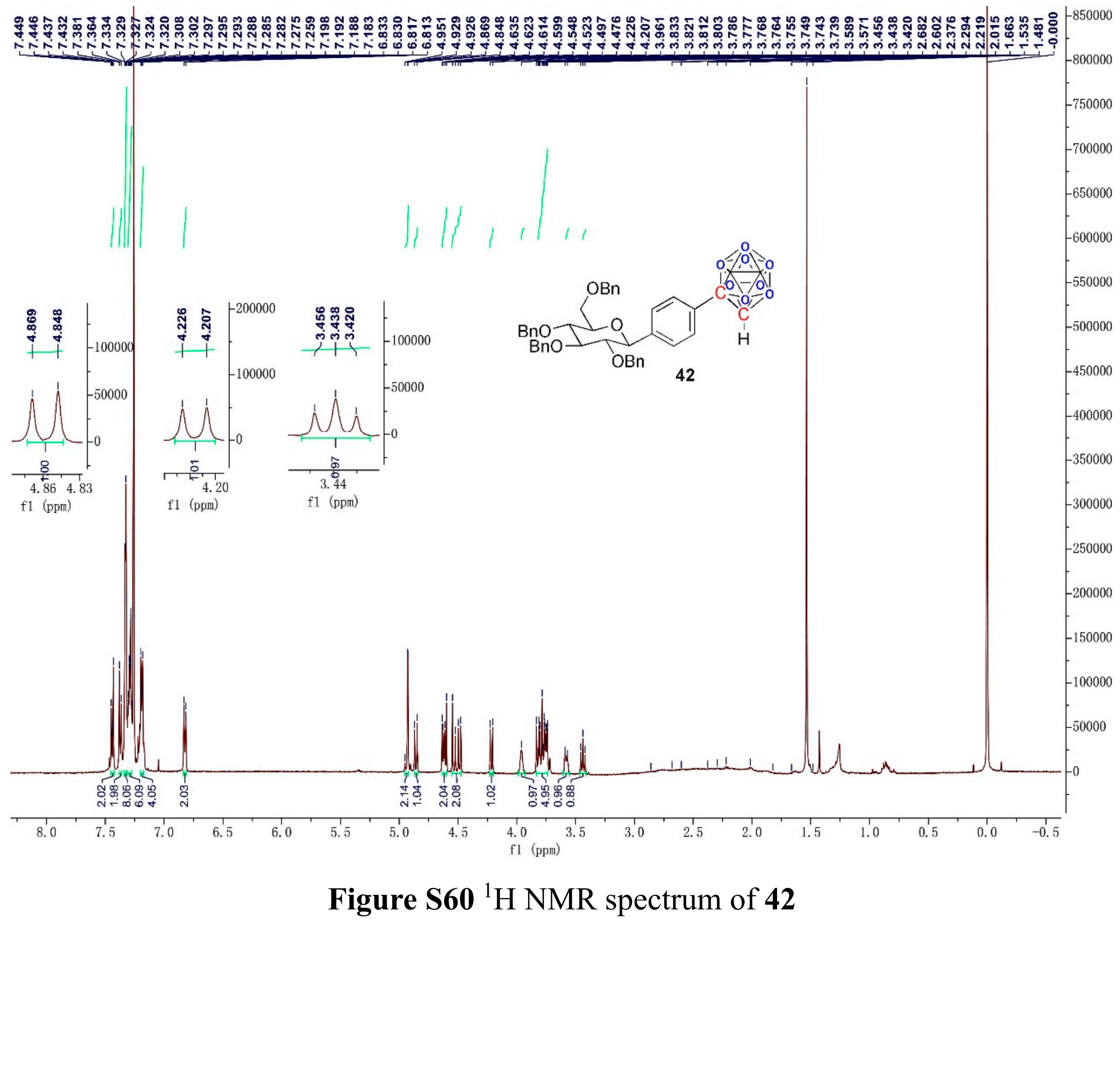

Figure S60 ^{1}H NMR spectrum of **42**

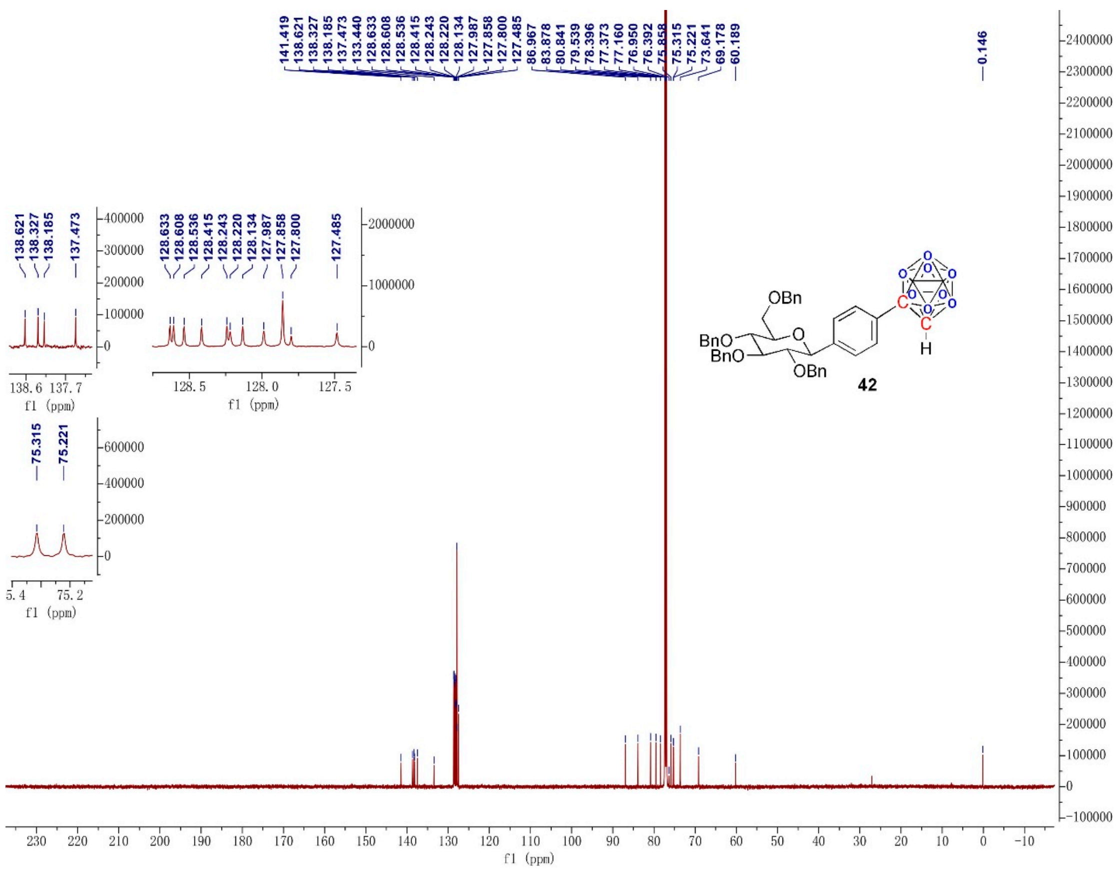

Figure S61 ^{13}C NMR spectrum of **42**

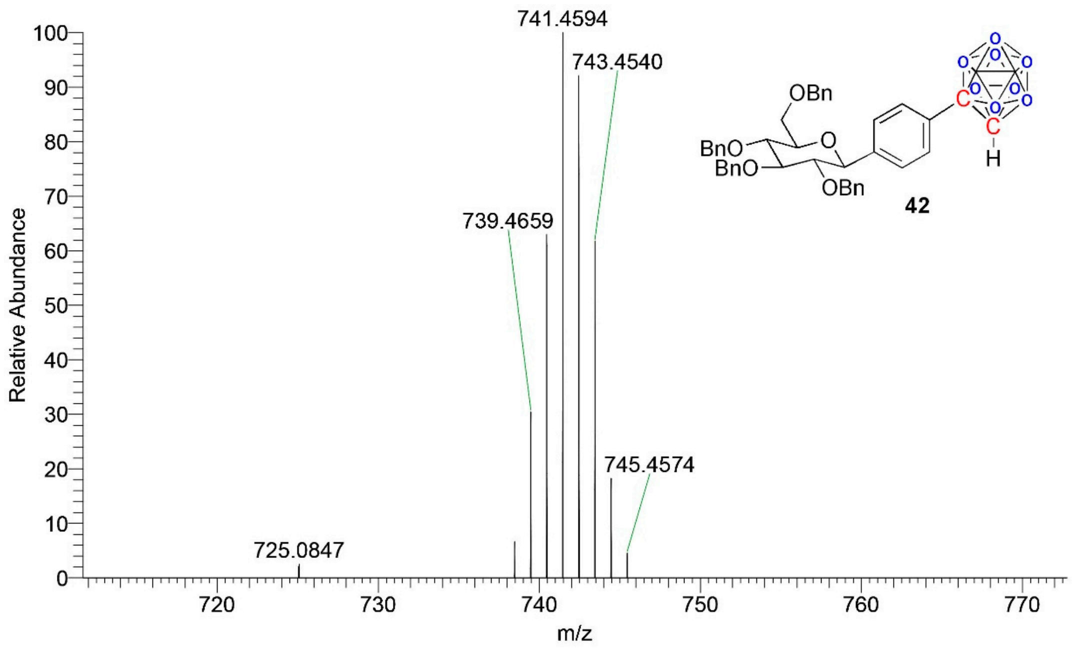

Figure S62 HR-MS (ESI/ion trap) spectrum of **42**

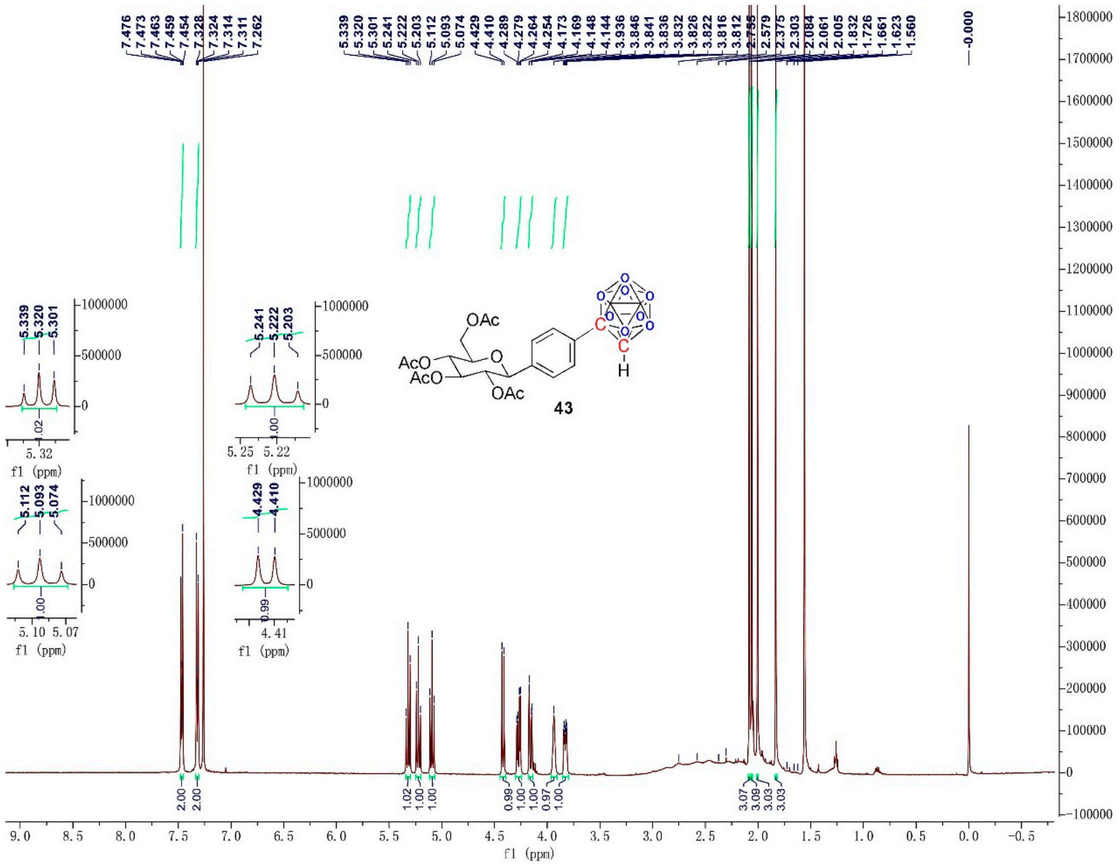

Figure S63 ^{1}H NMR spectrum of **43**

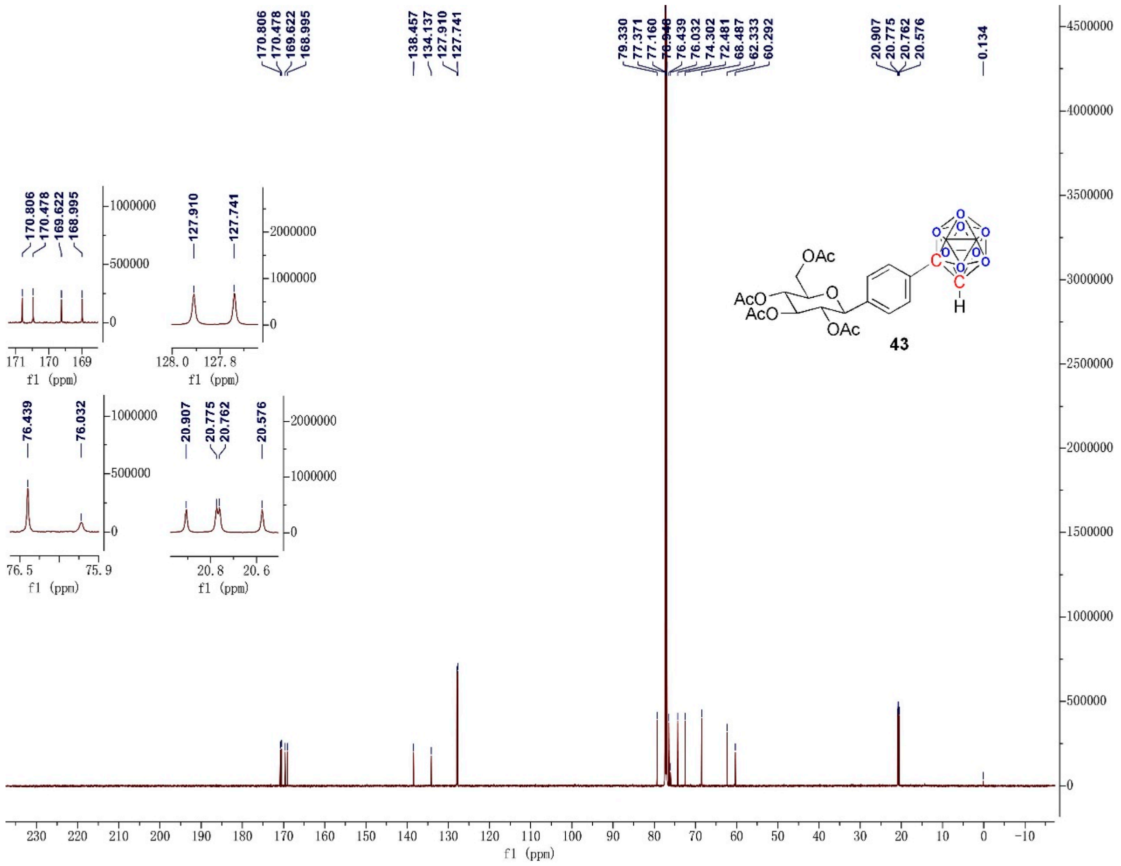

Figure S64 ^{13}C NMR spectrum of **43**

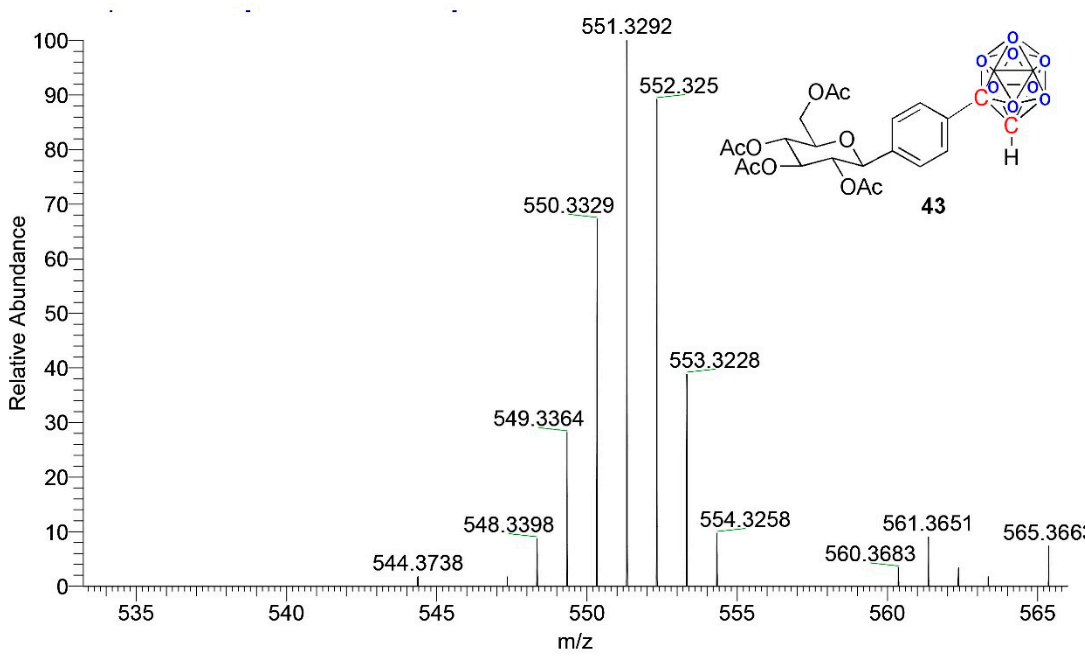

Figure S65 HR-MS (ESI/ion trap) spectrum of **43**

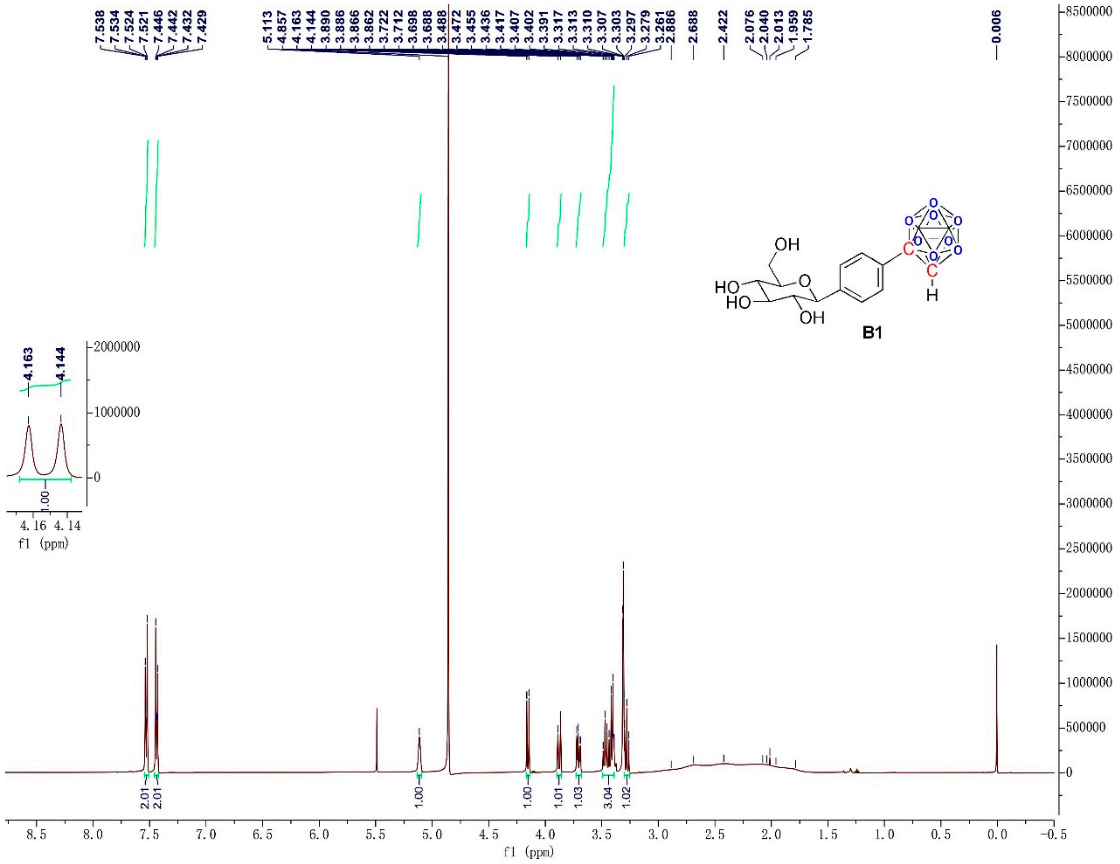

Figure S66 ^{1}H NMR spectrum of **B1**

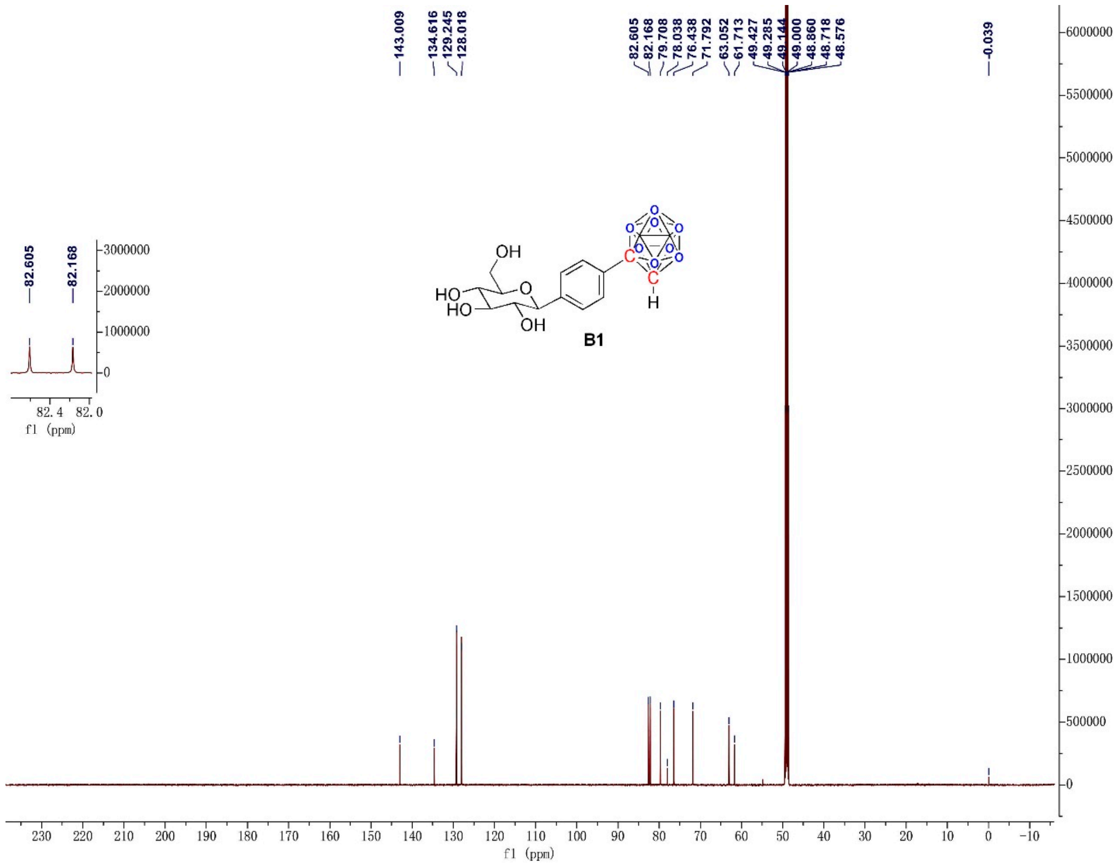

Figure S67 ^{13}C NMR spectrum of **B1**

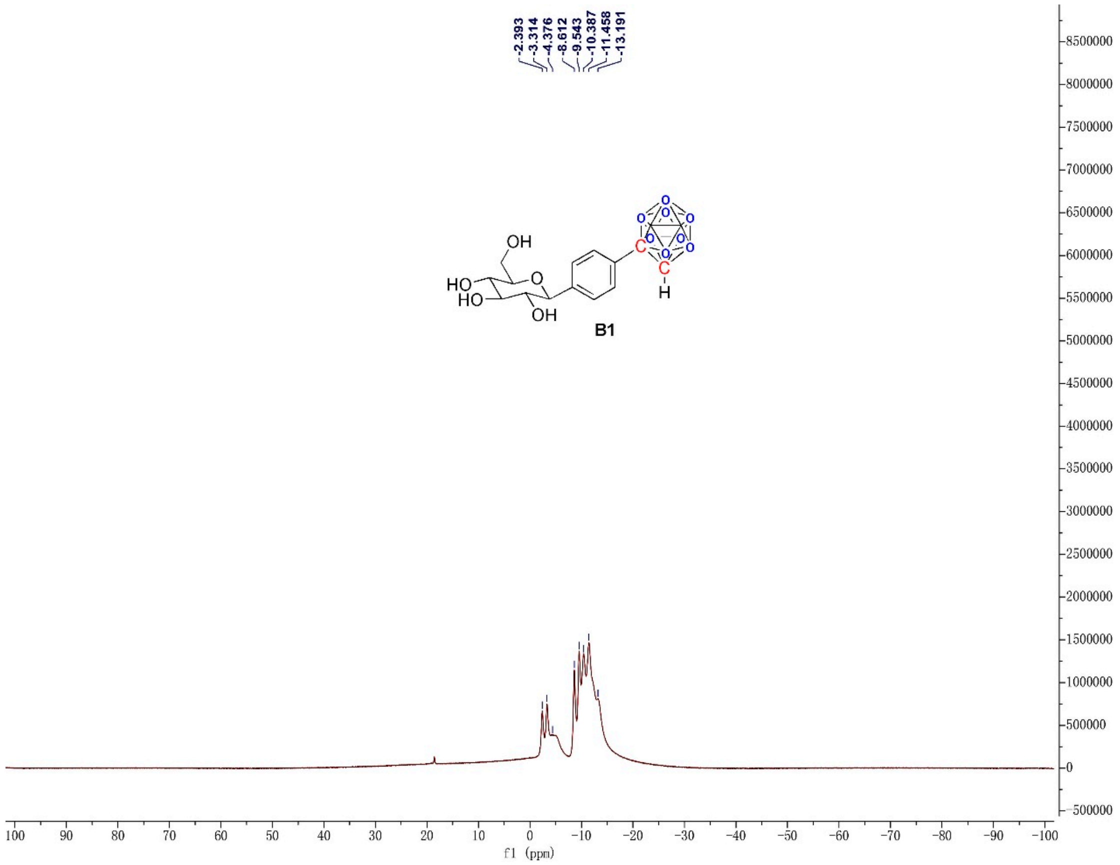

Figure S68 ^{11}B NMR spectrum of **B1**

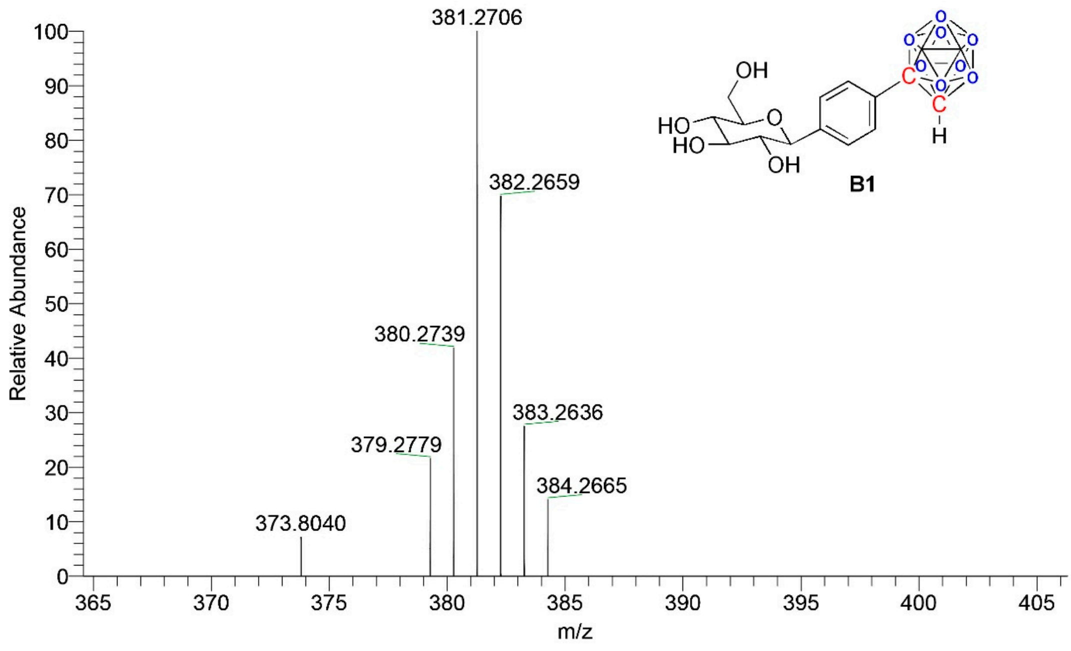

Figure S69 HR-MS (ESI/ion trap) spectrum of **B1**

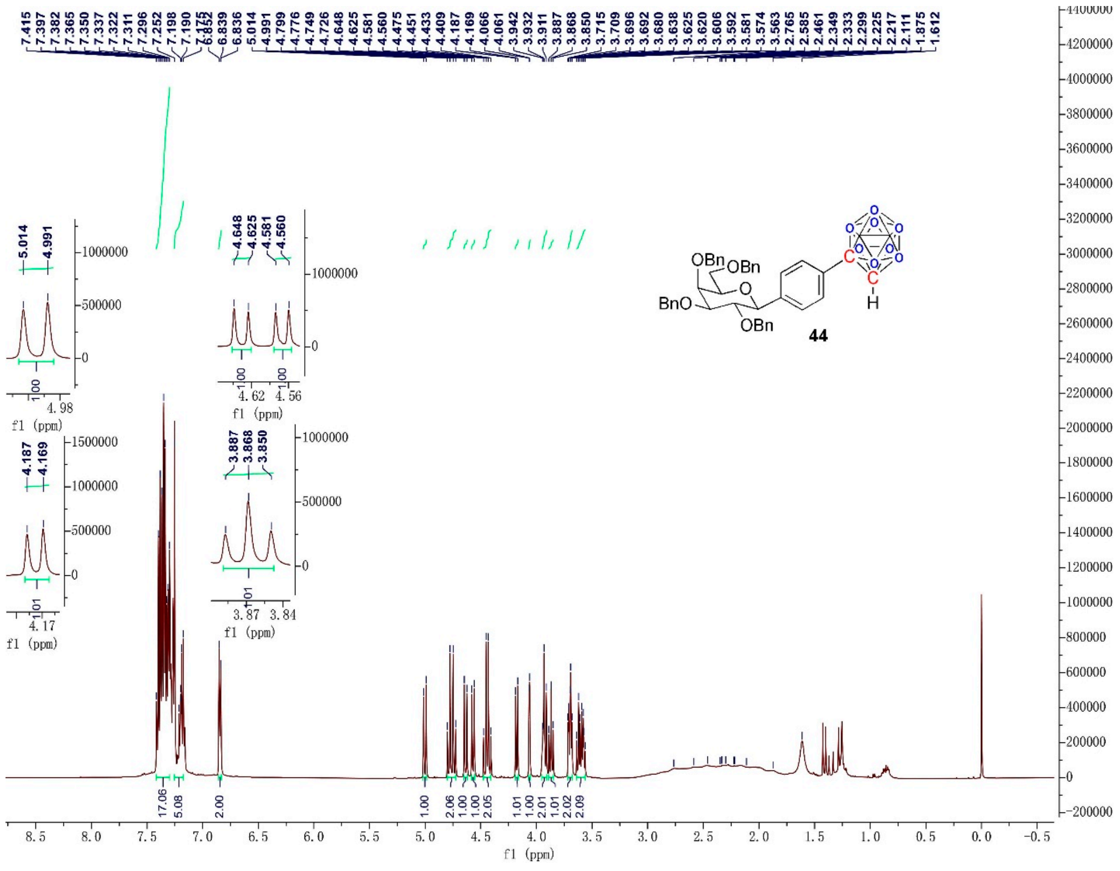

Figure S70 ^{1}H NMR spectrum of **44**

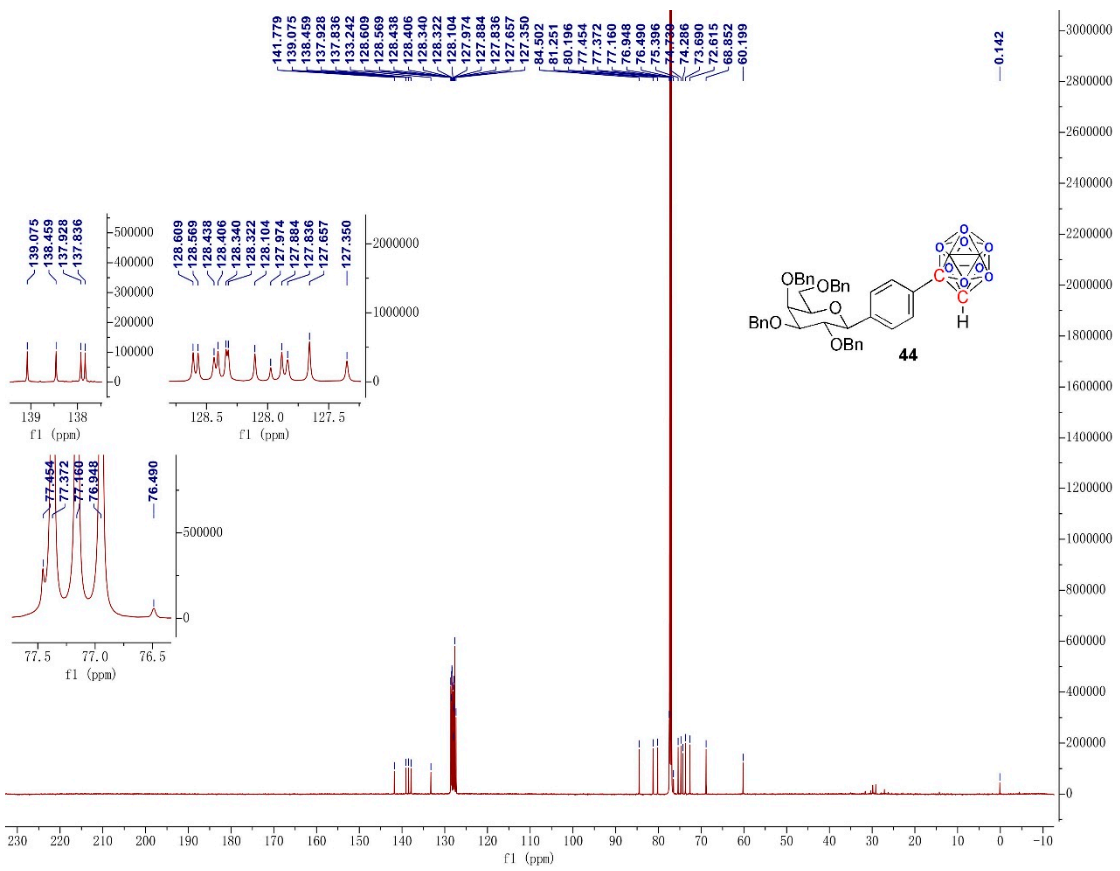

Figure S71 ^{13}C NMR spectrum of **44**

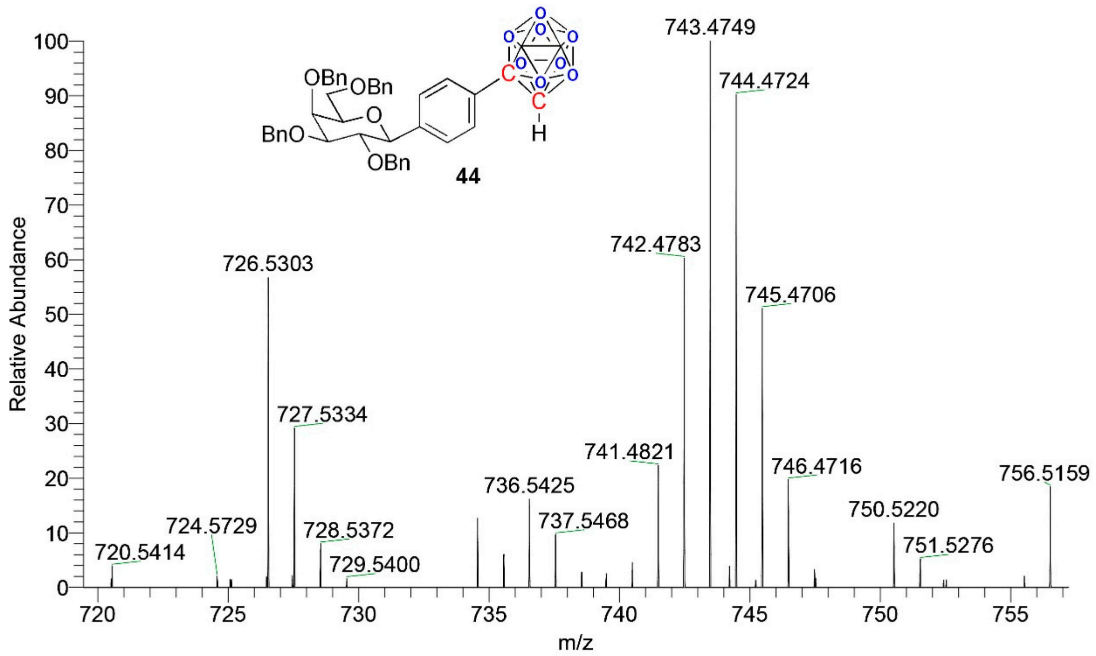

Figure S72 HR-MS (ESI/ion trap) spectrum of **44**

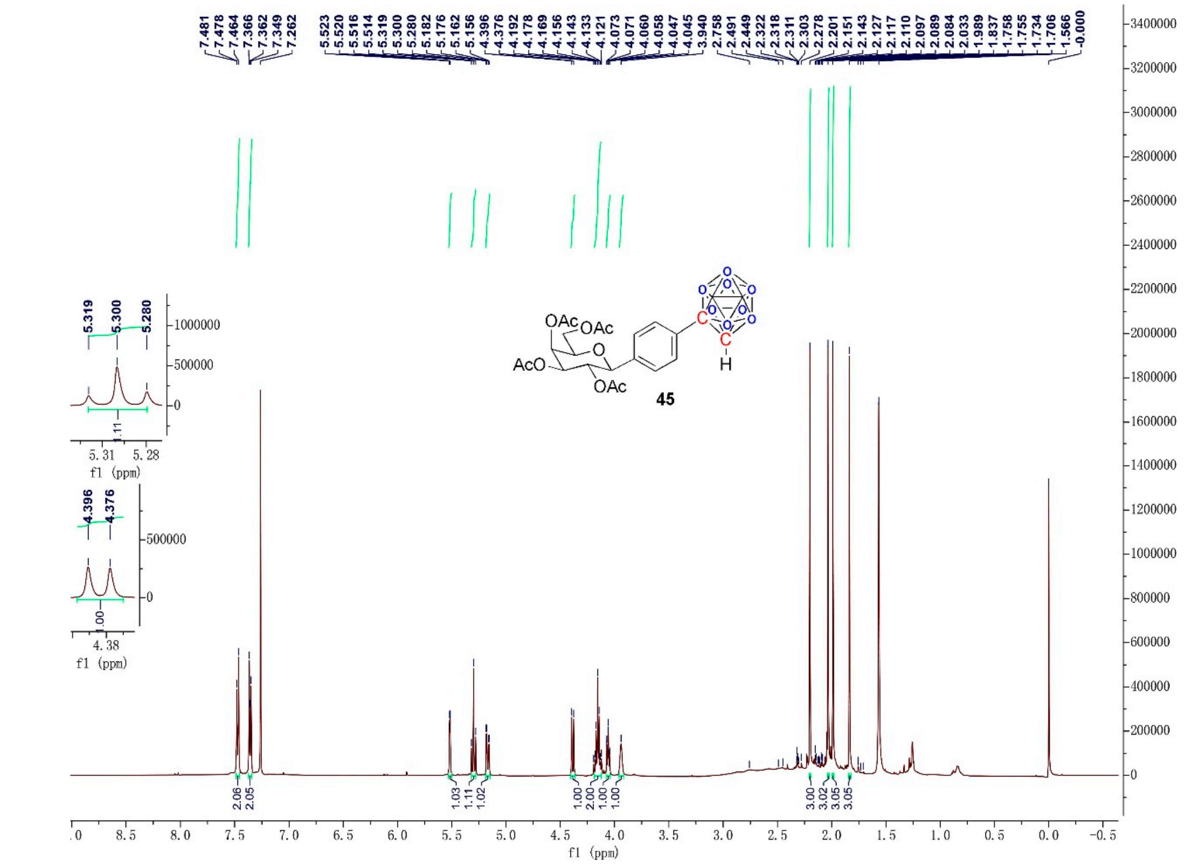

Figure S73 ^{1}H NMR spectrum of **45**

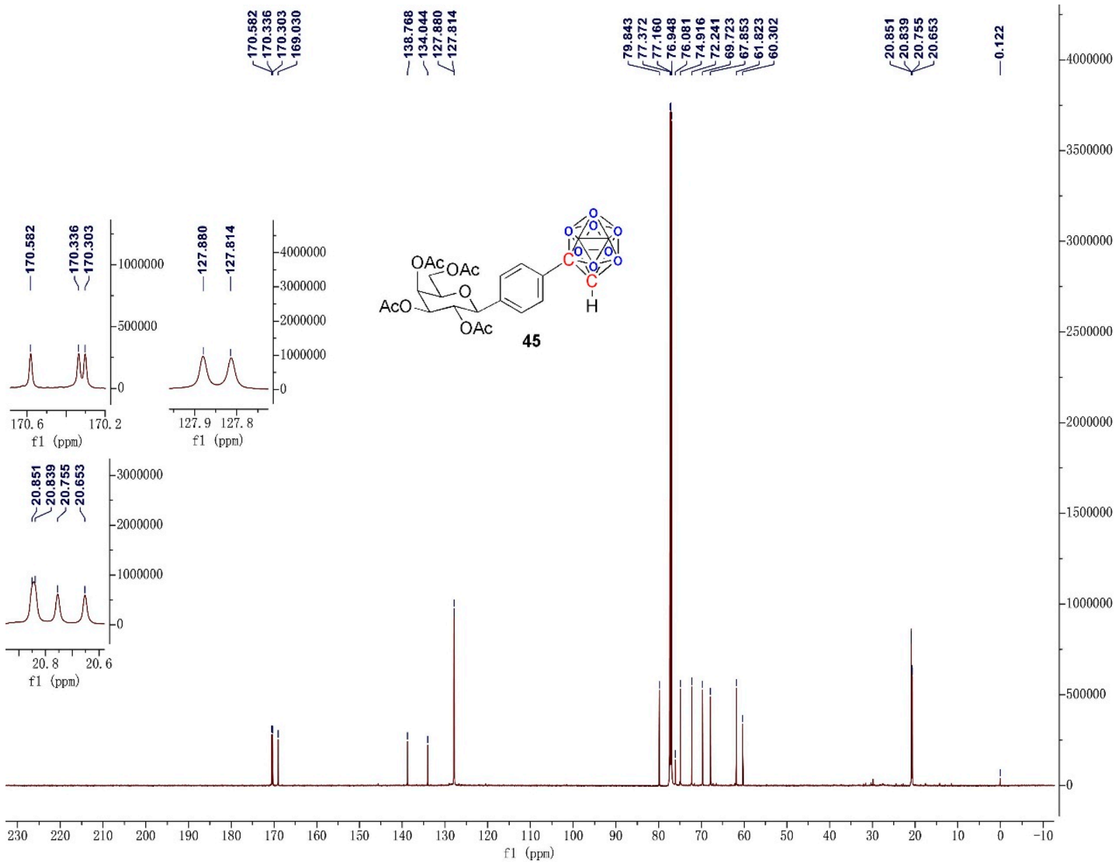

Figure S74 ^{13}C NMR spectrum of **45**

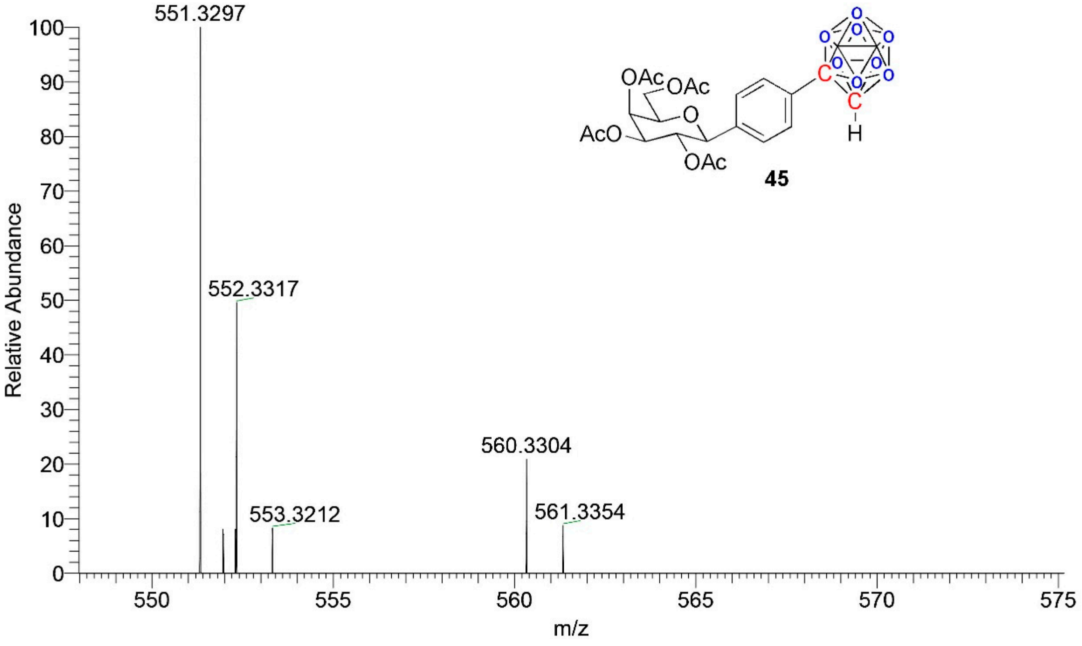

Figure S75 HR-MS (ESI/ion trap) spectrum of **45**

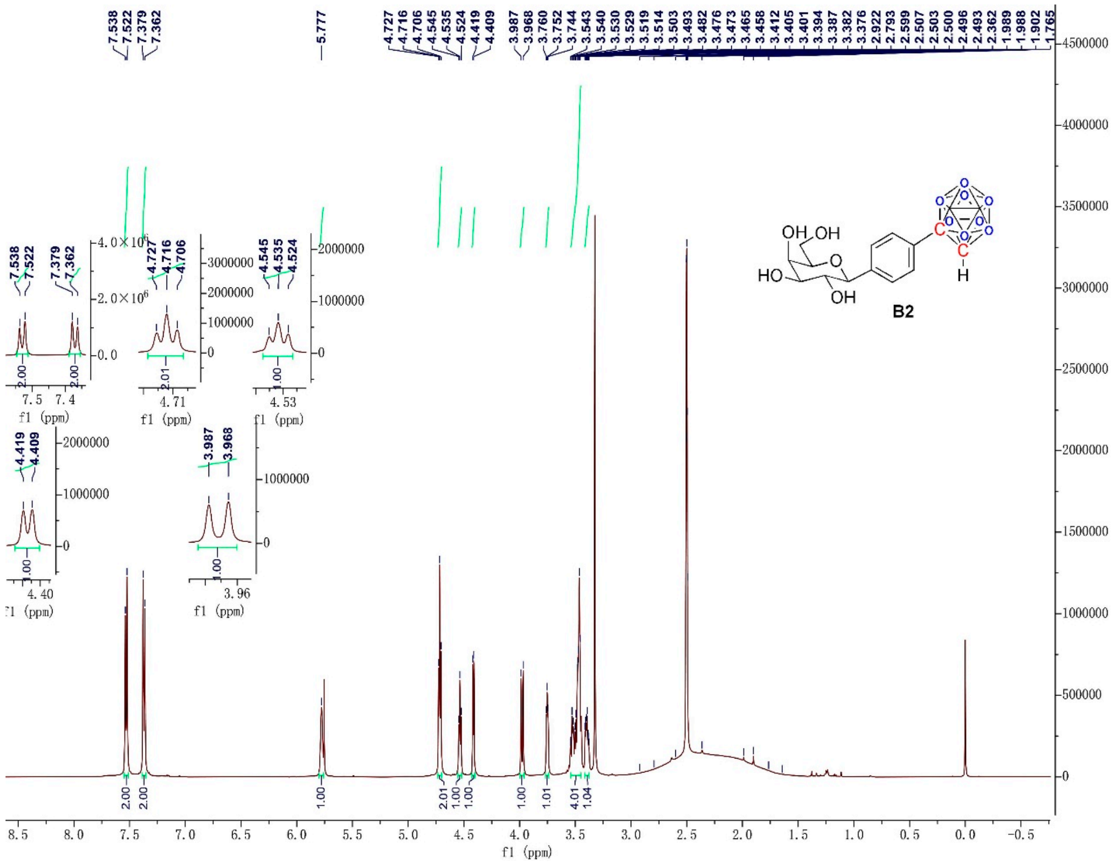

Figure S76 ^{1}H NMR spectrum of **B2**

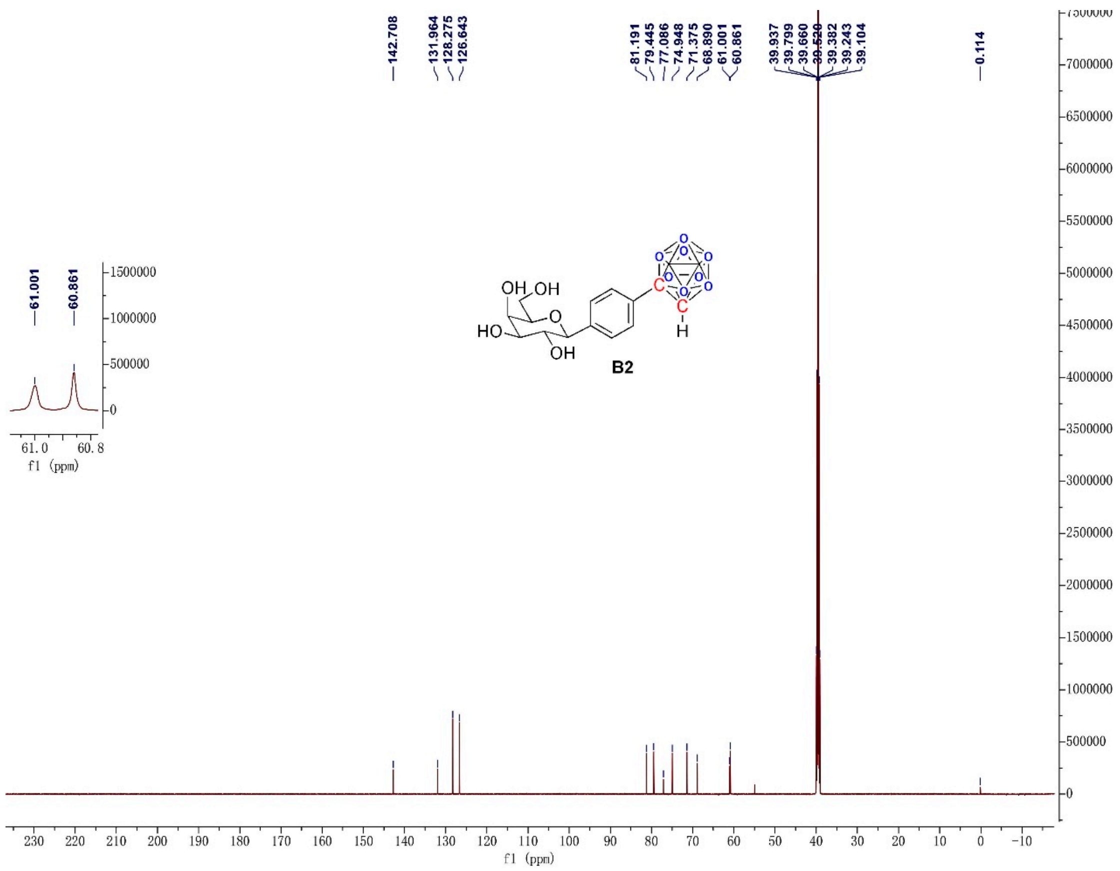

Figure S77 ^{13}C NMR spectrum of **B2**

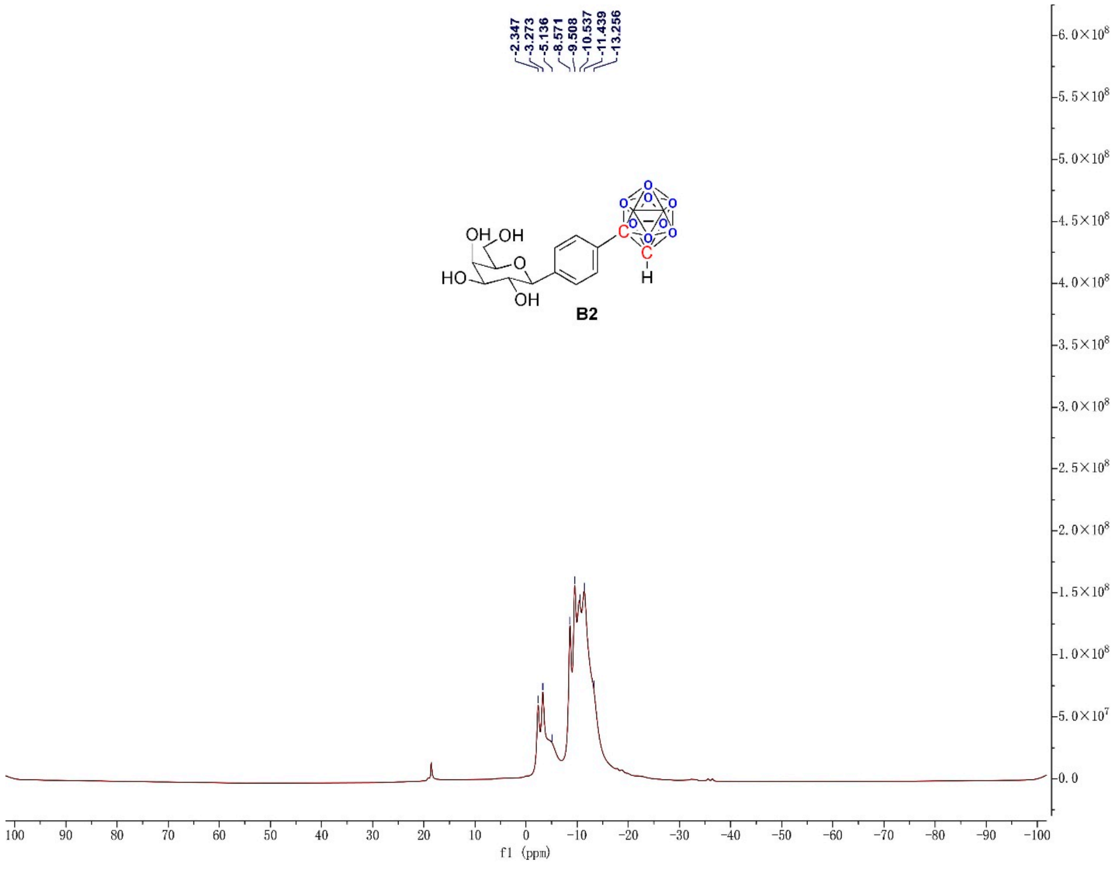

Figure S78 ^{11}B NMR spectrum of **B2**

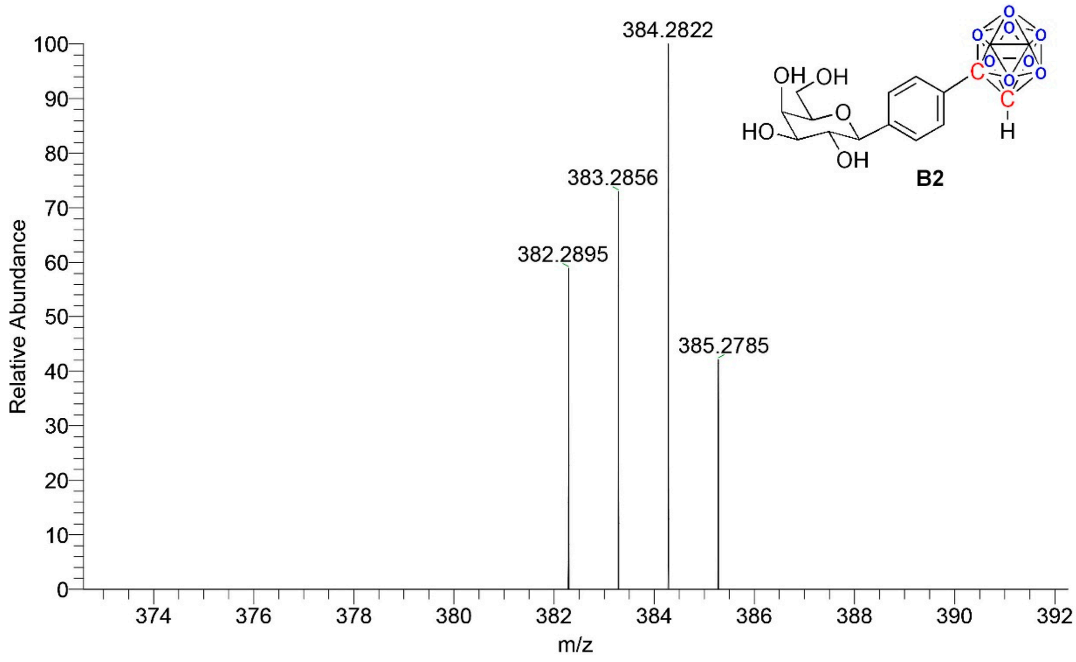

Figure S79 HR-MS (ESI/ion trap) spectrum of **B2**

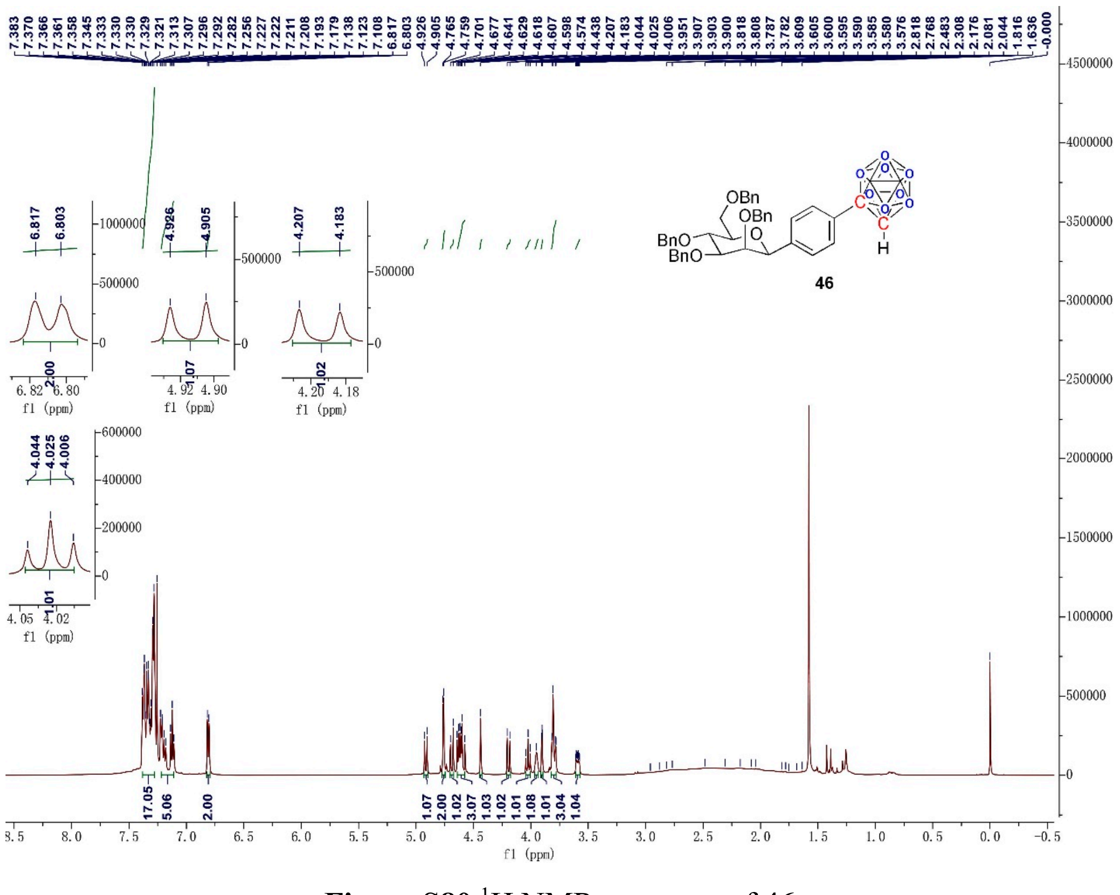

Figure S80 ^{1}H NMR spectrum of **46**

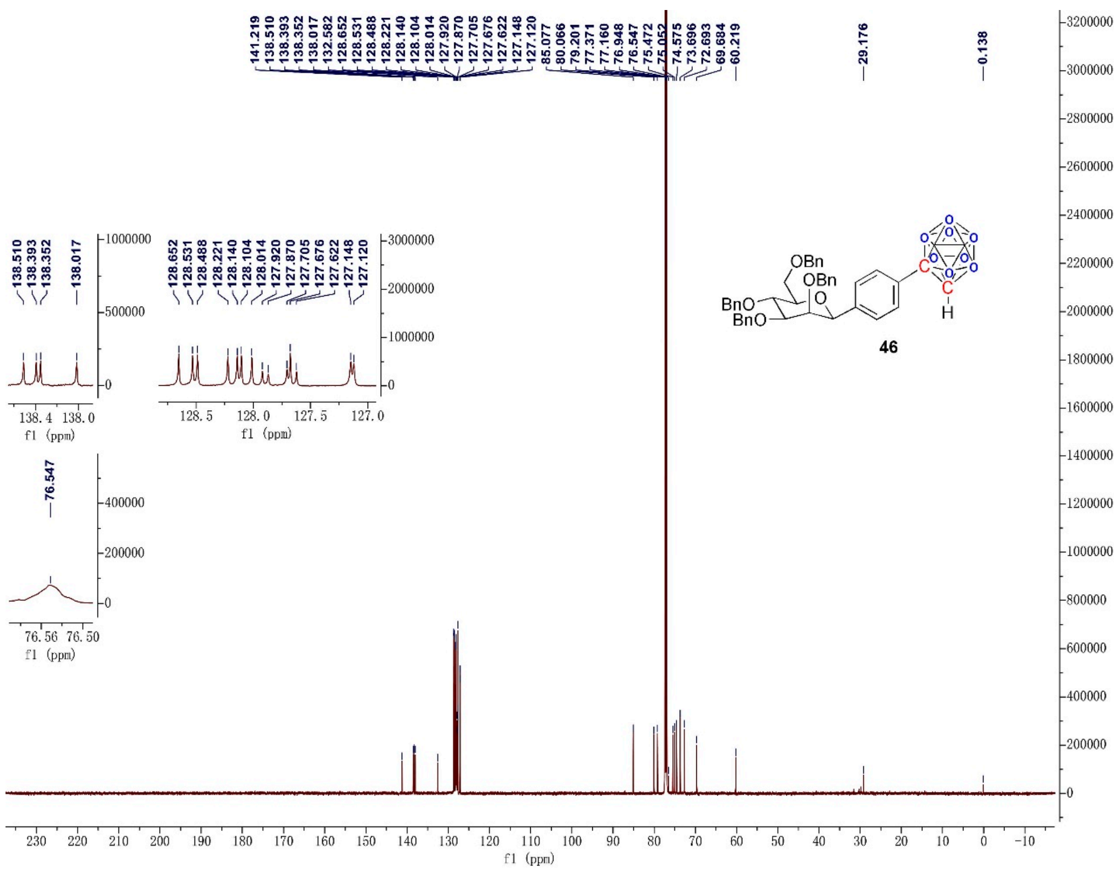

Figure S81 ^{13}C NMR spectrum of **46**

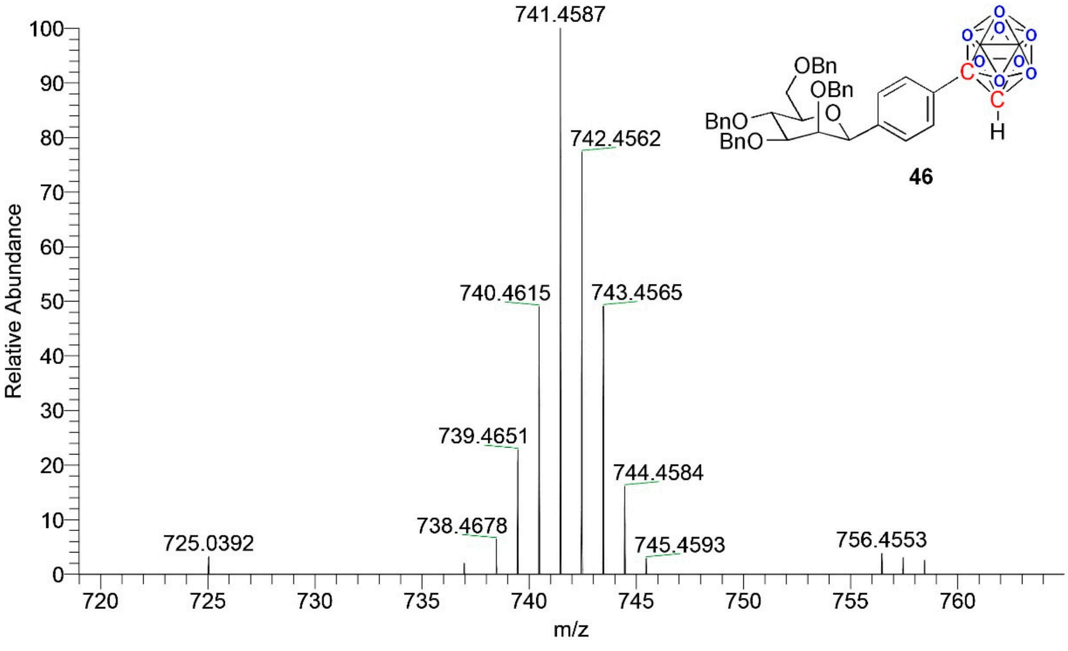

Figure S82 HR-MS (ESI/ion trap) spectrum of **46**

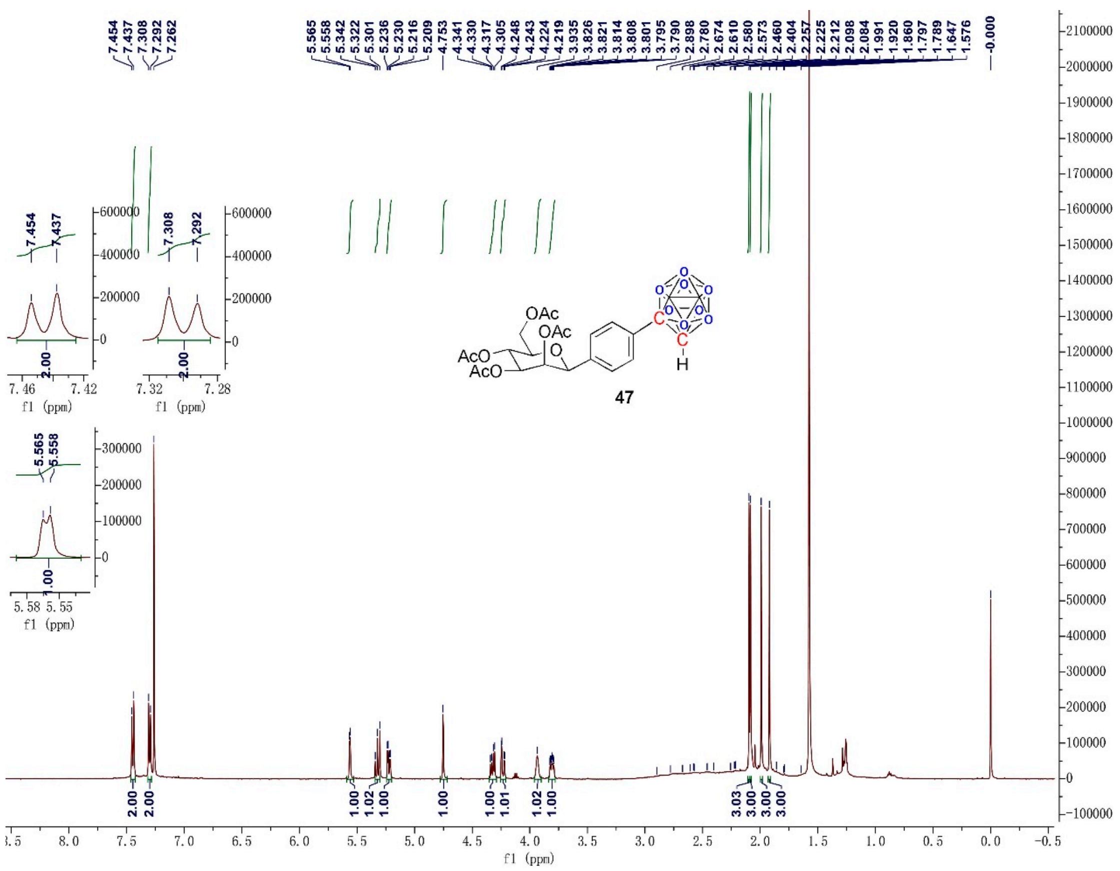

Figure S83 ^{1}H NMR spectrum of **47**

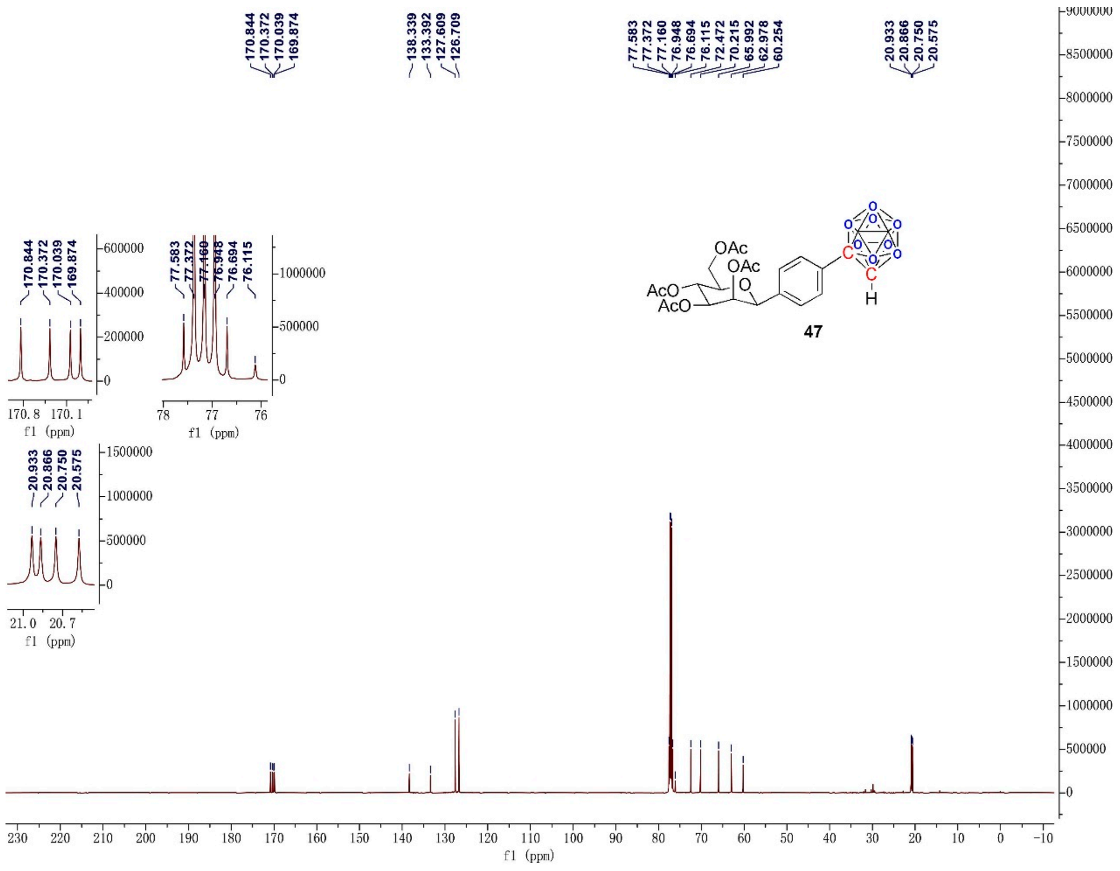

Figure S84 ^{13}C NMR spectrum of **47**

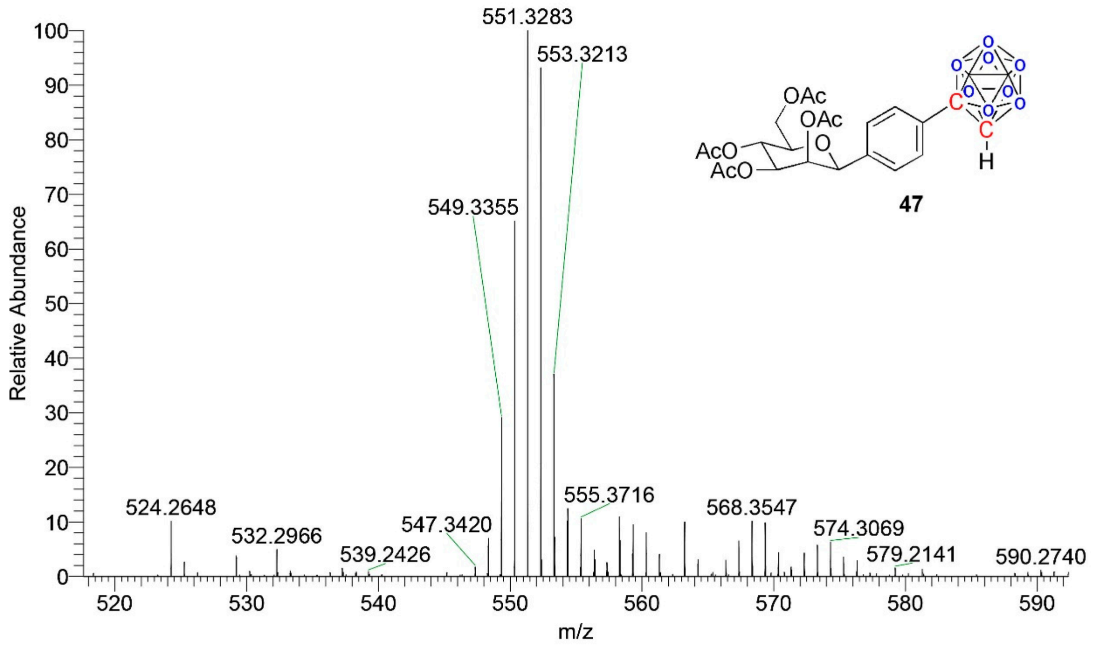

Figure S85 HR-MS (ESI/ion trap) spectrum of **47**

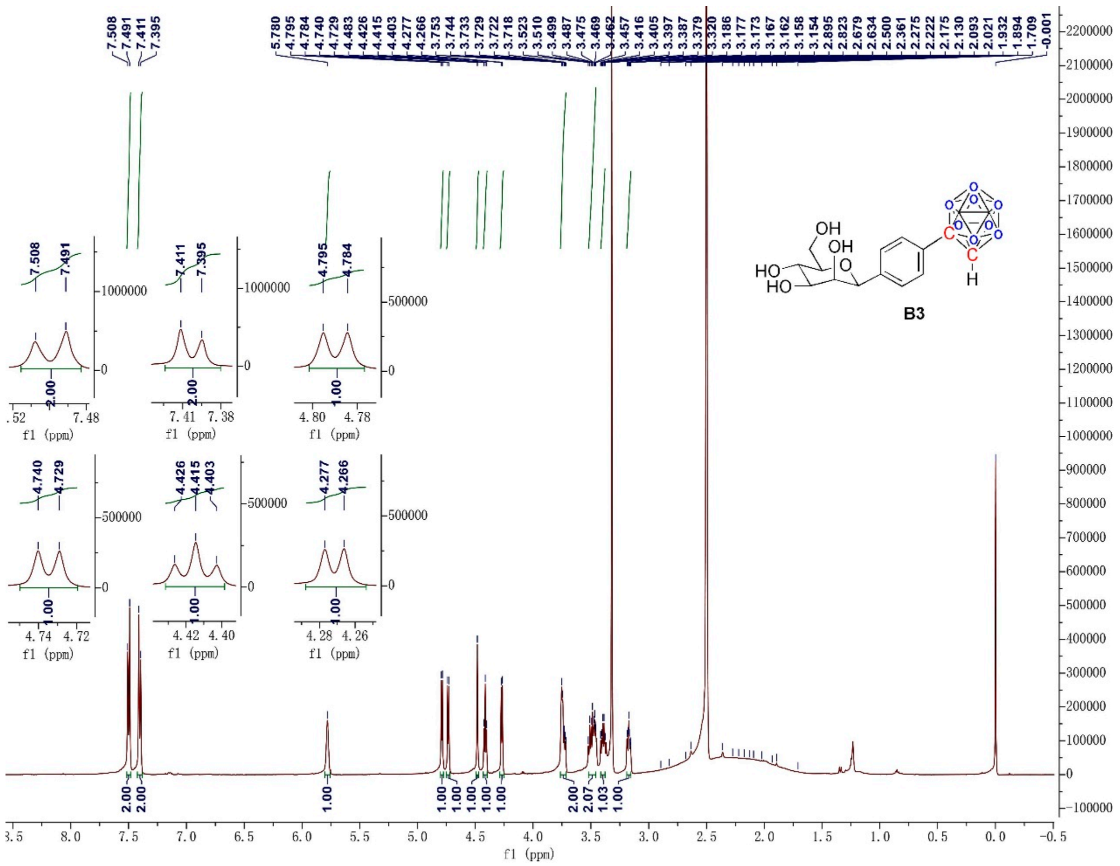

Figure S86 ^{1}H NMR spectrum of **B3**

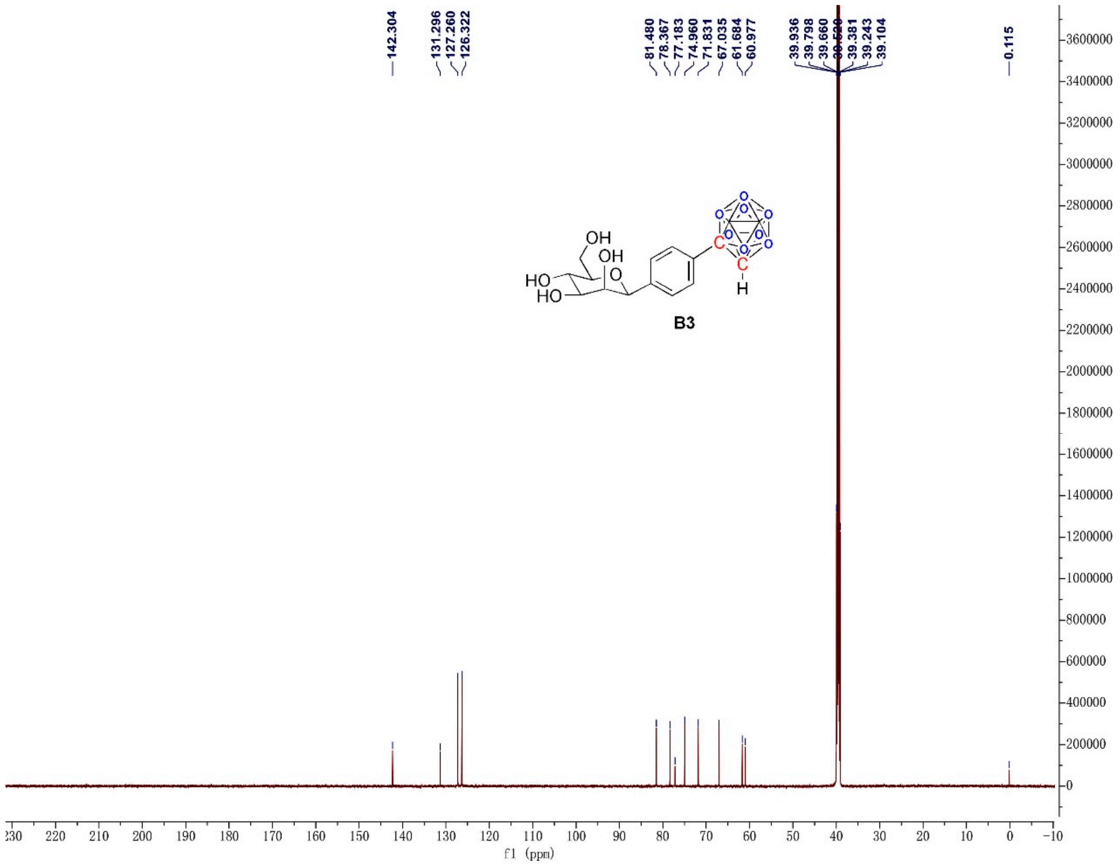

Figure S87 ^{13}C NMR spectrum of **B3**

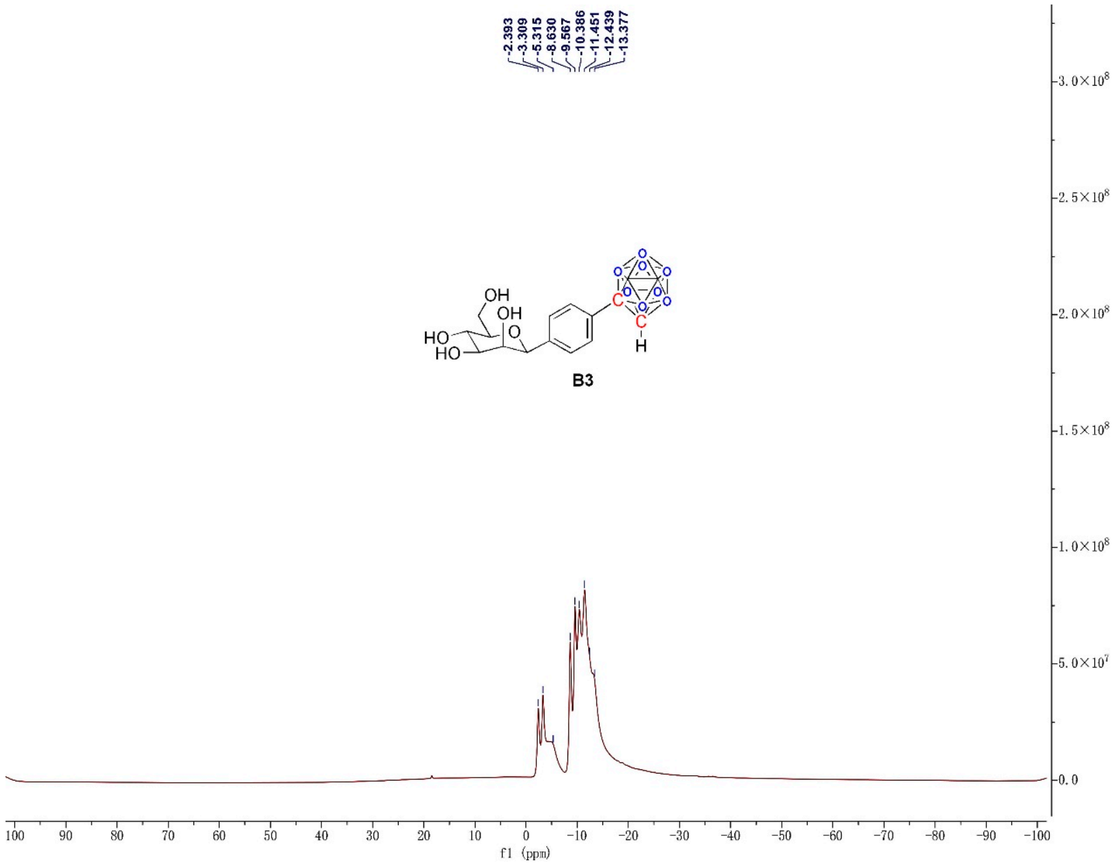

Figure S88 ^{11}B NMR spectrum of **B3**

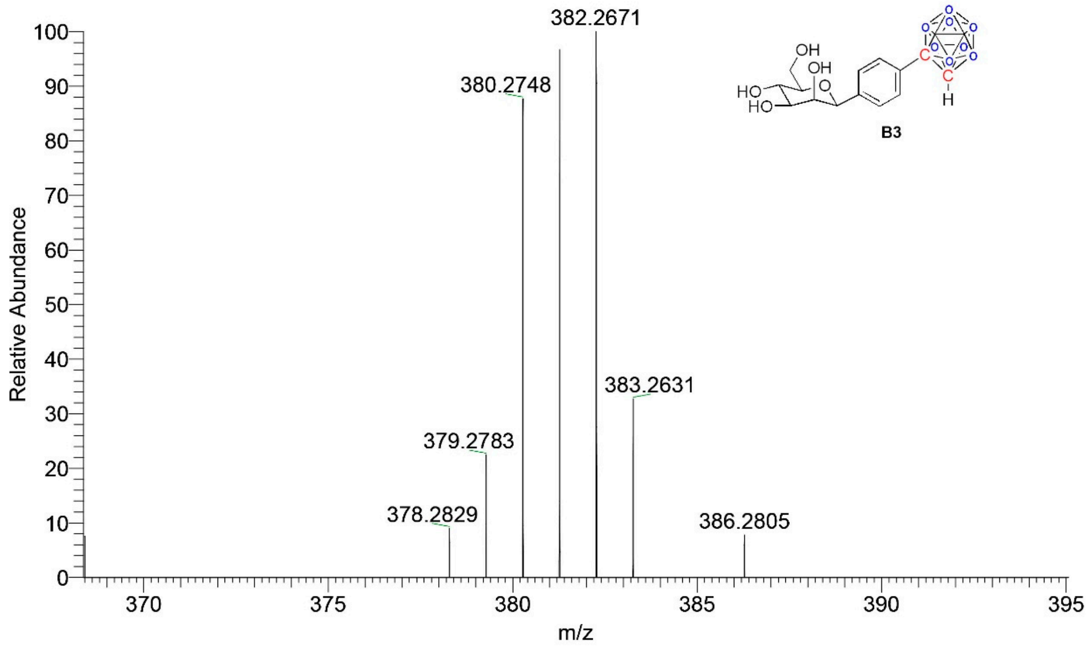

Figure S89 HR-MS (ESI/ion trap) spectrum of **B3**

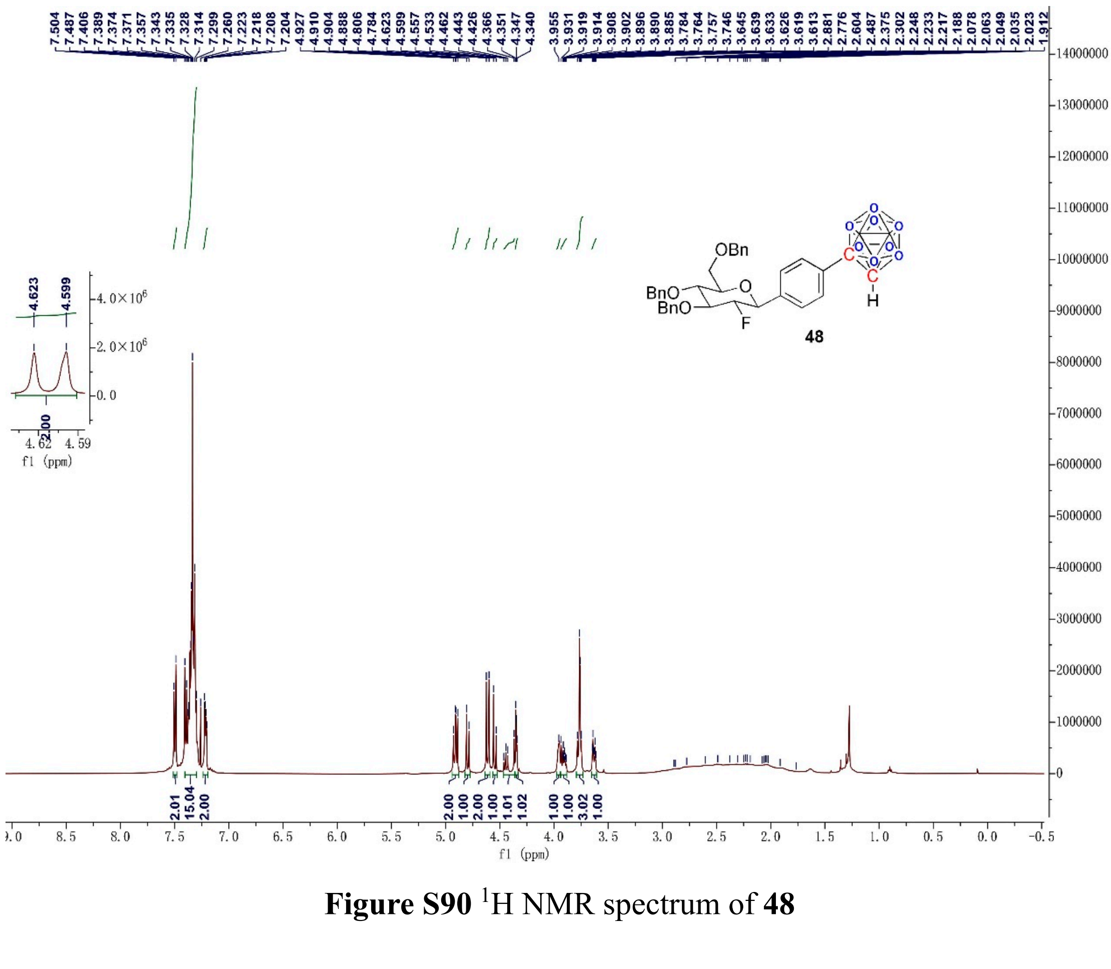

Figure S90 ^{1}H NMR spectrum of **48**

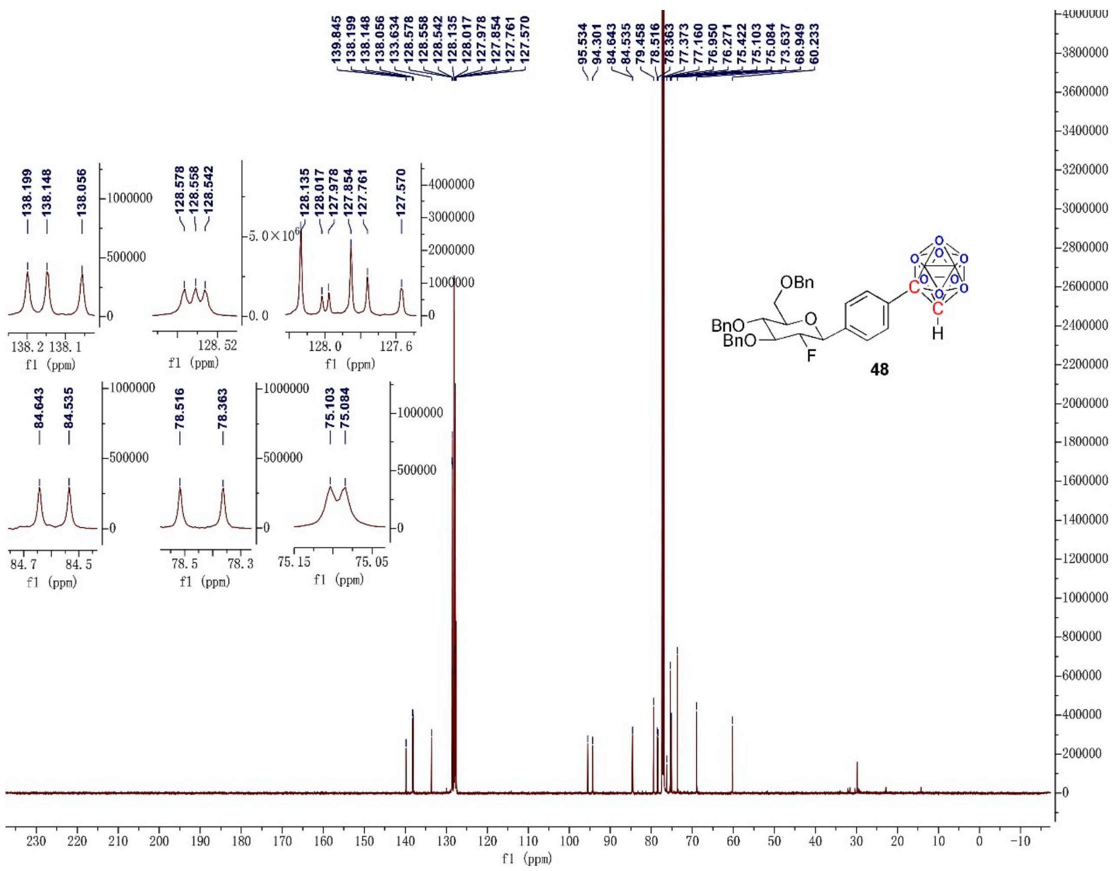

Figure S91 ^{13}C NMR spectrum of **48**

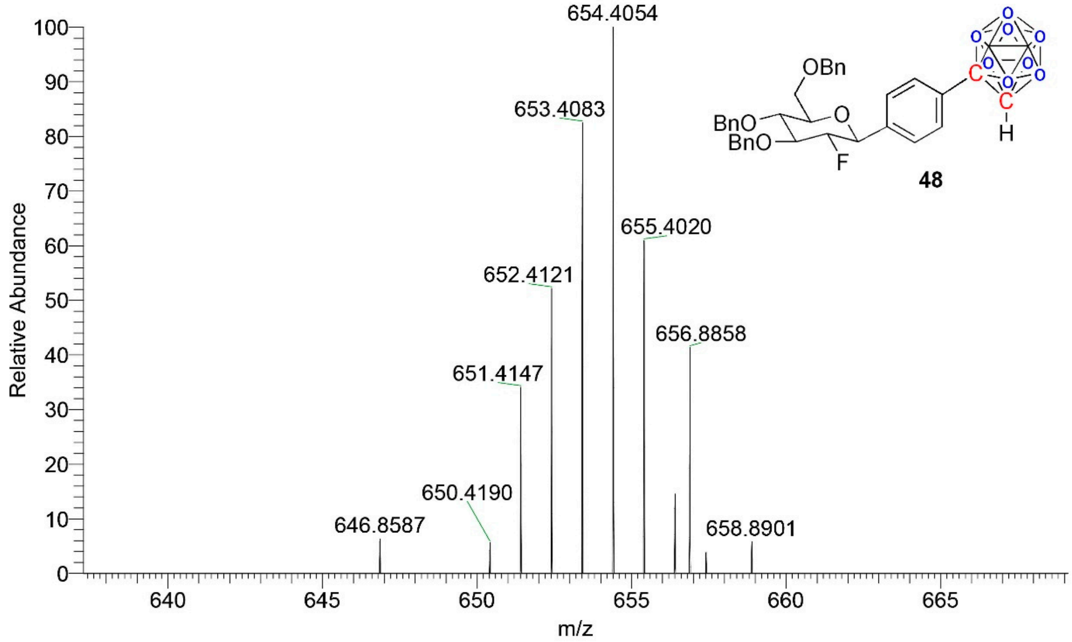

Figure S92 HR-MS (ESI/ion trap) spectrum of **48**

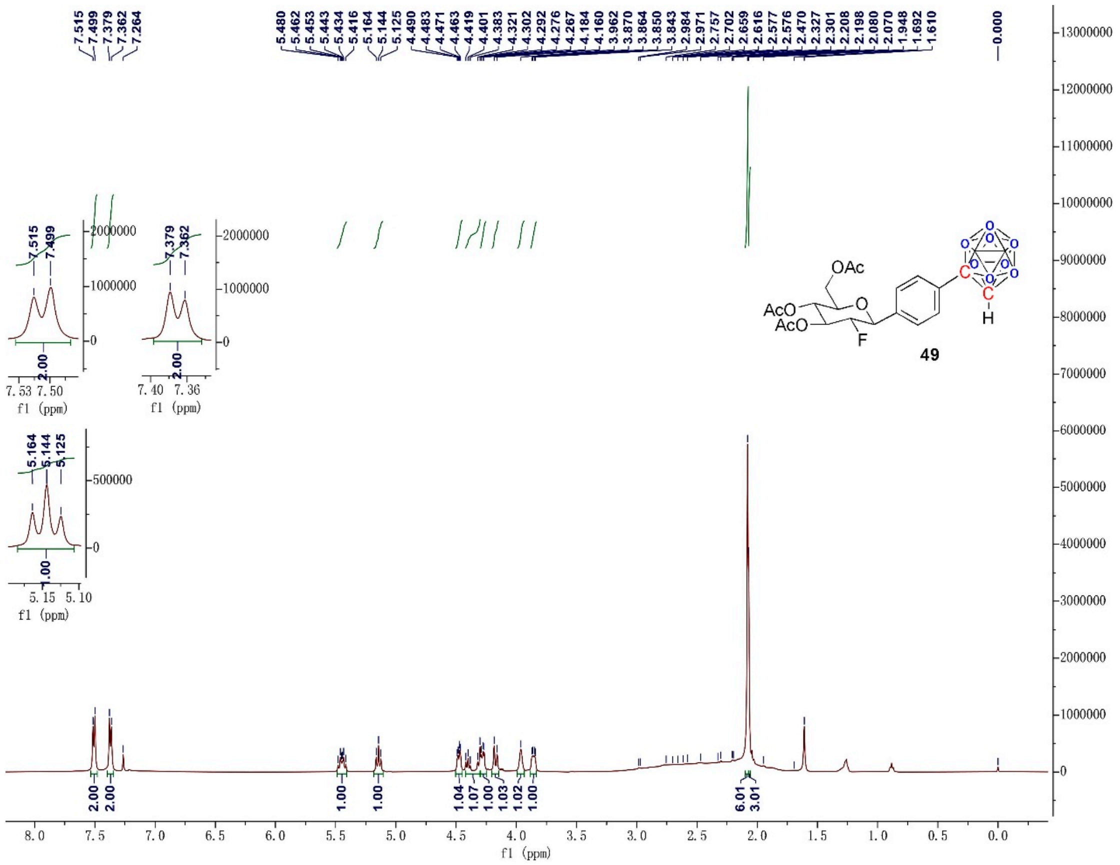

Figure S93 ^{1}H NMR spectrum of **49**

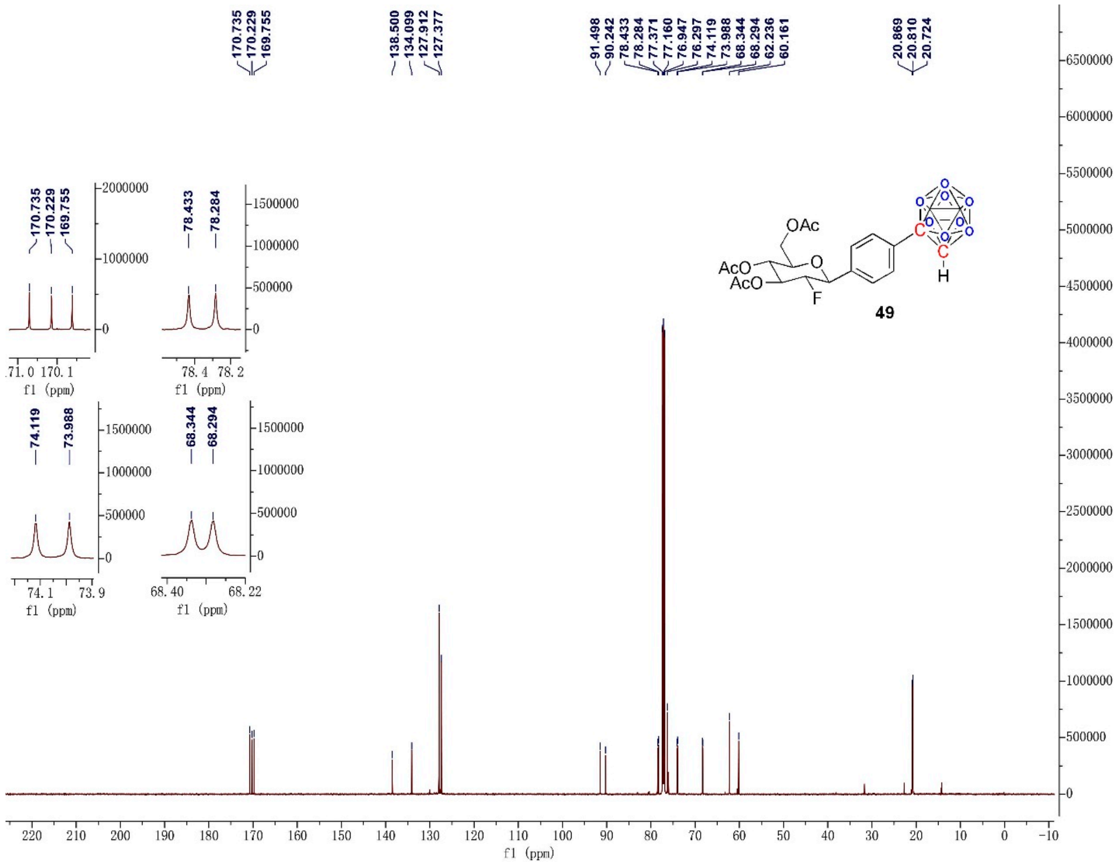

Figure S94 ^{13}C NMR spectrum of **49**

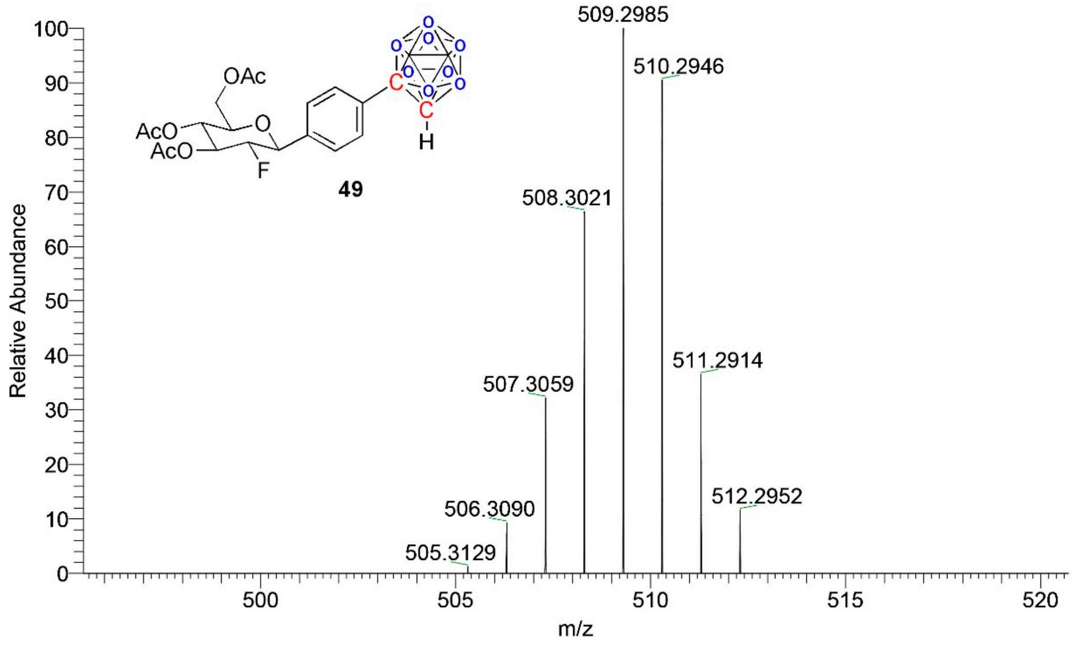

Figure S95 HR-MS (ESI/ion trap) spectrum of **49**

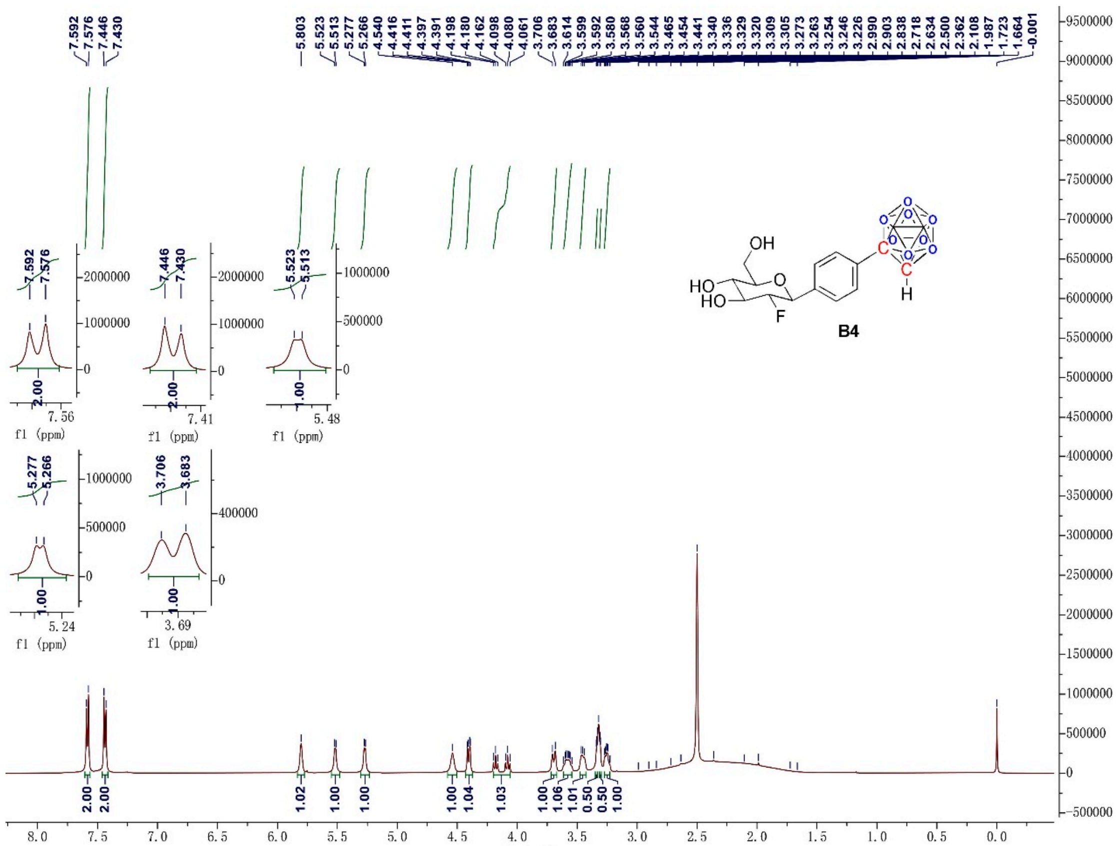

Figure S96 ^{1}H NMR spectrum of **B4**

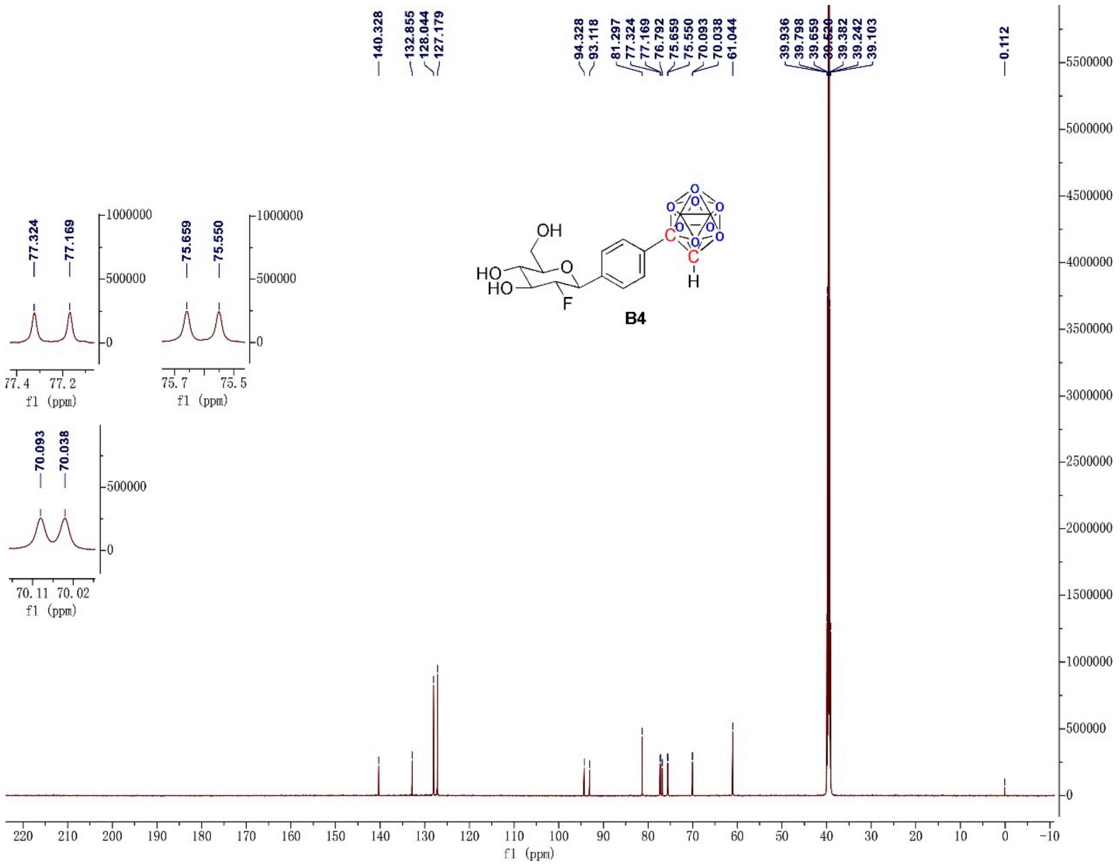

Figure S97 ^{13}C NMR spectrum of **B4**

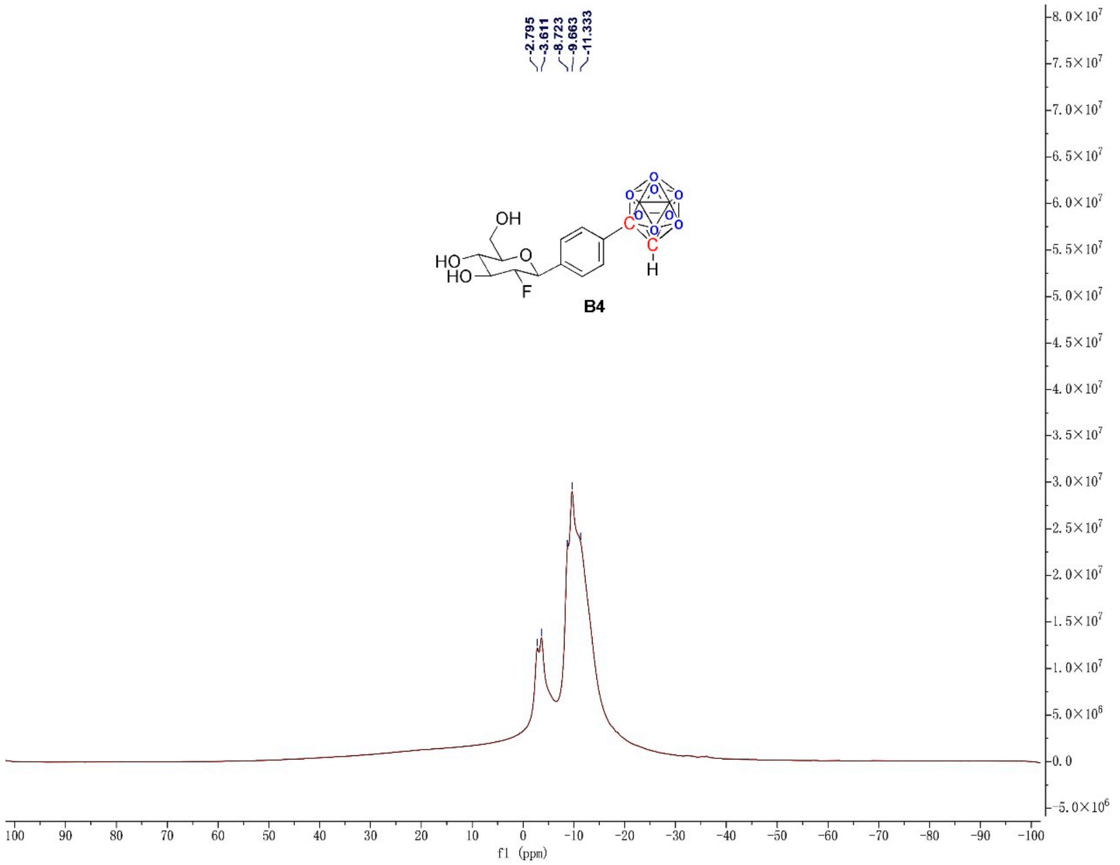

Figure S98 ^{11}B NMR spectrum of **B4**

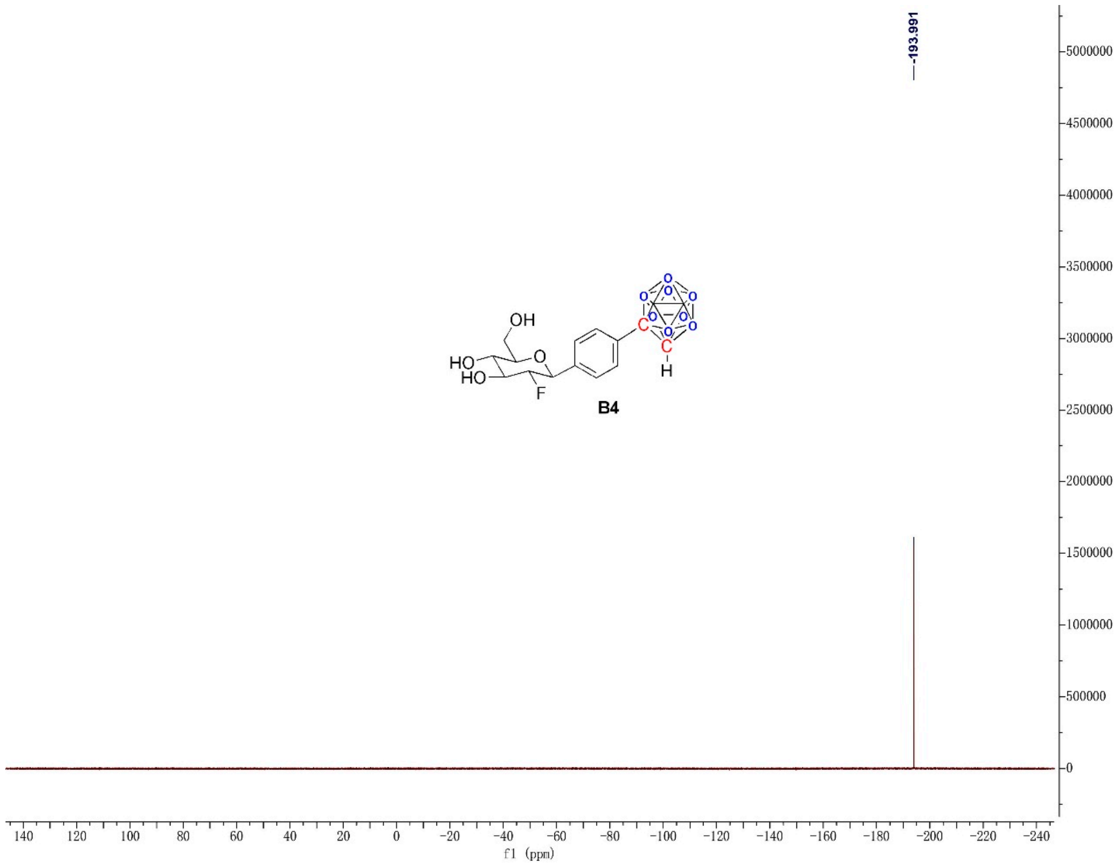

Figure S99 ^{19}F NMR spectrum of **B4**

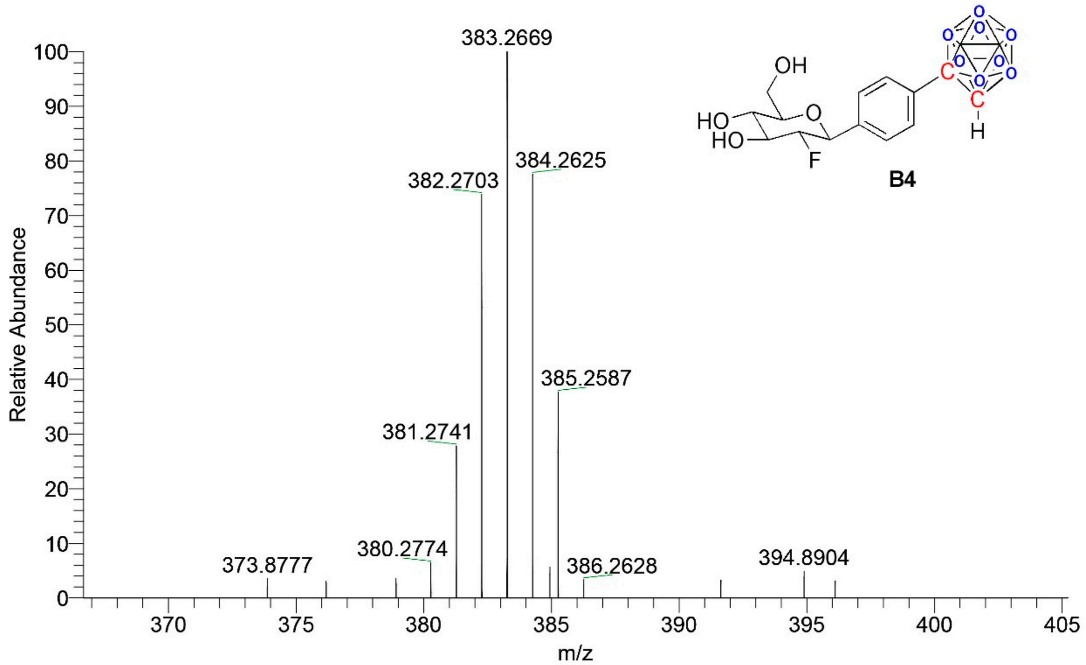

Figure S100 HR-MS (ESI/ion trap) spectrum of **B4**

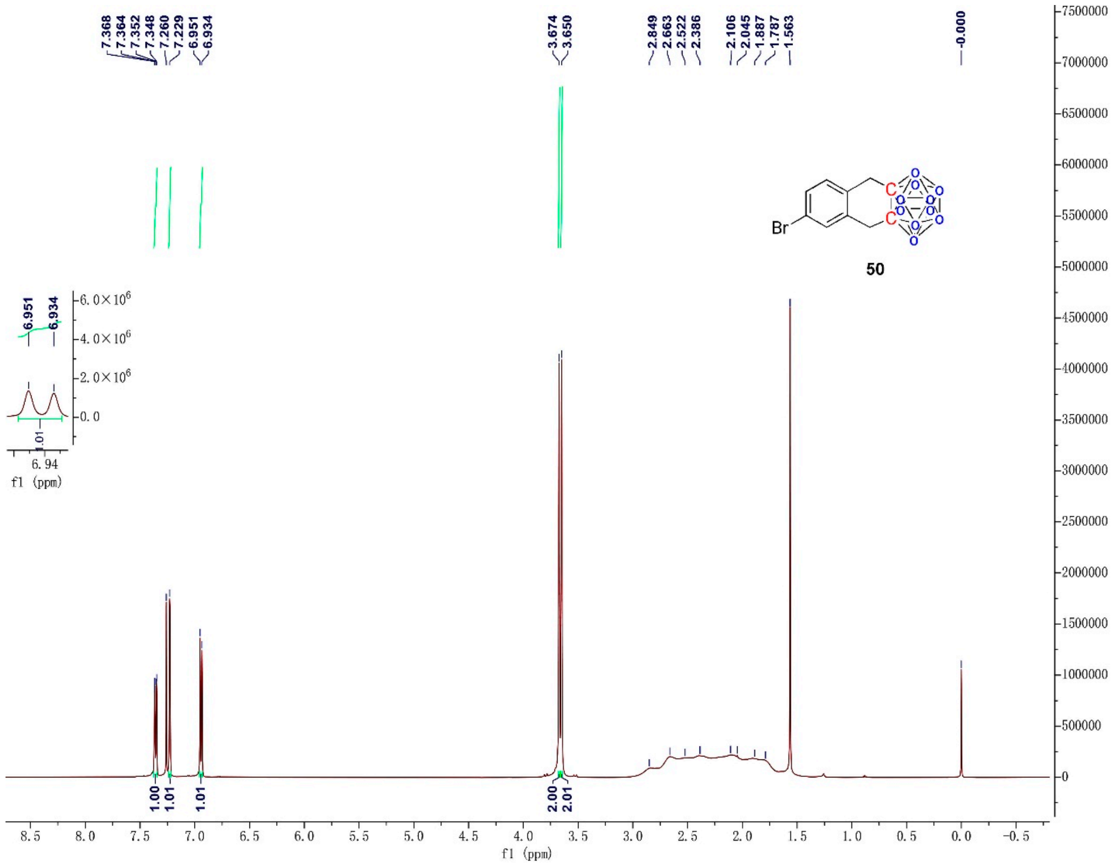

Figure S101 ^{1}H NMR spectrum of **50**

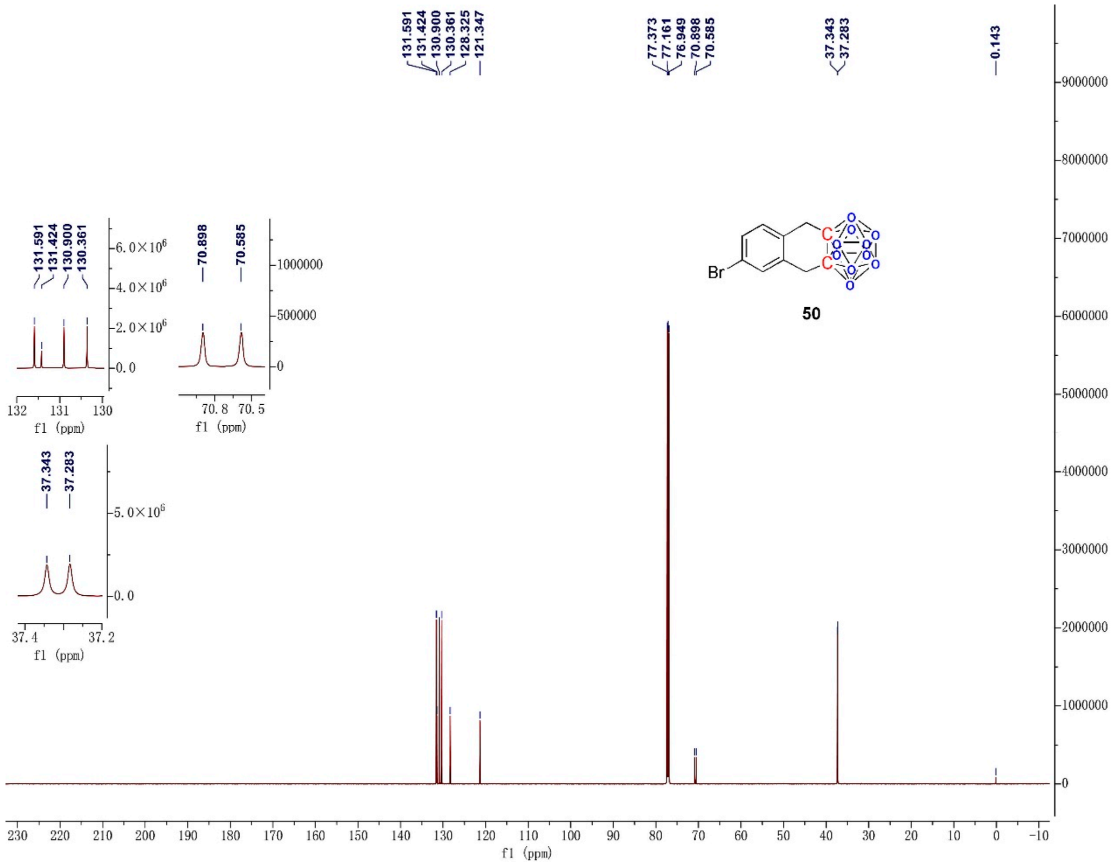

Figure S102 ^{13}C NMR spectrum of **50**

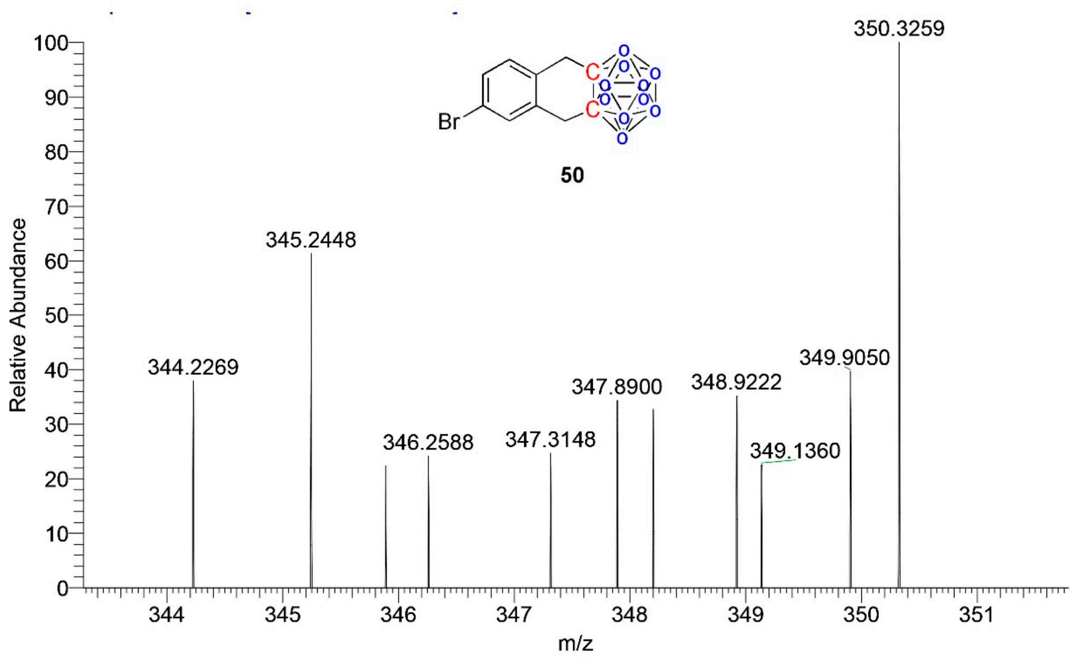

Figure S103 HR-MS (ESI/ion trap) spectrum of **50**

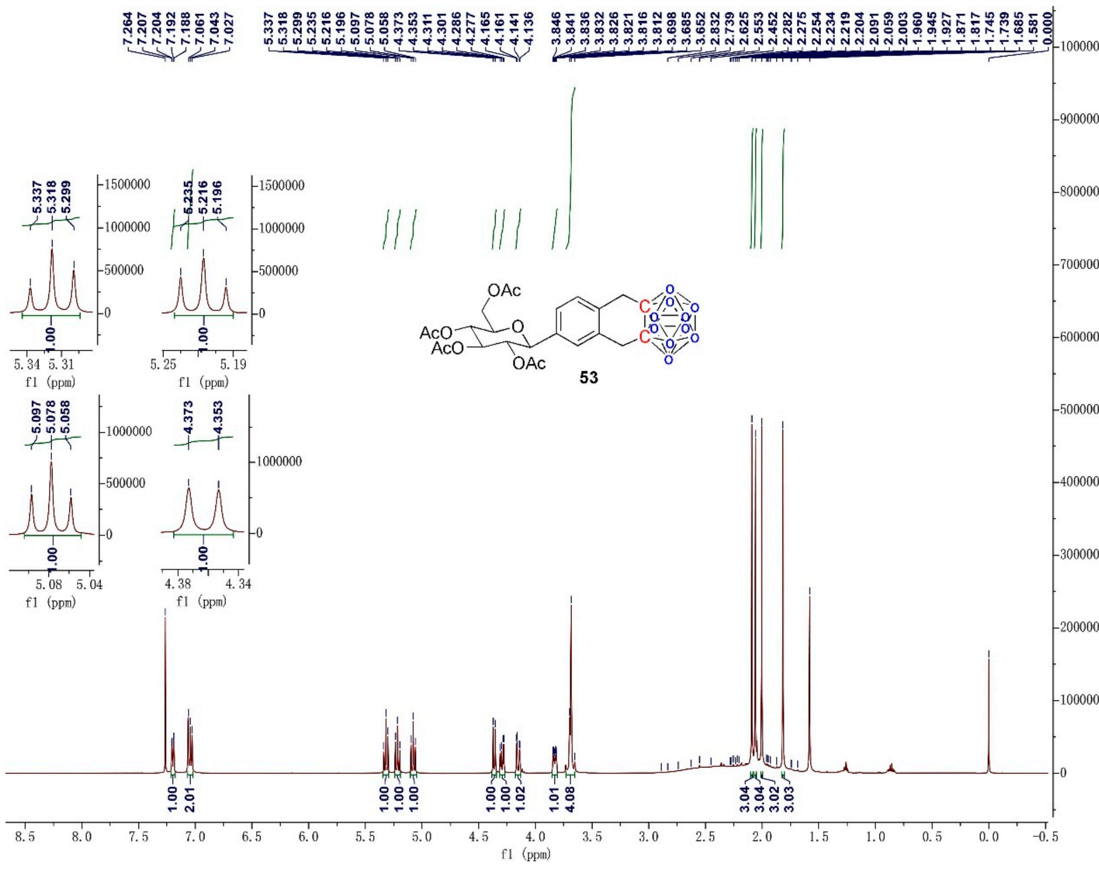

Figure S104 ^{1}H NMR spectrum of **53**

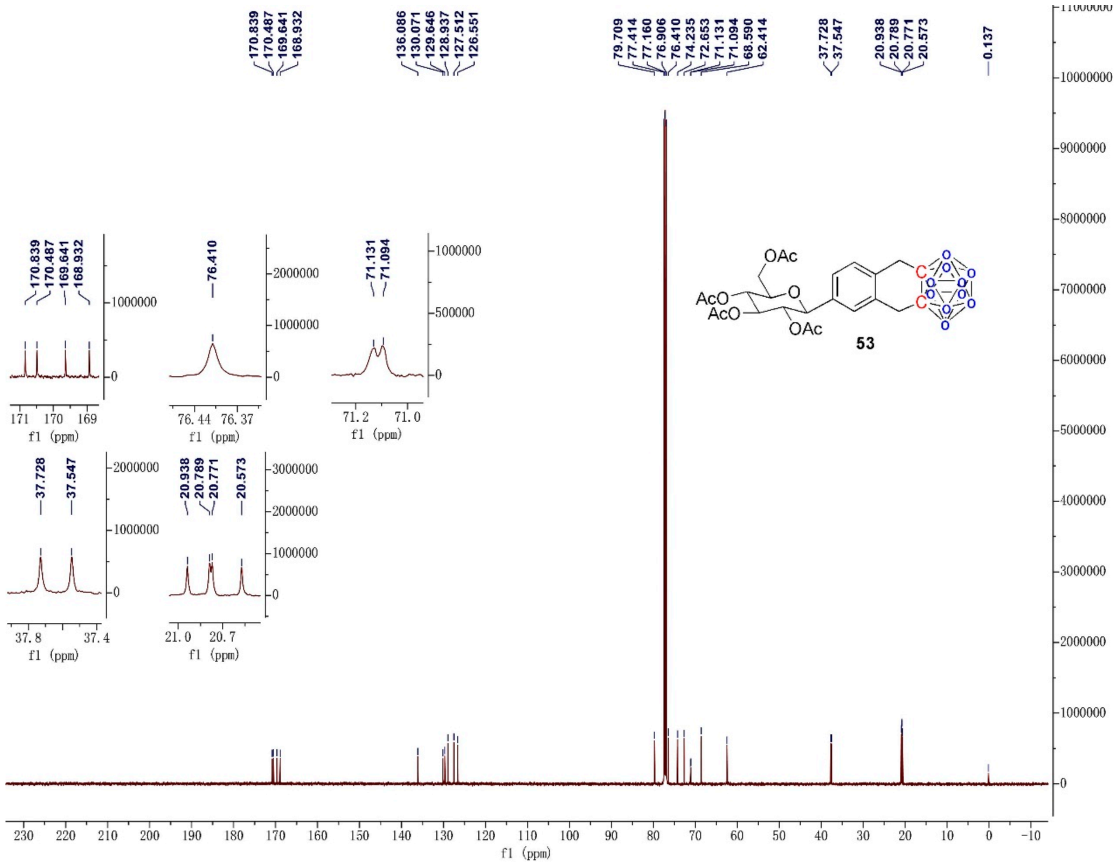

Figure S105 ^{13}C NMR spectrum of **53**

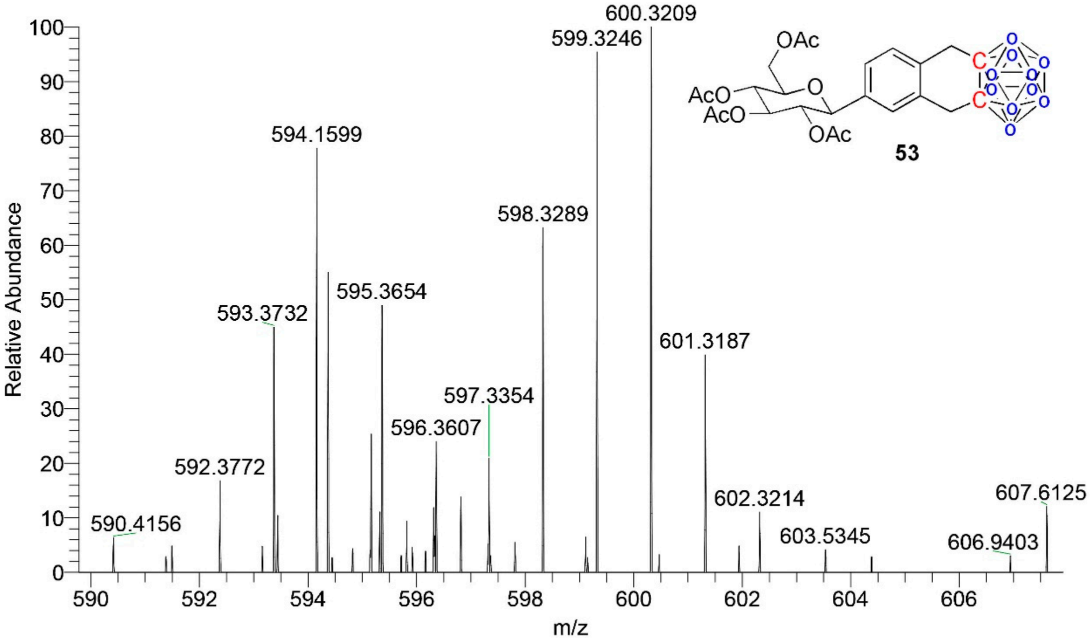

Figure S106 HR-MS (ESI/ion trap) spectrum of **53**

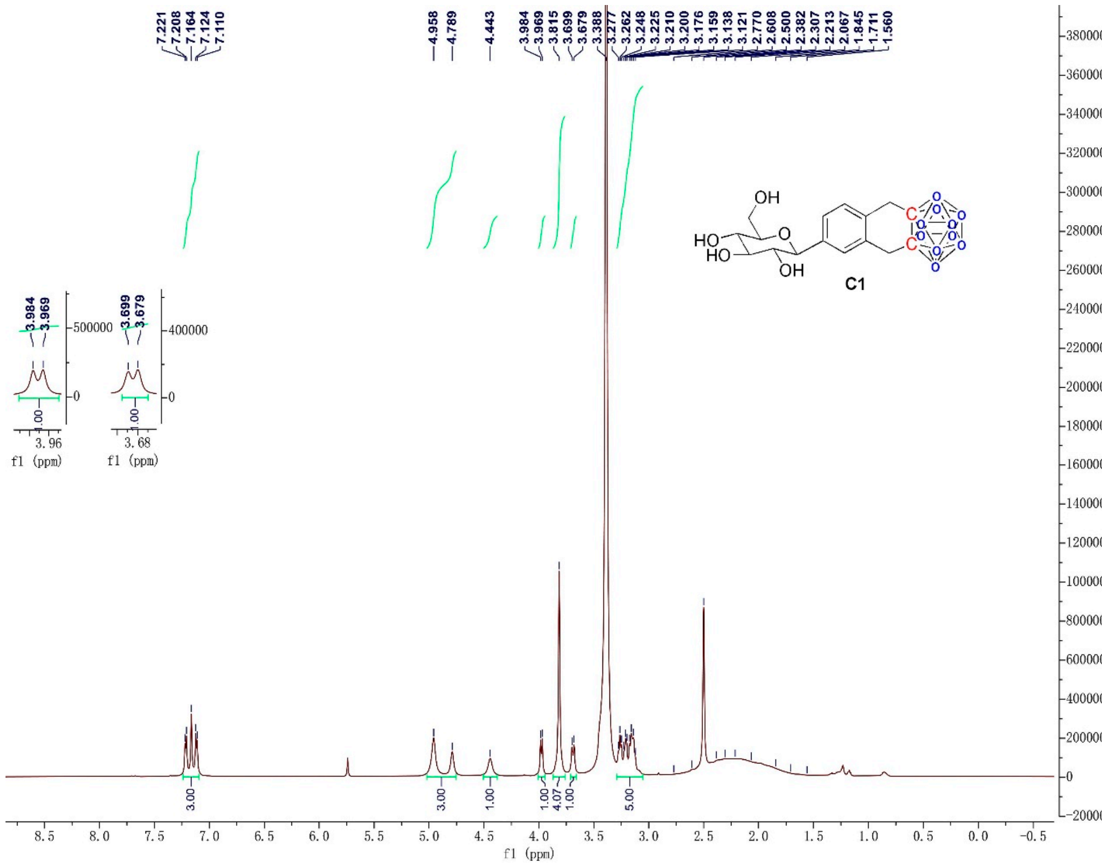

Figure S107 ^{1}H NMR spectrum of **C1**

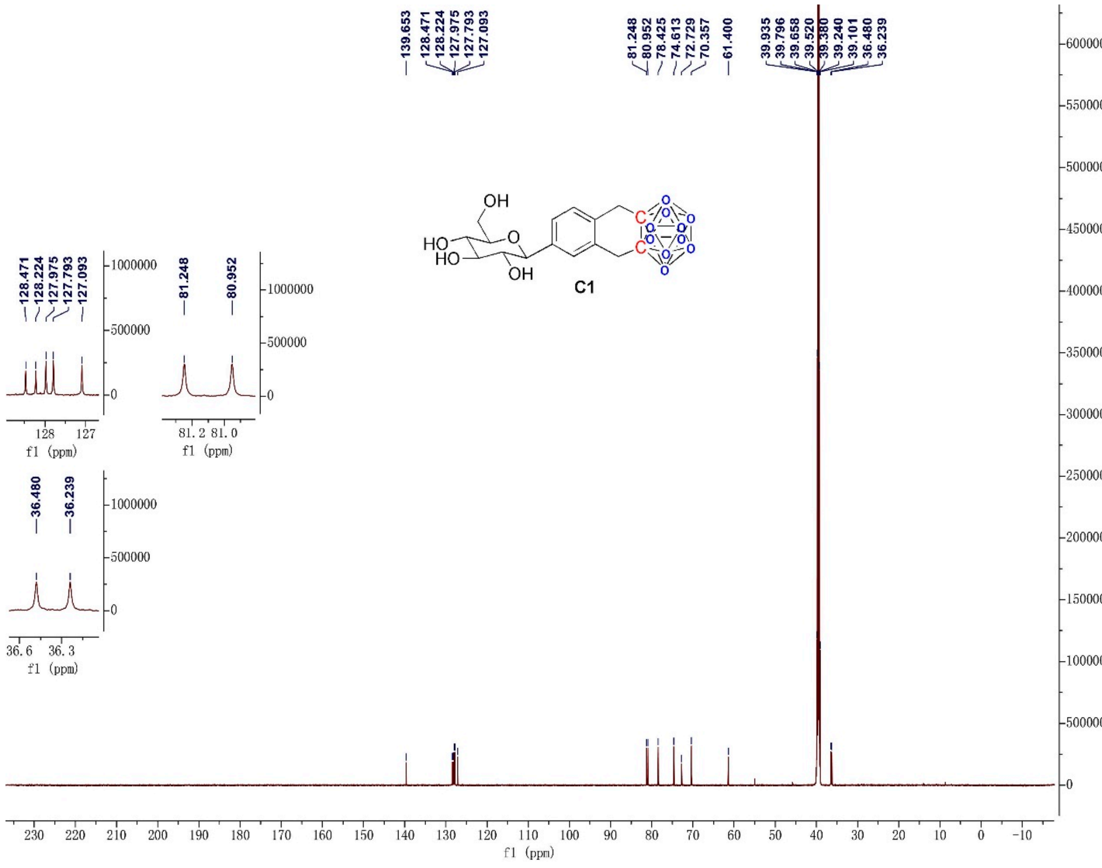

Figure S108 ^{13}C NMR spectrum of **C1**

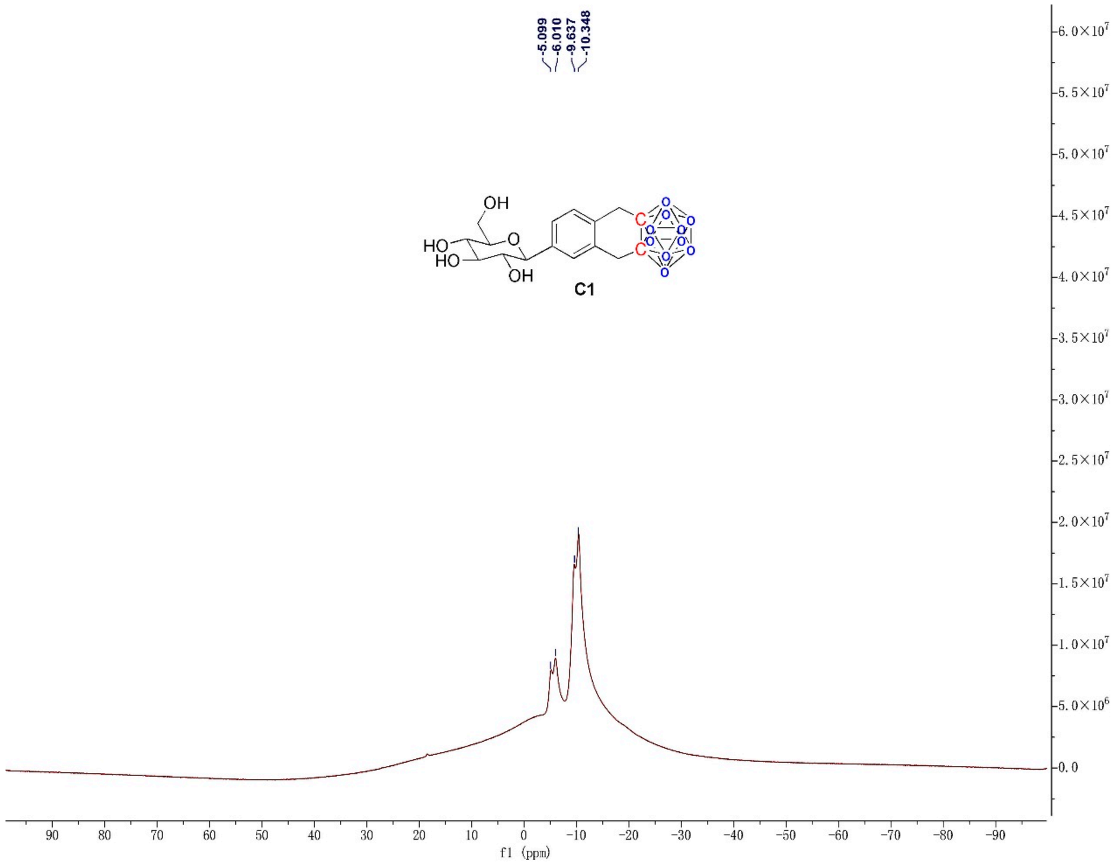

Figure S109 ^{11}B NMR spectrum of **C1**

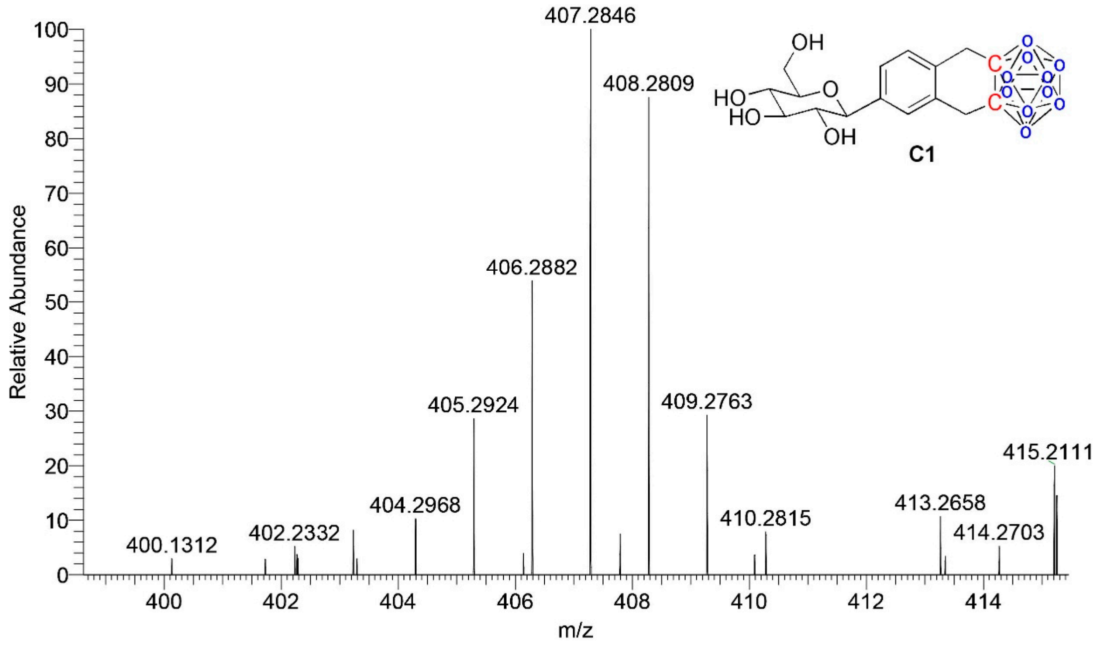

Figure S110 HR-MS (ESI/ion trap) spectrum of **C1**

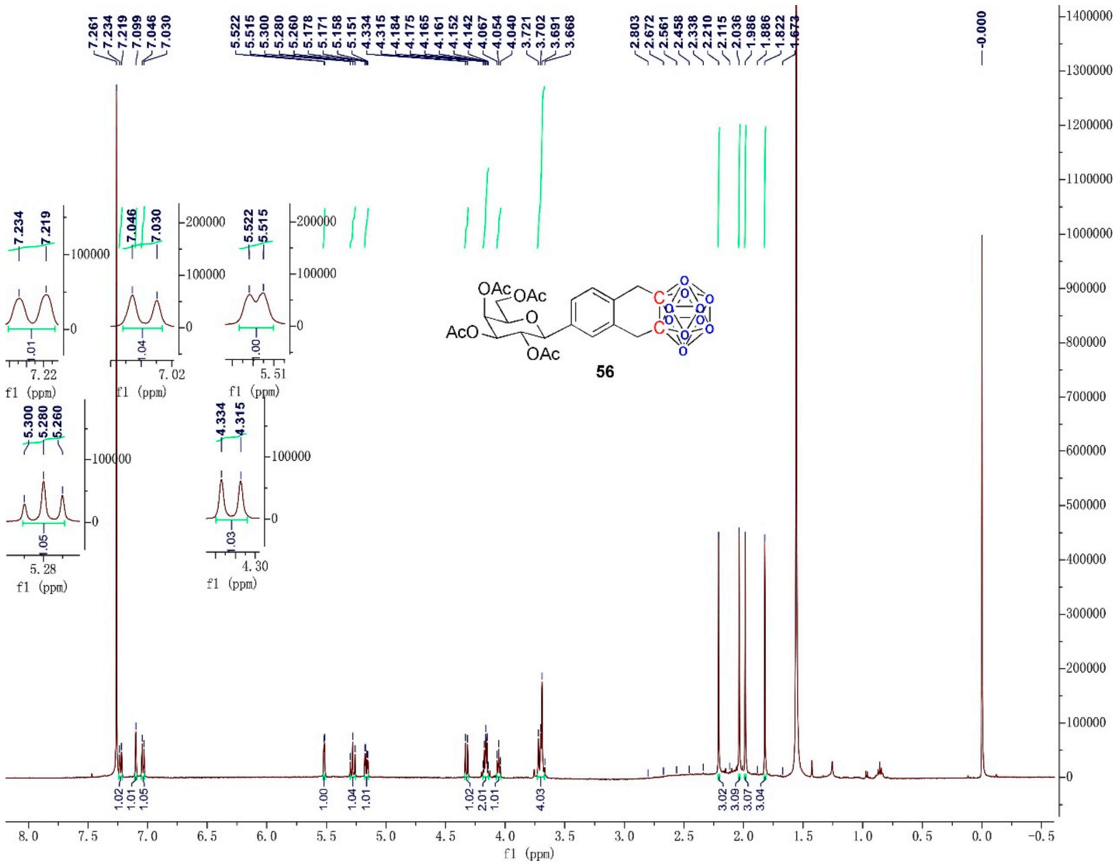

Figure S111 ^{1}H NMR spectrum of **56**

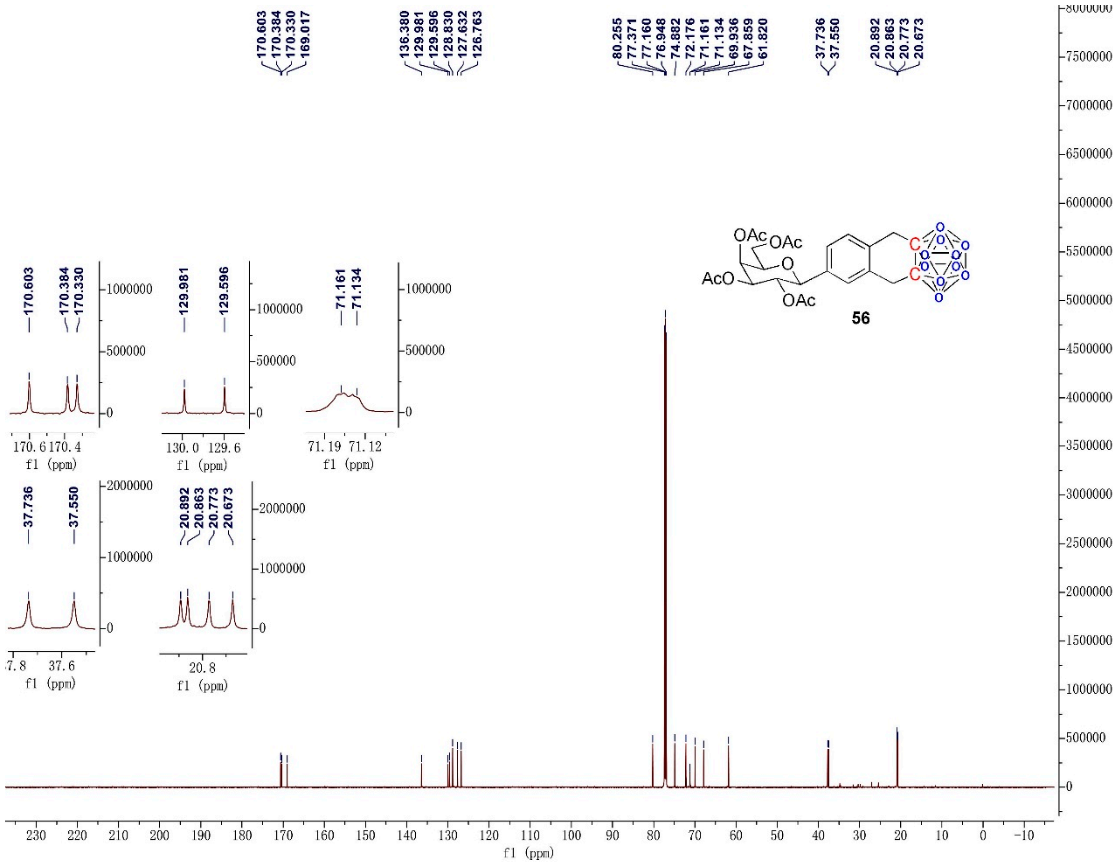

Figure S112 ^{13}C NMR spectrum of **56**

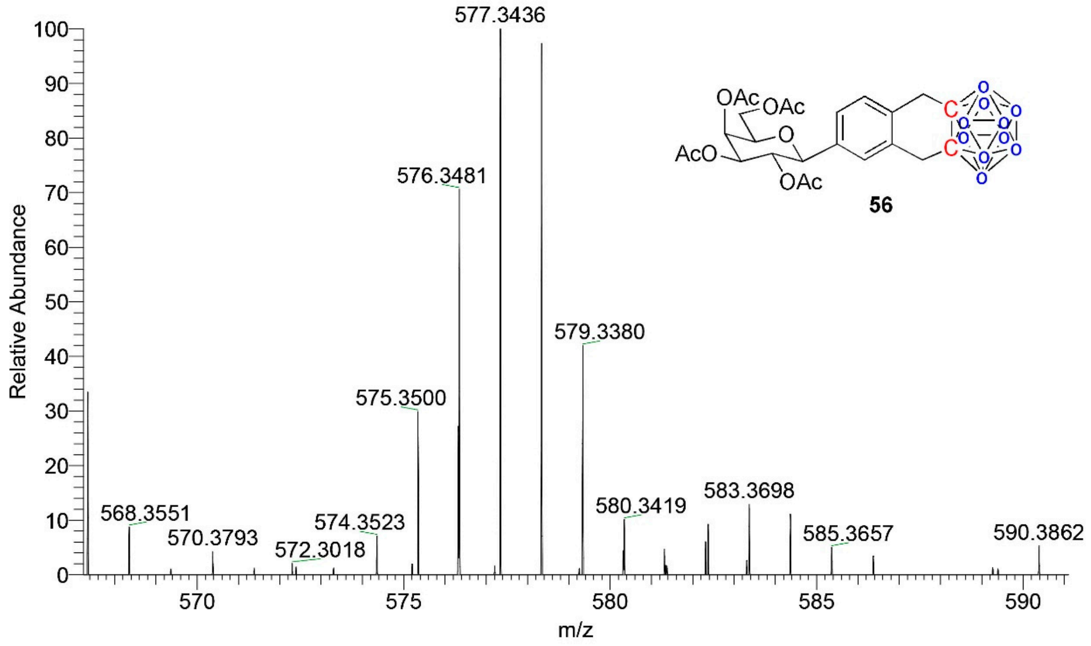

Figure S113 HR-MS (ESI/ion trap) spectrum of **56**

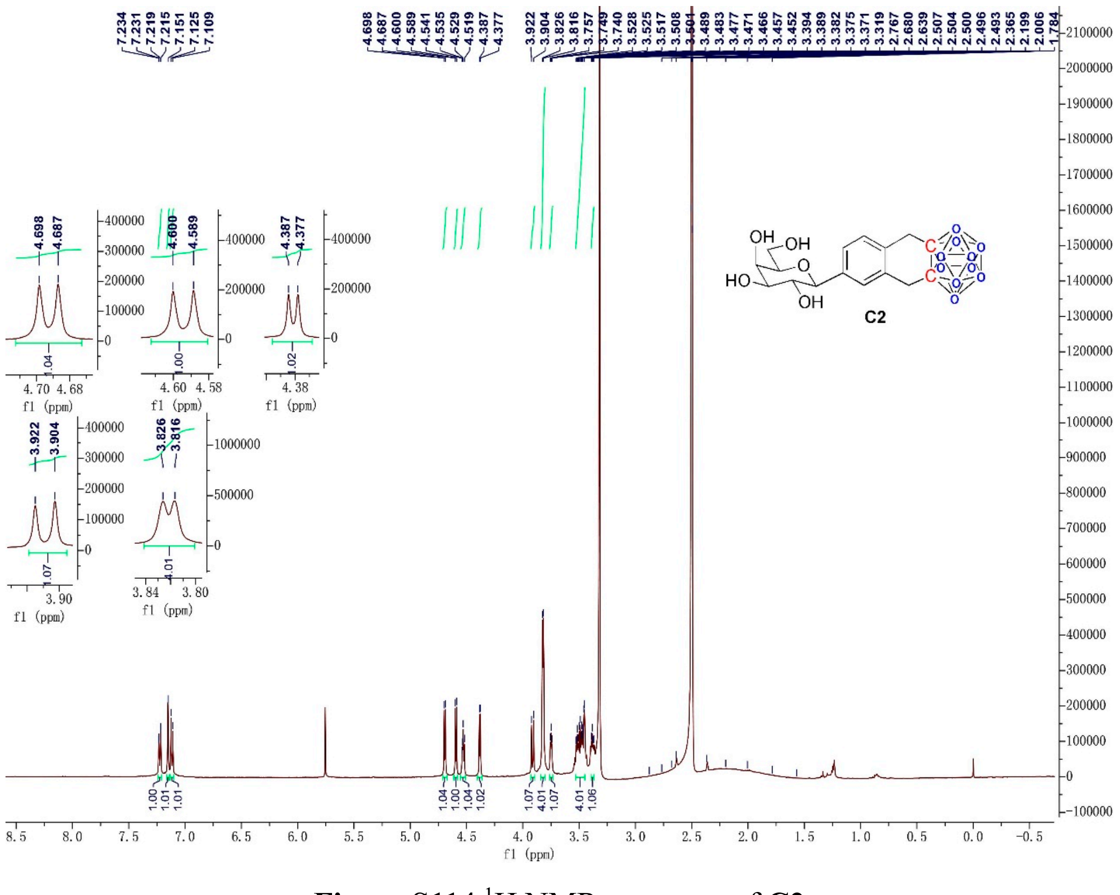

Figure S114 ^{1}H NMR spectrum of **C2**

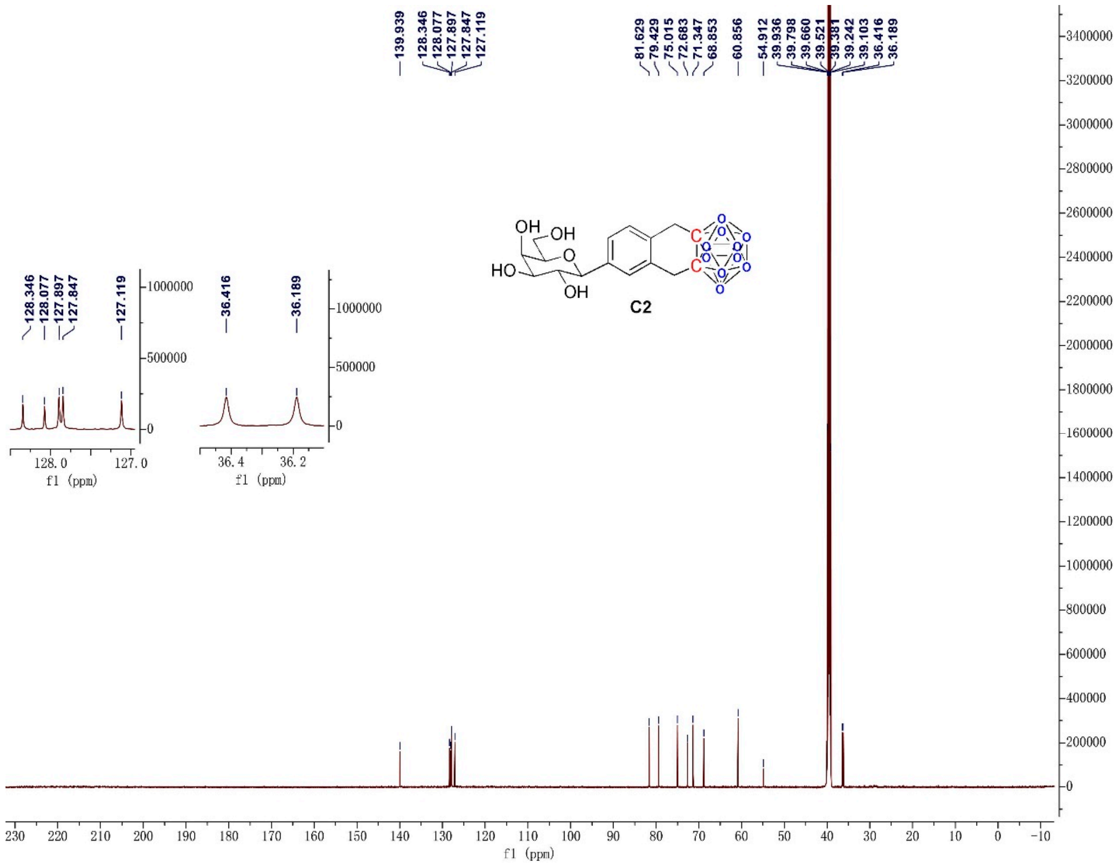

Figure S115 ^{13}C NMR spectrum of **C2**

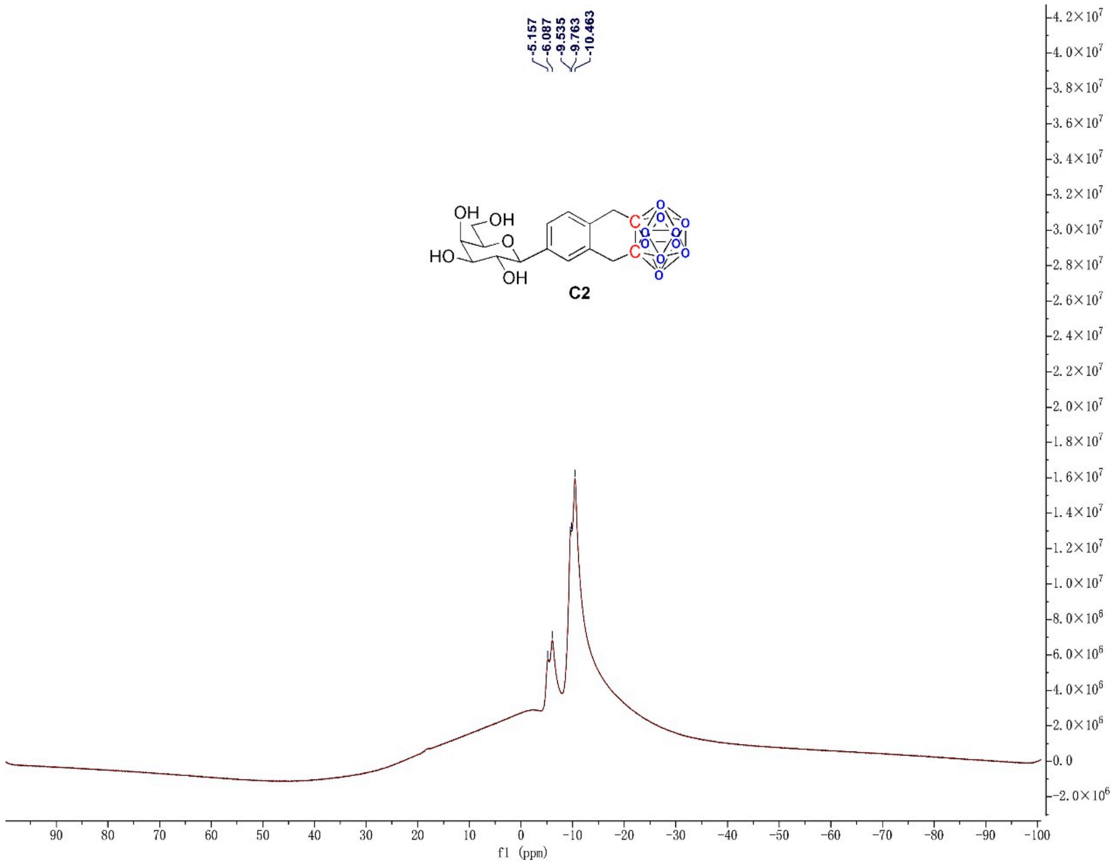

Figure S116 ^{11}B NMR spectrum of **C2**

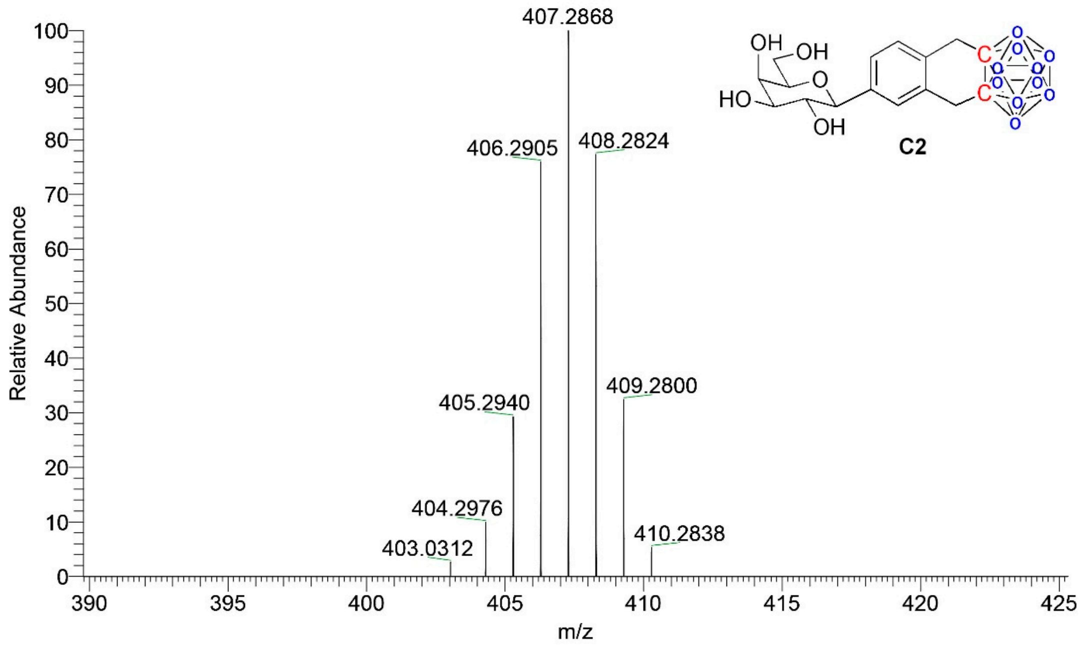

Figure S117 HR-MS (ESI/ion trap) spectrum of **C2**

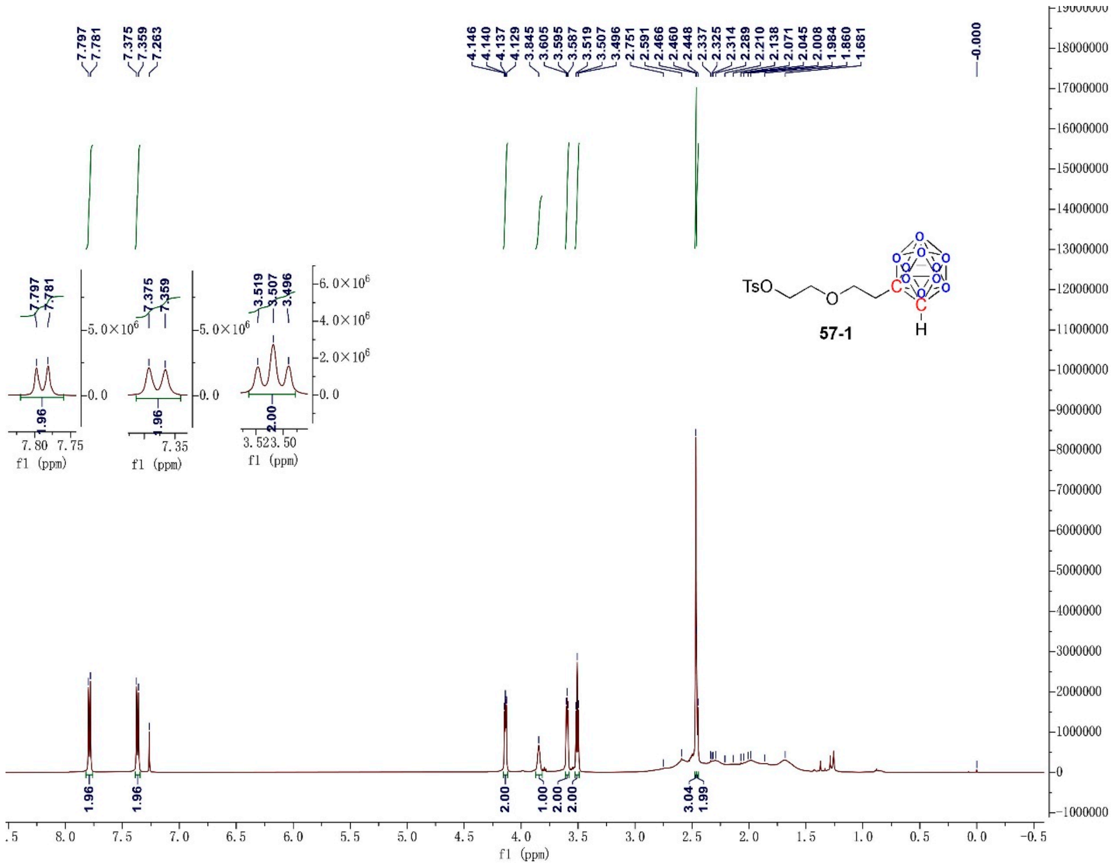

Figure S118 ^{1}H NMR spectrum of **57-1**

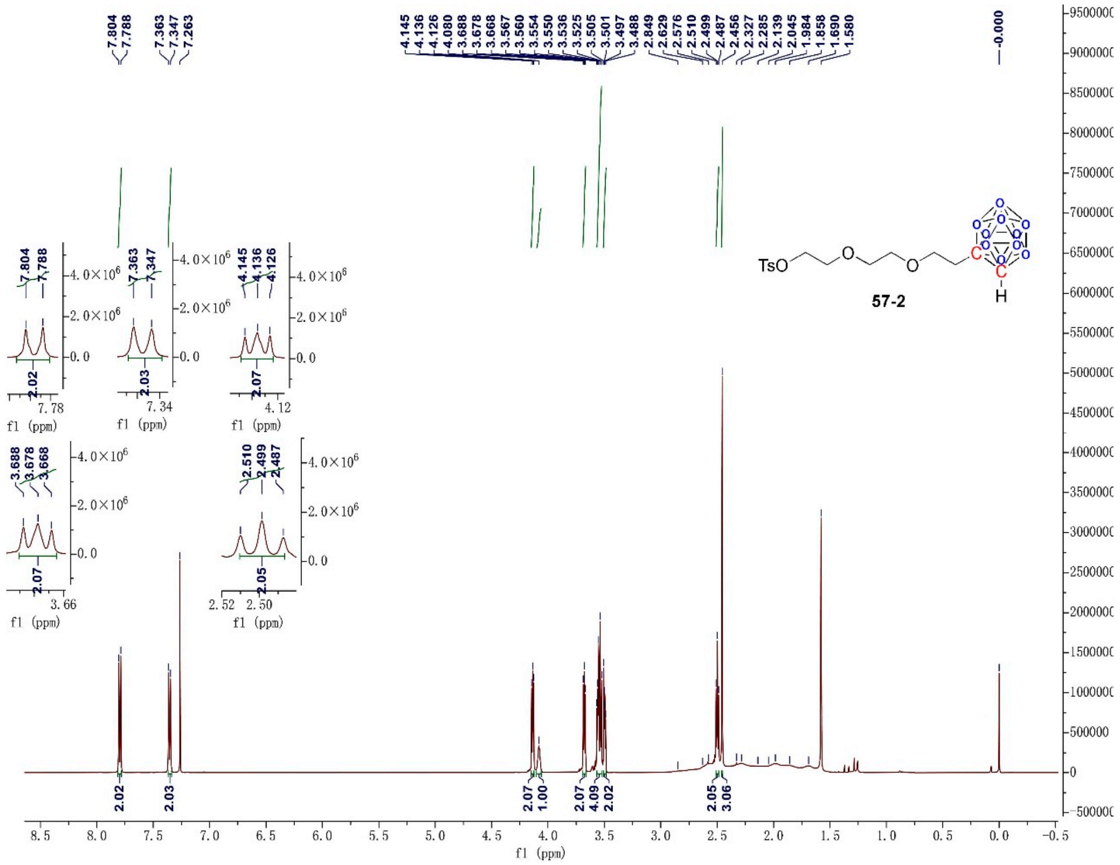

Figure S119 ^{1}H NMR spectrum of **57-2**

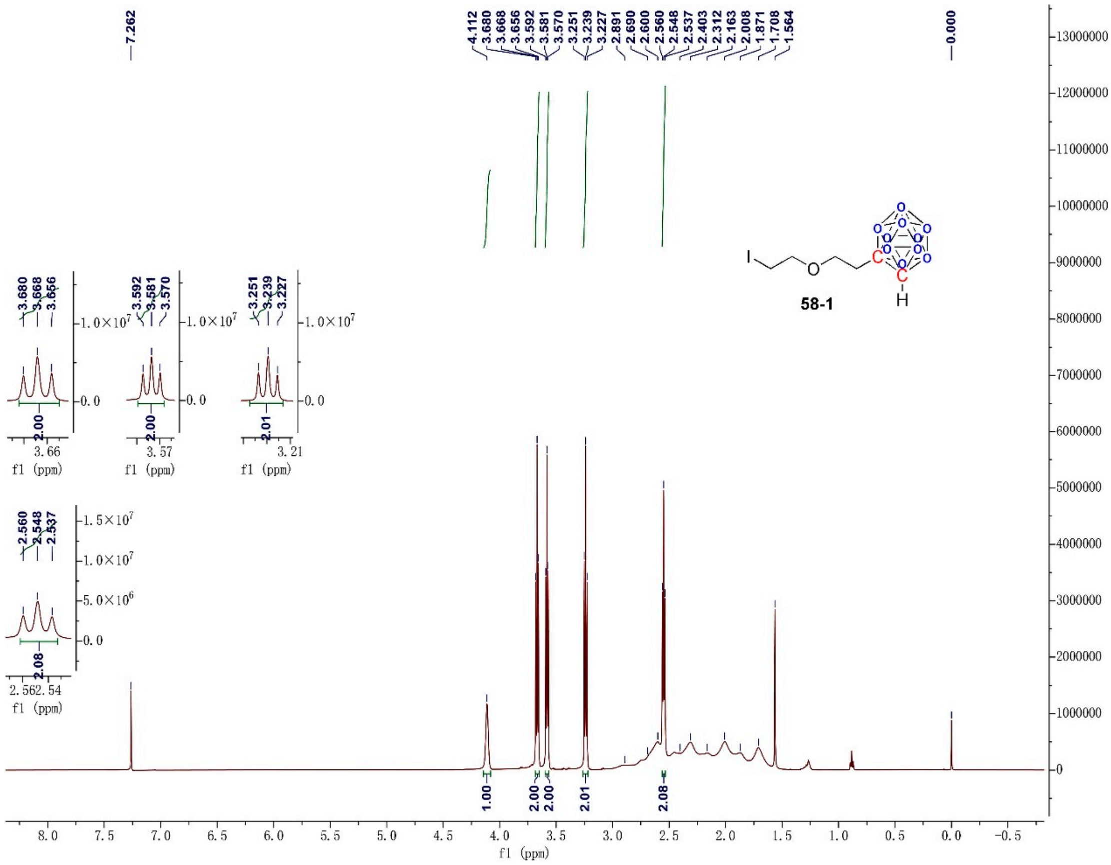

Figure S120 ^{1}H NMR spectrum of **58-1**

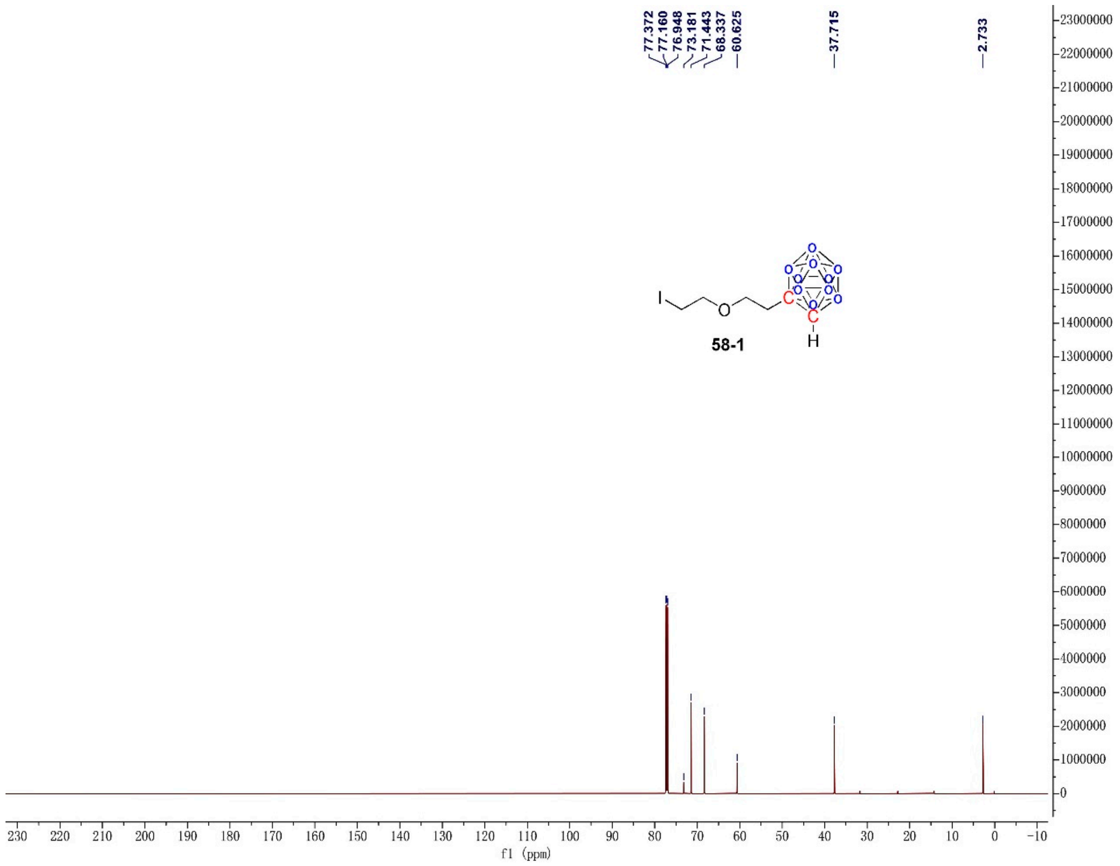

Figure S121 ^{13}C NMR spectrum of **58-1**

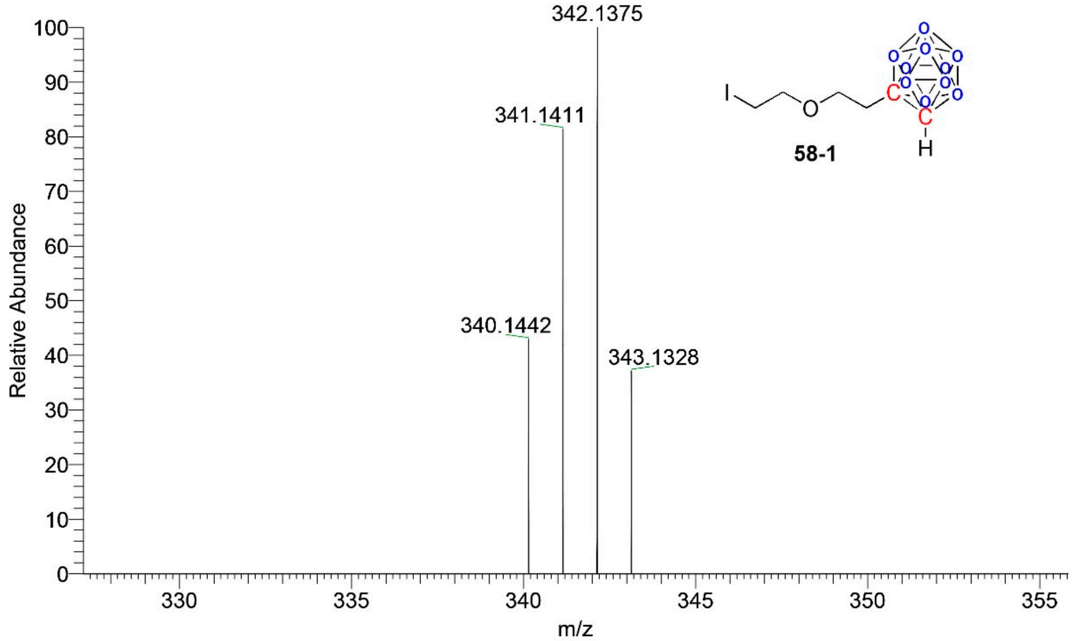

Figure S122 HR-MS (ESI/ion trap) spectrum of **58-1**

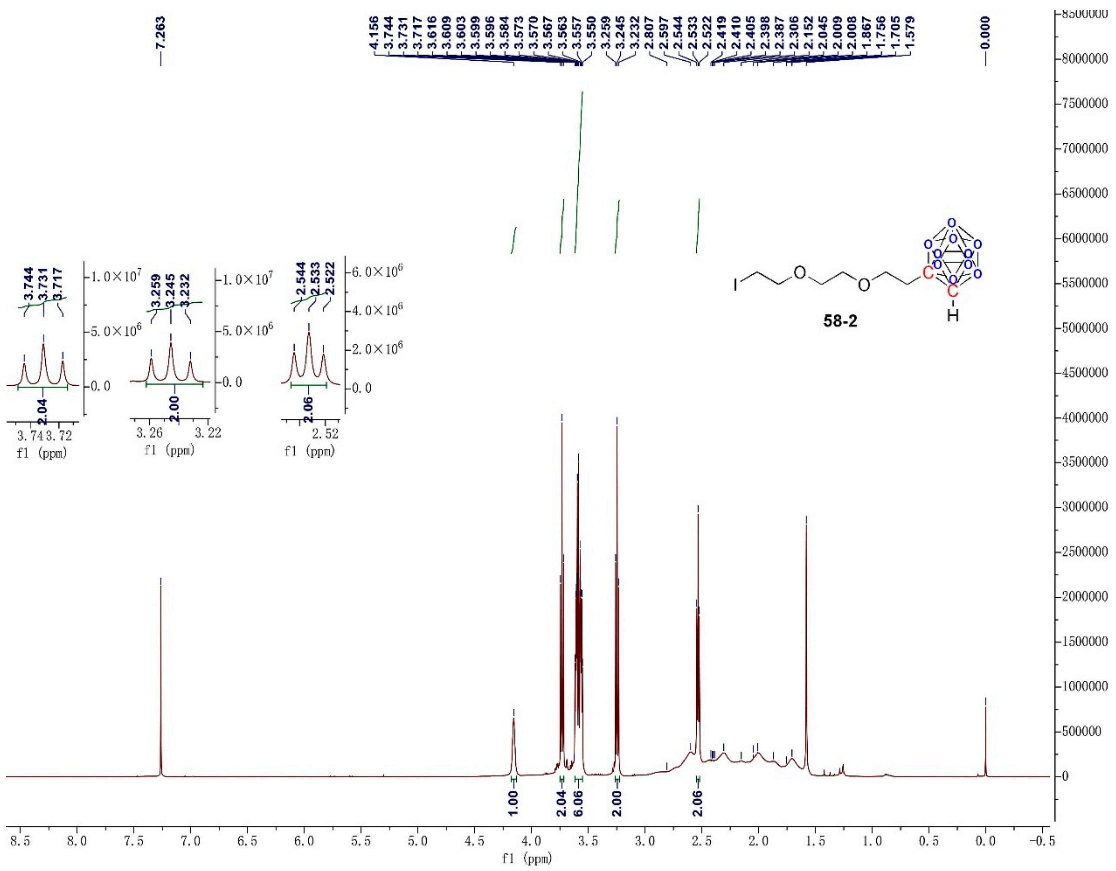

Figure S123 ^{1}H NMR spectrum of **58-2**

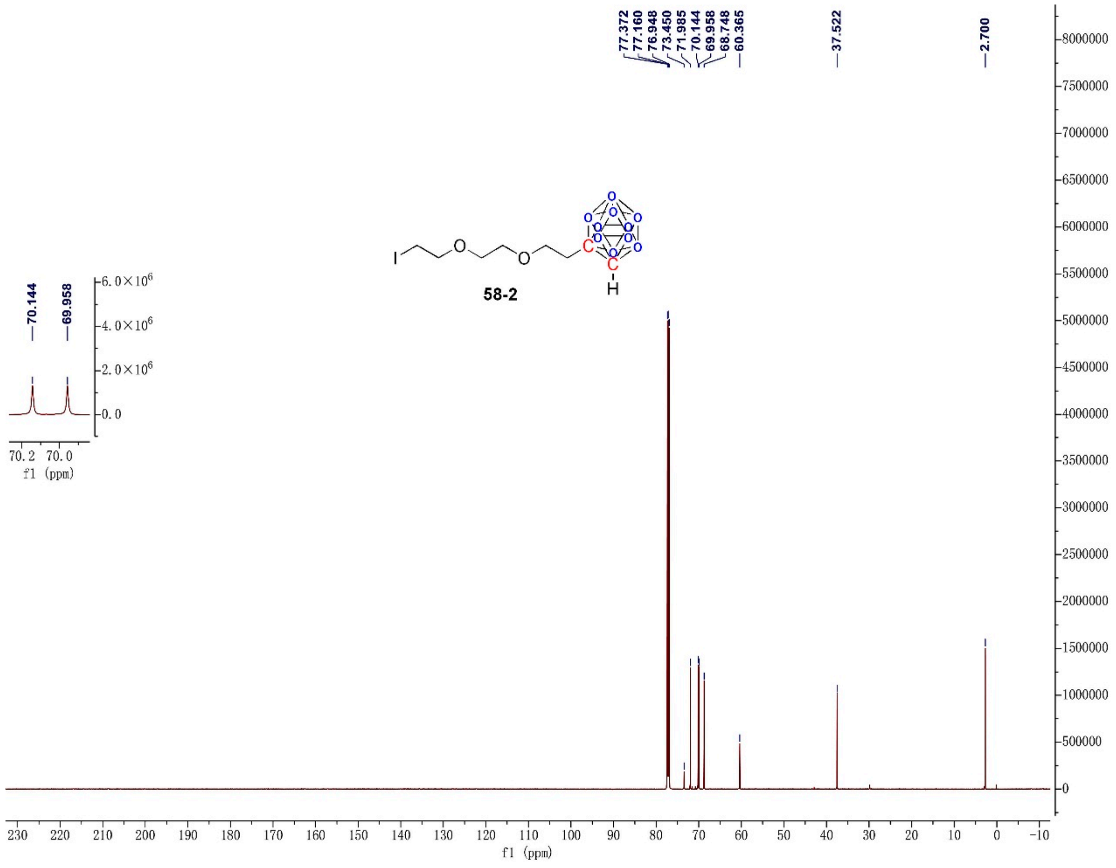

Figure S124 ^{13}C NMR spectrum of **58-2**

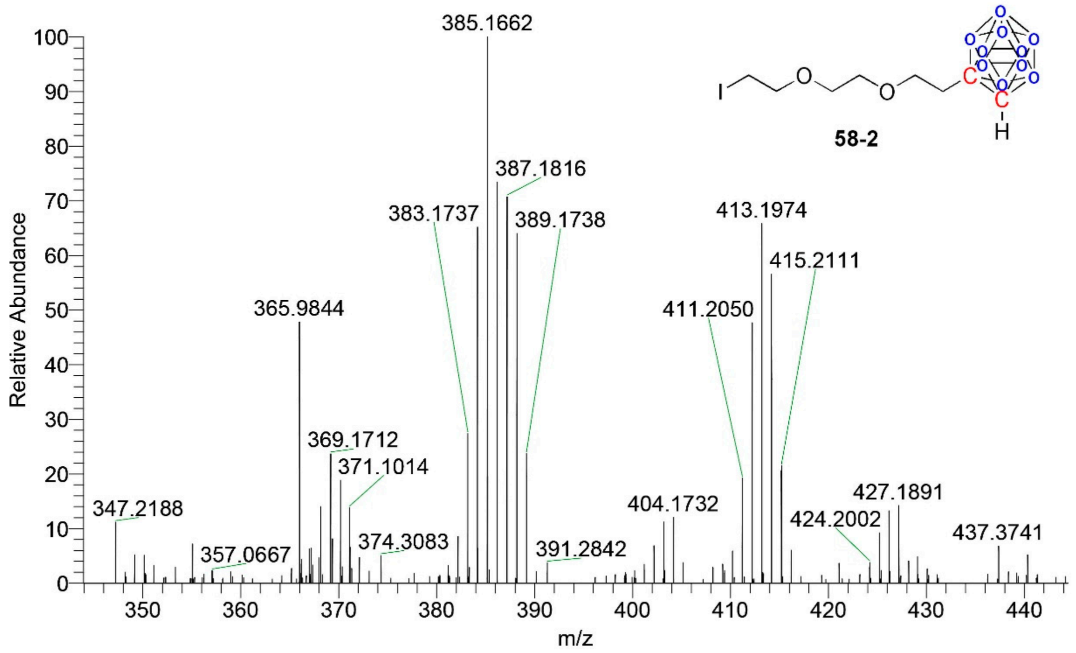

Figure S125 HR-MS (ESI/ion trap) spectrum of **58-2**

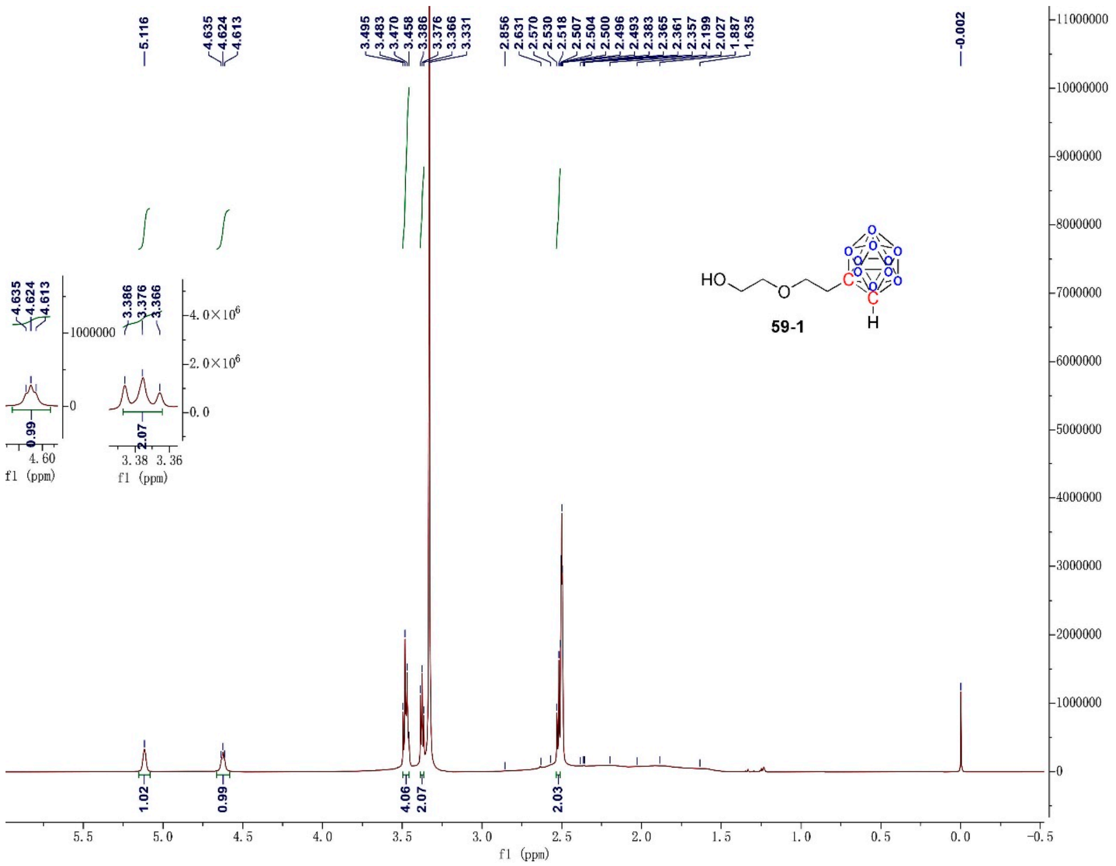

Figure S126 ^{1}H NMR spectrum of **59-1**

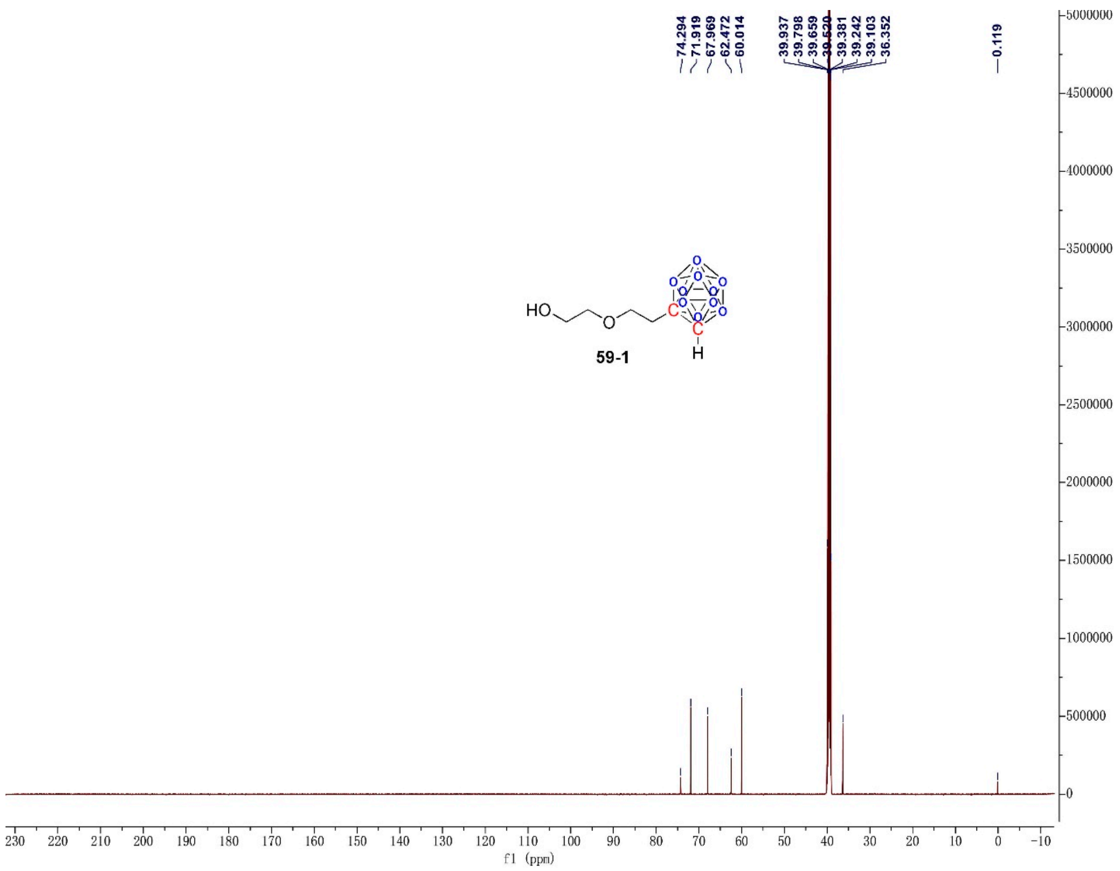

Figure S127 ^{13}C NMR spectrum of **59-1**

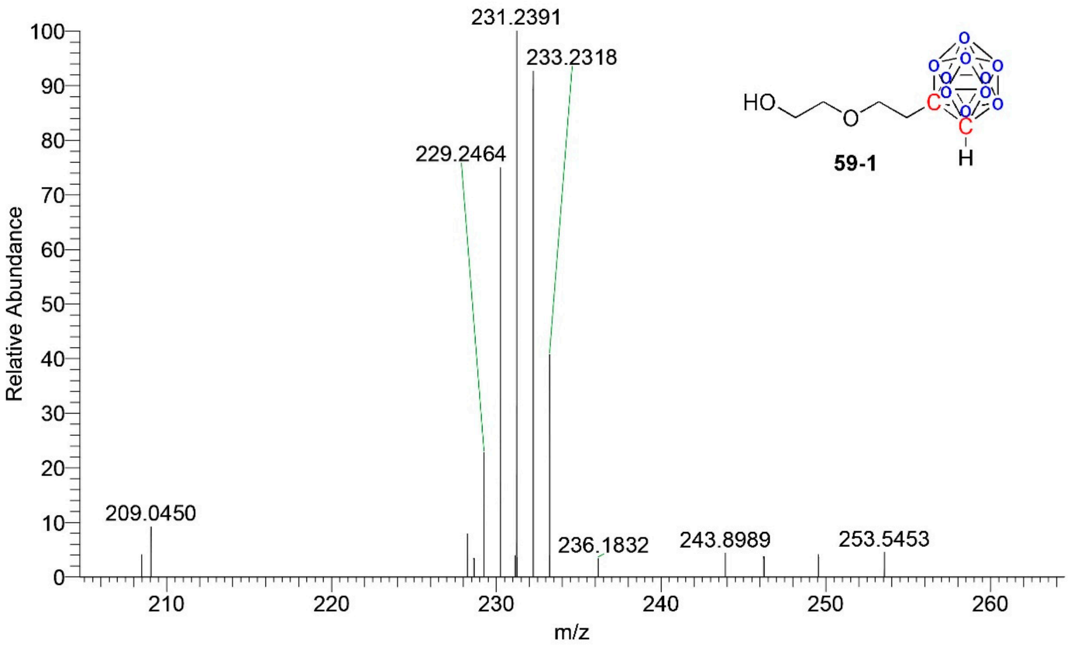

Figure S128 HR-MS (ESI/ion trap) spectrum of **59-1**

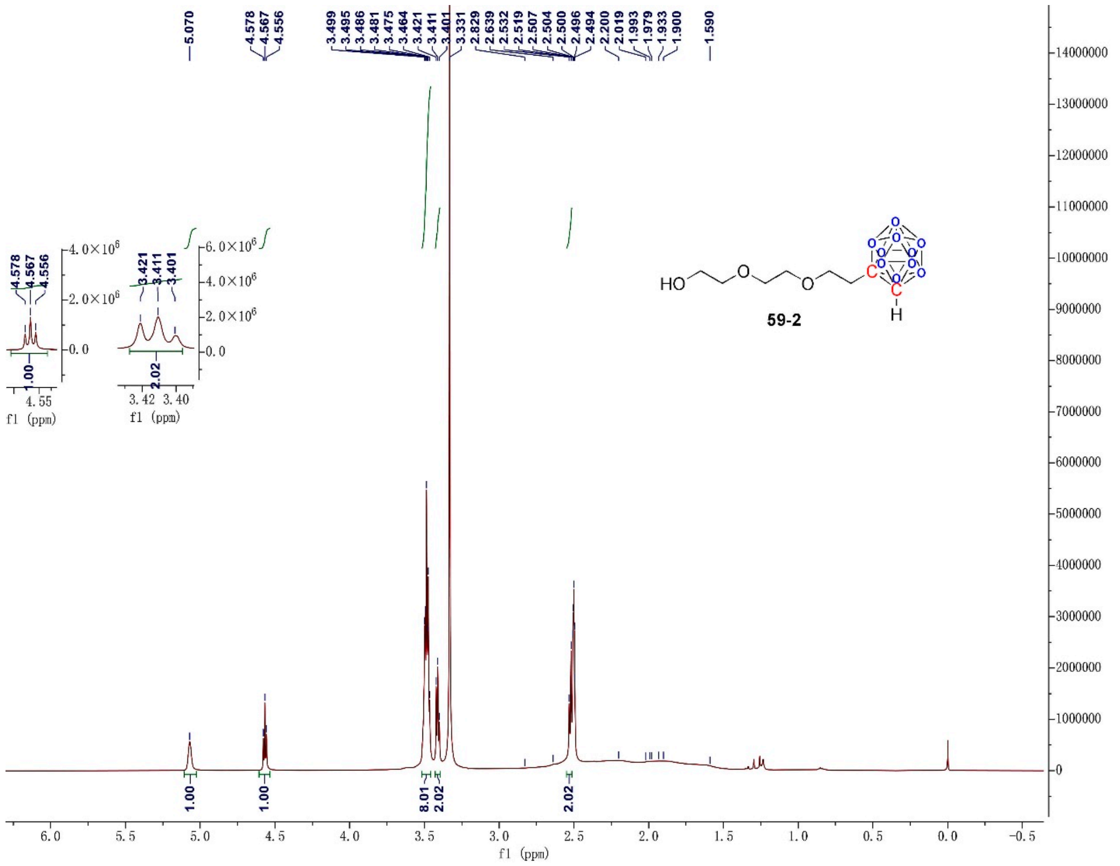

Figure S129 ^{1}H NMR spectrum of **59-2**

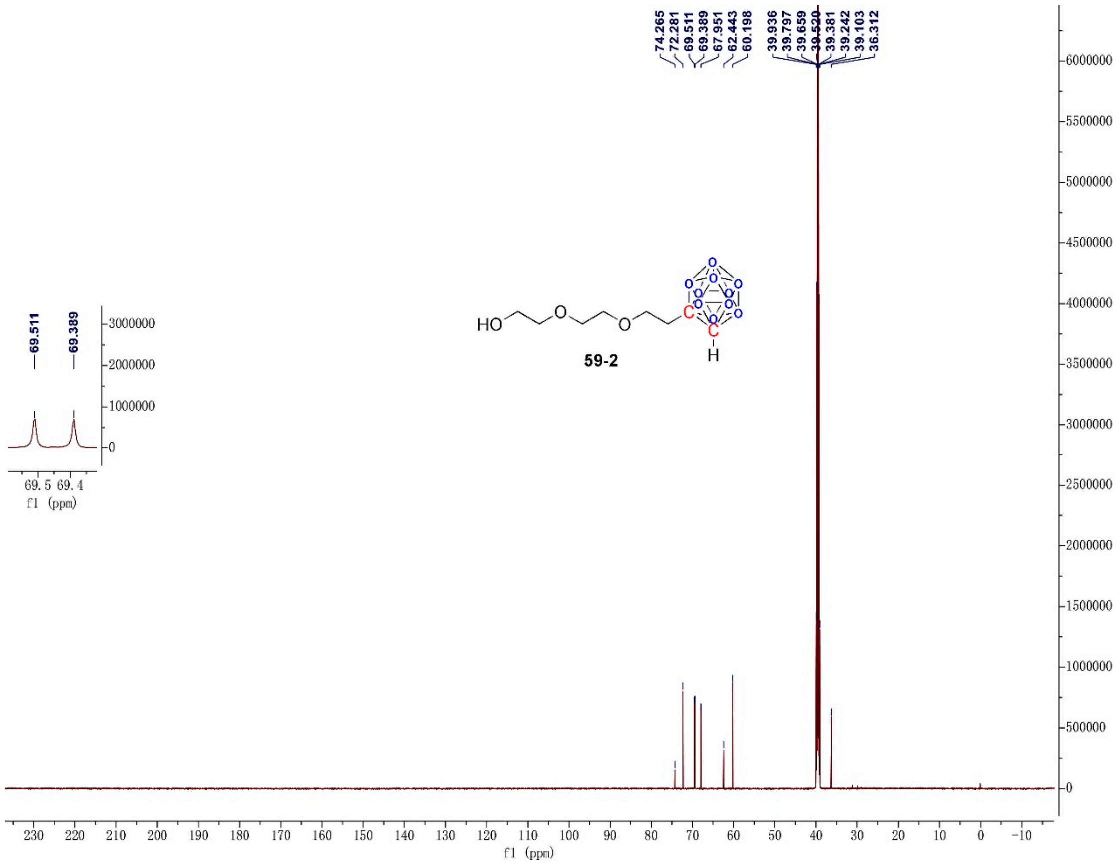

Figure S130 ^{13}C NMR spectrum of **59-2**

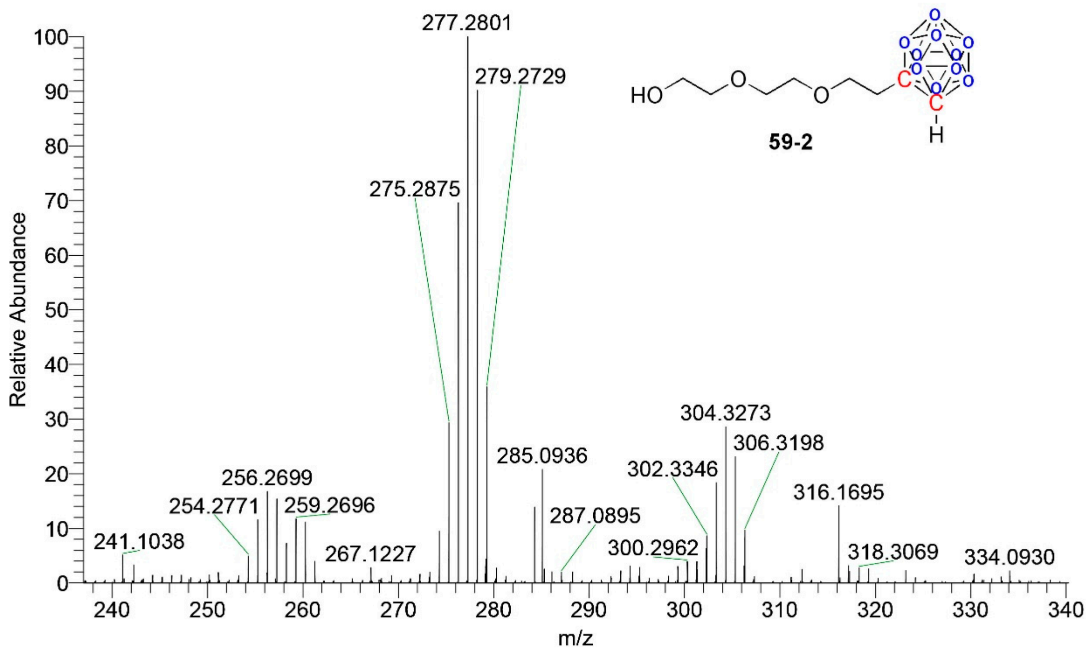

Figure S131 HR-MS (ESI/ion trap) spectrum of **59-2**

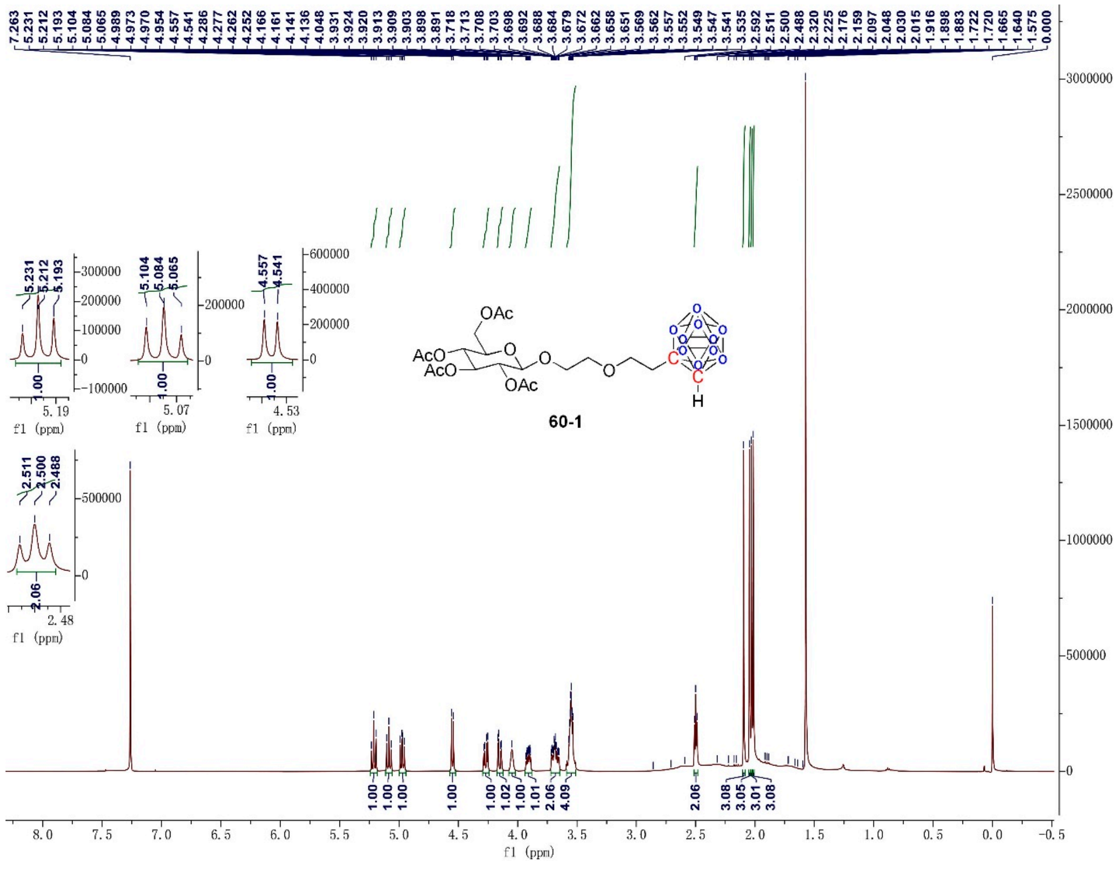

Figure S132 ^{1}H NMR spectrum of **60-1**

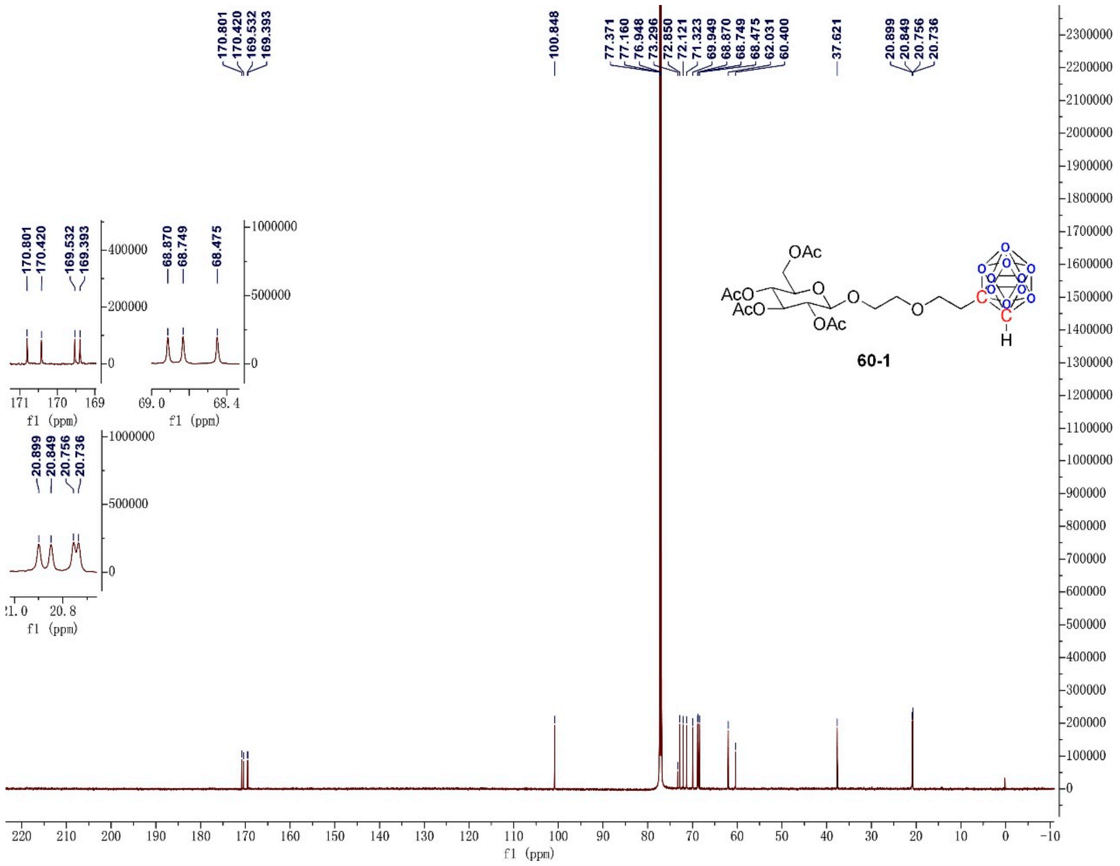

Figure S133 ^{13}C NMR spectrum of **60-1**

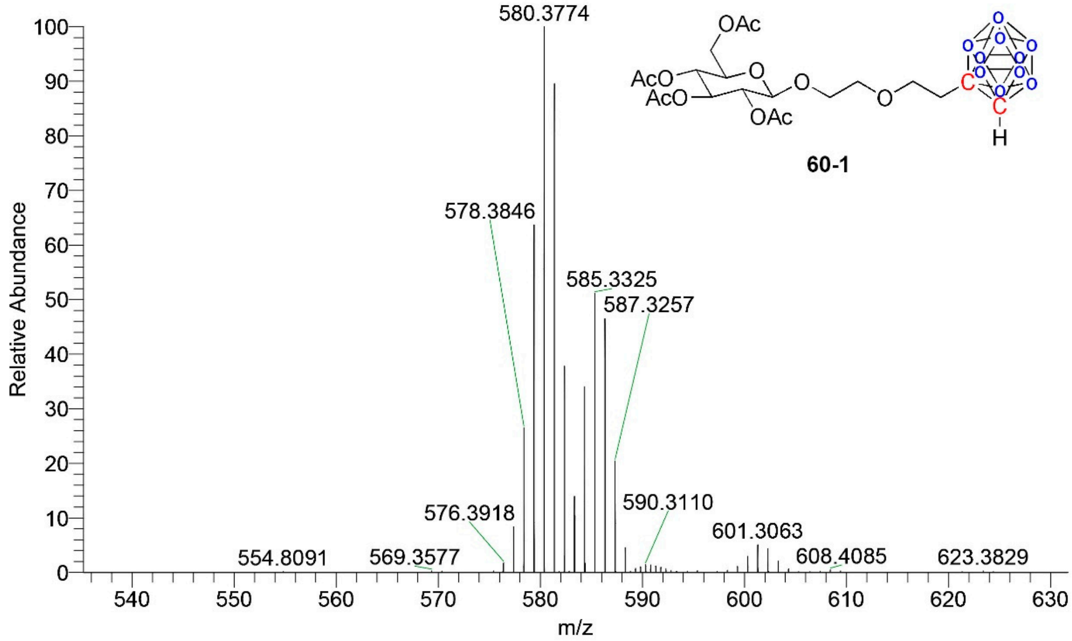

Figure S134 HR-MS (ESI/ion trap) spectrum of **60-1**

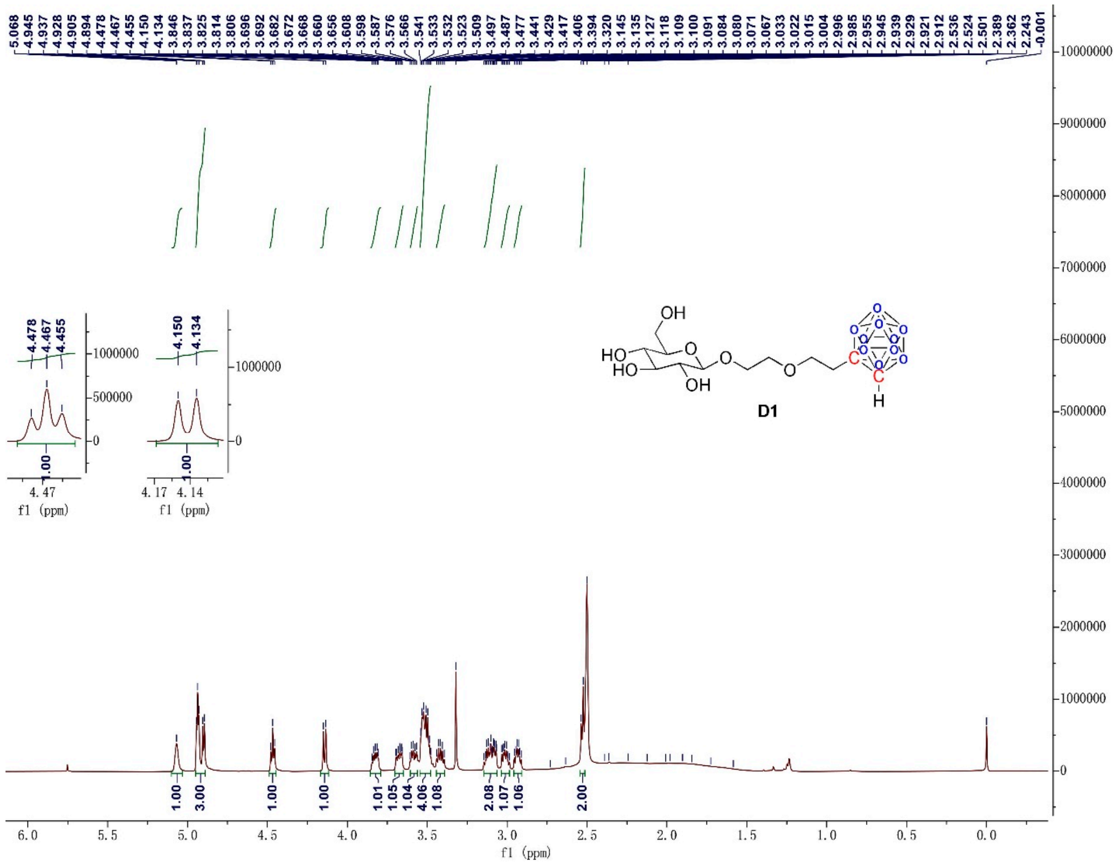

Figure S135 ^{1}H NMR spectrum of **D1**

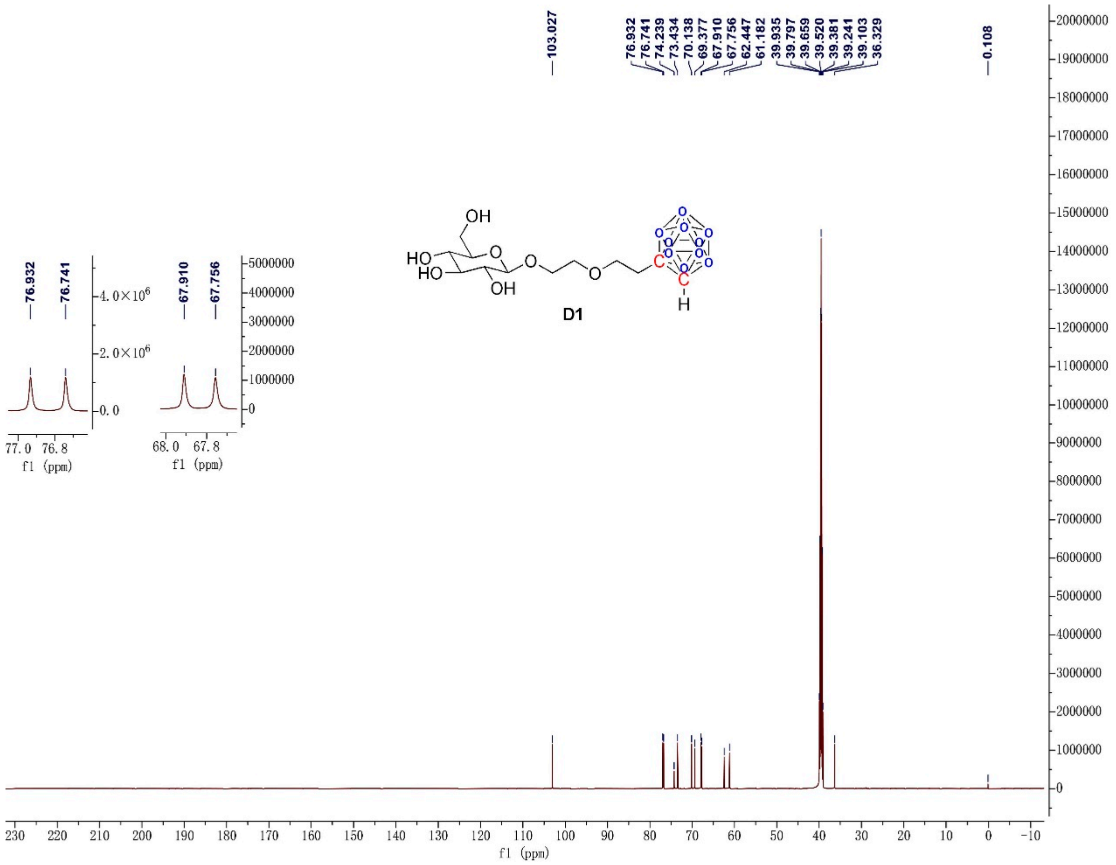

Figure S136 ^{13}C NMR spectrum of **D1**

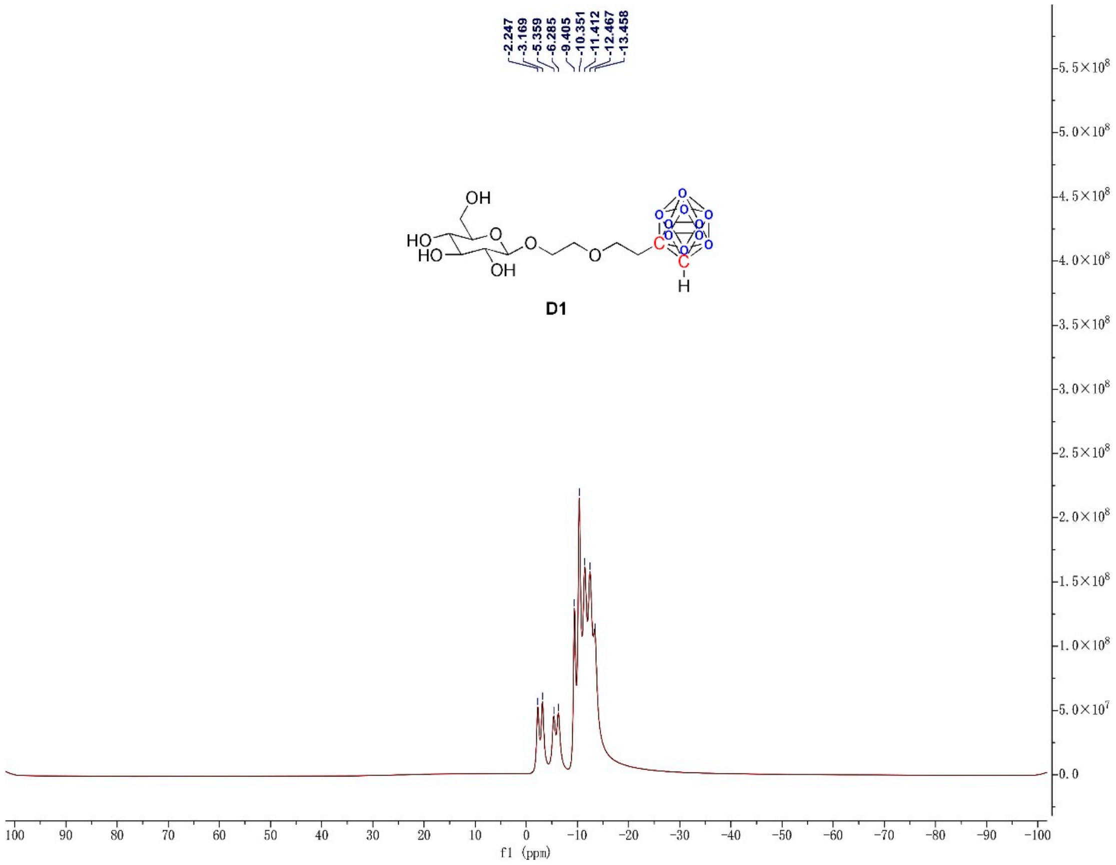

Figure S137 ^{11}B NMR spectrum of **D1**

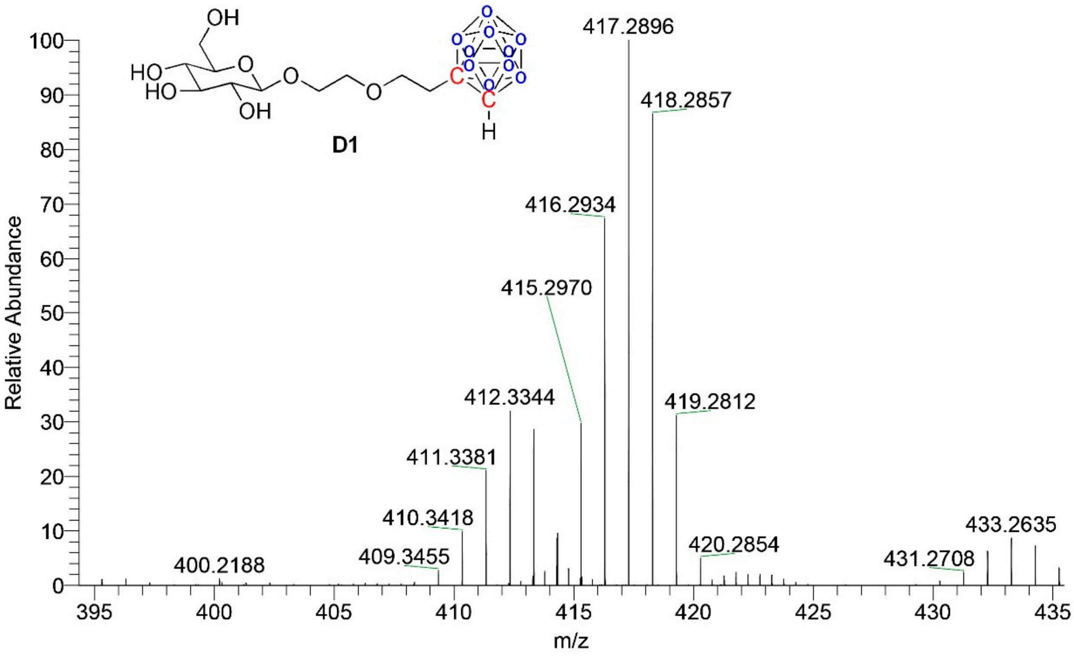

Figure S138 HR-MS (ESI/ion trap) spectrum of **D1**

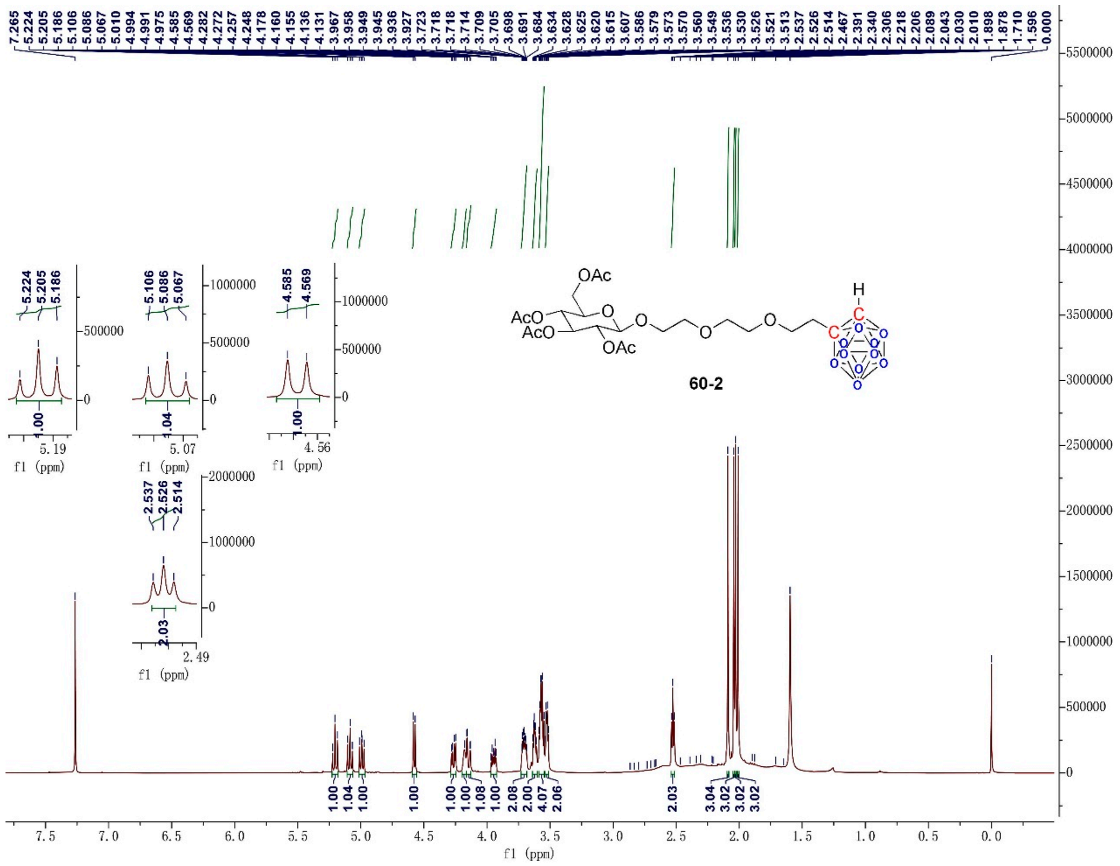

Figure S139 ^{1}H NMR spectrum of **60-2**

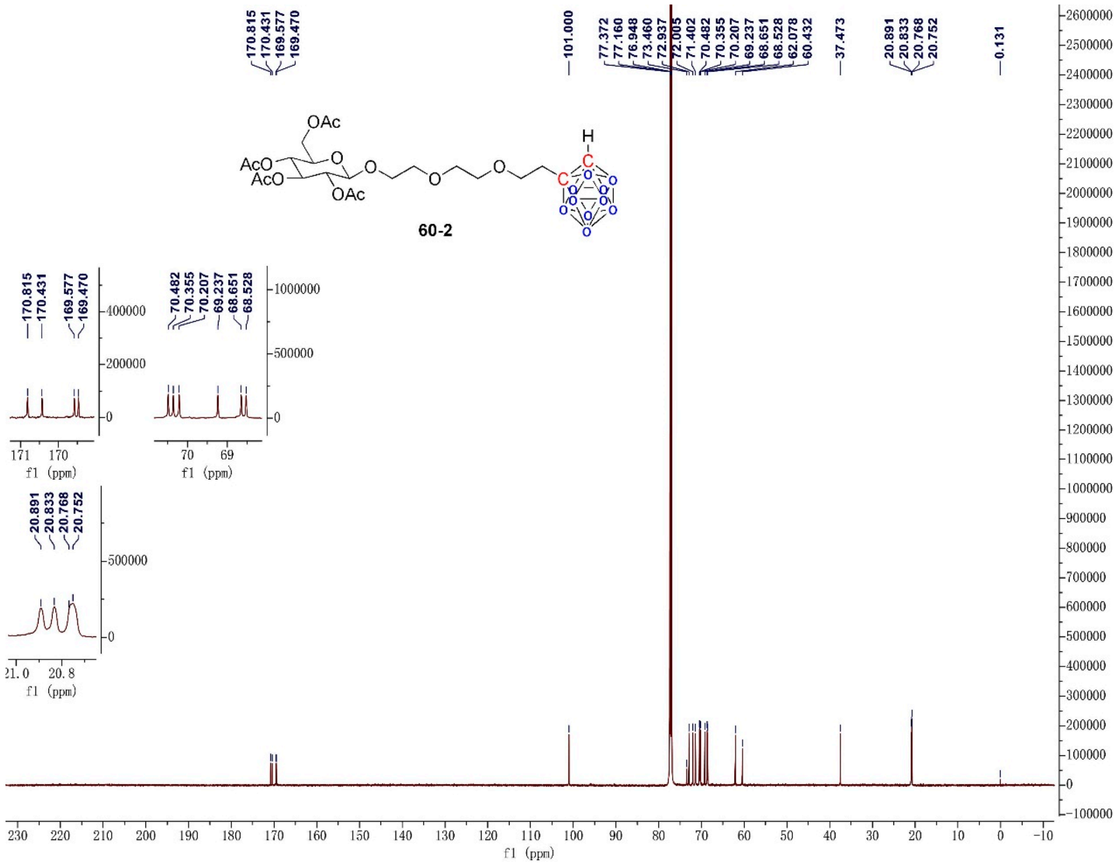

Figure S140 ^{13}C NMR spectrum of **60-2**

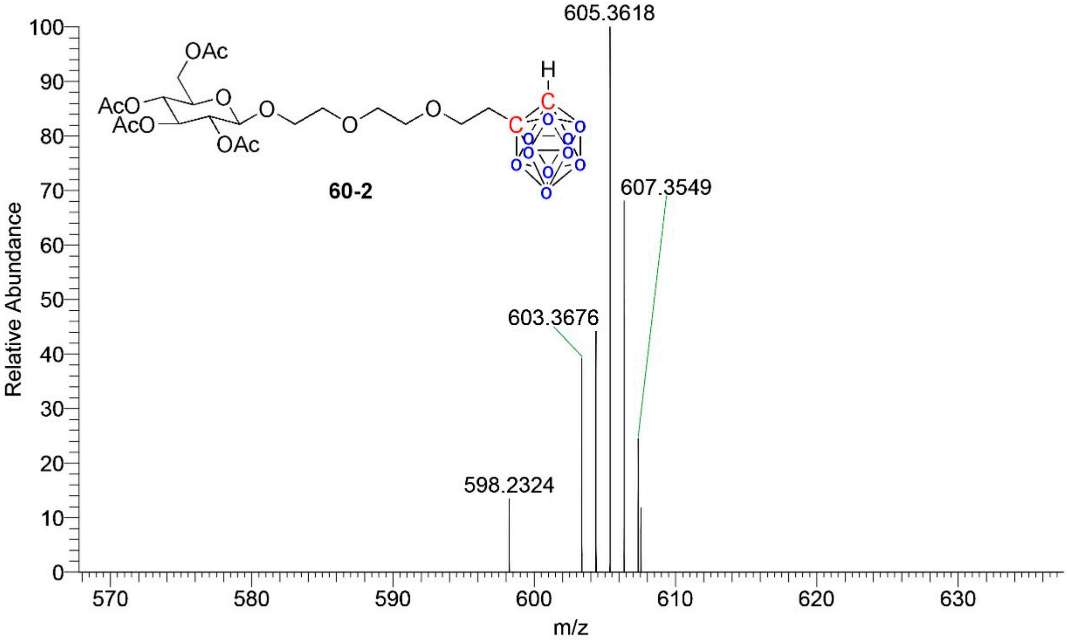

Figure S141 HR-MS (ESI/ion trap) spectrum of **60-2**

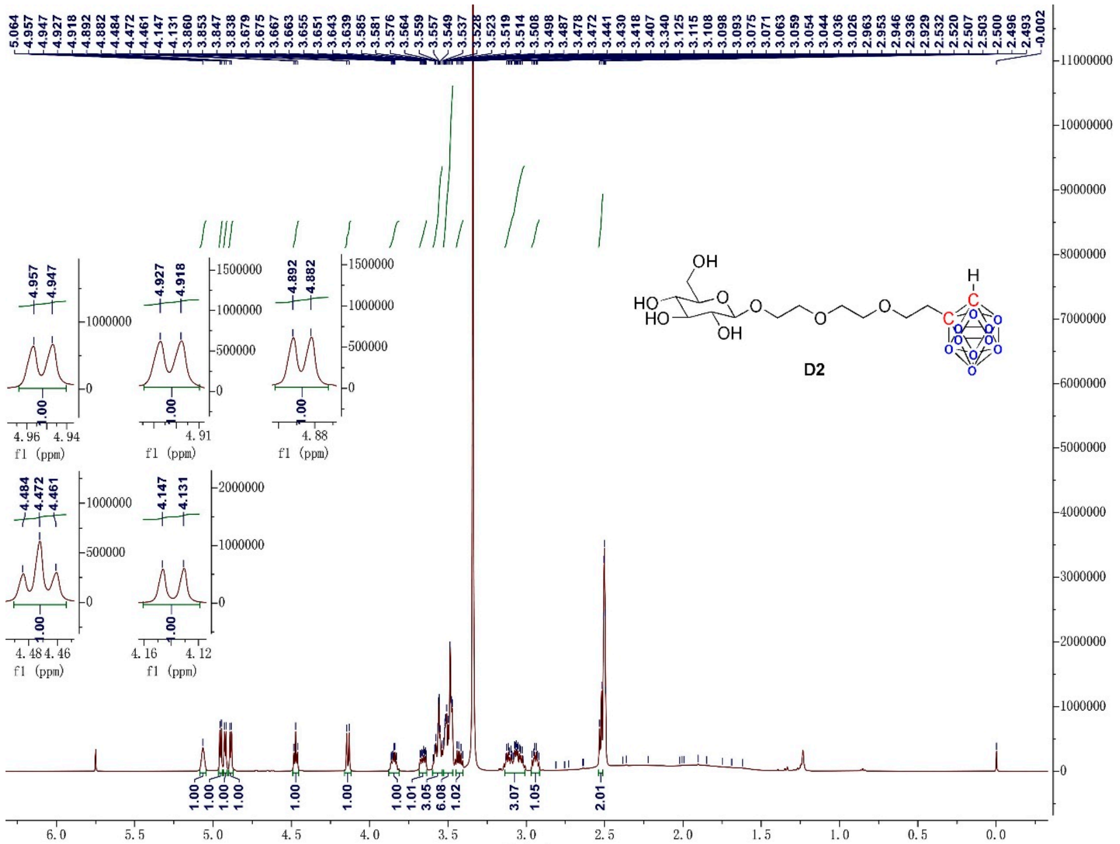

Figure S142 ^{1}H NMR spectrum of **D2**

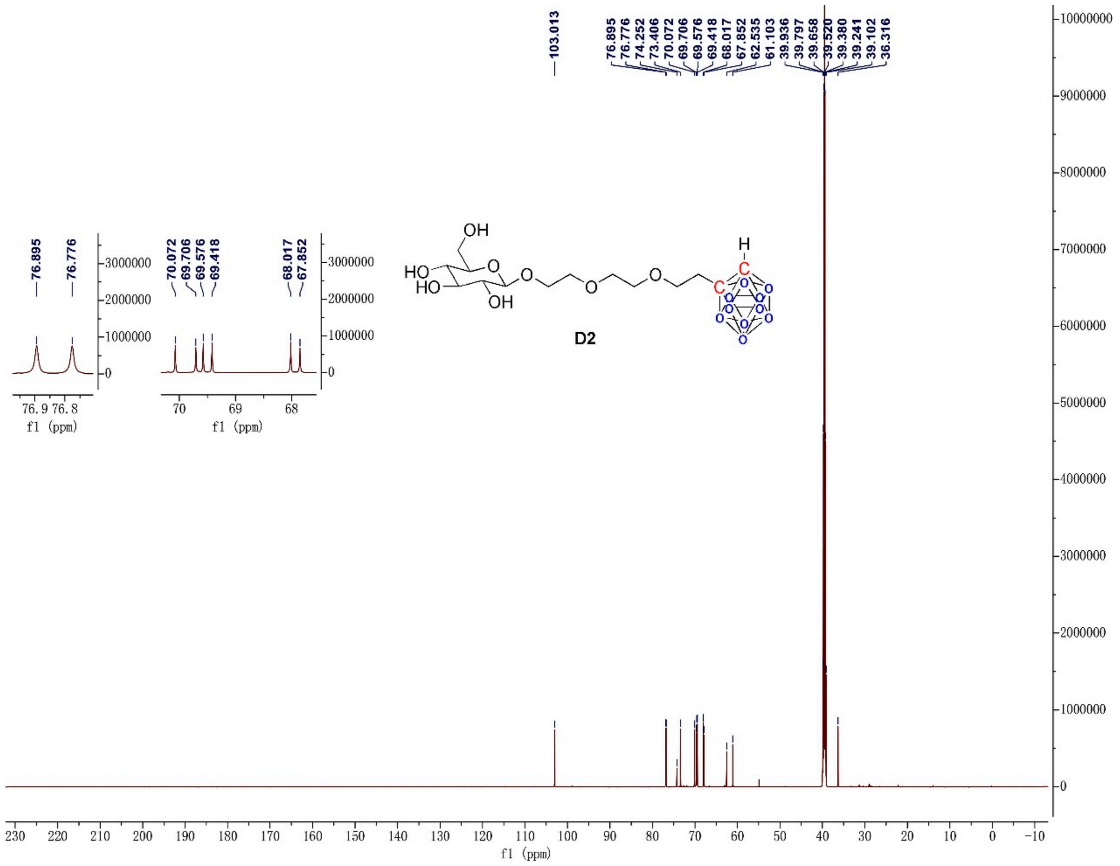

Figure S143 ^{13}C NMR spectrum of **D2**

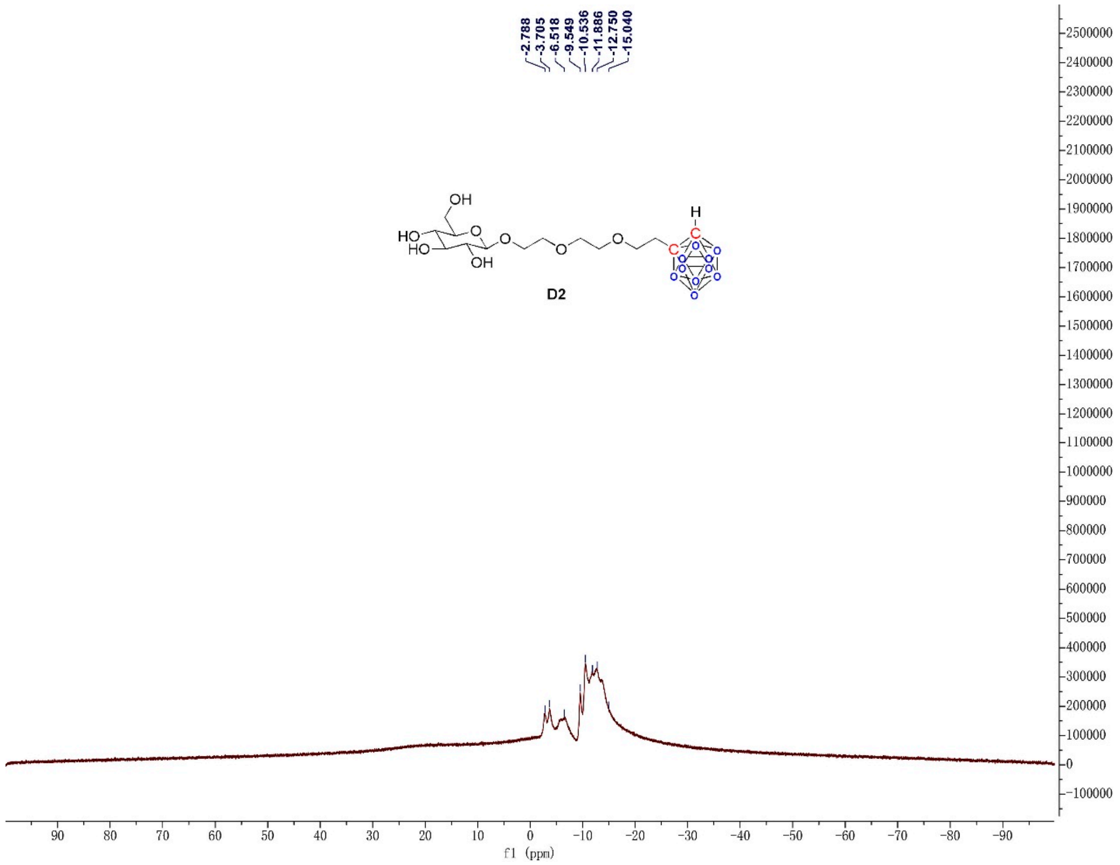

Figure S144 ^{11}B NMR spectrum of **D2**

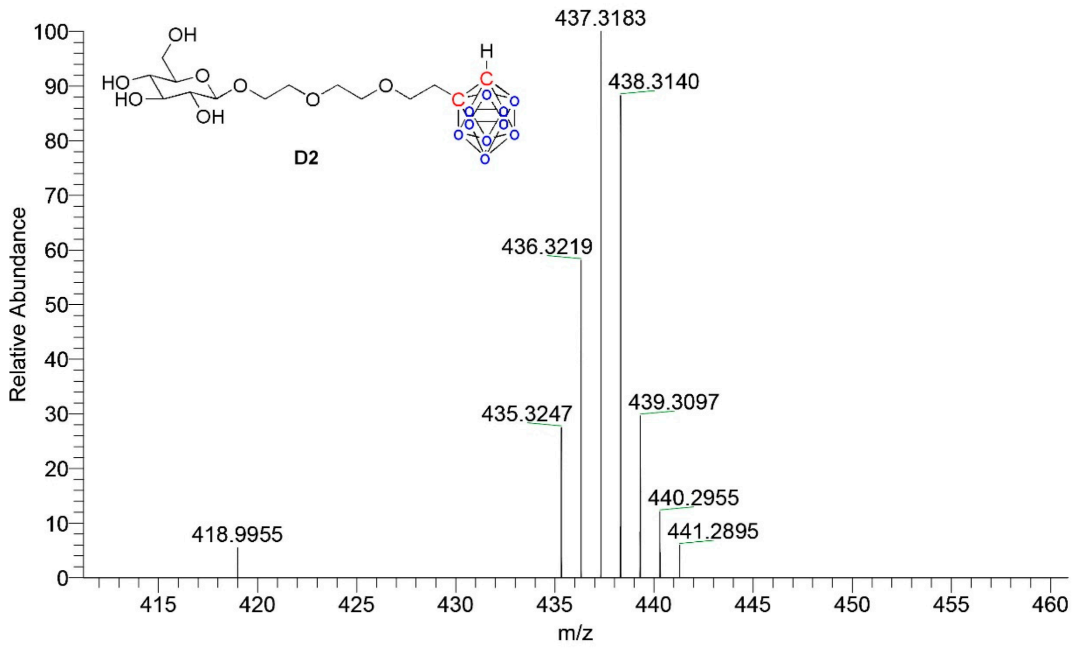

Figure S145 HR-MS (ESI/ion trap) spectrum of **D2**

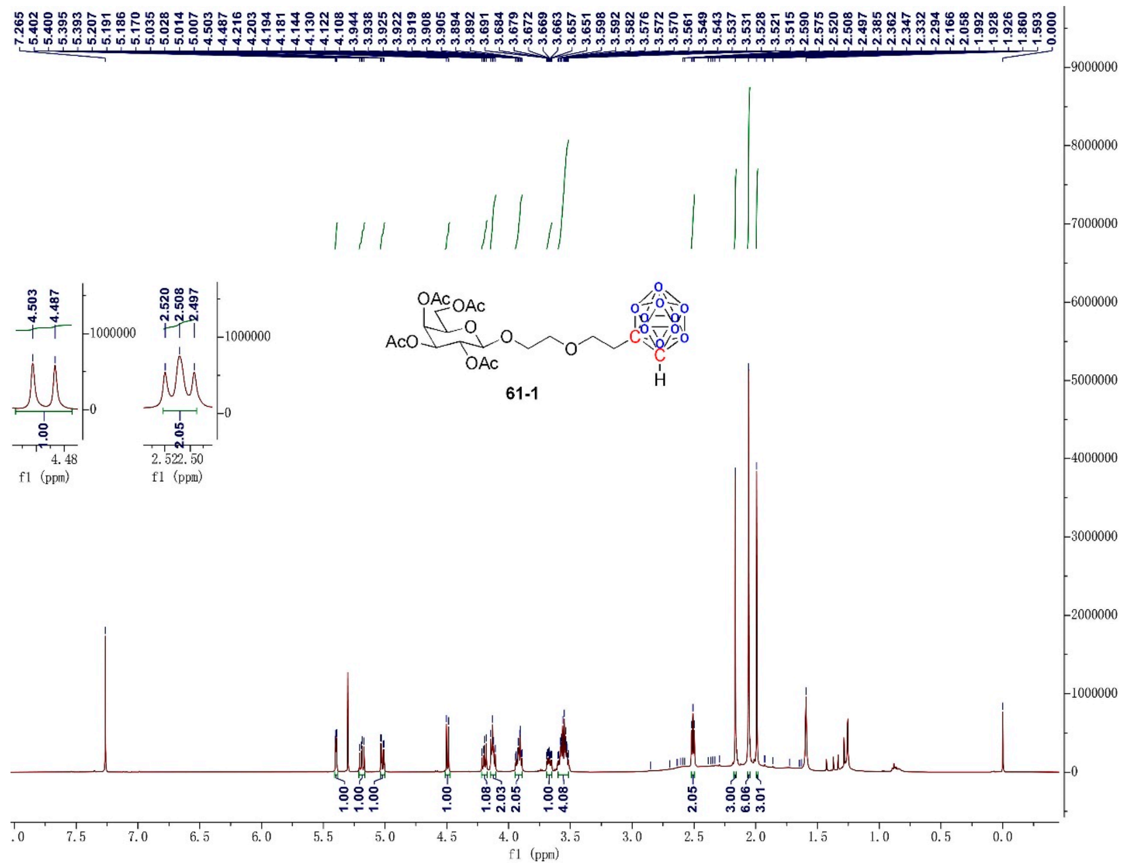

Figure S146 ^{1}H NMR spectrum of **61-1**

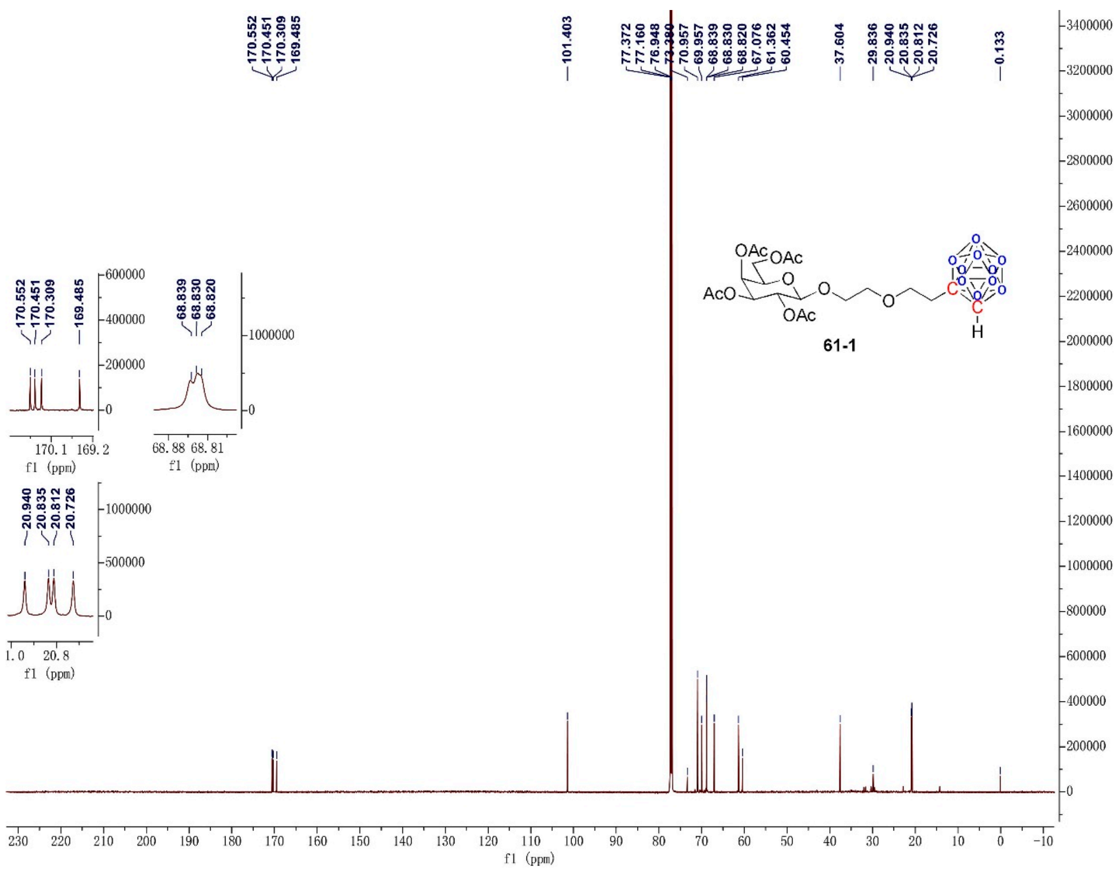

Figure S147 ^{13}C NMR spectrum of **61-1**

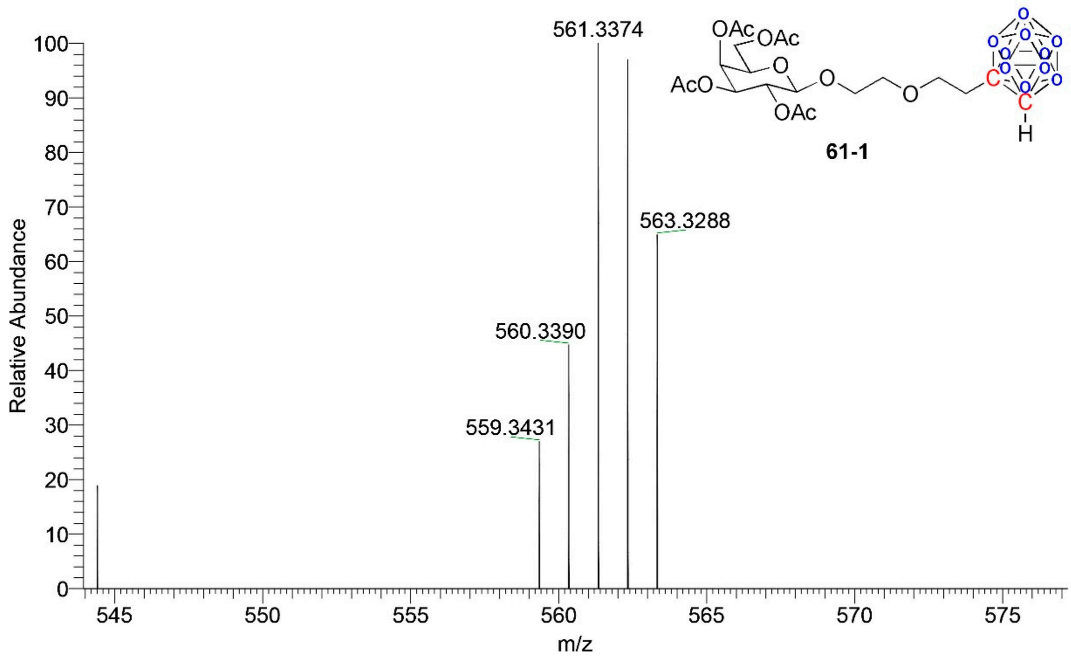

Figure S148 HR-MS (ESI/ion trap) spectrum of **61-1**

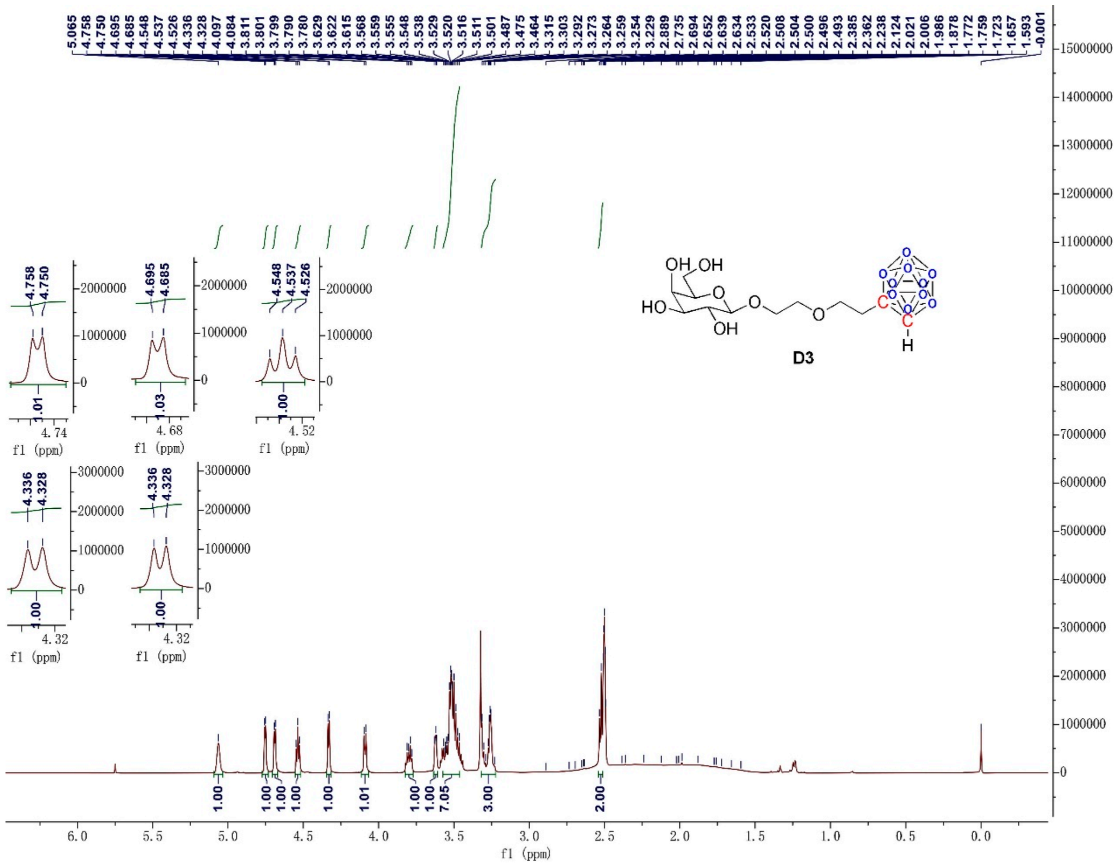

Figure S149 ^{1}H NMR spectrum of **D3**

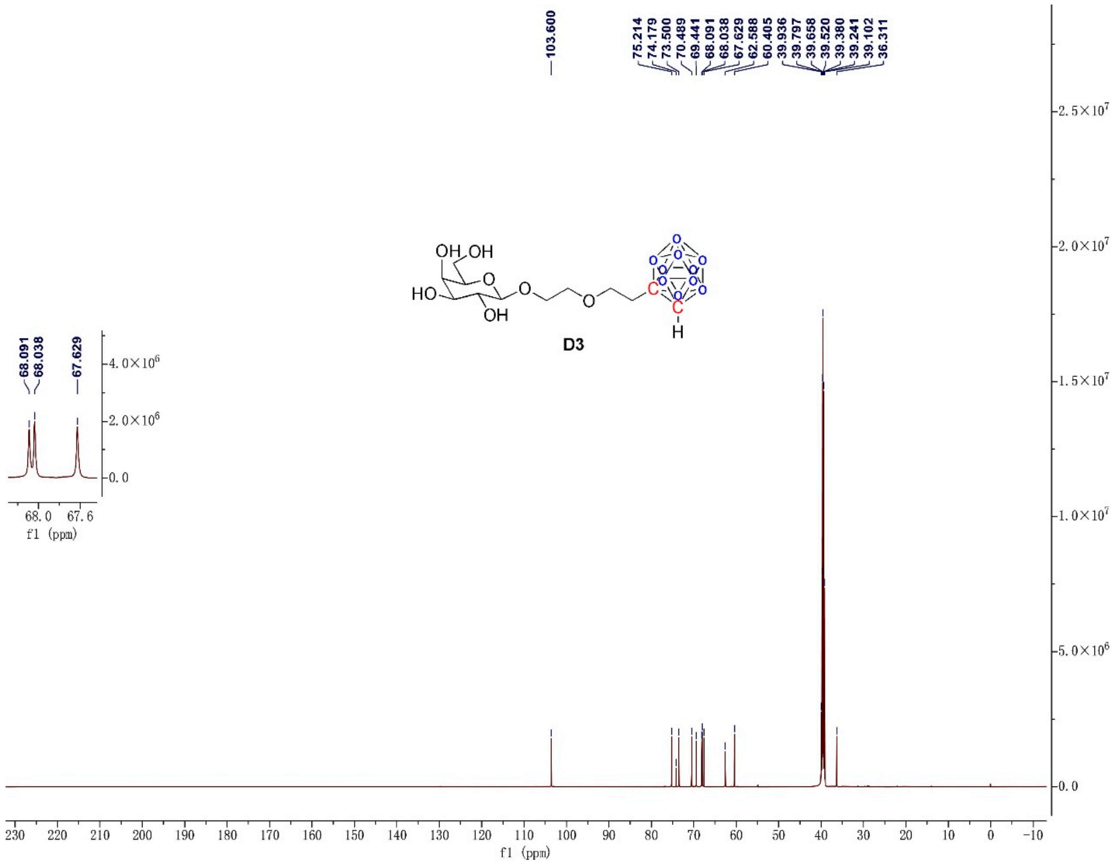

Figure S150 ^{13}C NMR spectrum of **D3**

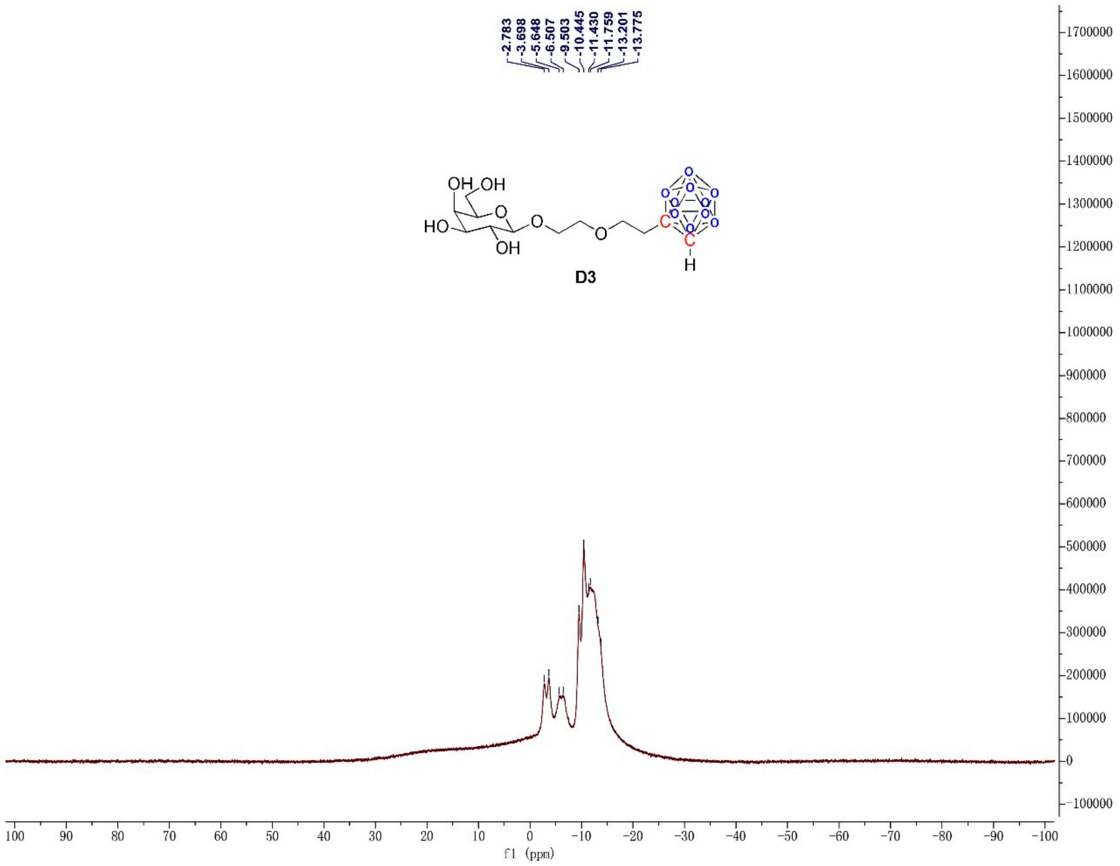

Figure S151 ^{11}B NMR spectrum of **D3**

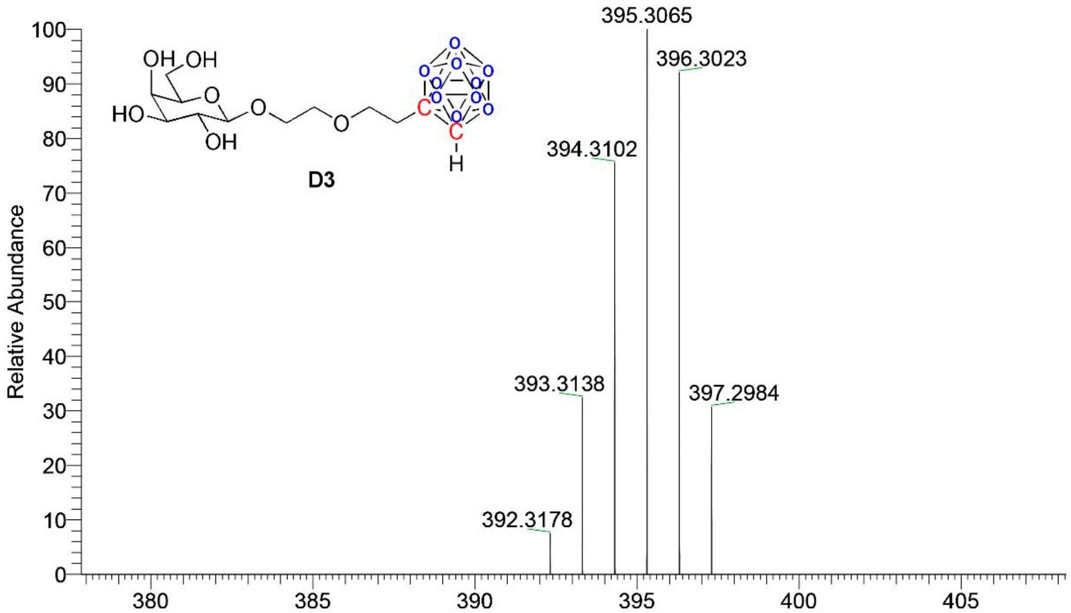

Figure S152 HR-MS (ESI/ion trap) spectrum of **D3**

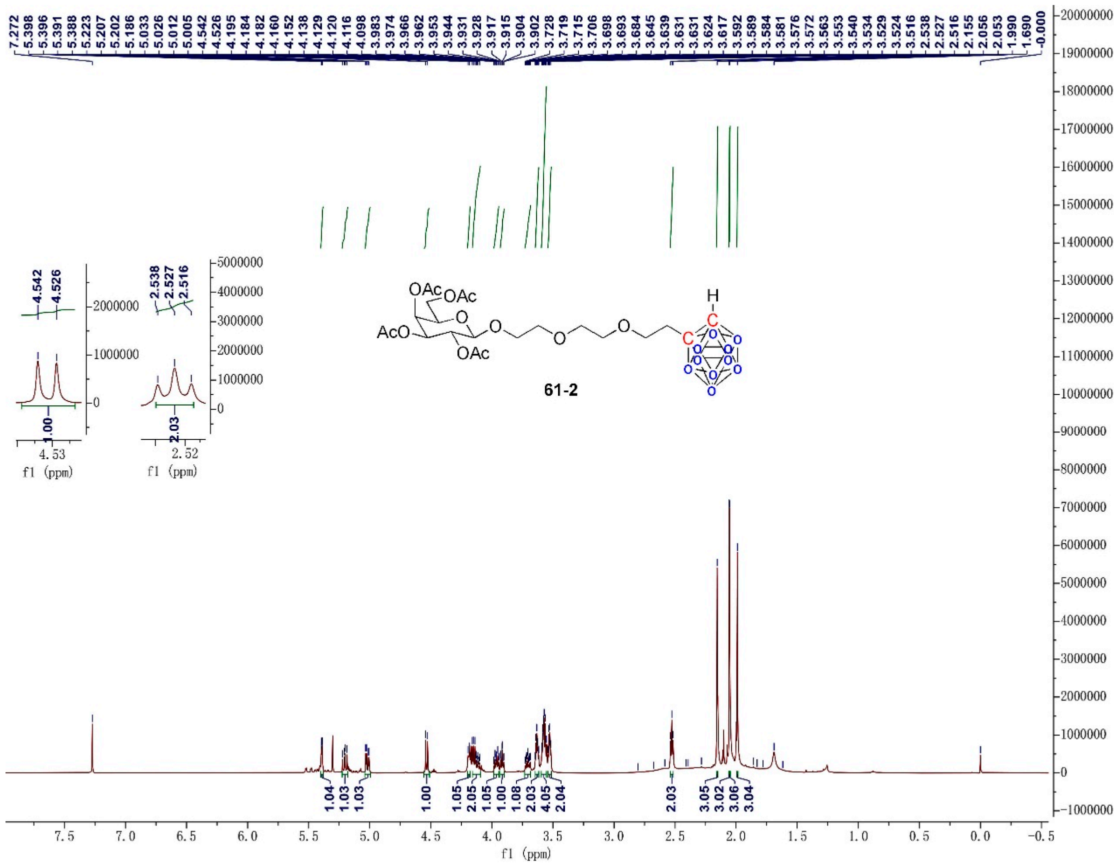

Figure S153 ^{1}H NMR spectrum of **61-2**

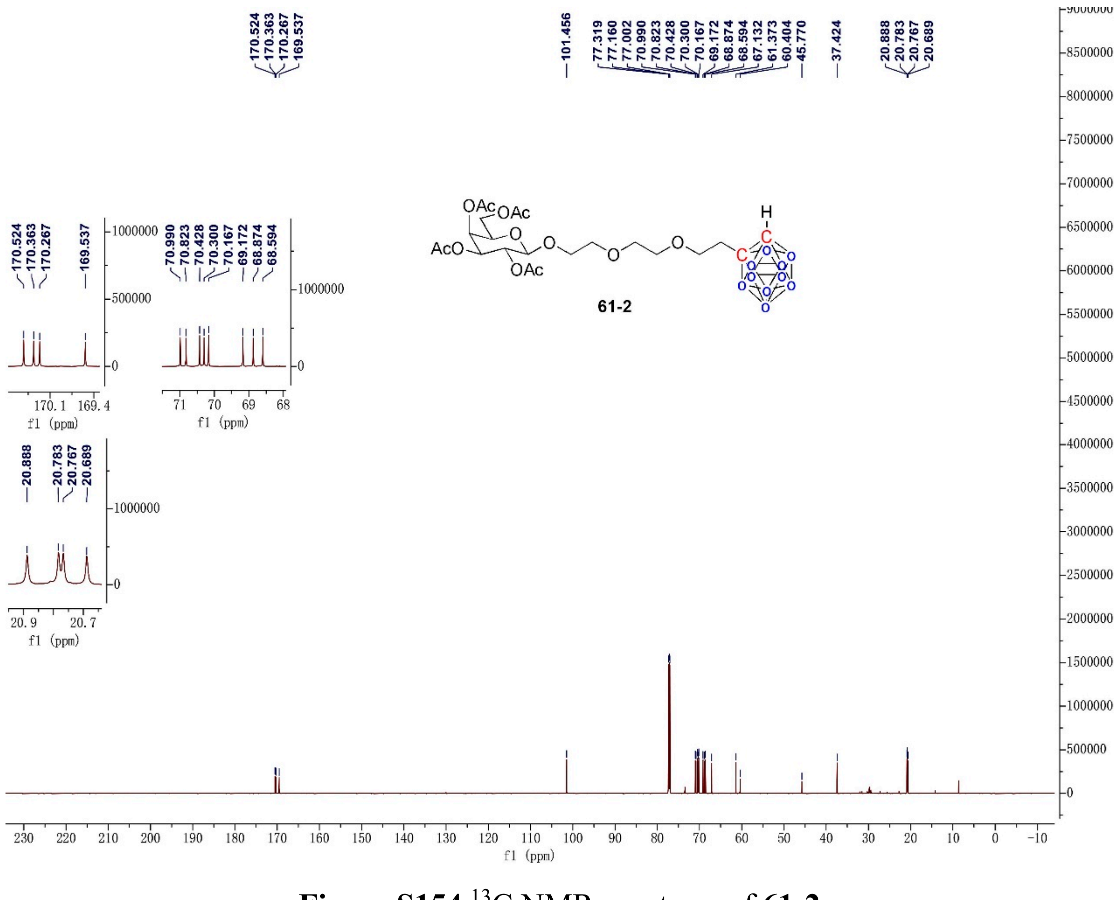

Figure S154 ^{13}C NMR spectrum of **61-2**

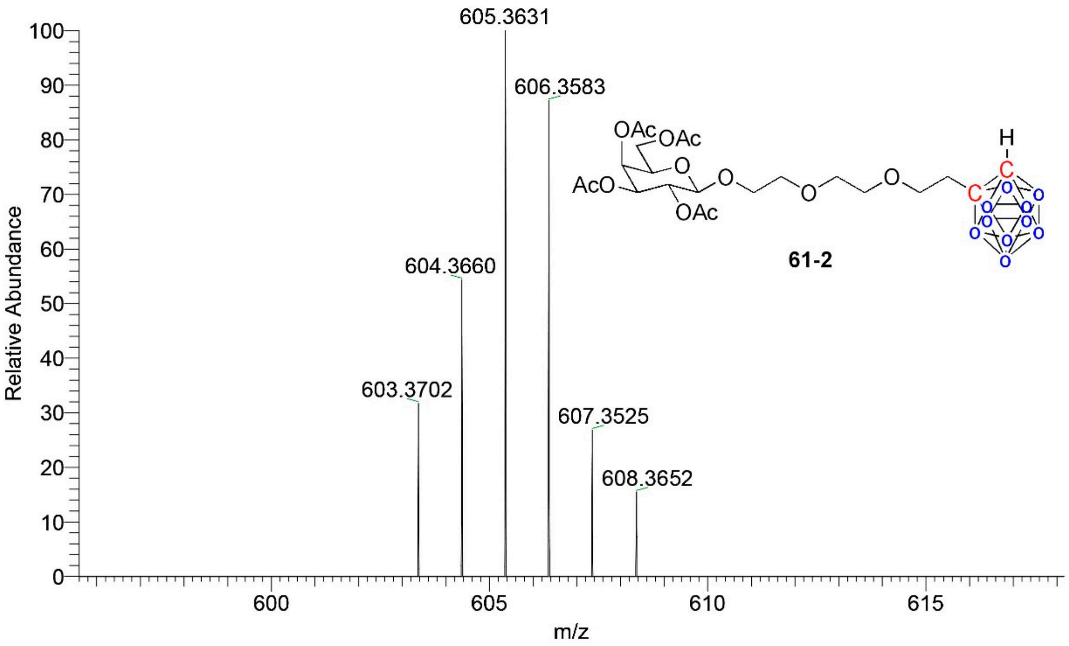

Figure S155 HR-MS (ESI/ion trap) spectrum of **61-2**

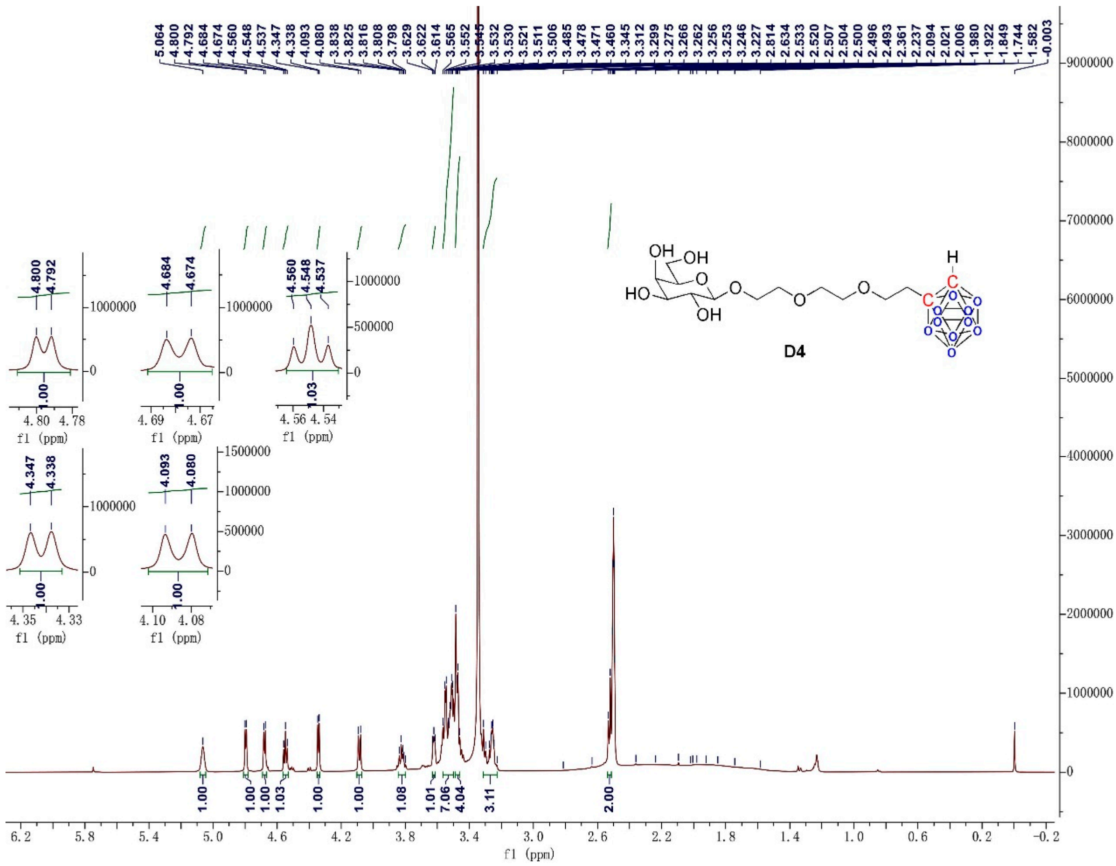

Figure S156 ^{1}H NMR spectrum of **D4**

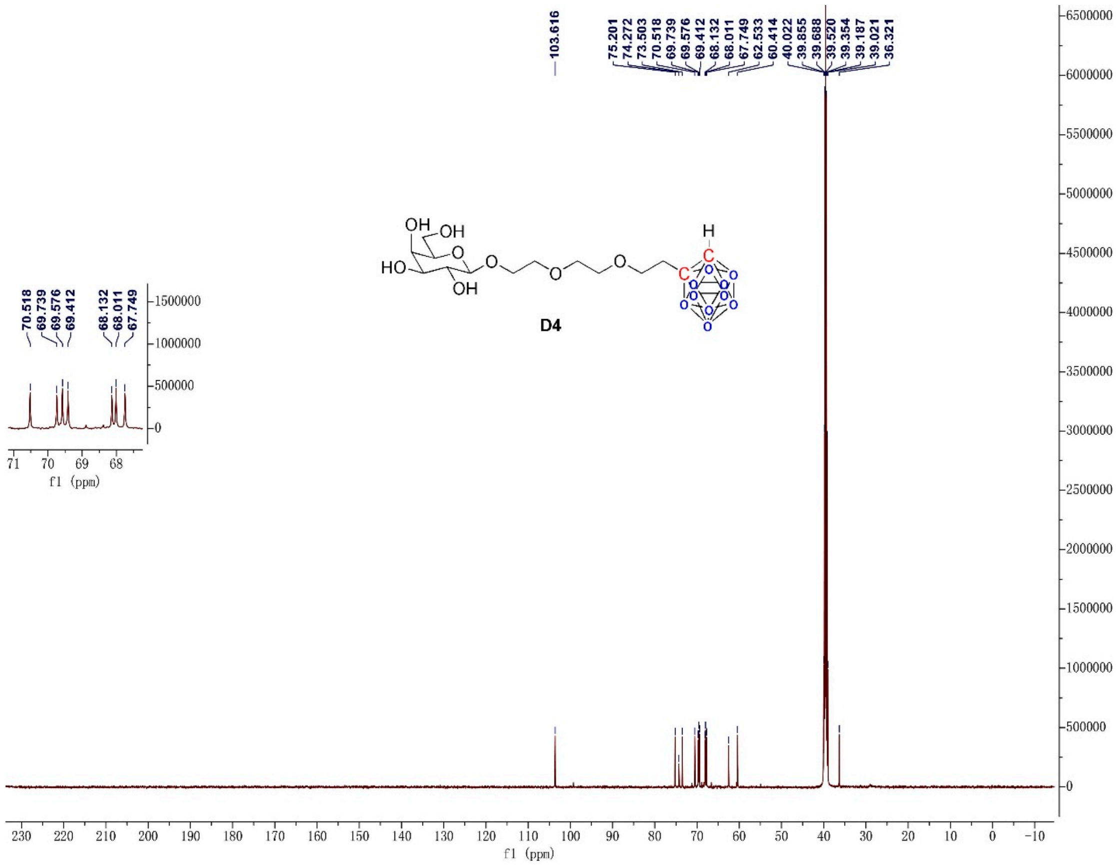

Figure S157 ^{13}C NMR spectrum of **D4**

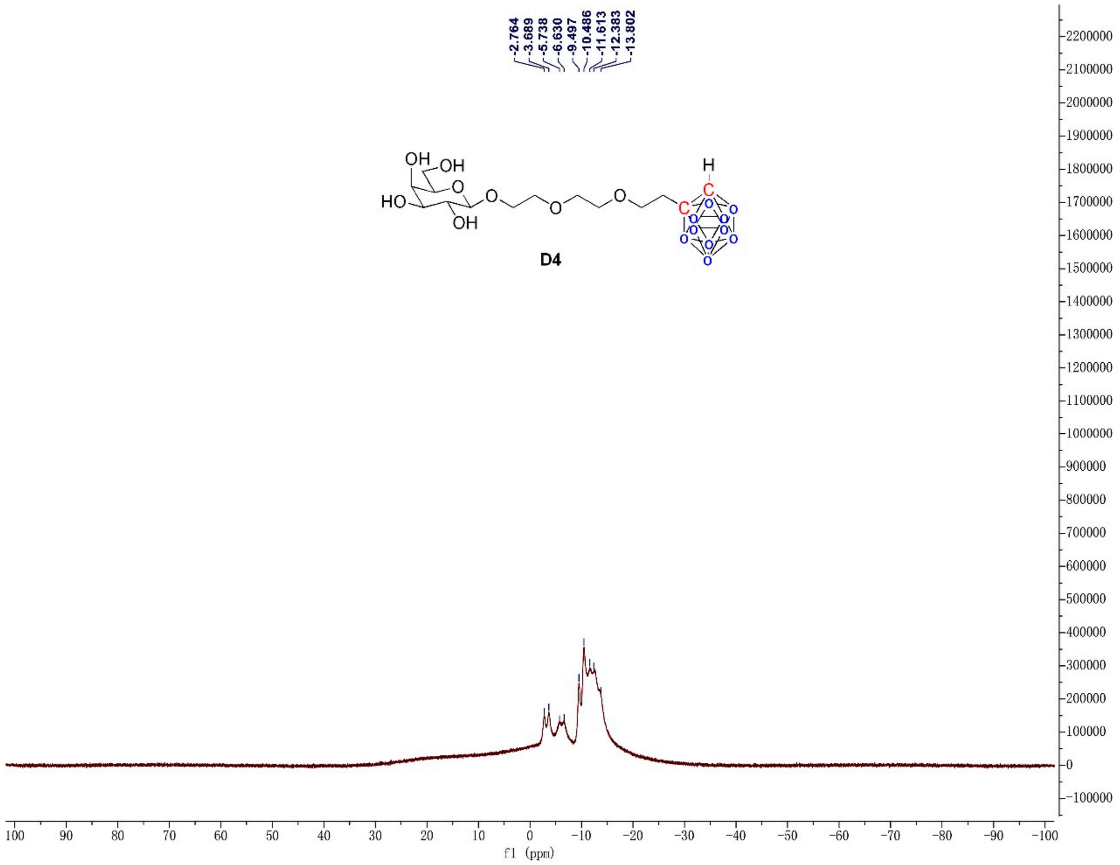

Figure S158 ^{11}B NMR spectrum of **D4**

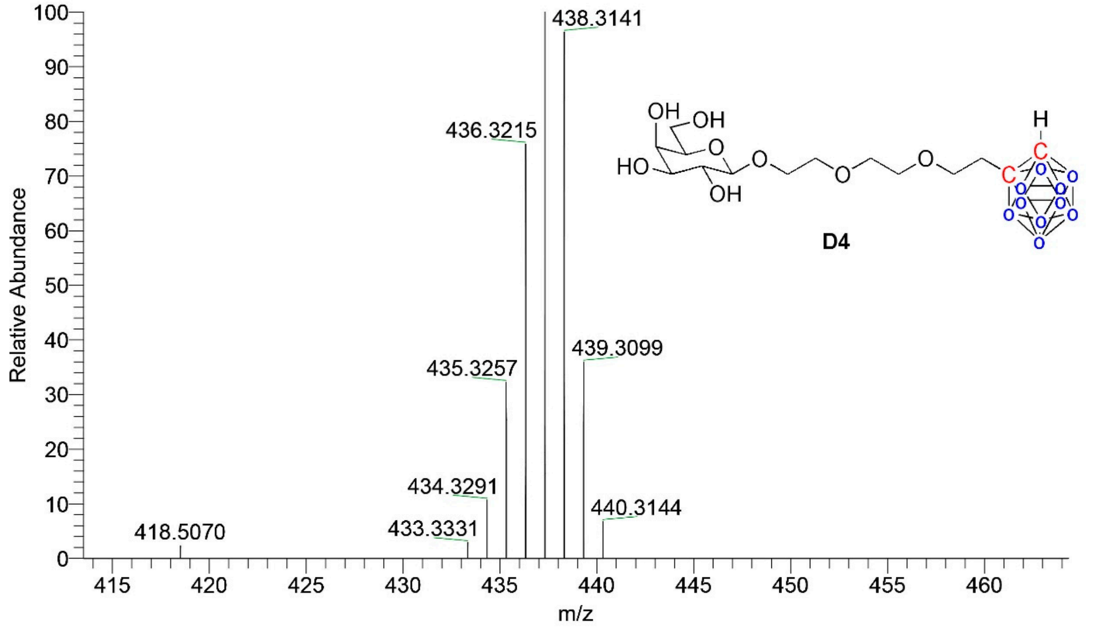

Figure S159 HR-MS (ESI/ion trap) spectrum of **D4**

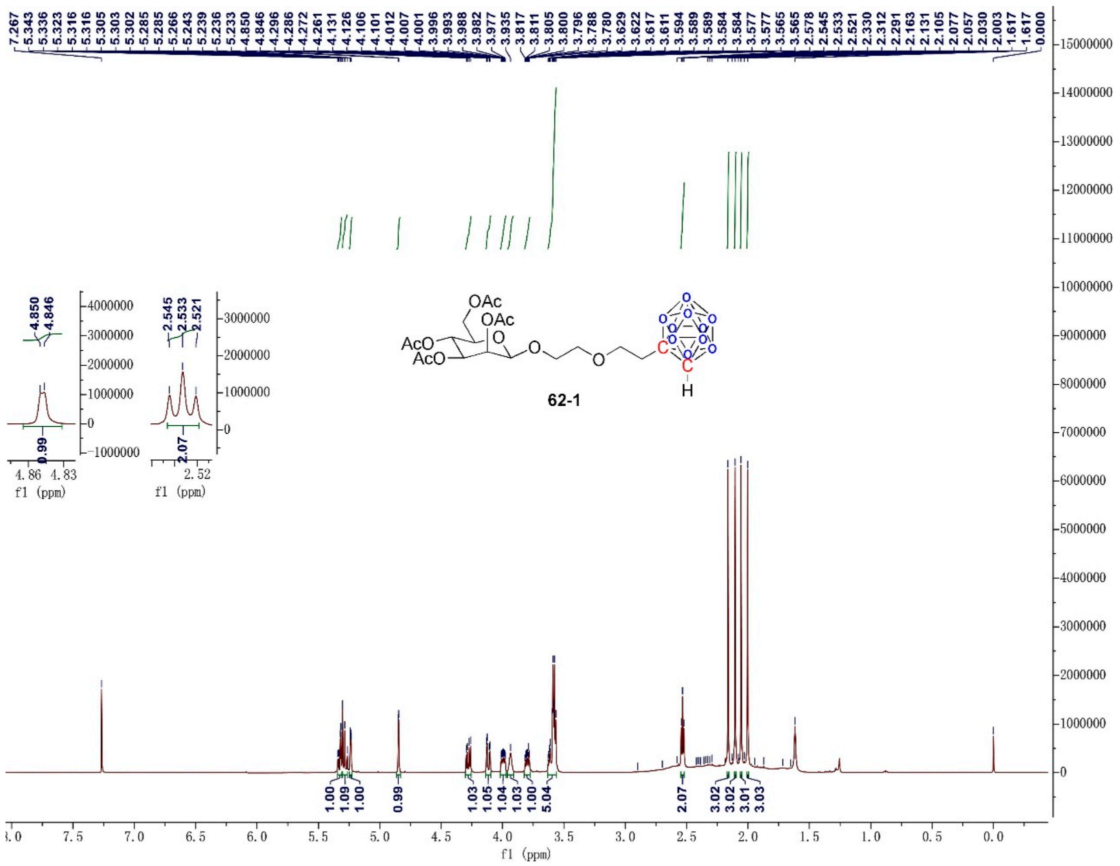

Figure S160 ^{1}H NMR spectrum of **62-1**

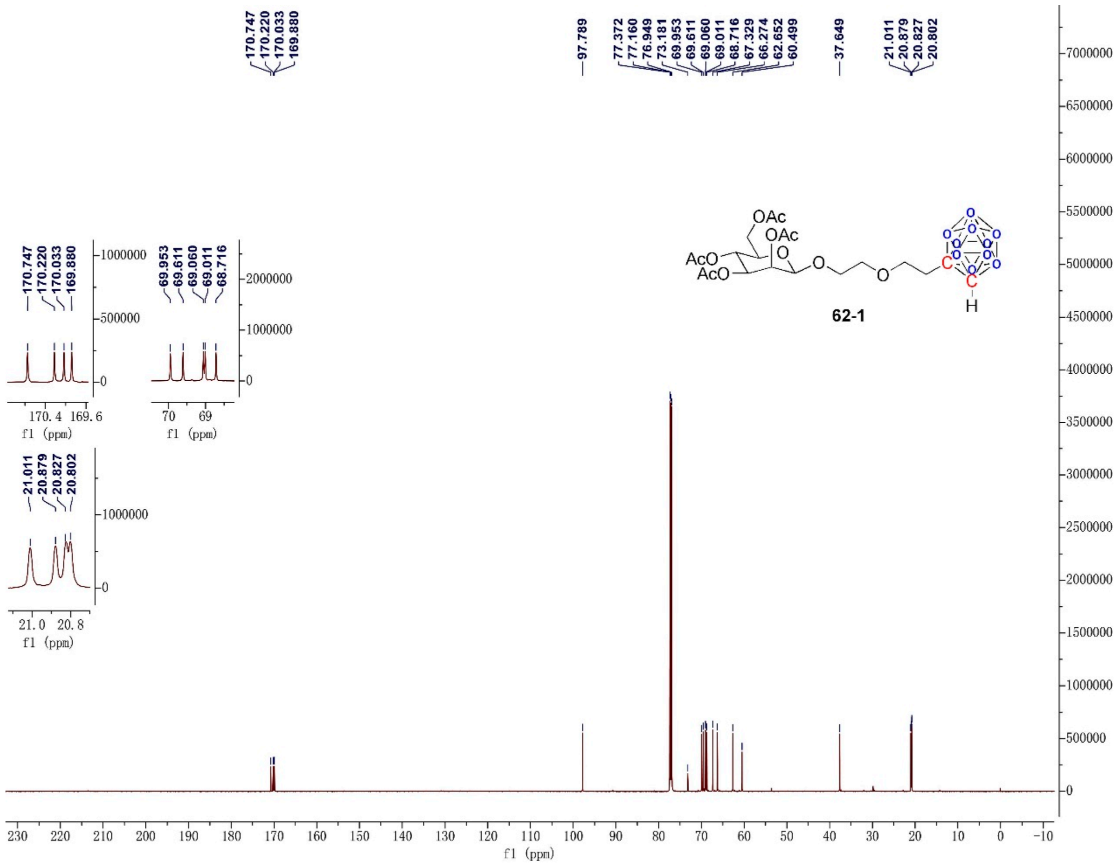

Figure S161 ^{13}C NMR spectrum of **62-1**

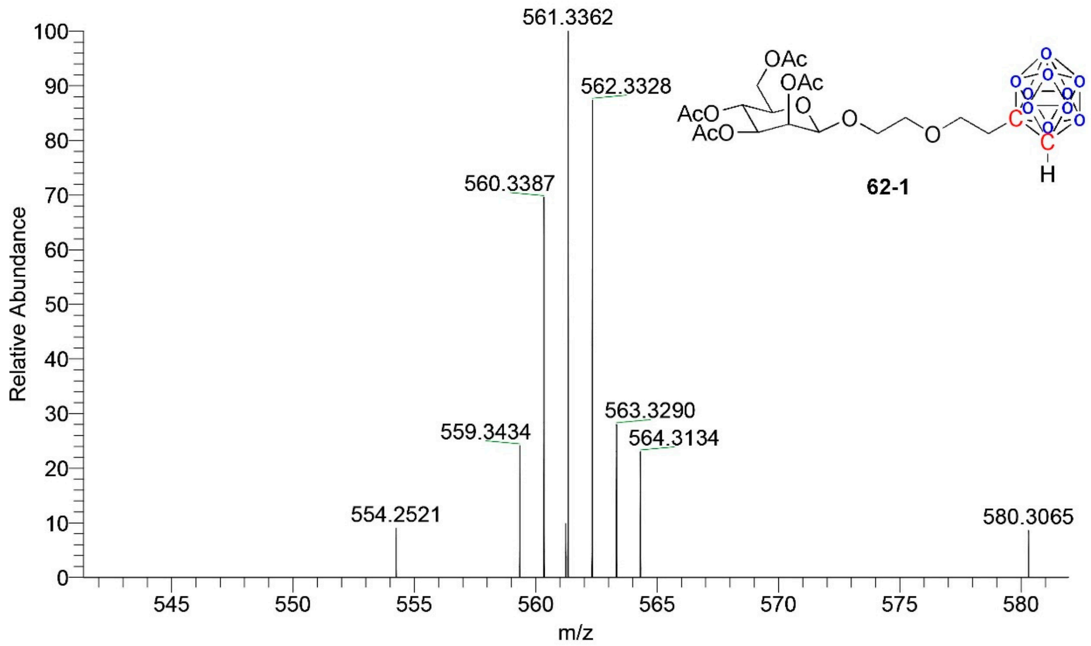

Figure S162 HR-MS (ESI/ion trap) spectrum of **62-1**

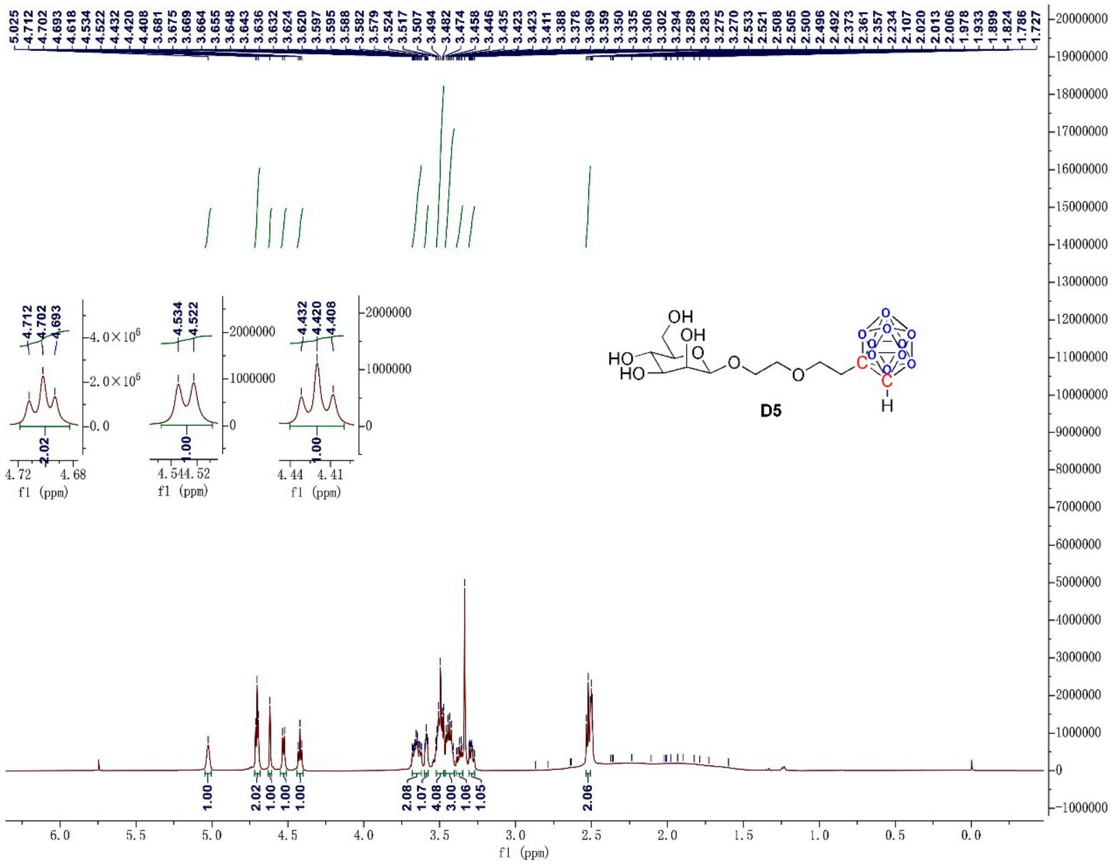

Figure S163 ^{1}H NMR spectrum of **D5**

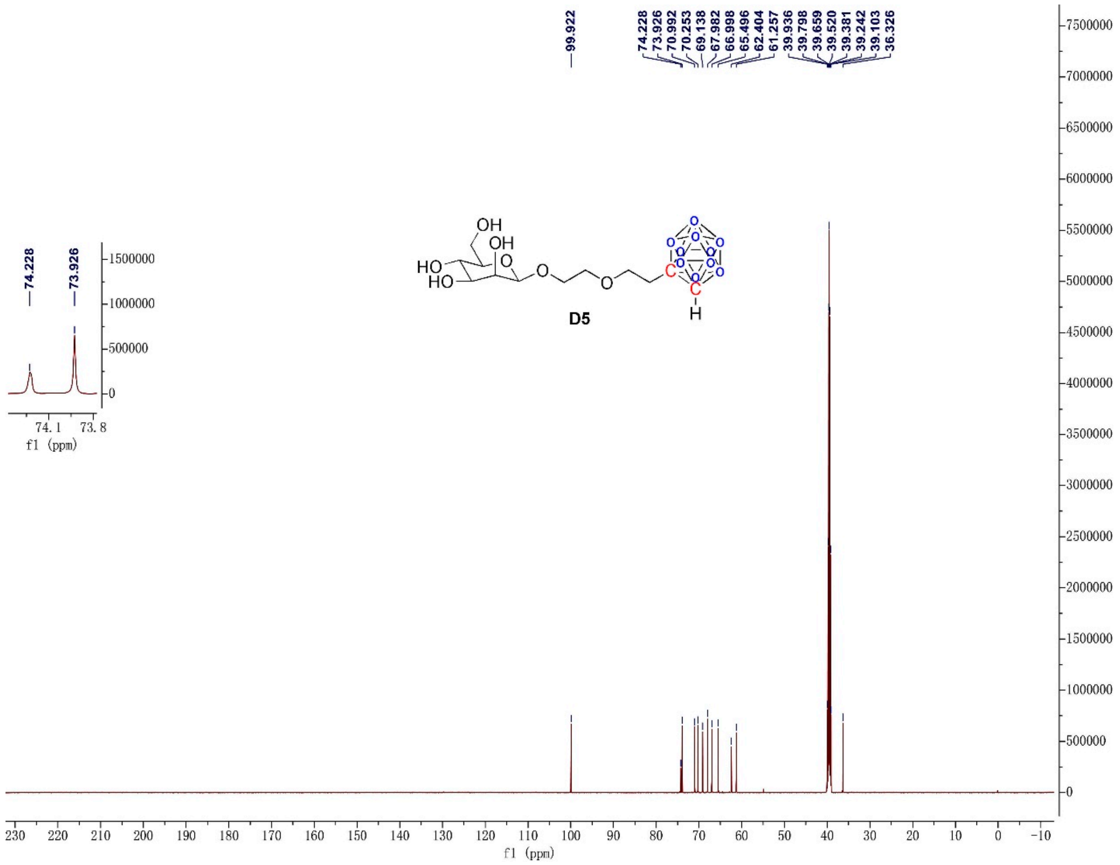

Figure S164 ^{13}C NMR spectrum of **D5**

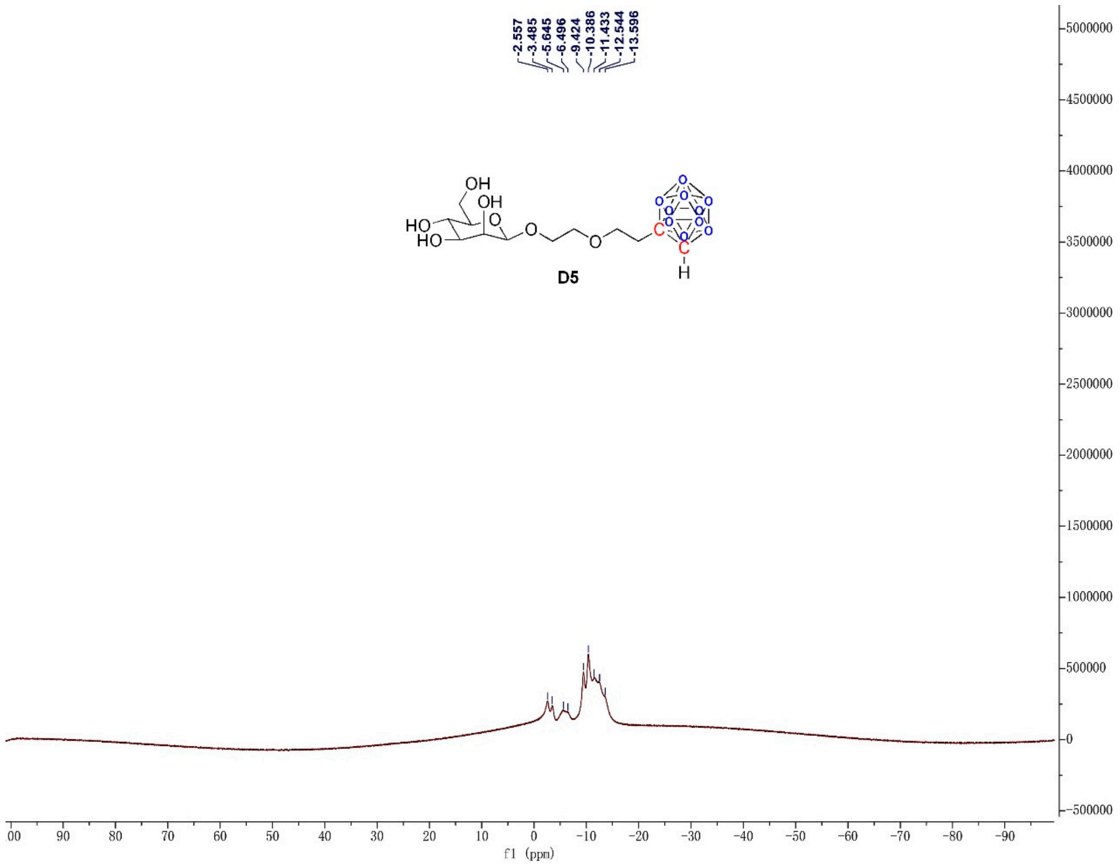

Figure S165 ^{11}B NMR spectrum of **D5**

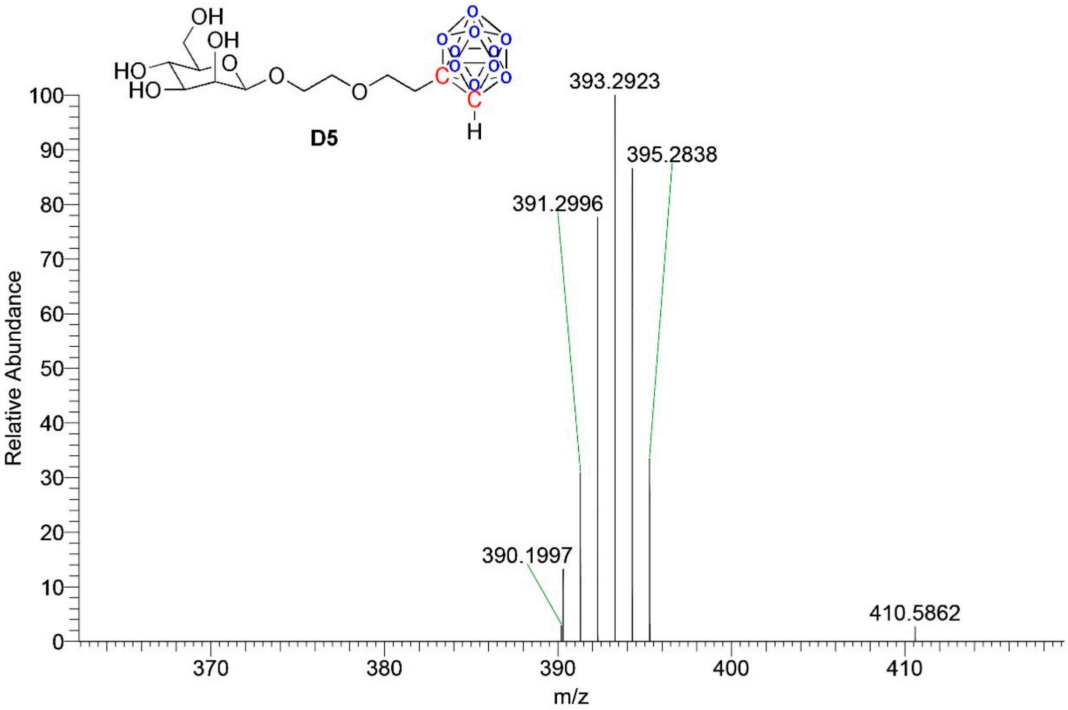

Figure S166 HR-MS (ESI/ion trap) spectrum of **D5**

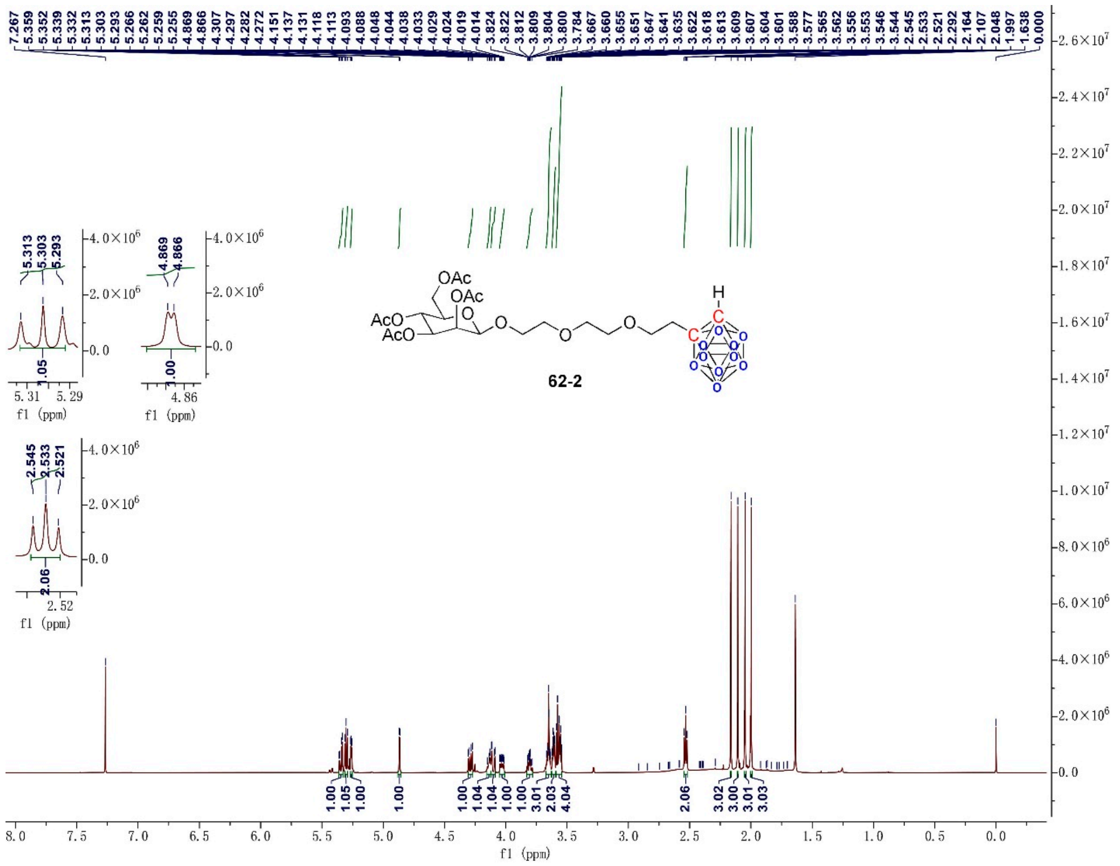

Figure S167 ^{1}H NMR spectrum of **62-2**

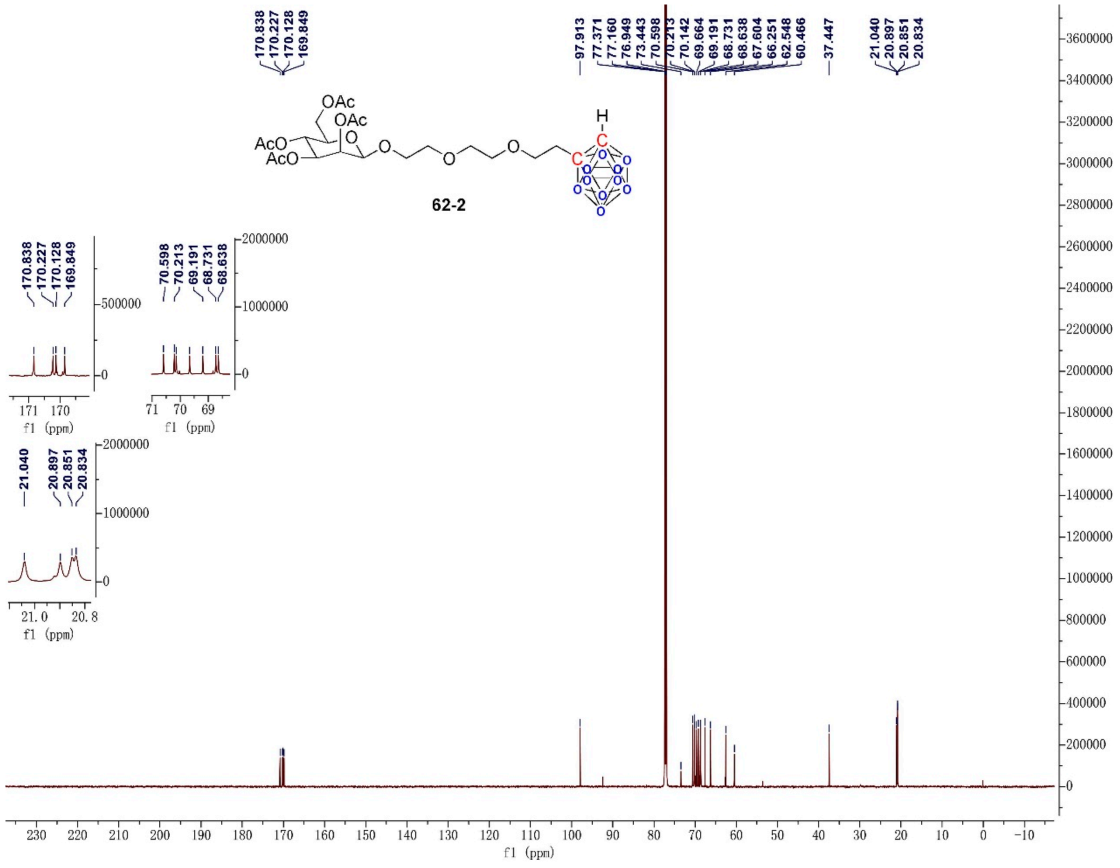

Figure S168 ^{13}C NMR spectrum of **62-2**

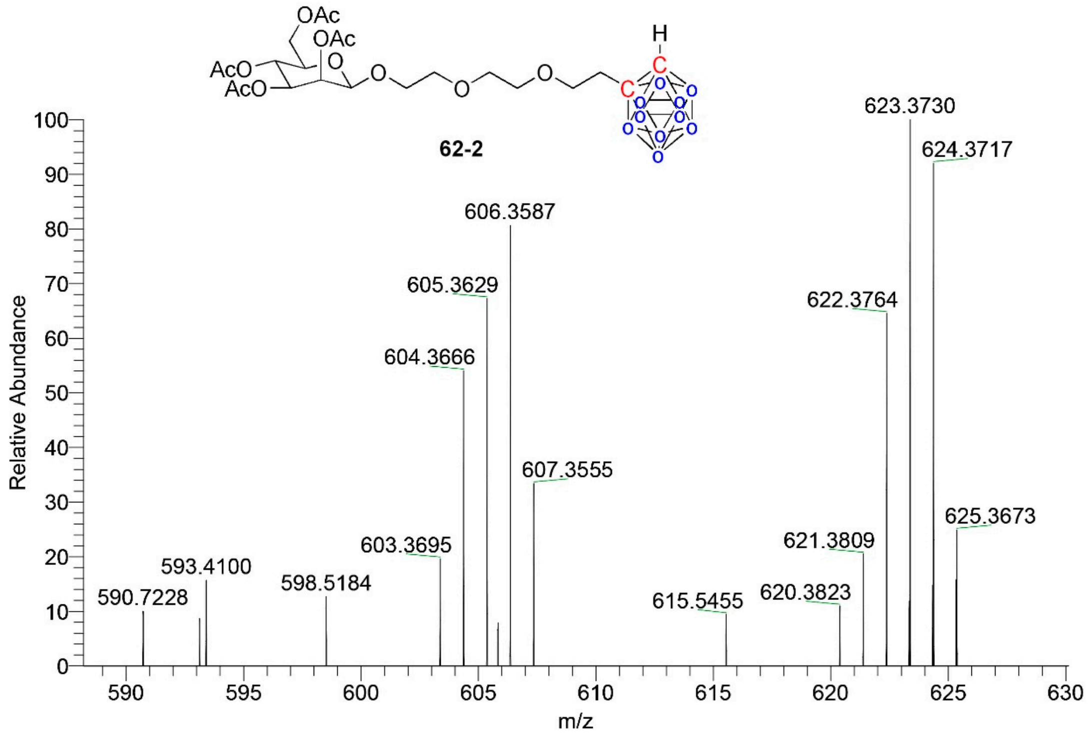

Figure S169 HR-MS (ESI/ion trap) spectrum of **62-2**

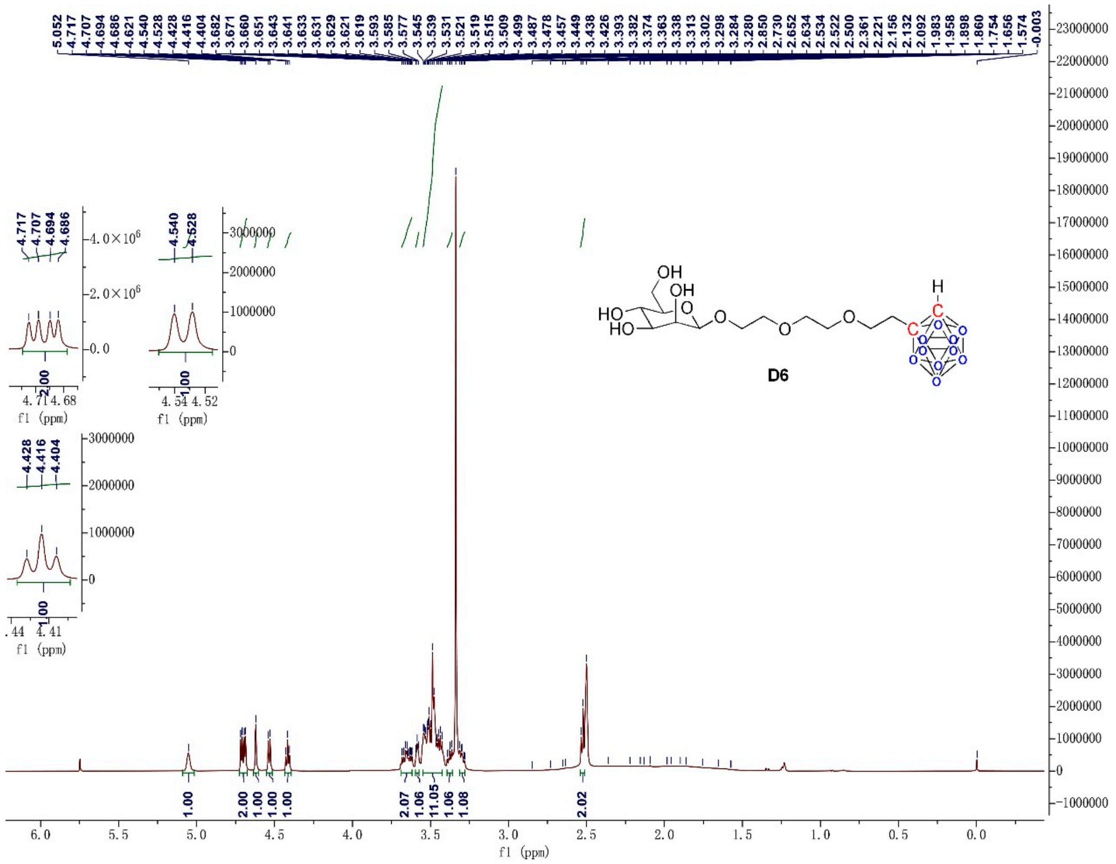

Figure S170 ^{1}H NMR spectrum of **D6**

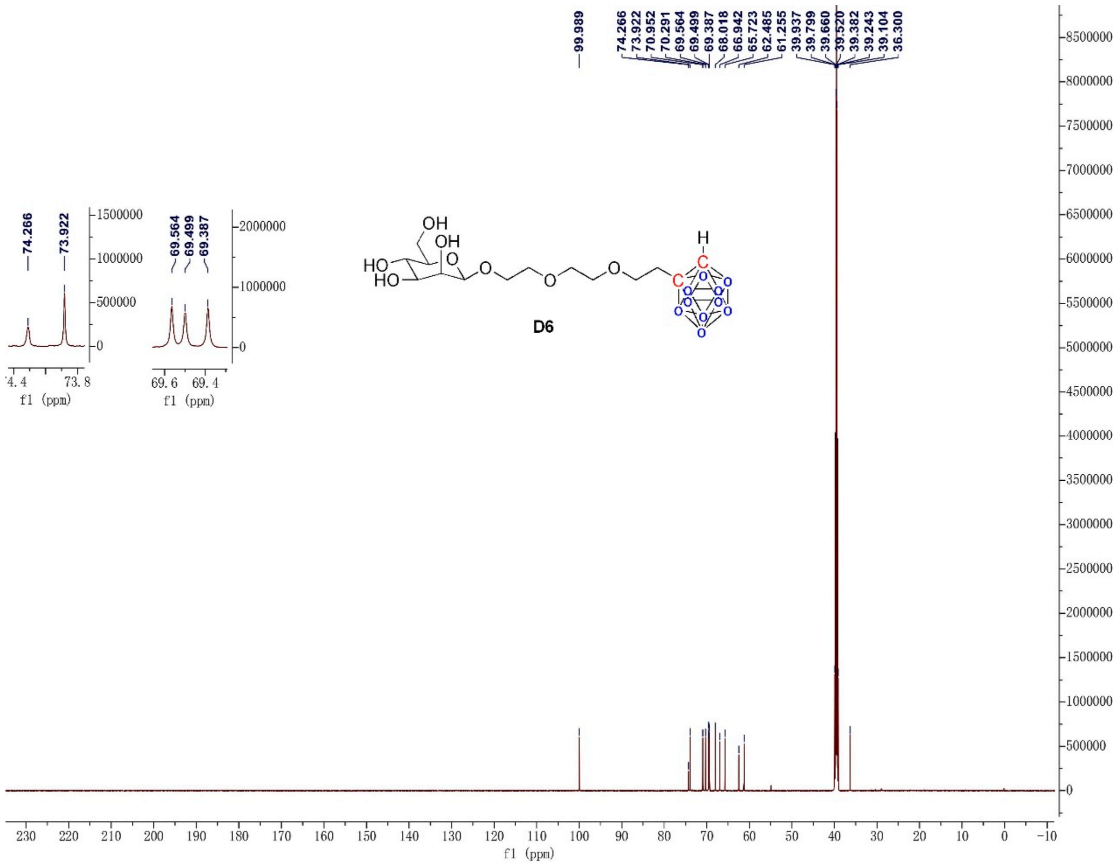

Figure S171 ^{13}C NMR spectrum of **D6**

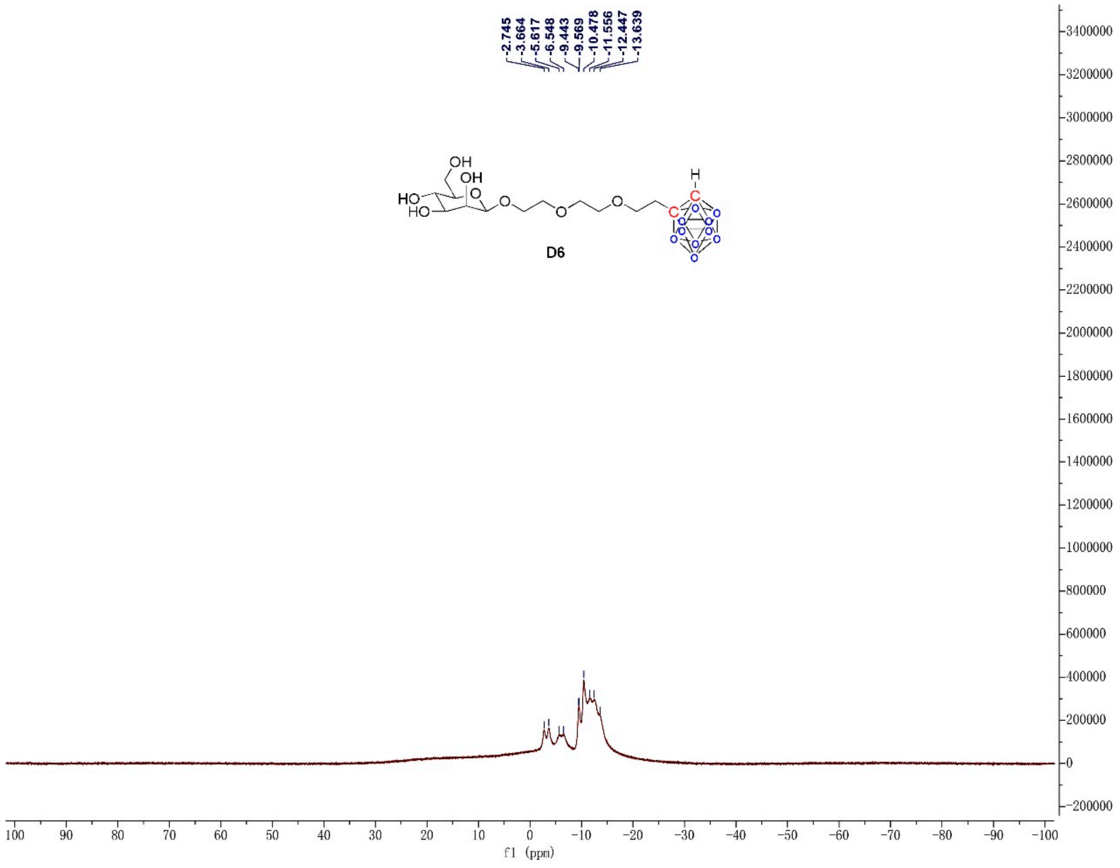

Figure S172 ^{11}B NMR spectrum of **D6**

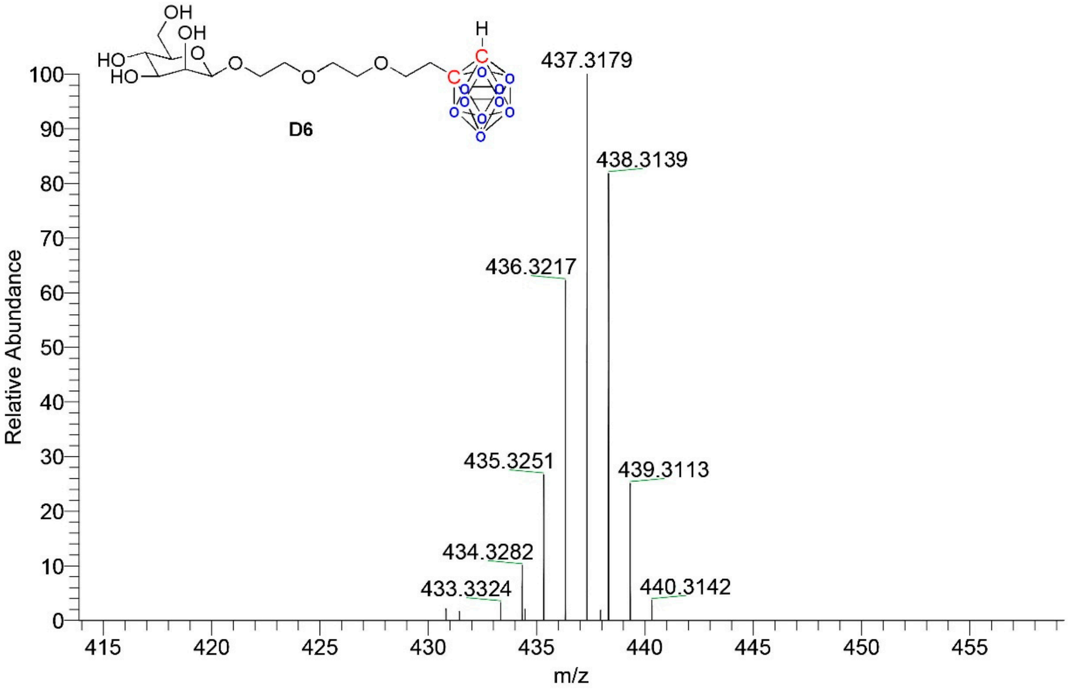

Figure S173 HR-MS (ESI/ion trap) spectrum of **D6**

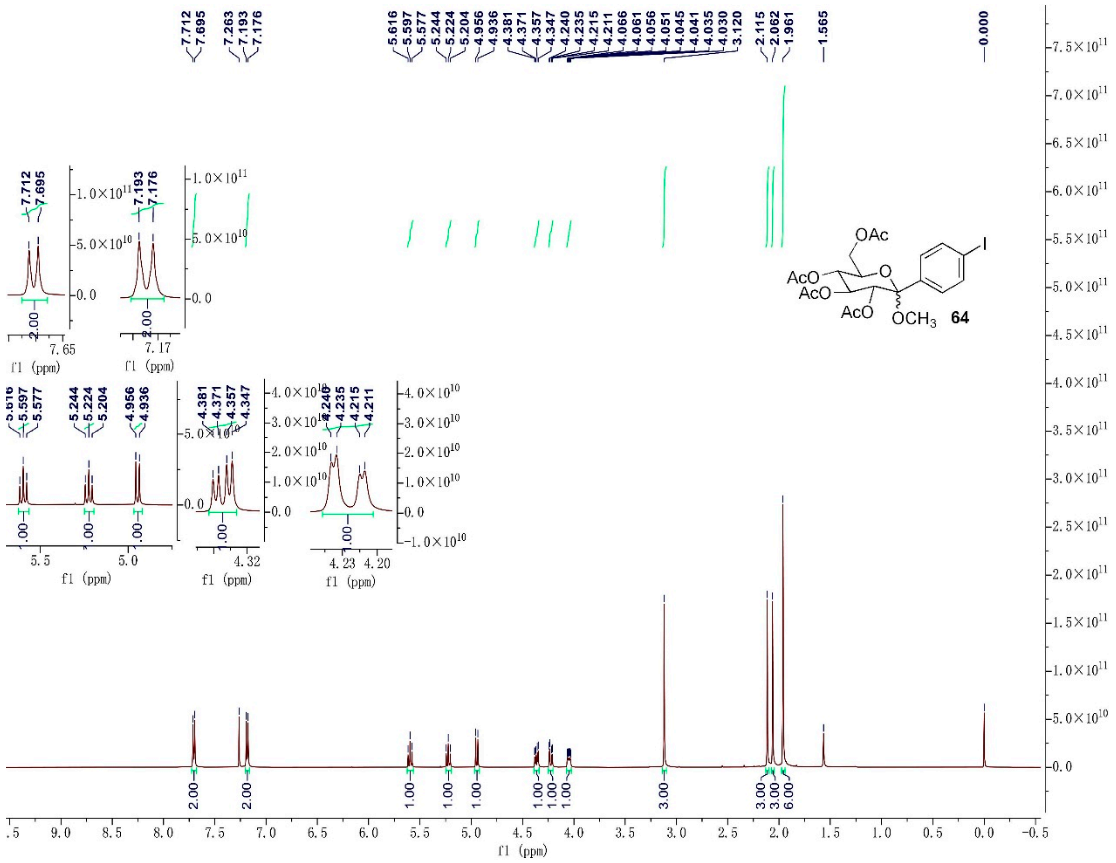

Figure S174 ^{1}H NMR spectrum of **64**

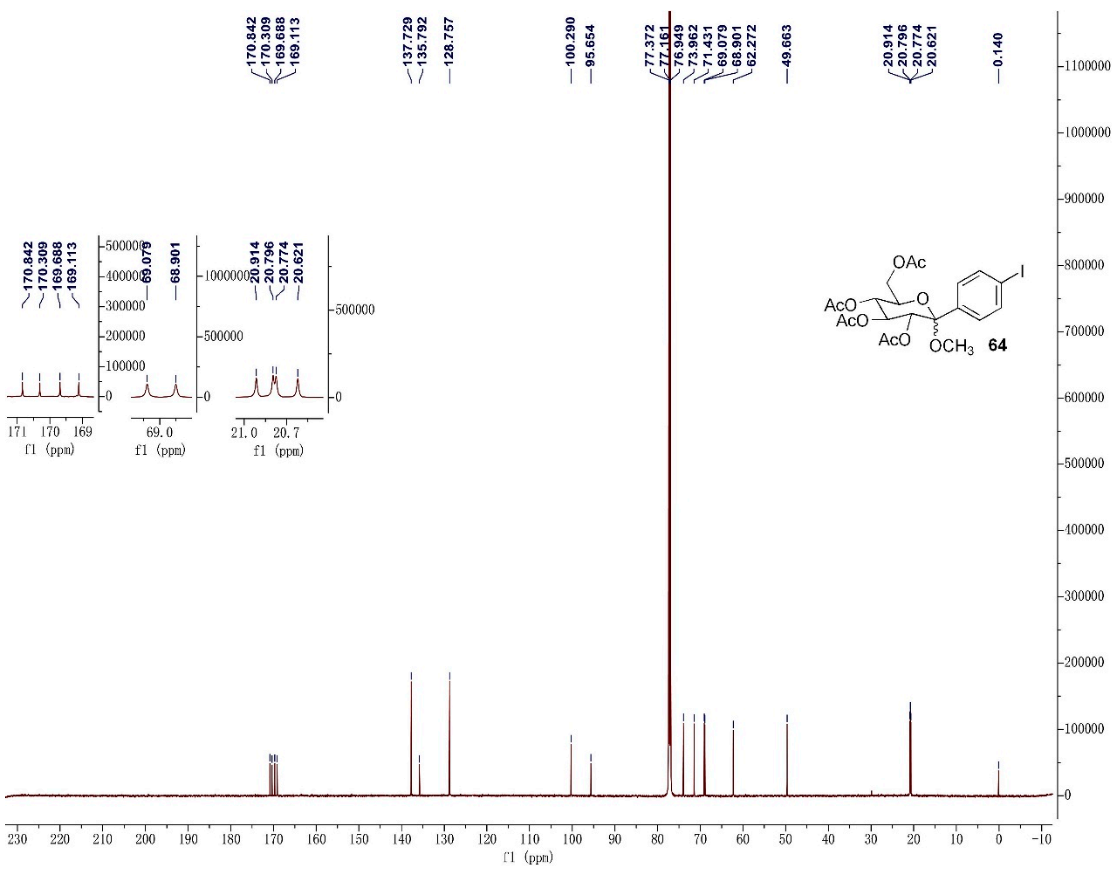

Figure S175 ^{13}C NMR spectrum of **64**

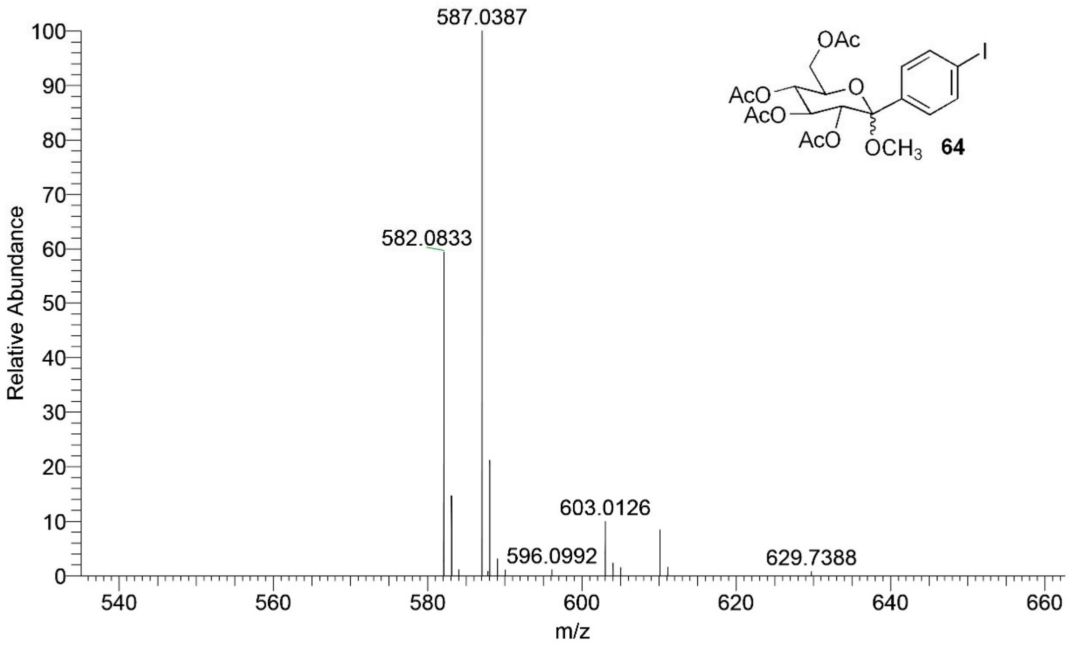

Figure S176 HR-MS (ESI/ion trap) spectrum of **64**

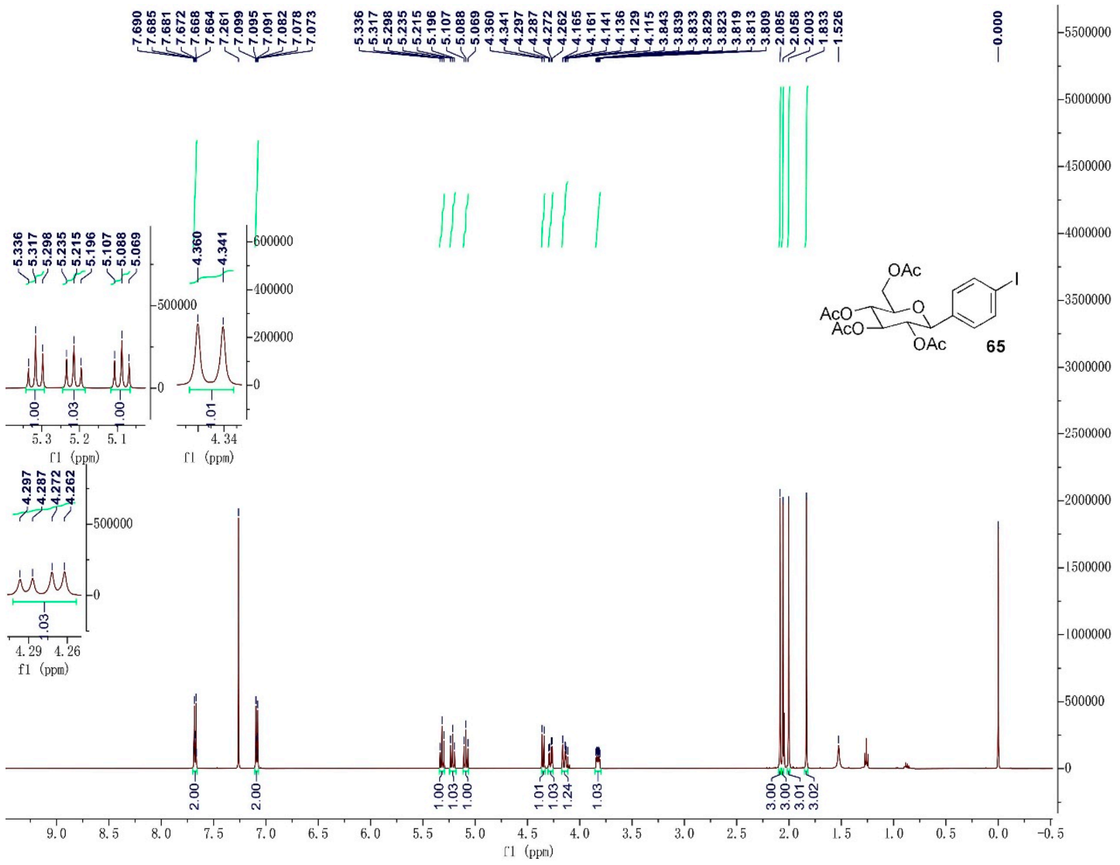

Figure S177 ^{1}H NMR spectrum of **65**

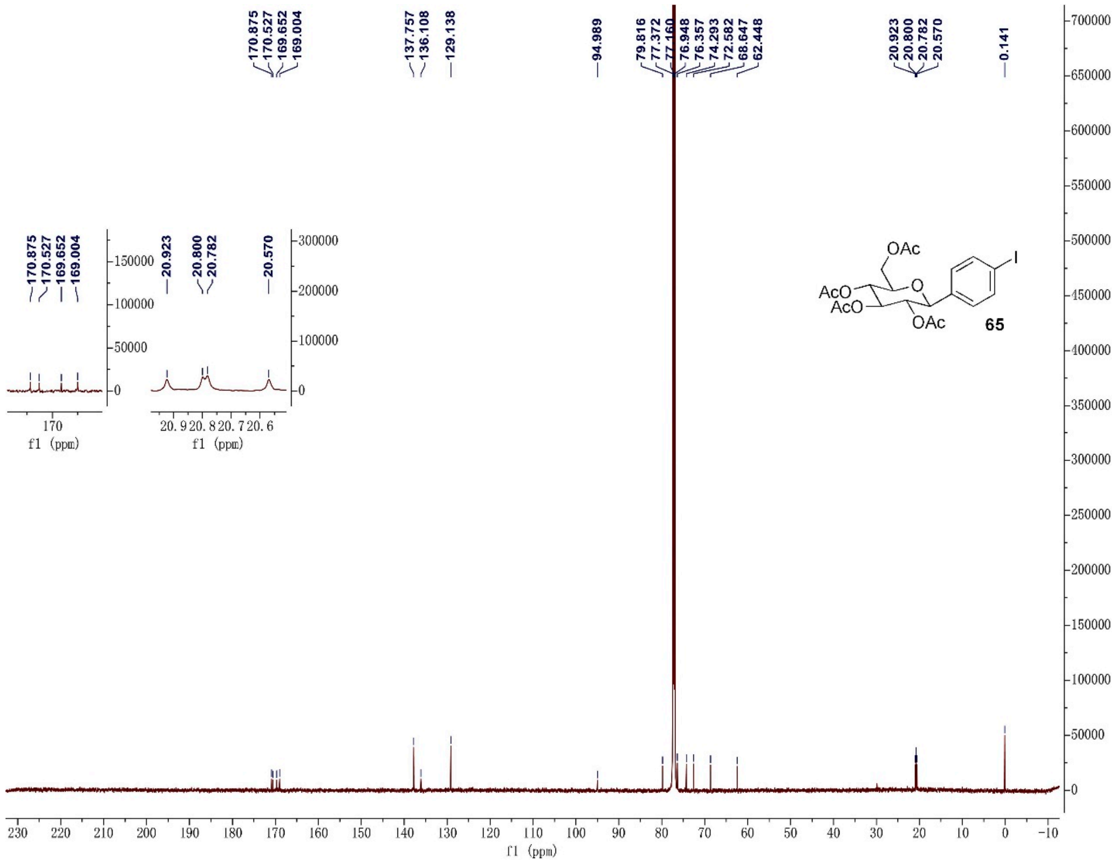

Figure S178 ^{13}C NMR spectrum of **65**

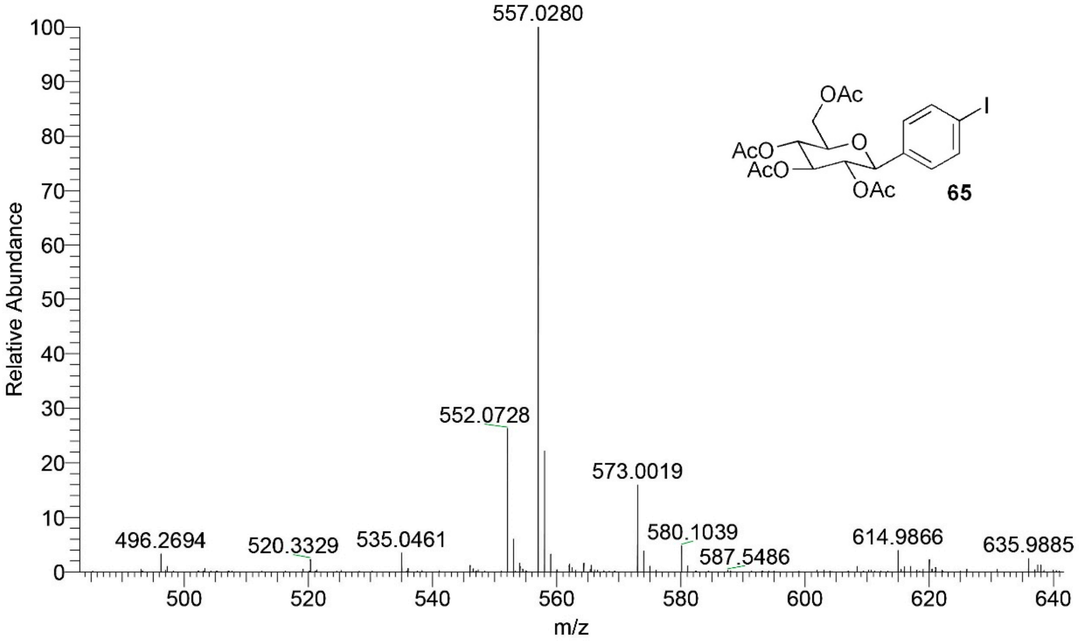

Figure S179 HR-MS (ESI/ion trap) spectrum of **65**

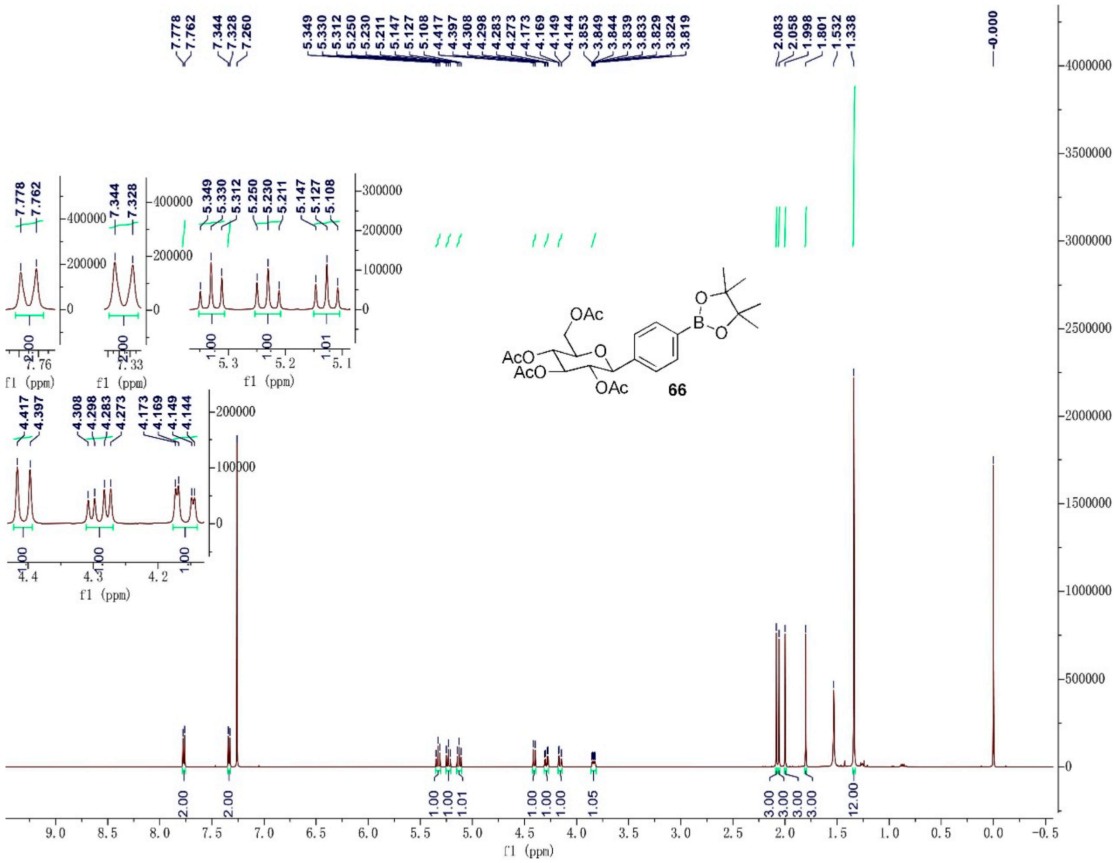

Figure S180 ^{1}H NMR spectrum of **66**

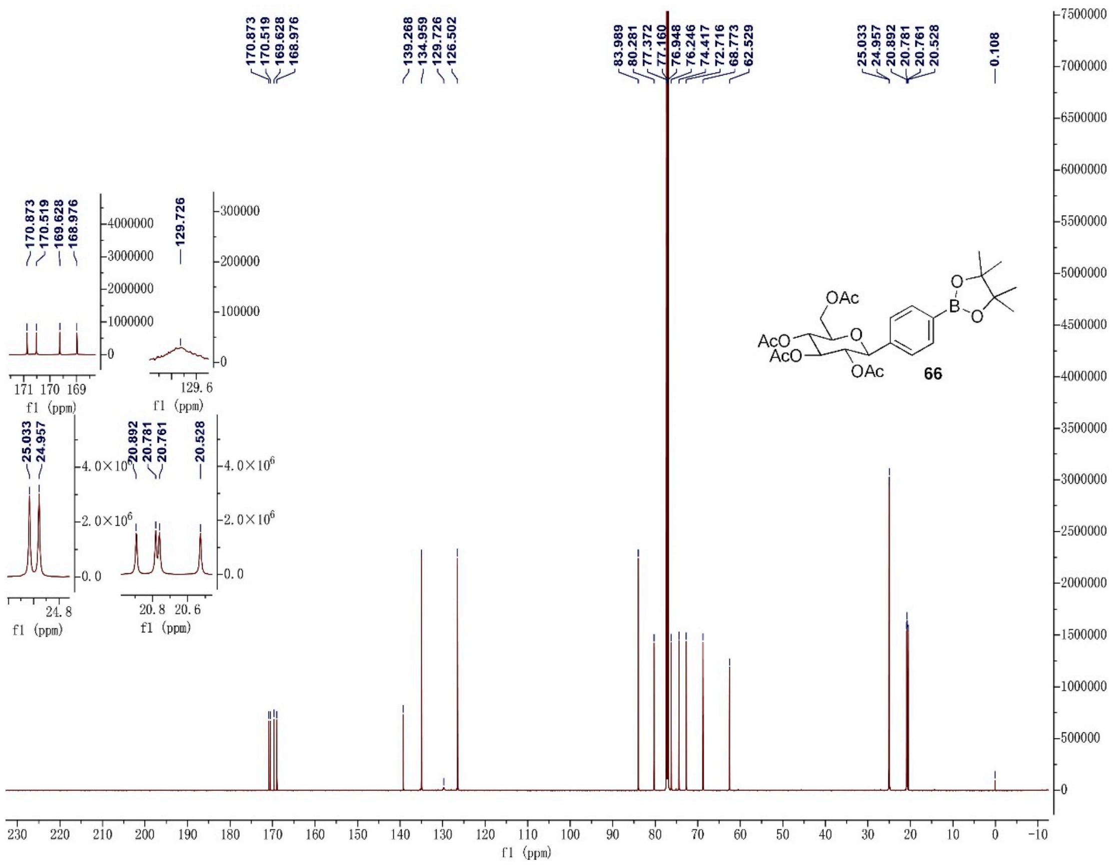

Figure S181 ^{13}C NMR spectrum of **66**

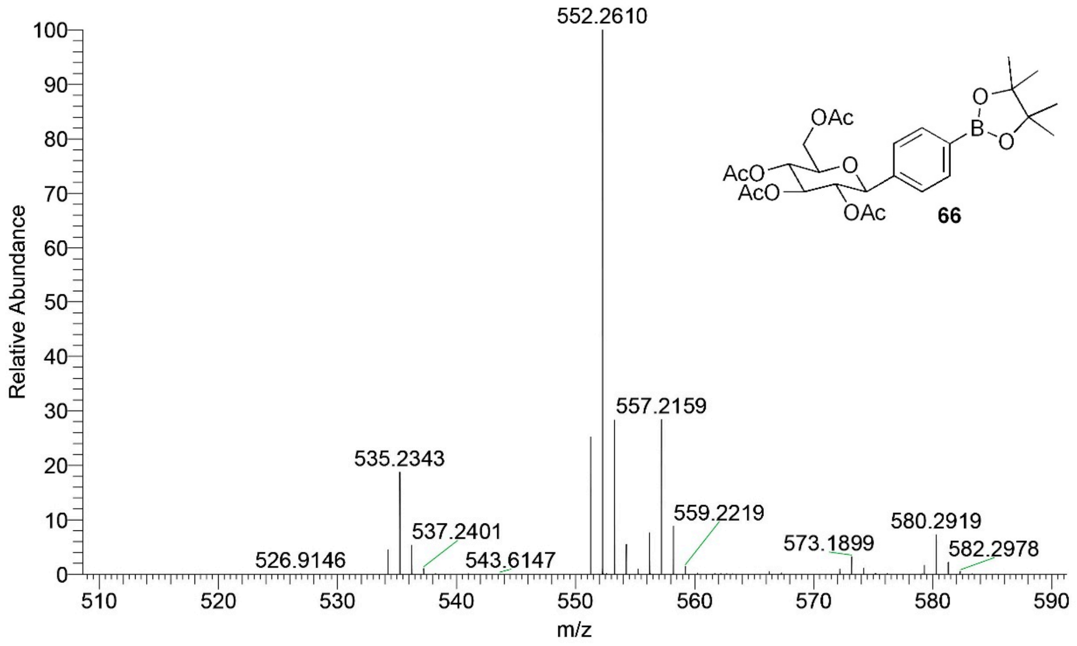

Figure S182 HR-MS (ESI/ion trap) spectrum of **66**

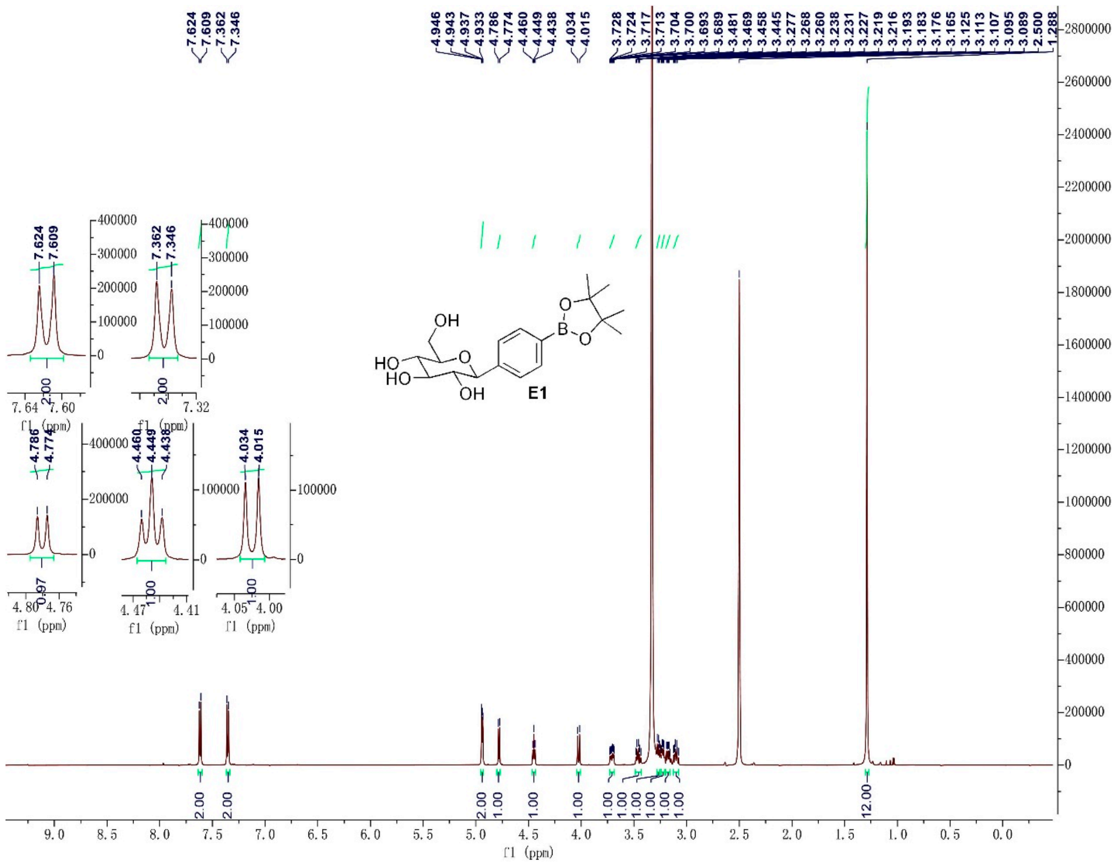

Figure S183 ^{1}H NMR spectrum of **E1**

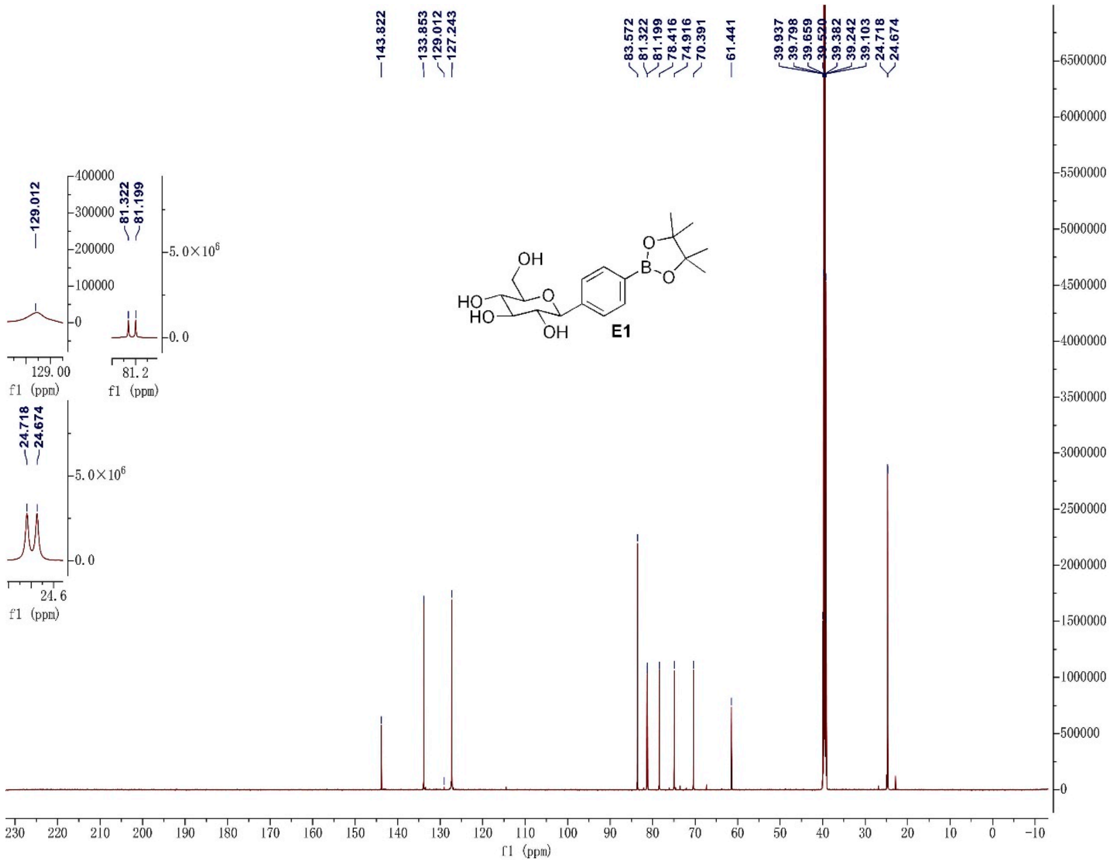

Figure S184 ^{13}C NMR spectrum of **E1**

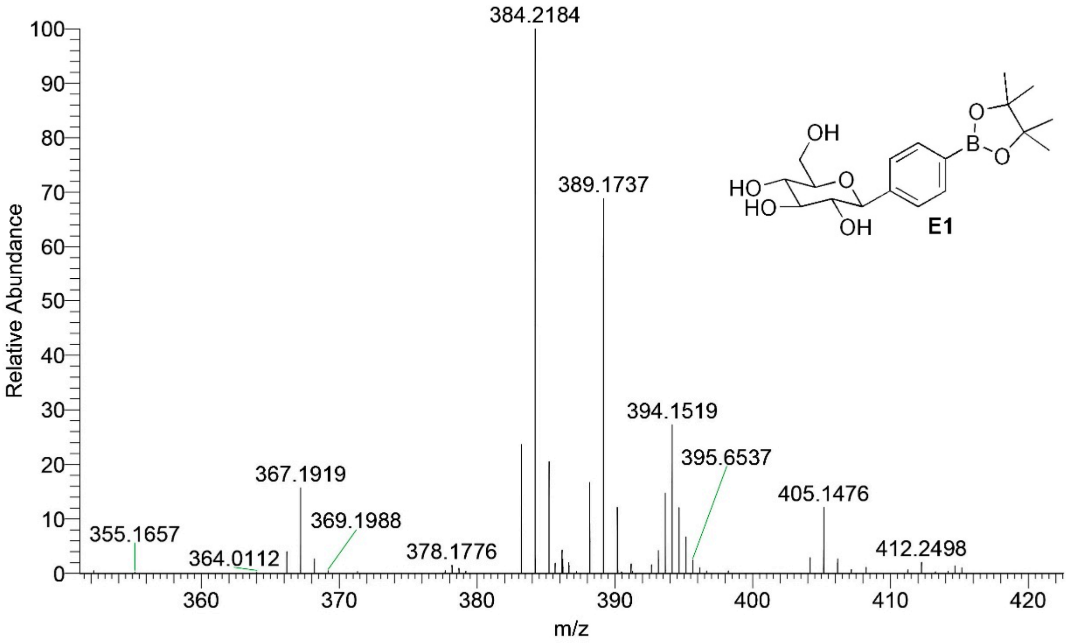

Figure S185 HR-MS (ESI/ion trap) spectrum of **E1**

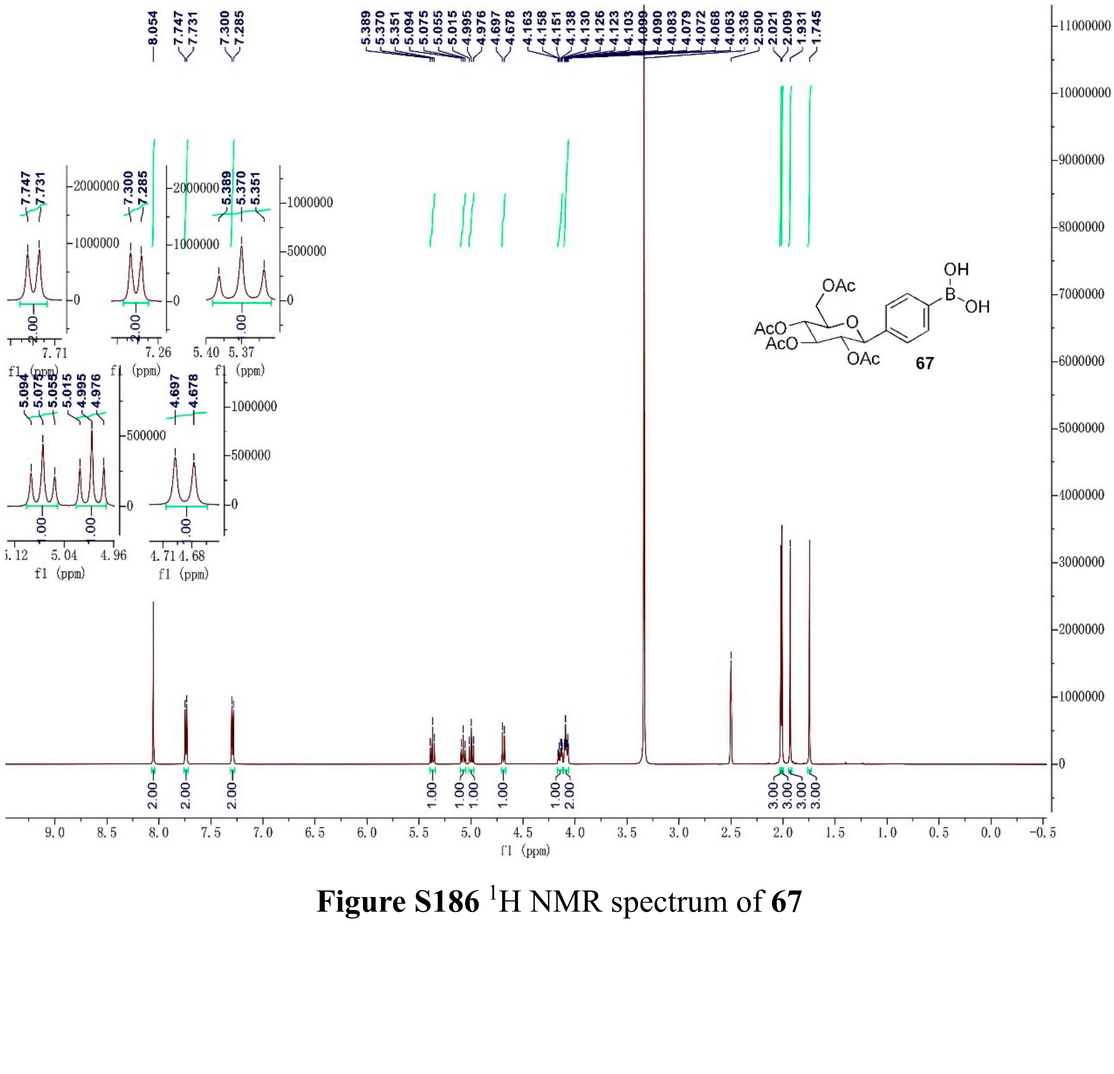

Figure S186 ^{1}H NMR spectrum of **67**

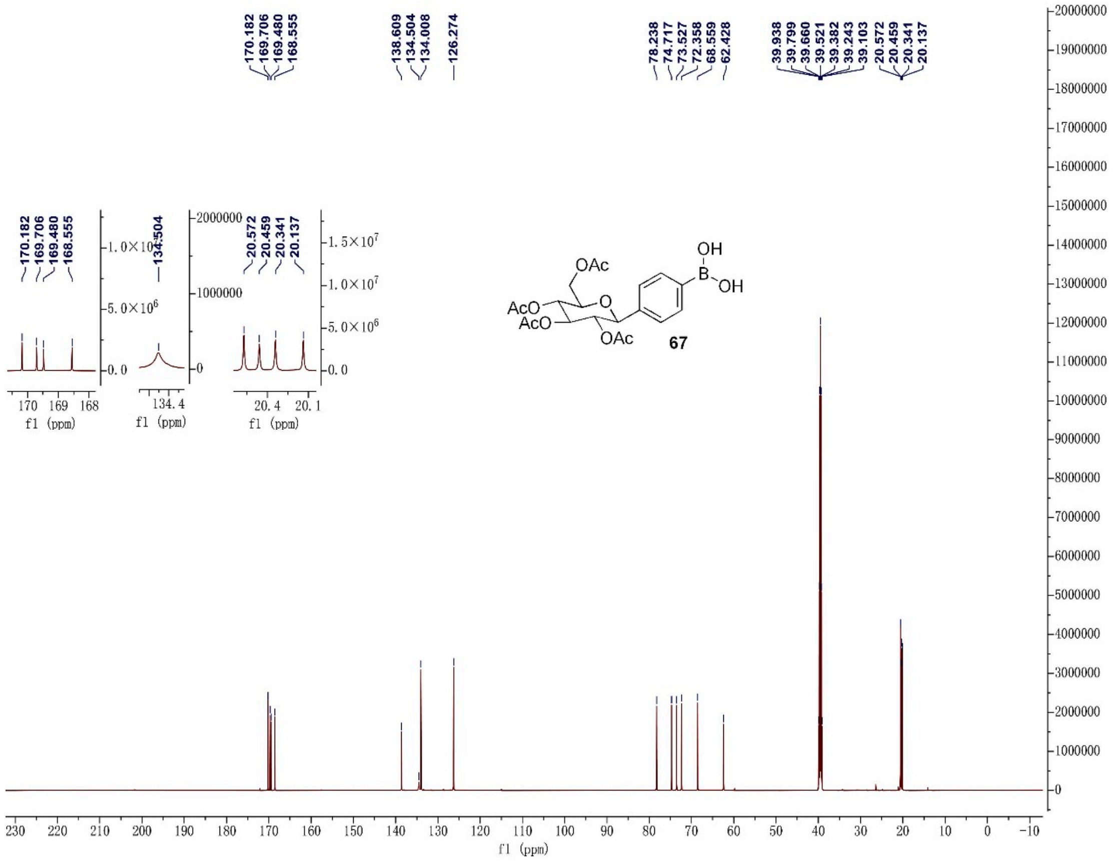

Figure S187 ^{13}C NMR spectrum of **67**

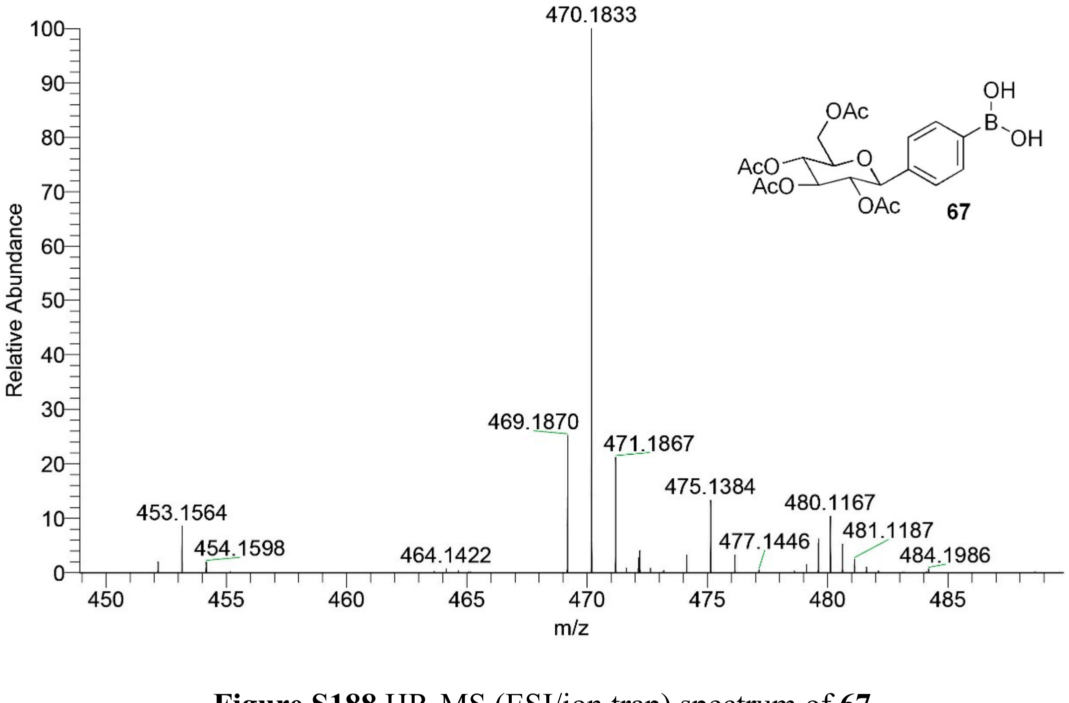

Figure S188 HR-MS (ESI/ion trap) spectrum of **67**

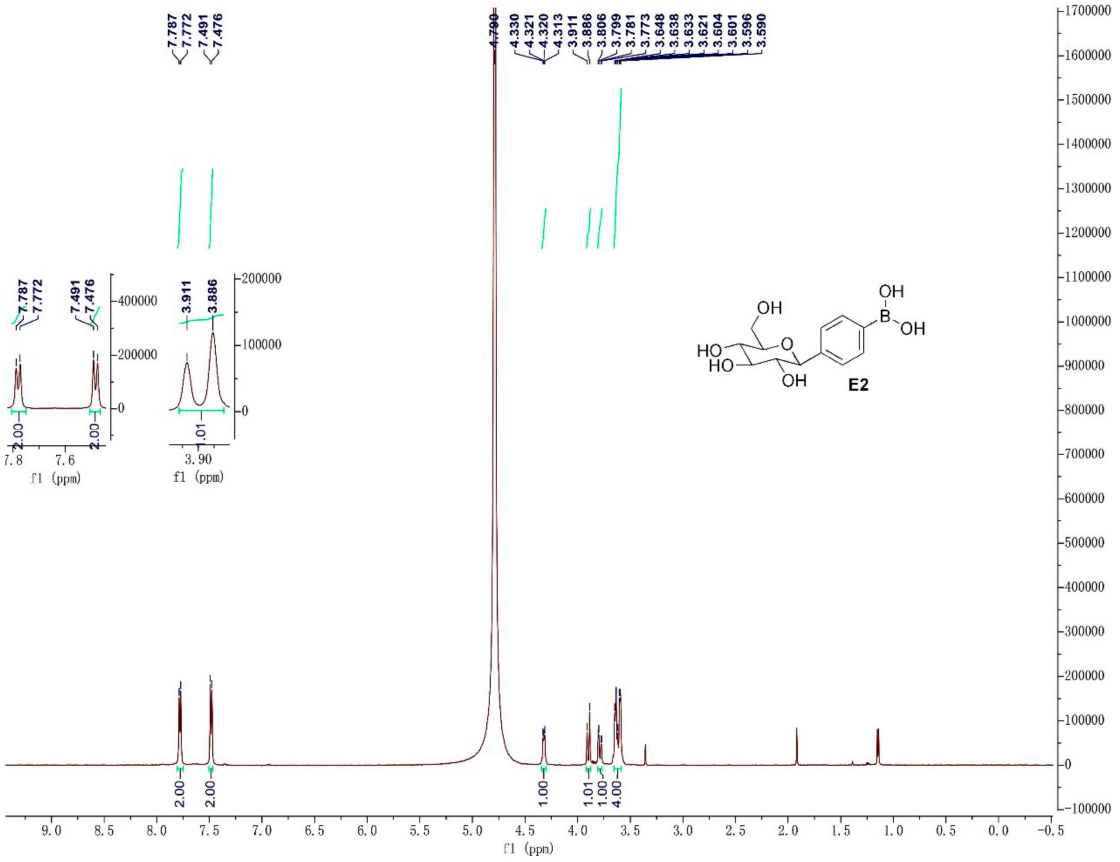

Figure S189 ^{1}H NMR spectrum of **E2**

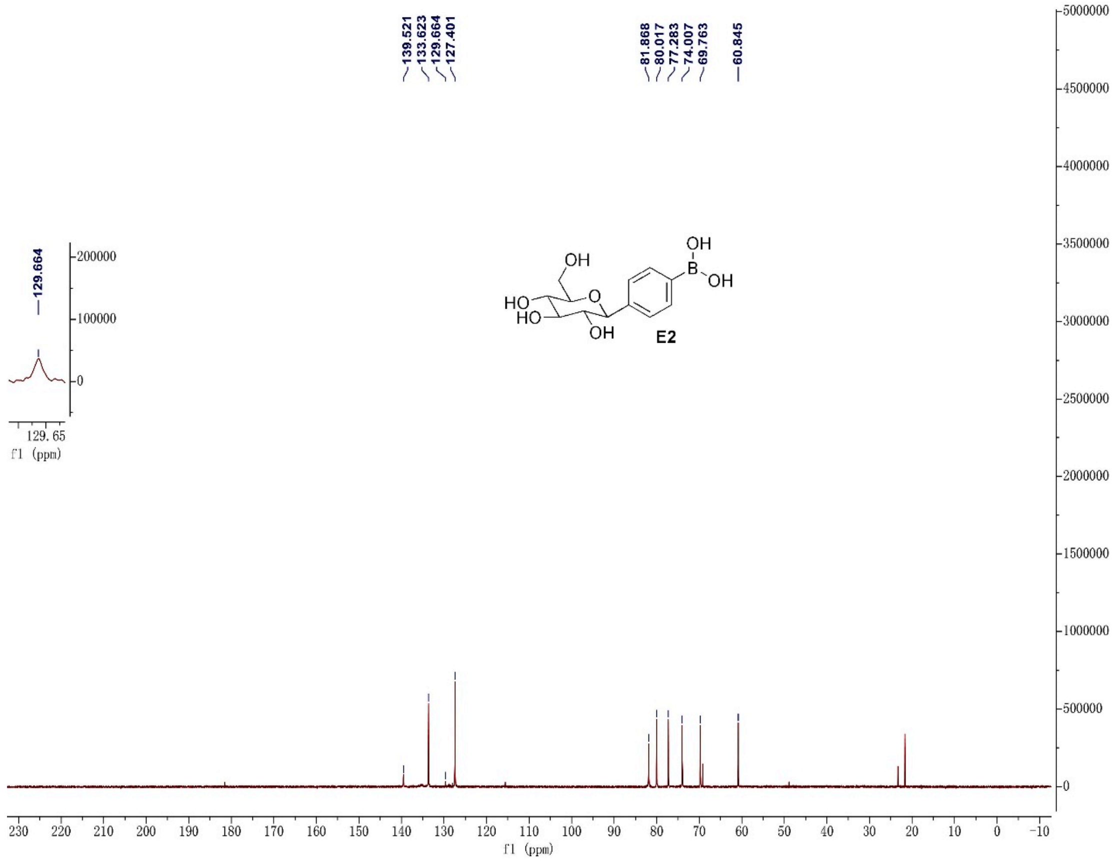

Figure S190 ^{13}C NMR spectrum of **E2**

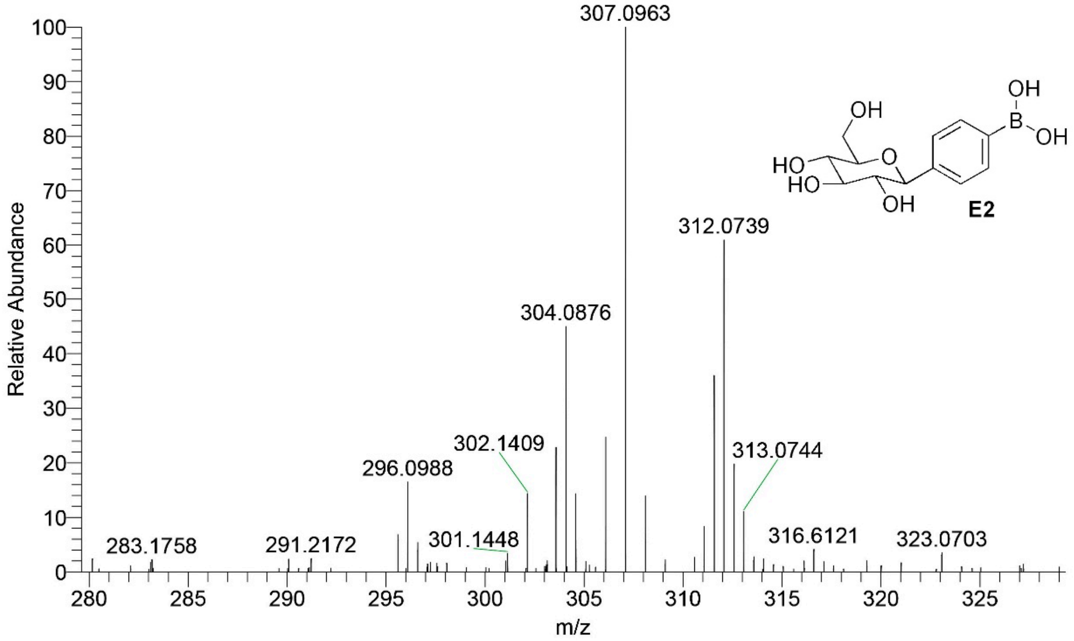

Figure S191 HR-MS (ESI/ion trap) spectrum of **E2**

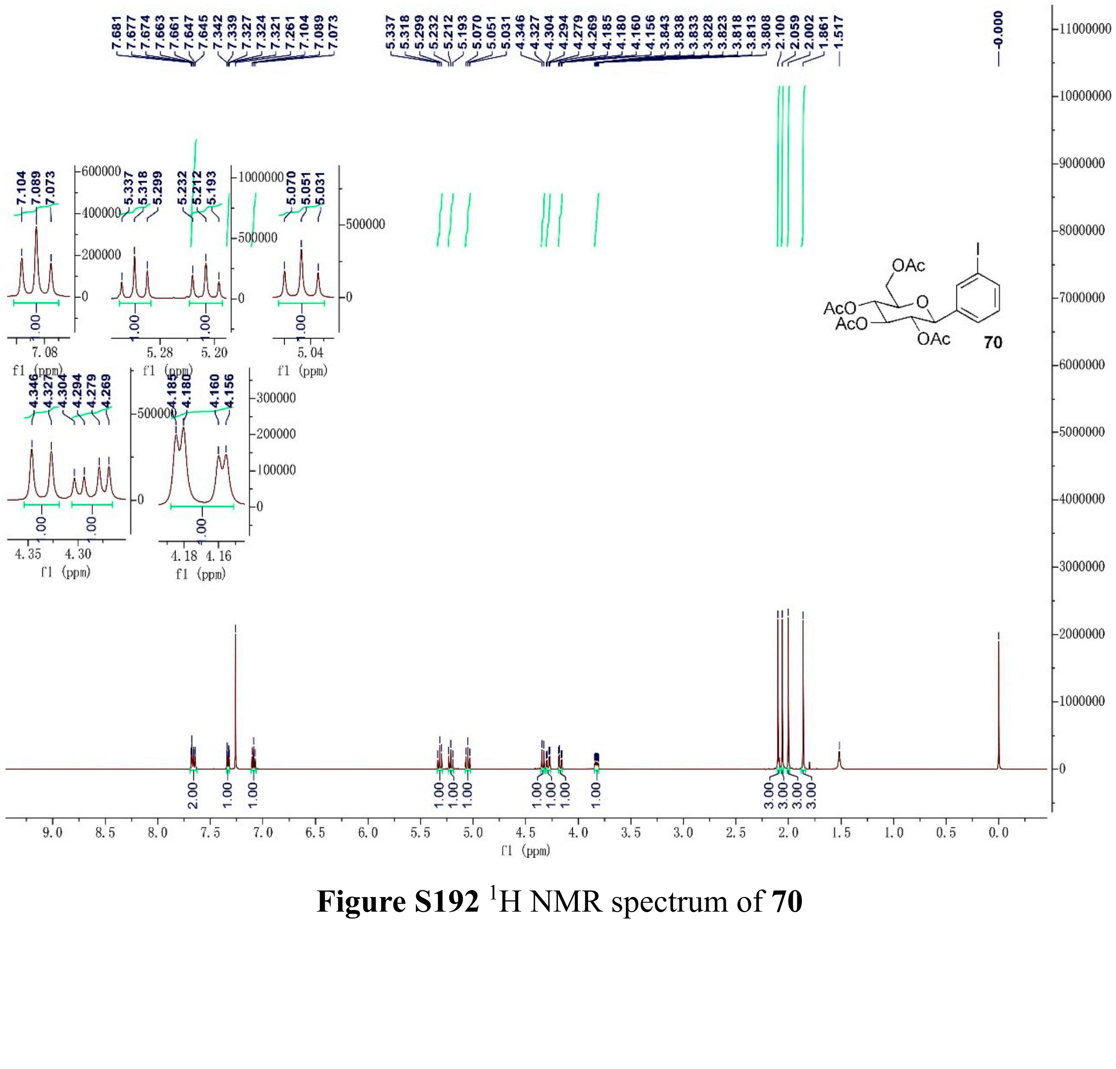

Figure S192 ^{1}H NMR spectrum of **70**

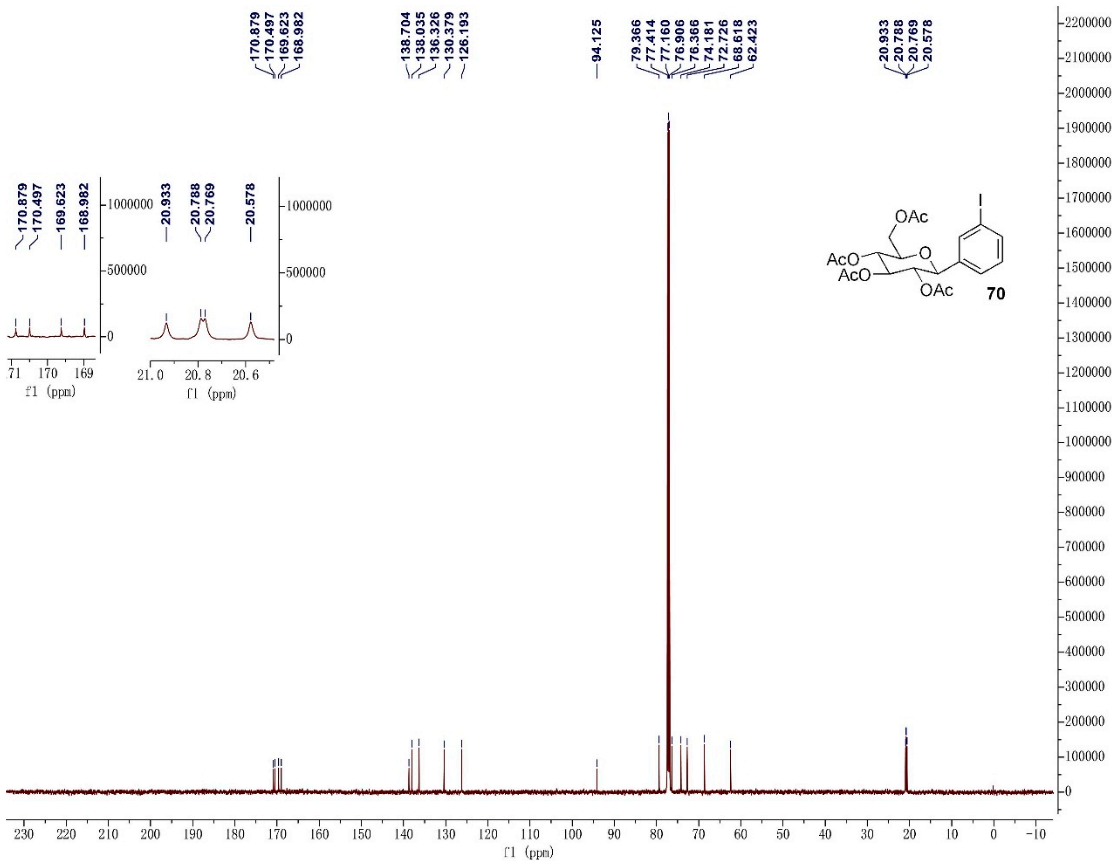

Figure S193 ^{13}C NMR spectrum of **70**

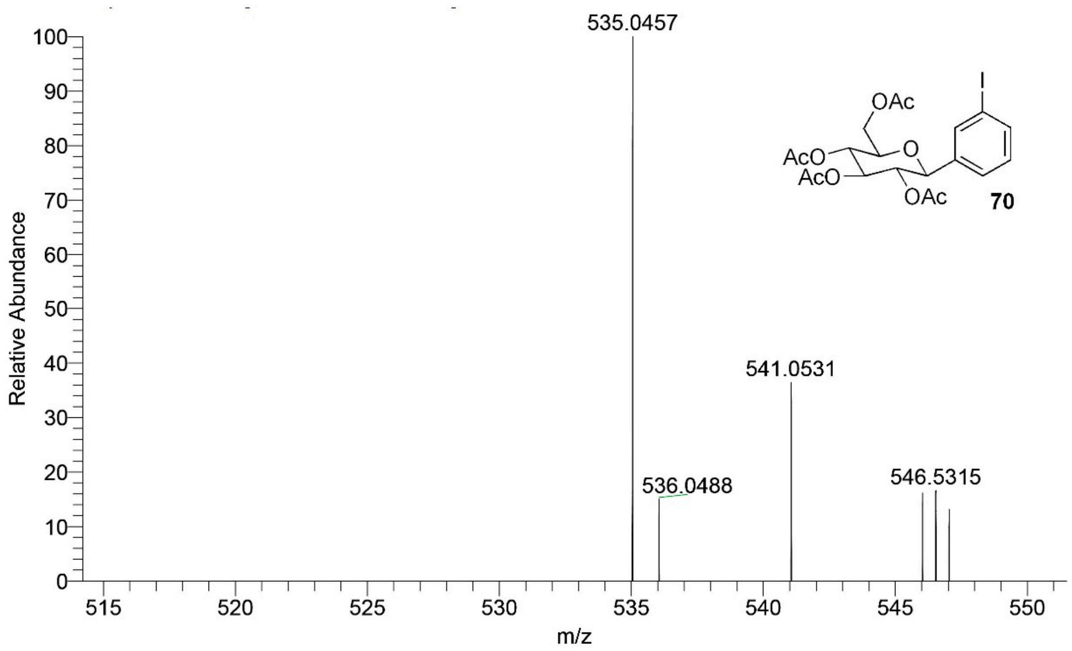

Figure S194 HR-MS (ESI/ion trap) spectrum of **70**

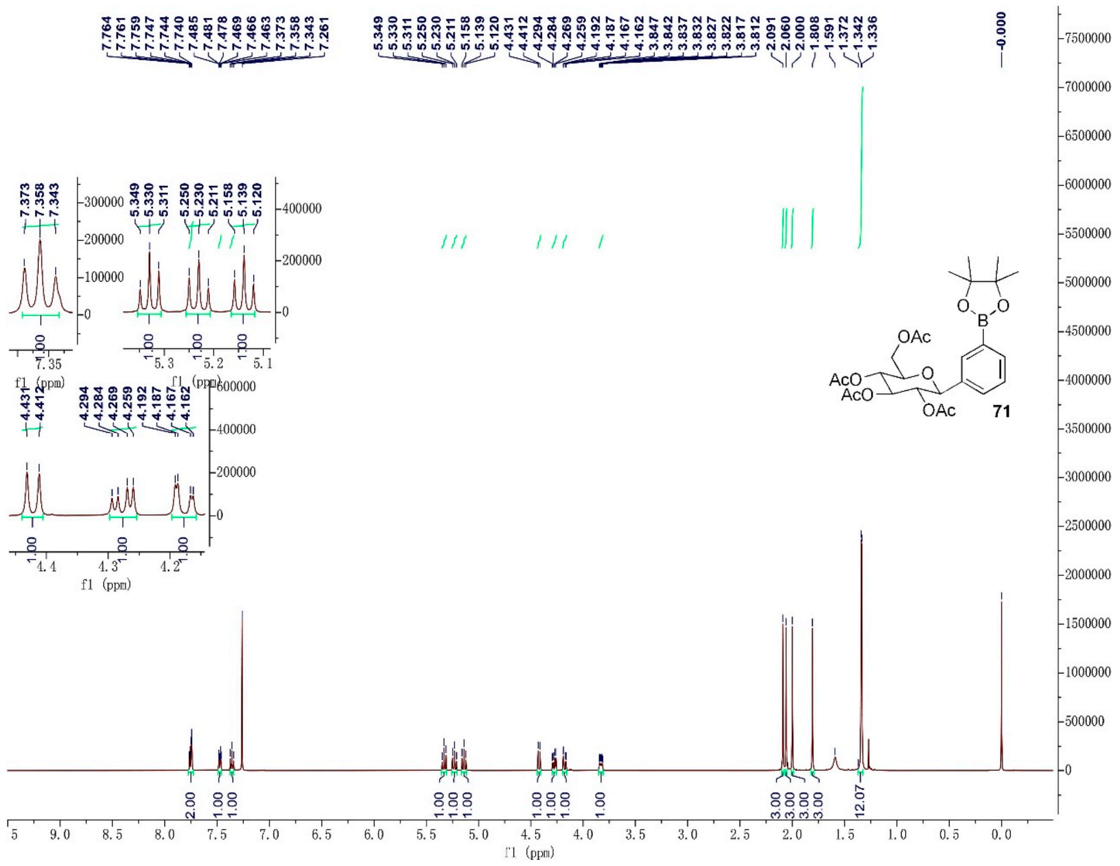

Figure S195 ^{1}H NMR spectrum of **71**

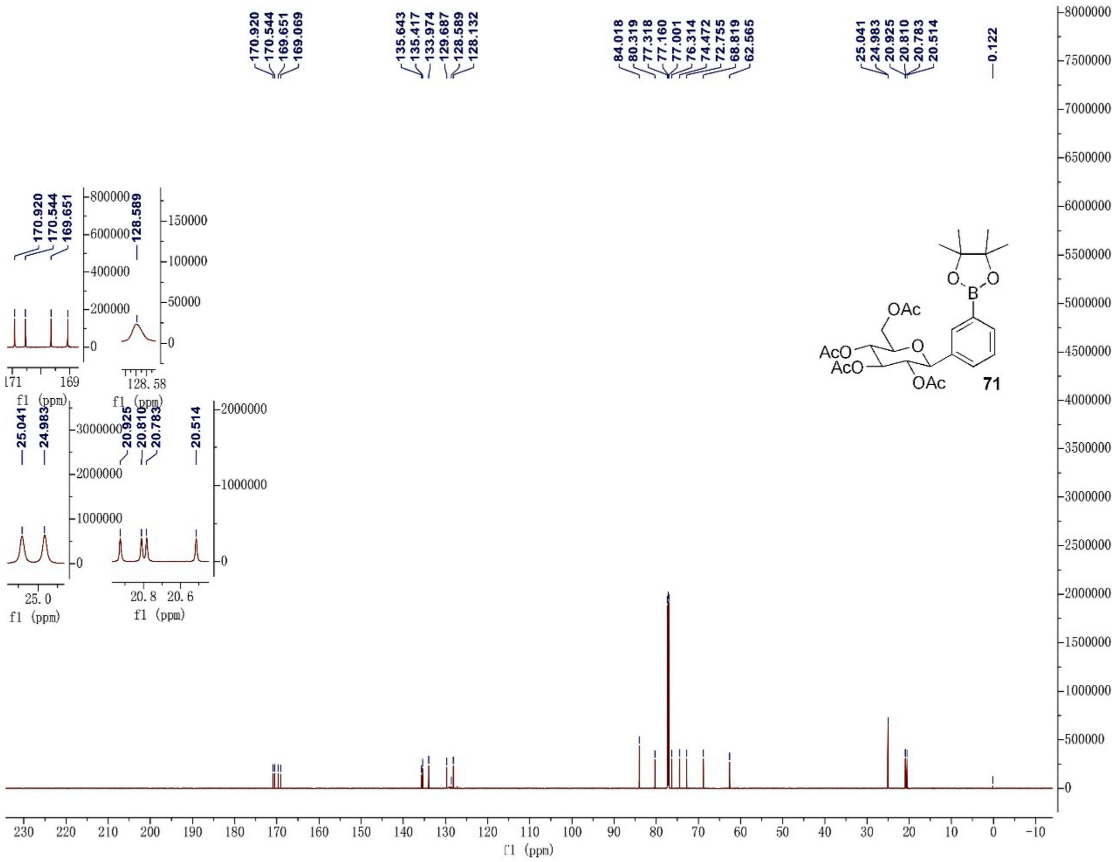

Figure S196 ^{13}C NMR spectrum of **71**

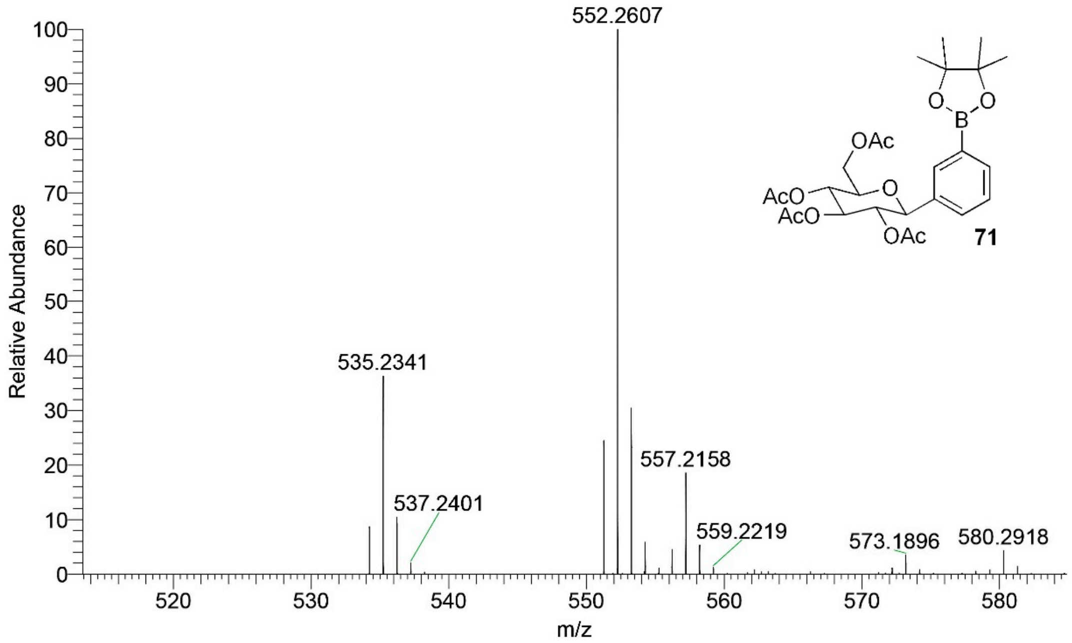

Figure S197 HR-MS (ESI/ion trap) spectrum of **71**

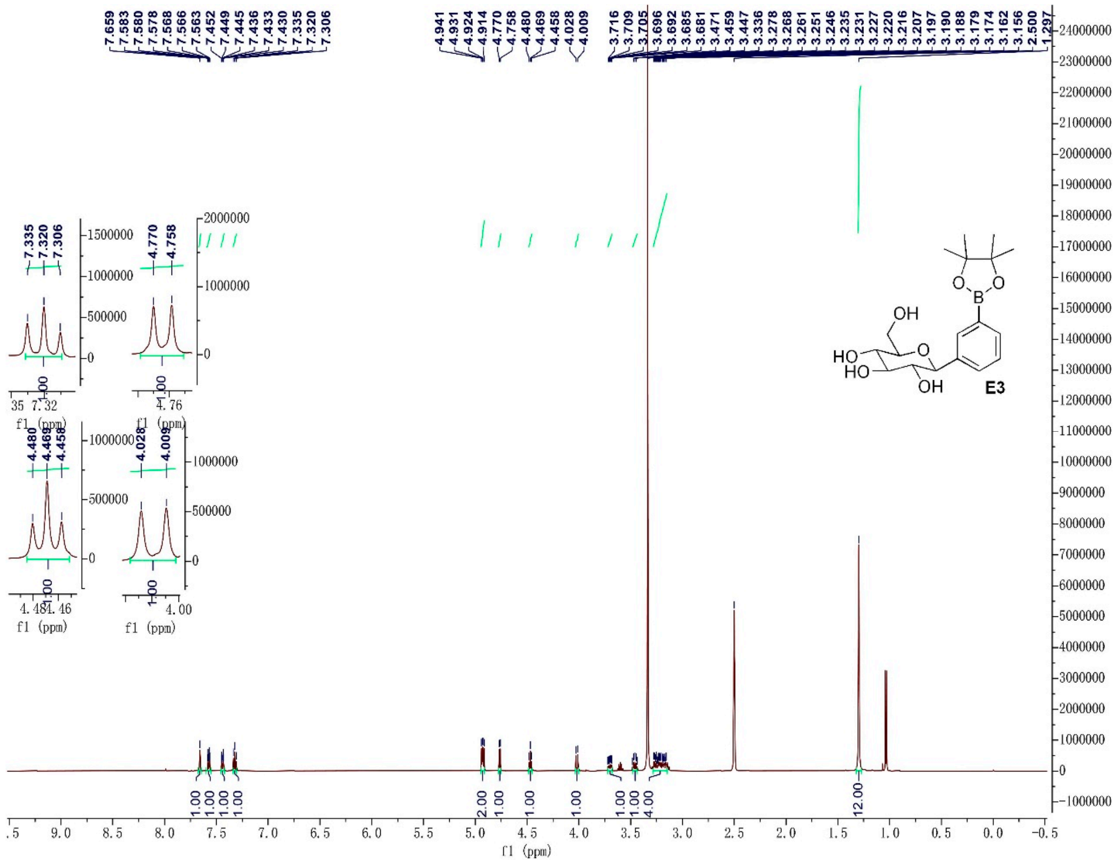

Figure S198 ^{1}H NMR spectrum of **E3**

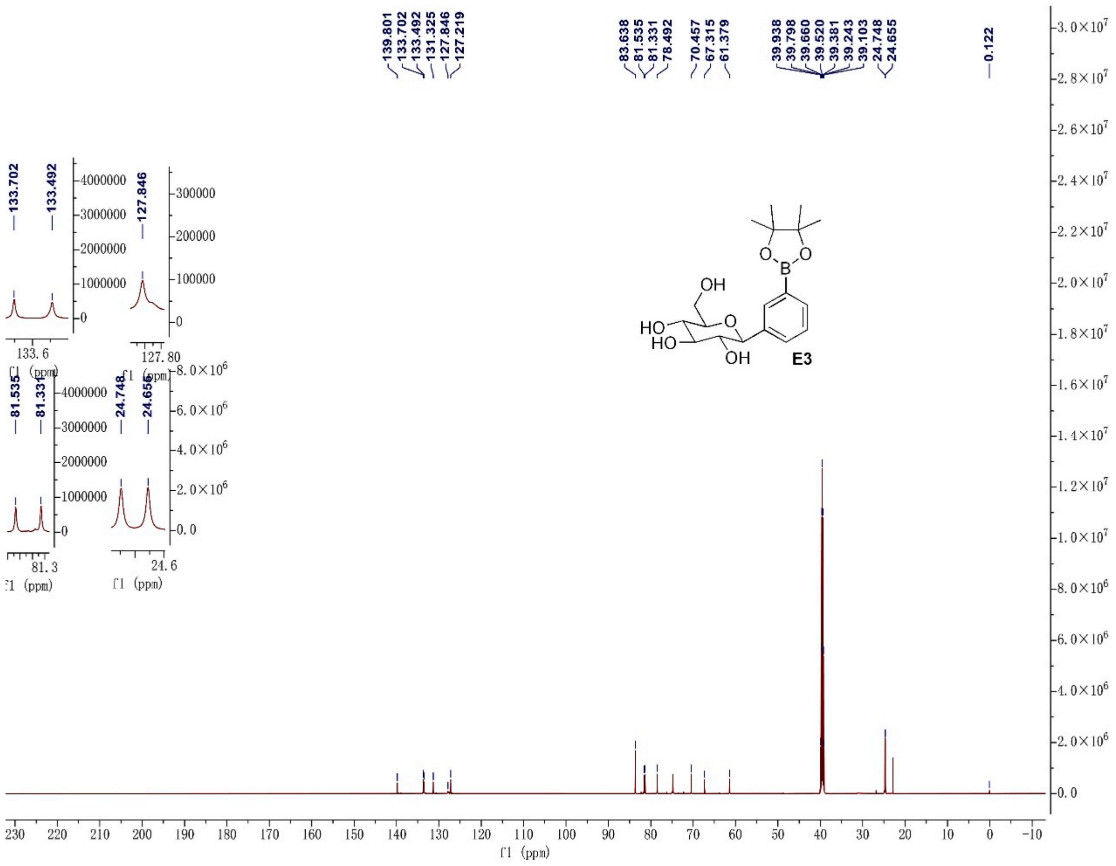

Figure S199 ^{13}C NMR spectrum of **E3**

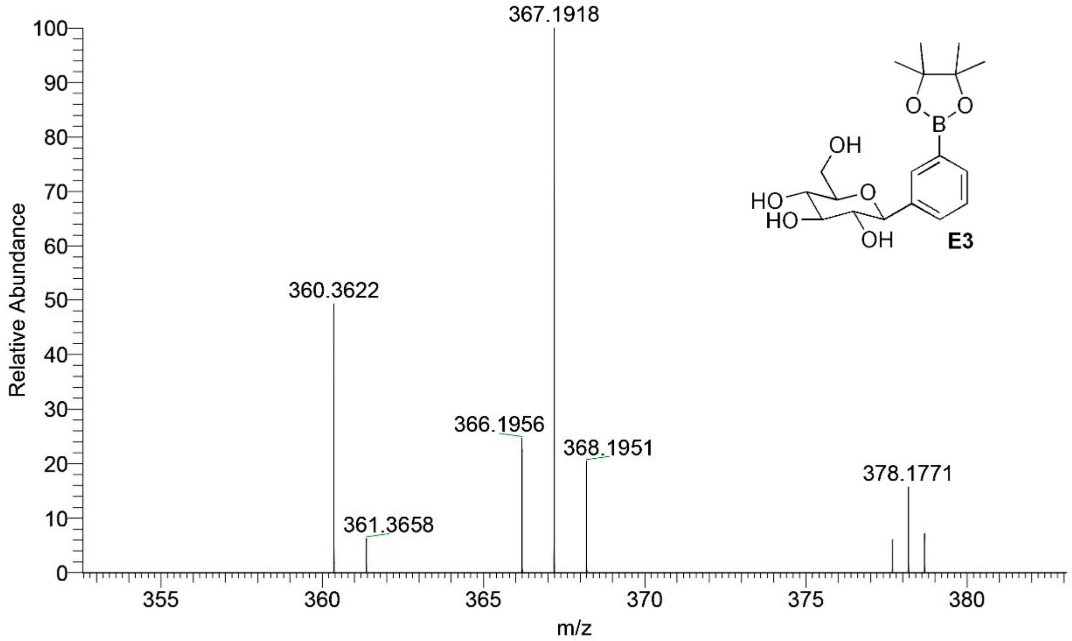

Figure S200 HR-MS (ESI/ion trap) spectrum of **E3**

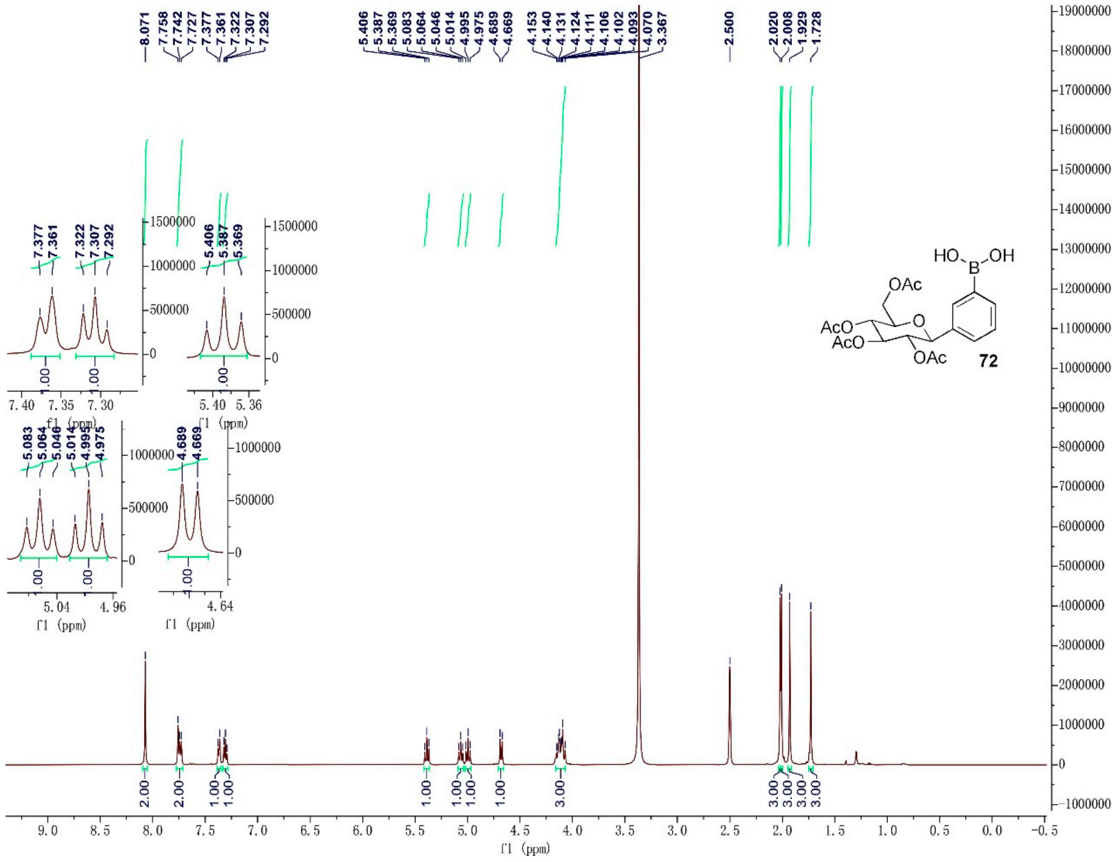

Figure S201 ^{1}H NMR spectrum of **72**

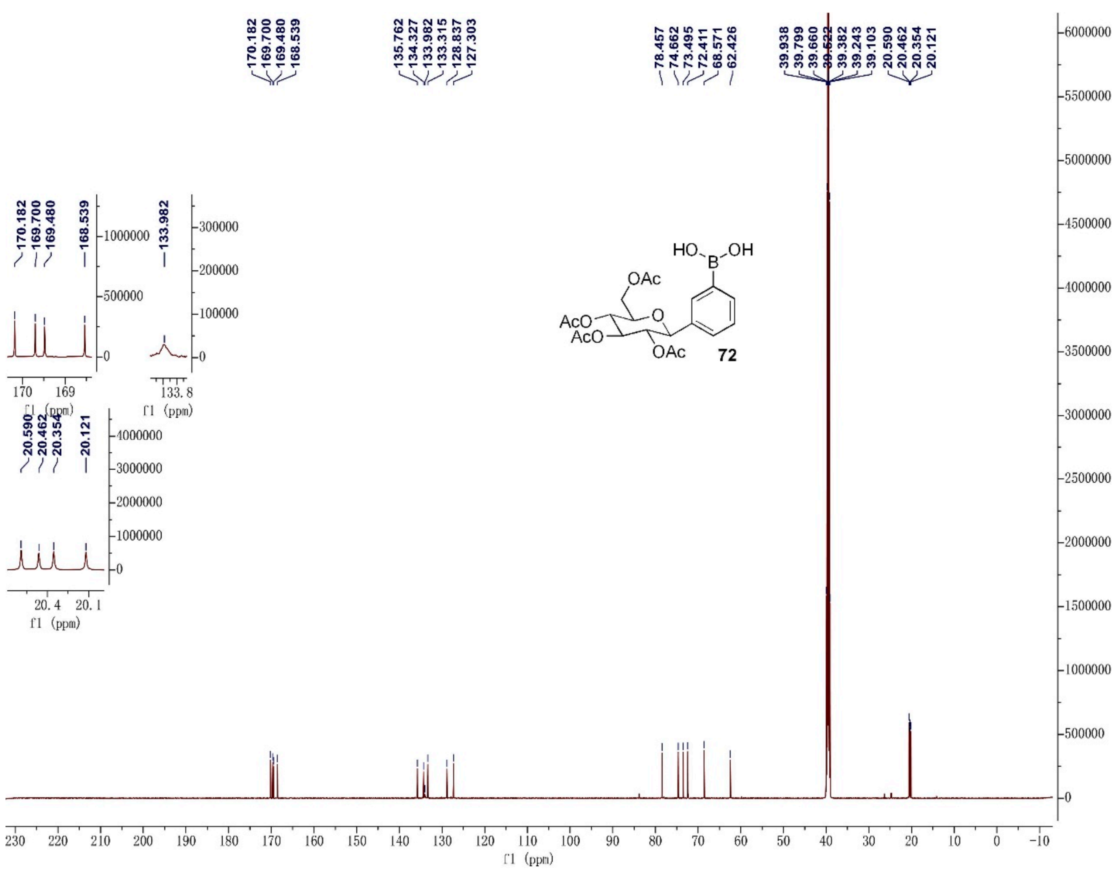

Figure S202 ^{13}C NMR spectrum of **72**

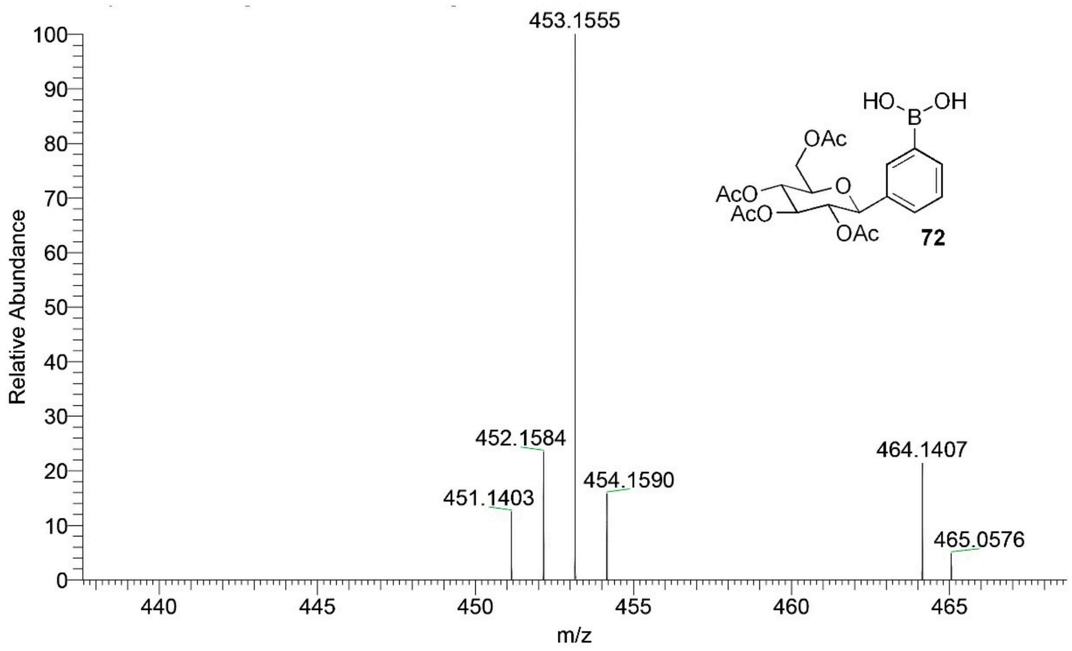

Figure S203 HR-MS (ESI/ion trap) spectrum of **72**

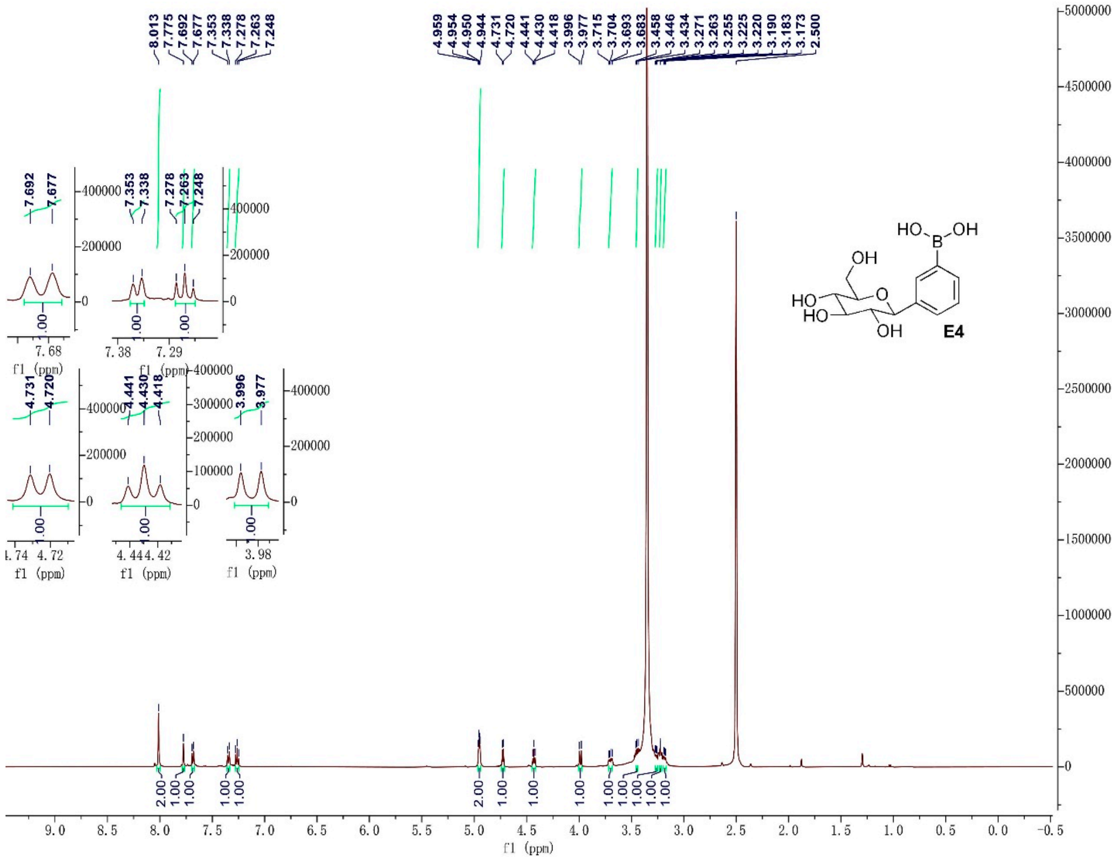

Figure S204 ^{1}H NMR spectrum of **E4**

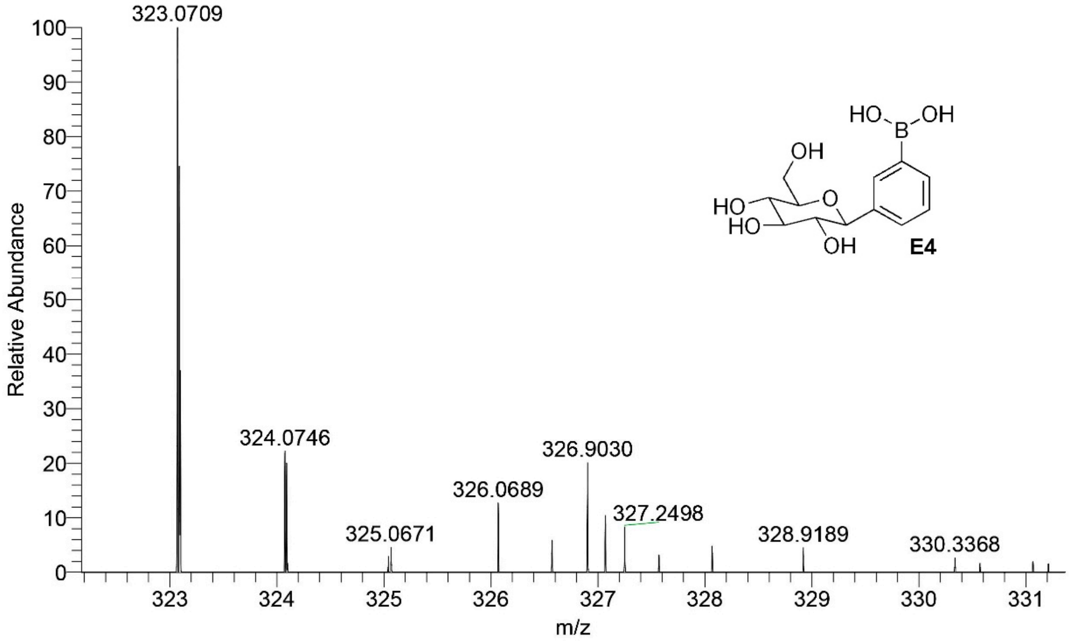

Figure S205 HR-MS (ESI/ion trap) spectrum of **E4**

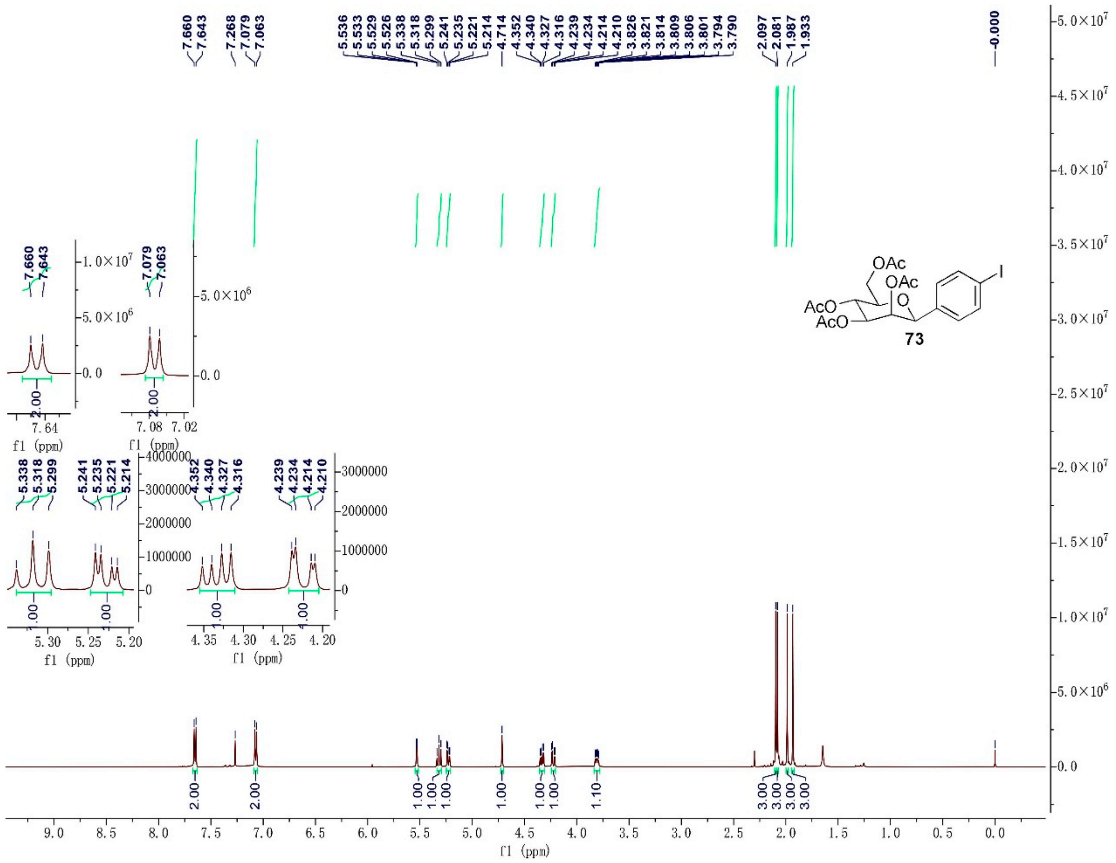

Figure S206 ^{1}H NMR spectrum of **73**

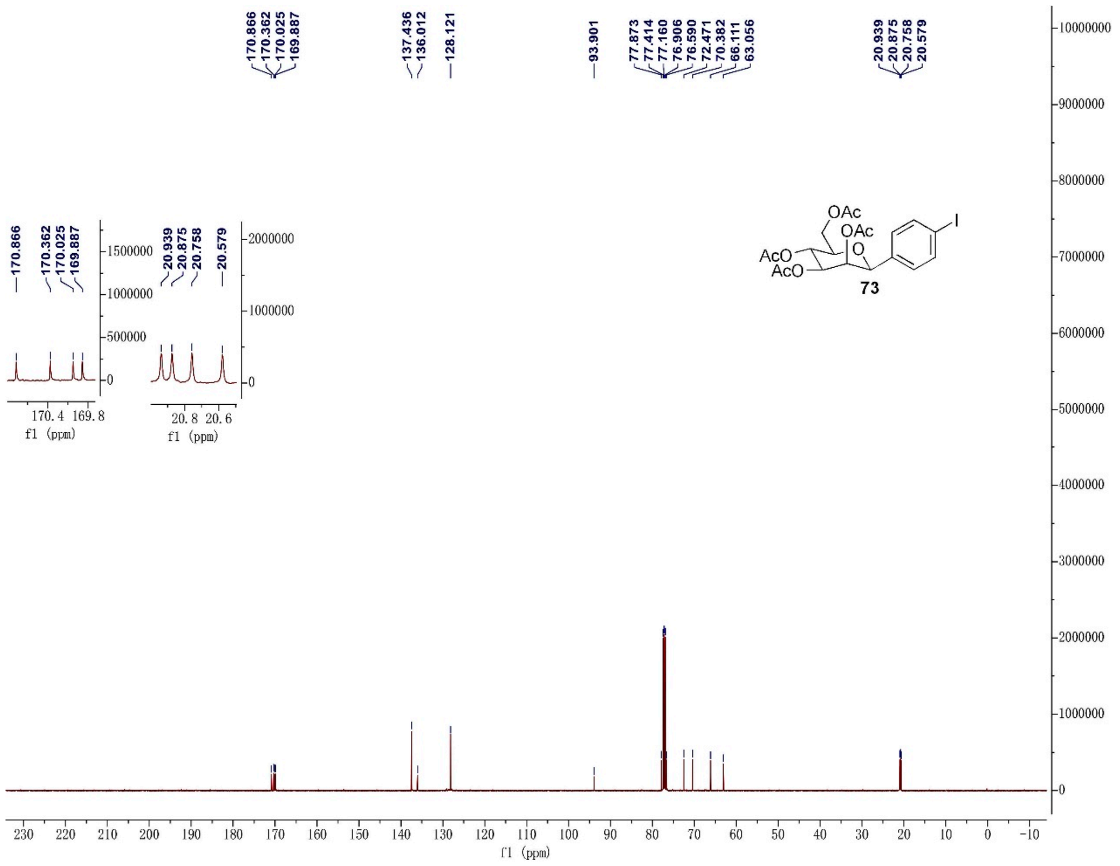

Figure S207 ^{13}C NMR spectrum of **73**

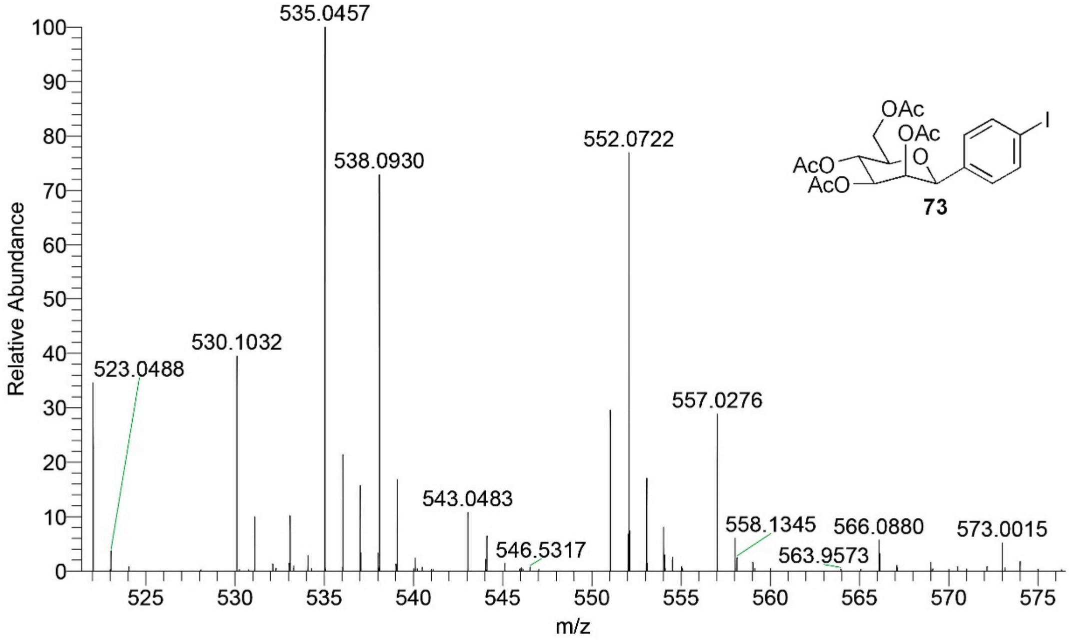

Figure S208 HR-MS (ESI/ion trap) spectrum of **73**

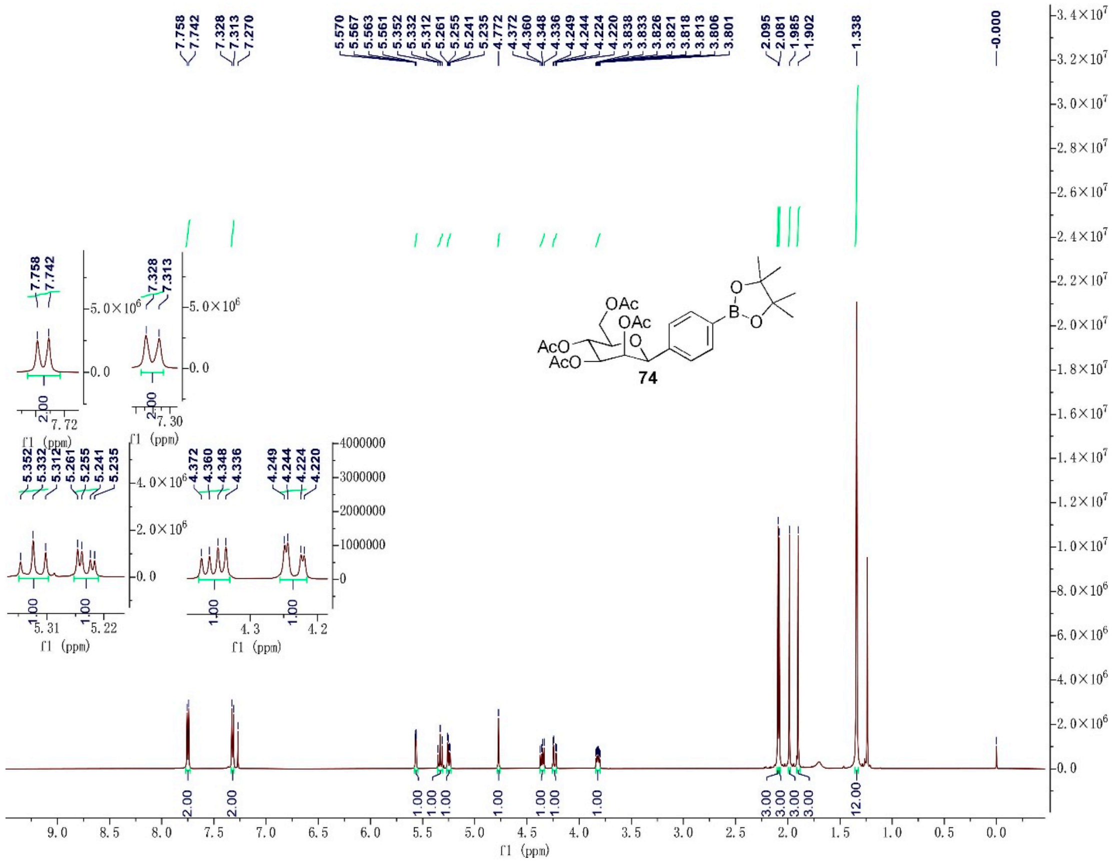

Figure S209 ^{1}H NMR spectrum of **74**

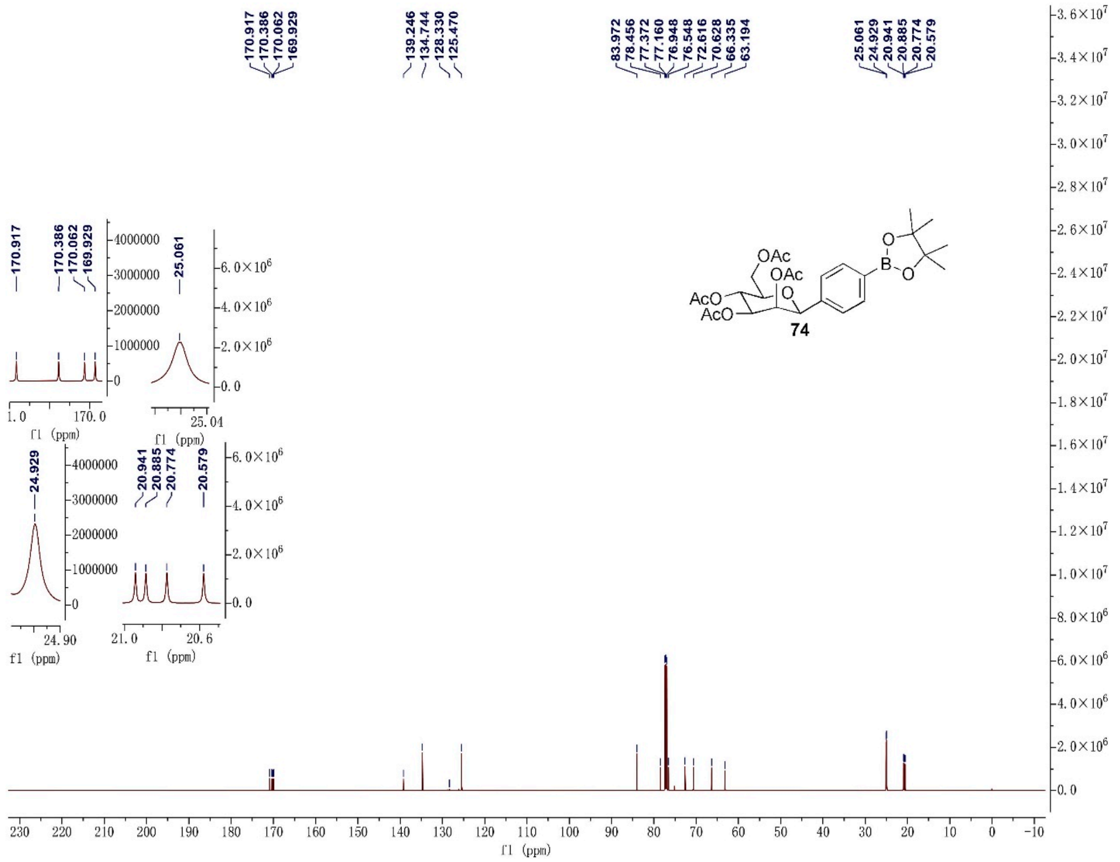

Figure S210 ^{13}C NMR spectrum of **74**

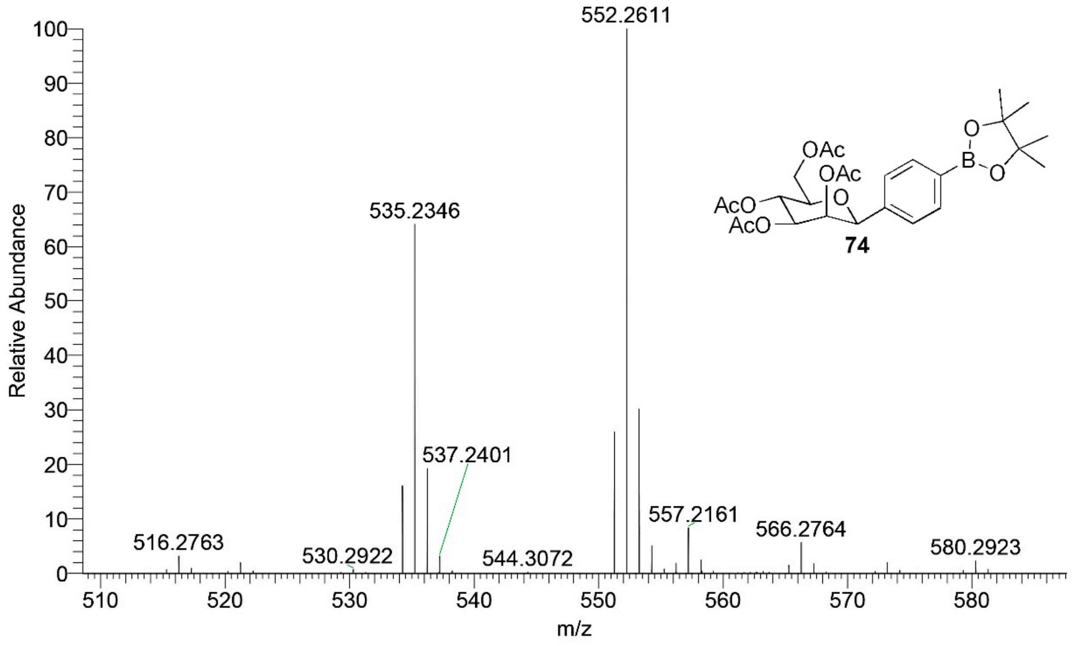

Figure S211 HR-MS (ESI/ion trap) spectrum of **74**

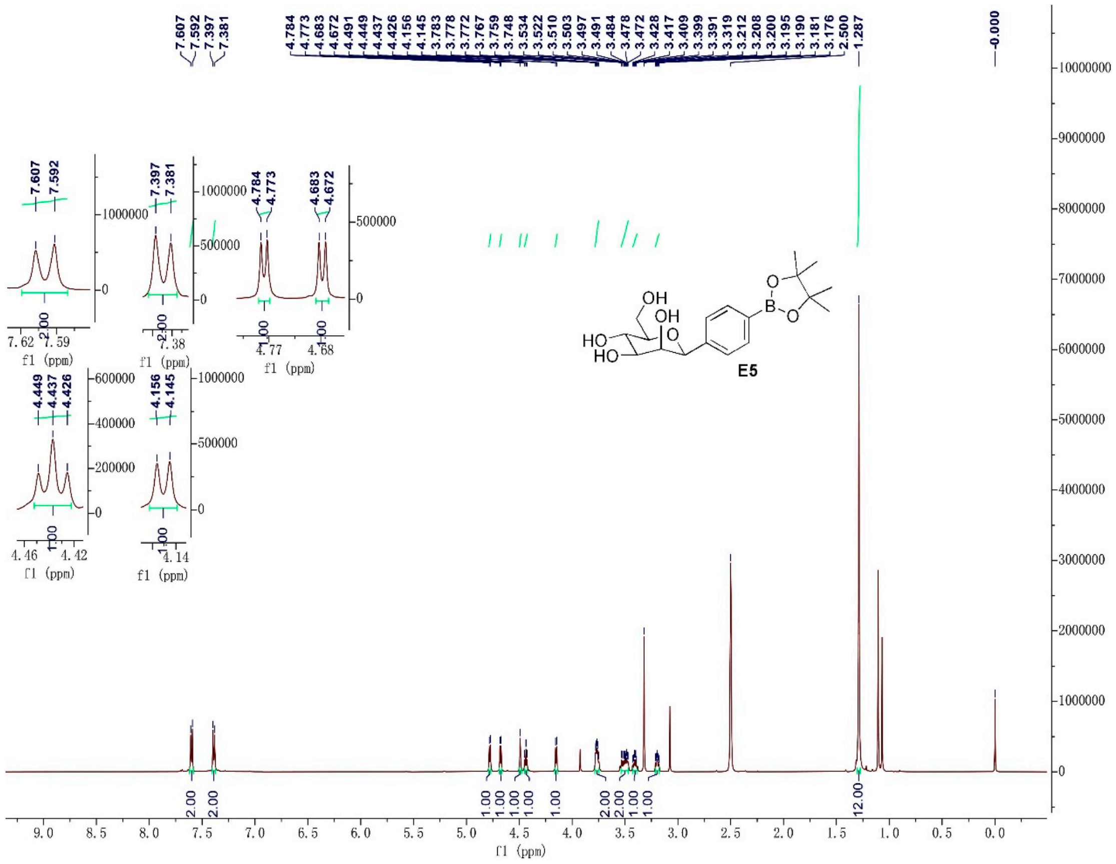

Figure S212 ^{1}H NMR spectrum of **E5**

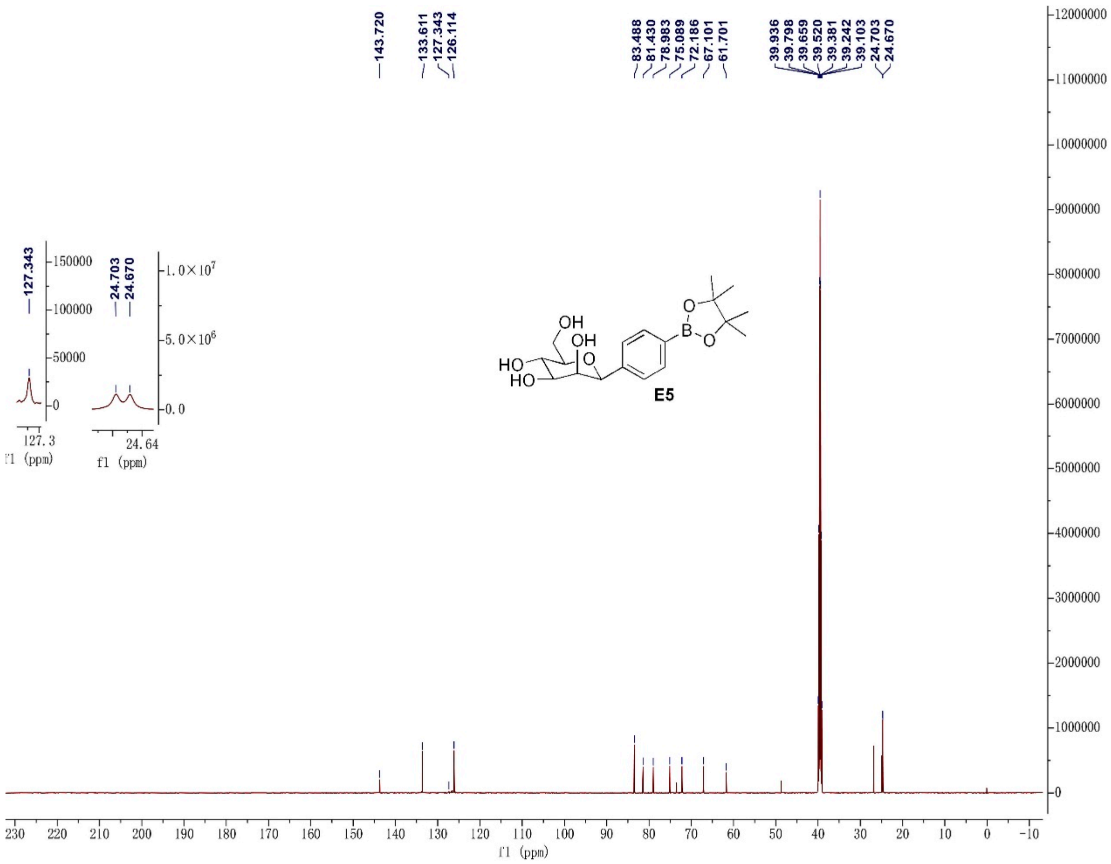

Figure S213 ^{13}C NMR spectrum of **E5**

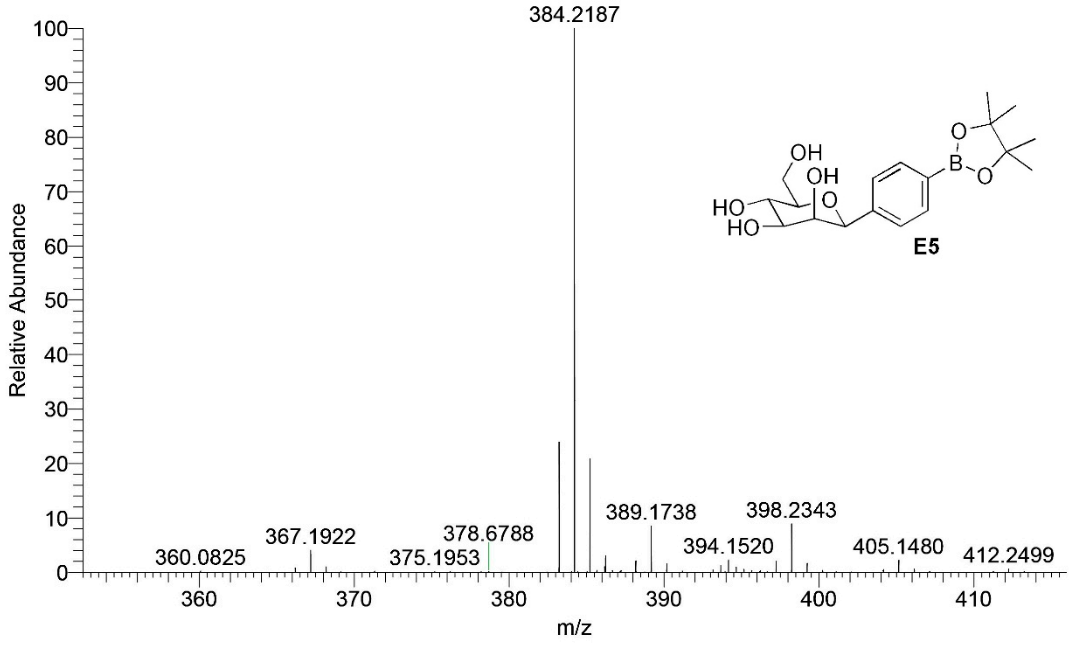

Figure S214 HR-MS (ESI/ion trap) spectrum of **E5**

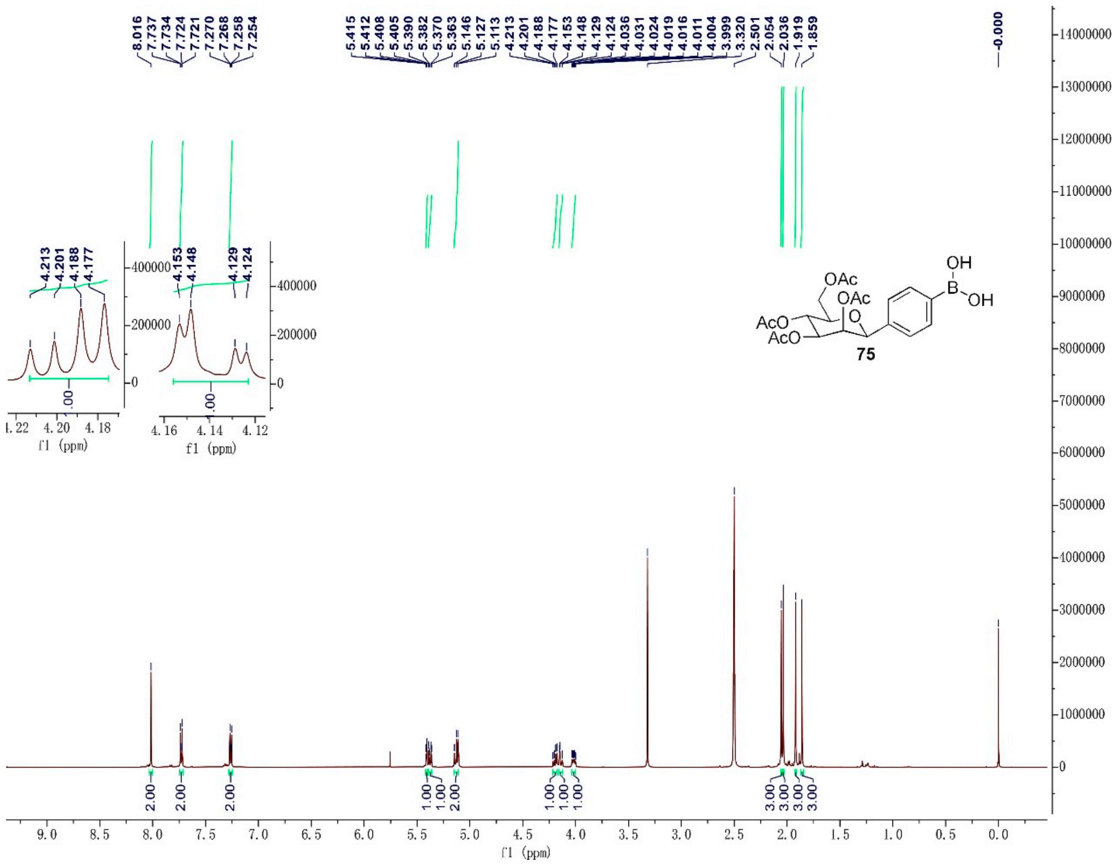

Figure S215 ^{1}H NMR spectrum of **75**

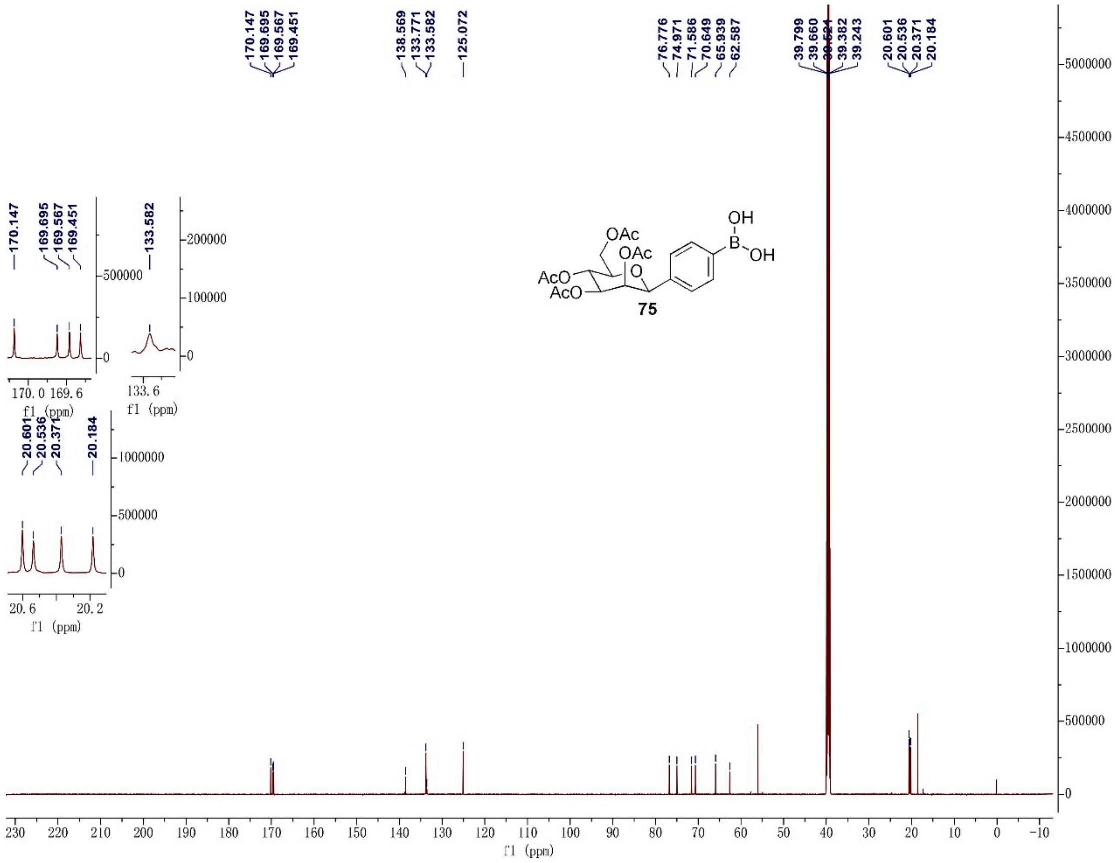

Figure S216 ^{13}C NMR spectrum of **75**

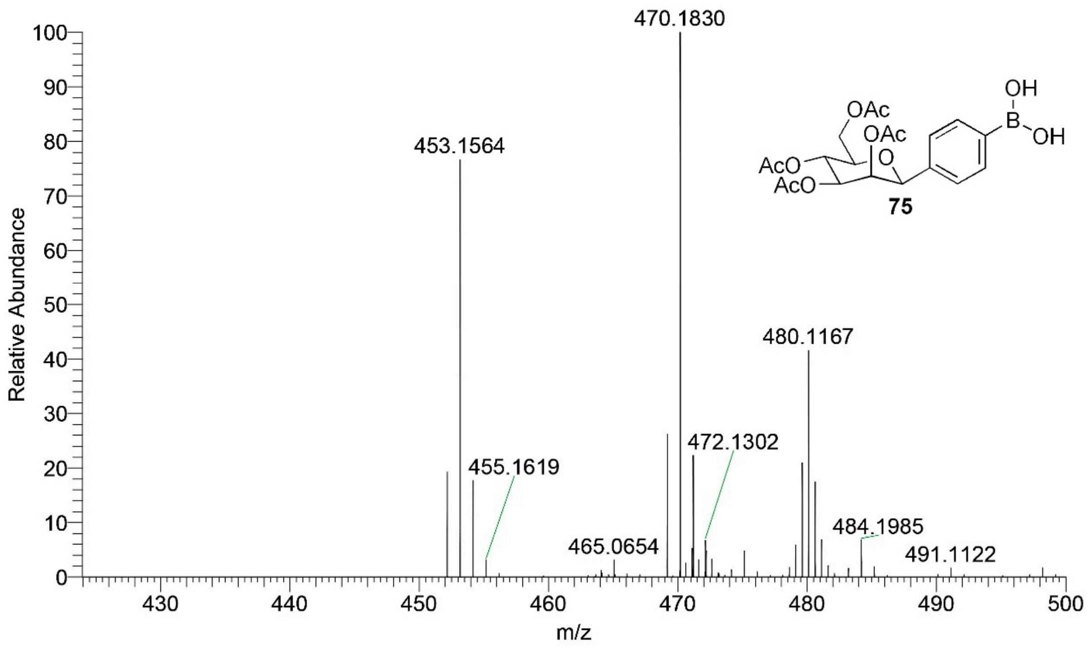

Figure S217 HR-MS (ESI/ion trap) spectrum of **75**

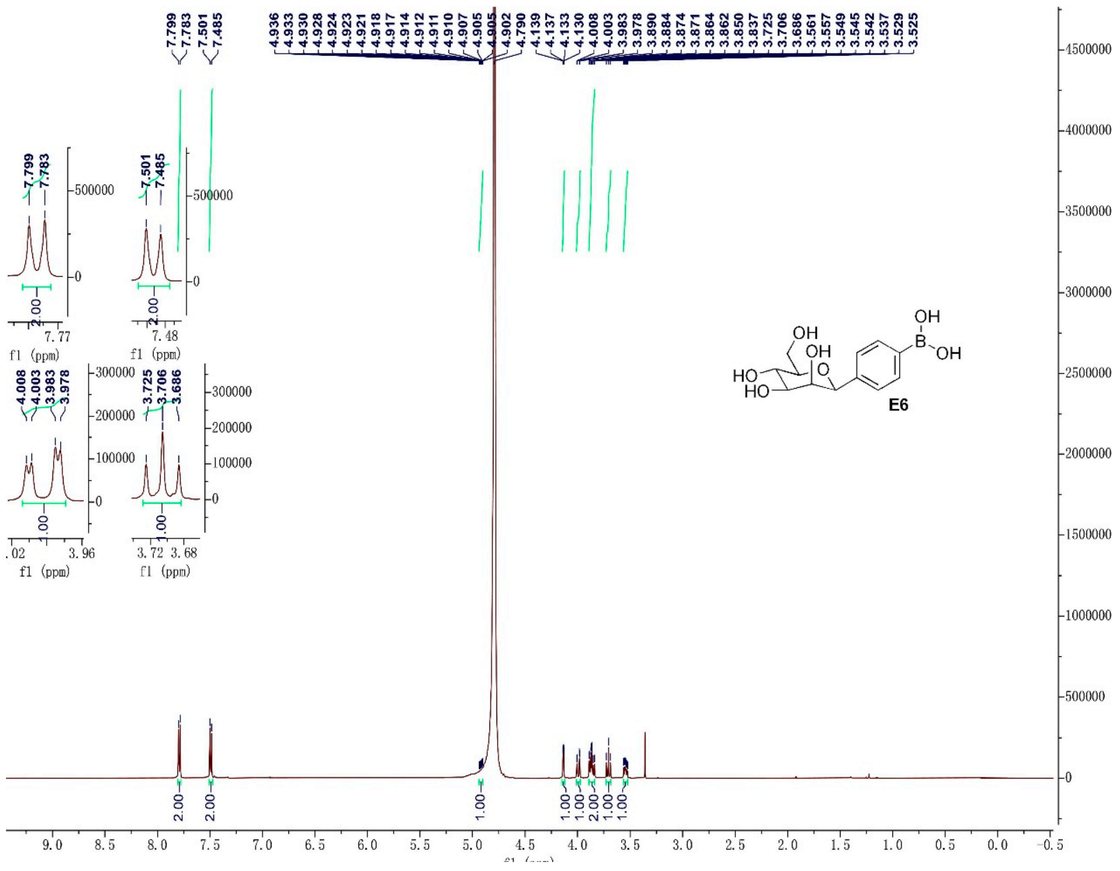

Figure S218 ^{1}H NMR spectrum of **E6**

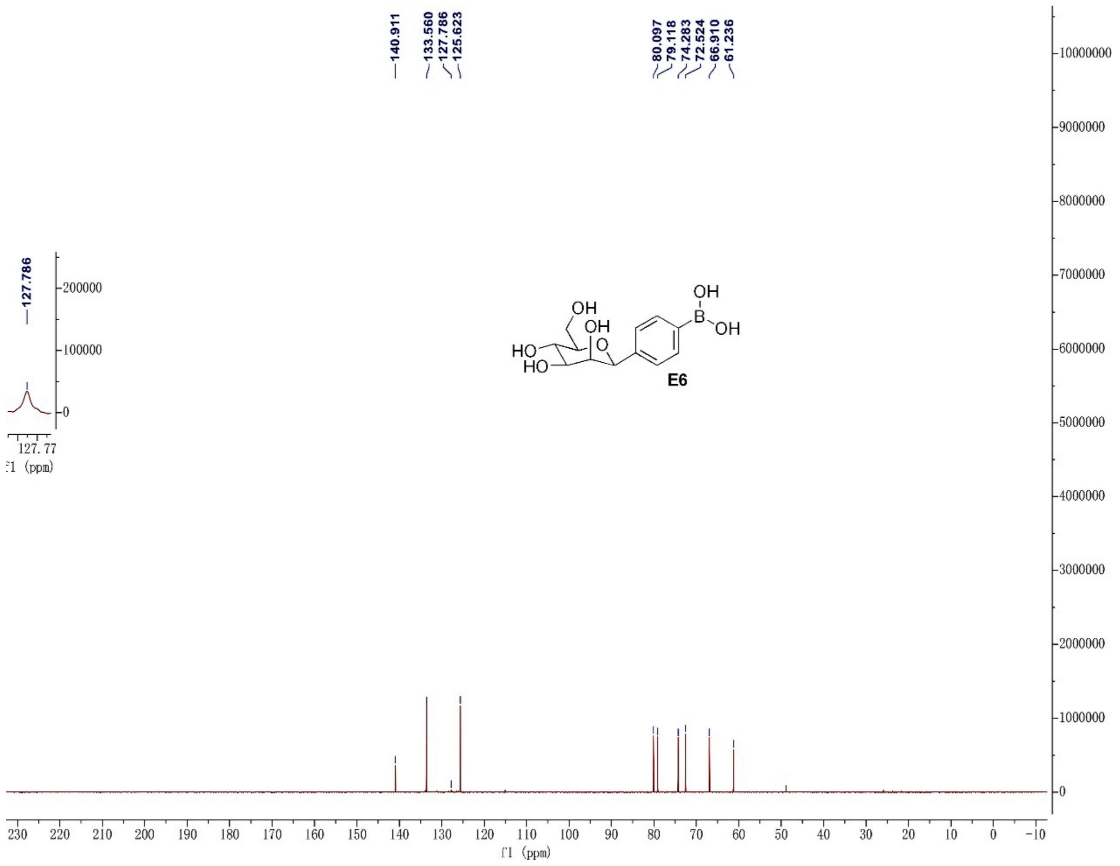

Figure S219 ^{13}C NMR spectrum of **E6**

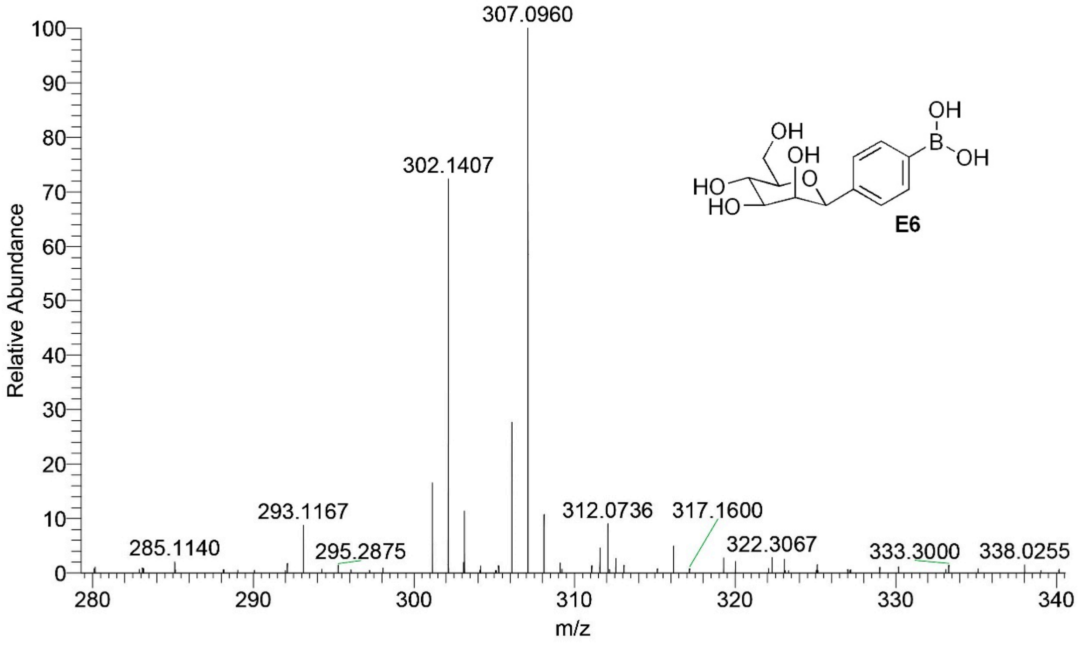

Figure S220 HR-MS (ESI/ion trap) spectrum of **E6**

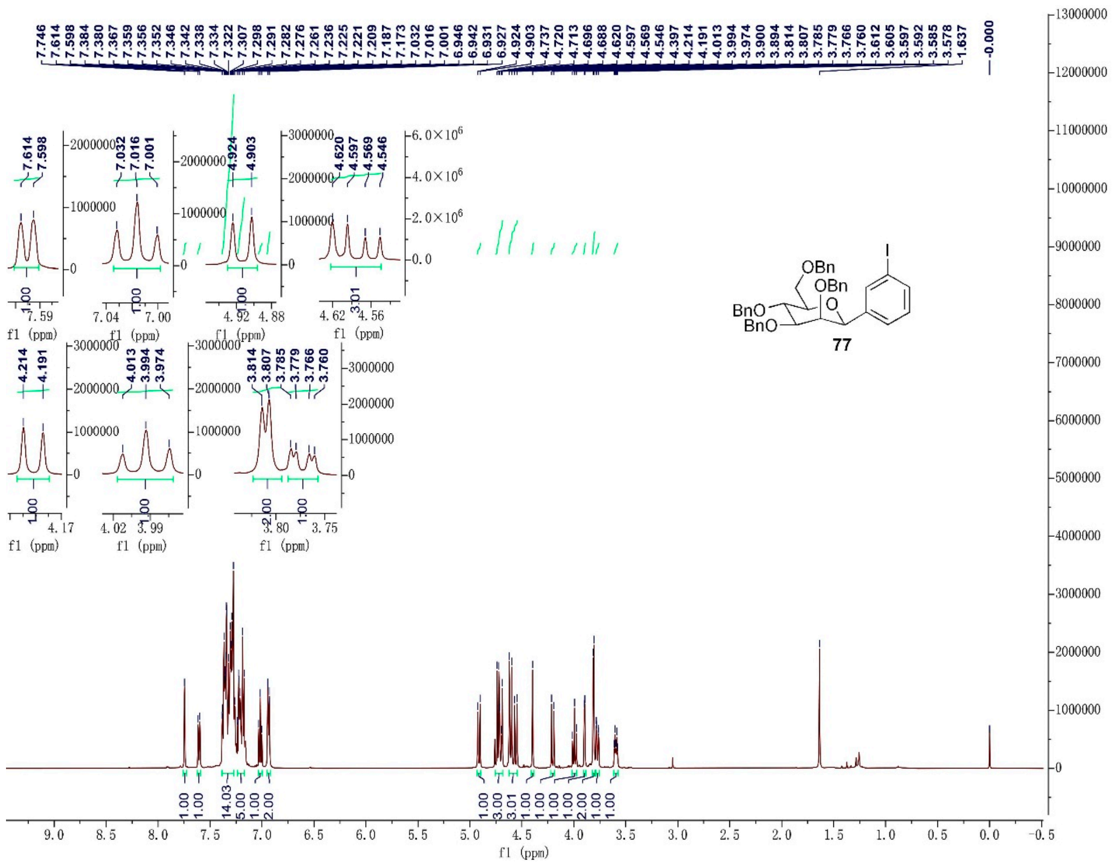

Figure S221 ^{1}H NMR spectrum of **77**

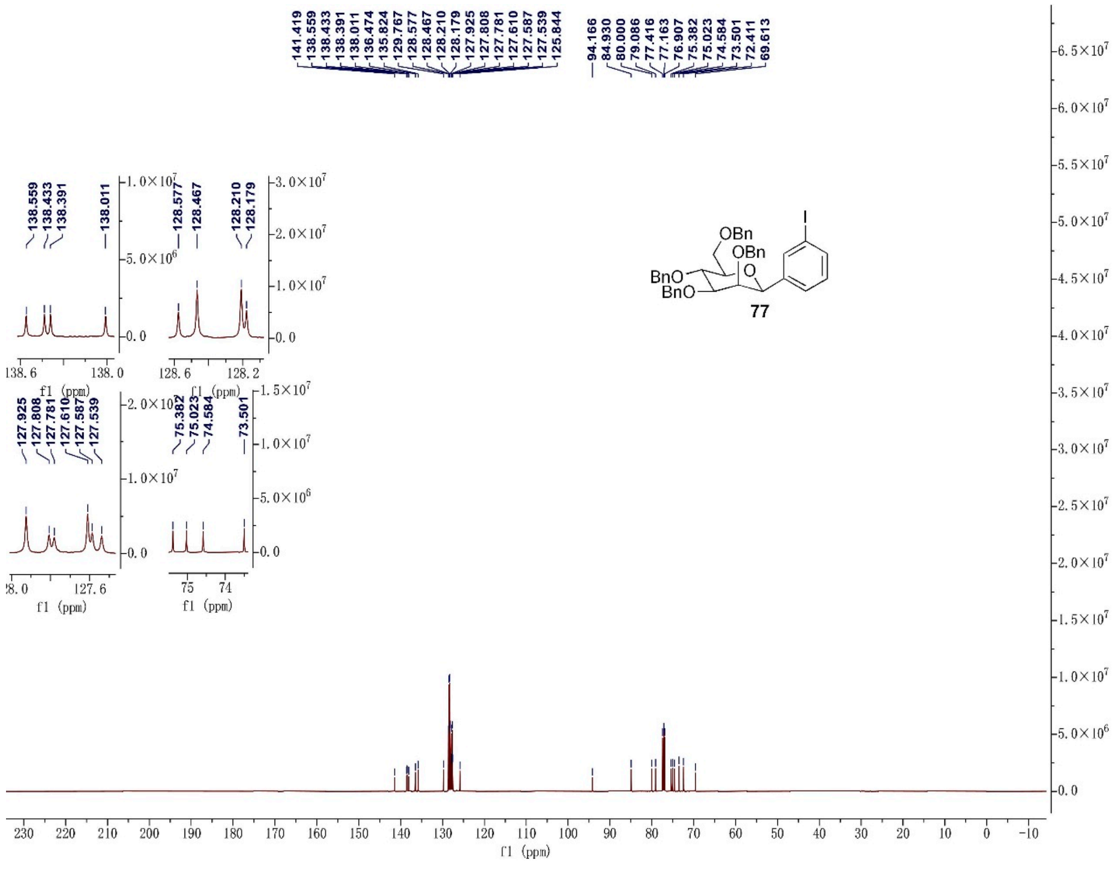

Figure S222 ^{13}C NMR spectrum of **77**

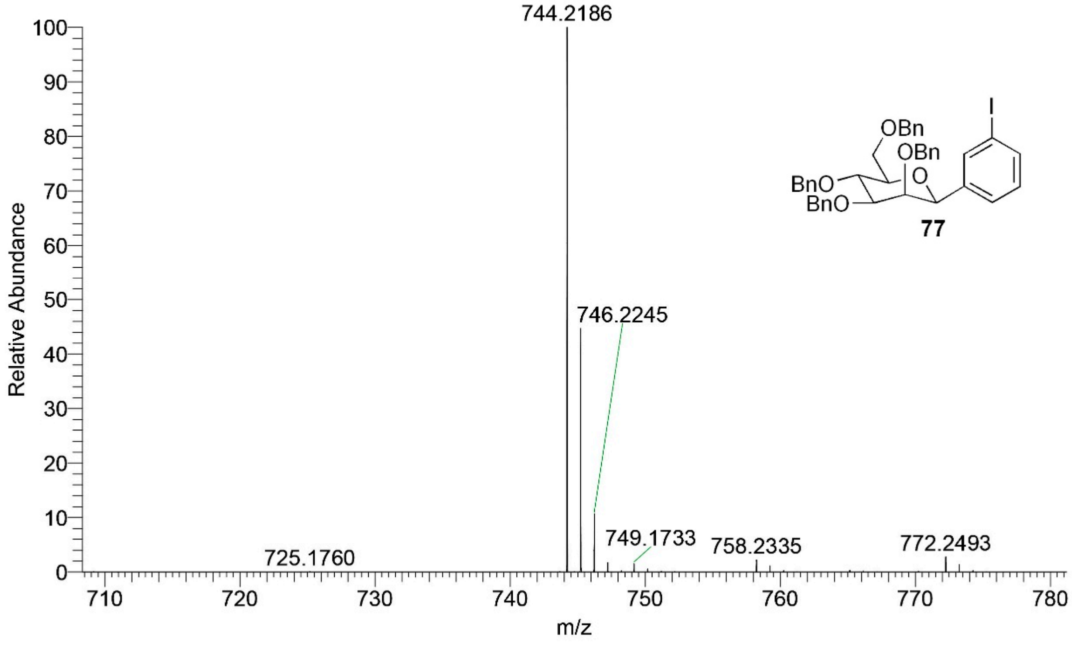

Figure S223 HR-MS (ESI/ion trap) spectrum of **77**

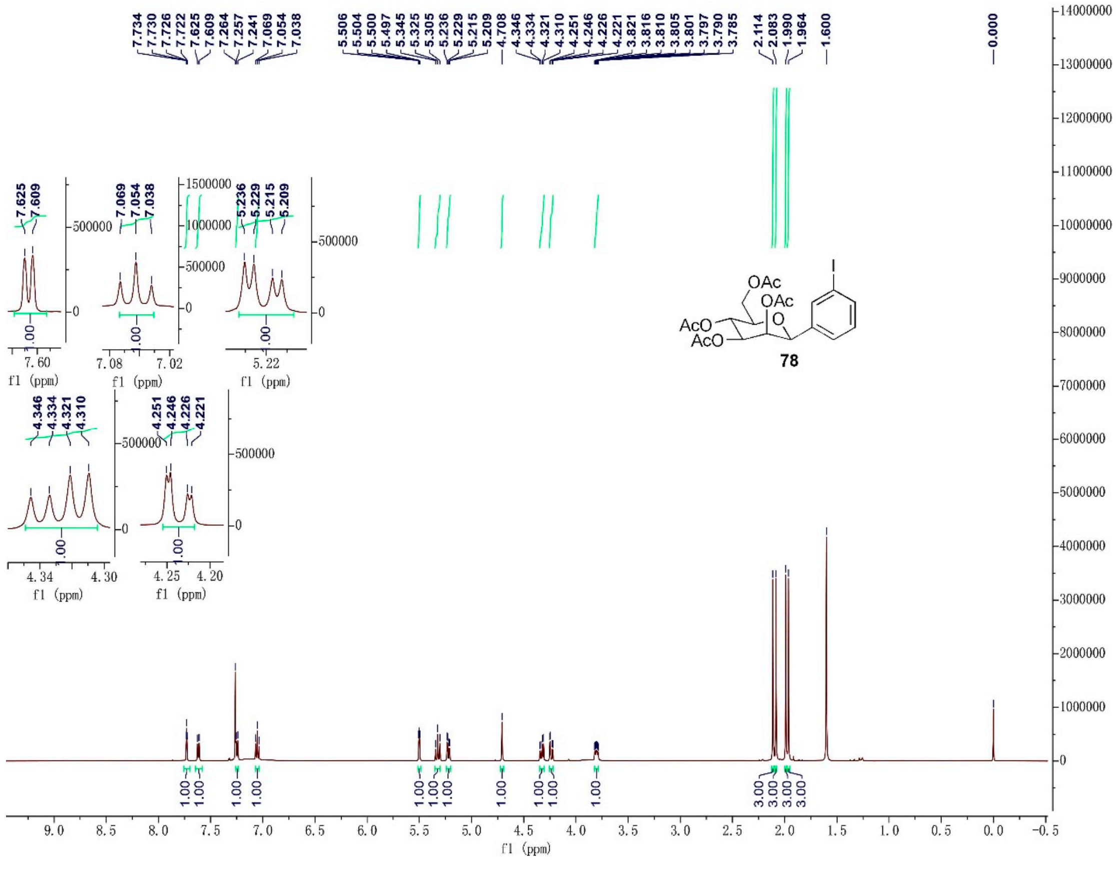

Figure S224 ^{1}H NMR spectrum of **78**

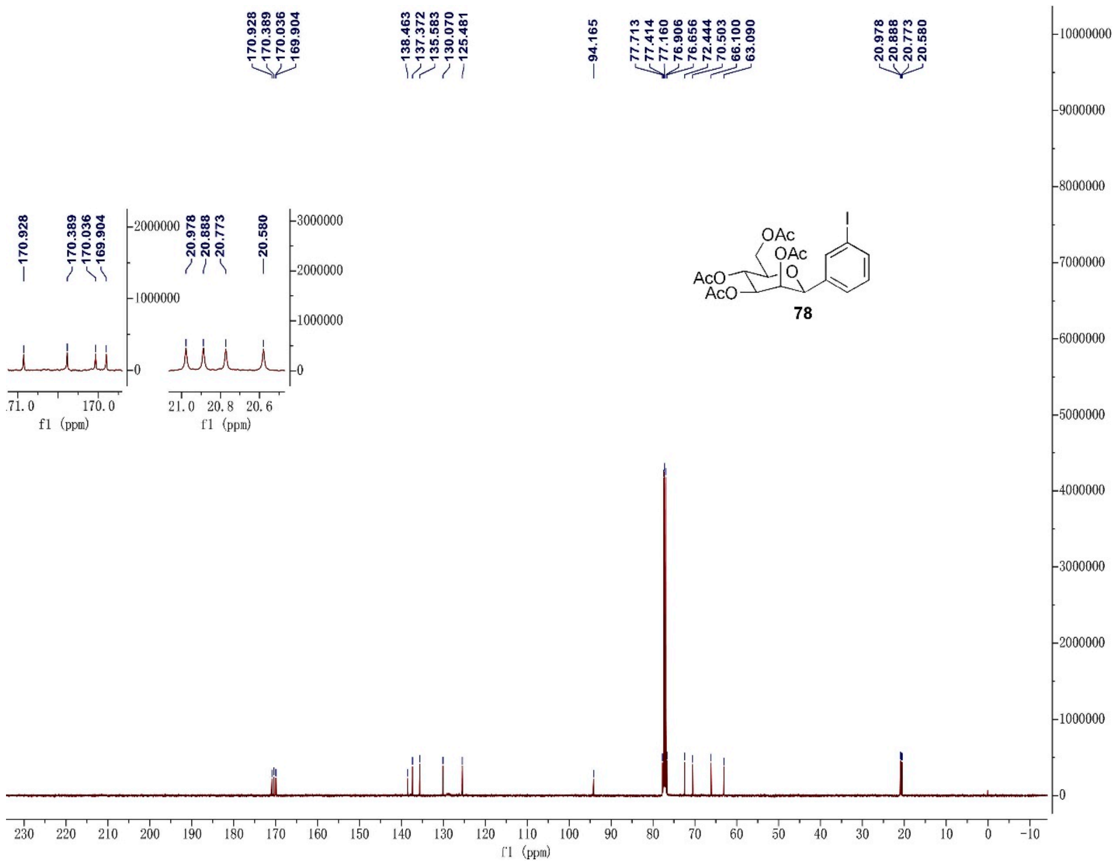

Figure S225 ^{13}C NMR spectrum of **78**

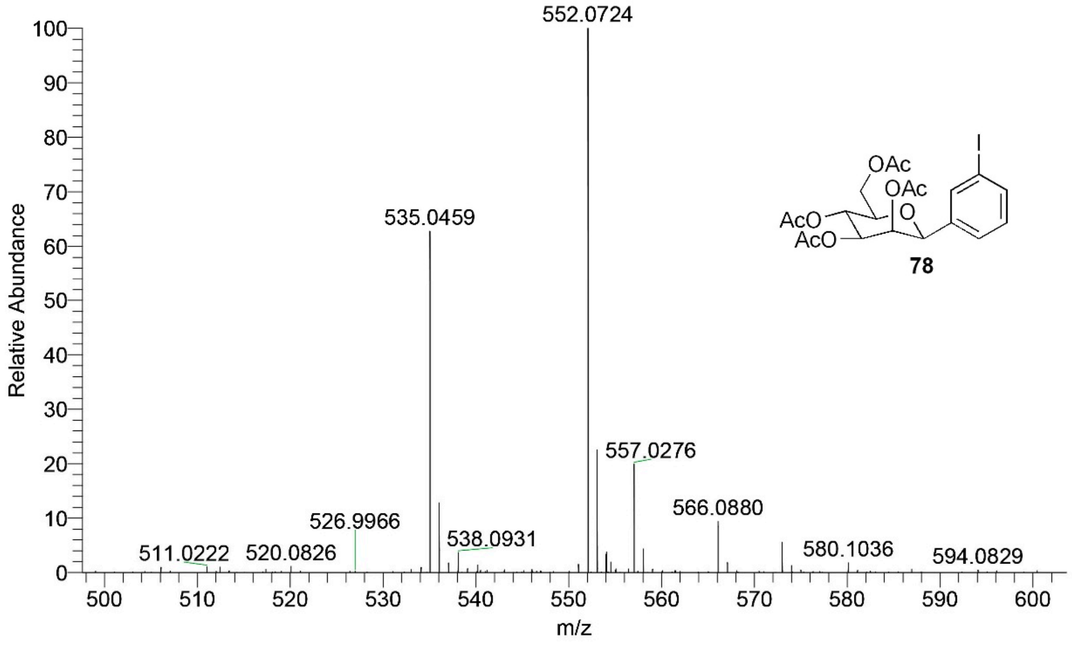

Figure S226 HR-MS (ESI/ion trap) spectrum of **78**

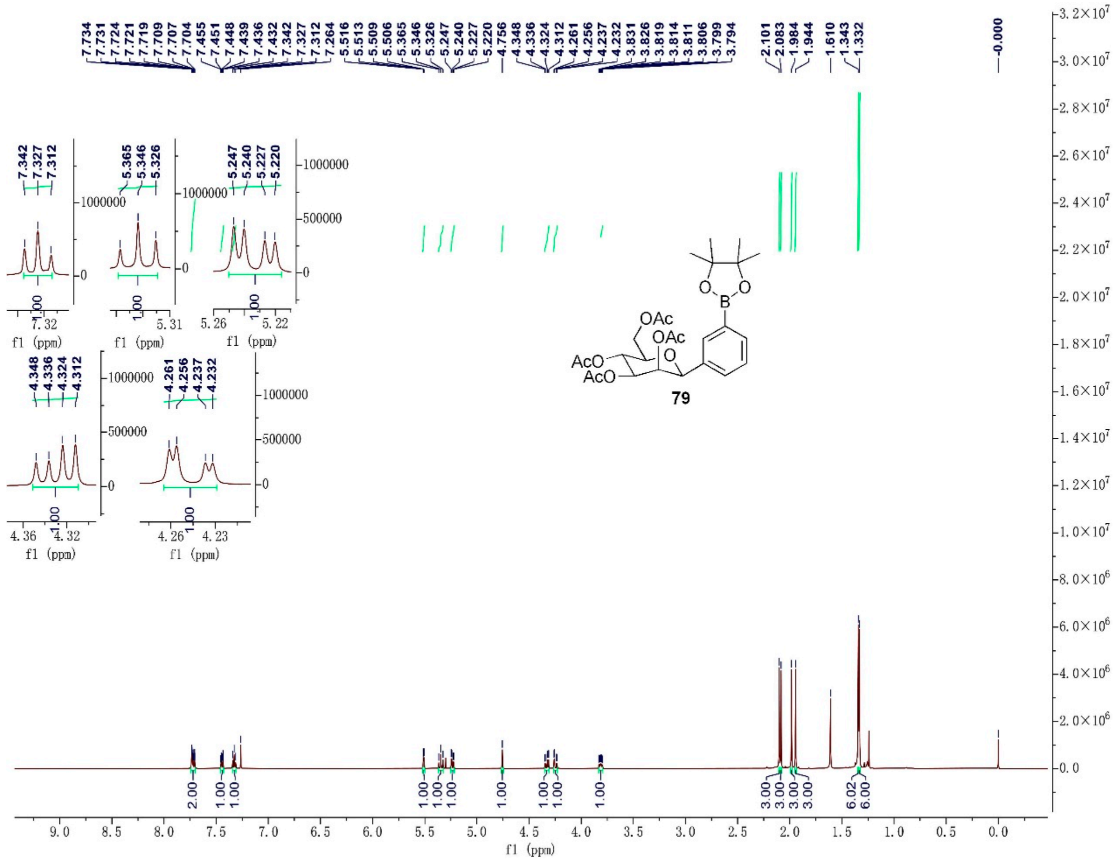

Figure S227 ^{1}H NMR spectrum of **79**

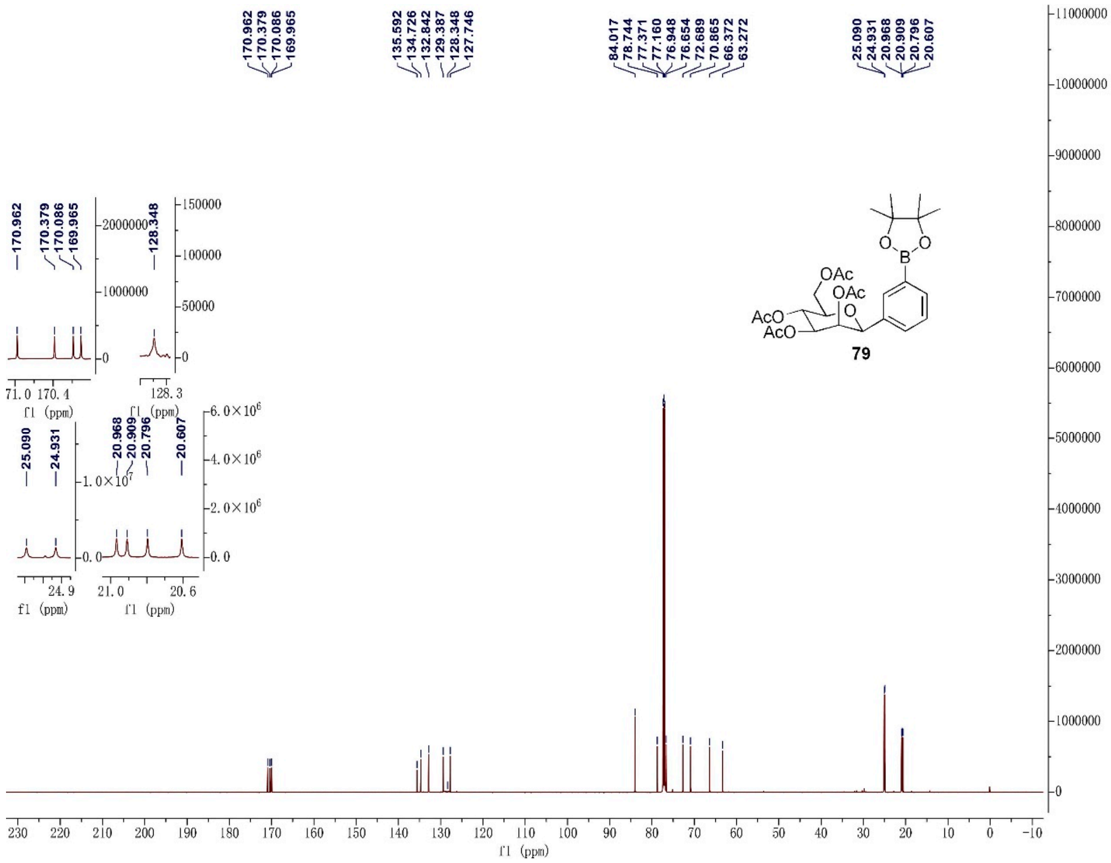

Figure S228 ^{13}C NMR spectrum of **79**

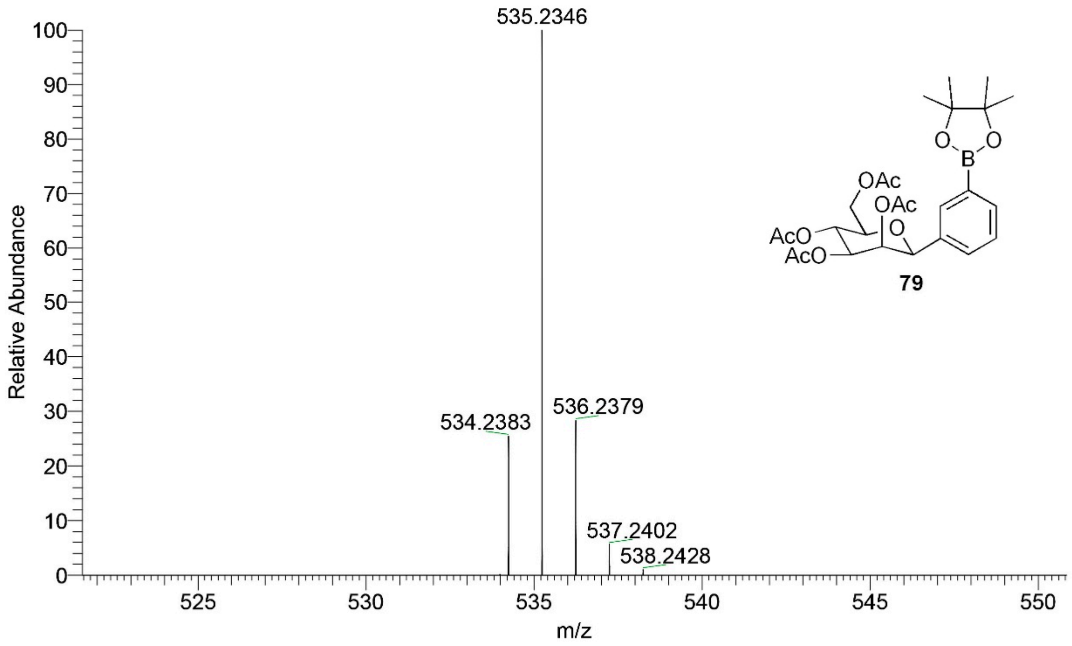

Figure S229 HR-MS (ESI/ion trap) spectrum of **79**

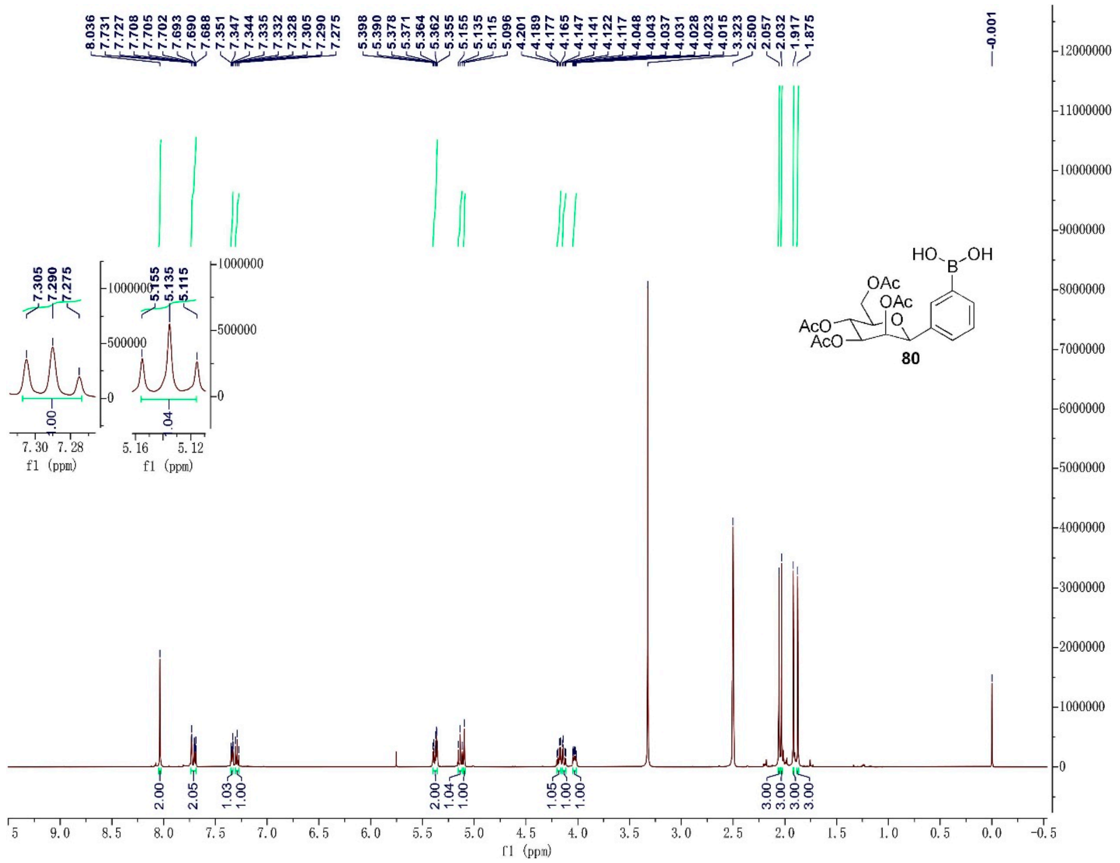

Figure S230 ^{1}H NMR spectrum of **80**

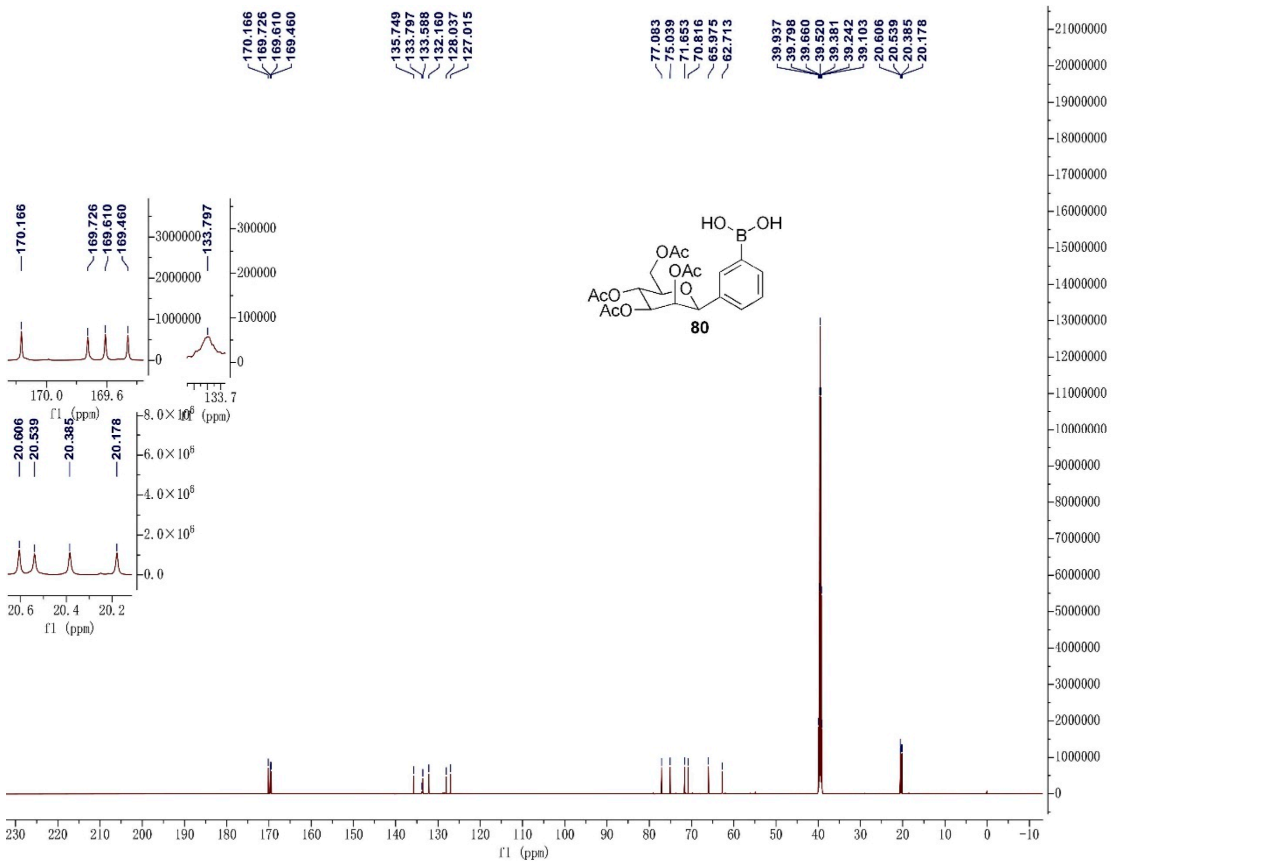

Figure S231 ^{13}C NMR spectrum of **80**

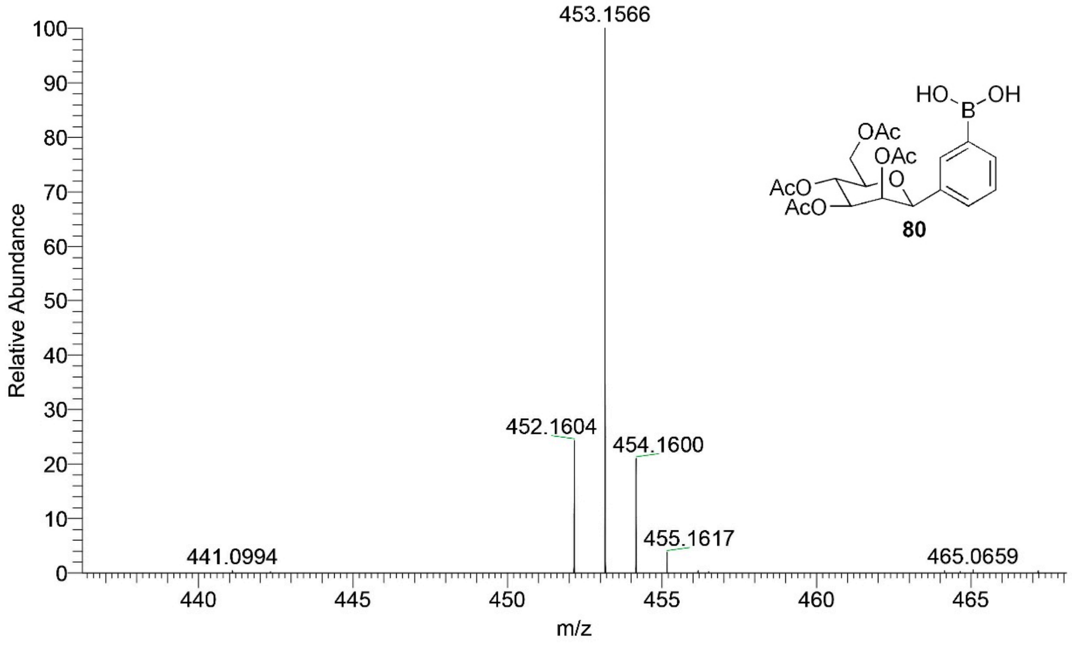

Figure S232 HR-MS (ESI/ion trap) spectrum of **80**

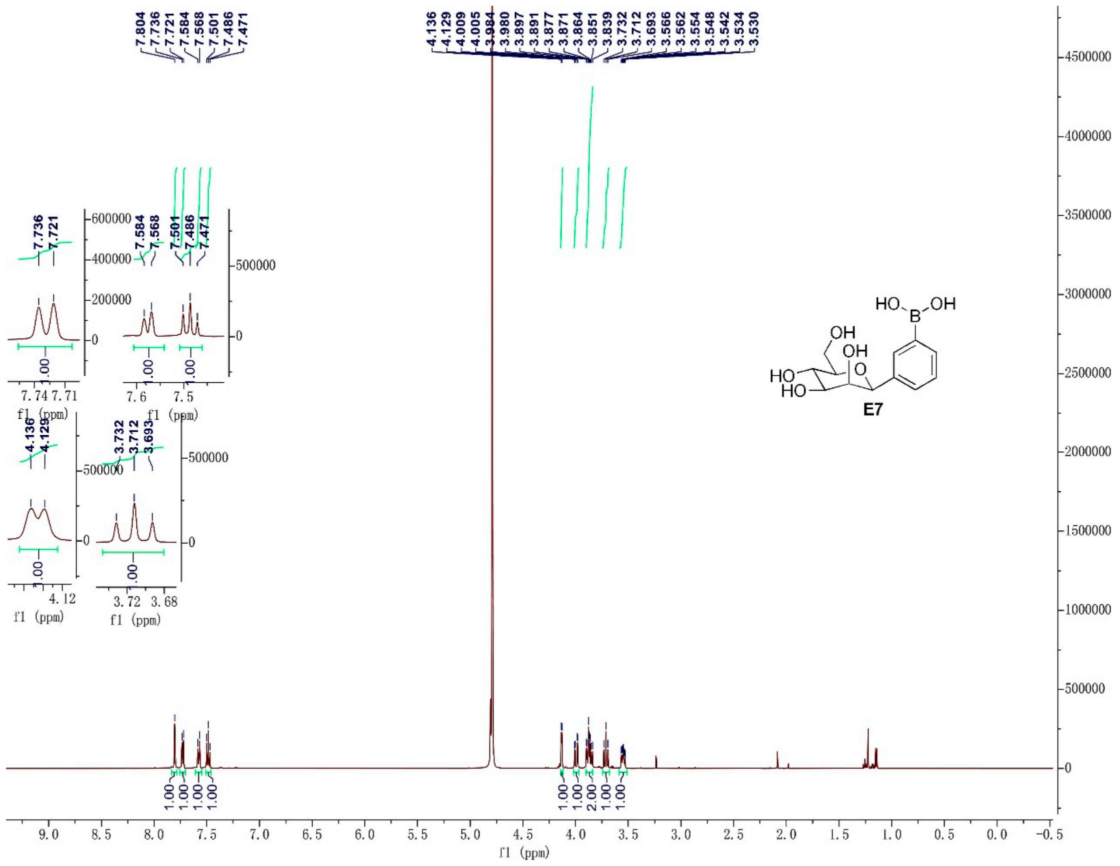

Figure S233 ^{1}H NMR spectrum of **E7**

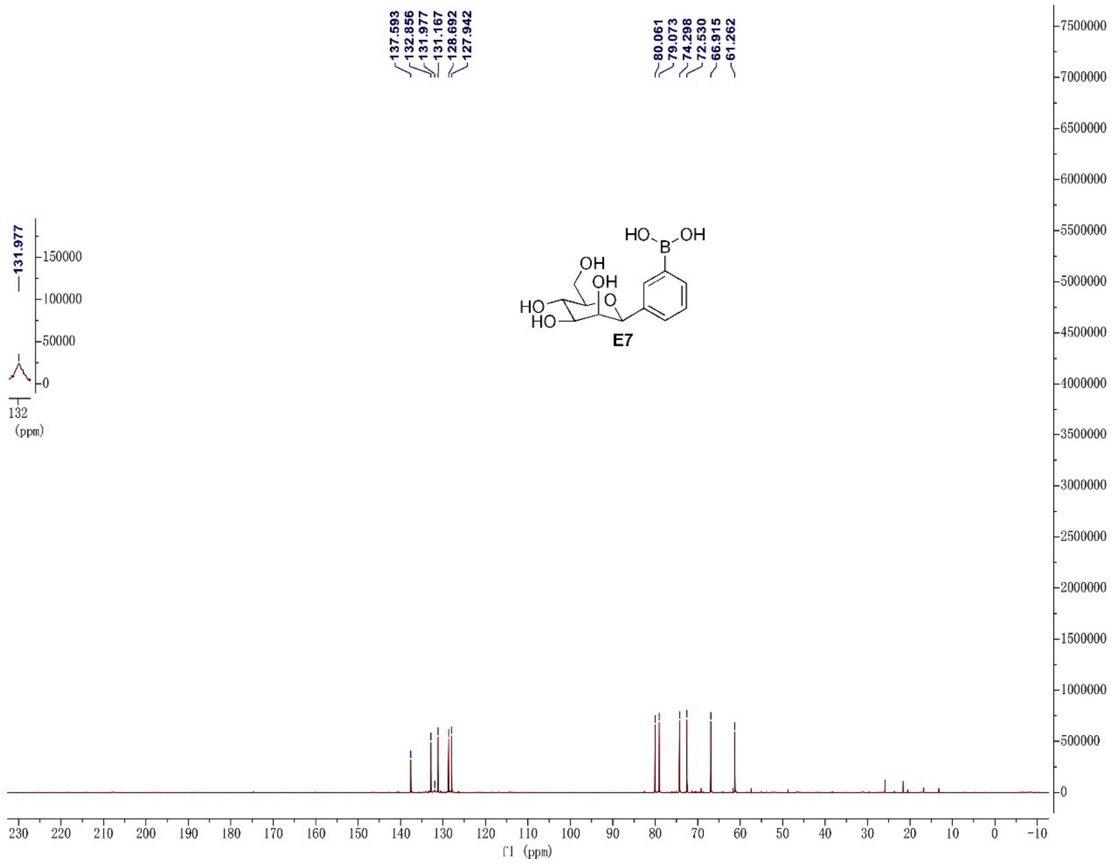

Figure S234 ^{13}C NMR spectrum of **E7**

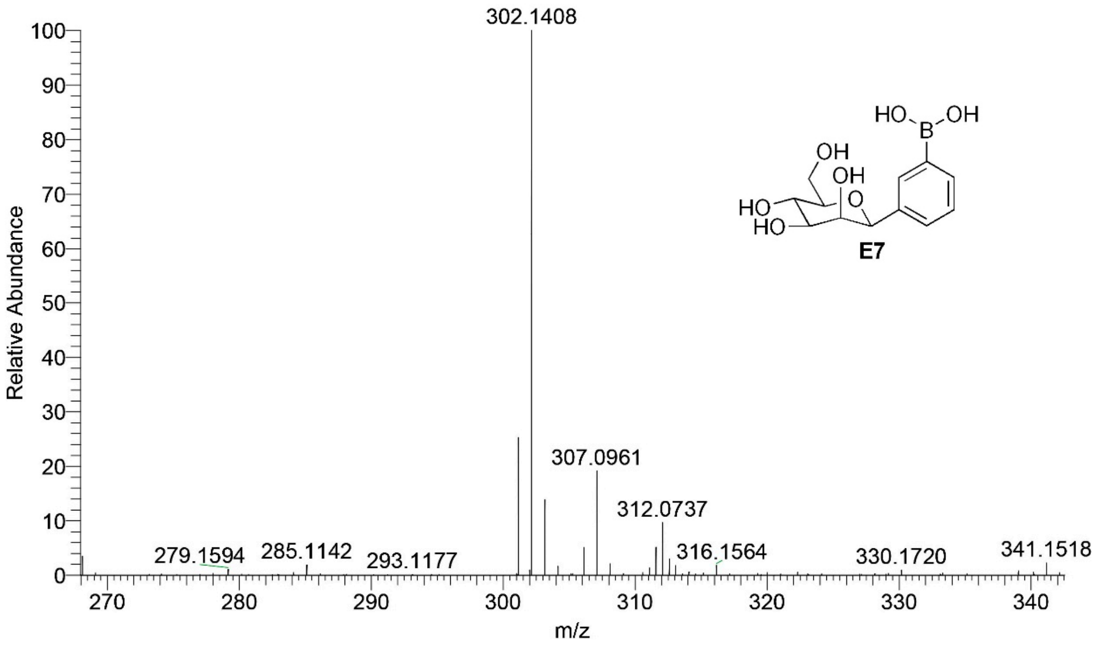

Figure S235 HR-MS (ESI/ion trap) spectrum of **E7**

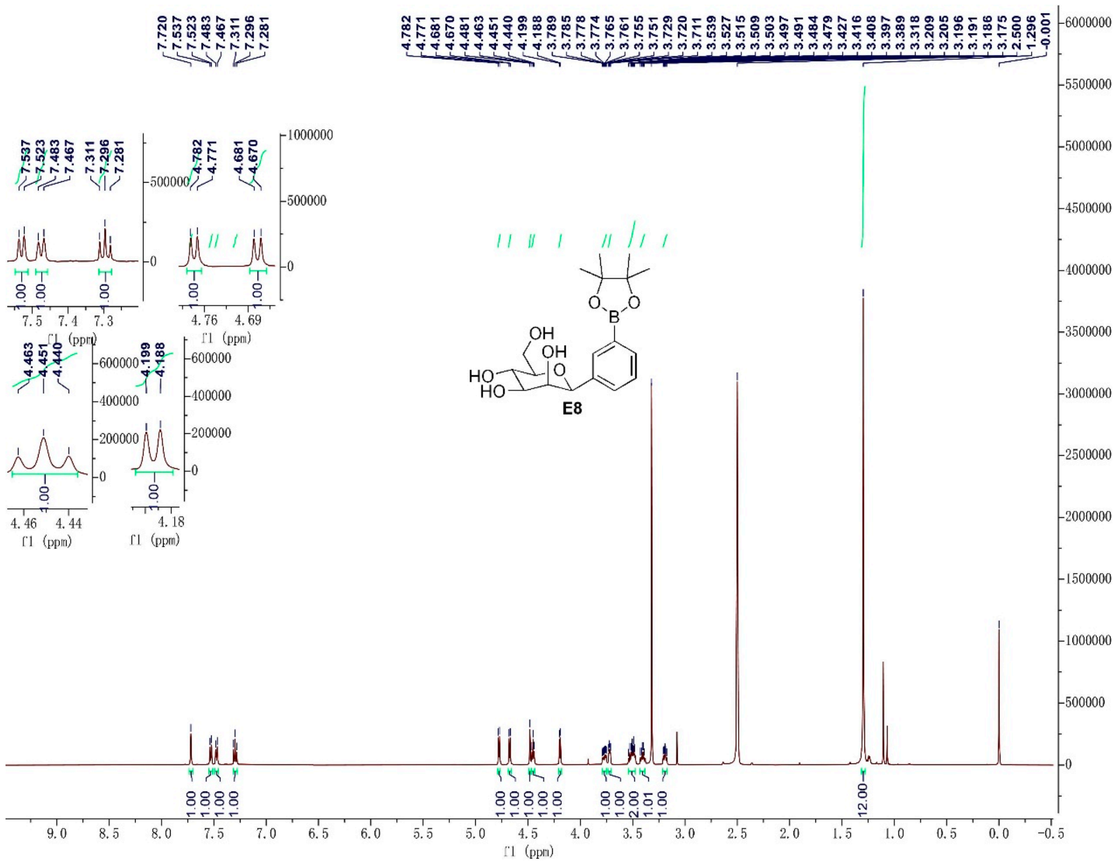

Figure S236 ^{1}H NMR spectrum of **E8**

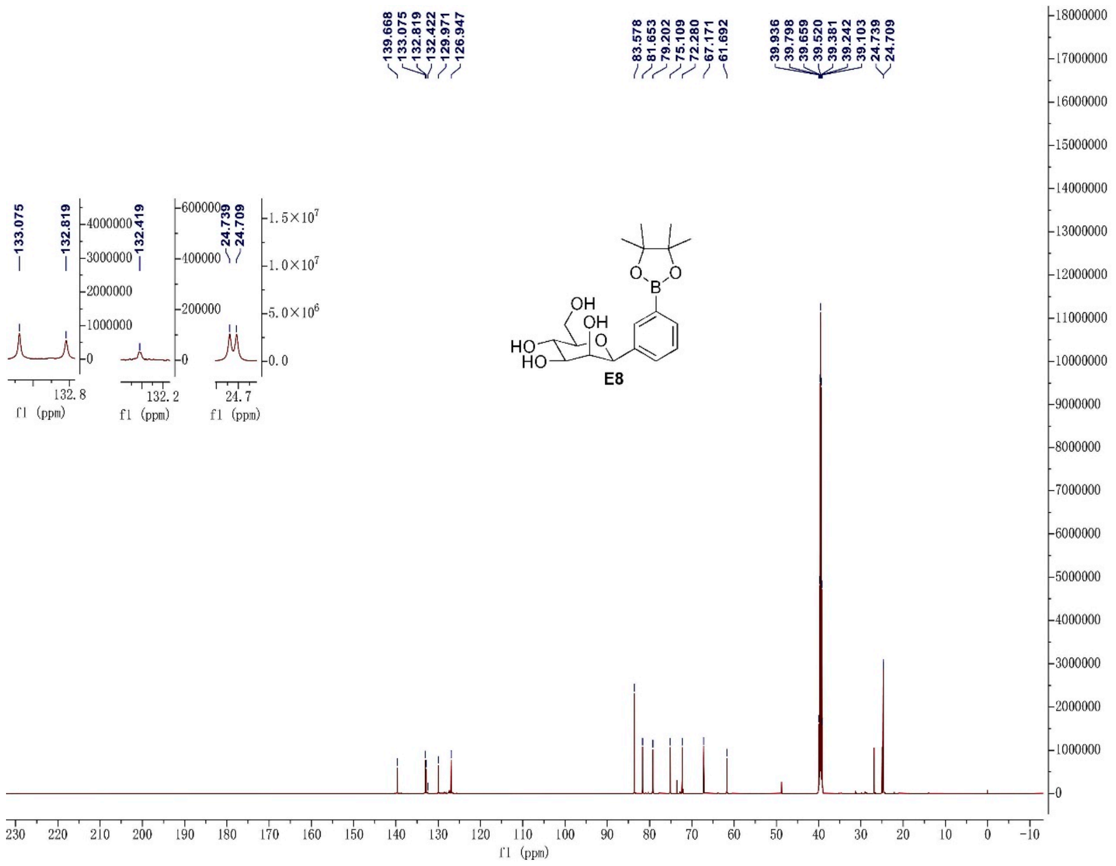

Figure S237 ^{13}C NMR spectrum of **E8**

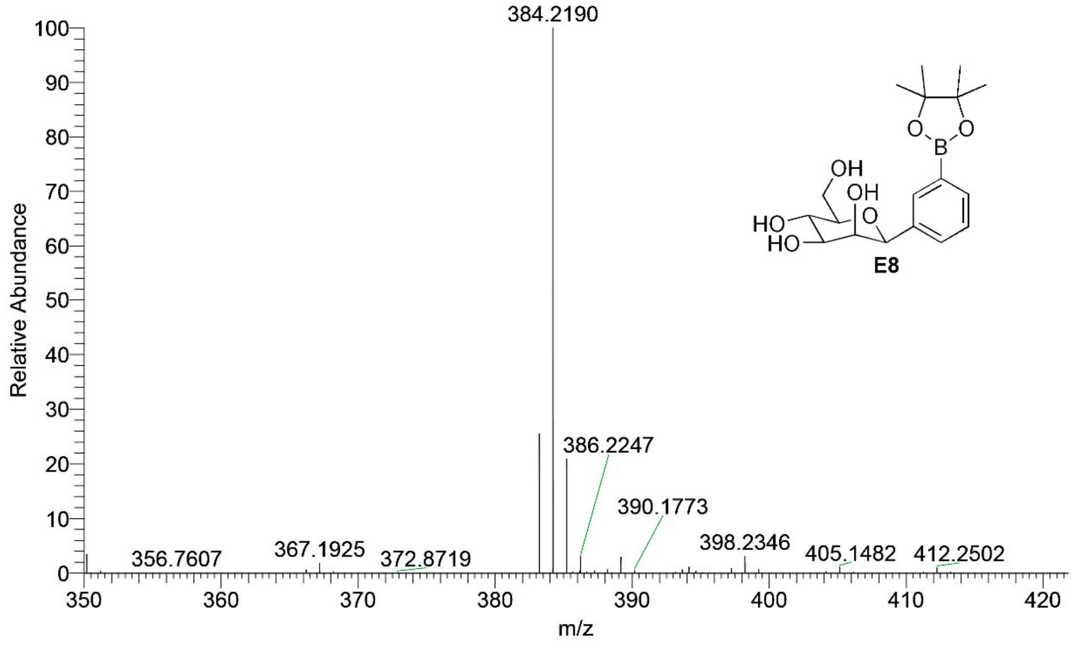

Figure S238 HR-MS (ESI/ion trap) spectrum of **E8**

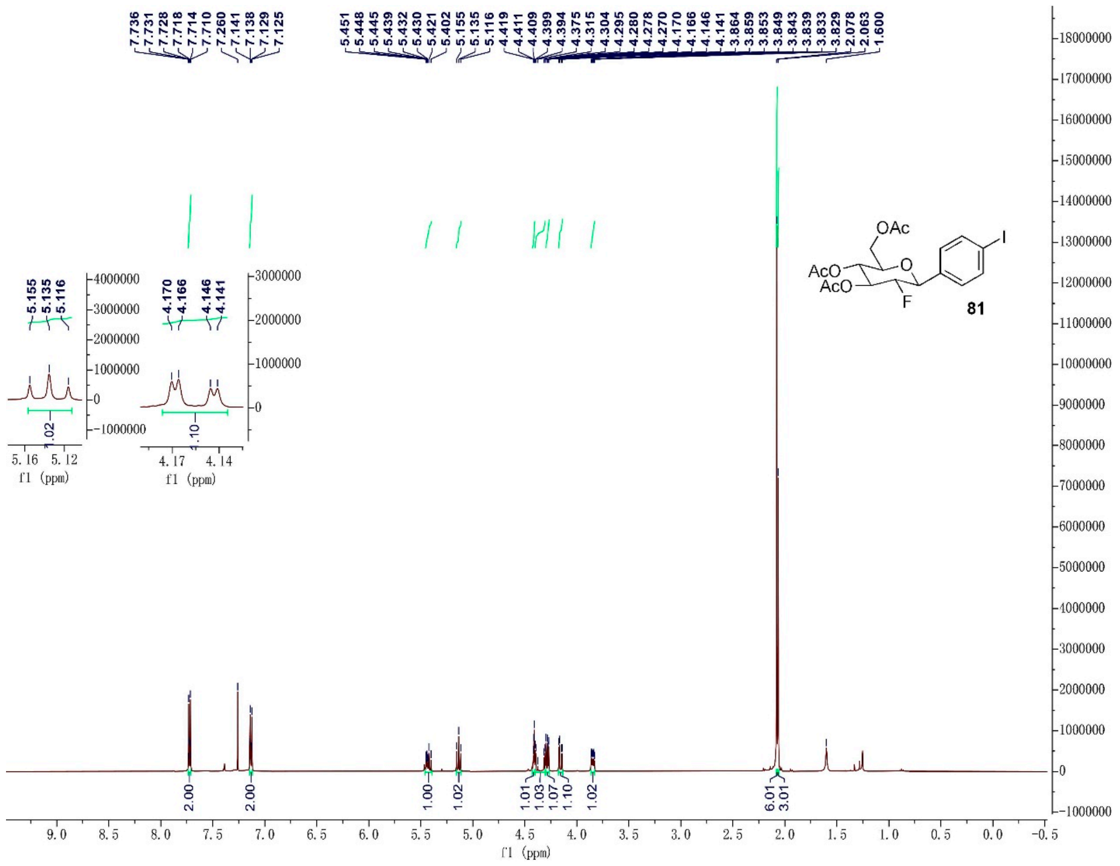

Figure S239 ^{1}H NMR spectrum of **81**

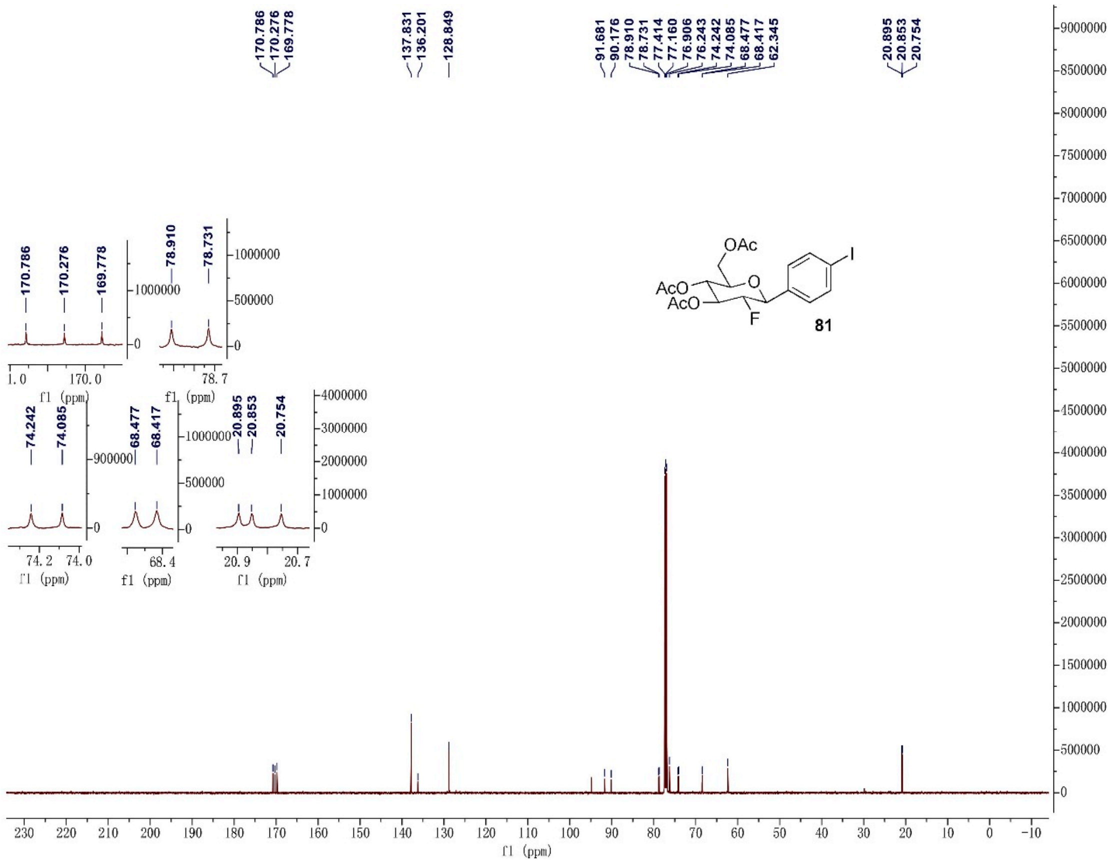

Figure S240 ^{13}C NMR spectrum of **81**

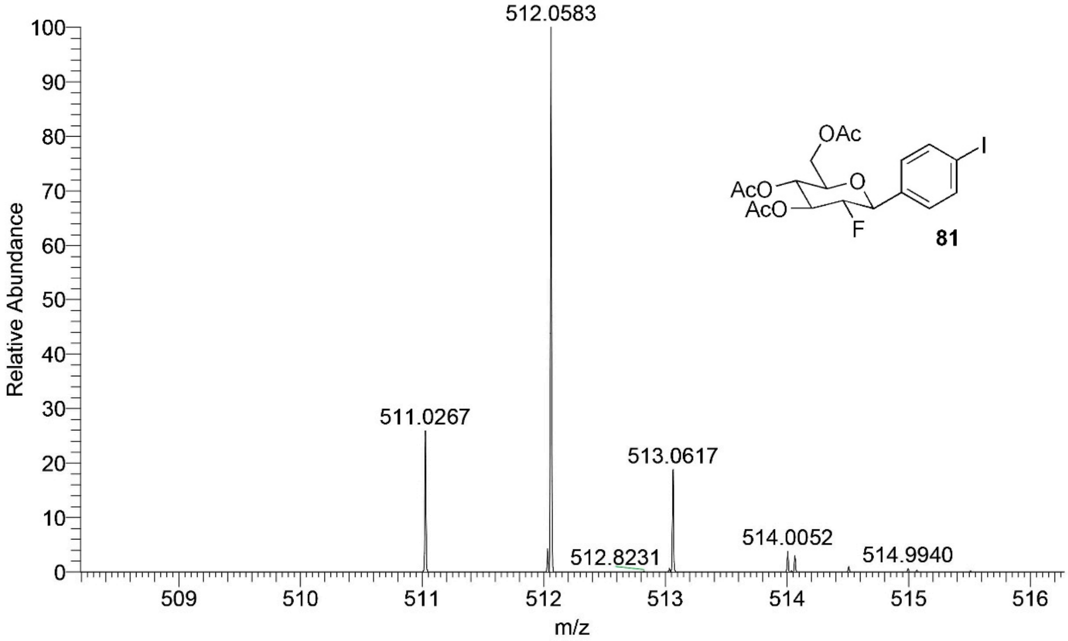

Figure S241 HR-MS (ESI/ion trap) spectrum of **81**

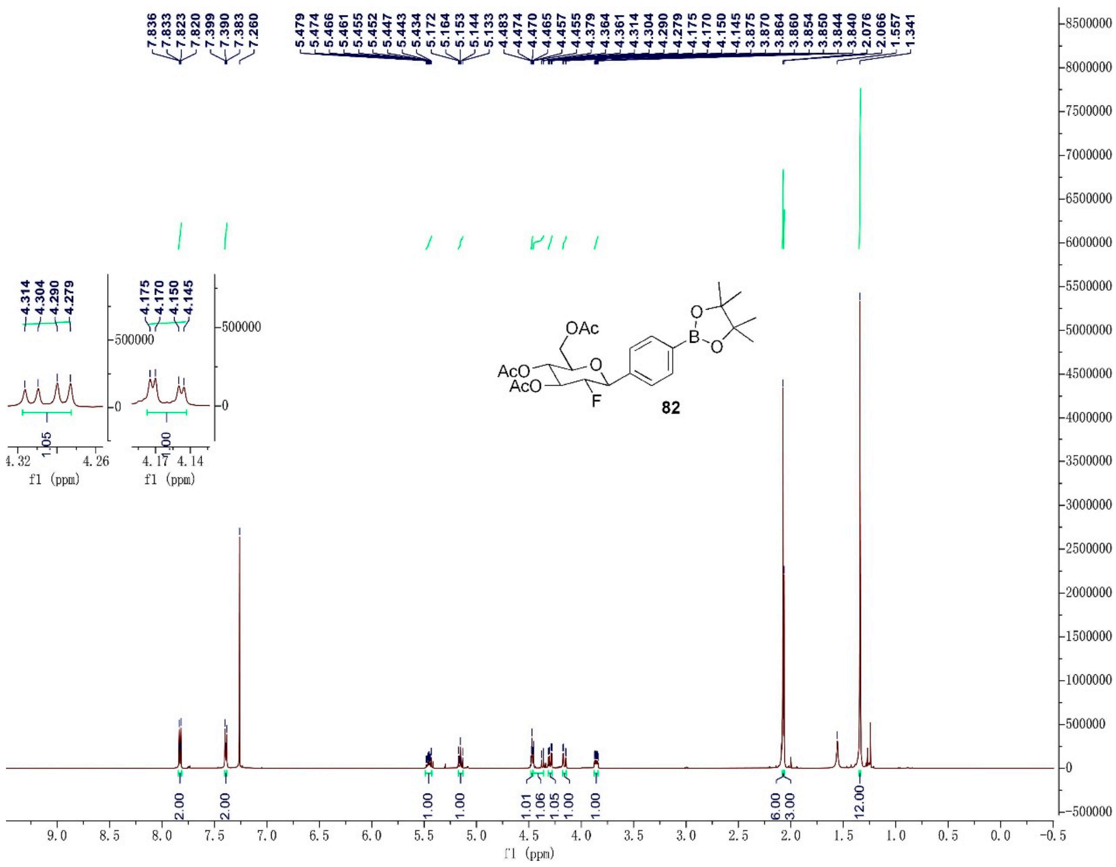

Figure S242 ^{1}H NMR spectrum of **82**

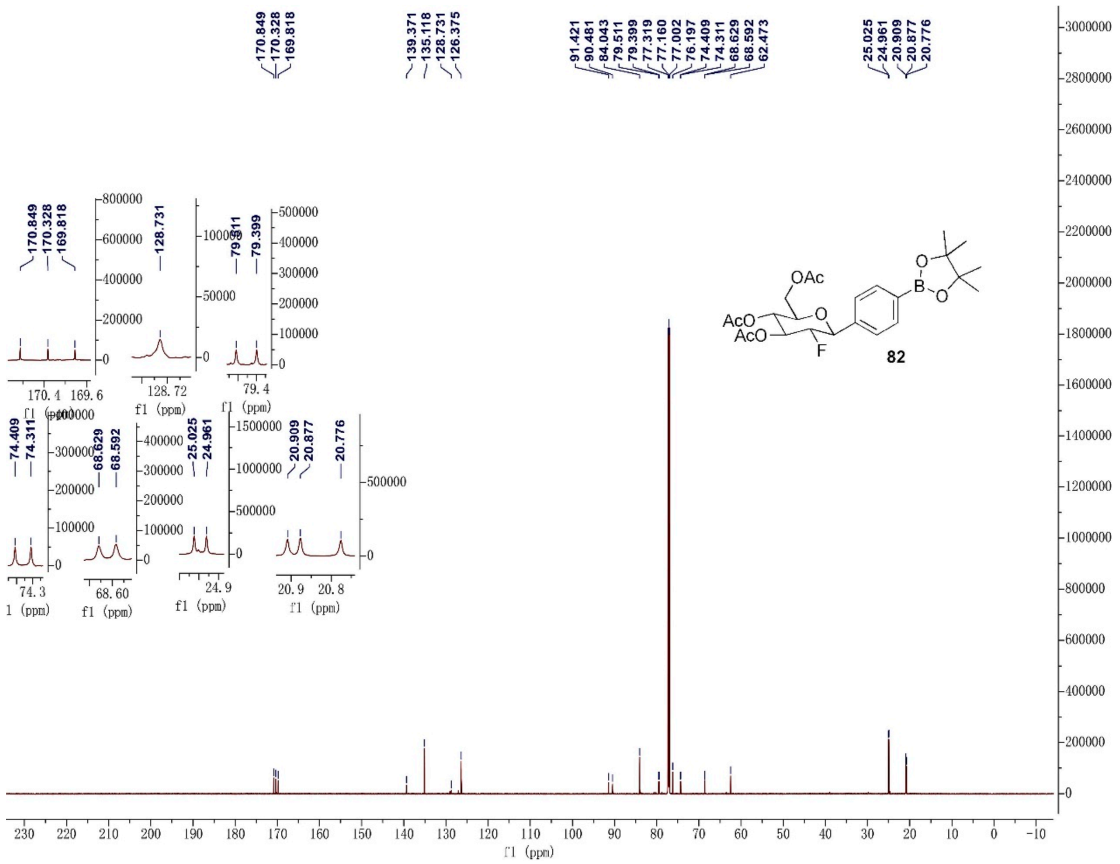

Figure S243 ^{13}C NMR spectrum of **82**

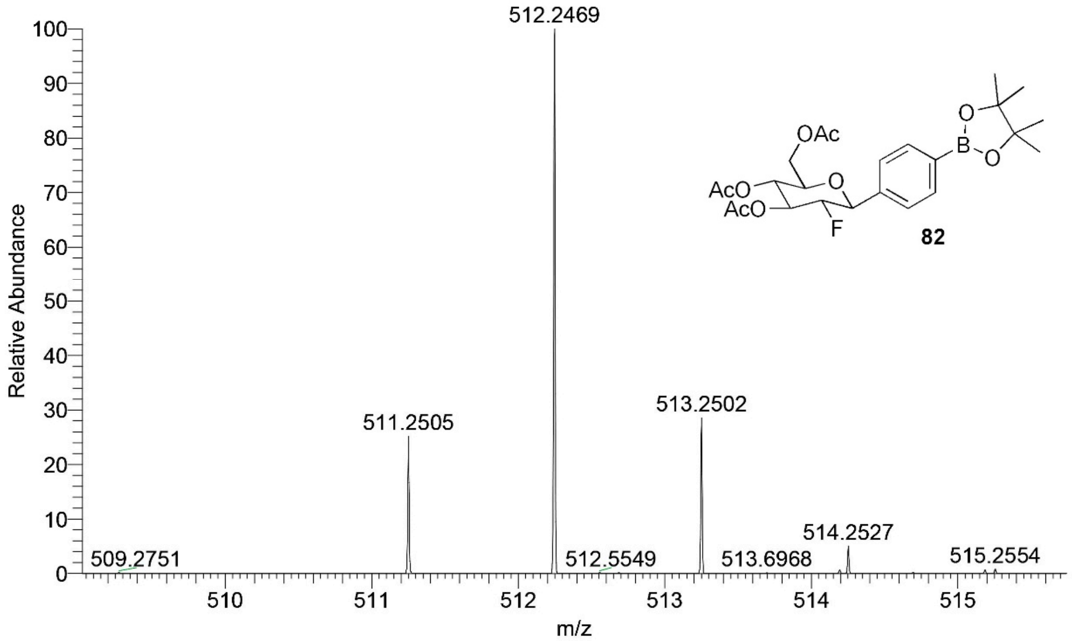

Figure S244 HR-MS (ESI/ion trap) spectrum of **82**

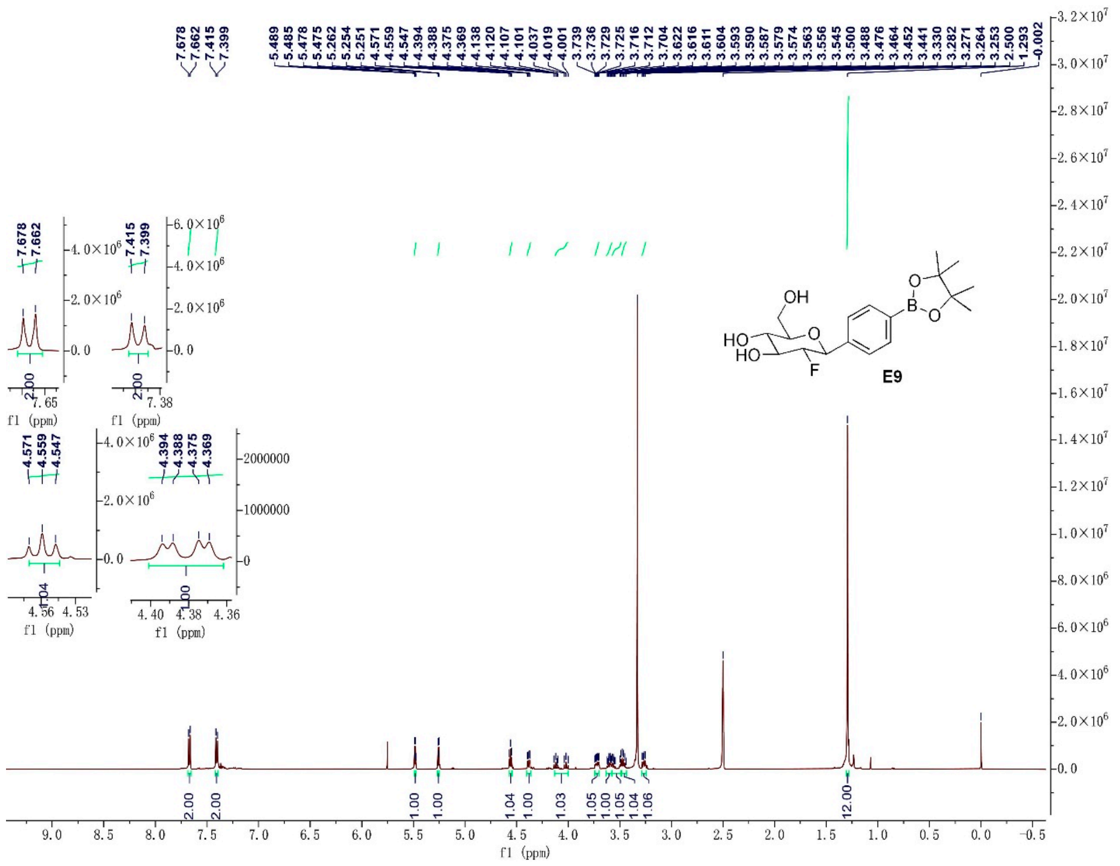

Figure S245 ^{1}H NMR spectrum of **E9**

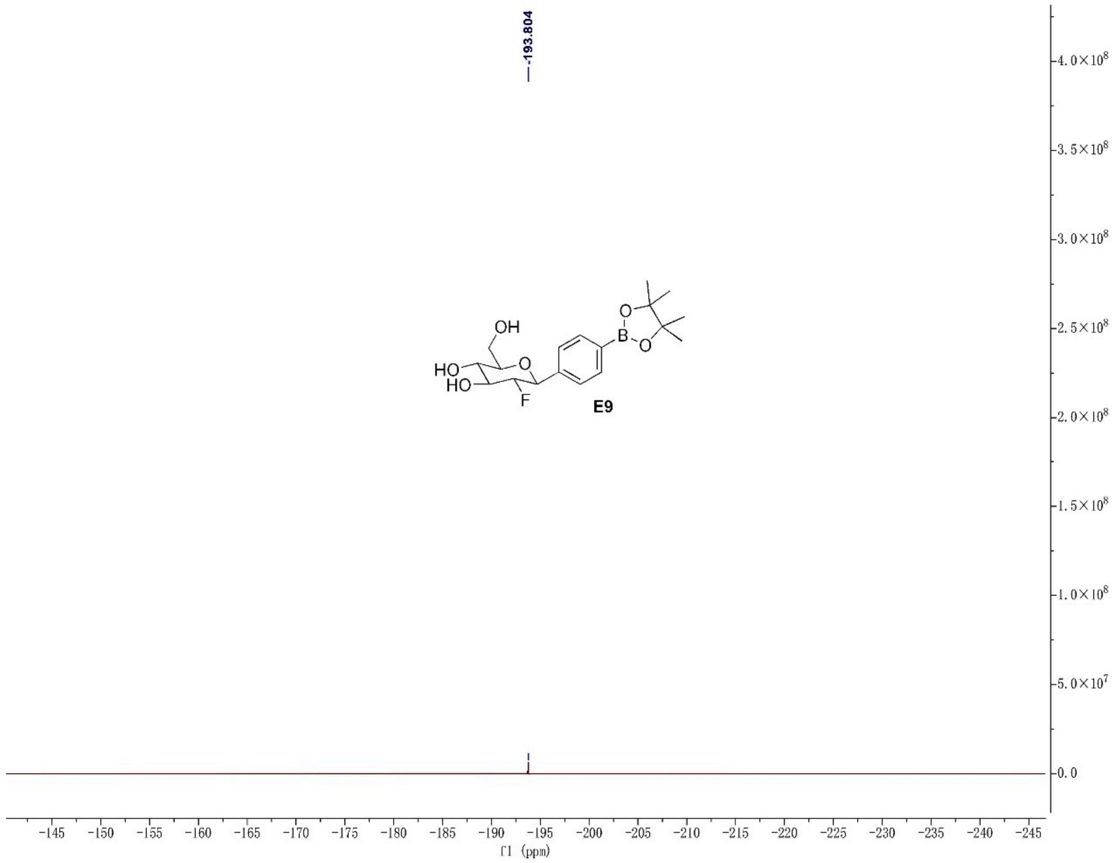

Figure S246 ^{19}F NMR spectrum of **E9**

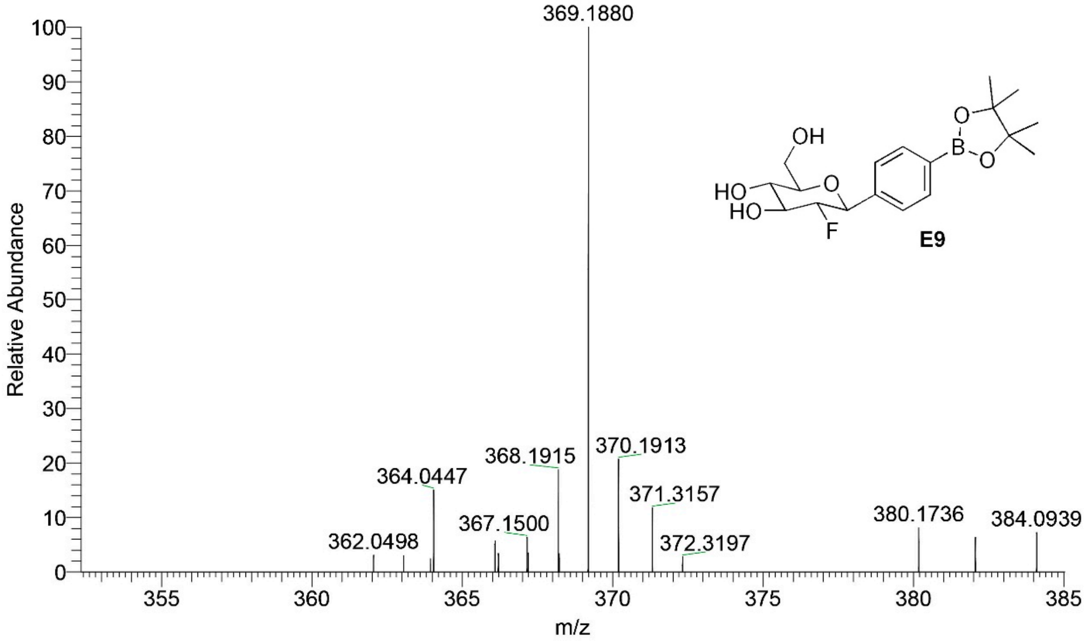

Figure S247 HR-MS (ESI/ion trap) spectrum of **E9**

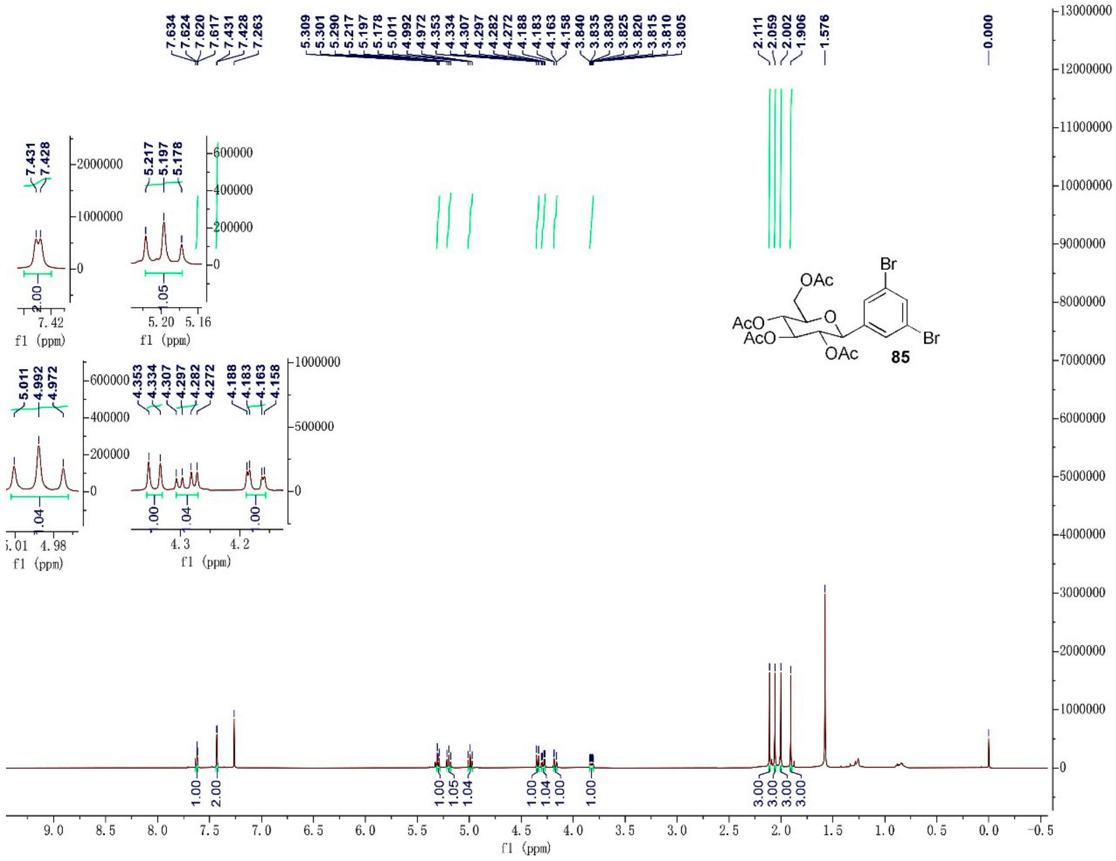

Figure S248 ^{1}H NMR spectrum of **85**

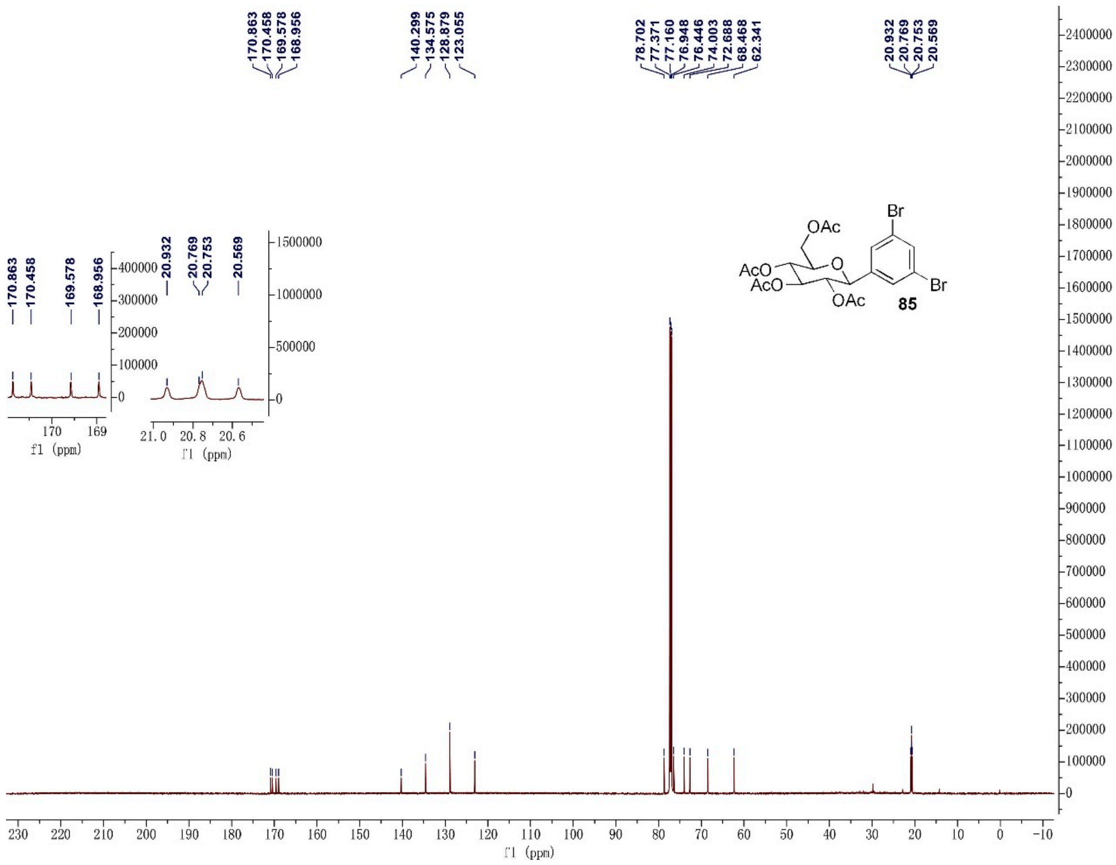

Figure S249 ^{13}C NMR spectrum of **85**

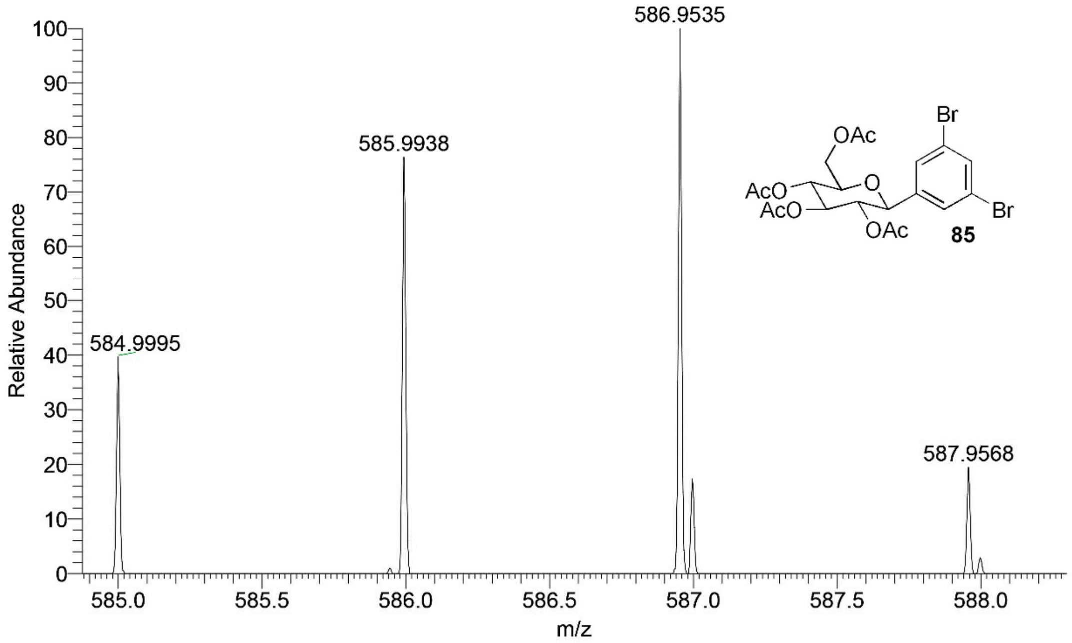

Figure S250 HR-MS (ESI/ion trap) spectrum of **85**

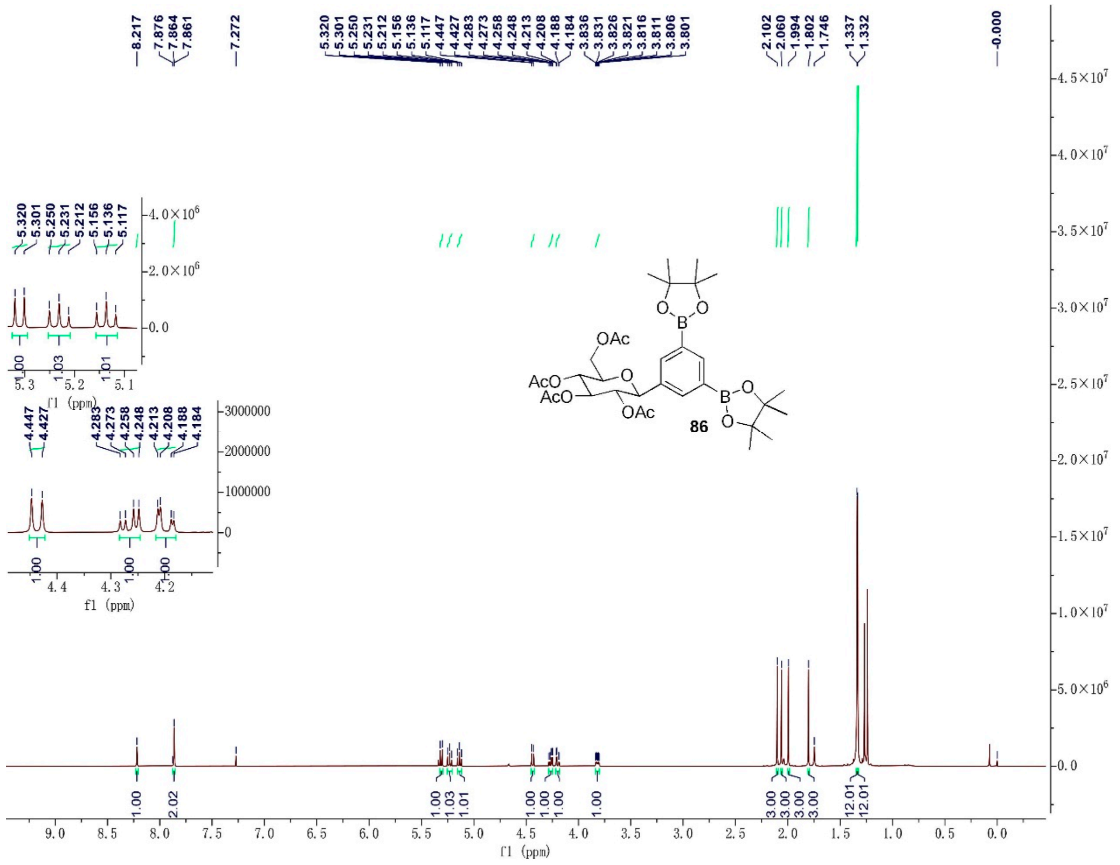

Figure S251 ^{1}H NMR spectrum of **86**

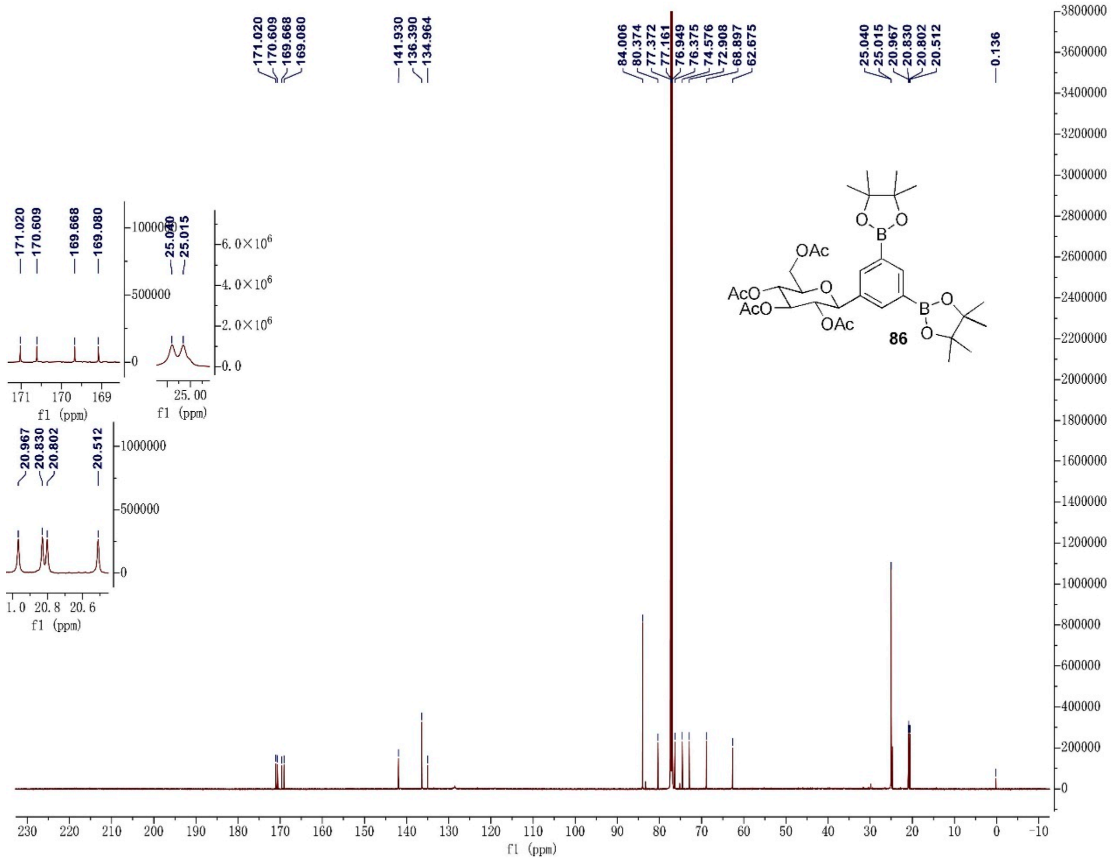

Figure S252 ^{13}C NMR spectrum of **86**

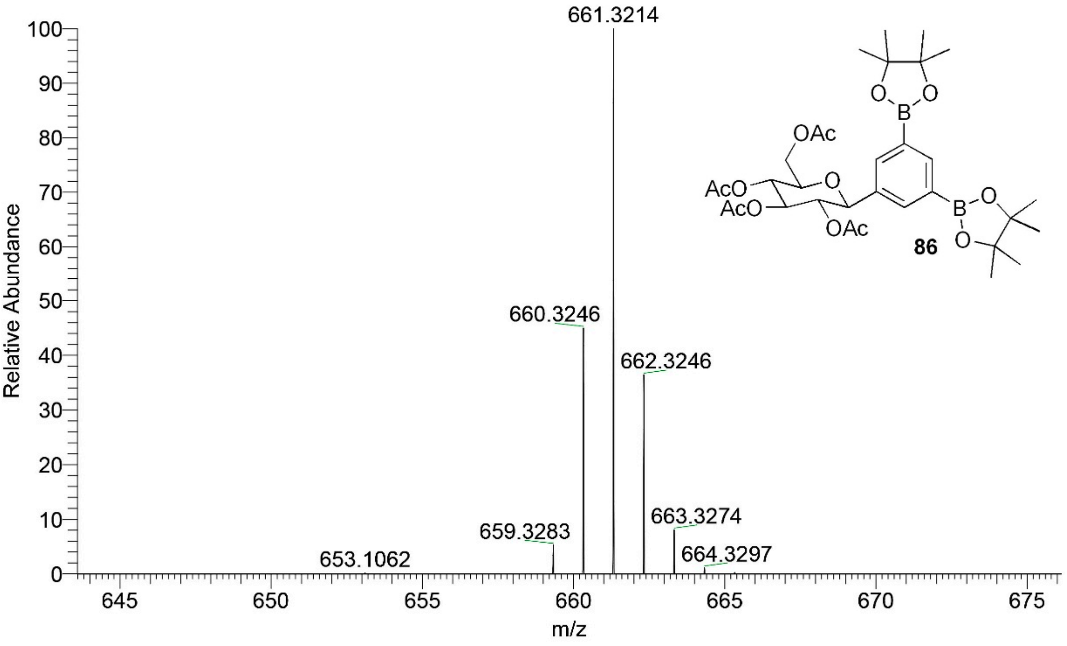

Figure S253 HR-MS (ESI/ion trap) spectrum of **86**

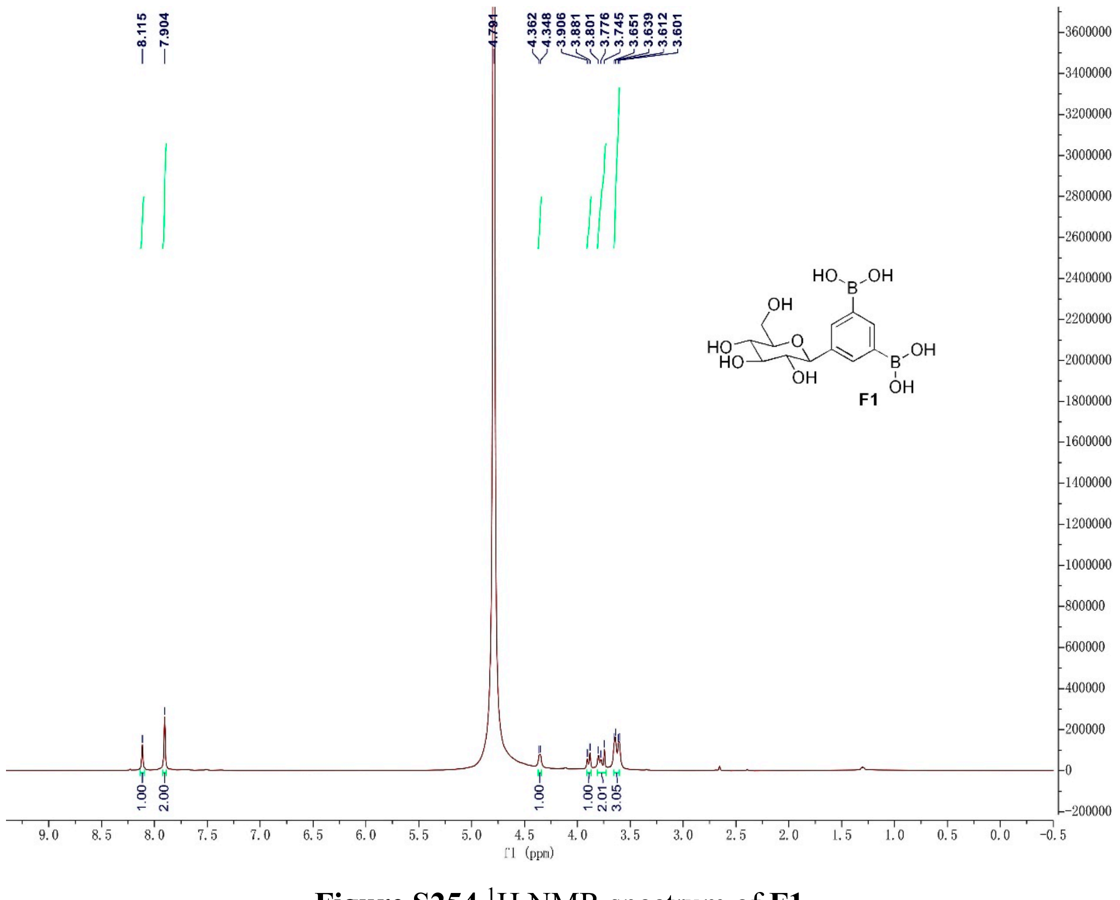

Figure S254 ^{1}H NMR spectrum of **F1**

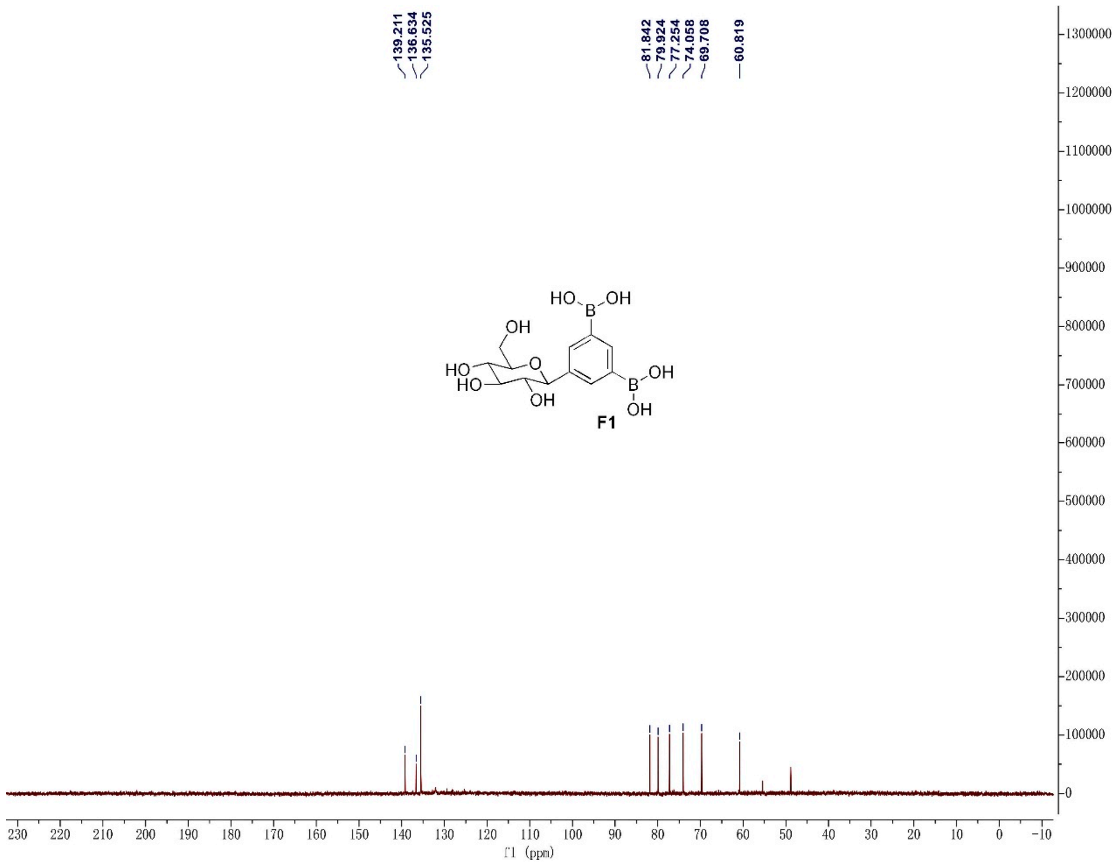

Figure S255 ^{13}C NMR spectrum of **F1**

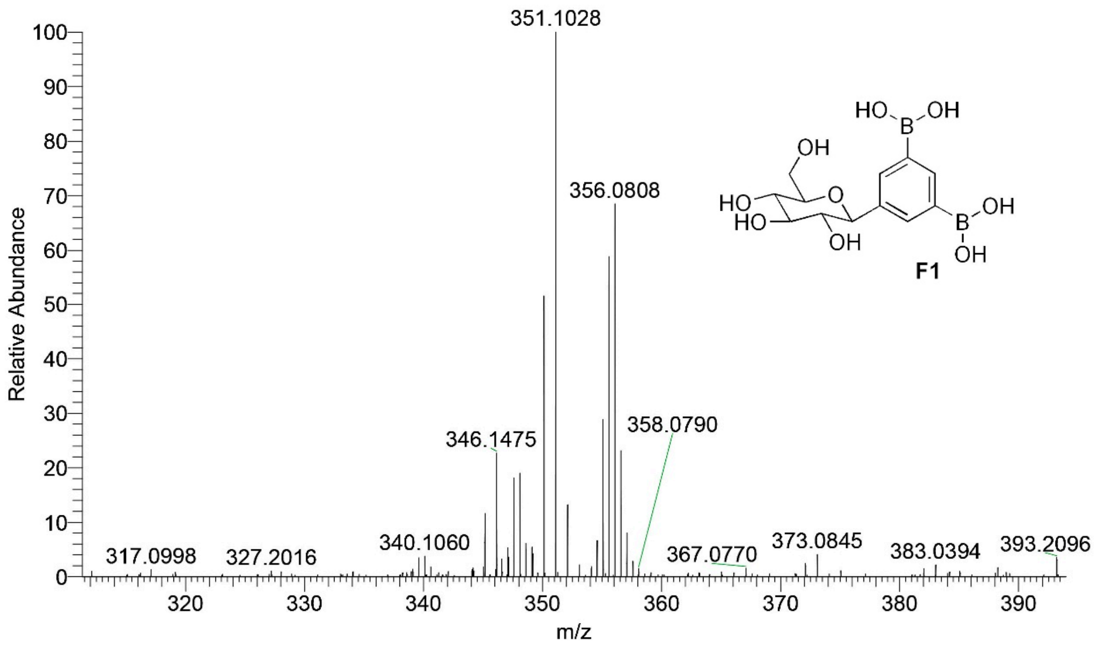

Figure S256 HR-MS (ESI/ion trap) spectrum of **F1**

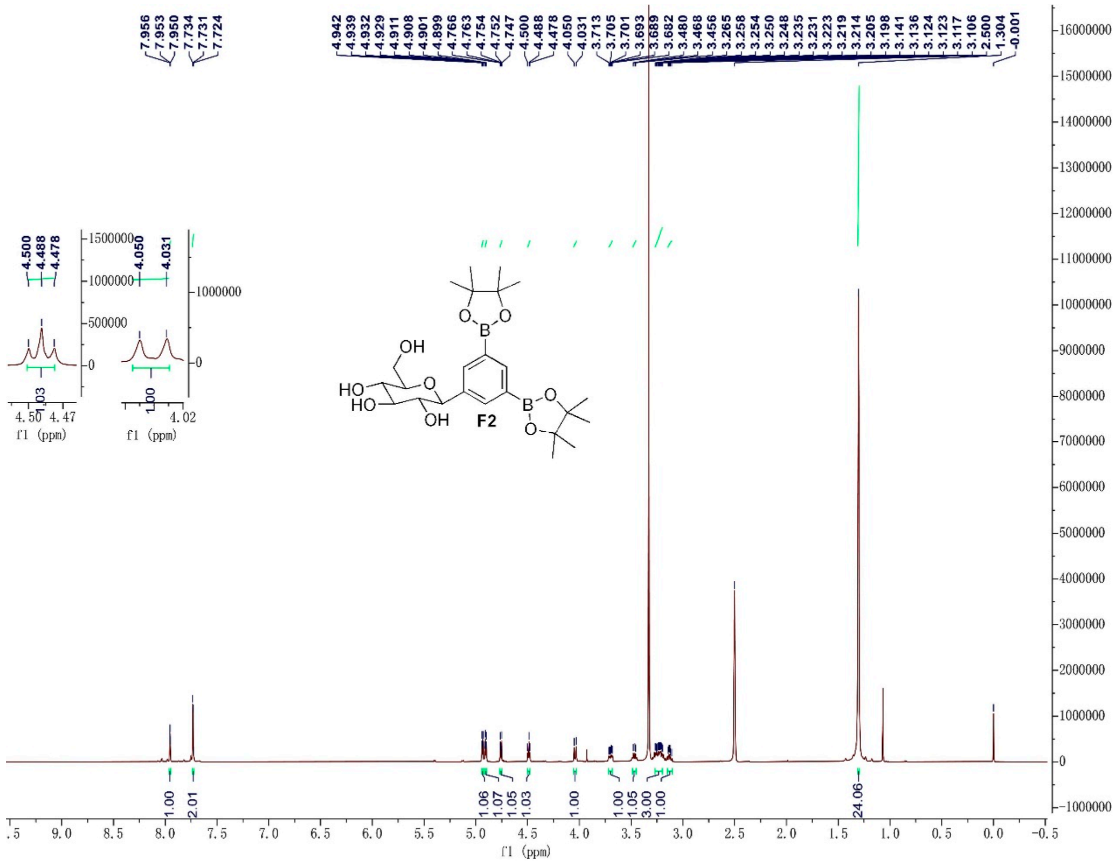

Figure S257 ^{1}H NMR spectrum of **F2**

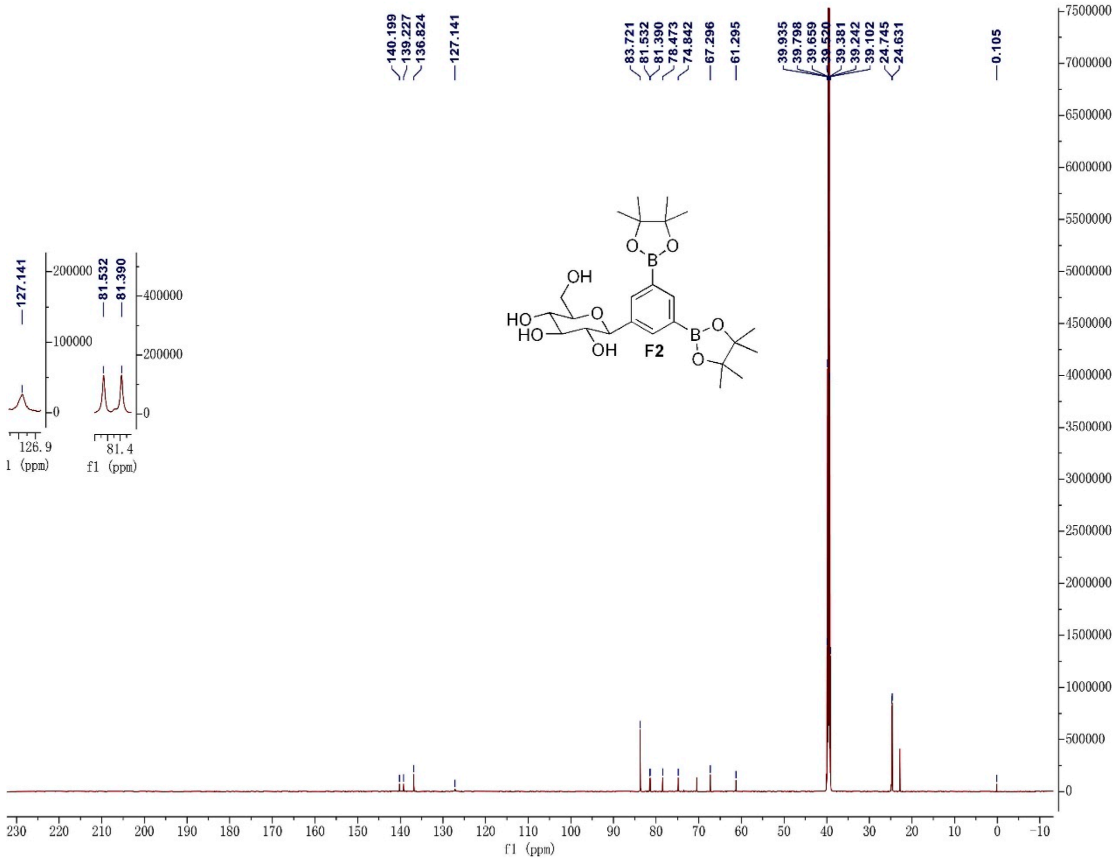

Figure S258 ^{13}C NMR spectrum of **F2**

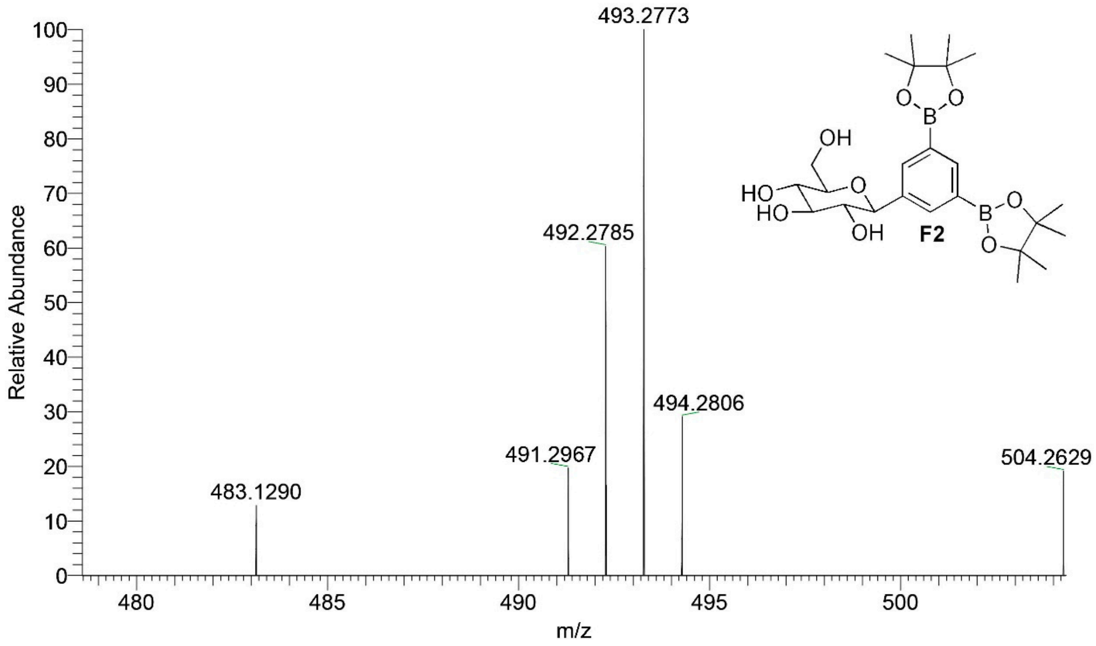

Figure S259 HR-MS (ESI/ion trap) spectrum of **F2**

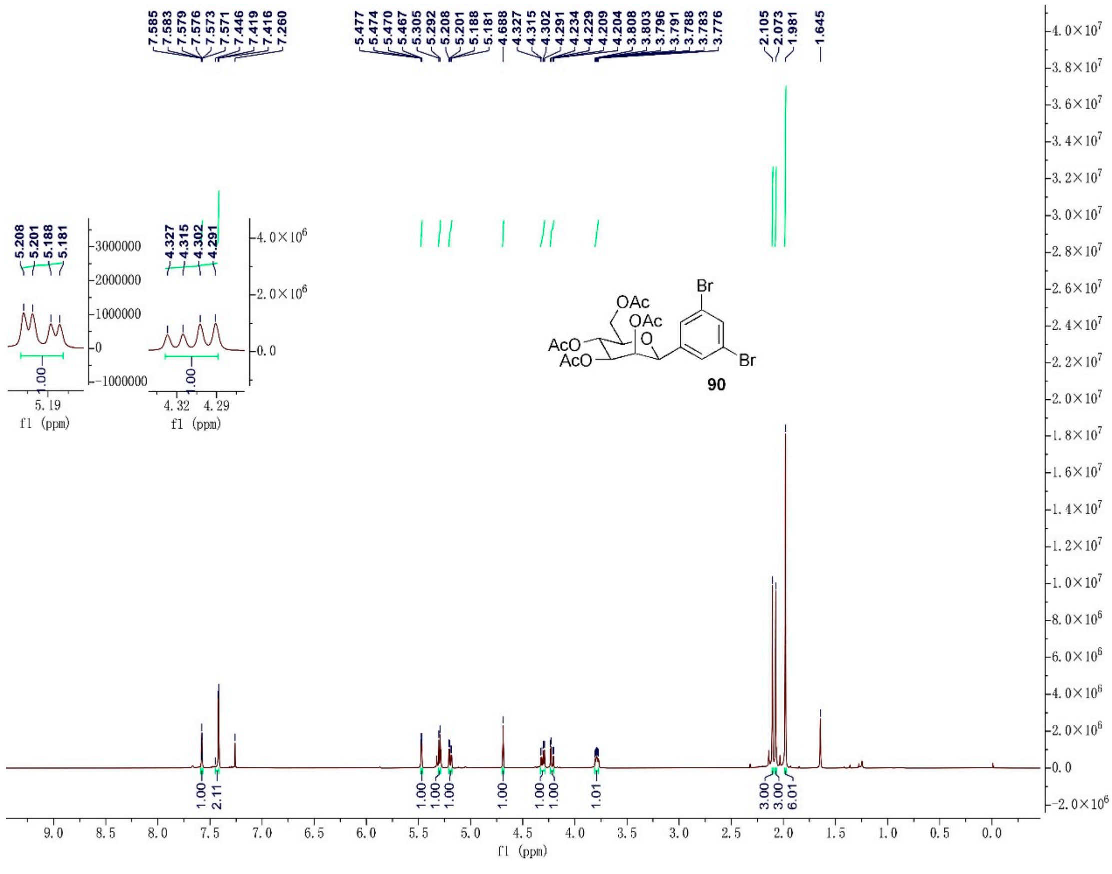

Figure S260 ^{1}H NMR spectrum of **90**

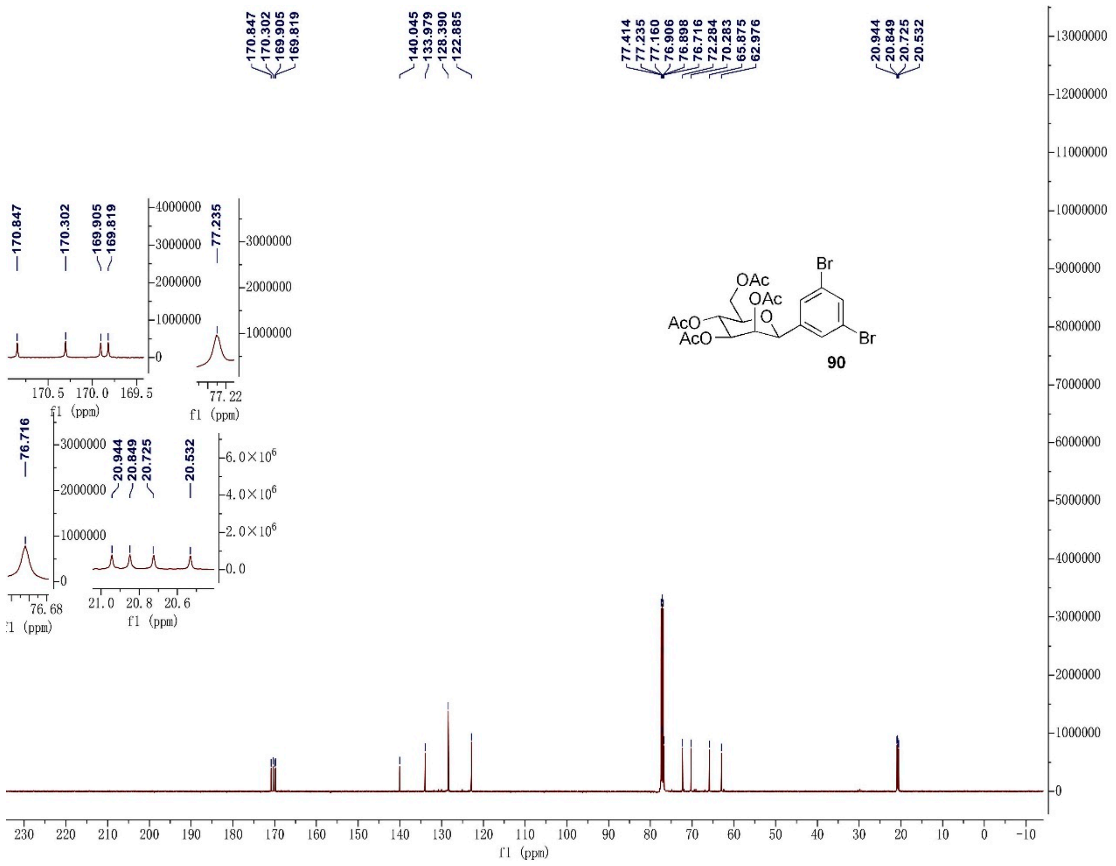

Figure S261 ^{13}C NMR spectrum of **90**

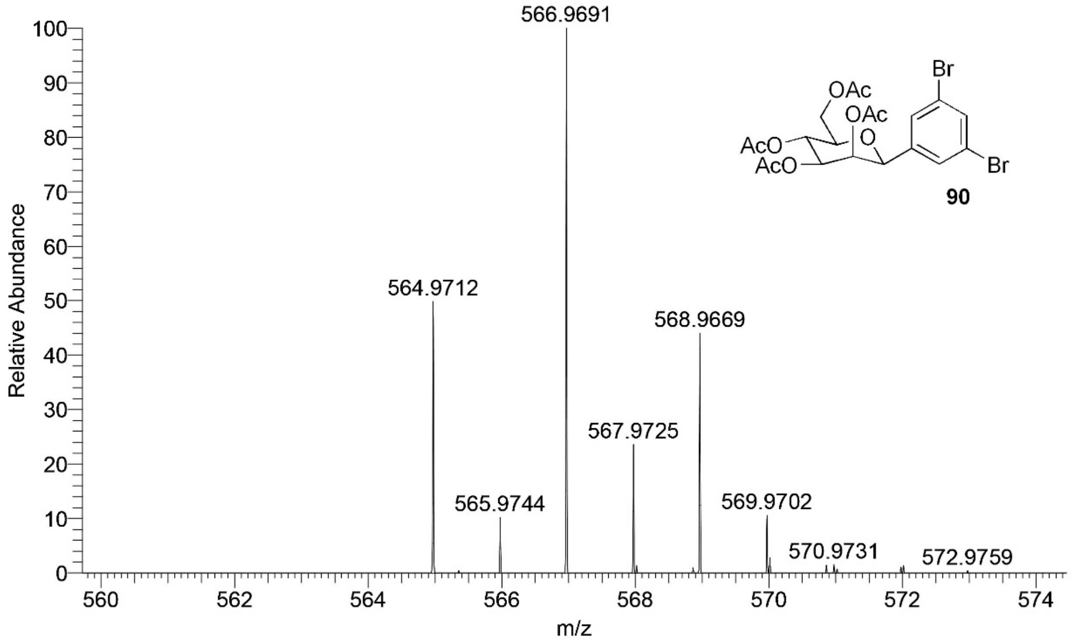

Figure S262 HR-MS (ESI/ion trap) spectrum of **90**

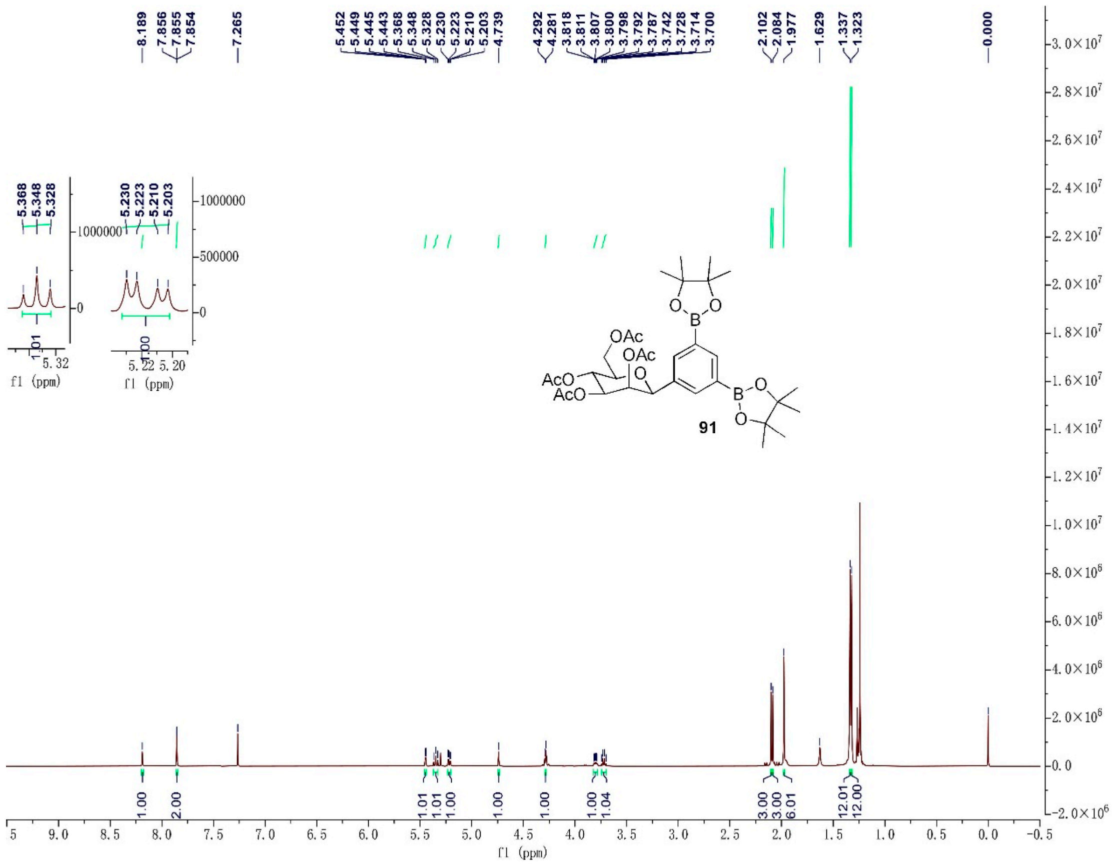

Figure S263 ^{1}H NMR spectrum of **91**

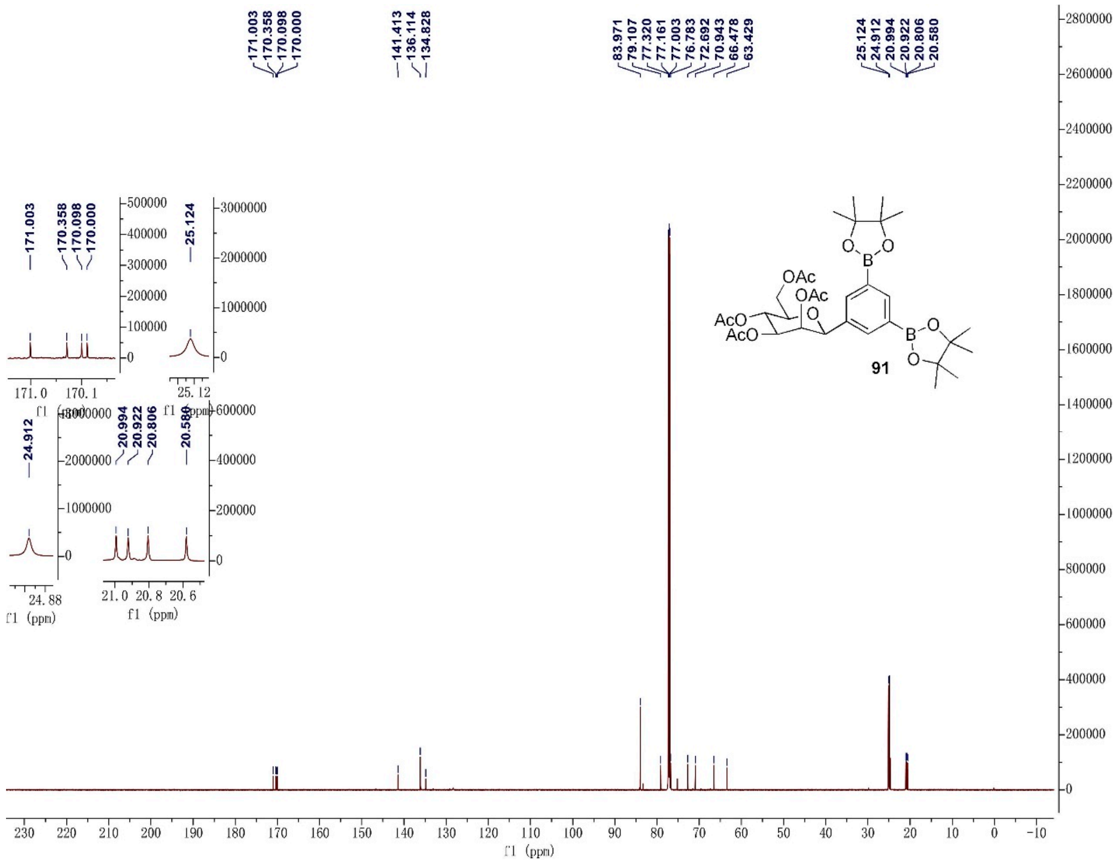

Figure S264 ^{13}C NMR spectrum of **91**

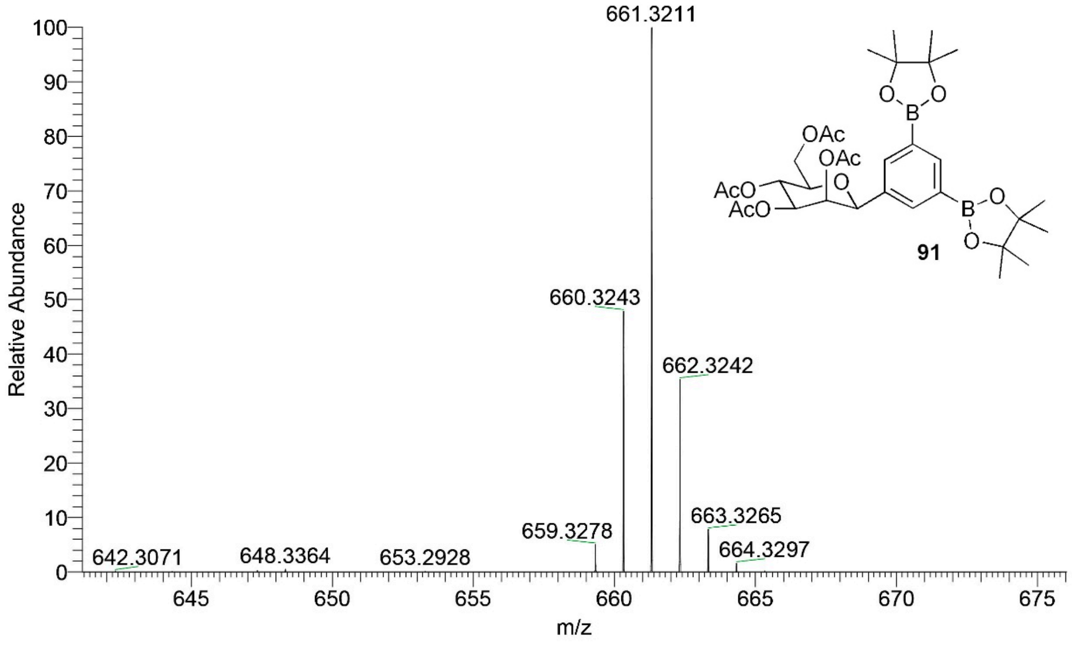

Figure S265 HR-MS (ESI/ion trap) spectrum of **91**

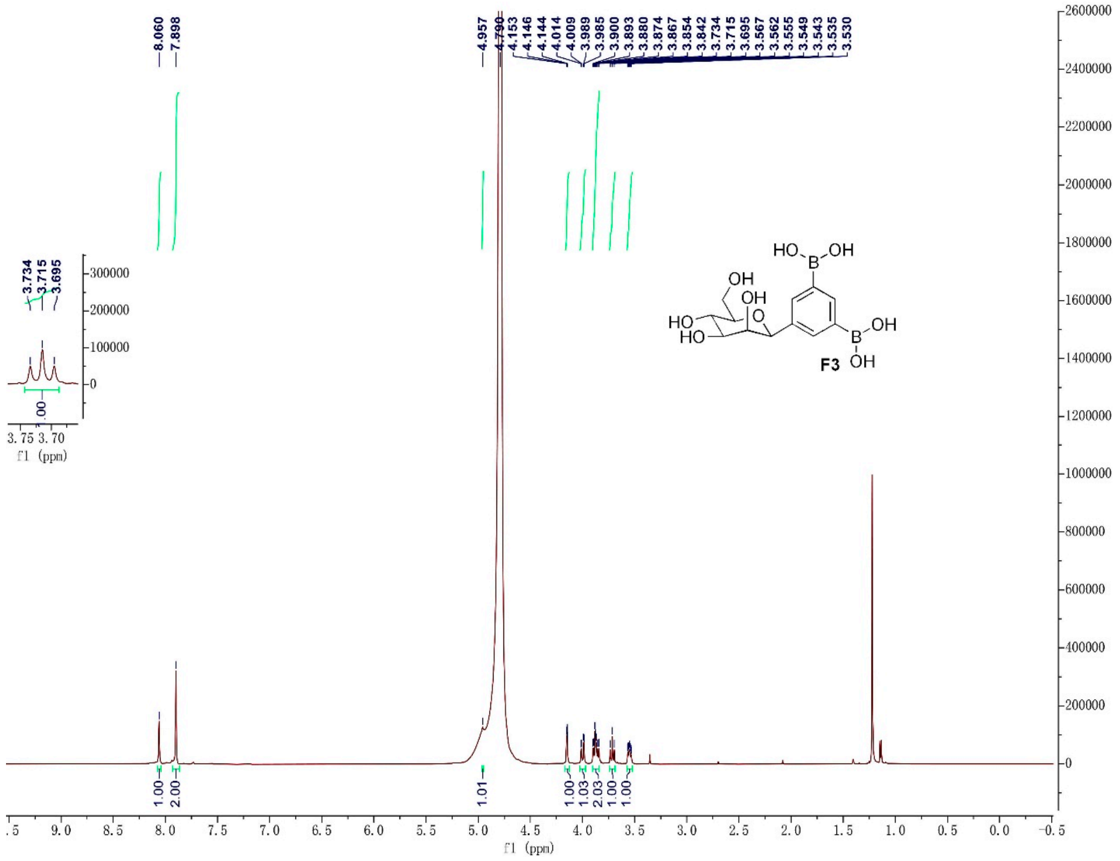

Figure S266 ^{1}H NMR spectrum of **F3**

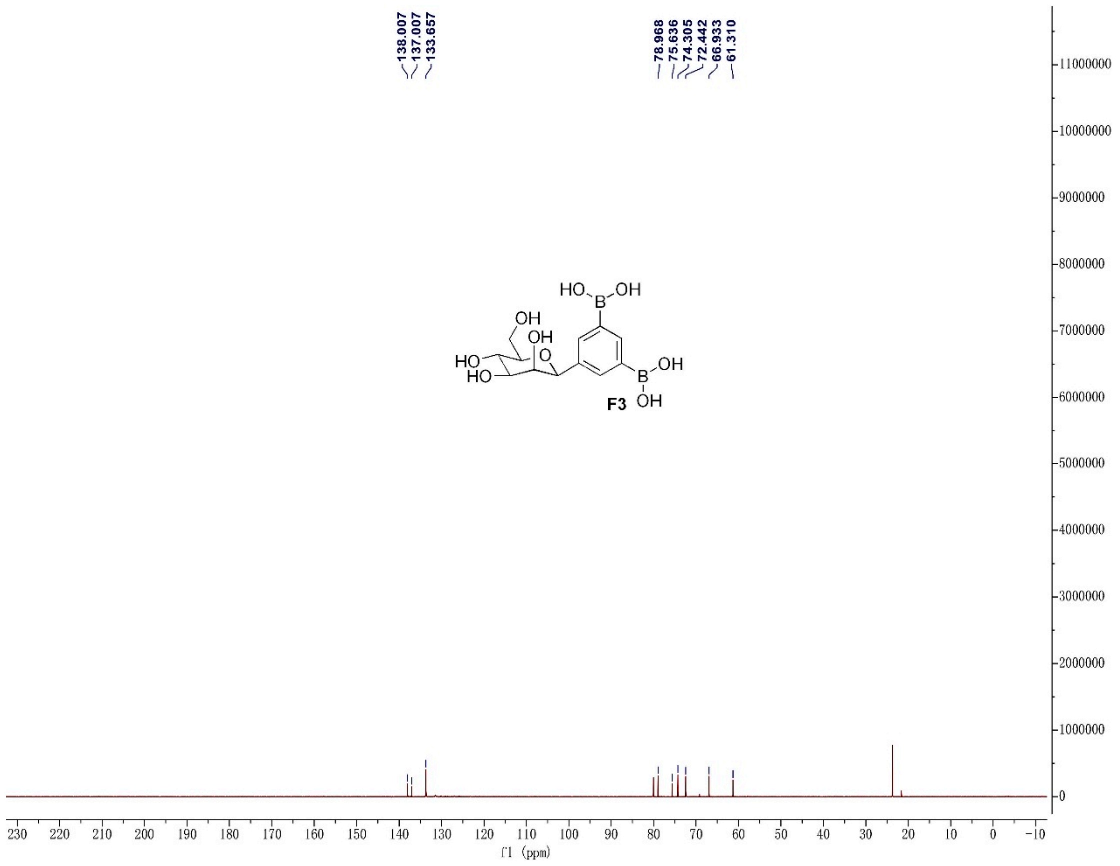

Figure S267 ^{13}C NMR spectrum of **F3**

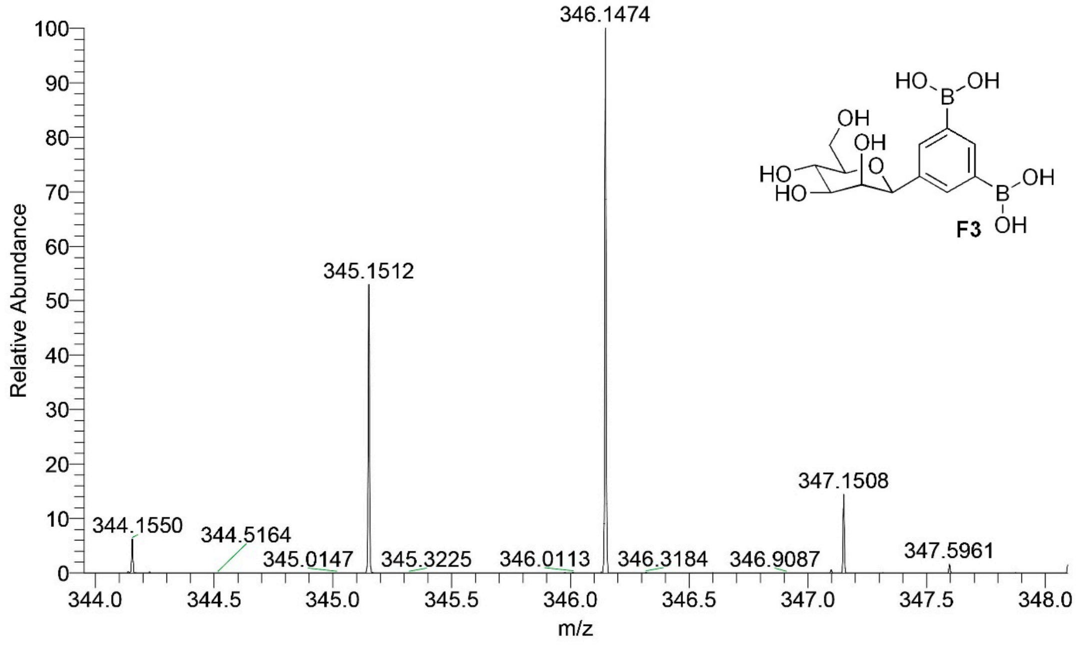

Figure S268 HR-MS (ESI/ion trap) spectrum of **F3**

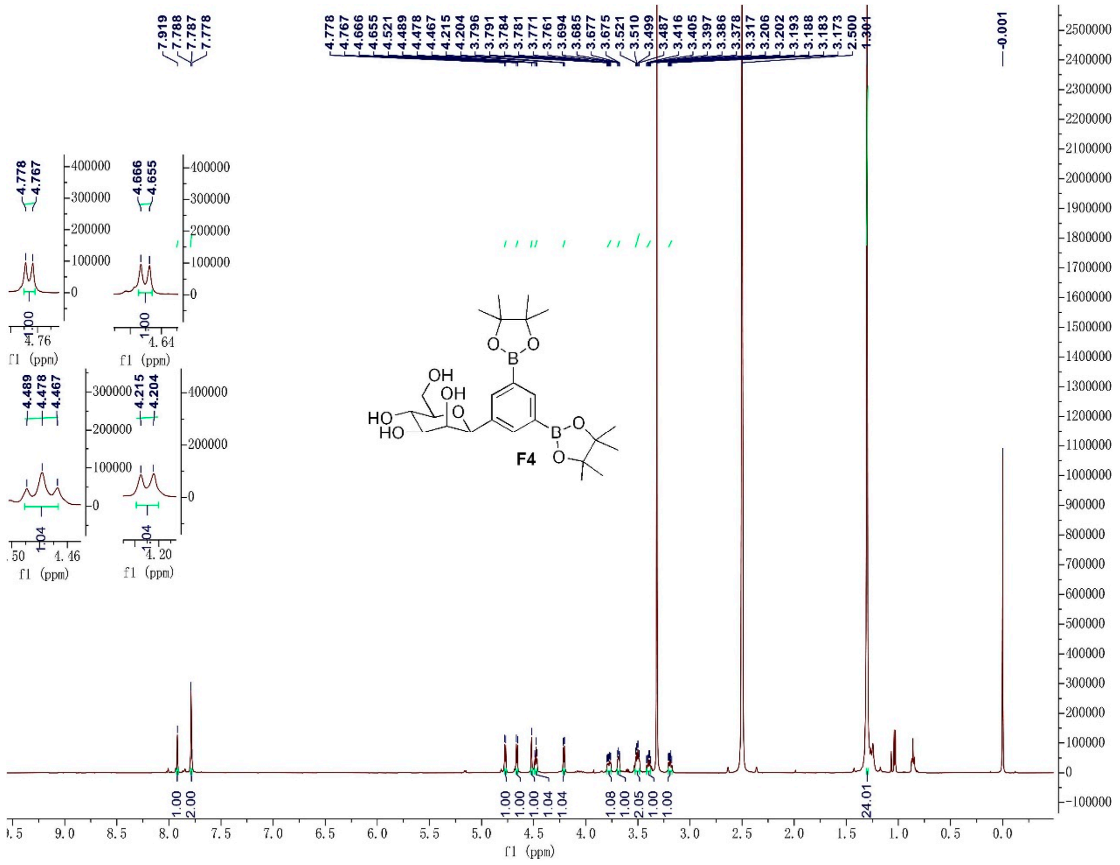

Figure S269 ^{1}H NMR spectrum of **F4**

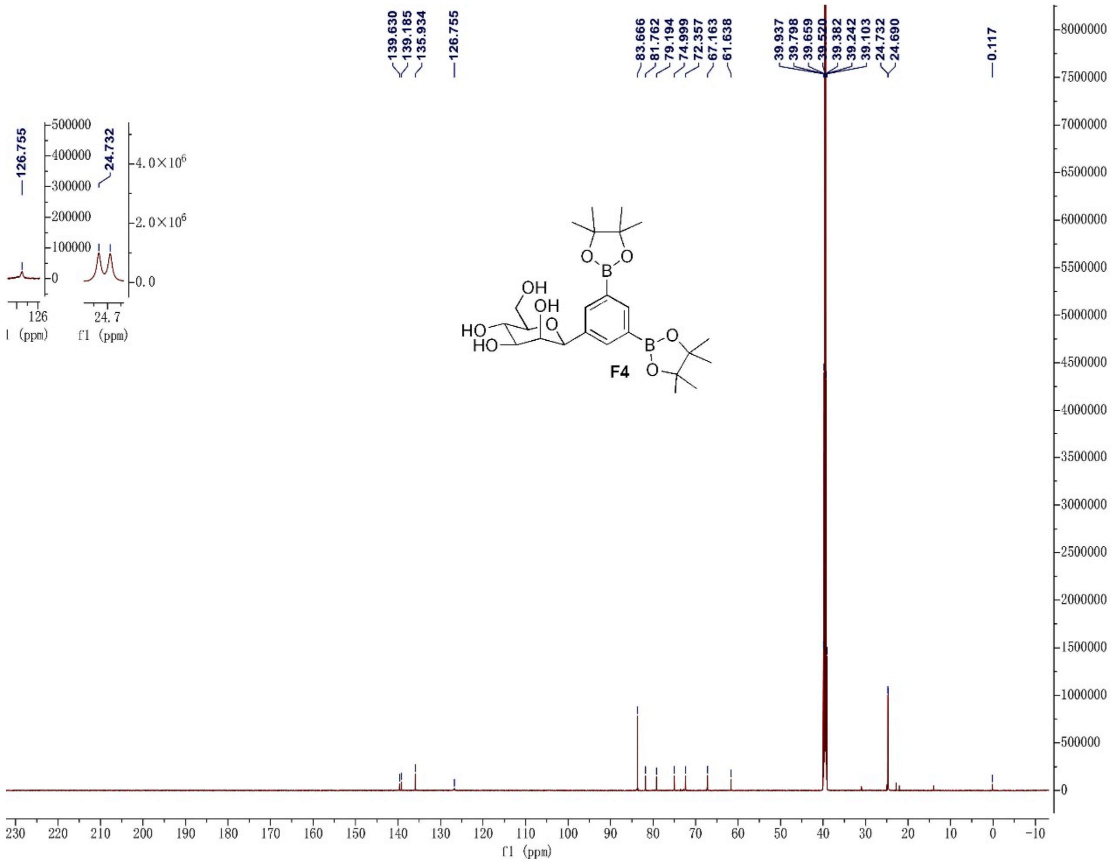

Figure S270 ^{13}C NMR spectrum of **F4**

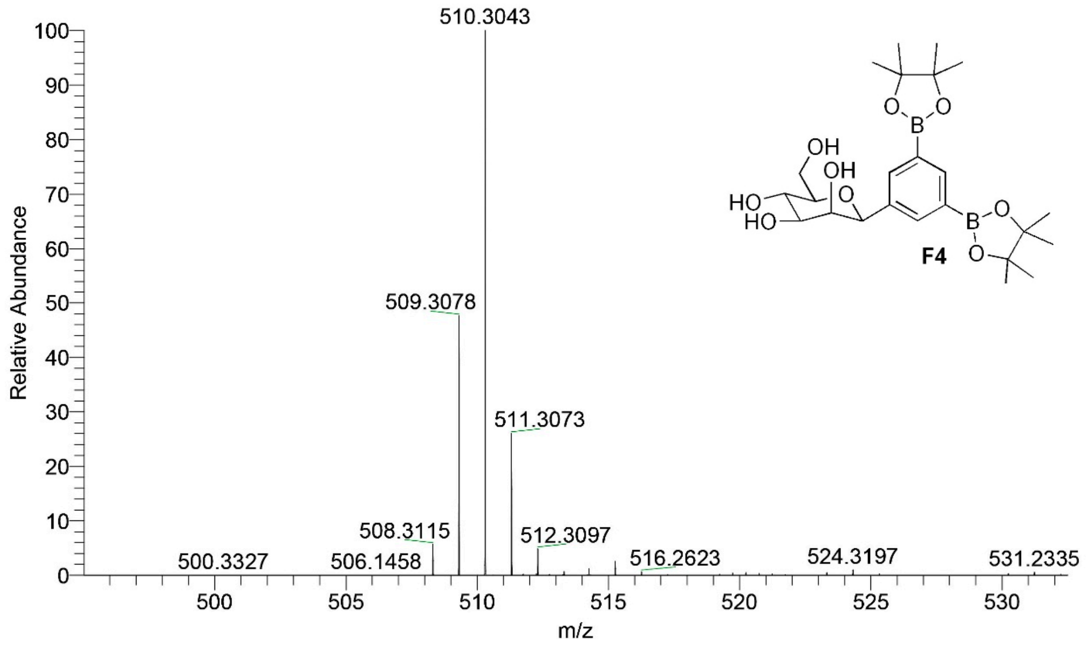

Figure S271 HR-MS (ESI/ion trap) spectrum of **F4**
